# Iridium-catalyzed enantioselective synthesis of chiral γ-amino alcohols and intermediates of (*S*)-duloxetine, (*R*)-fluoxetine, and (*R*)-atomoxetine

**DOI:** 10.1038/s42004-022-00678-4

**Published:** 2022-05-19

**Authors:** Chengyu Liu, Lei Zhang, Liming Cao, Yan Xiong, Yueyue Ma, Ruihua Cheng, Jinxing Ye

**Affiliations:** 1 Engineering Research Centre of Pharmaceutical Process Chemistry, Ministry of Education, Shanghai Key Laboratory of New Drug Design, School of Pharmacy, East China University of Science and Technology, Shanghai, China; 2 School of Chemical Engineering, East China University of Science and Technology, Shanghai, China; 3 School of Biomedical and Pharmaceutical Sciences, Guangdong University of Technology, Guangzhou, China

**Keywords:** Asymmetric catalysis, Synthetic chemistry methodology, Asymmetric synthesis

## Abstract

Chiral γ-amino alcohols are the prevalent structural motifs and building blocks in pharmaceuticals and bioactive molecules. Enantioselective hydrogenation of β-amino ketones provides a straightforward and powerful tool for the synthesis of chiral γ-amino alcohols, but the asymmetric transformation is synthetically challenging. Here, a series of tridentate ferrocene-based phosphine ligands bearing modular and tunable unsymmetrical vicinal diamine scaffolds were designed, synthesized, and evaluated in the iridium-catalyzed asymmetric hydrogenation of β-amino ketones. The system was greatly effective to substrates with flexible structure and functionality, and diverse β-tertiary-amino ketones and β-secondary-amino ketones were hydrogenated smoothly. The excellent reactivities and enantioselectivities were achieved in the asymmetric delivery of various chiral γ-amino alcohols with up to 99% yields, >99% ee values, and turnover number (TON) of 48,500. The gram-scale reactions with low catalyst loading showed the potential application in industrial synthesis of chiral drugs, such as (*S*)-duloxetine, (*R*)-fluoxetine, and (*R*)-atomoxetine.

## Abstract (web summary)

Enantioselective hydrogenation of β-amino ketones is a powerful tool to produce bioactive molecules, but their asymmetric transformation is synthetically challenging. Here, an iridium-catalysed system with tridentate ferrocene-based phosphine ligands bearing unsymmetrical vicinal diamine scaffolds is developed for the efficient asymmetric synthesis of diverse γtertiary-amino and γ-secondary-amino alcohols, including intermediates of (S)-duloxetine, (R)-fluoxetine and (R)-atomoxetine.

## Introduction

**Fig. 1:**
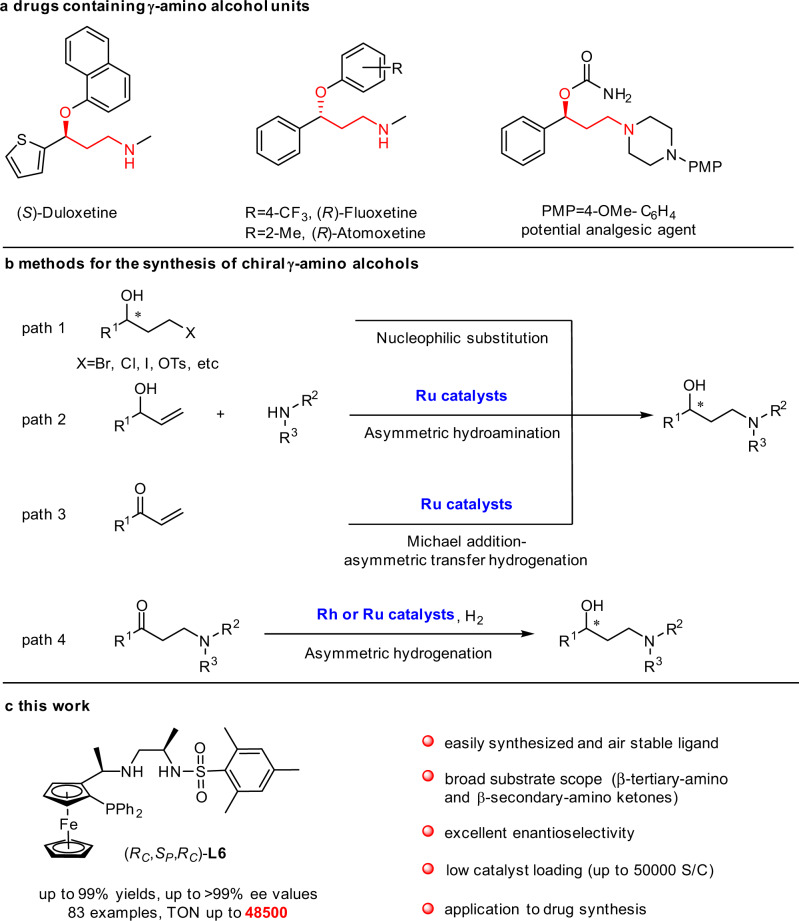
The designed ligand for asymmetric hydrogenation of β-amino ketones. **a** Drugs and potential analgesic agent containing γ-amino alcohol units. **b** Methods for the synthesis of chiral γ-amino alcohols. **c** The designed ligand for asymmetric hydrogenation of β-amino ketones.

Enantiomerically enriched γ-amino alcohols, a class of prevalent building blocks, play a vital role as indispensable intermediates in organic transformation and pharmaceutical production of the antidepressant drugs such as (*S*)-duloxetine, (*R*)-fluoxetine, and (*R*)-atomoxetine, as well as the potential chiral drugs (Fig. 1a)^1–6^. A multitude of processes have been developed for the synthesis of chiral γ-amino alcohols, for example, the nucleophilic substitution of the chiral alcohol bearing the leaving group in the γ-position with the amine (Fig. 1b, path 1)^7–10^, the Ru-catalyzed asymmetric hydroamination of the unsaturated alcohol and the amine (Fig. 1b, path 2)^11,12^, the Michael addition–asymmetric transfer hydrogenation of the unsaturated ketone and the amine (Fig. 1b, path 3)^13^, as well as the asymmetric hydrogenation of the β-amino ketone (Fig. 1b, path 4)^14–22^. The transition-metal-catalyzed asymmetric hydrogenation of prochiral β-amino ketones is doubtlessly regarded as one of the most promising and favorable methods due to the perfect atom economy and environmental benignity.

Enantioselective hydrogenation of β-amino ketones catalyzed by chiral Ru– and Rh–phosphine complexes to afford optically active amino alcohols has been extensively studied during the past decades, promoting the generation of chiral drugs to a certain extent. In 1991, Achiwa developed the (2*S*,4*S*)-MCCPM–Rh catalyst in the successful synthesis of (*R*)-fluoxetine by enantioselective reduction of 3-(methylbenzylamino)-1-phenyl-1-propanone hydrochloride with 90.8% ee and substrate to catalyst molar ratio (S/C) of 1000^14^. But the lower enantioselectivity of 79.8% ee was observed for 3-(methylamino)-1-phenyl-1-propanone hydrochloride. Subsequently, Noyori and co-workers reported efficient protocols for the asymmetric synthesis of γ-tertiary-amino alcohols using chiral [RuCl_2_(diphosphine)(1,2-diamine)] catalysts with excellent outcomes^15–17^. In 2005, Zhang and co-workers reported the enantioselective hydrogenation of β-secondary-amino ketones by Rh–duanphos catalyst in 90–93% yields with 93–99% ee and up to 4500 TON^18^. Moreover, Zhang applied RuPHOX–Ru complex and P-chiral Rh–bisphosphine complex in the asymmetric reduction of β-amino ketones providing the corresponding products with satisfactory results^19–22^.

Although the chiral Rh catalysts and Ru catalysts with varied phosphine ligands predominated in the asymmetric hydrogenation of β-amino ketones, the requirement of high catalyst loading and long reaction time, the poor stability, narrow substrate scope, as well as low reactivity and enantioselectivity of the reported catalysts could not be ignored. Therefore, the development of efficient and practical chiral catalysts is remarkably significant and dramatically desirable for the asymmetric access to γ-amino alcohols, albeit with the great challenge in finding novel and robust ligands. In addition to the Rh and Ru catalysts, the Ir catalysts were also widely employed in the asymmetric hydrogenation, and exhibited excellent reactivity and enantioselectivity for the reduction of various unsaturated compounds containing C═C^23–32^, C═O^33–41^, and C═N^42–48^, but the Ir-catalyzed enantioselective hydrogenation of challenging β-amino ketones was less studied. In 2011, Zhou presented excellent SpiroPAP-Ir catalyst for enantioselective hydrogenation of simple ketones, showing a dramatically high TON and promoting the development and application of Ir catalysts in the reduction of ketones^33^. In 2018, Zhang^39^ and Zhong^40^ reported the asymmetric reduction of simple ketones using Ir–PNN complexes containing C2 symmetric ethylenediamine and cyclohexanediamine scaffolds, respectively. The symmetric vicinal diamines were limited, while unsymmetrical vicinal diamines, such as those from amino acids were versatile, modular, and tunable. We envisioned that this type of ligand bearing diverse unsymmetrical diamines from amino acids might provide a great opportunity to systematically assess the catalytic performance of corresponding Ir catalysts for asymmetric hydrogenation through fine-tuning of electronic effect and steric hindrance, owing to the easy synthesis, multiple tuning sites, low cost, and high robustness. Herein, we reported a series of Ir catalysts with chiral ferrocene-based phosphine ligands bearing unsymmetrical vicinal diamine scaffolds and their successful application in asymmetric hydrogenation of β-amino ketones for the generation of various chiral γ-amino alcohols, including diverse γ-tertiary-amino and γ-secondary-amino alcohol intermediates of (*S*)-duloxetine, (*R*)-fluoxetine, and (*R*)-atomoxetine.

## Results

### Reaction optimization

The chiral tridentate ferrocene-based phosphine ligands (*R*_*C*_,*S*_*P*_,*R*_*C*_)-**L1**–**L7** bearing monosulfonyl unsymmetrical vicinal diamine scaffolds were easily accessed via efficient coupling of acetate derived from commercial (*R*)-Ugi’s amine and unsymmetrical vicinal diamines from common chiral amino alcohols (see 2.1 in Supplementary Information). With these stable ligands in hand, we investigated the catalytic performance in terms of reactivity and enantioselectivity with the catalysts prepared in situ by mixing the [Ir(COD)Cl]_2_ with ligands (*R*_*C*_,*S*_*P*_,*R*_*C*_)-**L1**–**L7** in *i*PrOH under 30 atm of H_2_ in the presence of LiO*t*Bu. *N*-Boc-3-(methylamino)-1-(2-thienyl)-1-propanone **1a** was tested as the model substrate, which was relatively challenging due to the additional coordination ability of the sulfur atom. For example, the aryl-substituted β-amino ketones could be hydrogenated smoothly by RuPHOX–Ru catalyst, while the reduction of thienyl-substituted substrates was failed^19^. As shown in Table 1, alkyl and aryl substituents in the unsymmetrical vicinal diamine scaffolds of ligands were proved to have an important impact on the catalytic behavior. The Me and *i*Pr as the R groups promoted the transformation to proceed smoothly in >99% conversions and with 87% and 45% ee, respectively (entries 1–2), while the *t*Bu led to the significant loss of the product (entry 3). The reaction performances deteriorated successively in the order of Me > *i*Pr > *t*Bu, suggesting that the steric hindrance of R group was crucial. When the phenyl or benzyl was on the α-position of sulfonamide group, 99% conversions were obtained with 76% and 93% ee, respectively (entries 4–5). Moreover, the significant effects on enantioselectivity were exerted by the steric hindrances of sulfonamide groups of ligands. The (*R*_*C*_,*S*_*P*_,*R*_*C*_)-**L6** with the replacement of *p*-toluenesulfonyl in (*R*_*C*_,*S*_*P*_,*R*_*C*_)-**L1** with 2,4,6-trimethylbenzenesulfonyl resulted in the obviously increased enantioselectivity of 97% ee, but Ir–(*R*_*C*_,*S*_*P*_,*R*_*C*_)-**L7** catalyst gave the similar catalytic outcomes with Ir–(*R*_*C*_,*S*_*P*_,*R*_*C*_)-**L5** catalyst (entries 6–7). X-ray crystallography determined the absolute configuration of **2a** to be *S* (see Supplementary Fig. 276 and Supplementary Data 1).

**Table 1:** Optimization of reaction conditions for asymmetric hydrogenation of 1a.


Entry^a^	Ligand	Base	Conv. [%]^b^	ee [%]^c^
1	(*R*_*C*_,*S*_*P*_,*R*_*C*_)-**L1**	LiO*t*Bu	>99	87
2	(*R*_*C*_,*S*_*P*_,*R*_*C*_)-**L2**	LiO*t*Bu	>99	45
3	(*R*_*C*_,*S*_*P*_,*R*_*C*_)-**L3**	LiO*t*Bu	NR	ND
4	(*R*_*C*_,*S*_*P*_,*R*_*C*_)-**L4**	LiO*t*Bu	>99	76
5	(*R*_*C*_,*S*_*P*_,*R*_*C*_)-**L5**	LiO*t*Bu	>99	93
6	(*R*_*C*_,*S*_*P*_,*R*_*C*_)-**L6**	LiO*t*Bu	>99	97
7	(*R*_*C*_,*S*_*P*_,*R*_*C*_)-**L7**	LiO*t*Bu	>99	93
8	(*R*_*C*_,*S*_*P*_,*R*_*C*_)-**L6**	NaO*t*Bu	>99	98
9	(*R*_*C*_,*S*_*P*_,*R*_*C*_)-**L6**	KO*t*Bu	>99	93
10	(*R*_*C*_,*S*_*P*_,*R*_*C*_)-**L6**	LiOH	>99	97
11	(*R*_*C*_,*S*_*P*_,*R*_*C*_)-**L6**	NaOH	>99	97
12	(*R*_*C*_,*S*_*P*_,*R*_*C*_)-**L6**	KOH	>99	94
13	(*R*_*C*_,*S*_*P*_,*R*_*C*_)-**L6**	K_2_CO_3_	NR	ND
14	(*R*_*C*_,*S*_*P*_,*R*_*C*_)-**L6**	Na_2_CO_3_	NR	ND
15^d^	(*R*_*C*_,*S*_*P*_,*R*_*C*_)-**L6**	NaO*t*Bu	NR	ND
16^e^	(*R*_*C*_,*S*_*P*_,*R*_*C*_)-**L6**	NaO*t*Bu	>99	99
17^f^	(*R*_*C*_,*S*_*P*_,*R*_*C*_)-**L6**	NaO*t*Bu	>99	98
18^g^	(*R*_*C*_,*S*_*P*_,*R*_*C*_)-**L6**	NaO*t*Bu	>99	99
19^h^	(*R*_*C*_,*S*_*P*_,*R*_*C*_)-**L6**	NaO*t*Bu	>99	99

^a^Reaction conditions: 0.4 mmol scale, 0.05 mol% [Ir(COD)Cl]_2_, 0.105 mol% Ligand, 5 mol% base, 2.0 mL solvent, room temperature (25–30 °C). ^b^Determined by ^1^H NMR analysis. ^c^Determined by HPLC analysis. ^d^2 mL MeOH as solvent. ^e^2 mL hexane as solvent. ^f^2 mL THF as solvent. ^g^2 mL CH_2_Cl_2_ as solvent. ^h^2 mL toluene as solvent.

Bases have shown extremely discrepant results in asymmetric hydrogenation. The mechanism research on the asymmetric reduction of acetophenone using [RuX_2_(diphosphine)(1,2-diamine)] catalysts showed the promptly accelerated effect for H-H bond cleavage and relatively lower barriers for hydride transfer in the presence of KO*t*Bu^49^. Thus, the base effect was evaluated in Ir–(*R*_*C*_,*S*_*P*_,*R*_*C*_)-**L6**-catalyzed asymmetric hydrogenation of **1a** in *i*PrOH. LiOH and NaOH presented quantitative conversions and equal ee values of 97%, as the same with LiO*t*Bu (entries 10–11), while NaO*t*Bu slightly increased the enantioselectivity to 98% ee (entry 8). Moreover, >99% conversions were observed in the presence of KO*t*Bu or KOH with 93% or 94% ee, respectively (entries 9 and 12). It seemed that the K^+^ caused the loss of enantioselectivity in contrast with the cases of Li^+^ and Na^+^, probably due to the electronegativity difference of alkali metal cations. Ulteriorly, the enantioselective reduction was adversely affected by the weaker bases including K_2_CO_3_ and Na_2_CO_3_, and no desired product was detected (entries 13–14). Thereby, NaO*t*Bu was identified to be the optimal base in this procedure. Next, we turned our concern into the influence of solvents. Unfortunately, the replacement of *i*PrOH with MeOH resulted in the lack of target product (entry 15). Subsequently, aprotic solvents were further explored. To our delight, hexane, CH_2_Cl_2_, or toluene as solvent all provided **2a** in >99% conversions and 99% ee values (entries 16 and 18–19). Moreover, THF also exhibited excellent performance in >99% conversion and 98% ee, suggesting the aprotic solvents benefited the Ir–(*R*_*C*_,*S*_*P*_,*R*_*C*_)-**L6**-catalyzed asymmetric hydrogenation of β-amino ketones (entry 17). Taking the consideration of the solubility, especially the toxicity and volatility of CH_2_Cl_2_, toluene was selected for further utilization, which was frequently used in industry.

### Substrate scope

**Fig. 2:**
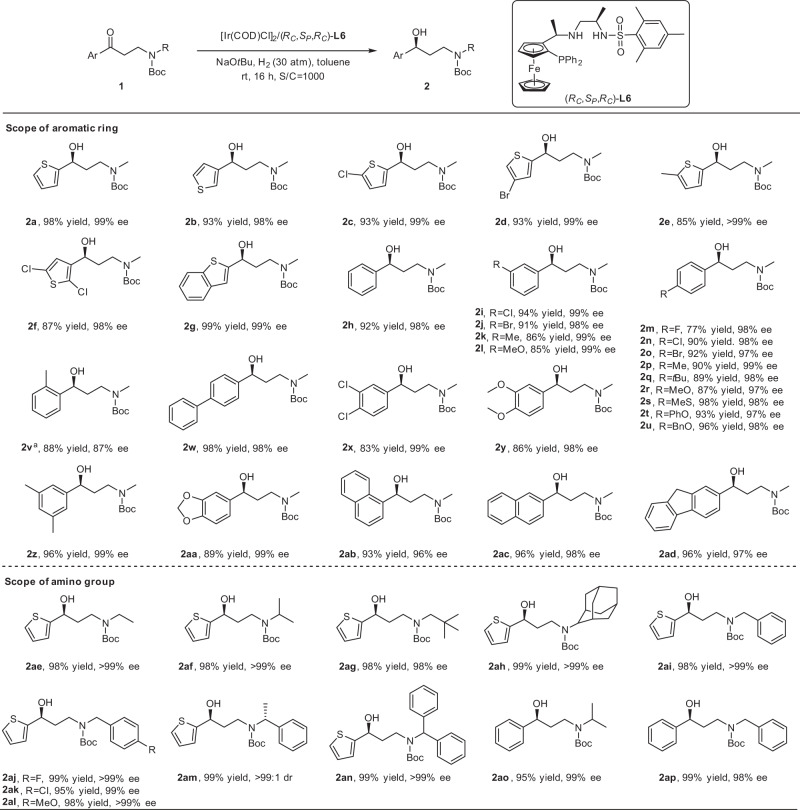
Asymmetric hydrogenation of various N-Boc-β-amino ketones with Ir–(RC,SP,RC)-L6 catalyst. Reaction conditions: 0.4 mmol scale, 0.05 mol% [Ir(COD)Cl]_2_, 0.105 mol% (*R*_*C*_,*S*_*P*_,*R*_*C*_)-**L6**, 5 mol% NaO*t*Bu, 2.0 mL toluene, room temperature (25–30 °C). Isolated yields. The ee determined by HPLC. ^a^at 60 °C.

With the optimized conditions of asymmetric hydrogenation in hand, the reduction of diverse *N*-Boc substituted substrates was performed, which could allow easy access to plentiful important γ-secondary-amino alcohols (Fig. 2). A variety of heteroaromatic and aromatic β-amino ketones were confirmed to be compatible with the system, affording the corresponding chiral γ-amino alcohols in excellent yields and tremendously high ee values. *N*-Boc-3-(methylamino)-1-(3-thienyl)-1-propanone **1b**, which was different in the substitution site on thienyl with model substrate, was examined, and 93% yield and 98% ee were obtained. The substrates bearing substituents in the *ortho-* or *meta-*position of sulfur atom on the thienyl ring provided the corresponding products with extremely high enantioselectivities and admirable yields (**2c**–**2e**). The thienyl-substituted substrate with multiple electron-withdrawing groups, like di-Cl groups, provided **2f** in 87% yield and 98% ee. Moreover, the fused ring benzothienyl-substituted ketone **1g** was hydrogenated smoothly and delivered the chiral alcohol **2g** with 99% yield and 99% ee. The outcomes of varied heteroaromatic substrates with substituted thienyl groups suggested that the sulfur atom had no influence on the catalytic capability of the Ir–(*R*_*C*_,*S*_*P*_,*R*_*C*_)-**L6** catalyst, though it usually poisoned catalysts in transition-metal-catalyzed reactions^50–53^. In addition, the aromatic β-amino ketones without substituent (**1h**) or bearing varied electron-withdrawing/electron-donating substituent groups formed admirable enantioselectivities of 97–99% ee and good to excellent yields of 77–98%, regardless in the *meta*- or *para*-position on the phenyl ring (**2h**–**2u**). The satisfactory results showed that electrical properties of substituents exerted no implication for the enantioselectivities. While the *ortho*-substituted substrate (**1v**) gave the product with a slightly lower ee, suggesting that substituent on the benzene ring adjacent to the carbonyl had a little influence on the enantioselectivity. Meanwhile, the π-π-conjugated biphenyl and disubstituted aryl β-amino ketones triumphantly participated in the reaction with excellent outcomes (**2w**–**2aa**). Replacement of the phenyl skeleton by fused rings, such as 1-naphthyl, 2-naphthyl, or 2-fluorenyl led to superb results with 96–98% ee and 93–96% yields (**2ab**–**2ad**).

More broadly, the scope of amino groups was then evaluated (Fig. 2). Alkyl substituents with different steric hindrances on the nitrogen atom of *N*-Boc-3-(alkylamino)-1-(2-thienyl)-1-propanone, even 2-adamantyl, were all compatible. The corresponding chiral products were obtained successfully in excellent yields of 98–99% and enantioselectivities of 98– > 99% ee (**2ae**–**2ah**). Substrates with benzyl or benzyl groups bearing substituents of different electronic properties on the nitrogen atom were also hydrogenated smoothly giving satisfactory outcomes (**2ai**–**2al**). The configuration of optically pure α-methyl benzylamino in **1am** could remain in the transformation, and **2am** was obtained with > 99:1 dr. The hydrogenation of the α-phenyl substituted benzylamino substrate provided **2an** in 99% yield and > 99% ee. In addition, *N*-Boc-3-(isopropylamino)-1-phenyl-1-propanone was hydrogenated to deliver **2ao** in 95% yield and 99% ee, and the case of *N*-Boc-3-(benzylamino)-1-phenyl-1-propanone for the synthesis of **2ap** was 99% yield and 98% ee.

**Fig. 3:**
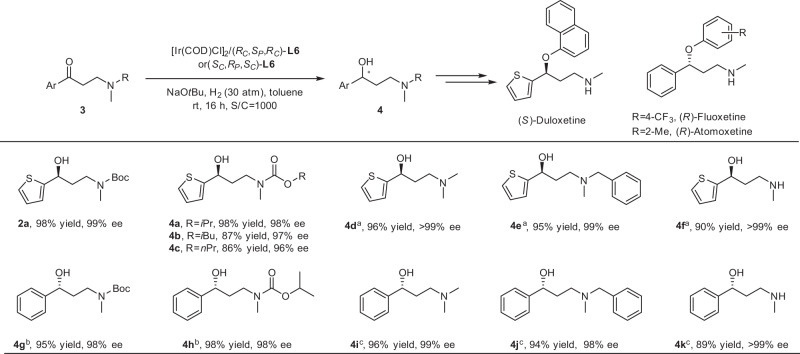
Asymmetric synthesis of chiral intermediates of (S)-duloxetine, (R)-fluoxetine, and (R)-atomoxetine. Reaction conditions: 0.4 mmol scale, 0.05 mol% [Ir(COD)Cl]_2_, 0.105 mol% (*R*_*C*_,*S*_*P*_,*R*_*C*_)-**L6**, 5 mol% NaO*t*Bu, 2.0 mL toluene, room temperature (25–30 °C). Isolated yields. The ee determined by HPLC. ^a^0.4 mmol **3**·HCl, 0.44 mmol NaO*t*Bu. ^b^Ir–(*S*_*C*_,*R*_*P*_,*S*_*C*_)-**L6** as catalyst. ^c^0.4 mmol **3**·HCl, 0.44 mmol NaO*t*Bu, Ir–(*S*_*C*_,*R*_*P*_,*S*_*C*_)-**L6** as catalyst.

This asymmetric method provided an efficient approach for the delivery of a diverse array of chiral γ-secondary-amino alcohols often encountered in bioactive molecules. On the basis of the extensive substrate compatibility, the β-amino ketones with other *N*-substituted groups were tested in Fig. 3. To our delight, all kinds of common intermediates of (*S*)-duloxetine, (*R*)-fluoxetine, and (*R*)-atomoxetine containing *N*-alkoxycarbonyl β-amino ketones, β-tertiary-amino ketones, and β-secondary-amino ketones underwent asymmetric hydrogenation smoothly. Diverse alkoxycarbonylamino groups, such as isopropoxycarbonylamino, isobutoxycarbonylamino, and *n*-propoxycarbonylamino substituted β-amino ketones, which were easily synthesized on a large scale, were hydrogenated successfully in Ir–(*R*_*C*_,*S*_*P*_,*R*_*C*_)-**L6**-catalyzed asymmetric hydrogenation with 86–98% yields and 96–98% ee (**4a**–**4c**). Moreover, 3-(dimethylamino)-1-(2-thienyl)-1-propanone hydrochloride (**3d**·HCl) and 3-(methylbenzylamino)-1-(2-thienyl)-1-propanone hydrochloride (**3e**·HCl) also performed well in asymmetric reduction and afforded the corresponding chiral alcohol products with excellent results (>99% and 99% ee values, 96% and 95% yields, respectively). Most notably, when the β-secondary-amino ketone hydrochloride (**3f**·HCl) in a more stable form was used, the high enantioselectivity of >99% ee and 90% yield were accessed. The hydrochlorides could ensure substrates stable in an alkaline environment and reduce the side reactions. In this way, a series of chiral γ-amino alcohols covering γ-secondary-amino alcohol and γ-tertiary-amino alcohol intermediates of (*S*)-duloxetine were obtained by Ir–(*R*_*C*_,*S*_*P*_,*R*_*C*_)-**L6** catalyst in high yields and enantioselectivities. It is worth mentioning that the catalysts that can exert great performance for the asymmetric hydrogenation of both heteroaromatic β-tertiary-amino ketones and β-secondary-amino ketones have not been reported. Further, the ligand (*S*_*C*_,*R*_*P*_,*S*_*C*_)-**L6**, the enantiomer of (*R*_*C*_,*S*_*P*_,*R*_*C*_)-**L6**, was synthesized and used in the asymmetric delivery of familiar γ-amino alcohol intermediates of (*R*)-fluoxetine and (*R*)-atomoxetine, which were contrary to the intermediates of (*S*)-duloxetine in configuration. The phenyl-substituted β-methyl alkoxycarbonylamino ketones and phenyl-substituted β-dimethylamino, β-methylbenzylamino, and β-monomethylamino ketone hydrochlorides underwent asymmetric reduction smoothly in 89–98% yields and 98– > 99% ee values (**4g**–**4k**). These dramatic results showed that this Ir-catalyzed system could provide efficiently both (*S*)- and (*R*)-configurations of various γ-amino alcohols.

**Fig. 4:**
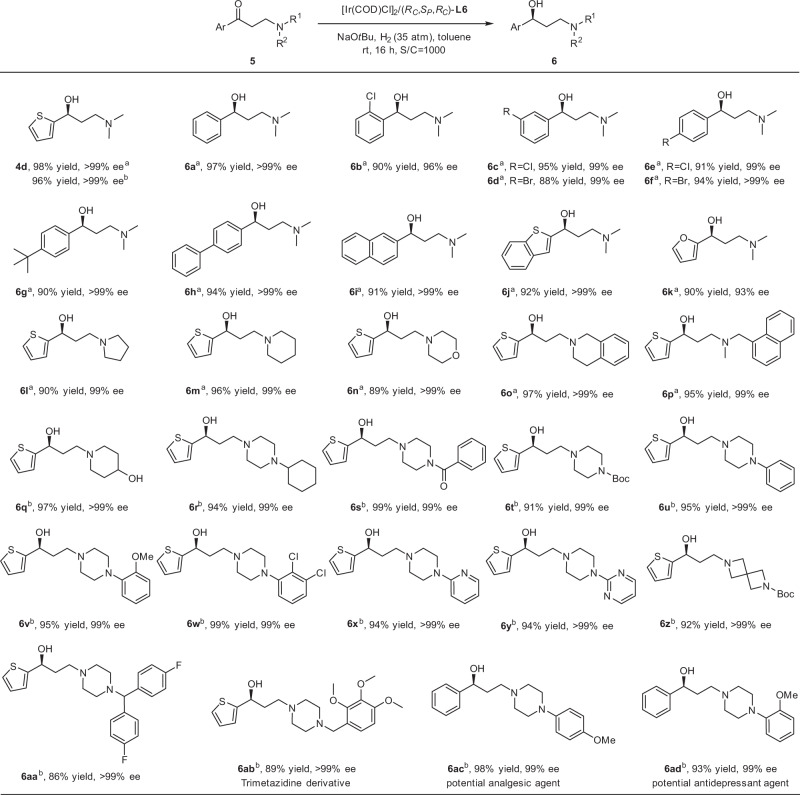
Asymmetric hydrogenation of various β-tertiary-amino ketones with Ir-(RC,SP,RC)-L6 catalyst. ^a^Condition A: 0.4 mmol **5**·HCl, 0.05 mol% [Ir(COD)Cl]_2_, 0.105 mol% (*R*_*C*_,*S*_*P*_,*R*_*C*_)-**L6**, 0.44 mmol NaO*t*Bu, 3.0 mL toluene, room temperature (25–30 °C). ^b^Condition B: 0.4 mmol **5**, 0.05 mol% [Ir(COD)Cl]_2_, 0.105 mol% (*R*_*C*_,*S*_*P*_,*R*_*C*_)-**L6**, 5 mol% NaO*t*Bu, 3.0 mL toluene, room temperature (25–30 °C). Isolated yields. The ee determined by HPLC.

The development of efficient method for asymmetric catalysis of β-dialkyl amino ketones was also highly desired, because different γ-dialkyl amino alcohols were involved in many important potential drugs^54–58^. Besides the Rh–MCCPM catalyst and Ru complexes^14–17,19^, Genov^59^ and Huang^60^ also respectively reported Ru^II^–((*R*,*R*)-bicp) complex and [RuCl_2_(BIDN)(DPPF)] complex for the reduction of 3-(dimethylamino)-1-(2-thienyl)-1-propanone to obtain the corresponding product with 96% and 94% ee, respectively. This type of substrate was very different from simple ketones in terms of stability and was inclined to undergo facile elimination to produce a large number of by-products in alkaline environment, which made the asymmetric transformation extremely challenging. To our delight, the Ir-catalyzed system was dramatically amenable to β-dialkyl amino ketones with excellent reactivity and enantioselectivity. As shown in Fig. 4, free amino ketone or its hydrochloride underwent smooth hydrogenation with excellent results (**4d**). Diverse aromatic β-dimethylamino ketone hydrochlorides with halogen atoms on the *ortho*-, *meta*-, or *para*-position as well as *t*Bu or phenyl on the *para*-position of phenyl ring were tolerated and the corresponding products were provided in 88–97% yields with extremely high enantioselectivities of 96– > 99% ee values (**6a**–**6h**). The asymmetric reduction of fused ring aryl and heteroaryl substrates also proceeded smoothly (**6i**–**6j**). The furanyl-substituted ketone gave **6k** in 90% yield and 93% ee. A vast of substrates with various functionalized amino groups worked well in this transformation. Cyclic amino groups such as tetrahydropyrrolidinyl, piperidinyl, morpholinyl, and tetrahydroisoquinolinyl substituted substrates provided chiral alcohols in 89–97% yields with 99– > 99% ee values (**6l**–**6o**). The acyclic amino-substituted product **6p** was formed in 95% yield and 98% ee. Gratifyingly, **6q** with 4-hydroxypiperidinyl substituent was also obtained in 97% yield and >99% ee, indicating the hydroxyl had no effect on the catalytic behaviors. In addition, the piperazinyl-substituted substrates were also investigated to further certify the tolerance of functional groups. Varied substituents including cycloalkyl, benzoyl, Boc, aryl, heteroaryl, or benzyl on the nitrogen atom of piperazinyl were compatible, and the corresponding alcohols were afforded in 86–99% yields with 99– > 99% ee values (**6r**–**6y**, **6aa**–**6ad**). Thereinto, **6ac**, and **6ad** could be converted to potential analgesic agent and antidepressant agent, respectively^5,54^. Furthermore, the substrate bearing spirocyclic amino group offered the chiral product **6z** in 92% yield and >99% ee.

### Gram-scale reactions

**Fig. 5:**
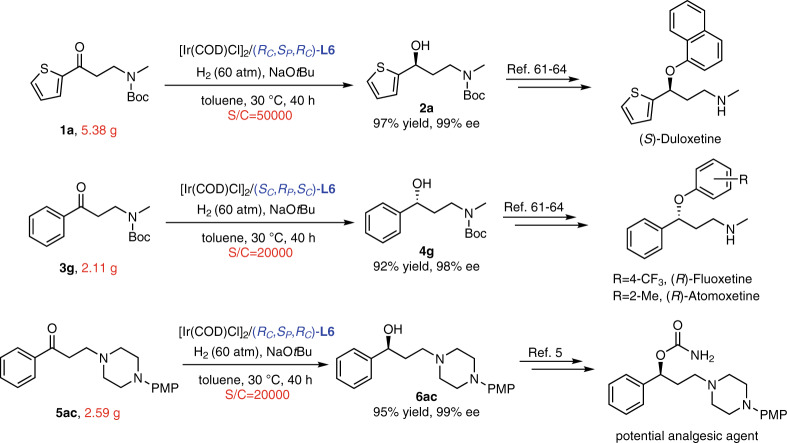
Gram-scale reactions. The asymmetric hydrogenation of **1a**, **3g**, and **5ac** with high S/C.

In order to demonstrate the potential utility of the protocol for the generation of important chiral drugs, gram-scale reactions were conducted under high S/C (Fig. 5). **1a** was hydrogenated smoothly by Ir–(*R*_*C*_,*S*_*P*_,*R*_*C*_)-**L6** catalyst with S/C of 50000, and the excellent performances remained (97% yield and 99% ee). Moreover, the Ir–(*S*_*C*_,*R*_*P*_,*S*_*C*_)-**L6** catalyst was used in the asymmetric transformation of **3g** accomplishing the 20000 S/C with 92% yield and 98% ee, and the case of Ir–(*R*_*C*_,*S*_*P*_,*R*_*C*_)-**L6** catalyst for **5ac** was 95% yield and 99% ee. The corresponding products could be further converted to the antidepressant drugs^61–64^ or potential analgesic agents^5^. It indicated that this Ir-catalyzed asymmetric transformation had great potential in industrial applications.

## Discussion

In summary, we developed an efficient catalytic system using iridium catalysts with chiral tridentate ferrocene-based phosphine ligands bearing unsymmetrical vicinal diamine scaffolds for the asymmetric hydrogenation of diverse β-amino ketones. This method allowed the access to all kinds of chiral γ-amino alcohols including γ-tertiary-amino alcohols and γ-secondary-amino alcohols with up to 99% yields, up to >99% ee values, and up to 48500 TON, providing an efficient approach for the synthesis of bioactive compounds. The intermediates of (*S*)-duloxetine, (*R*)-fluoxetine, and (*R*)-atomoxetine were obtained at gram scale with extremely low catalyst loading, indicating the method was of great significance in the industrial synthesis of chiral pharmaceutical drugs.

## Methods

### General procedure for the asymmetric hydrogenation of **1** (S/C = 1000)

Under nitrogen atmosphere, [Ir(COD)Cl]_2_ (1.4 mg, 0.002 mmol), (*R*_*C*_,*S*_*P*_,*R*_*C*_)-**L6** (2.8 mg, 0.0042 mmol), and anhydrous *i*PrOH (1 mL) were added to an oven-dried 10 mL schlenk tube. The mixture was stirred at room temperature for 2.5 h to give an orange solution. A part of catalyst solution (100 μL, 0.0004 mmol) was transferred into a 10 mL vial containing *N*-Boc-β-amino ketone **1** (0.4 mmol), NaO*t*Bu (1.9 mg, 0.02 mmol), and anhydrous toluene (2.0 mL). The vials were transferred to an autoclave, and the autoclave was purged with nitrogen and hydrogen three times in sequence, then charged with 30 atm of H_2_ and stirred for 16 h at room temperature. After hydrogen pressure was slowly released, the solvent was evaporated and the residue was purified by silica gel column chromatography to give the corresponding hydrogenation product **2**. Then the enantiomeric excesses were determined by HPLC analysis.

### General procedure for the asymmetric hydrogenation of **3** (S/C = 1000)

Under nitrogen atmosphere, [Ir(COD)Cl]_2_ (1.4 mg, 0.002 mmol), (*R*_*C*_,*S*_*P*_,*R*_*C*_)-**L6** or (*S*_*C*_,*R*_*P*_,*S*_*C*_)-**L6** (2.8 mg, 0.0042 mmol), and anhydrous *i*PrOH (1 mL) were added to an oven-dried 10 mL schlenk tube. The mixture was stirred at room temperature for 2.5 h to give an orange solution. A part of catalyst solution (100 μL, 0.0004 mmol) was transferred into a 10 mL vial containing ketone **3** or **3**·HCl (0.4 mmol), NaO*t*Bu (1.9 mg for **3a**–**3c** and **3g**–**3h**, 42.2 mg for **3d**–**3f**·HCl and **3i**–**3k**·HCl), and anhydrous toluene (2.0 mL). The vials were transferred to an autoclave, and the autoclave was purged with nitrogen and hydrogen three times in sequence, then charged with 30 atm of H_2_ and stirred for 16 h at room temperature. After hydrogen pressure was slowly released, the solvent was evaporated and the residue was purified by silica gel column chromatography to give the corresponding hydrogenation product **4**. Then the enantiomeric excesses were determined by HPLC analysis.

### General procedure for the asymmetric hydrogenation of **5** (S/C = 1000)

Under nitrogen atmosphere, [Ir(COD)Cl]_2_ (1.4 mg, 0.002 mmol), (*R*_*C*_,*S*_*P*_,*R*_*C*_)-**L6** (2.8 mg, 0.0042 mmol), and anhydrous *i*PrOH (1 mL) were added to an oven-dried 10 mL schlenk tube. The mixture was stirred at room temperature for 2.5 h to give an orange solution. A part of catalyst solution (100 μL, 0.0004 mmol) was transferred into a 10 mL vial containing β-tertiary-amino ketone **5** or **5**·HCl (0.4 mmol), NaO*t*Bu (42.2 mg for **5a**–**5p**·HCl, 1.9 mg for **5q**–**5ad**), and anhydrous toluene (3.0 mL). The vials were transferred to an autoclave, and the autoclave was purged with nitrogen and hydrogen three times in sequence, then charged with 35 atm of H_2_ and stirred for 16 h at room temperature. After hydrogen pressure was slowly released, the solvent was evaporated and the residue was purified by silica gel column chromatography to give the corresponding hydrogenation product **6**. Then the enantiomeric excesses were determined by HPLC analysis.

## Supplementary information

Description of Additional Supplementary Files

Supplementary Information

Supplementary Data 1

## Data Availability

The authors declare that the data supporting the findings of this study are available within the article and Supplementary Information. For experimental details and compound characterization data see Supplementary Notes 1, 2. For ^1^H NMR^13^,C NMR, and ^31^P NMR spectra see Supplementary Figs. 1–192 and HPLC spectra see Supplementary Figs. 193–275. The X-ray crystallographic data for **2a** could be obtained free of charge from The Cambridge Crystallographic Data Centre with the accession code CCDC 2116544 via www.ccdc.cam.ac.uk/data_request/cif.
