# Supplementary material for: Iridium-catalyzed enantioselective synthesis of chiral γ-amino alcohols and intermediates of (S)-duloxetine, (R)-fluoxetine, and (R)-atomoxetine
Source: Commun Chem. 2022 May 19;5:63. doi: 10.1038/s42004-022-00678-4 (PMC9814375; doi:10.1038/s42004-022-00678-4)
Supplement: Supplementary file 1 — Description of Additional Supplementary Files [file 42004_2022_678_MOESM1_ESM.pdf]

Description of Additional Supplementary Files

File Name: Supplementary Data 1

Description: Small molecule crystallographic data for 2a
