# Supplementary material for: Iridium-catalyzed enantioselective synthesis of chiral γ-amino alcohols and intermediates of (S)-duloxetine, (R)-fluoxetine, and (R)-atomoxetine
Source: Commun Chem. 2022 May 19;5:63. doi: 10.1038/s42004-022-00678-4 (PMC9814375; doi:10.1038/s42004-022-00678-4)
Supplement: Supplementary file 2 — Supplementary Information [file 42004_2022_678_MOESM2_ESM.pdf]

Supplementary Information

Iridium-Catalyzed Enantioselective Synthesis of Chiral γ-Amino Alcohols and Intermediates of (*S*)-Duloxetine, (*R*)-Fluoxetine, and (*R*)-Atomoxetine

Chengyu Liu, Lei Zhang, Liming Cao, Yan Xiong, Yueyue Ma, Ruihua Cheng* & Jinxing Ye*

Table of Contents

1. Supplementary Note 1 .... S2

2. Supplementary Note 2 .... S2

2.1. General procedure for preparation of the ligands S2

2.2. Preparation of substrates S7

2.3. Asymmetric hydrogenation of β-amino ketones S9

2.4. NMR spectra S41

2.5. HPLC spectra S138

2.6. Crystal structure of 2a S221

3. Supplementary References S222

1. Supplementary Note 1

^{1}H NMR spectra, ^{13}C NMR spectra, and ^{31}P NMR spectra were recorded on Bruker ADVANCE III 400 spectrometer or Bruker Ascend 600 spectrometer. Chemical shifts (δ) for protons are reported in parts per million (ppm) downfield from tetramethylsilane and referenced to residual solvent peak. Chemical shifts (δ) for carbons are reported in parts per million (ppm) downfield from tetramethylsilane and referenced to the carbon resonances of the solvent. Chemical shifts for phosphorus are reported in parts per million (ppm) downfield from the external 85% H_3PO_4 signal at 0.0 ppm as a standard. Data are reported as follows: chemical shift, multiplicity (br = broad, s = singlet, d = doublet, dd = doublet of doublets, t = triplet, dt = doublet of triplets, q = quartet, quint = quintet, m = multiplet, qd = quartet of doublets, ddd = doublet of doublet of doublets), coupling constants (*J*) in Hertz (Hz), integration; "app" is used to denote the apparent splitting of a signal.

High resolution mass spectrometry (ESI) was carried out using a Waters Quatro Macro triple quadrupole mass spectrometer. High resolution mass spectrometry (EI) was carried out using MicroMass GCT CA 055 instrument and recorded on a MicroMass LCTTM spectrometer.

Optical rotations were measured on an Autopol III automatic polarimeter (Rudolph Research analytical). $[\alpha]_D^T$ values are reported in 10^{-1} deg cm^2 g^{-1}; concentrations (*c*) are quoted in g/100 mL; D refers to the D-line of sodium (589 nm); temperatures (T) are given in degrees Celsius (°C).

Enantiomeric excesses were determined by HPLC analysis on an Agilent HPLC 1200 Series instrument, using the chiral stationary phase column (25 cm x 4.6 mm internal diameter, Chiralpak AD-H, OD-H as noted) specified in the individual experiment.

Anhydrous *i*PrOH, MeOH, EtOH, toluene, THF, CH_2Cl_2, and hexane were dried by 4 Å molecular sieves. $[Ir(COD)Cl]_2$ was purchased from commercial supplier.

2. Supplementary Note 2

2.1. General procedures for preparation of the ligands

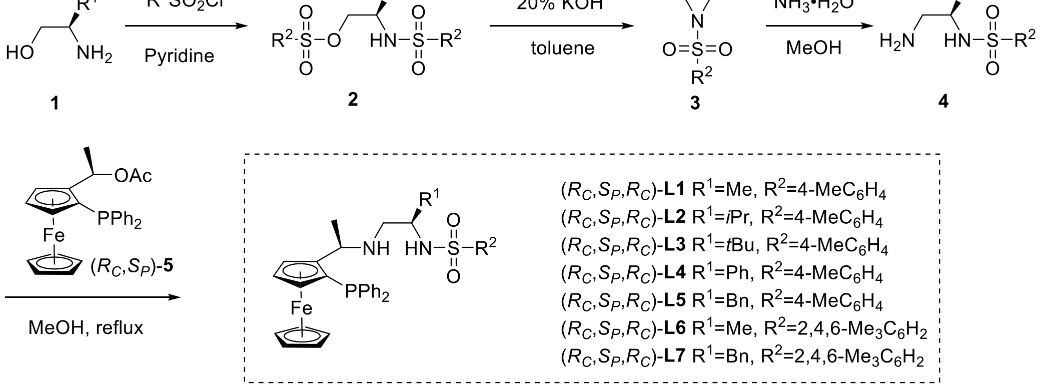

General procedure [1-6]

The synthesis of 2: To a solution of sulfonyl chloride R^2SO_2Cl (110 mmol) in pyridine (15 mL) was added, the solution of corresponding amino alcohol (50 mmol) in pyridine (5 mL). The mixture was stirred at room temperature for 24 h and quenched with 2 M HCl aqueous solution (50 mL), water (100 mL) and ethyl acetate (100 mL). The organic layer was separated, and the aqueous layer was extracted with ethyl acetate (50 mL x 3). The combined organic layer was washed with brine (40 mL), dried over anhydrous Na_2SO_4, then filtered and evaporated. The residue was recrystallization from ethyl acetate and hexane to afford the yellow solid **2** (68–87% yields).

The synthesis of 3: To a suspension of **2** (20 mmol) in toluene (80 mL) was added 20% KOH aqueous solution (32 mL). The mixture was stirred at room temperature for 2 h and then added water (80 mL). The organic layer was separated, and the aqueous layer was extracted with ethyl acetate (40 mL x 3). The combined organic layer was washed with brine (40 mL), dried over anhydrous Na_2SO_4, then filtered and evaporated to afford the light yellow solid **3** (98–99% yields).

The synthesis of 4: Aqueous ammonia (25–28%, 30 mL) was added to a stirred solution of aziridine **3** (10 mmol) in methanol (20 mL) at room temperature. Then the reaction was heated to 60 °C and stirred for 2 h. After the aziridine was completely consumed, the volatiles were removed in vacuo, and the product was extracted from the resulting aqueous mixture with CH_2Cl_2 (50 mL x 3). The combined organic layer was dried over anhydrous Na_2SO_4, filtered, and concentrated in vacuo. The crude residue was purified by silica gel column chromatography (CH_2Cl_2/MeOH = 25/1–10/1 as eluent) to afford white solid **4** (40–89% yields).

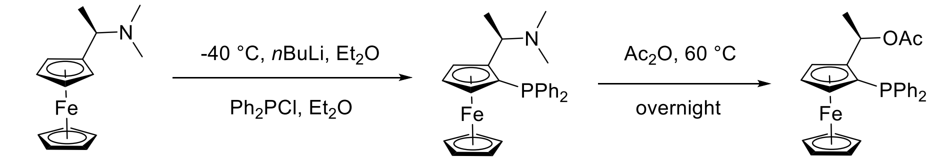

(*R*)-Ugi's amine (*R*C,*S*P)-**6** (*R*C,*S*P)-**5**

The synthesis of (*R*C,*S*P)-6: To a solution of (*R*)-Ugi's amine (7.7 g, 30.0 mmol) in anhydrous ether (60 mL) in a three-necked flask was added dropwise *n*BuLi in hexane (15 mL, 2.5 M) at -40 °C for 20–30 min. After addition was completed, the mixture was warmed to room temperature and stirred for 2 h. Then diphenylphosphorus chloride (13.2 g, 60 mmol) in ether (20 mL) was added dropwise to the mixture. Next, the mixture was heated to reflux for 4 h and quenched with saturated sodium bicarbonate aqueous solution. The organic phase was separated, washed with saturated brine (40 mL), dried over anhydrous Na_2SO_4, filtered, and concentrated in vacuo to remove the solvent. The residue was purified by silica gel column chromatography to obtain yellow solid (*R*C,*S*P)-**6** (7.4 g, 56% yield).

The synthesis of (*R_C*,*S_P*)-5: A solution of (R_C,S_P)-**6** (3.0 g, 6.8 mmol) in acetic anhydride (6 mL) was heated to 60 °C overnight under nitrogen atmosphere. After the completion of the reaction, acetic anhydride was removed by vacuum distillation to obtain the crude product (R_C,S_P)-**5** without further purification. Product (R_C,S_P)-**5** was stored at low temperature and used directly in the next step.

The synthesis of ligands (R_C,S_P,R_C)-L1-L7 and (S_C,R_P,S_C)-L6 : A mixture of (R_C,S_P)-**5** (114.1 mg, 0.5 mmol) and unsymmetric vicinal diamine **4** (1 mmol) in dry methanol (2 mL) was refluxed overnight under nitrogen. The reaction mixture was cooled to room temperature, and then the solvent was removed under vacuum. The crude product was purified by silica gel column chromatography to yield (R_C,S_P,R_C)-**L1**–**L7**. (S_C,R_P,S_C)-**L6** was synthesized though the same procedure for (R_C,S_P,R_C)-**L6** using (S_C,R_P)-**5** as starting material.

Characterization data of ligands

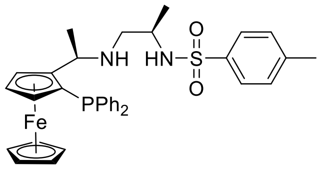

(R_C,S_P,R_C)-**L1**

Orange solid, 168 mg, 54% yield. $[\alpha]_D^{25}$ = -82.6 (*c* 0.5, CH_2Cl_2). ^{1}H NMR (600 MHz, $CDCl_3$) δ 7.71 (d, *J* = 8.2 Hz, 2H), 7.58 – 7.49 (m, 2H), 7.41 – 7.35 (m, 3H), 7.30 – 7.24 (m, 2H), 7.23 – 7.13 (m, 5H), 5.19 (s, 1H), 4.39 (dt, *J* = 2.9, 1.6 Hz, 1H), 4.29 (t, *J* = 2.5 Hz, 1H), 3.98 (s, 5H), 3.96 – 3.90 (m, 1H), 3.81 (dt, *J* = 2.4, 1.1 Hz, 1H), 2.73 (q, *J* = 5.9 Hz, 1H), 2.43 (s, 3H), 2.11 (dd, *J* = 5.3, 1.6 Hz, 2H), 1.31 (d, *J* = 6.5 Hz, 3H), 0.55 (d, *J* = 6.6 Hz, 3H). ^{13}C NMR (151 MHz, $CDCl_3$) δ 142.94, 140.04 (d, *J* = 9.5 Hz), 138.25, 137.11 (d, *J* = 8.9 Hz), 135.11, 134.97, 132.85, 132.72, 129.55, 129.27, 128.43, 128.39, 128.29, 128.24, 127.37, 97.08 (d, *J* = 24.0 Hz), 75.28 (d, *J* = 7.0 Hz), 71.60 (d, *J* = 4.9 Hz), 69.85, 69.28 (d, *J* = 4.7 Hz), 69.10, 51.34 (d, *J* = 8.7 Hz), 50.77, 49.34, 21.67, 19.36, 18.72. ^{31}P NMR (243 MHz, $CDCl_3$) δ -24.76 (s). HRMS (ESI): exact mass calculated for $C_{34}H_{38}FeN_2O_2PS$ $[M+H]^+$ 625.1736, found 625.1749.

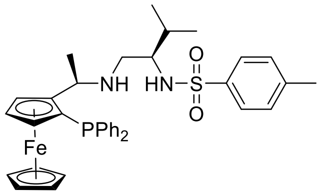

(R_C,S_P,R_C)-**L2**

Orange solid, 205 mg, 63% yield. $[\alpha]_D^{25}$ = -84.2 (*c* 0.5, CH_2Cl_2). ^{1}H NMR (600 MHz, $CDCl_3$) δ 7.80 (d, *J* = 8.0 Hz, 2H), 7.62 – 7.47 (m, 2H), 7.40 – 7.36 (m, 3H), 7.29 (d, *J* = 8.0 Hz, 2H), 7.22 – 7.11 (m, 5H), 5.09 (s, 1H), 4.38 (s, 1H), 4.28 (s, 1H), 3.95 (s, 5H), 3.84 – 3.79 (m, 2H), 2.51 (s, 1H), 2.44 (s, 3H), 2.40 (dd, *J* = 12.5, 4.6 Hz, 1H), 1.95 (dd, *J* = 12.5, 4.6 Hz, 1H), 1.25

(d, J = 6.5 Hz, 3H), 0.96 – 0.86 (m, 1H), 0.50 (dd, J = 8.8, 6.8 Hz, 6H). ^{13}C NMR (151 MHz, $CDCl_3$) δ 142.80, 140.32 (d, J = 9.0 Hz), 138.75, 137.62 (d, J = 8.5 Hz), 135.37, 135.23, 132.60, 132.48, 129.45, 129.27, 128.31, 128.26, 128.23, 128.18, 128.04, 127.50 (d, J = 2.5 Hz), 97.18 (d, J = 24.7 Hz), 75.21 (d, J = 7.5 Hz), 71.77 (d, J = 5.5 Hz), 69.81, 69.23 (d, J = 4.9 Hz), 69.10, 59.32, 52.08 (d, J = 7.9 Hz), 45.96, 29.06, 21.69, 19.05, 18.98, 18.64. ^{31}P NMR (243 MHz, $CDCl_3$) δ -24.34 (s). HRMS (ESI): exact mass calculated for $C_{36}H_{42}FeN_2O_2PS$ $[M+H]^+$ 653.2049, found 653.2032.

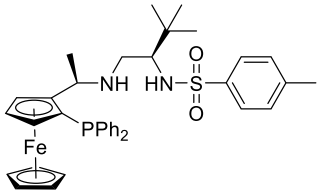

(R_C,S_P,R_C)-**L3**

Orange solid, 156 mg, 47% yield. $[\alpha]_D^{25}$ = -92.0 (c 0.5, CH_2Cl_2). ^{1}H NMR (600 MHz, $CDCl_3$) δ 7.85 (d, J = 8.3 Hz, 2H), 7.58 – 7.52 (m, 2H), 7.40 – 7.35 (m, 3H), 7.29 (d, J = 7.8 Hz, 2H), 7.24 – 7.22 (m, 2H), 7.21 – 7.18 (m, 2H), 5.19 (s, 1H), 4.32 – 4.30 (m, 1H), 4.28 (t, J = 2.5 Hz, 1H), 3.91 (s, 5H), 3.90 – 3.88 (m, 1H), 3.80 – 3.75 (m, 1H), 2.65 (t, J = 4.6 Hz, 1H), 2.44 (s, 3H), 2.37 (dd, J = 13.0, 4.3 Hz, 1H), 1.99 (dd, J = 13.0, 5.0 Hz, 1H), 1.23 (d, J = 6.6 Hz, 3H), 0.49 (s, 6H). ^{13}C NMR (151 MHz, $CDCl_3$) δ 142.78, 140.19 (d, J = 9.0 Hz), 139.07, 137.95 (d, J = 8.8 Hz), 135.33, 135.18, 132.70, 132.58, 129.36, 129.24, 128.43, 128.39, 128.22, 128.17, 128.13, 127.69 (d, J = 2.7 Hz), 97.32 (d, J = 25.6 Hz), 75.12 (d, J = 8.2 Hz), 71.78 (d, J = 5.4 Hz), 69.77, 69.39 (d, J = 5.4 Hz), 69.07, 61.76, 51.92 (d, J = 8.2 Hz), 45.25, 34.61, 27.19, 21.73, 19.20. ^{31}P NMR (243 MHz, $CDCl_3$) δ -25.83 (s). HRMS (ESI): exact mass calculated for $C_{37}H_{44}FeN_2O_2PS$ $[M+H]^+$ 667.2205, found 667.2189.

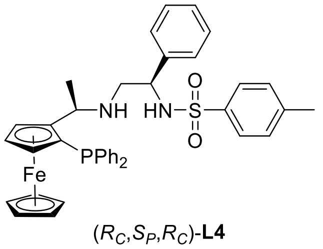

(R_C,S_P,R_C)-**L4**

Orange solid, 154 mg, 45% yield. $[\alpha]_D^{25}$ = -122.8 (c 0.5, CH_2Cl_2).^{1}H NMR (600 MHz, $CDCl_3$) δ 7.60 – 7.51 (m, 4H), 7.40 – 7.36 (m, 3H), 7.32 – 7.27 (m, 1H), 7.24 – 7.17 (m, 4H), 7.13 (d, J = 8.0 Hz, 2H), 7.08 – 7.04 (m, 1H), 7.01 (dd, J = 8.2, 6.6 Hz, 2H), 6.74 – 6.64 (m, 2H), 5.90 (s, 1H), 4.39 (dd, J = 2.6, 1.5 Hz, 1H), 4.28 (t, J = 2.5 Hz, 1H), 3.96 (s, 5H), 3.94 – 3.90 (m, 1H), 3.88 – 3.81 (m, 2H), 2.55 (dd, J = 12.1, 6.5 Hz, 1H), 2.41 – 2.32 (m, 4H), 1.32 (d, J = 6.4 Hz, 3H). ^{13}C NMR (151 MHz, $CDCl_3$) δ 142.70, 140.38 (d, J = 9.7 Hz), 139.42, 137.94, 137.60 (d, J = 9.0 Hz), 135.22, 135.08, 132.88, 132.76, 129.27, 129.18, 128.48, 128.44, 128.29 (d, J =

3.1 Hz), 128.22, 128.11, 127.46, 127.01, 126.76, 97.00 (d, J = 24.2 Hz), 75.64 (d, J = 7.5 Hz), 71.76 (d, J = 5.5 Hz), 69.84, 69.22 (d, J = 4.9 Hz), 69.11, 57.12, 52.45, 51.78 (d, J = 8.2 Hz), 21.61, 19.37. ^{31}P NMR (243 MHz, $CDCl_3$) δ -24.56 (s). HRMS (ESI): exact mass calculated for $C_{39}H_{40}FeN_2O_2PS$ $[M+H]^+$ 687.1892, found 687.1906.

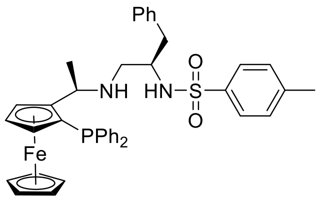

(R_C,S_P,R_C)-**L5**

Orange solid, 182 mg, 52% yield. $[\alpha]_D^{25}$ = -101.3 (c 0.5, CH_2Cl_2). ^{1}H NMR (600 MHz, $CDCl_3$) δ 7.56 – 7.48 (m, 4H), 7.37 (dt, J = 5.4, 1.5 Hz, 3H), 7.18 (d, J = 3.1 Hz, 4H), 7.17 – 7.12 (m, 8H), 6.87 – 6.80 (m, 2H), 4.86 (s, 1H), 4.41 (dt, J = 2.8, 1.6 Hz, 1H), 4.31 (t, J = 2.5 Hz, 1H), 3.97 (s, 5H), 3.92 (qd, J = 6.5, 2.7 Hz, 1H), 3.85 – 3.68 (m, 1H), 3.08 – 2.99 (m, 1H), 2.40 (s, 3H), 2.29 (qd, J = 12.2, 4.9 Hz, 2H), 2.23 (dd, J = 13.7, 6.3 Hz, 1H), 2.14 (dd, J = 13.7, 7.4 Hz, 1H), 1.28 (d, J = 6.5 Hz, 3H). ^{13}C NMR (151 MHz, $CDCl_3$) δ 142.90, 140.47 (d, J = 9.8 Hz), 137.79, 137.64, 137.33 (d, J = 9.0 Hz), 135.23, 135.09, 132.66, 132.53, 129.54 (d, J = 3.3 Hz), 129.30, 128.53, 128.49, 128.43, 128.35, 128.27, 128.21, 127.14, 126.33, 97.67 (d, J = 24.3 Hz), 74.85 (d, J = 8.3 Hz), 71.46 (d, J = 4.9 Hz), 69.79, 69.35, 69.29 (d, J = 4.8 Hz), 54.79, 51.24 (d, J = 8.9 Hz), 48.11, 39.00, 21.64, 19.88. ^{31}P NMR (243 MHz, $CDCl_3$) δ -24.51 (s). HRMS (ESI): exact mass calculated for $C_{40}H_{42}FeN_2O_2PS$ $[M+H]^+$ 701.2049, found 701.2034.

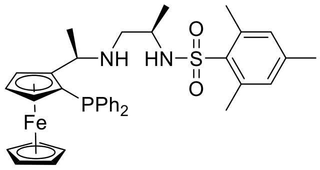

(R_C,S_P,R_C)-**L6**

Orange solid, 198 mg, 61% yield. $[\alpha]_D^{25}$ = -81.3 (c 0.5, CH_2Cl_2). ^{1}H NMR (600 MHz, $CDCl_3$) δ 7.52 – 7.49 (m, 2H), 7.40 – 7.34 (m, 3H), 7.21 – 7.07 (m, 5H), 6.91 (s, 2H), 5.26 (d, J = 4.7 Hz, 1H), 4.40 (dt, J = 3.0, 1.6 Hz, 1H), 4.29 (t, J = 2.5 Hz, 1H), 4.01 (s, 5H), 3.97 (dd, J = 6.6, 2.9 Hz, 1H), 3.81 – 3.77 (m, 1H), 2.59 (s, 6H), 2.52 (d, J = 6.9 Hz, 1H), 2.29 (s, 3H), 2.10 (qd, J = 11.9, 5.6 Hz, 2H), 1.36 (d, J = 6.5 Hz, 3H), 0.56 (d, J = 6.5 Hz, 3H). ^{13}C NMR (151 MHz, $CDCl_3$) δ 141.73, 139.92 (d, J = 9.8 Hz), 139.20, 136.93 (d, J = 9.0 Hz), 135.00, 134.86, 134.66, 132.87, 132.74, 131.89, 129.23, 128.45 (d, J = 3.0 Hz), 128.40, 128.29, 128.24, 97.18 (d, J = 23.8 Hz), 75.29 (d, J = 7.0 Hz), 71.44 (d, J = 4.9 Hz), 69.86, 69.17 (d, J = 4.6 Hz), 69.05, 51.22, 51.15 (d, J = 9.1 Hz), 49.13, 23.20, 21.06, 19.51, 18.57. ^{31}P NMR (243 MHz, $CDCl_3$) δ -24.95 (s). HRMS (ESI): exact mass calculated for $C_{36}H_{42}FeN_2O_2PS$ $[M+H]^+$ 653.2049, found 653.2058.

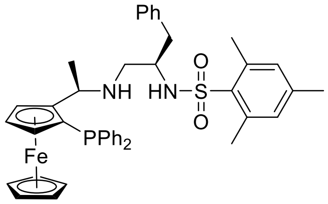

(R_C,S_P,R_C)-**L7**

Orange solid, 149 mg, 41% yield. $[\alpha]_D^{25}$ = -129.2 (*c* 0.5, CH_2Cl_2). ^{1}H NMR (600 MHz, $CDCl_3$) δ 7.55 – 7.48 (m, 2H), 7.39 – 7.34 (m, 3H), 7.30 – 7.25 (m, 1H), 7.22 – 7.13 (m, 4H), 7.09 – 7.03 (m, 1H), 7.04 – 6.98 (m, 2H), 6.78 (d, *J* = 1.0 Hz, 2H), 6.78 – 6.73 (m, 2H), 5.84 (s, 1H), 4.52 – 4.38 (m, 1H), 4.29 (t, *J* = 2.5 Hz, 1H), 4.01 (s, 5H), 3.93 (dd, *J* = 6.5, 2.9 Hz, 1H), 3.82 – 3.75 (m, 1H), 3.47 (dd, *J* = 8.5, 4.3 Hz, 1H), 2.52 (s, 1H), 2.43 (s, 6H), 2.40 – 2.29 (m, 3H), 2.24 (s, 3H), 1.39 (d, *J* = 6.4 Hz, 3H). ^{13}C NMR (151 MHz, $CDCl_3$) δ 141.73, 139.99 (d, *J* = 10.8 Hz), 139.37, 139.35, 137.02 (d, *J* = 9.4 Hz), 134.97, 134.83, 134.24, 133.00, 132.87, 131.62, 129.21, 128.62, 128.58 (d, *J* = 3.5 Hz), 128.30, 128.24, 127.98, 127.19, 126.77, 96.99 (d, *J* = 23.7 Hz), 75.59 (d, *J* = 7.0 Hz), 71.52 (d, *J* = 4.9 Hz), 69.87, 69.84, 69.19 (d, *J* = 4.8 Hz), 69.08, 57.44, 53.04, 51.21 (d, *J* = 9.4 Hz), 22.99, 20.99, 19.65. ^{31}P NMR (243 MHz, $CDCl_3$) δ -24.84 (s). HRMS (ESI): exact mass calculated for $C_{42}H_{46}FeN_2O_2PS$ $[M+H]^+$ 729.2362, found 729.2354.

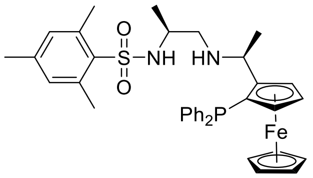

(S_C,R_P,S_C)-**L6**

Orange solid, 150 mg, 46% yield. $[\alpha]_D^{25}$ = +78.3 (*c* 0.5, CH_2Cl_2). ^{1}H NMR (600 MHz, $CDCl_3$) δ 7.54 – 7.49 (m, 2H), 7.38 – 7.35 (m, 3H), 7.19 – 7.10 (m, 5H), 6.91 (s, 2H), 5.27 (s, 1H), 4.48 – 4.35 (m, 1H), 4.29 (t, *J* = 2.5 Hz, 1H), 4.01 (s, 5H), 3.99 – 3.95 (m, 1H), 3.79 (dt, *J* = 2.3, 1.1 Hz, 1H), 2.60 (s, 6H), 2.52 (q, *J* = 6.2 Hz, 1H), 2.29 (s, 3H), 2.11 (qd, *J* = 11.9, 5.6 Hz, 2H), 1.37 (d, *J* = 6.5 Hz, 3H), 0.56 (d, *J* = 6.5 Hz, 3H). ^{13}C NMR (151 MHz, $CDCl_3$) δ 141.72, 139.91 (d, *J* = 10.0 Hz), 139.17, 136.93 (d, *J* = 9.0 Hz), 134.98, 134.84, 134.66, 132.85, 132.73, 131.87, 129.22, 128.44 (d, *J* = 2.9 Hz), 128.52 – 128.38 (m), 128.38, 128.27, 128.22, 97.15 (d, *J* = 23.9 Hz), 75.28 (d, *J* = 7.0 Hz), 71.42 (d, *J* = 4.9 Hz), 69.85, 69.16 (d, *J* = 4.8 Hz), 69.05, 51.19, 51.17, 51.11, 49.11, 23.18, 21.04, 19.49, 18.55. ^{31}P NMR (243 MHz, $CDCl_3$) δ -24.96 (s). HRMS (ESI): exact mass calculated for $C_{36}H_{42}FeN_2O_2PS$ $[M+H]^+$ 653.2049, found 653.2032.

2.2. Preparation of substrates

General synthesis of N-Boc-β-amino ketones[7]

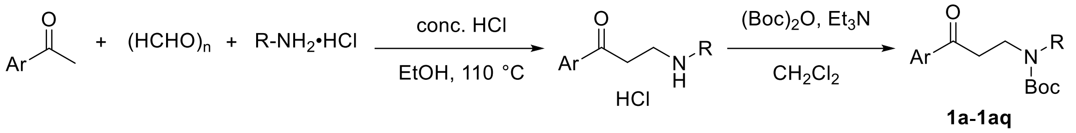

1a-1aq

A mixture of ketone (15 mmol), alkylamine hydrochloride (16.5 mmol), paraformaldehyde (630.0 mg, 21 mmol) and conc. HCl (75 μL) in ethanol (7.5 mL) was heated in a sealed tube at 110 °C for 16 h. After cooling to room temperature, the solvent was removed in vacuo. To the residue was added ethyl acetate (20 mL) and stirred for 30 minutes, then filtered. The filter cake was collected without further purification and dissolved in dichloromethane (22.5 mL), then triethylamine (3.0 g, 30 mmol) and Boc_2O (3.2 g, 15 mmol) were added dropwise to the reaction system in turn. After the addition was completed, the mixture was stirred for 2 h at room temperature. Then 2 M HCl aqueous solution was added to adjust the pH to neutral. The organic layer was separated, and the aqueous layer was extracted with CH_2Cl_2 (20 mL × 2). The combined organic layer was washed with saturated brine (20 mL), dried over anhydrous Na_2SO_4, then filtered, and concentrated in vacuo. The residue was purified by silica gel column chromatography to afford the corresponding product **1a**–**1aq** (30%–67% yields).

General synthesis of N-alkoxycarbonyl β-amino ketones[8]

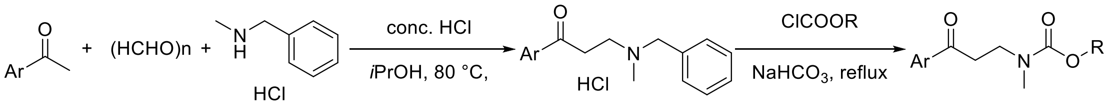

3e•HCl, **3j**•HCl

3a-3c, **3h**

A mixture of ketone (15 mmol), *N*-methylbenzylamine hydrochloride (2.0 g, 16.5 mmol), paraformaldehyde (675.0 mg, 22.5 mmol) and conc. HCl (150 μL) in *i*PrOH (4.5 mL) was heated in a sealed tube at 80 °C overnight. After cooling to room temperature, the precipitated solid was collected by filtration, washed with ethanol, and dried at 50 °C under vacuum to give the β-amino ketone hydrochloride **3e**·HCl, **3j**·HCl.

To the solution of β-amino ketone hydrochloride (3 mmol) and $NaHCO_3$ (630.0 mg, 7.5 mmol) in toluene (6 mL), was added chloroformate (4.5 mmol). The mixture was stirred for 3 h under reflux, diluted with water and then extracted with toluene. The combined organic layer was dried over anhydrous Na_2SO_4, filtered, and concentrated in vacuo. The residue was purified by silica gel column chromatography to afford the corresponding product **3a**–**3c** and **3h** (81%–85% yields for two steps).

General synthesis of β-tertiary-amino ketone hydrochlorides[9-10]

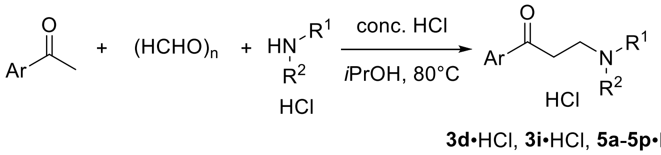

3d•HCl, **3i**•HCl, **5a-5p**•HCl

A mixture of ketone (15 mmol), secondary amine hydrochloride (16.5 mmol), paraformaldehyde (675.0 mg, 22.5 mmol) and conc. HCl (150 μL) in *i*PrOH (4.5 mL) was heated in a sealed tube at 80 °C overnight. After cooling to room temperature, the precipitated solid was collected by filtration, washed with ethanol, and dried at 50 °C under vacuum to give the β-amino ketone hydrochloride **3d**·HCl, **3i**·HCl, **5a**–**5p**·HCl (60–85% yields).

General synthesis of β-tertiary-amino ketones[11]

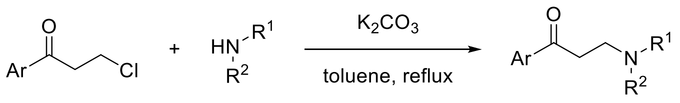

5q-5ad

A mixture of ketone (2.5 mmol), secondary amine (2.5 mmol) and potassium carbonate (691.0 mg, 5 mmol) in toluene (10 mL) was refluxed for 3 h. After cooling to room temperature, the solvent was removed in vacuo. The residue was purified by silica gel column chromatography to afford the β-tertiary-amino ketone **5q**–**5ad** (60–95% yields).

Synthesis of β-secondary-amino ketone hydrochlorides[7]

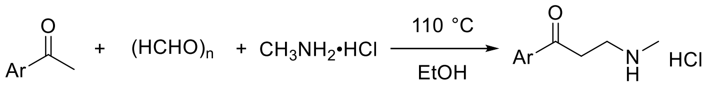

3f•HCl, **3k**•HCl

A mixture of ketone (15 mmol), methylamine hydrochloride (1.1 g, 16.5 mmol), paraformaldehyde (630.0 mg, 21 mmol) and conc. HCl (75 μL) in ethanol (7.5 mL) was heated in a sealed tube at 110 °C for 16 h. After cooling to room temperature, the solvent was removed in vacuo. To the residue was added ethyl acetate (30 mL) and stirred for 30 minutes, then filtered. The filter cake was collected and purified by silica gel column chromatography to afford the β-secondary-amino ketone hydrochloride **3f**·HCl (1.38 g, 45% yield), **3k**·HCl (1.52 g, 51% yield).

2.3. Asymmetric hydrogenation of β-amino ketones

General procedure for the asymmetric hydrogenation of 1 (S/C=1000): Under nitrogen atmosphere, $[Ir(COD)Cl]_2$ (1.4 mg, 0.002 mmol), (*R*$_C$,*S*$_P$,*R*$_C$)-**L6** (2.8 mg, 0.0042 mmol), and anhydrous *i*PrOH (1 mL) were added to an oven-dried 10 mL schlenk tube. The mixture was stirred at room temperature for 2.5 h to give an orange solution. A part of catalyst solution (100 μL, 0.0004 mmol) was transferred into a 10 mL vial containing *N*-Boc-β-amino ketone **1** (0.4 mmol), NaO*t*Bu (1.9 mg, 0.02 mmol), and anhydrous toluene (2.0 mL). The vials were transferred to an autoclave, and the autoclave was purged with nitrogen and hydrogen three times in sequence, then charged with 30 atm of H_2 and stirred for 16 h at room temperature. After hydrogen pressure was slowly released, the solvent was evaporated and the residue was

purified by silica gel column chromatography to give the corresponding hydrogenation product **2**. Then the enantiomeric excesses were determined by HPLC analysis.

General procedure for the asymmetric hydrogenation of 3 (S/C=1000): Under nitrogen atmosphere, $[Ir(COD)Cl]_2$ (1.4 mg, 0.002 mmol), (R_C,S_P,R_C)-**L6** or (S_C,R_P,S_C)-**L6** (2.8 mg, 0.0042 mmol), and anhydrous *i*PrOH (1 mL) were added to an oven-dried 10 mL schlenk tube. The mixture was stirred at room temperature for 2.5 h to give an orange solution. A part of catalyst solution (100 μL, 0.0004 mmol) was transferred into a 10 mL vial containing ketone **3** or **3**·HCl (0.4 mmol), NaO*t*Bu (1.9 mg for **3a**–**3c** and **3g**–**3h**, 42.2 mg for **3d**–**3f**·HCl and **3i**–**3k**·HCl), and anhydrous toluene (2.0 mL). The vials were transferred to an autoclave, and the autoclave was purged with nitrogen and hydrogen three times in sequence, then charged with 30 atm of H_2 and stirred for 16 h at room temperature. After hydrogen pressure was slowly released, the solvent was evaporated and the residue was purified by silica gel column chromatography to give the corresponding hydrogenation product **4**. Then the enantiomeric excesses were determined by HPLC analysis.

General procedure for the asymmetric hydrogenation of 5 (S/C=1000): Under nitrogen atmosphere, $[Ir(COD)Cl]_2$ (1.4 mg, 0.002 mmol), (R_C,S_P,R_C)-**L6** (2.8 mg, 0.0042 mmol), and anhydrous *i*PrOH (1 mL) were added to an oven-dried 10 mL schlenk tube. The mixture was stirred at room temperature for 2.5 h to give an orange solution. A part of catalyst solution (100 μL, 0.0004 mmol) was transferred into a 10 mL vial containing β-tertiary-amino ketone **5** or **5**·HCl (0.4 mmol), NaO*t*Bu (42.2 mg for **5a**–**5p**·HCl, 1.9 mg for **5q**–**5ad**), and anhydrous toluene (3.0 mL). The vials were transferred to an autoclave, and the autoclave was purged with nitrogen and hydrogen three times in sequence, then charged with 35 atm of H_2 and stirred for 16 h at room temperature. After hydrogen pressure was slowly released, the solvent was evaporated and the residue was purified by silica gel column chromatography to give the corresponding hydrogenation product **6**. Then the enantiomeric excesses were determined by HPLC analysis.

General procedure for the asymmetric hydrogenation of 1a (S/C=50000): Under nitrogen atmosphere, $[Ir(COD)Cl]_2$ (1.4 mg, 0.002 mmol), (R_C,S_P,R_C)-**L6** (2.8 mg, 0.0042 mmol), and anhydrous *i*PrOH (1 mL) were added to an oven-dried 10 mL schlenk tube. The mixture was stirred at room temperature for 2.5 h to give an orange solution. A part of catalyst solution (100 μL, 0.0004 mmol) was transferred into a 30 mL hydrogenation vessel containing **1a** (5.38 g, 20.0 mmol), NaO*t*Bu (64.0 mg, 0.67 mmol), and anhydrous toluene (20 mL). The vessel was transferred to an autoclave, and the autoclave was purged with nitrogen and hydrogen three times in sequence, then charged with 60 atm of H_2 and stirred for 40 h at room temperature. After hydrogen pressure was slowly released, the solvent was evaporated and the residue was

purified by silica gel column chromatography to give the corresponding hydrogenation product **2a** (5.24 g, 97% yield, 99% ee).

General procedure for the asymmetric hydrogenation of 3g (S/C=20000): Under nitrogen atmosphere, $[Ir(COD)Cl]_2$ (1.4 mg, 0.002 mmol), (S_C,R_P,S_C)-**L6** (2.8 mg, 0.0042 mmol), and anhydrous *i*PrOH (1 mL) were added to an oven-dried 10 mL schlenk tube. The mixture was stirred at room temperature for 2.5 h to give an orange solution. A part of catalyst solution (100 μL, 0.0004 mmol) was transferred into a 30 mL hydrogenation vessel containing **3g** (2.11 g, 8.0 mmol), NaO*t*Bu (32.0 mg, 0.33 mmol), and anhydrous toluene (12 mL). The vessel was transferred to an autoclave, and the autoclave was purged with nitrogen and hydrogen three times in sequence, then charged with 60 atm of H_2 and stirred for 40 h at room temperature. After hydrogen pressure was slowly released, the solvent was evaporated and the residue was purified by silica gel column chromatography to give the corresponding hydrogenation product **4g** (1.95 g, 92% yield, 98% ee).

General procedure for the asymmetric hydrogenation of 5ac (S/C=20000): Under nitrogen atmosphere, $[Ir(COD)Cl]_2$ (1.4 mg, 0.002 mmol), (R_C,S_P,R_C)-**L6** (2.8 mg, 0.0042 mmol), and anhydrous *i*PrOH (1 mL) were added to an oven-dried 10 mL schlenk tube. The mixture was stirred at room temperature for 2.5 h to give an orange solution. A part of catalyst solution (100 μL, 0.0004 mmol) was transferred into a 30 mL hydrogenation vessel containing **5ac** (2.59 g, 8.0 mmol), NaO*t*Bu (32.0 mg, 0.33 mmol), and anhydrous toluene (12 mL). The vessel was transferred to an autoclave, and the autoclave was purged with nitrogen and hydrogen three times in sequence, then charged with 60 atm of H_2 and stirred for 40 h at room temperature. After hydrogen pressure was slowly released, the solvent was evaporated and the residue was purified by silica gel column chromatography to give the corresponding hydrogenation product **6ac** (2.47 g, 95% yield, 99% ee).

tert-butyl (*S*)-(3-hydroxy-3-(thiophen-2-yl)propyl)(methyl)carbamate (2a)

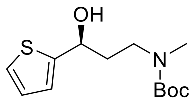

White solid, 98% yield, 99% ee. $[\alpha]_D^{25}$ = 26.8 (*c* 2.0, CH_2Cl_2). The ee was determined by HPLC on Chiralpak AD-H column, hexane:isopropanol = 95:5; flow rate = 0.8 mL/min; UV detection at 220 nm; t_R = 14.242 min (major), t_R = 16.277 min (minor). ^{1}H NMR (400 MHz, CD_3OD) δ 7.29 (d, *J* = 4.9 Hz, 1H), 7.06 – 6.85 (m, 2H), 4.88 (t, *J* = 6.6 Hz, 1H), 3.45 – 3.19 (m, 2H), 2.85 (s, 3H), 2.02 (q, *J* = 7.6, 7.1 Hz, 2H), 1.44 (s, 9H). ^{13}C NMR (101 MHz, CD_3OD) δ 156.21, 148.66, 126.10, 123.87, 123.22, 79.58, 67.08, 45.68, 37.28, 33.43, 27.33. HRMS (EI): exact mass calculated for $C_{13}H_{21}NO_3S$ $[M]^+$ 271.1242, found 271.1246.

tert-butyl (*S*)-(3-hydroxy-3-(thiophen-3-yl)propyl)(methyl)carbamate (2b)

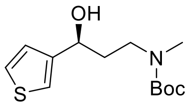

Colorless oil, 93% yield, 98% ee. $[\alpha]_D^{25}$ = 7.9 (*c* 2.0, CH_2Cl_2). The ee was determined by HPLC on Chiralpak AD-H column, hexane:isopropanol = 95:5; flow rate = 0.8 mL/min; UV detection at 220 nm; t_R = 19.362 min (major), t_R = 27.392 min (minor). ^{1}H NMR (400 MHz, CD_3OD) δ 7.35 (dd, *J* = 5.1, 3.0 Hz, 1H), 7.24 (d, *J* = 2.8 Hz, 1H), 7.09 (dd, *J* = 5.0, 1.3 Hz, 1H), 4.71 (t, *J* = 6.6 Hz, 1H), 3.45 – 3.16 (m, 2H), 2.83 (s, 3H), 2.06 – 1.83 (m, 2H), 1.43 (s, 9H). ^{13}C NMR (101 MHz, CD_3OD) δ 156.23, 146.01, 125.47, 120.21, 79.53, 67.43, 45.70, 36.19, 33.45, 27.41. HRMS (EI): exact mass calculated for $C_{13}H_{21}NO_3S$ $[M]^+$ 271.1242, found 271.1241.

tert-butyl (*S*)-(3-(5-chlorothiophen-2-yl)-3-hydroxypropyl)(methyl)carbamate (2c)

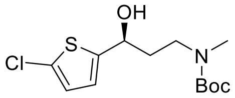

Colorless oil, 93% yield, 99% ee. $[\alpha]_D^{25}$ = 30.2 (*c* 2.0, CH_2Cl_2). The ee was determined by HPLC on Chiralpak AD-H column, hexane:isopropanol = 95:5; flow rate = 0.8 mL/min; UV detection at 220 nm; t_R = 13.572 min (major), t_R = 16.656 min (minor). ^{1}H NMR (400 MHz, CD_3OD) δ 6.94 – 6.66 (m, 2H), 4.76 (t, *J* = 6.5 Hz, 1H), 3.32 (s, 2H), 2.85 (s, 3H), 2.02 – 1.90 (m, 2H), 1.44 (s, 9H). ^{13}C NMR (101 MHz, CD_3OD) δ 156.19, 148.34, 128.01, 125.49, 122.53, 79.62, 67.25, 45.23, 36.98, 33.50, 27.34. HRMS (EI): exact mass calculated for $C_{13}H_{20}ClNO_3S$ $[M]^+$ 305.0852, found 305.0854.

tert-butyl (*S*)-(3-(4-bromothiophen-2-yl)-3-hydroxypropyl)(methyl)carbamate (2d)

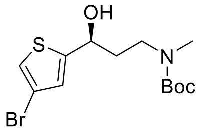

Colorless oil, 93% yield, 99% ee. $[\alpha]_D^{25}$ = 23.2 (*c* 2.0, CH_2Cl_2). The ee was determined by HPLC on Chiralpak AD-H column, hexane:isopropanol = 95:5; flow rate = 0.8 mL/min; UV detection at 220 nm; t_R = 15.212 min (major), t_R = 18.439 min (minor). ^{1}H NMR (400 MHz, CD_3OD) δ 6.93 (d, *J* = 3.8 Hz, 1H), 6.77 (d, *J* = 3.7 Hz, 1H), 4.79 (t, *J* = 6.5 Hz, 1H), 3.33 (s, 2H), 2.85 (s, 3H), 2.12 – 1.89 (m, 2H), 1.44 (s, 9H). ^{13}C NMR (101 MHz, CD_3OD) δ 156.19, 151.16, 129.27, 123.56, 110.23, 79.63, 67.23, 45.46, 37.00, 33.45, 27.33. HRMS (EI): exact mass calculated for $C_{13}H_{20}ClNO_3S$ [M]+ 349.0347, found 349.0435.

tert-butyl (*S*)-(3-hydroxy-3-(5-methylthiophen-2-yl)propyl)(methyl)carbamate (2e)

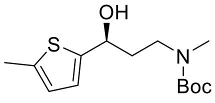

Colorless oil, 85% yield, >99% ee. $[\alpha]_D^{25}$ = 23.6 (*c* 2.0, CH_2Cl_2). The ee was determined by HPLC on Chiralpak AD-H column, hexane:isopropanol = 95:5; flow rate = 0.8 mL/min; UV detection at 220 nm; t_R = 20.532 min (major), t_R = 24.815 min (minor). ^{1}H NMR (400 MHz, CD_3OD) δ 6.74 (d, *J* = 3.4 Hz, 1H), 6.59 (d, *J* = 3.4 Hz, 1H), 4.75 (t, *J* = 6.7 Hz, 1H), 3.35 – 3.21 (m, 2H), 2.84 (s, 3H), 2.43 (s, 3H), 1.98 (d, *J* = 7.4 Hz, 2H), 1.43 (s, 9H). ^{13}C NMR (101 MHz, CD_3OD) δ 156.61, 148.76, 126.14, 123.85, 123.16, 79.72, 67.21, 57.83, 46.39, 37.70, 36.58, 33.49, 27.40. HRMS (EI): exact mass calculated for $C_{14}H_{23}NO_3S$ $[M]^+$ 285.1399, found 285.1396.

tert-butyl (*S*)-(3-(2,5-dichlorothiophen-3-yl)-3-hydroxypropyl)(methyl)carbamate (2f)

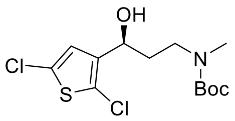

Colorless oil, 87% yield, 98% ee. $[\alpha]_D^{25}$ = 29.6 (*c* 2.0, CH_2Cl_2). The ee was determined by HPLC on Chiralpak AD-H column, hexane:isopropanol = 95:5; flow rate = 0.8 mL/min; UV detection at 220 nm; t_R = 10.298 min (major), t_R = 11.302 min (minor). 1H NMR (400 MHz, CD_3OD) δ 6.96 (s, 1H), 4.74 (t, *J* = 6.7 Hz, 1H), 3.41 – 3.18 (m, 2H), 2.86 (s, 3H), 1.88 (q, *J* = 6.8 Hz, 2H), 1.44 (s, 9H). ^{13}C NMR (101 MHz, CD_3OD) δ 156.18, 142.45, 126.24, 125.01, 120.87, 79.61, 64.71, 45.40, 35.23, 33.69, 27.34. HRMS (EI): exact mass calculated for $C_{13}H_{19}Cl_2NO_3S$ $[M]^+$ 339.0463, found 339.0463.

tert-butyl (*S*)-(3-(benzo[b]thiophen-2-yl)-3-hydroxypropyl)(methyl)carbamate (2g)

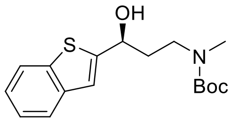

Colorless oil, 99% yield, 99% ee. $[\alpha]_D^{25}$ = 32.8 (*c* 2.0, CH_2Cl_2). The ee was determined by HPLC on Chiralpak AD-H column, hexane:isopropanol = 95:5; flow rate = 0.8 mL/min; UV detection at 220 nm; t_R = 36.455 min (major), t_R = 40.653 min (minor). ^{1}H NMR (400 MHz, CD_3OD) δ 7.79 (d, *J* = 7.8 Hz, 1H), 7.74 – 7.63 (m, 1H), 7.34 – 7.21 (m, 2H), 7.21 (s, 1H), 3.41 – 3.32 (m, 2H), 2.84 (s, 3H), 2.12 – 2.00 (m, 2H), 1.40 (s, 9H). ^{13}C NMR (101 MHz, CD_3OD) δ 156.21, 149.63, 139.73, 139.29, 123.88, 123.73, 123.08, 121.94, 119.65, 79.61, 67.62, 45.57, 36.94, 33.46, 27.37. HRMS (EI): exact mass calculated for $C_{17}H_{23}Cl_2NO_3S$ $[M]^+$ 321.1399, found 321.1396.

tert-butyl (*S*)-(3-hydroxy-3-phenylpropyl)(methyl)carbamate (2h)

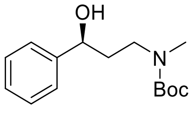

Colorless oil, 92% yield, 98% ee. $[\alpha]_D^{25}$ = 1.6 (*c* 2.0, CH_2Cl_2). The ee was determined by HPLC on Chiralpak AD-H column, hexane:isopropanol = 95:5; flow rate = 0.8 mL/min; UV detection at 220 nm; t_R = 15.377 min (major), t_R = 18.301 min (minor). ^{1}H NMR (400 MHz, CD_3OD) δ 7.37 – 7.29 (m, 4H), 7.27 – 7.19 (m, 1H), 4.61 (t, *J* = 6.6 Hz, 1H), 3.45 – 3.15 (m, 2H), 2.83 (s, 3H), 1.92 (d, *J* = 7.2 Hz, 2H), 1.41 (s, 9H). ^{13}C NMR (101 MHz, CD_3OD) δ 156.22, 144.66, 128.00, 126.95, 125.53, 79.50, 71.32, 45.74, 36.87, 33.45, 27.35. HRMS (EI): exact mass calculated for $C_{15}H_{23}NO_3$ $[M]^+$ 265.1678, found 265.1675.

tert-butyl (*S*)-(3-(3-chlorophenyl)-3-hydroxypropyl)(methyl)carbamate (2i)

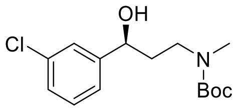

Colorless oil, 94% yield, 99% ee. $[\alpha]_D^{25}$ = 3.3 (*c* 2.0, CH_2Cl_2). The ee was determined by HPLC on Chiralpak AD-H column, hexane:isopropanol = 95:5; flow rate = 0.8 mL/min; UV detection at 220 nm; t_R = 12.761 min (major), t_R = 15.058 min (minor). ^{1}H NMR (400 MHz, CD_3OD) δ 7.38 (s, 1H), 7.34 – 7.20 (m, 3H), 4.61 (t, *J* = 6.5 Hz, 1H), 3.42 – 3.18 (m, 2H), 2.84 (s, 3H), 1.97 – 1.83 (m, 2H), 1.41 (s, 9H). ^{13}C NMR (101 MHz, CD_3OD) δ 156.23, 147.33, 133.90, 129.52, 126.88, 125.56, 123.90, 79.56, 70.56, 45.47, 36.69, 33.39, 27.32. HRMS (EI): exact mass calculated for $C_{15}H_{22}ClNO_3$ $[M]^+$ 299.1288, found 299.1286.

tert-butyl (*S*)-(3-(3-bromophenyl)-3-hydroxypropyl)(methyl)carbamate (2j)

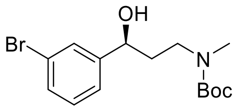

Colorless oil, 91% yield, 98% ee. $[\alpha]_D^{25}$ = 2.6 (*c* 2.0, CH_2Cl_2). The ee was determined by HPLC on Chiralpak AD-H column, hexane:isopropanol = 95:5; flow rate = 0.8 mL/min; UV detection at 220 nm; t_R = 13.802 min (major), t_R = 16.389 min (minor). ^{1}H NMR (400 MHz, CD_3OD) δ 7.53 (s, 1H), 7.39 (d, *J* = 7.8 Hz, 1H), 7.31 (d, *J* = 7.7 Hz, 1H), 7.24 (t, *J* = 7.7 Hz, 1H), 4.60 (t, *J* = 6.5 Hz, 1H), 3.43 – 3.18 (m, 2H), 2.84 (s, 3H), 1.90 (q, *J* = 7.1 Hz, 2H), 1.41 (s, 9H). ^{13}C NMR (101 MHz, CD_3OD) δ 156.21, 147.60, 129.85 (d, *J* = 7.1 Hz), 128.55, 124.35, 122.02, 79.56, 70.50, 45.48, 36.72, 33.38, 27.35. HRMS (EI): exact mass calculated for $C_{17}H_{23}BrNO_3$ $[M]^+$ 343.0783, found 343.0785.

tert-butyl (*S*)-(3-hydroxy-3-(m-tolyl)propyl)(methyl)carbamate (2k)

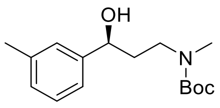

Colorless oil, 86% yield, 99% ee. $[\alpha]_D^{25}$ = 3.8 (*c* 2.0, CH_2Cl_2). The ee was determined by HPLC on Chiralpak AD-H column, hexane:isopropanol = 95:5; flow rate = 0.8 mL/min; UV detection at 220 nm; t_R = 21.409 min (major), t_R = 28.365 min (minor). ^{1}H NMR (400 MHz, CD_3OD) δ 7.27 – 7.10 (m, 3H), 7.06 (d, *J* = 7.5 Hz,1H), 4.57 (t, *J* = 6.6 Hz, 1H), 3.25 (s, 2H), 2.83 (s, 3H), 2.33 (s, 3H), 1.90 (s, 2H), 1.41 (s, 9H). ^{13}C NMR (101 MHz, CD_3OD) δ 156.23, 144.54, 137.63, 127.89, 127.61, 126.17, 122.63, 79.49, 71.37, 45.78, 36.72, 33.42, 27.31, 20.16. HRMS (EI): exact mass calculated for $C_{16}H_{25}NO_3$ $[M]^+$ 279.1834, found 279.1836.

tert-butyl (*S*)-(3-hydroxy-3-(3-methoxyphenyl)propyl)(methyl)carbamate (2l)

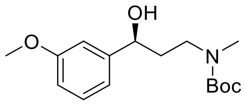

Colorless oil, 85% yield, 99% ee. $[\alpha]_D^{25}$ = 5.5 (*c* 2.0, CH_2Cl_2). The ee was determined by HPLC on Chiralpak AD-H column, hexane:isopropanol = 95:5; flow rate = 0.8 mL/min; UV detection at 220 nm; t_R = 25.893 min (major), t_R = 31.852 min (minor). ^{1}H NMR (400 MHz, CD_3OD) δ 7.23 (t, *J* = 7.8 Hz, 1H), 6.97 – 6.86 (m, 2H), 6.81 – 6.69 (m, 1H), 4.58 (t, *J* = 6.5 Hz, 1H), 3.78 (s, 3H), 3.26 (s, 2H), 2.83 (s, 3H), 1.91 (d, *J* = 6.9 Hz, 2H), 1.41 (s, 9H). ^{13}C NMR (101 MHz, CD_3OD) δ 159.87, 156.23, 146.33, 128.99, 117.77, 112.28, 111.08, 79.51, 71.24, 54.20, 45.72, 36.80, 33.43, 27.32. HRMS (EI): exact mass calculated for $C_{16}H_{25}NO_4$ $[M]^+$ 295.1784, found 295.1781.

tert-butyl (*S*)-(3-(4-fluorophenyl)-3-hydroxypropyl)(methyl)carbamate (2m)

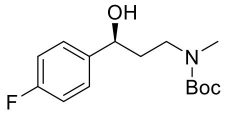

Colorless oil, 77% yield, 98% ee. The ee was determined by HPLC on Chiralpak AD-H column, hexane: sopropanol = 95:5; flow rate = 0.8 mL/min; UV detection at 220 nm; t_R = 14.123 min (major), t_R = 18.333 min (minor). ^{1}H NMR (400 MHz, CD_3OD) δ 7.36 (dd, *J* = 8.4, 5.4 Hz, 2H), 7.05 (t, *J* = 8.6 Hz, 2H), 4.61 (t, *J* = 6.6 Hz, 1H), 3.26 (d, *J* = 7.2 Hz, 2H), 2.84 (s, 3H), 1.89 (s, 2H), 1.42 (s, 9H). ^{13}C NMR (101 MHz, CD_3OD) δ 162.06 (d, *J* = 243.5 Hz), 160.85, 156.22, 140.74 (d, *J* = 3.2 Hz), 127.36 (d, *J* = 8.0 Hz), 114.55 (d, *J* = 21.6 Hz), 79.53, 70.61, 45.65, 36.79, 33.41, 27.30. HRMS (EI): exact mass calculated for $C_{15}H_{22}FNO_3$ $[M]^+$ 283.1584, found 283.1586.

tert-butyl (*S*)-(3-(4-chlorophenyl)-3-hydroxypropyl)(methyl)carbamate (2n)

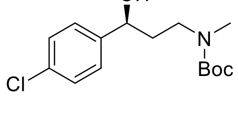

Colorless oil, 90% yield, 98% ee. $[\alpha]_D^{25}$ = 6.6 (c 2.0, CH_2Cl_2). The ee was determined by HPLC on Chiralpak AD-H column, hexane:isopropanol = 95:5; flow rate = 0.8 mL/min; UV detection at 220 nm; t_R = 14.623 min (major), t_R = 19.292 min (minor). ^{1}H NMR (400 MHz, CD_3OD) δ 7.37 – 7.29 (m, 4H), 4.61 (t, J = 6.5 Hz, 1H), 3.36 – 3.23 (m, 2H), 2.83 (s, 3H), 1.90 (q, J = 7.8, 7.4 Hz, 2H), 1.43 (s, 9H). ^{13}C NMR (101 MHz, CD_3OD) δ 156.20, 143.62, 132.50, 128.03, 127.16, 79.54, 70.54, 46.98, 36.81, 33.43, 27.32. HRMS (EI): exact mass calculated for $C_{15}H_{22}ClNO_3$ $[M]^+$ 299.1288, found 299.1286.

tert-butyl (*S*)-(3-(4-bromophenyl)-3-hydroxypropyl)(methyl)carbamate (2o)

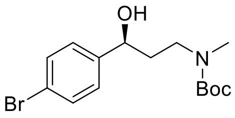

Colorless oil, 92% yield, 97% ee. $[\alpha]_D^{25}$ = 9.9 (c 2.0, CH_2Cl_2). The ee was determined by HPLC on Chiralpak AD-H column, hexane:isopropanol = 95:5; flow rate = 0.8 mL/min; UV detection at 220 nm; t_R = 16.628 min (major), t_R = 22.129 min (minor). ^{1}H NMR (400 MHz, DMSO) δ 7.51 (d, J = 8.4 Hz, 2H), 7.29 (d, J = 8.4 Hz, 2H), 5.33 (d, J = 4.5 Hz, 1H), 4.52 (q, J = 5.4 Hz, 1H), 3.26 – 3.03 (m, 2H), 2.75 (s, 3H), 1.76 (d, J = 8.0 Hz, 2H), 1.34 (s, 9H). ^{13}C NMR (101 MHz, DMSO) δ 155.18, 145.73, 131.34, 128.41, 120.07, 78.72, 70.00, 45.70, 37.61, 34.45, 28.52. HRMS (EI): exact mass calculated for $C_{15}H_{22}BrNO_3$ $[M]^+$ 344.0783, found 344.0786.

tert-butyl (*S*)-(3-hydroxy-3-(p-tolyl)propyl)(methyl)carbamate (2p)

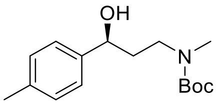

Colorless oil, 90% yield, 99% ee. $[\alpha]_D^{25}$ = 5.3 (c 2.0, CH_2Cl_2). The ee was determined by HPLC on Chiralpak AD-H column, hexane:isopropanol = 95:5; flow rate = 0.8 mL/min; UV detection at 220 nm; t_R = 18.462 min (major), t_R = 20.777 min (minor). ^{1}H NMR (400 MHz, CD_3OD) δ 7.22 (d, J = 7.9 Hz, 2H), 7.13 (d, J =7.6 Hz, 2H), 4.56 (t, J = 6.6 Hz, 1H), 3.34 – 3.19 (m, 1H), 2.82 (s, 3H), 2.30 (s, 3H), 1.91 (s, 2H), 1.42 (s, 9H). ^{13}C NMR (101 MHz, CD_3OD) δ 156.20, 141.57, 136.62, 128.60, 125.54, 79.48, 71.19, 45.77, 36.81, 33.46, 27.36, 19.83. HRMS (EI): exact mass calculated for $C_{16}H_{25}NO_3$ $[M]^+$ 279.1834, found 279.1836.

tert-butyl (*S*)-(3-(4-(tert-butyl)phenyl)-3-hydroxypropyl)(methyl)carbamate (2q)

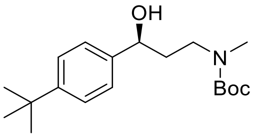

Colorless oil, 89% yield, 98% ee. $[\alpha]_D^{25}$ = 6.2 (c 2.0, CH_2Cl_2). The ee was determined by HPLC on Chiralpak AD-H column, hexane:isopropanol = 95:5; flow rate = 0.8 mL/min; UV detection

at 220 nm; t_R = 13.969 min (major), t_R = 16.705 min (minor). ^{1}H NMR (400 MHz, CD_3OD) δ 7.37 (d, J = 8.1 Hz, 2H), 7.27 (d, J = 8.2 Hz, 2H), 4.58 (t, J = 6.6 Hz, 1H), 3.36-3.17 (m, 2H), 2.82 (s, 3H), 1.92 (d, J = 8.7 Hz, 2H), 1.48 – 1.36 (m, 9H), 1.30 (s, 9H). ^{13}C NMR (101 MHz, CD_3OD) δ 156.20, 149.93, 141.53, 125.34, 124.85, 79.47, 71.12, 45.66, 36.74, 33.94, 33.35, 30.50, 27.38. HRMS (EI): exact mass calculated for $C_{19}H_{31}NO_3$ $[M]^+$ 321.2304, found 321.2307.

tert-butyl (*S*)-(3-hydroxy-3-(4-methoxyphenyl)propyl)(methyl)carbamate (2r)

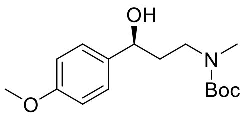

Colorless oil, 87% yield, 97% ee. $[\alpha]_D^{25}$ = 7.6 (c 2.0, CH_2Cl_2). The ee was determined by HPLC on Chiralpak AD-H column, hexane:isopropanol = 95:5; flow rate = 0.8 mL/min; UV detection at 220 nm; t_R = 27.915 min (major), t_R = 34.908 min (minor). ^{1}H NMR (400 MHz, CD_3OD) δ 7.26 (d, J = 8.5 Hz, 2H), 6.88 (d, J = 8.4 Hz, 2H), 4.55 (t, J = 6.7 Hz, 1H), 3.76 (s, 3H), 3.39 – 3.14 (m, 2H), 2.82 (s, 3H), 1.90 (q, J = 7.1 Hz, 2H), 1.41 (s, 9H). ^{13}C NMR (101 MHz, CD_3OD) δ 159.08, 156.20, 136.55, 126.80, 113.36, 79.49, 70.97, 54.30, 45.81, 36.76, 33.45, 27.36. HRMS (EI): exact mass calculated for $C_{16}H_{25}NO_4$ $[M]^+$ 295.1784, found 295.1787.

tert-butyl (*S*)-(3-hydroxy-3-(4-(methylthio)phenyl)propyl)(methyl)carbamate (2s)

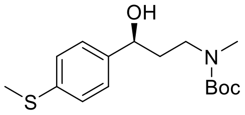

Colorless oil, 98% yield, 98% ee. $[\alpha]_D^{25}$ = 13.3 (c 2.0, CH_2Cl_2). The ee was determined by HPLC on Chiralpak AD-H column, hexane:isopropanol = 95:5; flow rate = 0.8 mL/min; UV detection at 220 nm; t_R = 29.183 min (major), t_R = 38.433 min (minor). ^{1}H NMR (400 MHz, CD_3OD) δ 7.32 – 7.23 (m, 4H), 4.57 (t, J = 6.6 Hz, 1H), 3.32 – 3.21 (m, 2H), 2.82 (s, 3H), 2.44 (s, 3H), 1.90 (d, J = 7.0 Hz, 2H), 1.42 (s, 9H). ^{13}C NMR (101 MHz, CD_3OD) δ 156.20, 141.58, 137.46, 126.32, 126.19, 79.52, 70.91, 45.72, 36.79, 33.44, 27.40, 14.57. HRMS (EI): exact mass calculated for $C_{16}H_{25}NO_3S$ $[M]^+$ 311.1555, found 311.1557.

tert-butyl (*S*)-(3-hydroxy-3-(4-phenoxyphenyl)propyl)(methyl)carbamate (2t)

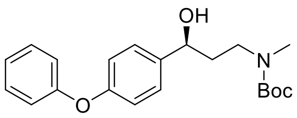

Colorless oil, 93% yield, 97% ee. $[\alpha]_D^{25}$ = 11.6 (c 2.0, CH_2Cl_2). The ee was determined by HPLC on Chiralpak AD-H column, hexane:isopropanol = 95:5; flow rate = 0.8 mL/min; UV detection at 220 nm; t_R = 22.759 min (major), t_R = 29.960 min (minor). ^{1}H NMR (400 MHz, CD_3OD) δ 7.33 (t, J = 8.1 Hz, 4H), 7.09 (t, J = 7.4 Hz, 1H), 6.95 (dd, J = 8.4, 3.5 Hz, 4H), 4.61

(t, *J* = 6.6 Hz, 1H), 3.34 – 3.22 (m, 2H), 2.83 (s, 3H), 1.93 (s, 2H), 1.43 (s, 9H). ^{13}C NMR (101 MHz, CD_3OD) δ 157.39, 156.50, 156.22, 139.67, 129.48, 127.14, 122.95, 118.38, 118.36, 79.53, 70.84, 45.75, 36.78, 33.42, 27.39. HRMS (EI): exact mass calculated for $C_{21}H_{27}NO_4$ $[M]^+$ 357.1940, found 357.1942.

tert-butyl (*S*)-(3-(4-(benzyloxy)phenyl)-3-hydroxypropyl)(methyl)carbamate (2u)

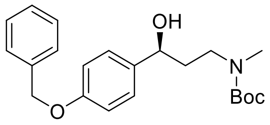

White solid, 96% yield, 98% ee. $[\alpha]_D^{25}$ = 11.3 (*c* 2.0, CH_2Cl_2). The ee was determined by HPLC on Chiralpak AD-H column, hexane:isopropanol = 95:5; flow rate = 0.8 mL/min; UV detection at 220 nm; t_R = 38.696 min (major), t_R = 49.877 min (minor). ^{1}H NMR (400 MHz, CD_3OD) δ 7.40 (d, *J* = 7.1 Hz, 2H), 7.33 (t, *J* = 7.4 Hz, 2H), 7.27 (dd, J = 10.2, 7.7 Hz, 3H), 6.95 (d, *J* = 8.4 Hz, 2H), 5.03 (s, 2H), 4.54 (t, *J* = 6.6 Hz, 1H), 3.35 – 3.17 (m, 2H), 2.80 (s, 3H), 1.90 (q, *J* = 7.1 Hz, 2H), 1.40 (s, 9H). ^{13}C NMR (101 MHz, CD_3OD) δ 158.15, 156.21, 137.37, 136.90, 128.11, 127.46, 127.16, 126.85, 114.45, 79.50, 70.96, 69.59, 45.80, 36.73, 33.46, 27.39. HRMS (EI): exact mass calculated for $C_{22}H_{29}NO_4$ $[M]^+$ 371.2097, found 371.2095.

tert-butyl (*S*)-(3-hydroxy-3-(o-tolyl)propyl)(methyl)carbamate (2v)

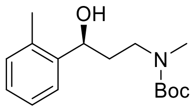

Colorless oil, 88% yield, 87% ee. $[\alpha]_D^{25}$ = -13.1 (*c* 2.0, CH_2Cl_2). The ee was determined by HPLC on Chiralpak OD-H column, hexane:isopropanol = 95:5; flow rate = 0.8 mL/min; UV detection at 220 nm; t_R = 10.068 min (minor), t_R = 10.803 min (major). ^{1}H NMR (400 MHz, CD_3OD) δ 7.45 (d, *J* = 7.6 Hz, 1H), 7.18 (dd, *J* = 8.3, 4.7 Hz, 1H), 7.11 (d, *J* = 4.4 Hz, 2H), 4.93 – 4.87 (m, 1H), 3.52 – 3.27 (m, 2H), 2.87 (s, 3H), 2.31 (s, 3H), 1.98 – 1.71 (m, 2H), 1.41 (s, 9H). ^{13}C NMR (101 MHz, CD_3OD) δ 156.28, 142.64, 133.86, 129.89, 126.61, 125.77, 124.80, 79.53, 67.32, 45.98, 35.94, 33.55, 27.32, 17.72. HRMS (EI): exact mass calculated for $C_{16}H_{25}NO_3$ $[M]^+$ 279.1834, found 279.1837.

tert-butyl (*S*)-(3-([1,1'-biphenyl]-4-yl)-3-hydroxypropyl)(methyl)carbamate (2w)

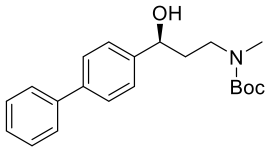

White solid, 98% yield, 98% ee. $[\alpha]_D^{25}$ = 22.1 (*c* 2.0, CH_2Cl_2). The ee was determined by HPLC on Chiralpak AD-H column, hexane:isopropanol = 95:5; flow rate = 0.8 mL/min; UV detection

at 220 nm; t_R = 22.171 min (major), t_R = 33.850 min (minor). ^{1}H NMR (400 MHz, CD_3OD) δ 7.58 (d, *J* = 7.5 Hz, 4H), 7.46 – 7.36 (m, 4H), 7.30 (t, *J* = 7.4 Hz, 1H), 4.66 (t, *J* = 6.5 Hz, 1H), 3.45 – 3.23 (m, 3H), 2.84 (s, 3H), 1.96 (q, *J* = 7.6, 6.7 Hz, 2H), 1.40 (s, 9H). ^{13}C NMR (101 MHz, CD_3OD) δ 156.22, 143.74, 140.77, 140.11, 128.48, 126.90, 126.60, 126.53, 126.09, 79.52, 71.04, 45.71, 36.88, 33.42, 27.37. HRMS (EI): exact mass calculated for $C_{21}H_{27}NO_3$ $[M]^+$ 341.1991, found 341.1993.

tert-butyl (*S*)-(3-(3,4-dichlorophenyl)-3-hydroxypropyl)(methyl)carbamate (2x)

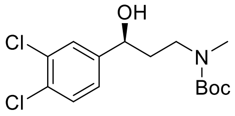

Colorless oil, 83% yield, 99% ee. $[\alpha]_D^{25}$ = 6.6 (*c* 2.0, CH_2Cl_2). The ee was determined by HPLC on Chiralpak AD-H column, hexane:isopropanol = 95:5; flow rate = 0.8 mL/min; UV detection at 220 nm; t_R = 13.207 min (major), t_R = 15.545 min (minor). ^{1}H NMR (400 MHz, CD_3OD) δ 7.52 (d, *J* = 2.0 Hz, 1H), 7.46 (d, *J* = 8.3 Hz, 1H), 7.27 (dd, *J* = 8.3, 2.0 Hz, 1H), 4.62 (dd, *J* = 7.7, 5.2 Hz, 1H), 3.35 – 3.21 (m, 2H), 2.84 (s, 3H), 1.89 (ddd, *J* = 18.1, 11.5, 7.7 Hz, 2H), 1.42 (s, 9H). ^{13}C NMR (101 MHz, CD_3OD) δ 156.20, 145.92, 131.84, 130.38, 130.09, 127.63, 125.39, 79.58, 69.93, 45.40, 36.73, 33.60, 27.38. HRMS (EI): exact mass calculated for $C_{15}H_{21}Cl_2NO_3$ $[M]^+$ 333.0898, found 333.0896.

tert-butyl (*S*)-(3-(3,4-dimethoxyphenyl)-3-hydroxypropyl) (methyl)carbamate (2y)

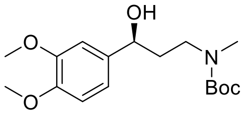

Colorless oil, 86% yield, 98% ee. $[\alpha]_D^{25}$ = 3.4 (*c* 2.0, CH_2Cl_2). The ee was determined by HPLC on Chiralpak AD-H column, hexane:isopropanol = 95:5; flow rate = 0.8 mL/min; UV detection at 220 nm; t_R = 46.050 min (major), t_R = 66.116 min (minor). ^{1}H NMR (400 MHz, CD_3OD) δ 6.98 (s, 1H), 6.93 – 6.84 (m, 2H), 4.56 (t, *J* = 6.6 Hz, 1H), 3.83 (s, 3H), 3.81 (s, 3H), 3.29 – 3.18 (m, 2H), 2.84 (s, 3H), 1.91 (q, *J* = 7.3 Hz, 2H), 1.42 (s, 9H). ^{13}C NMR (101 MHz, CD_3OD) δ 156.22, 149.10, 148.40, 137.55, 118.12, 111.44, 109.51, 79.48, 71.18, 55.15, 55.03, 45.73, 36.83, 33.43, 27.33. HRMS (EI): exact mass calculated for $C_{17}H_{27}NO_5$ $[M]^+$ 325.1889, found 325.1891.

tert-butyl (*S*)-(3-(3,5-dimethylphenyl)-3-hydroxypropyl)(methyl)carbamate (2z)

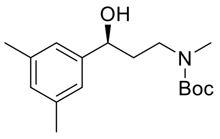

Colorless oil, 96% yield, 99% ee. $[\alpha]_D^{25}$ = 6.8 (*c* 2.0, CH_2Cl_2). The ee was determined by HPLC on Chiralpak AD-H column, hexane:isopropanol = 95:5; flow rate = 0.8 mL/min; UV detection at 220 nm; t_R = 12.585 min (major), t_R = 15.928 min (minor). ^{1}H NMR (400 MHz, CD_3OD) δ 6.94 (s, 2H), 6.87 (s, 1H), 4.52 (t, *J* = 6.6 Hz, 1H), 3.40 – 3.11 (m, 2H), 2.82 (s, 3H), 2.28 (s, 6H), 1.89 (s, 2H), 1.40 (s, 9H). ^{13}C NMR (101 MHz, CD_3OD) δ 156.19, 144.52, 137.47, 128.45, 123.37, 79.44, 71.38, 45.81, 36.78, 33.47, 27.43, 20.22. HRMS (EI): exact mass calculated for $C_{17}H_{27}NO_3$ $[M]^+$ 293.1991, found 293.1993.

tert-butyl (*S*)-(3-(benzo[d][1,3]dioxol-5-yl)-3-hydroxypropyl)(methyl)carbamate (2aa)

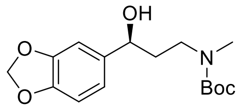

Colorless oil, 89% yield, 99% ee. $[\alpha]_D^{25}$ = 27.9 (*c* 2.0, CH_2Cl_2). The ee was determined by HPLC on Chiralpak AD-H column, hexane:isopropanol = 95:5; flow rate = 0.8 mL/min; UV detection at 220 nm; t_R = 34.228 min (major), t_R = 37.633 min (minor). ^{1}H NMR (400 MHz, CD_3OD) δ 6.86 (d, *J* = 1.5 Hz, 1H), 6.82 – 6.73 (m, 1H), 5.91 (q, *J* = 1.2 Hz, 2H), 4.52 (t, *J* = 6.6 Hz, 1H), 3.24 (s, 2H), 2.83 (s, 3H), 1.88 (q, *J* = 7.2 Hz, 2H), 1.42 (s, 9H). ^{13}C NMR (101 MHz, CD_3OD) δ 156.20, 147.78, 146.84, 138.70, 118.96, 107.47, 105.91, 100.86, 79.51, 71.20, 45.72, 36.72, 33.42, 27.34. HRMS (EI): exact mass calculated for $C_{16}H_{23}NO_5$ $[M]^+$ 309.1576, found 309.1578.

tert-butyl (*S*)-(3-hydroxy-3-(naphthalen-1-yl)propyl)(methyl)carbamate (2ab)

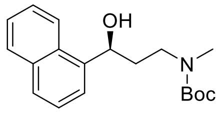

Colorless oil, 93% yield, 96% ee. $[\alpha]_D^{25}$ = -20.2 (*c* 2.0, CH_2Cl_2). The ee was determined by HPLC on Chiralpak AD-H column, hexane:isopropanol = 95:5; flow rate = 0.8 mL/min; UV detection at 220 nm; t_R = 22.602 min (major), t_R = 32.398 min (minor). ^{1}H NMR (400 MHz, CD_3OD) δ 8.05 (d, *J* = 9.0 Hz, 1H), 7.86 (dd, *J* = 7.1, 2.2 Hz, 1H), 7.76 (d, *J* = 8.2 Hz, 1H), 7.68 (d, *J* = 7.1 Hz, 1H), 7.53 – 7.36 (m, 3H), 5.58 – 5.39 (m, 1H), 3.63 – 3.29 (m, 2H), 2.88 (s, 3H), 2.29 – 2.10 (m, 1H), 1.93 (s, 1H), 1.39 (9H). ^{13}C NMR (101 MHz, CD_3OD) δ 157.66, 141.74, 135.34, 131.60, 129.97, 128.71, 126.95, 126.49, 124.01, 123.73, 123.72, 80.89, 69.07, 47.56, 37.77, 35.14, 28.74. HRMS (EI): exact mass calculated for $C_{19}H_{25}NO_3$ $[M]^+$ 315.1834, found 315.1837.

tert-butyl (*S*)-(3-hydroxy-3-(naphthalen-2-yl)propyl)(methyl)carbamate (2ac)

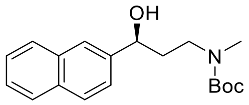

Colorless oil, 96% yield, 98% ee. $[\alpha]_D^{25}$ = 9.4 (*c* 2.0, CH_2Cl_2). The ee was determined by HPLC on Chiralpak AD-H column, hexane:isopropanol = 95:5; flow rate = 0.8 mL/min; UV detection at 220 nm; t_R = 33.363 min (major), t_R = 38.623 min (minor). ^{1}H NMR (400 MHz, CD_3OD) δ 7.87 – 7.72 (m, 4H), 7.53 – 7.38 (m, 3H), 4.78 (t, *J* = 6.6 Hz, 1H), 3.35 – 3.22 (m, 2H), 2.82 (t, *J* = 4.0 Hz, 3H), 2.02 (q, *J* = 6.8 Hz, 2H), 1.39 (9H).^{13}C NMR (101 MHz, CD_3OD) δ 156.22, 142.05, 133.42, 133.02, 127.80, 127.57, 127.28, 125.73, 125.39, 124.17, 123.84, 79.51, 71.43, 45.72, 36.69, 33.40, 27.36. HRMS (EI): exact mass calculated for $C_{19}H_{25}NO_3$ $[M]^+$ 315.1834, found 315.1836.

tert-butyl (*S*)-(3-(9H-fluoren-2-yl)-3-hydroxypropyl)(methyl)carbamate (2ad)

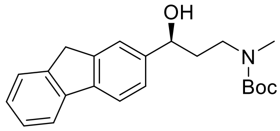

Colorless oil, 96% yield, 97% ee. $[\alpha]_D^{25}$ = 20.4 (*c* 2.0, CH_2Cl_2). The ee was determined by HPLC on Chiralpak AD-H column, hexane:isopropanol = 95:5; flow rate = 0.8 mL/min; UV detection at 220 nm; t_R = 16.481 min (major), t_R = 23.707 min (minor). ^{1}H NMR (400 MHz, CD_3OD) δ 7.72 (dd, *J* = 7.8, 4.5 Hz, 2H), 7.51 (s, 1H), 7.47 (d, *J* = 7.4 Hz, 1H), 7.31 (q, *J* = 7.8, 7.4 Hz, 2H), 7.23 (t, *J* = 7.4 Hz, 1H), 4.67 (t, *J* = 6.6 Hz, 1H), 3.79 (s, 2H), 3.45 – 3.17 (m, 2H), 2.82 (s, 3H), 1.96 (d, *J* = 8.3 Hz, 2H), 1.38 (s, 9H). ^{13}C NMR (101 MHz, CD_3OD) δ 156.19, 143.43, 143.34, 143.24, 141.33, 140.86, 126.45, 126.32, 124.68, 124.43, 122.26, 119.39, 119.30, 79.49, 71.56, 45.84, 36.91, 36.27, 33.49, 27.42. HRMS (EI): exact mass calculated for $C_{22}H_{27}NO_3$ $[M]^+$ 353.1991, found 353.1993.

tert-butyl (*S*)-ethyl(3-hydroxy-3-(thiophen-2-yl)propyl)carbamate (2ae)

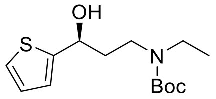

Colorless oil, 98% yield, >99% ee. $[\alpha]_D^{25}$ = 29.3 (*c* 2.0, CH_2Cl_2). The ee was determined by HPLC on Chiralpak AD-H column, hexane:isopropanol = 95:5; flow rate = 0.8 mL/min; UV detection at 220 nm; t_R = 15.094 min (major), t_R = 17.805 min (minor). ^{1}H NMR (400 MHz, CD_3OD) δ 7.29 (dd, *J* = 5.0, 1.3 Hz, 1H), 6.99 – 6.96 (m, 1H), 6.95 (dd, *J* = 5.0, 3.5 Hz, 1H), 4.88 (t, *J* = 6.6 Hz, 1H), 3.35 – 3.15 (m, 4H), 2.10 – 1.95 (m, 1H), 1.44 (s, 9H), 1.09 (*t*, J = 7.1 Hz, 3H). ^{13}C NMR (101 MHz, CD_3OD) δ 155.88, 148.70, 126.13, 123.85, 123.18, 79.49, 67.17, 43.61, 41.94, 38.10, 27.40, 12.62. HRMS (EI): exact mass calculated for $C_{14}H_{23}NO_3S$ $[M]^+$ 285.1399, found 285.1396.

tert-butyl (*S*)-(3-hydroxy-3-(thiophen-2-yl)propyl)(isopropyl)carbamate (2af)

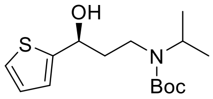

Colorless oil, 98% yield, >99% ee. $[\alpha]_D^{25}$ = 23.3 (*c* 2.0, CH_2Cl_2). The ee was determined by HPLC on Chiralpak AD-H column, hexane:isopropanol = 95:5; flow rate = 0.8 mL/min; UV detection at 220 nm; t_R = 14.343 min (major), t_R = 17.261 min (minor). ^{1}H NMR (400 MHz, CD_3OD) δ 7.29 (dd, *J* = 5.0, 1.3 Hz, 1H), 7.20 – 6.71 (m, 2H), 4.88 (t, *J* = 6.6 Hz, 1H), 4.30 – 3.93 (m, 1H), 3.28 – 2.98 (m, 2H), 2.04 (q, *J* = 7.0 Hz, 2H), 1.45 (s, 9H), 1.12 (d, *J* = 7.0 Hz, 6H). ^{13}C NMR (101 MHz, CD_3OD) δ 155.82, 148.72, 126.12, 123.83, 123.13, 79.53, 67.71, 39.35, 27.49, 19.62. Carbon atoms not found in the carbon spectrum were identified by HMQC. HRMS (EI): exact mass calculated for $C_{15}H_{25}NO_3S$ $[M]^+$ 299.1555, found 299.1553.

tert-butyl (*S*)-(3-hydroxy-3-(thiophen-2-yl)propyl)(neopentyl)carbamate (2ag)

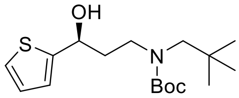

White solid, 98% yield, 98% ee. $[\alpha]_D^{25}$ = 10.9 (*c* 2.0, CH_2Cl_2). The ee was determined by HPLC on Chiralpak AD-H column, hexane:isopropanol = 95:5; flow rate = 0.8 mL/min; UV detection at 220 nm; t_R = 11.634 min (major), t_R = 13.542 min (minor). ^{1}H NMR (400 MHz, CD_3OD) δ 7.28 (s, 1H), 7.14 – 6.90 (m, 2H), 4.84 (t, *J* = 7.5 Hz, 1H), 3.39 – 3.24 (m, 2H), 3.05 (s, 2H), 2.10 – 1.92 (m, 2H), 1.44 (s, 9H), 0.89 (s, 9H). ^{13}C NMR (101 MHz, CD_3OD) δ 156.61, 148.76, 126.14, 123.85, 123.16, 79.53, 67.21, 57.83, 46.39, 37.14, 33.44, 27.40, 27.28. HRMS (EI): exact mass calculated for $C_{17}H_{29}NO_3S$ $[M]^+$ 327.1868, found 327.1866.

tert-butyl ((1*S*,3*S*,5*S*,7*S*)-adamantan-2-yl)((*S*)-3-hydroxy-3-(thiophen-2-yl)propyl)carbamate (2ah)

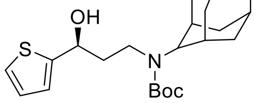

White solid, 99% yield, >99% ee. $[\alpha]_D^{25}$ = 14.7 (c 2.0, CH_2Cl_2). The ee was determined by HPLC on Chiralpak AD-H column, hexane:isopropanol = 95:5; flow rate = 0.8 mL/min; UV detection at 220 nm; t_R = 15.130 min (major), t_R = 17.228 min (minor). ^{1}H NMR (400 MHz, CD_3OD) δ 7.27 (dd, *J* = 4.9, 1.3 Hz, 1H), 6.98 – 6.95 (m, 1H), 6.94 (dd, *J* = 5.0, 3.5 Hz, 1H), 4.88 (t, *J* = 6.5 Hz, 1H), 3.83 (d, *J* = 2.5 Hz, 1H), 3.58 – 3.44 (m, 1H), 3.43 – 3.30 (m, 1H), 2.22 – 2.02 (m, 4H), 1.98 – 1.78 (m, 8H), 1.74 (s, 2H), 1.63 – 1.54 (m, 2H), 1.44 (s, 9H). ^{13}C NMR (101 MHz, CD_3OD) δ 156.83, 148.75, 126.11, 123.81, 123.10, 79.49, 67.91, 60.86, 40.83,

39.77, 38.45, 38.35, 37.55, 31.94, 31.90, 31.52, 31.48, 27.68, 27.51, 27.09. HRMS (EI): exact mass calculated for $C_{22}H_{33}NO_3S$ $[M]^+$ 391.2181, found 391.2183.

tert-butyl (*S*)-benzyl(3-hydroxy-3-(thiophen-2-yl)propyl)carbamate (2ai)

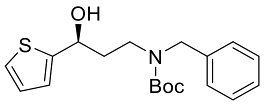

Colorless oil, 98% yield, >99% ee. $[\alpha]_D^{25}$ = -2.8 (*c* 2.0, CH_2Cl_2). The ee was determined by HPLC on Chiralpak AD-H column, hexane:isopropanol = 95:5; flow rate = 0.8 mL/min; UV detection at 220 nm; t_R = 29.671 min (major), t_R = 33.673 min (minor). ^{1}H NMR (400 MHz, CD_3OD) δ 7.34 – 7.09 (m, 6H), 6.92 (d, *J* = 4.3 Hz, 1H), 4.83 (t, *J* = 6.5 Hz, 1H), 4.42 (s, 2H), 3.43 – 3.14 (m, 2H), 2.00 (qd, *J* = 7.1, 2.7 Hz, 2H), 1.45 (s, 9H). ^{13}C NMR (101 MHz, CD_3OD) δ 156.24, 148.62, 138.26, 128.21, 127.22, 126.95, 126.14, 123.88, 123.21, 79.98, 67.17, 50.28, 43.72, 37.46, 27.40. HRMS (EI): exact mass calculated for $C_{19}H_{25}NO_3S$ $[M]^+$ 347.1555, found 347.1552.

tert-butyl (*S*)-(4-fluorobenzyl)(3-hydroxy-3-(thiophen-2-yl)propyl)carbamate (2aj)

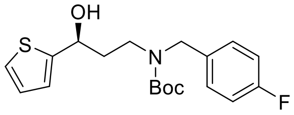

Colorless oil, 99% yield, >99% ee. $[\alpha]_D^{25}$ = -1.7 (*c* 2.0, CH_2Cl_2). The ee was determined by HPLC on Chiralpak AD-H column, hexane:isopropanol = 95:5; flow rate = 0.8 mL/min; UV detection at 220 nm; t_R = 29.729 min (major), t_R = 33.485 min (minor). ^{1}H NMR (400 MHz, CD_3OD) δ 7.30 – 7.26 (m, 1H) 7.22 (dd, *J* = 8.4, 5.4 Hz, 2H), 7.03 (t, *J* = 8.6 Hz, 2H), 6.93 (d, *J* = 3.5 Hz, 2H), 4.84 (t, *J* = 6.8 Hz, 1H), 4.40 (s, 2H), 3.43 – 3.13 (m, 2H), 2.09 – 1.89 (m, 2H), 1.45 (s, 9H). ^{13}C NMR (101 MHz, CD_3OD) δ 162.10 (d, *J* = 244.0 Hz), 156.13, 148.63, 134.33, 129.10, 126.16, 123.90, 123.22, 114.87 (d, *J* = 21.6 Hz), 80.06, 67.16, 49.51, 43.65, 37.63, 27.40. HRMS (EI): exact mass calculated for $C_{19}H_{24}FNO_3S$ $[M]^+$ 365.1461, found 365.1463.

tert-butyl (*S*)-(4-chlorobenzyl)(3-hydroxy-3-(thiophen-2-yl)propyl)carbamate (2ak)

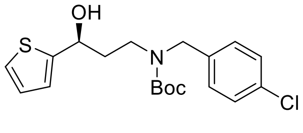

Colorless oil, 95% yield, 99% ee. $[\alpha]_D^{25}$ = -2.2 (*c* 2.0, CH_2Cl_2). The ee was determined by HPLC on Chiralpak OD-H column, hexane:isopropanol = 95:5; flow rate = 0.8 mL/min; UV detection at 220 nm; t_R = 23.317 min (minor), t_R = 25.069 min (major). ^{1}H NMR (400 MHz, CD_3OD) δ 7.38 – 7.23 (m, 3H), 7.18 (d, *J* = 8.1 Hz, 2H), 6.93 (d, *J* = 3.5 Hz, 2H), 4.84 (d, *J* = 6.4 Hz, 1H), 4.39 (s, 2H), 3.46 – 3.12 (m, 2H), 2.03 (s, 2H), 1.46 (s, 9H). ^{13}C NMR (101 MHz, CD_3OD) δ

156.11, 148.64, 137.22, 132.66, 128.73, 128.32, 126.21, 123.94, 123.25, 80.14, 67.15, 49.70, 43.78, 37.48, 27.45. HRMS (EI): exact mass calculated for $C_{19}H_{24}ClNO_3S$ $[M]^+$ 381.1165, found 381.1162.

tert-butyl (*S*)-(3-hydroxy-3-(thiophen-2-yl)propyl)(4-methoxybenzyl)carbamate (2al)

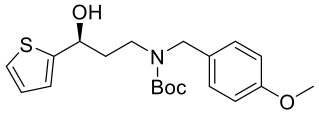

Colorless oil, 98% yield, >99% ee. $[\alpha]_D^{25}$ = -2.9 (*c* 2.0, CH_2Cl_2). The ee was determined by HPLC on Chiralpak AD-H column, hexane:isopropanol = 95:5; flow rate = 0.8 mL/min; UV detection at 220 nm; t_R = 39.080 min (major), t_R = 43.789 min (minor). ^{1}H NMR (400 MHz, CD_3OD) δ 7.33 – 7.18 (m, 1H), 7.13 (d, *J* = 8.4 Hz, 2H), 6.92 (d, *J* = 4.8 Hz, 2H), 6.85 (d, *J* = 8.2 Hz, 2H), 4.82 (t, *J* = 6.6 Hz, 1H), 4.35 (s, 2H), 3.75 (s, 3H), 3.22 (s, 2H), 1.98 (q, *J* = 6.7, 6.0 Hz, 2H), 1.46 (s, 9H). ^{13}C NMR (101 MHz, CD_3OD) δ 159.08, 156.21, 148.61, 130.16, 128.61, 126.12, 123.87, 123.22, 113.58, 79.90, 67.20, 54.32, 49.61, 43.35, 37.41, 27.41. HRMS (EI): exact mass calculated for $C_{20}H_{27}NO_4$ $[M]^+$ 377.1661, found 377.1663.

tert-butyl ((*S*)-3-hydroxy-3-(thiophen-2-yl)propyl)((R)-1-phenylethyl)carbamate (2am)

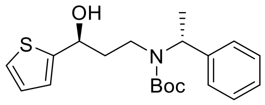

Colorless oil, 99% yield, >99:1 dr. $[\alpha]_D^{25}$ = 48.5 (*c* 2.0, CH_2Cl_2). The dr was determined by HPLC on Chiralpak AD-H column, hexane:isopropanol = 95:5; flow rate = 0.8 mL/min; UV detection at 220 nm; t_R = 11.177 min (major), t_R = 13.285 min (minor). ^{1}H NMR (400 MHz, CD_3OD) δ 7.46 – 7.16 (m, 6H), 6.91 (dd, *J* = 5.1, 3.5 Hz, 1H), 6.85 (d, *J* = 3.6 Hz, 1H), 5.38 (br, 1H), 4.72 (s, 1H), 3.12 (s, 2H), 2.06 – 1.79 (m, 2H), 1.52 (d, *J* = 7.2 Hz, 3H), 1.46 (s, 9H). ^{13}C NMR (101 MHz, CD_3OD) δ 156.14, 148.72, 141.56, 128.19, 127.01, 126.76, 126.15, 123.79, 122.94, 79.93, 67.87, 53.21, 40.80, 39.37, 27.57, 16.60. HRMS (EI): exact mass calculated for $C_{20}H_{27}NO_3S$ $[M]^+$ 361.1712, found 361.1715.

tert-butyl (*S*)-benzhydryl(3-hydroxy-3-(thiophen-2-yl)propyl)carbamate (2an)

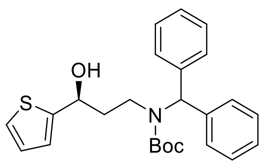

Colorless oil, 99% yield, >99% ee. $[\alpha]_D^{25}$ = -41.9 (*c* 2.0, CH_2Cl_2). The ee was determined by HPLC on Chiralpak AD-H column, hexane:isopropanol = 95:5; flow rate = 0.8 mL/min; UV detection at 220 nm; t_R = 27.181 min (major), t_R = 71.013 min (minor). ^{1}H NMR (400 MHz,

CD_3OD) δ 7.40 – 7.26 (m, 6H), 7.22 – 7.07 (m, 5H), 6.84 (d, *J* = 3.5 Hz, 1H), 6.68 (d, *J* = 3.2 Hz, 1H), 6.47 (br, 1H), 4.53 (t, *J* = 6.6 Hz, 1H), 3.40 (ddd, *J* = 14.9, 10.5, 4.8 Hz, 1H), 3.31 – 3.17 (m, 1H), 1.70 – 1.56 (m, 2H), 1.41 (s, 9H). ^{13}C NMR (101 MHz, CD_3OD) δ 156.26, 148.38, 139.99, 128.40, 128.26, 127.27, 126.05, 123.74, 122.88, 80.34, 67.77, 63.55, 42.66, 38.22, 27.47. HRMS (EI): exact mass calculated for $C_{25}H_{29}NO_3S$ $[M]^+$ 423.1868, found 423.1869.

tert-butyl (*S*)-(3-hydroxy-3-phenylpropyl)(isopropyl)carbamate (2ao)

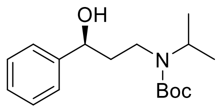

Colorless oil, 95% yield, 99% ee. $[\alpha]_D^{25}$ = -7.2 (*c* 2.0, CH_2Cl_2). The ee was determined by HPLC on Chiralpak AD-H column, hexane:isopropanol = 95:5; flow rate = 0.8 mL/min; UV detection at 220 nm; t_R = 14.102 min (major), t_R = 20.927 min (minor). ^1H NMR (400 MHz, CD_3OD) δ 7.41 – 7.28 (m, 4H), 7.24 (t, *J* = 6.6 Hz, 1H), 4.64 (t, *J* = 6.5 Hz, 1H), 3.13 (d, *J* = 45.2 Hz, 1H), 1.96 (d, *J* = 7.3 Hz, 2H), 1.44 (s, 9H), 1.11 (d, *J* = 4.0 Hz, 6H). ^{13}C NMR (101 MHz, CD_3OD) δ 155.83, 144.68, 127.99, 126.94, 125.52, 79.44, 71.91, 39.34, 27.47, 19.60. The position of carbon atoms in ^{13}C NMR spectrum is assigned by analogy to **2af**. HRMS (EI): exact mass calculated for $C_{17}H_{27}NO_3$ $[M]^+$ 293.1991, found 293.1989.

tert-butyl (*S*)-benzyl(3-hydroxy-3-phenylpropyl) carbamate (2ap)

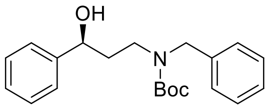

Colorless oil, 99% yield, 98% ee. $[\alpha]_D^{25}$ = -39.8 (*c* 2.0, CH_2Cl_2). The ee was determined by HPLC on Chiralpak AD-H column, hexane:isopropanol = 95:5; flow rate = 0.8 mL/min; UV detection at 220 nm; t_R = 30.031 min (major), t_R = 36.741 min (minor). ^1H NMR (400 MHz, CD_3OD) δ 7.33 – 7.24 (m, 6H), 7.27 – 7.15 (m, 4H), 4.56 (d, *J* = 6.6 Hz, 1H), 4.40 (s, 2H), 3.42 – 3.07 (m, 2H), 1.91 (q, *J* = 7.0 Hz, 2H), 1.43 (s, 9H). ^{13}C NMR (101 MHz, CD_3OD) δ 156.26, 144.59, 138.32, 128.20, 127.99, 127.23, 126.95, 126.92, 125.53, 79.91, 71.36, 50.24, 43.70, 37.15, 27.39. HRMS (EI): exact mass calculated for $C_{21}H_{27}NO_3$ $[M]^+$ 341.1991, found 341.1993.

isopropyl (*S*)-(3-hydroxy-3-(thiophen-2-yl)propyl)(methyl)carbamate (4a)

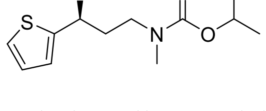

Colorless oil, 98% yield, 98% ee. $[\alpha]_D^{25}$ = -3.2 (*c* 2.0, CH_2Cl_2). The ee was determined by HPLC on Chiralpak AD-H column, hexane:isopropanol = 95:5; flow rate = 0.8 mL/min; UV detection at 220 nm; t_R = 21.212 min (major), t_R = 22.543 min (minor). ^1H NMR (400 MHz, CD_3OD) δ

7.28 (d, *J* = 5.0 Hz, 1H), 6.98 (dt, *J* = 3.6, 1.0 Hz, 1H), 6.94 (dd, *J* = 5.1, 3.5 Hz, 1H), 4.87 (t, *J* = 6.7 Hz, 1H), 4.85 – 4.79 (m, 2H), 3.41 – 3.29 (m, 2H), 2.87 (s, 3H), 2.19 – 1.92 (m, 2H), 1.22 (d, *J* = 6.4 Hz, 6H). ^{13}C NMR (101 MHz, CD_3OD) ^{13}C NMR (101 MHz, CD_3OD) δ 156.54, 144.68, 128.01, 126.97, 125.56, 71.30, 68.72, 45.65, 36.70, 33.43, 21.14. HRMS (EI): exact mass calculated for $C_{12}H_{19}NO_3S$ $[M]^+$ 257.1086, found 257.1089.

isobutyl (*S*)-(3-hydroxy-3-(thiophen-2-yl)propyl)(methyl)carbamate (4b)

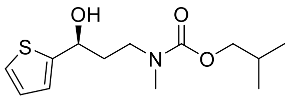

Colorless oil, 87% yield, 97% ee. $[\alpha]_D^{25}$ = 10.5 (*c* 1.0, CH_2Cl_2). The ee was determined by HPLC on Chiralpak AD-H column, hexane:isopropanol = 95:5; flow rate = 0.8 mL/min; UV detection at 220 nm; t_R = 24.131 min (major), t_R = 25.633 min (minor). ^{1}H NMR (400 MHz, CD_3OD) δ 7.28 (d, *J* = 4.9 Hz, 1H), 6.96 (dd, *J* = 12.8, 4.0 Hz, 2H), 4.87 (s, 2H), 3.82 (d, *J* = 6.5 Hz, 2H), 3.37 (d, *J* = 7.4 Hz, 2H), 2.89 (s, 2H), 2.04 (d, *J* = 7.2 Hz, 2H), 1.97 – 1.79 (m, 1H), 0.93 (s, 4H). ^{13}C NMR (101 MHz, CD_3OD) δ 156.94, 148.71, 126.15, 123.91, 123.22, 45.69, 37.03, 33.60, 27.90, 18.11. HRMS (EI): exact mass calculated for $C_{13}H_{21}NO_3S$ $[M]^+$ 271.1242, found 271.1244.

isopropyl (*S*)-(3-hydroxy-3-(thiophen-2-yl)propyl)(methyl)carbamate (4c)

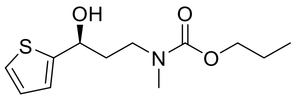

Colorless oil, 86% yield, 96% ee. $[\alpha]_D^{25}$ = 11.7 (*c* 1.0, CH_2Cl_2). The ee was determined by HPLC on Chiralpak AD-H column, hexane:isopropanol = 95:5; flow rate = 0.8 mL/min; UV detection at 220 nm; t_R = 27.238 min (major), t_R = 29.127 min (minor). ^{1}H NMR (400 MHz, CD_3OD) δ 7.34 – 7.18 (m, 1H), 7.03 – 6.92 (m, 2H), 4.89 (t, *J* = 6.6 Hz, 1H), 4.00 (t, *J* = 6.6 Hz, 2H), 3.38 (t, *J* = 7.4 Hz, 2H), 2.90 (s, 3H), 2.53 – 1.88 (m, 2H), 1.65 (q, *J* = 7.2 Hz, 2H), 0.95 (t, *J* = 7.5 Hz, 3H). ^{13}C NMR (101 MHz, CD_3OD) δ 157.02, 148.70, 126.13, 123.90, 123.23, 67.10, 66.91, 45.65, 36.99, 33.56, 22.06, 9.42. HRMS (EI): exact mass calculated for $C_{12}H_{19}NO_3S$ $[M]^+$ 257.1086, found 257.1089.

(*S*)-3-(dimethylamino)-1-(thiophen-2-yl)propan-1-ol (4d)

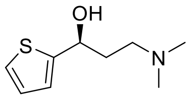

White solid, 96% yield, >99% ee. $[\alpha]_D^{25}$ = -7.4 (*c* 1.0, MeOH). The ee was determined by HPLC on Chiralpak AD-H column, hexane:isopropanol:Et_2NH= 95:5:0.2; flow rate = 0.8 mL/min; UV detection at 254 nm; t_R = 14.147 min (major), t_R = 16.392 min (minor). ^{1}H NMR (400 MHz,

$CDCl_3$) δ 7.19 (dd, *J* = 4.9, 1.4 Hz, 1H), 7.00 – 6.86 (m, 2H), 5.88 (br, 1H), 5.16 (dd, *J* = 7.5, 4.0 Hz, 1H), 2.77 – 2.51 (m, 2H), 2.31 (s, 6H), 2.02 – 1.90 (m, 2H). ^{13}C NMR (101 MHz, $CDCl_3$) δ 149.64, 126.66, 123.79, 122.40, 71.74, 57.89, 45.16, 34.58. HRMS (EI): exact mass calculated for $C_9H_{15}NOS$ $[M]^+$ 185.0874, found 185.0876.

(*S*)-3-(benzyl(methyl)amino)-1-(thiophen-2-yl)propan-1-ol (4e)

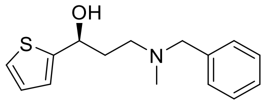

Colorless oil, 95% yield, 99% ee. $[\alpha]_D^{25}$ = -34.9 (*c* 1.0, CH_2Cl_2). The ee was determined by HPLC on Chiralpak AD-H column, hexane:isopropanol = 95:5; flow rate = 0.8 mL/min; UV detection at 220 nm; t_R = 12.571 min (major), t_R = 15.633 min (minor). ^1H NMR (400 MHz, $CDCl_3$) δ 7.40 – 7.26 (m, 5H), 7.21 (dd, *J* = 5.0, 1.2 Hz, 1H), 6.96 (dd, *J* = 5.0, 3.5 Hz, 1H), 6.93 – 6.84 (m, 1H), 5.22 – 5.12 (m, 1H), 3.65 (d, *J* = 12.8 Hz, 1H), 3.49 (d, *J* = 12.8 Hz, 1H), 2.83 (ddd, *J* = 12.9, 8.8, 4.3 Hz, 1H), 2.67 (ddd, *J* = 12.7, 5.6, 3.7 Hz, 1H), 2.26 (s, 3H), 2.12 – 1.92 (m, 2H). ^{13}C NMR (151 MHz, $CDCl_3$) δ 148.60, 136.62, 128.30, 127.59, 126.53, 125.65, 122.76, 121.35, 71.23, 61.89, 55.26, 40.70, 33.63. HRMS (EI): exact mass calculated for $C_{15}H_{19}NOS$ $[M]^+$ 261.1187, found 261.1185.

(*S*)-3-(methylamino)-1-(thiophen-2-yl)propan-1-ol (4f)

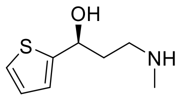

White solid, 90% yield, >99% ee. $[\alpha]_D^{25}$ = -13.0 (*c* 1.0, MeOH). The ee was determined by the corresponding acetamide **4f**-NAc on HPLC using Chiralpak OD-H column, hexane:isopropanol = 90:10; flow rate = 0.8 mL/min; UV detection at 220 nm; t_R = 25.953 min (major), t_R = 31.023 min (minor). ^1H NMR (400 MHz, $CDCl_3$) δ 7.20 (dd, *J* = 5.0, 1.2 Hz, 1H), 6.96 (dd, *J* = 5.0, 3.5 Hz, 1H), 6.92 (d, *J* = 3.4 Hz, 1H), 5.18 (dd, *J* = 8.4, 3.2 Hz, 1H), 3.76 (br, 2H), 3.14 – 2.76 (m, 2H), 2.43 (s, 3H), 2.10 – 1.78 (m, 2H). ^{13}C NMR (101 MHz, $CDCl_3$) δ δ 149.82, 126.66, 123.82, 122.45, 72.02, 50.26, 36.99, 36.07. HRMS (EI): exact mass calculated for $C_{17}H_{29}NO_3S$ $[M]^+$ 171.0718, found 171.0715.

tert-butyl (*R*)-(3-hydroxy-3-phenylpropyl)(methyl)carbamate (4g)

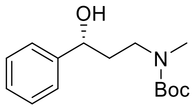

Colorless oil, 95% yield, 98% ee. $[\alpha]_D^{25}$ = -2.0 (*c* 2.0, CH_2Cl_2). The ee was determined by HPLC on Chiralpak AD-H column, hexane:isopropanol = 95:5; flow rate = 0.8 mL/min; UV detection at 220 nm; t_R = 15.501 min (minor), t_R = 17.873 min (major). ^1H NMR (400 MHz, CD_3OD) δ

7.39 – 7.29 (m, 4H), 7.27 – 7.19 (m, 1H), 4.61 (t, J = 6.6 Hz, 1H), 3.42 – 3.13 (m, 2H), 2.83 (s, 3H), 1.92 (q, J = 7.6, 7.0 Hz, 2H), 1.42 (s, 9H). ^{13}C NMR (101 MHz, CD_3OD) δ 156.23, 144.66, 127.99, 126.95, 125.53, 79.51, 71.33, 45.69, 36.85, 33.39, 27.34. HRMS (EI): exact mass calculated for $C_{15}H_{23}NO_3$ $[M]^+$ 265.1678, found 265.1679.

isopropyl (*R*)-(3-hydroxy-3-phenylpropyl)(methyl)carbamate (4h)

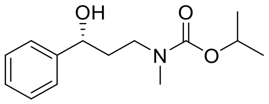

Colorless oil, 98% yield, 98% ee. $[\alpha]_D^{25}$ = 3.2 (c 1.0, CH_2Cl_2). The ee was determined by HPLC on Chiralpak AD-H column, hexane:isopropanol = 95:5; flow rate = 0.8 mL/min; UV detection at 220 nm; t_R = 19.561 min (minor), t_R = 22.378 min (major). ^{1}H NMR (400 MHz, CD_3OD) δ 7.39 – 7.29 (m, 4H), 7.29 – 7.20 (m, 1H), 4.85 – 4.79 (m, 1H), 4.62 (t, J = 6.6 Hz, 1H), 3.42 – 3.26 (m, 2H), 2.87 (s, 3H), 1.98 – 1.89 (m, 2H), 1.21 (d, J = 6.4 Hz, 6H). ^{13}C NMR (101 MHz, CD_3OD) δ 156.56, 144.63, 127.98, 126.96, 125.53, 71.32, 68.73, 45.62, 36.66, 33.37, 21.06. HRMS (EI): exact mass calculated for $C_{14}H_{21}NO_3$ $[M]^+$ 251.1521, found 251.1523.

(*R*)-3-(dimethylamino)-1-phenylpropan-1-ol (4i)

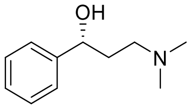

Colorless oil, 96% yield, 99% ee. $[\alpha]_D^{25}$ = 22.5 (c 1.0, CH_2Cl_2). The ee was determined by HPLC on Chiralpak AD-H column, hexane:isopropanol:Et_2NH= 95:5:0.2; flow rate = 0.8 mL/min; UV detection at 254 nm; t_R = 11.016 min (minor), t_R = 14.190 min (major). ^{1}H NMR (400 MHz, $CDCl_3$) δ 7.45 – 7.30 (m, 4H), 7.28 – 7.12 (m, 1H), 4.92 (dd, J = 7.7, 4.1 Hz, 1H), 2.64 (ddd, J = 12.9, 8.7, 4.4 Hz, 1H), 2.46 (ddd, J = 12.5, 5.5, 3.9 Hz, 1H), 2.29 (s, 6H), 1.98 – 1.61 (m, 2H). ^{13}C NMR (101 MHz, $CDCl_3$) δ 145.16, 128.25, 126.93, 125.64, 75.77, 58.45, 45.38, 34.63. HRMS (EI): exact mass calculated for $C_{17}H_{29}NO_3S$ $[M]^+$ 179.1310, found 179.1313.

(*R*)-3-(benzyl(methyl)amino)-1-phenylpropan-1-ol (4j)

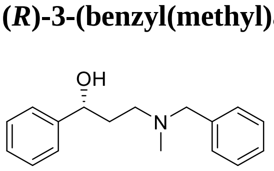

Colorless oil, 94% yield, 98% ee. $[\alpha]_D^{25}$ = 49.3 (c 1.0, CH_2Cl_2). The ee was determined by HPLC on Chiralpak OD-H column, hexane:isopropanol = 95:5; flow rate = 0.8 mL/min; UV detection at 220 nm; t_R = 13.910 min (minor), t_R = 16.157 min (major). ^{1}H NMR (400 MHz, $CDCl_3$) δ 7.45 – 7.26 (m, 9H), 7.24 – 7.18 (m, 1H), 4.89 (dd, J = 7.8, 3.8 Hz, 1H), 3.63 (d, J =

12.8 Hz, 1H), 3.46 (d, J = 12.8 Hz, 1H), 2.80 (ddd, J = 13.1, 9.1, 4.2 Hz, 1H), 2.57 (ddd, J = 12.7, 5.3, 3.6 Hz, 1H), 2.25 (s, 3H), 1.96 – 1.79 (m, 2H). ^{13}C NMR (101 MHz, $CDCl_3$) δ 145.01, 137.80, 129.29, 128.57, 128.23, 127.48, 126.91, 125.60, 75.79, 62.84, 56.54, 41.83, 34.56. HRMS (EI): exact mass calculated for $C_{17}H_{21}NO$ $[M]^+$ 255.1623, found 255.1626.

(*R*)-3-(methylamino)-1-phenylpropan-1-ol (4k)

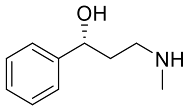

Colorless oil, 89% yield, >99% ee. $[\alpha]_D^{25}$ = 30.5 (c 1.0, CH_2Cl_2). The ee was determined by the corresponding acetamide **4k**-NAc on HPLC using Chiralpak OD-H column, hexane:isopropanol = 90:10; flow rate = 0.8 mL/min; UV detection at 220 nm; t_R = 23.381 min (minor), t_R = 29.864 min (major). ^{1}H NMR (400 MHz, $CDCl_3$) δ 7.39 – 7.29 (m, 4H), 7.27 – 7.19 (m, 1H), 4.90 (dd, J = 8.7, 3.2 Hz, 1H), 4.38 (br, 2H), 2.94 – 2.77 (m, 2H), 2.42 (s, 3H), 1.96 – 1.65 (m, 2H). ^{13}C NMR (101 MHz, $CDCl_3$) δ 145.08, 128.28, 127.02, 125.67, 75.19, 50.18, 36.80, 35.85. HRMS (EI): exact mass calculated for $C_{17}H_{29}NO_3S$ $[M]^+$ 165.1154, found 165.1157.

(*S*)-3-(dimethylamino)-1-phenylpropan-1-ol (6a)

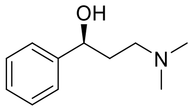

White solid, 97% yield, >99% ee. $[\alpha]_D^{25}$ = -27.0 (c 1.0, CH_2Cl_2). The ee was determined by HPLC on Chiralpak AD-H column, hexane:isopropanol:Et_2NH= 95:5:0.2; flow rate = 0.8 mL/min; UV detection at 254 nm; t_R = 10.379 min (major), t_R = 14.019 min (minor). ^{1}H NMR (400 MHz, $CDCl_3$) δ 7.42 – 7.31 (m, 4H), 7.28 – 7.21 (m, 1H), 4.93 (dd, J = 7.7, 3.9 Hz, 1H), 2.66 (ddd, J = 12.9, 8.9, 4.2 Hz, 1H), 2.48 (ddd, J = 12.5, 5.6, 3.8 Hz, 1H), 2.30 (s, 6H), 1.95 – 1.76 (m, 2H). ^{13}C NMR (101 MHz, $CDCl_3$) δ 145.24, 128.31, 126.99, 125.70, 75.88, 58.57, 45.45, 34.66. HRMS (EI): exact mass calculated for $C_{11}H_{17}NO$ $[M]^+$ 179.1310, found 179.1312.

(*S*)-1-(2-chlorophenyl)-3-(dimethylamino)propan-1-ol (6b)

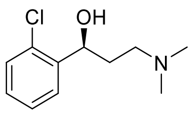

Colorless oil, 90% yield, 96% ee. $[\alpha]_D^{25}$ = -26.7 (c 1.0, CH_2Cl_2). The ee was determined by HPLC on Chiralpak OD-H column, hexane:isopropanol:Et_2NH= 95:5:0.2; flow rate = 0.8 mL/min; UV detection at 254 nm; t_R = 6.914 min (major), t_R = 7.365 min (minor). ^{1}H NMR (400 MHz, $CDCl_3$) δ 7.69 – 7.66 (m, 1H), 7.33 – 7.28 (m, 2H), 7.20 – 7.15 (m, 1H), 5.27 (dd,

J = 8.1, 3.0 Hz, 1H), 2.68 (ddd, J = 12.7, 9.8, 3.0 Hz, 1H), 2.48 (ddd, J = 12.6, 6.3, 3.1 Hz, 1H), 2.32 (s, 6H), 2.03 – 1.94 (m, 1H), 1.75 – 1.64 (m, 1H). ^{13}C NMR (101 MHz, $CDCl_3$) δ 142.41, 131.41, 129.32, 128.04, 127.53, 126.97, 72.79, 58.51, 45.40, 32.12. HRMS (EI): exact mass calculated for $C_{11}H_{16}ClNO$ $[M]^+$ 213.0920, found 213.0923.

(*S*)-1-(3-chlorophenyl)-3-(dimethylamino)propan-1-ol (6c)

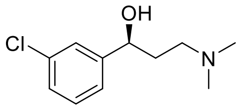

White solid, 95% yield, 99% ee. $[\alpha]_D^{25}$ = -12.1 (c 1.0, CH_2Cl_2). The ee was determined by HPLC on Chiralpak AD-H column, hexane:isopropanol:Et_2NH = 95:5:0.2; flow rate = 0.8 mL/min; UV detection at 254 nm; t_R = 9.437 min (minor), t_R = 14.137 min (minor). ^{1}H NMR (400 MHz, $CDCl_3$) δ 7.39 (s, 1H), 7.32 – 7.16 (m, 3H), 4.89 (dd, J = 8.3, 3.4 Hz, 1H), 2.67 (ddd, J = 13.0, 9.4, 3.7 Hz, 1H), 2.49 (ddd, J = 12.6, 5.7, 3.6 Hz, 1H), 2.31 (s, 6H), 1.95 – 1.70 (m, 2H). ^{13}C NMR (151 MHz, $CDCl_3$) δ 147.36, 134.22, 129.55, 127.04, 125.89, 123.81, 75.06, 58.31, 45.28, 34.40. HRMS (EI): exact mass calculated for $C_{11}H_{16}ClNO$ $[M]^+$ 213.0920, found 213.0922.

(*S*)-1-(3-bromophenyl)-3-(dimethylamino)propan-1-ol (6d)

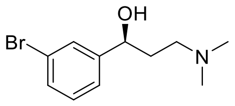

White solid, 88% yield, 99% ee. $[\alpha]_D^{25}$ = -8.1 (c 1.0, CH_2Cl_2). The ee was determined by HPLC on Chiralpak AD-H column, hexane:isopropanol = 95:5; flow rate = 0.8 mL/min; UV detection at 220 nm; t_R = 11.408 min (major), t_R = 20.656 min (minor). ^{1}H NMR (400 MHz, $CDCl_3$) δ 7.61 – 7.50 (m, 1H), 7.36 (ddd, J = 7.8, 2.1, 1.2 Hz, 1H), 7.32 – 7.25 (m, 1H), 7.19 (t, J = 7.8 Hz, 1H), 4.89 (dd, J = 8.2, 3.4 Hz, 1H), 2.66 (ddd, J = 12.9, 9.6, 3.5 Hz, 1H), 2.47 (ddd, J = 12.6, 5.8, 3.4 Hz, 1H), 2.30 (s, 6H), 1.91 – 1.67 (m, 2H). ^{13}C NMR (101 MHz, $CDCl_3$) δ 147.69, 129.98, 129.87, 128.85, 124.31, 122.56, 75.25, 58.44, 45.37, 34.46. HRMS (EI): exact mass calculated for $C_{11}H_{16}BrNO$ $[M]^+$ 257.0415, found 257.0417.

(*S*)-1-(4-chlorophenyl)-3-(dimethylamino)propan-1-ol (6e)

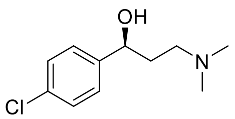

White solid, 91% yield, 99% ee. $[\alpha]_D^{25}$ = -16.4 (c 1.0, CH_2Cl_2). The ee was determined by HPLC on Chiralpak AD-H column, hexane:isopropanol:Et_2NH = 95:5:0.2; flow rate = 0.8 mL/min; UV detection at 254 nm; t_R = 10.487 min (major), t_R = 12.868 min (minor). ^{1}H NMR (400 MHz, $CDCl_3$) δ 7.30 (m, 4H), 4.90 (dd, J = 8.1, 3.5 Hz, 1H), 2.65 (ddd, J = 12.9, 9.4, 3.8 Hz, 1H),

2.46 (ddd, J = 12.6, 5.6, 3.5 Hz, 1H), 2.29 (s, 6H), 1.87 – 1.68 (m, 2H). ^{13}C NMR (151 MHz, $CDCl_3$) δ 143.76, 132.50, 128.38, 127.07, 75.23, 58.44, 45.37, 34.53. HRMS (EI): exact mass calculated for $C_{11}H_{16}ClNO$ $[M]^+$ 213.0920, found 213.0919.

(*S*)-1-(4-bromophenyl)-3-(dimethylamino)propan-1-ol (6f)

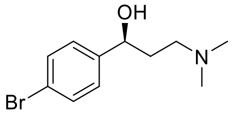

White solid, 94% yield, >99% ee. $[\alpha]_D^{25}$ = -13.2 (c 1.0, CH_2Cl_2). The ee was determined by HPLC on Chiralpak AD-H column, hexane:isopropanol = 95:5; flow rate = 0.8 mL/min; UV detection at 220 nm; t_R = 11.453 min (major). ^{1}H NMR (400 MHz, $CDCl_3$) δ 7.54 – 7.35 (m, 2H), 7.25 (d, J = 8.2 Hz, 2H), 4.88 (dd, J = 8.2, 3.4 Hz, 1H), 2.65 (ddd, J = 12.9, 9.5, 3.6 Hz, 1H), 2.46 (ddd, J = 9.3, 6.0, 2.8 Hz, 1H), 2.29 (s, 6H), 1.89 – 1.66 (m, 2H). ^{13}C NMR (101 MHz, $CDCl_3$) δ 144.30, 131.31, 127.45, 120.59, 75.29, 58.44, 45.38, 34.47. HRMS (EI): exact mass calculated for $C_{11}H_{16}BrNO$ $[M]^+$ 257.0415, found 257.0413.

(*S*)-1-(4-(tert-butyl)phenyl)-3-(dimethylamino)propan-1-ol (6g)

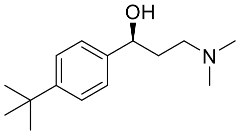

White solid, 90% yield, >99% ee. $[\alpha]_D^{25}$ = -10.8 (c 1.0, CH_2Cl_2). The ee was determined by HPLC on Chiralpak AD-H column, hexane:isopropanol = 95:5; flow rate = 0.8 mL/min; UV detection at 220 nm; t_R = 9.975 min (major), t_R = 11.099 min (minor). ^{1}H NMR (400 MHz, $CDCl_3$) δ 7.41 – 7.36 (m, 2H), 7.32 (d, J = 8.4 Hz, 2H), 4.90 (t, J = 5.9 Hz, 1H), 2.66 (ddd, J = 13.0, 7.2, 6.0 Hz, 1H), 2.48 (dt, J = 12.5, 4.8 Hz, 1H), 2.30 (s, 6H), 1.89 – 1.77 (m, 2H), 1.33 (s, 9H). ^{13}C NMR (101 MHz, $CDCl_3$) δ 149.71, 142.18, 125.35, 125.13, 75.56, 58.59, 45.42, 34.62, 34.51, 31.50. HRMS (EI): exact mass calculated for $C_{15}H_{25}NO$ $[M]^+$ 235.1936, found 235.1938.

(*S*)-3-morpholino-1-(thiophen-2-yl)propan-1-ol (6h)

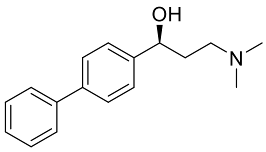

White solid, 94% yield, >99% ee. $[\alpha]_D^{25}$ = -16.6 (c 1.0, CH_2Cl_2). The ee was determined by HPLC on Chiralpak AD-H column, hexane:isopropanol = 95:5. flow rate = 0.8 mL/min; UV detection at 220 nm; t_R = 14.297 min (major), t_R = 17.425 min (minor). ^{1}H NMR (400 MHz, $CDCl_3$) δ 7.62 (ddd, J = 8.6, 7.5, 1.8 Hz, 4H), 7.51 – 7.42 (m, 4H), 7.39 – 7.32 (m, 1H), 5.00

(dd, *J* = 7.4, 4.3 Hz, 1H), 2.69 (ddd, *J* = 12.9, 8.4, 4.8 Hz, 1H), 2.52 (ddd, *J* = 12.5, 5.4, 4.1 Hz, 1H), 2.33 (s, 6H), 1.97 – 1.81 (m, 2H). ^{13}C NMR (101 MHz, $CDCl_3$) δ 144.32, 141.13, 139.80, 128.76, 127.13, 127.10, 126.99, 126.07, 75.51, 58.48, 45.37, 34.62. HRMS (EI): exact mass calculated for $C_{17}H_{21}NO$ $[M]^+$ 255.1623, found 255.1625.

(*S*)-3-(dimethylamino)-1-(naphthalen-2-yl)propan-1-ol (6i)

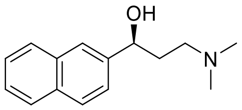

White solid, 91% yield, >99% ee. $[\alpha]_D^{25}$ = -20.0 (c 1.0, CH_2Cl_2). The ee was determined by HPLC on Chiralpak AD-H column, hexane:isopropanol = 95:5. flow rate = 0.8 mL/min; UV detection at 220 nm; t_R = 24.774 min (major), t_R = 36.366 min (minor). ^{1}H NMR (400 MHz, $CDCl_3$) δ 7.98 – 7.71 (m, 4H), 7.57 – 7.38 (m, 3H), 5.11 (dd, *J* = 8.0, 3.5 Hz, 1H), 2.69 (ddd, *J* = 12.8, 9.2, 3.8 Hz, 1H), 2.51 (ddd, *J* = 12.5, 5.8, 3.6 Hz, 1H), 2.33 (s, 6H), 2.00 – 1.83 (m, 2H). ^{13}C NMR (101 MHz, $CDCl_3$) δ 142.66, 133.53, 132.83, 128.08, 127.96, 127.73, 126.01, 125.56, 124.29, 124.12, 75.89, 58.50, 45.43, 34.52. HRMS (EI): exact mass calculated for $C_{15}H_{19}NO$ $[M]^+$ 299.1467, found 299.1464.

(*S*)-1-(benzo[b]thiophen-2-yl)-3-(dimethylamino)propan-1-ol (6j)

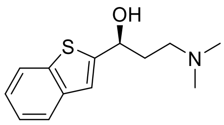

White solid, 92% yield, >99% ee. $[\alpha]_D^{25}$ = 3.4 (*c* 1.0, CH_2Cl_2). The ee was determined by HPLC on Chiralpak AD-H column, hexane:isopropanol:Et_2NH = 95:5:0.2; flow rate = 0.8 mL/min; UV detection at 254 nm; t_R = 18.846 min (major), t_R = 25.414 min (minor). ^{1}H NMR (400 MHz, $CDCl_3$) δ 7.80 (d, *J* = 6.9 Hz, 1H), 7.70 (dd, *J* = 7.5, 1.4 Hz, 1H), 7.37 – 7.23 (m, 2H), 7.16 (s, 1H), 5.29 – 5.23 (m, 1H), 2.73 – 2.56 (m, 2H), 2.31 (s, 6H), 2.15 – 1.89 (m, 2H). ^{13}C NMR (101 MHz, $CDCl_3$) δ 150.72, 140.04, 139.34, 124.13, 123.71, 123.29, 122.45, 118.86, 72.52, 57.97, 45.34, 34.18. HRMS (EI): exact mass calculated for $C_{13}H_{17}NOS$ $[M]^+$ 235.1031, found 235.1033.

(*S*)-3-(dimethylamino)-1-(furan-2-yl)propan-1-ol (6k)

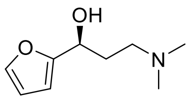

Colorless oil, 90% yield, 93% ee. $[\alpha]_D^{25}$ = -9.4 (*c* 1.0, CH_2Cl_2). The ee was determined by HPLC on Chiralpak AD-H column, hexane:isopropanol:Et_2NH = 95:5:0.02; flow rate = 0.8 mL/min; UV detection at 220 nm; t_R = 11.369 min (major), t_R = 12.891 min (minor). ^{1}H NMR (400 MHz,

$CDCl_3$) δ 7.37 – 7.27 (m, 1H), 6.29 (dd, *J* = 3.3, 1.9 Hz, 1H), 6.26 – 6.17 (m, 1H), 4.89 (dd, *J* = 7.9, 3.6 Hz, 1H), 2.75 – 2.48 (m, 2H), 2.29 (s, 6H), 2.04 – 1.83 (m, 2H). ^{13}C NMR (101 MHz, $CDCl_3$) δ 156.19, 140.53, 109.07, 104.36, 68.25, 56.78, 44.13, 29.87. HRMS (EI): exact mass calculated for $C_9H_{15}NO_2$ $[M]^+$ 169.1103, found 169.1105.

(*S*)-3-(pyrrolidin-1-yl)-1-(thiophen-2-yl)propan-1-ol (6l)

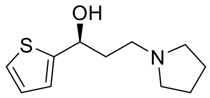

White solid, 90% yield, 99% ee. $[\alpha]_D^{25}$ = -6.9 (*c* 1.0, MeOH). The ee was determined by HPLC on Chiralpak AD-H column, hexane:isopropanol:Et_2NH = 95:5:0.2; flow rate = 0.8 mL/min; UV detection at 254 nm; t_R = 10.723 min (major), t_R = 13.003 min (minor). ^1H NMR (400 MHz, $CDCl_3$) δ 7.20 (dd, *J* = 5.0, 1.2 Hz, 1H), 6.96 (dd, *J* = 5.0, 3.5 Hz, 1H), 6.93 – 6.90 (m, 1H), 5.18 (dd, *J* = 7.9, 3.6 Hz, 1H), 2.91 (ddd, *J* = 12.9, 9.1, 4.0 Hz, 1H), 2.77 – 2.64 (m, 3H), 2.62 – 2.52 (m, 2H), 2.06 – 1.90 (m, 2H), 1.84 – 1.73 (m, 4H). ^{13}C NMR (151 MHz, $CDCl_3$) δ 149.85, 126.64, 123.71, 122.26, 72.16, 54.56, 54.12, 35.79, 23.52. HRMS (EI): exact mass calculated for $C_{11}H_{17}NOS$ $[M]^+$ 211.1031, found 211.1033.

(*S*)-3-(piperidin-1-yl)-1-(thiophen-2-yl)propan-1-ol (6m)

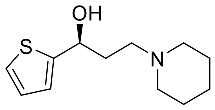

Colorless oil, 96% yield, 99% ee. $[\alpha]_D^{25}$ = 3.5 (*c* 1.0, CH_2Cl_2). The ee was determined by HPLC on Chiralpak AD-H column, hexane:isopropanol:Et_2NH = 95:5:0.2; flow rate = 0.8 mL/min; UV detection at 254 nm; t_R = 7.109 min (major). t_R = 8.571 min (minor). ^1H NMR (400 MHz, $CDCl_3$) δ 7.20 (dd, *J* = 5.1, 1.2 Hz, 1H), 6.96 (dd, *J* = 5.0, 3.5 Hz, 1H), 6.91 (dt, *J* = 3.5, 1.2 Hz, 1H), 5.18 (ddd, *J* = 6.7, 4.4, 1.0 Hz, 1H), 2.76 – 2.26 (m, 6H), 2.14 – 1.84 (m, 2H), 1.61 (p, *J* = 5.6 Hz, 4H), 1.54 – 1.34 (m, 2H). ^{13}C NMR (101 MHz, $CDCl_3$) δ 149.97, 126.69, 123.71, 122.21, 72.25, 57.63, 54.72, 33.79, 26.11, 24.28. HRMS (EI): exact mass calculated for $C_{12}H_{19}NOS$ $[M]^+$ 225.1187, found 225.1189.

(*S*)-3-morpholino-1-(thiophen-2-yl)propan-1-ol (6n)

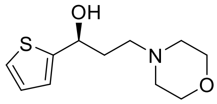

White solid, 89% yield, >99% ee. $[\alpha]_D^{25}$ =7.1 (*c* 1.0, CH_2Cl_2). The ee was determined by HPLC on Chiralpak AD-H column, hexane:isopropanol = 95:5; flow rate = 0.8 mL/min; UV detection at 220 nm; t_R = 19.106 min (major), t_R = 23.751 min (minor). ^1H NMR (400 MHz, $CDCl_3$) δ

7.20 (dd, *J* = 5.0, 1.2 Hz, 1H), 6.96 (dd, *J* = 5.0, 3.5 Hz, 1H), 6.91 (dt, *J* = 3.5, 1.1 Hz, 1H), 5.18 (ddd, *J* = 6.1, 4.8, 1.0 Hz, 1H), 3.72 (t, *J* = 4.7 Hz, 5H), 2.85 – 2.33 (m, 6H), 2.08 – 1.86 (m, 2H). ^{13}C NMR (101 MHz, $CDCl_3$) δ 149.34, 126.72, 123.88, 122.40, 72.02, 66.97, 57.31, 53.71, 33.51. HRMS (EI): exact mass calculated for $C_{11}H_{17}NO_2S$ $[M]^+$ 227.0980, found 227.0982.

(*S*)-3-(3,4-dihydroisoquinolin-2(1H)-yl)-1-(thiophen-2-yl)propan-1-ol (6o)

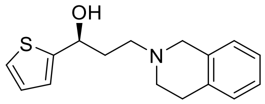

Colorless oil, 97% yield, >99% ee. $[\alpha]_D^{25}$ = -6.1 (*c* 1.0, CH_2Cl_2). The ee was determined by HPLC on Chiralpak AD-H column, hexane:isopropanol = 95:5; flow rate = 0.8 mL/min; UV detection at 220 nm; t_R = 18.332 min (major), t_R = 24.987 min (minor). ^1H NMR (400 MHz, $CDCl_3$) δ 7.23 (dd, *J* = 5.0, 1.2 Hz, 1H), 7.19 – 7.10 (m, 3H), 7.08 – 7.02 (m, 1H), 7.00 (dd, *J* = 5.0, 3.5 Hz, 1H), 6.96 – 6.94 (m, 1H), 5.24 (ddd, *J* = 7.4, 3.8, 0.9 Hz, 1H), 3.80 (d, *J* = 14.9 Hz, 1H), 3.71 (d, *J* = 14.9 Hz, 1H), 3.04 – 2.75 (m, 6H), 2.16 – 2.04 (m, 2H). ^{13}C NMR (101 MHz, $CDCl_3$) δ 148.64, 132.91, 132.81, 127.67, 125.69, 125.61, 125.47, 124.86, 122.79, 121.36, 70.89, 55.38, 55.16, 49.68, 33.14, 27.86. HRMS (EI): exact mass calculated for $C_{11}H_{17}NO_2S$ $[M]^+$ 273.1187, found 273.1189.

(*S*)-3-(methyl(naphthalen-1-ylmethyl)amino)-1-(thiophen-2-yl)propan-1-ol (6p)

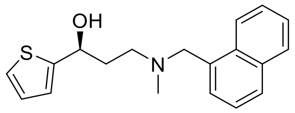

Colorless oil, 95% yield, 99% ee. $[\alpha]_D^{25}$ = -27.2 (*c* 1.0, CH_2Cl_2). The ee was determined by HPLC on Chiralpak OD-H column, hexane:isopropanol = 95:5; flow rate = 0.8 mL/min; UV detection at 220 nm; t_R = 15.012 min (major), t_R = 17.051 min (minor). ^1H NMR (400 MHz, $CDCl_3$) δ 8.23 (dd, *J* = 8.6, 1.2 Hz, 1H), 7.91 (dd, *J* = 8.2, 1.3 Hz, 1H), 7.85 (dd, *J* = 6.2, 3.4 Hz, 1H), 7.63 (ddd, *J* = 8.5, 6.8, 1.4 Hz, 1H), 7.54 (ddd, *J* = 8.1, 6.8, 1.2 Hz, 1H), 7.47 – 7.40 (m, 2H), 7.19 (dd, *J* = 5.1, 1.2 Hz, 1H), 6.92 (dd, *J* = 5.0, 3.5 Hz, 1H), 6.82 – 6.74 (m, 1H), 5.14 – 4.98 (m, 1H), 4.31 – 3.81 (m, 2H), 2.91 – 2.66 (m, 2H), 2.36 (s, 3H), 2.05 (q, *J* = 5.9 Hz, 2H). ^{13}C NMR (101 MHz, $CDCl_3$) δ 149.38, 133.93, 133.40, 132.31, 128.66, 128.42, 128.00, 126.52, 126.37, 125.88, 125.14, 124.03, 123.67, 122.43, 71.74, 61.06, 56.09, 41.93, 34.86. HRMS (EI): exact mass calculated for $C_{11}H_{17}NO_2S$ $[M]^+$ 311.1344, found 311.1346.

(*S*)-1-(3-hydroxy-3-(thiophen-2-yl)propyl)piperidin-4-ol (6q)

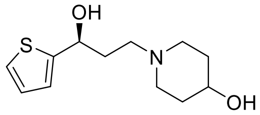

Colorless oil, 97% yield, >99% ee. $[\alpha]_D^{25}$ = -2.0 (*c* 1.0, CH_2Cl_2). The ee was determined by HPLC on Chiralpak OD-H column, hexane:isopropanol:Et_2NH = 95:5:0.1; flow rate = 0.8 mL/min; UV detection at 254 nm; t_R = 31.098 min (major). ^1H NMR (400 MHz, $CDCl_3$) δ 7.20 (dd, *J* = 5.0, 1.3 Hz, 1H), 6.96 (dd, *J* = 5.0, 3.5 Hz, 1H), 6.90 (d, *J* = 3.3 Hz, 1H), 5.16 (dd, *J* = 7.1, 4.0 Hz, 1H), 3.70 (s, 1H), 2.90 (s, 1H), 2.80 (s, 1H), 2.69 – 2.58 (m, 2H), 2.30 (s, 1H), 2.14 (s, 1H), 2.01 – 1.83 (m, 4H), 1.67 – 1.50 (m, 2H). ^{13}C NMR (101 MHz, $CDCl_3$) δ 148.52, 125.71, 122.82, 121.38, 71.07, 66.30, 55.70, 50.41, 49.78, 33.49, 33.38, 33.02. HRMS (EI): exact mass calculated for $C_{12}H_{19}NO_2S$ $[M]^+$ 241.1136, found 241.1138.

(*S*)-3-(4-cyclohexylpiperazin-1-yl)-1-(thiophen-2-yl)propan-1-ol (6r)

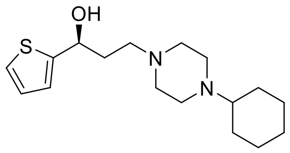

Colorless oil, 94% yield, 99% ee. $[\alpha]_D^{25}$ = -8.3 (*c* 1.0, CH_2Cl_2). The ee was determined by HPLC on Chiralpak AD-H column, hexane:isopropanol:Et_2NH = 95:5:0.2; flow rate = 0.8 mL/min; UV detection at 254 nm; t_R = 11.556 min (major), t_R = 16.782 min (minor). ^1H NMR (400 MHz, $CDCl_3$) δ 7.18 (dd, *J* = 5.0, 1.2 Hz, 1H), 6.94 (dd, *J* = 5.0, 3.5 Hz, 1H), 6.89 (d, *J* = 3.4 Hz, 1H), 5.15 (dd, *J* = 6.4, 4.8 Hz, 1H), 2.80 – 2.41 (m, 10H), 2.29 – 2.13 (m, 1H), 1.94 (q, *J* = 4.6, 4.2 Hz, 2H), 1.88 – 1.73 (m, 4H), 1.67 – 1.54 (m, 1H), 1.29 – 1.02 (m, 5H). ^{13}C NMR (101 MHz, $CDCl_3$) δ 149.63, 126.59, 123.65, 122.15, 72.06, 63.39, 56.79, 53.66, 48.88, 33.62, 28.96, 26.28, 25.86. HRMS (EI): exact mass calculated for $C_{17}H_{28}N_2OS$ $[M]^+$ 308.1922, found 308.1924.

(*S*)-(4-(3-hydroxy-3-(thiophen-2-yl)propyl)piperazin-1-yl)(phenyl)methanone (6s)

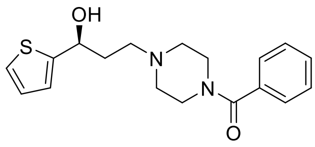

Colorless oil, 99% yield, 99% ee. $[\alpha]_D^{25}$ = 6.0 (*c* 1.0, CH_2Cl_2). The ee was determined by HPLC on Chiralpak AD-H column, hexane:isopropanol = 70:30; flow rate = 0.6 mL/min; UV detection at 220 nm; t_R = 15.491 min (major), t_R = 18.644 min (minor). ^1H NMR (400 MHz, $CDCl_3$) δ 7.44 – 7.31 (m, 5H), 7.17 (dd, *J* = 5.0, 1.2 Hz, 1H), 6.93 (dd, *J* = 5.0, 3.5 Hz, 1H), 6.88 (d, *J* = 3.5 Hz, 1H), 5.13 (dd, *J* = 6.6, 4.8 Hz, 1H), 3.77 (s, 2H), 3.42 (s, 2H), 2.83 – 2.19 (m, 6H), 2.04 – 1.83 (m, 2H). ^{13}C NMR (101 MHz, $CDCl_3$) δ 170.20, 149.01, 135.41, 129.77, 128.46, 126.97, 126.62, 123.84, 122.43, 71.43, 56.42, 53.11, 47.49, 41.95, 33.85. HRMS (EI): exact mass calculated for $C_{18}H_{22}N_2O_2S$ $[M]^+$ 330.1402, found 330.1405.

tert-butyl (*S*)-4-(3-hydroxy-3-(thiophen-2-yl)propyl)piperazine-1-carboxylate (6t)

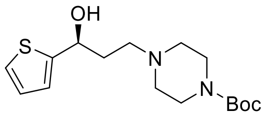

White solid, 91% yield, 99% ee. $[\alpha]_D^{25}$ = 5.0 (*c* 1.0, CH_2Cl_2). The ee was determined by HPLC on Chiralpak AD-H column, hexane:isopropanol = 90:10; flow rate = 0.8 mL/min; UV detection at 220 nm; t_R = 14.171 min (major), t_R = 17.479 min (minor). ^{1}H NMR (400 MHz, $CDCl_3$) δ 7.18 (dd, *J* = 5.0, 1.2 Hz, 1H), 6.94 (dd, *J* = 5.0, 3.5 Hz, 1H), 6.89 (dt, *J* = 3.5, 1.1 Hz, 1H), 5.19 – 5.09 (m, 1H), 3.43 (t, *J* = 5.1 Hz, 4H), 2.67 – 2.60 (m, 2H), 2.57 – 2.46 (m, 2H), 2.46 – 2.36 (m, 2H), 2.05 – 1.91 (m, 2H), 1.43 (s, 9H). ^{13}C NMR (101 MHz, $CDCl_3$) δ 154.59, 149.24, 126.66, 123.84, 122.39, 79.86, 71.79, 56.76, 53.03, 43.56, 33.79, 28.43. HRMS (EI): exact mass calculated for $C_{16}H_{26}N_2O_3S$ $[M]^+$ 326.1644, found 326.1645.

(*S*)-3-(pyrrolidin-1-yl)-1-(thiophen-2-yl)propan-1-ol (6u)

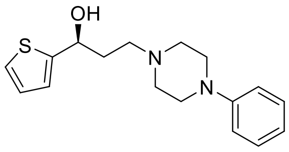

White solid, 95% yield, >99% ee. $[\alpha]_D^{25}$ = -16.0 (*c* 1.0, CH_2Cl_2). The ee was determined by HPLC on Chiralpak AD-H column, hexane:isopropanol = 90:10; flow rate = 0.8 mL/min; UV detection at 220 nm; t_R = 15.238 min (major), t_R = 21.473 min (minor). ^{1}H NMR (400 MHz, $CDCl_3$) δ 7.30 – 7.16 (m, 3H), 6.96 (dd, *J* = 5.0, 3.5 Hz, 1H), 6.94 – 6.83 (m, 14H), 5.18 (t, *J* = 5.6 Hz, 1H), 3.20 (t, *J* = 5.0 Hz, 4H), 2.91 – 2.50 (m, 6H), 2.16 – 1.92 (m, 2H). ^{13}C NMR (151 MHz, $CDCl_3$) δ 151.04, 149.36, 129.16, 126.67, 123.81, 122.36, 120.02, 116.26, 71.92, 56.75, 53.20, 49.24, 33.79. HRMS (EI): exact mass calculated for $C_{17}H_{22}N_2OS$ $[M]^+$ 302.1453, found 302.1456.

(*S*)-3-(4-(2-methoxyphenyl)piperazin-1-yl)-1-(thiophen-2-yl)propan-1-ol (6v)

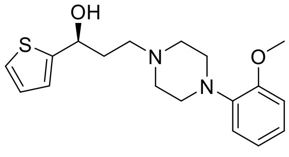

White solid, 95% yield, 99% ee. $[\alpha]_D^{25}$ = -16.7 (*c* 1.0, CH_2Cl_2). The ee was determined by HPLC on Chiralpak AD-H column, hexane:isopropanol = 90:10; flow rate = 0.8 mL/min; UV detection at 220 nm; t_R = 15.468 min (major), t_R = 21.371 min (minor). ^{1}H NMR (400 MHz, $CDCl_3$) δ 7.22 (dd, *J* = 5.0, 1.3 Hz, 1H), 7.08 – 6.96 (m, 2H), 6.93 (t, *J* = 3.5 Hz, 3H), 6.87 (d, *J* = 7.5 Hz, 1H), 5.21 (t, *J* = 5.7 Hz, 1H), 3.86 (s, 3H), 3.36 – 2.53 (m, 10H), 2.02 (q, *J* = 5.3 Hz, 2H). ^{13}C NMR (151 MHz, $CDCl_3$) δ 152.19, 149.49, 140.91, 126.60, 123.71, 123.12, 122.22,

121.02, 118.27, 111.15, 72.03, 56.83, 55.34, 53.38, 50.61, 33.64. HRMS (EI): exact mass calculated for $C_{18}H_{24}N_2O_2S$ $[M]^+$ 332.1558, found 332.1557.

(*S*)-3-(4-(2,3-dichlorophenyl)piperazin-1-yl)-1-(thiophen-2-yl)propan-1-ol (6w)

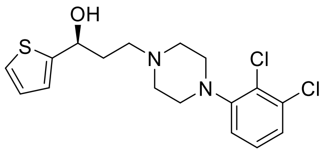

White solid, 99% yield, 99% ee. $[\alpha]_D^{25}$ = -8.3 (*c* 1.0, CH_2Cl_2). The ee was determined by HPLC on Chiralpak AD-H column, hexane:isopropanol = 90:10; flow rate = 0.8 mL/min; UV detection at 220 nm; t_R = 13.082 min (major), t_R = 18.405 min (minor). ^1H NMR (400 MHz, $CDCl_3$) δ 7.22 (dd, *J* = 5.0, 1.2 Hz, 1H), 7.20 – 7.12 (m, 2H), 6.98 (dd, *J* = 5.1, 3.5 Hz, 1H), 6.96 – 6.91 (m, 2H), 5.25 – 5.11 (m, 1H), 3.09 (s, 4H), 2.89 – 2.66 (m, 6H), 2.17 – 1.94 (m, 2H). ^{13}C NMR (101 MHz, $CDCl_3$) δ 150.92, 149.42, 134.04, 127.58, 127.55, 126.69, 124.84, 123.80, 122.31, 118.71, 72.05, 56.74, 53.29, 51.31, 33.70. HRMS (EI): exact mass calculated for $C_{17}H_{20}Cl_2N_2OS$ $[M]^+$ 370.0673, found 370.0676.

(*S*)-3-(4-(pyridin-2-yl)piperazin-1-yl)-1-(thiophen-2-yl)propan-1-ol (6x)

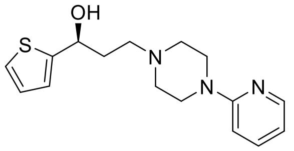

White solid, 94% yield, >99% ee. $[\alpha]_D^{25}$ = -2.1 (*c* 1.0, CH_2Cl_2). The ee was determined by HPLC on Chiralpak AD-H column, hexane:isopropanol = 90:10; flow rate = 0.8 mL/min; UV detection at 220 nm; t_R = 19.271 min (major), t_R = 29.576 min (minor). ^1H NMR (400 MHz, $CDCl_3$) δ 8.19 (dd, *J* = 5.4, 1.9 Hz, 1H), 7.62 – 7.37 (m, 1H), 7.21 (d, *J* = 1.2 Hz, 1H), 6.98 (dd, *J* = 5.0, 3.5 Hz, 1H), 6.93 (d, *J* = 3.5 Hz, 1H), 6.68 – 6.60 (m, 2H), 5.21 (dd, *J* = 6.6, 4.6 Hz, 1H), 3.67 – 3.42 (m, 4H), 2.79 – 2.66 (m, 4H), 2.60 (dt, *J* = 10.9, 5.1 Hz, 2H). 2.03 (q, *J* = 4.6 Hz, 2H). ^{13}C NMR (101 MHz, $CDCl_3$) δ 159.33, 149.35, 147.99, 137.58, 126.69, 123.85, 122.41, 113.60, 107.11, 71.94, 56.87, 53.06, 45.24, 33.81. HRMS (EI): exact mass calculated for $C_{16}H_{21}N_3OS$ $[M]^+$ 303.1405, found 303.1407.

(*S*)-3-(4-(pyrimidin-2-yl)piperazin-1-yl)-1-(thiophen-2-yl)propan-1-ol (6y)

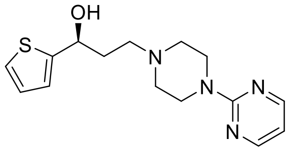

White solid, 94% yield, >99% ee. $[\alpha]_D^{25}$ = 12.9 (*c* 1.0, CH_2Cl_2). The ee was determined by HPLC on Chiralpak AD-H column, hexane:isopropanol = 90:10; flow rate = 0.8 mL/min; UV

detection at 220 nm; t_R = 16.972 min (major), t_R = 22.062 min (minor). ^{1}H NMR (400 MHz, $CDCl_3$) δ 8.27 (dd, *J* = 4.8, 1.3 Hz, 2H), 7.19 (dd, *J* = 5.0, 1.3 Hz, 1H), 7.04 – 6.92 (m, 1H), 6.91 (t, *J* = 2.1 Hz, 1H), 6.48 – 6.44 (m, 1H), 5.18 (t, *J* = 5.6 Hz, 1H), 3.83 (t, *J* = 5.1 Hz, 4H), 2.65 (ddd, *J* = 12.4, 9.5, 5.3 Hz, 4H), 2.56 – 2.37 (m, 2H), 2.04 – 1.96 (m, 2H). ^{13}C NMR (101 MHz, $CDCl_3$) δ 161.52, 157.73, 149.32, 126.68, 123.86, 122.40, 110.09, 71.90, 56.90, 53.13, 43.62, 33.78. HRMS (EI): exact mass calculated for $C_{15}H_{20}NO_4S$ [M]$^+$ 304.1358, found 304.1356.

tert-butyl (*S*)-6-(3-hydroxy-3-(thiophen-2-yl)propyl)-2,6-diazaspiro[3.3]heptane-2-carboxylate (6z)

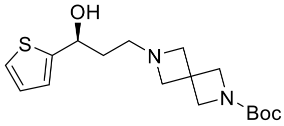

White solid, 92% yield, >99% ee. $[\alpha]_D^{25}$ = -16.1 (*c* 1.0, CH_2Cl_2). The ee was determined by HPLC on Chiralpak AD-H column, hexane:isopropanol = 90:10; flow rate = 0.8 mL/min; UV detection at 220 nm; t_R = 27.246 min (minor), t_R = 44.785 min (major). ^{1}H NMR (400 MHz, $CDCl_3$) δ 7.18 (d, *J* = 5.0 Hz, 1H), 6.93 (dd, *J* = 5.0, 3.6 Hz, 1H), 6.87 (d, *J* = 3.5 Hz, 1H), 5.07 (dd, *J* = 8.4, 3.1 Hz, 1H), 3.94 (s, 4H), 3.46 – 3.22 (m, 4H), 2.82 – 2.52 (m, 2H), 1.92 – 1.65 (m, 2H), 1.40 (s, 9H). ^{13}C NMR (101 MHz, $CDCl_3$) δ 156.05, 149.24, 126.61, 123.86, 122.48, 79.62, 71.48, 64.27, 59.21, 57.71, 35.20, 33.29, 28.40. HRMS (EI): exact mass calculated for $C_{17}H_{26}N_2O_3S$ [M]$^+$ 338.1664, found 338.1667.

(*S*)-3-(4-(bis(4-fluorophenyl)methyl)piperazin-1-yl)-1-phenylpropan-1-ol (6aa)

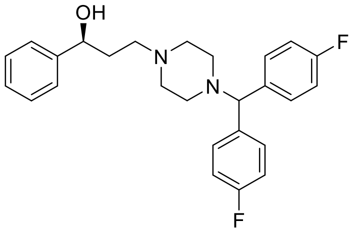

Colorless oil, 86% yield, >99% ee. $[\alpha]_D^{25}$ = -9.1 (*c* 1.0, CH_2Cl_2). The ee was determined by HPLC on Chiralpak AD-H column, hexane:isopropanol = 90:10; flow rate = 0.8 mL/min; UV detection at 220 nm; t_R = 18.041 min (major), t_R = 29.901 min (minor). ^{1}H NMR (400 MHz, $CDCl_3$) δ 7.34 (dt, *J* = 9.4, 5.1 Hz, 4H), 7.20 (dd, *J* = 5.0, 1.2 Hz, 1H), 7.11 – 6.81 (m, 6H), 5.17 (dd, *J* = 7.0, 4.1 Hz, 1H), 4.22 (s, 1H), 3.04 – 2.22 (m, 10H), 2.10 – 1.80 (m, 2H). ^{13}C NMR (101 MHz, $CDCl_3$) δ 163.09, 160.65, 149.58, 138.11 (d, *J* = 3.1 Hz), 129.29 (dd, *J* = 7.9, 2.5 Hz), 126.68, 123.76, 122.25, 115.50 (d, *J* = 21.3 Hz), 74.43, 72.10, 56.68, 53.39, 51.80, 33.70. HRMS (EI): exact mass calculated for $C_{26}H_{28}F_2N_2O$ [M]$^+$ 422.2170, found 422.2173.

(*S*)-1-(thiophen-2-yl)-3-(4-(2,3,4-trimethoxybenzyl)piperazin-1-yl)propan-1-ol (6ab)

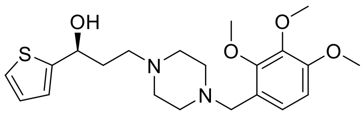

Colorless oil, 89% yield, >99% ee. $[\alpha]_D^{25}$ = -1.9 (*c* 1.0, CH_2Cl_2). The ee was determined by HPLC on Chiralpak AD-H column, hexane:isopropanol = 90:10; flow rate = 0.8 mL/min; UV detection at 220 nm; t_R = 15.662 min (major), t_R = 25.960 min (minor). ^{1}H NMR (400 MHz, $CDCl_3$) δ 7.18 (dd, *J* = 5.0, 1.2 Hz, 1H), 6.98 – 6.92 (m, 2H), 6.91 – 6.88 (m, 1H), 6.62 (d, *J* = 8.5 Hz, 1H), 5.20 – 5.11 (m, 1H), 3.86 (s, 3H), 3.86 (s, 3H), 3.83 (s, 3H), 3.46 (s, 2H), 2.94 – 2.22 (m, 10H), 2.01 – 1.87 (m, 2H). ^{13}C NMR (101 MHz, $CDCl_3$) δ 152.96, 152.69, 149.64, 142.31, 126.63, 125.19, 123.80, 123.70, 122.19, 106.95, 72.09, 61.21, 60.81, 56.75, 56.43, 56.00, 53.31, 52.85, 33.67. HRMS (EI): exact mass calculated for $C_{21}H_{30}N_2O_4S$ $[M]^+$ 406.1926, found 406.1928.

(*S*)-3-(4-(4-methoxyphenyl)piperazin-1-yl)-1-phenylpropan-1-ol (6ac)

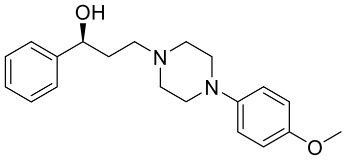

White solid, 98% yield, 99% ee. $[\alpha]_D^{25}$ = -45.4 (*c* 1.0, CH_2Cl_2). The ee was determined by HPLC on Chiralpak AD-H column, hexane:isopropanol = 90:10; flow rate = 0.8 mL/min; UV detection at 220 nm; t_R = 23.513 min (major), t_R = 33.407 min (minor). ^{1}H NMR (400 MHz, $CDCl_3$) δ 7.47 – 7.31 (m, 4H), 7.23 (t, *J* = 5.4 Hz, 1H), 7.13 – 6.67 (m, 4H), 4.92 (t, *J* = 5.8 Hz, 1H), 3.73 (s, 3H), 3.11 (t, *J* = 5.0 Hz, 4H), 2.80 – 2.66 (m, 1H), 2.66 – 2.55 (m, 1H), 1.87 (q, *J* = 5.9 Hz, 2H). ^{13}C NMR (101 MHz, $CDCl_3$) δ 153.95, 145.42, 144.84, 128.22, 126.93, 125.53, 118.35, 114.43, 75.39, 56.98, 55.51, 53.32, 50.72, 33.79. HRMS (EI): exact mass calculated for $C_{20}H_{26}N_2O_2$ $[M]^+$ 326.1994, found 326.1995.

(*S*)-3-(4-(2-methoxyphenyl)piperazin-1-yl)-1-phenylpropan-1-ol (6ad)

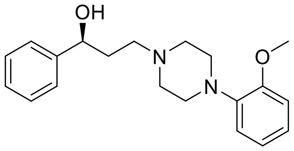

White solid, 93% yield, 99% ee. $[\alpha]_D^{25}$ = -45.6 (*c* 1.0, CH_2Cl_2). The ee was determined by HPLC on Chiralpak AD-H column, hexane:isopropanol = 90:10; flow rate = 0.8 mL/min; UV detection at 220 nm; t_R =14.096 min (major), t_R =21.632 min (minor). ^{1}H NMR (400 MHz, $CDCl_3$) δ 7.42 – 7.29 (m, 4H), 7.28 – 7.21 (m, 1H), 7.01 (ddd, *J* = 8.2, 6.0, 3.0 Hz, 1H), 6.95 – 6.90 (m, 1H), 6.89 – 6.75 (m, 1H), 4.96 (t, *J* = 5.7 Hz, 1H), 3.85 (s, 3H), 3.28 – 2.52 (m, 10H), 2.02 – 1.81 (m, 2H). ^{13}C NMR (101 MHz, $CDCl_3$) δ 151.27, 143.93, 140.02, 127.25, 125.94,

124.57, 122.16, 120.08, 117.33, 110.22, 74.61, 56.17, 54.40, 52.49, 49.72, 32.69. HRMS (EI): exact mass calculated for $C_{20}H_{26}N_2O_2$ $[M]^+$ 326.1994, found 326.1992.

2.4. NMR spectra

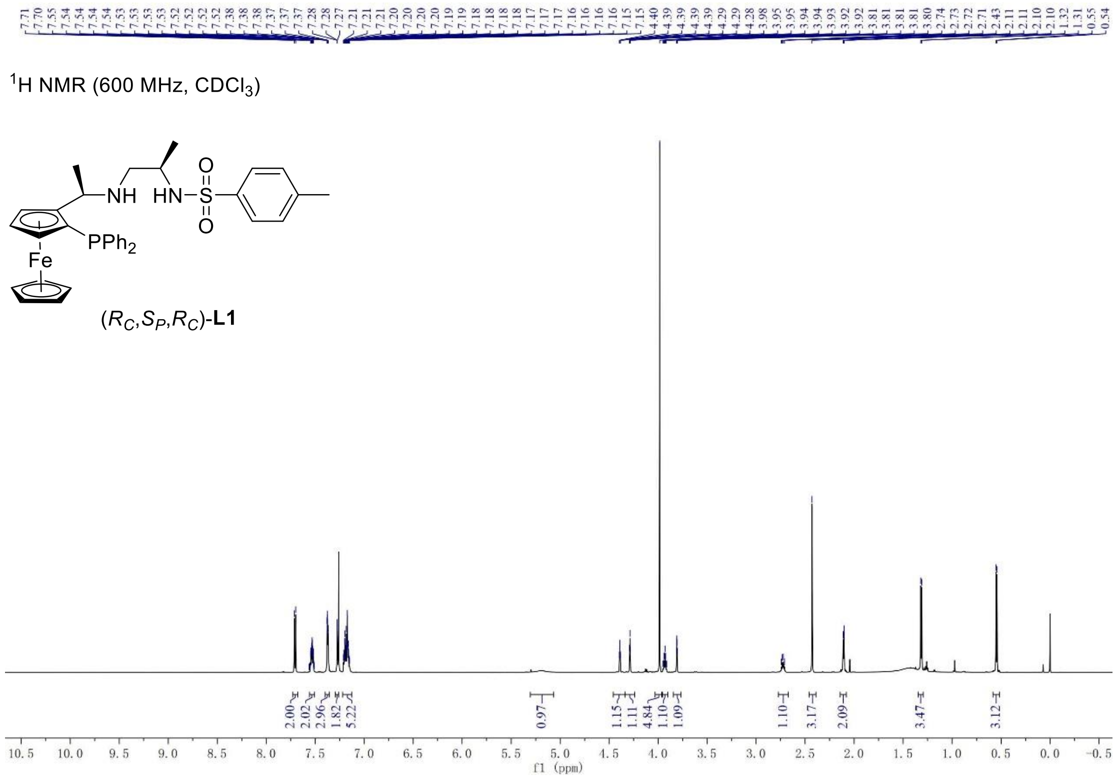

Supplementary Figure 1. ^{1}H NMR spectra of (R_C,S_P,R_C)-**L1**

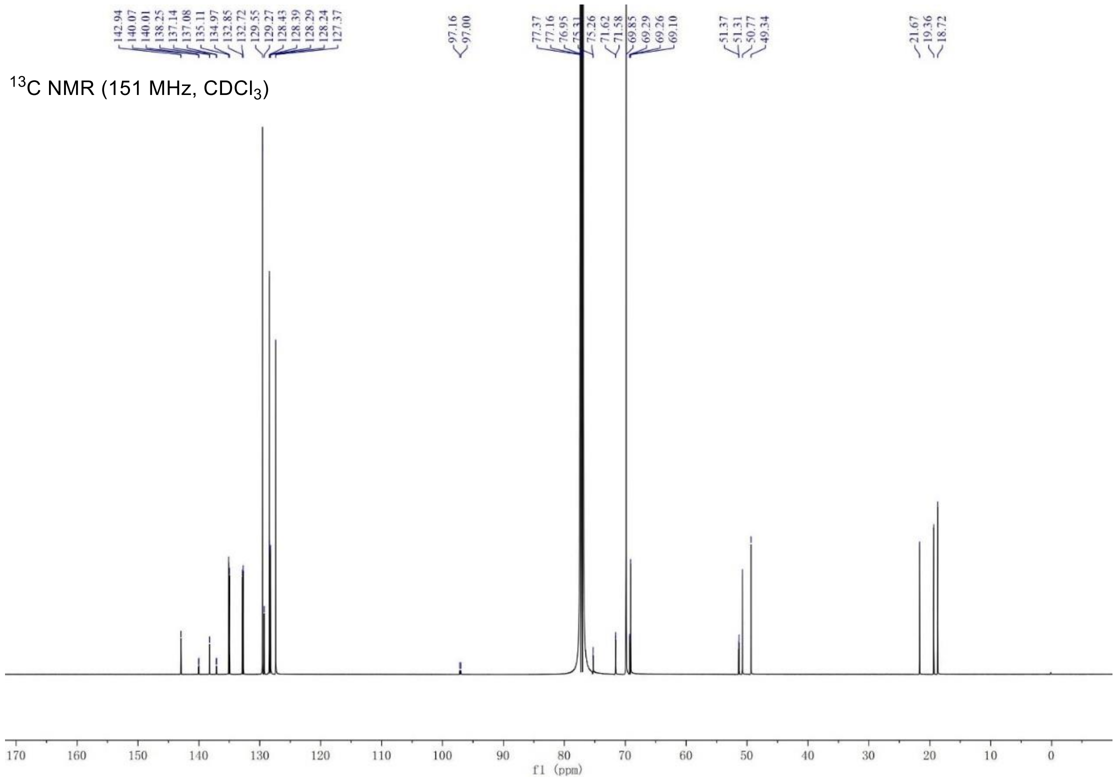

Supplementary Figure 2. ^{13}C NMR spectra of (R_C,S_P,R_C)-**L1**

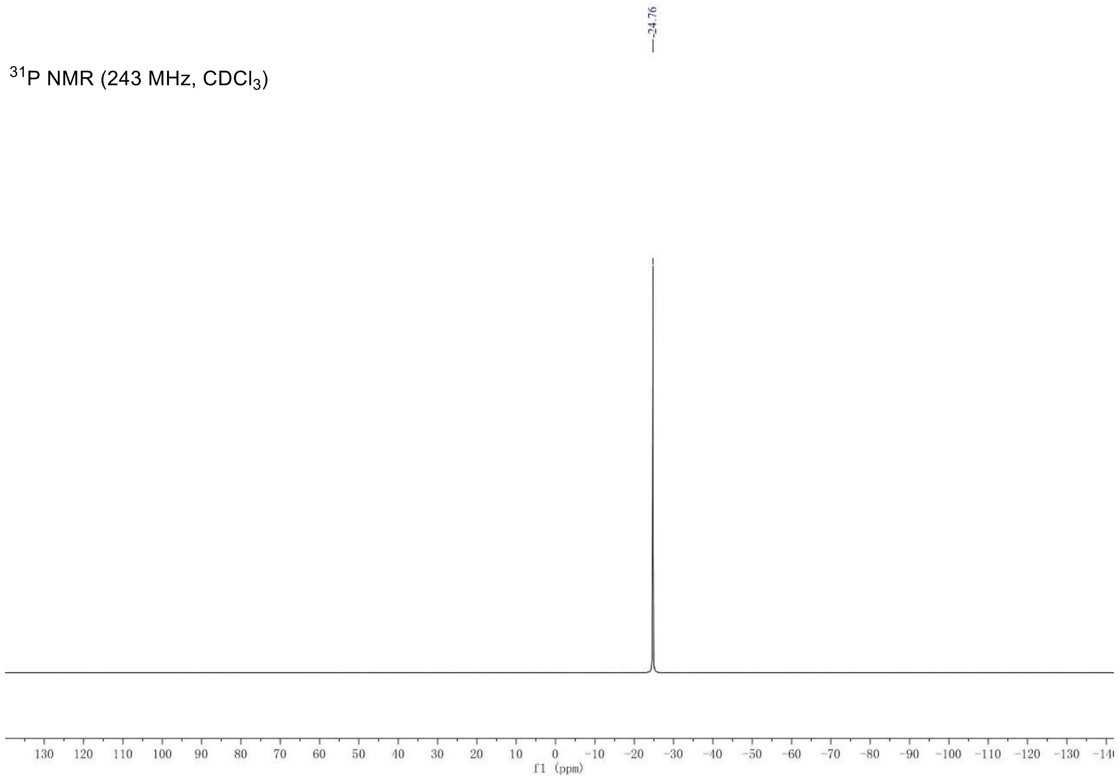

Supplementary Figure 3. ^{31}P NMR spectra of (R_C,S_P,R_C)-**L1**

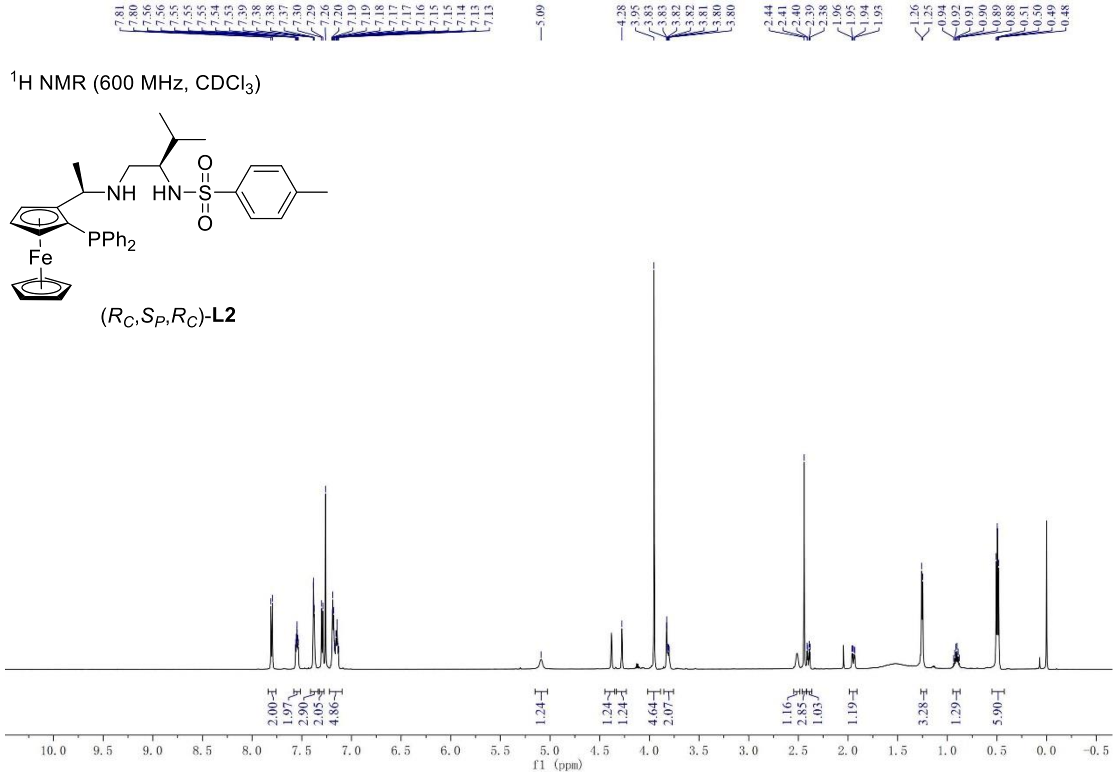

Supplementary Figure 4. ^{1}H NMR spectra of (R_C,S_P,R_C)-**L2**

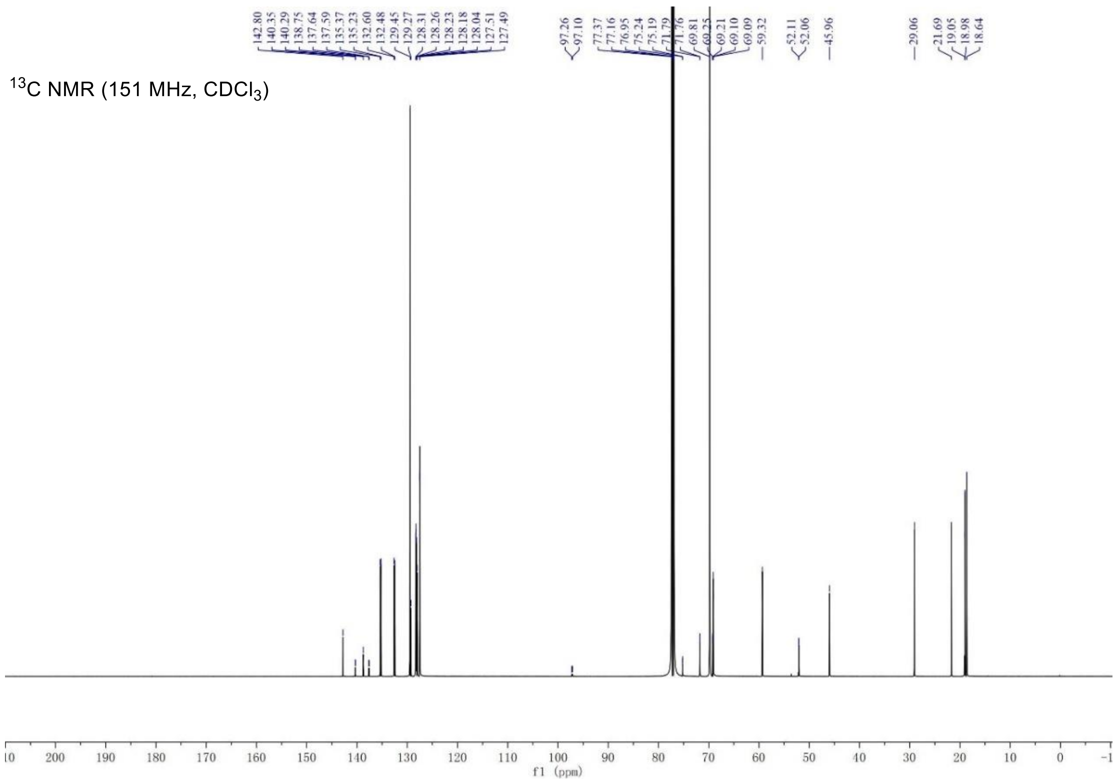

Supplementary Figure 5. ^{13}C NMR spectra of (R_C,S_P,R_C)-**L2**

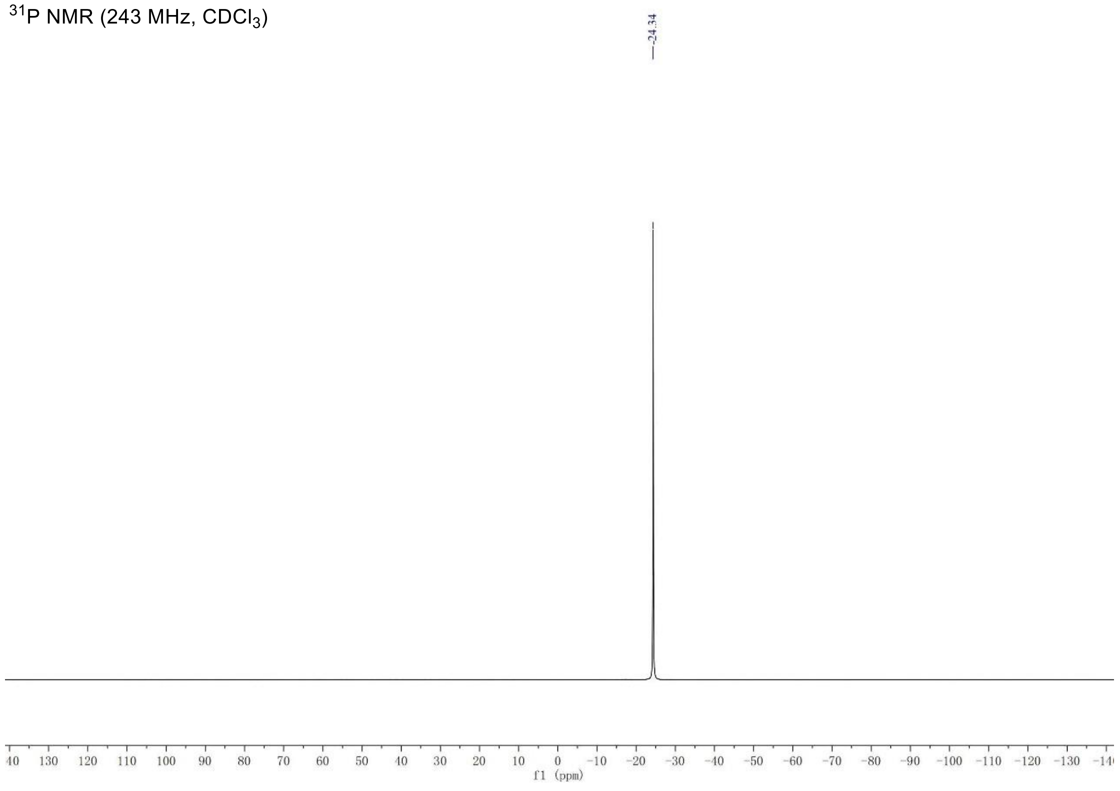

Supplementary Figure 6. ^{31}P NMR spectra of (R_C,S_P,R_C)-**L2**

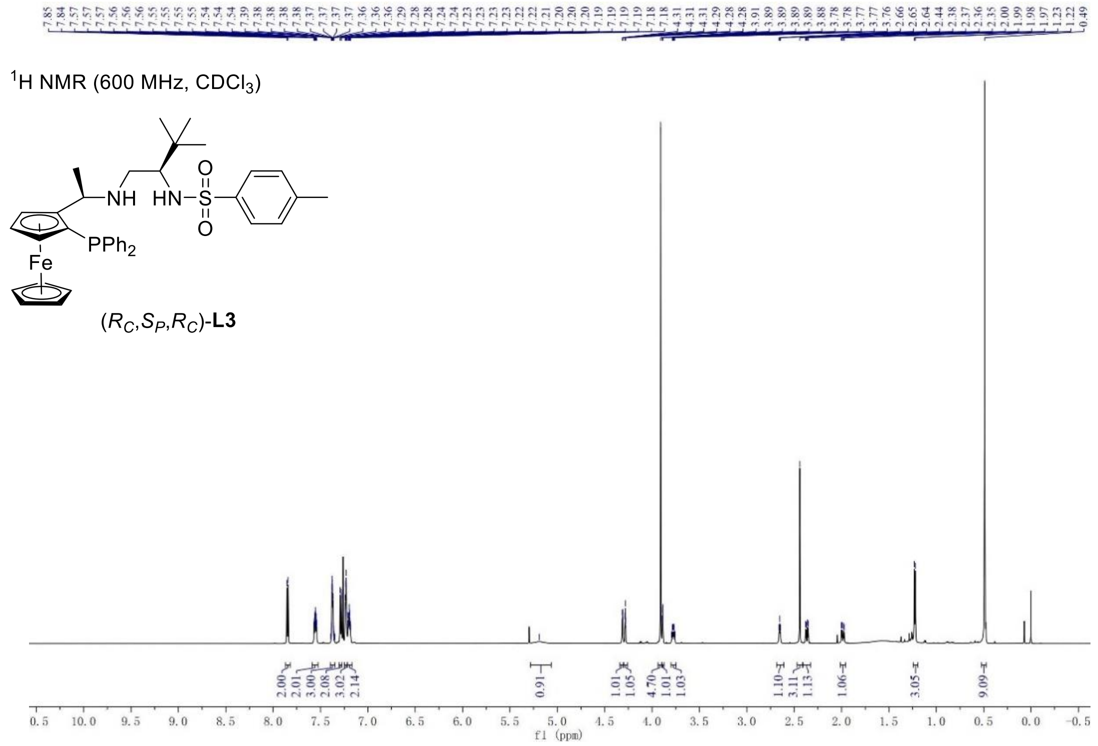

Supplementary Figure 7. ^{1}H NMR spectra of (R_C,S_P,R_C)-**L3**

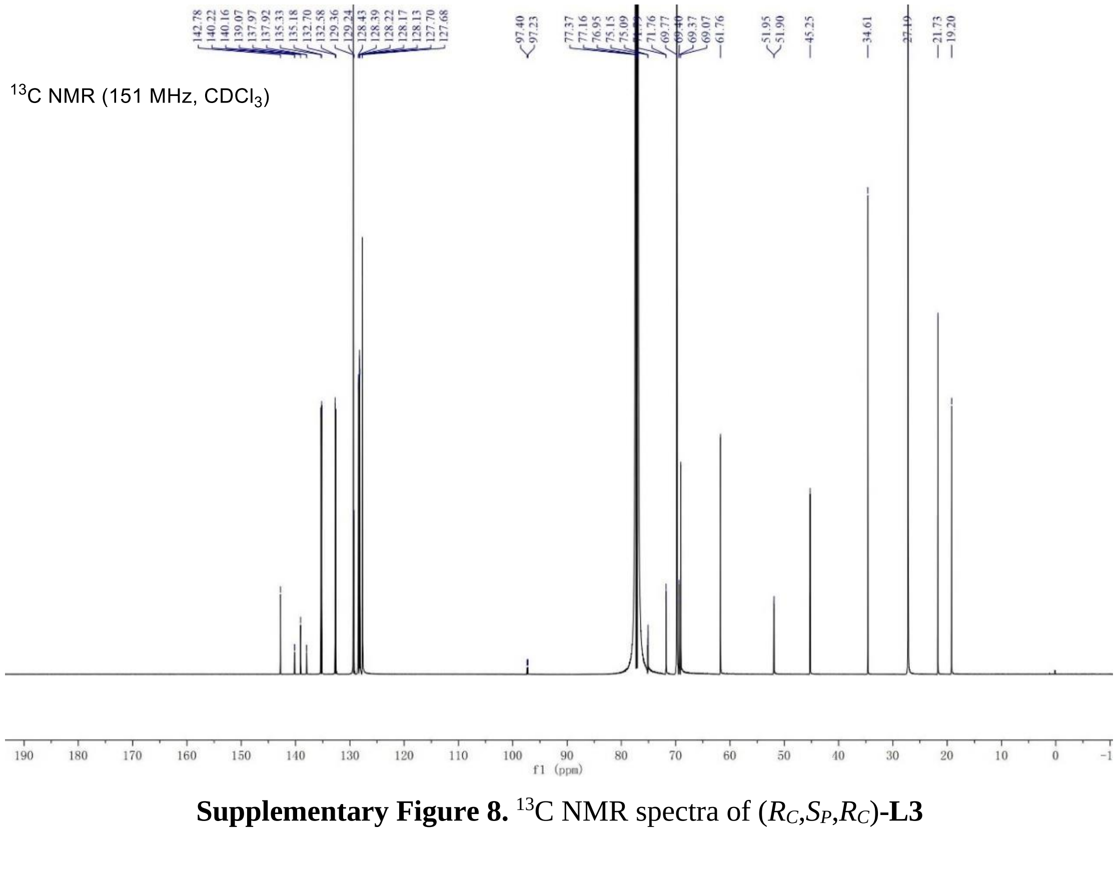

Supplementary Figure 8. ^{13}C NMR spectra of (R_C,S_P,R_C)-**L3**

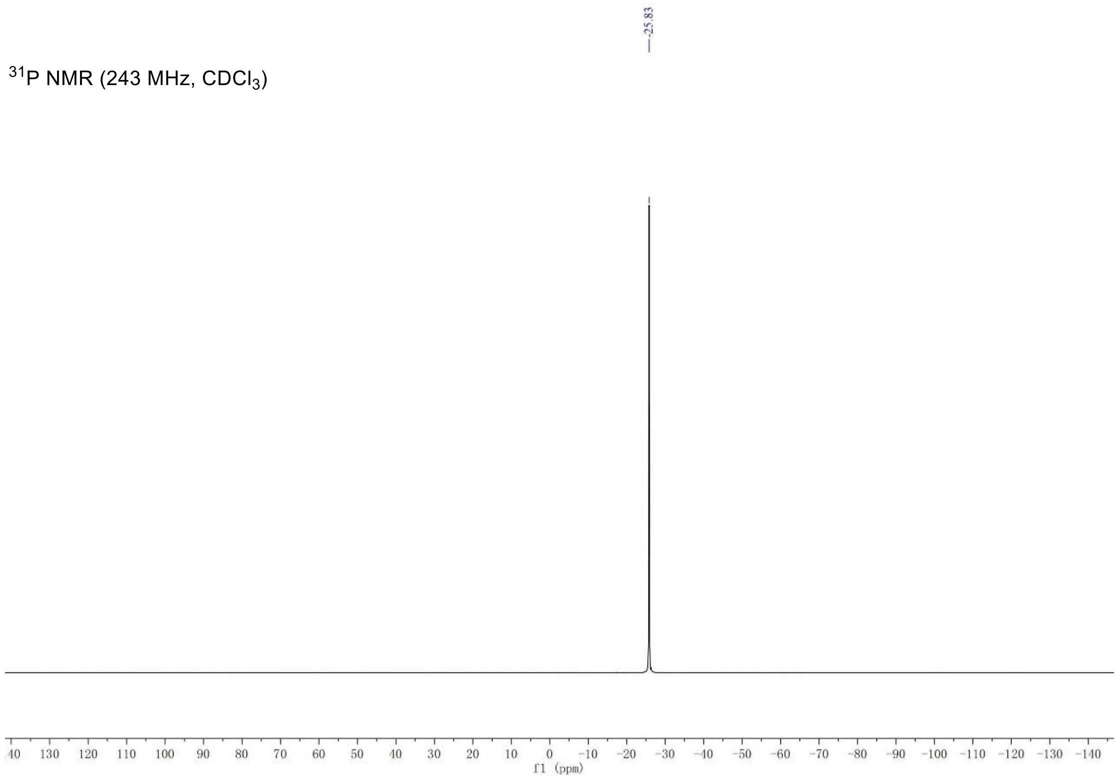

Supplementary Figure 9. ^{31}P NMR spectra of (R_C,S_P,R_C)-**L3**

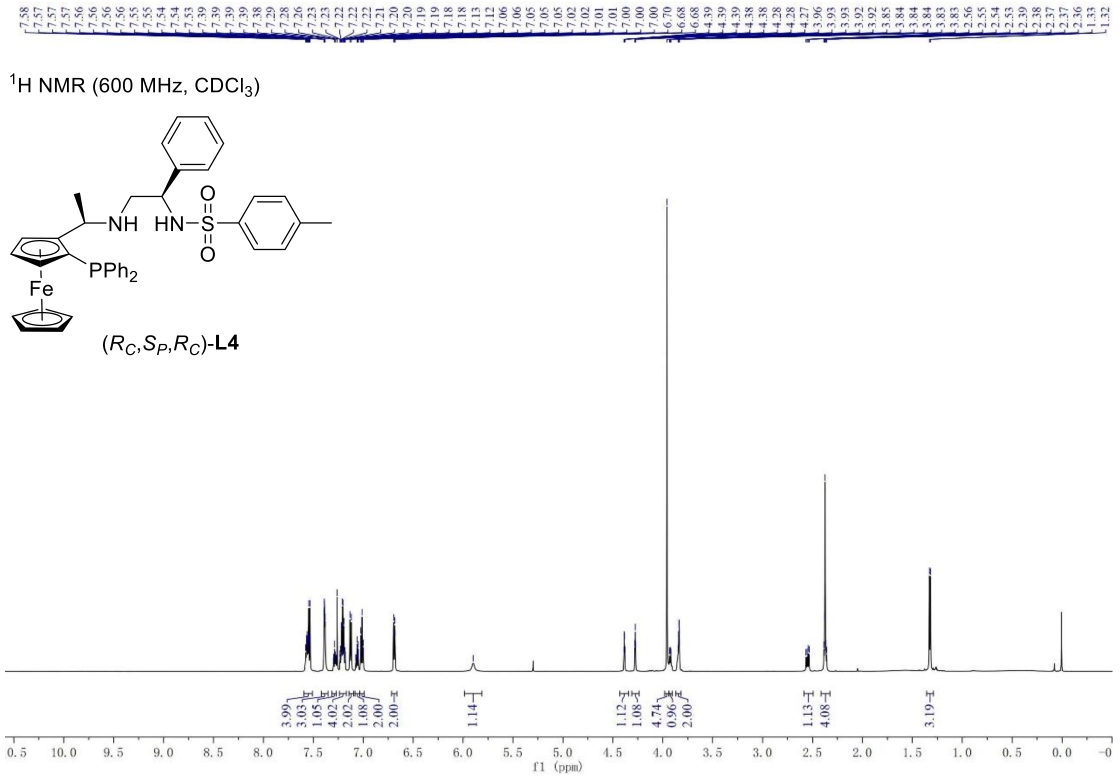

Supplementary Figure 10. ^{1}H NMR spectra of (R_C,S_P,R_C)-**L4**

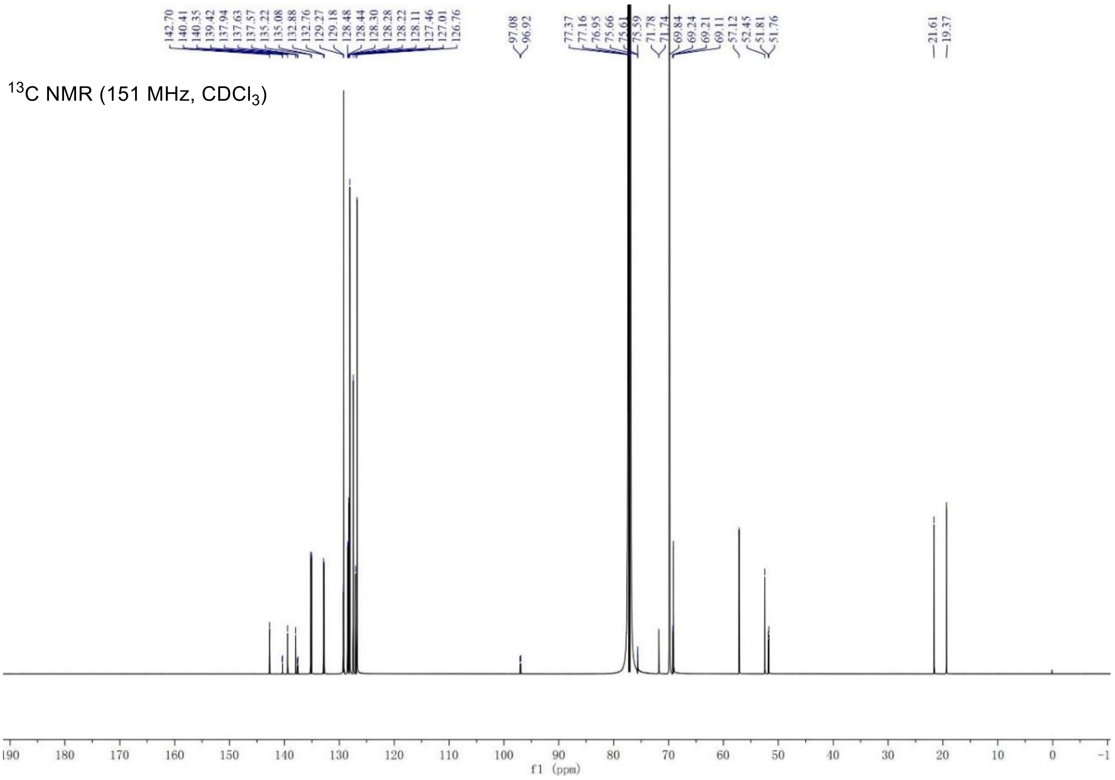

Supplementary Figure 11. ^{13}C NMR spectra of (R_C,S_P,R_C)-**L4**

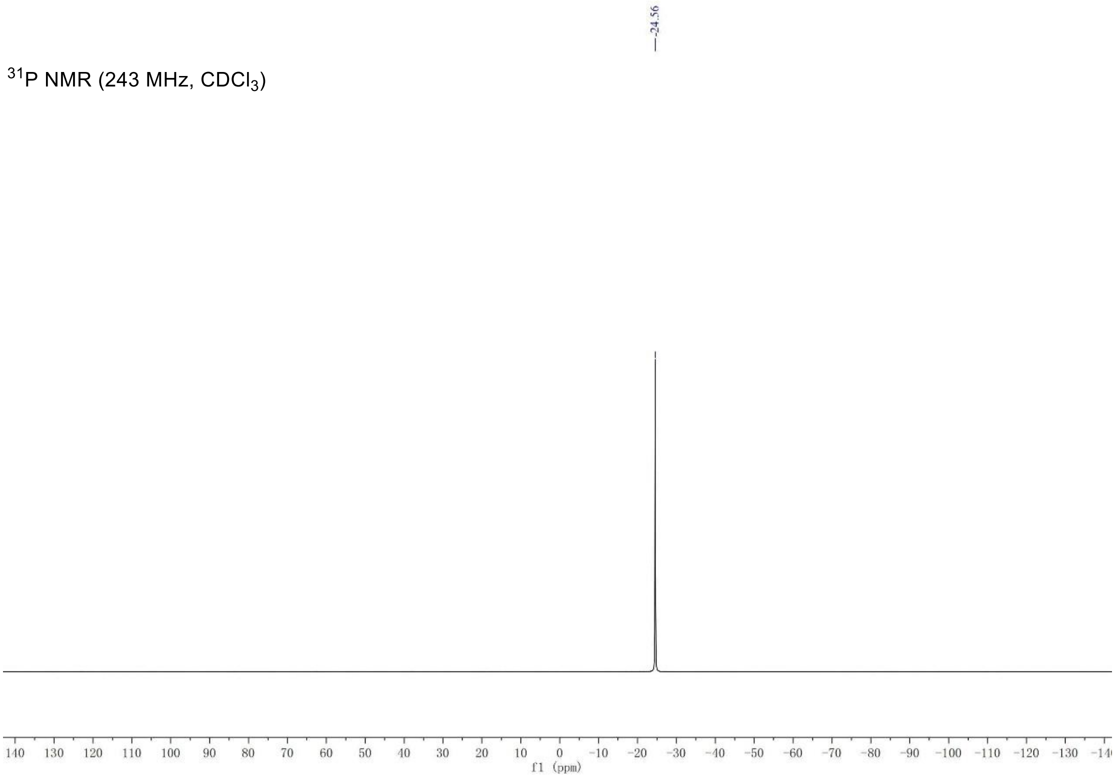

Supplementary Figure 12. ^{31}P NMR spectra of (R_C,S_P,R_C)-**L4**

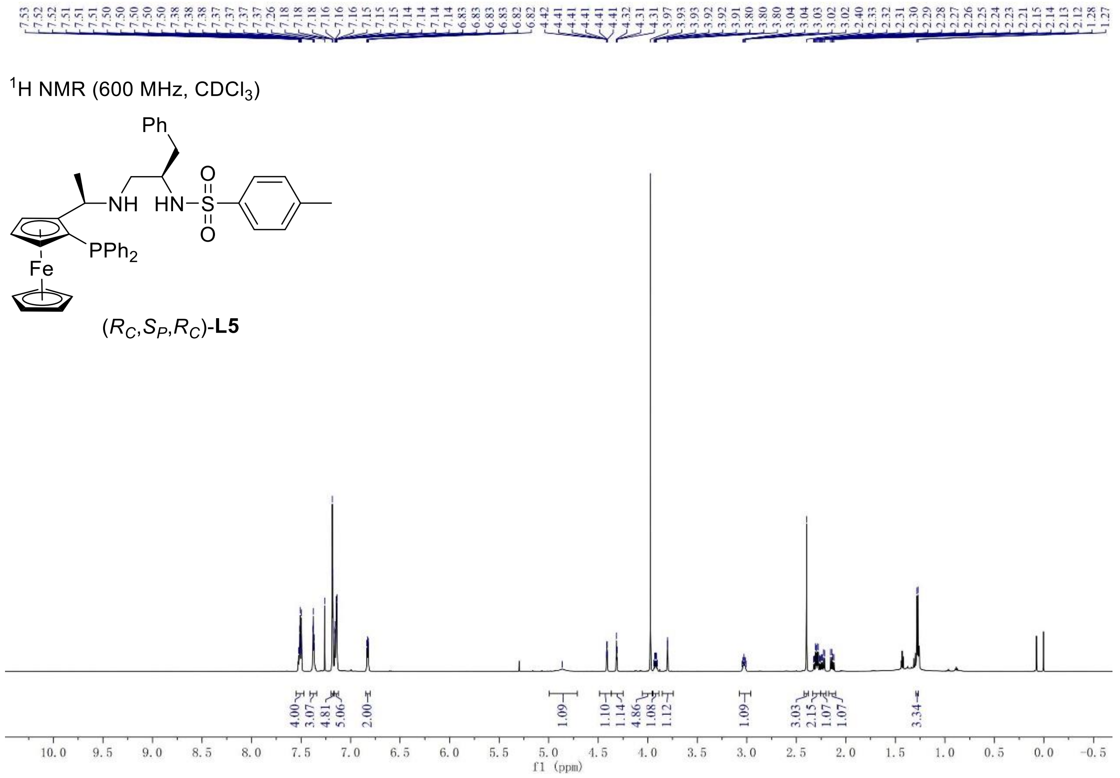

Supplementary Figure 13. ^{1}H NMR spectra of (R_C,S_P,R_C)-**L5**

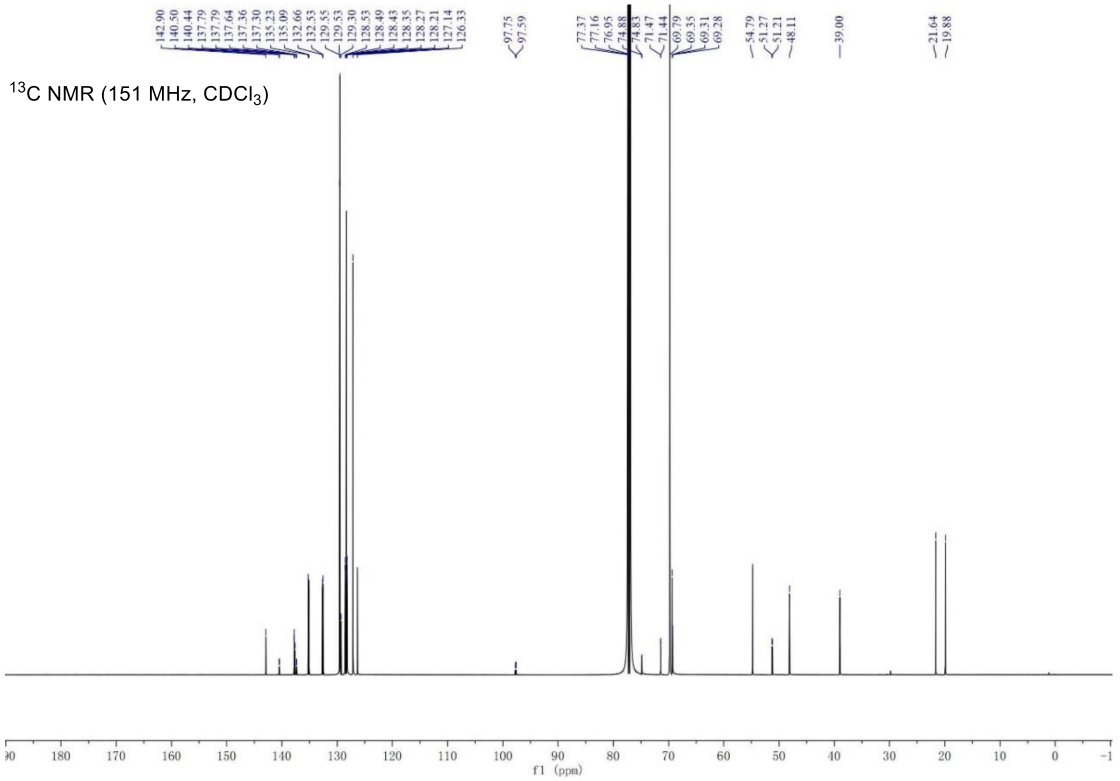

Supplementary Figure 14. ^{13}C NMR spectra of (R_C,S_P,R_C)-**L5**

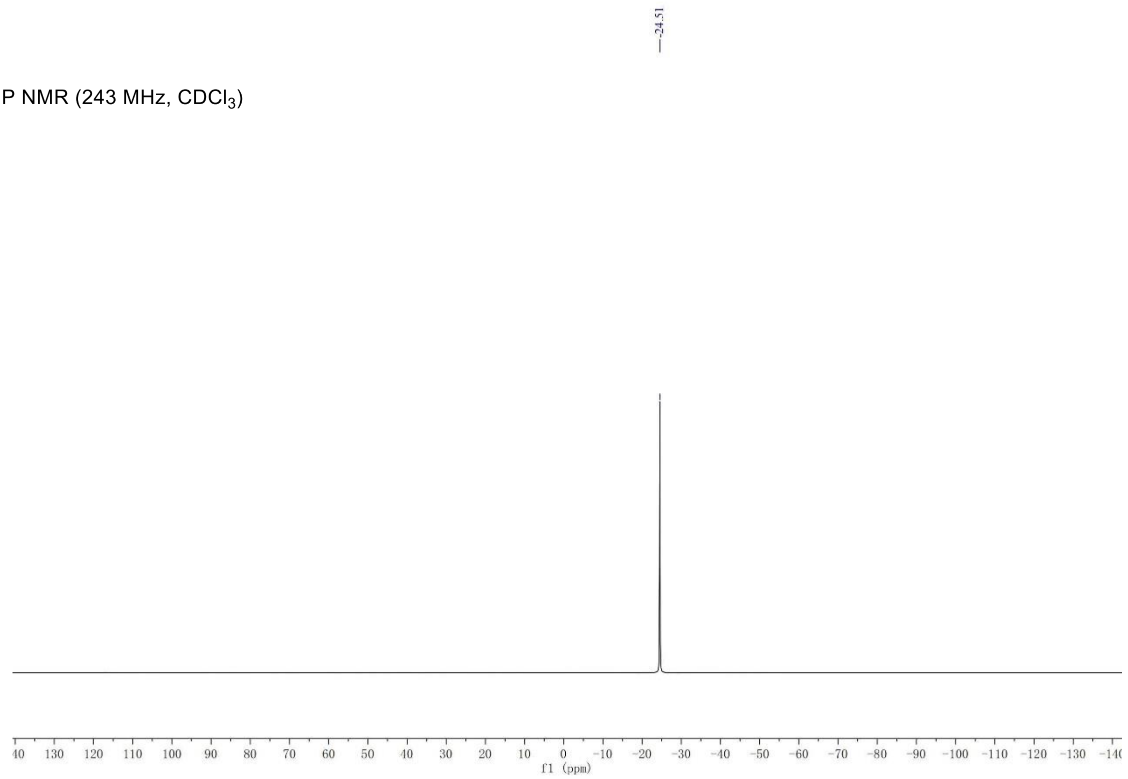

Supplementary Figure 15. ^{31}P NMR spectra of (R_C,S_P,R_C)-**L5**

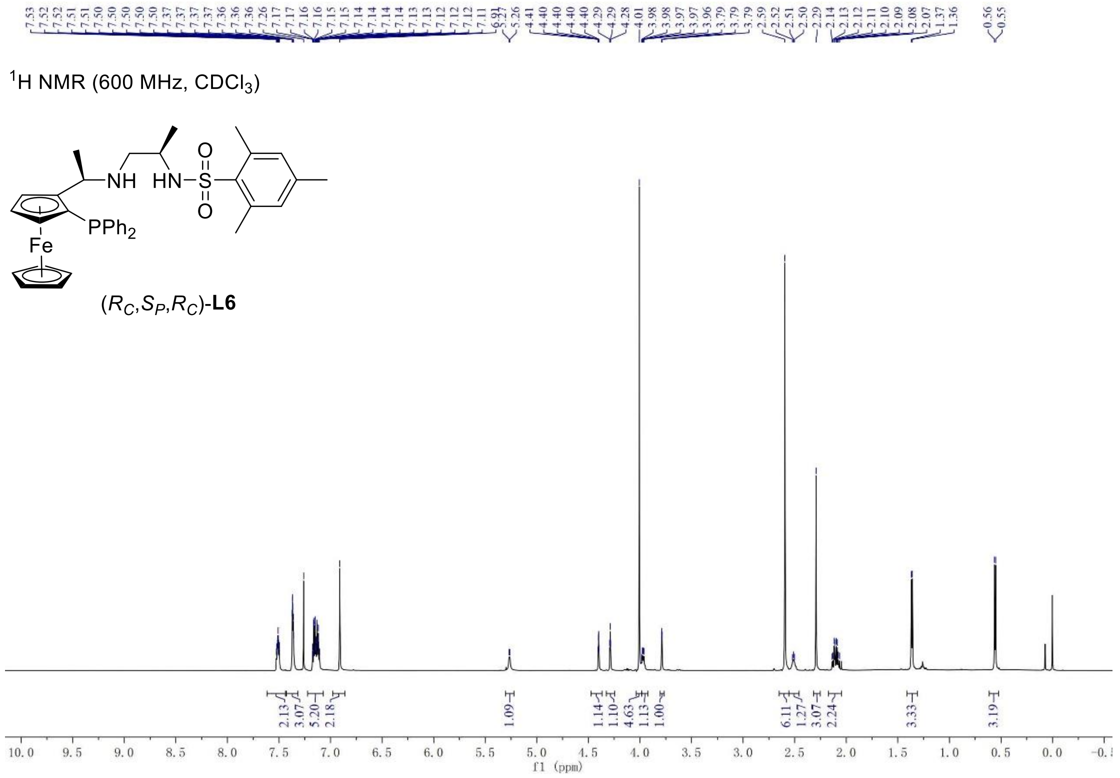

Supplementary Figure 16. ^{1}H NMR spectra of (R_C,S_P,R_C)-**L6**

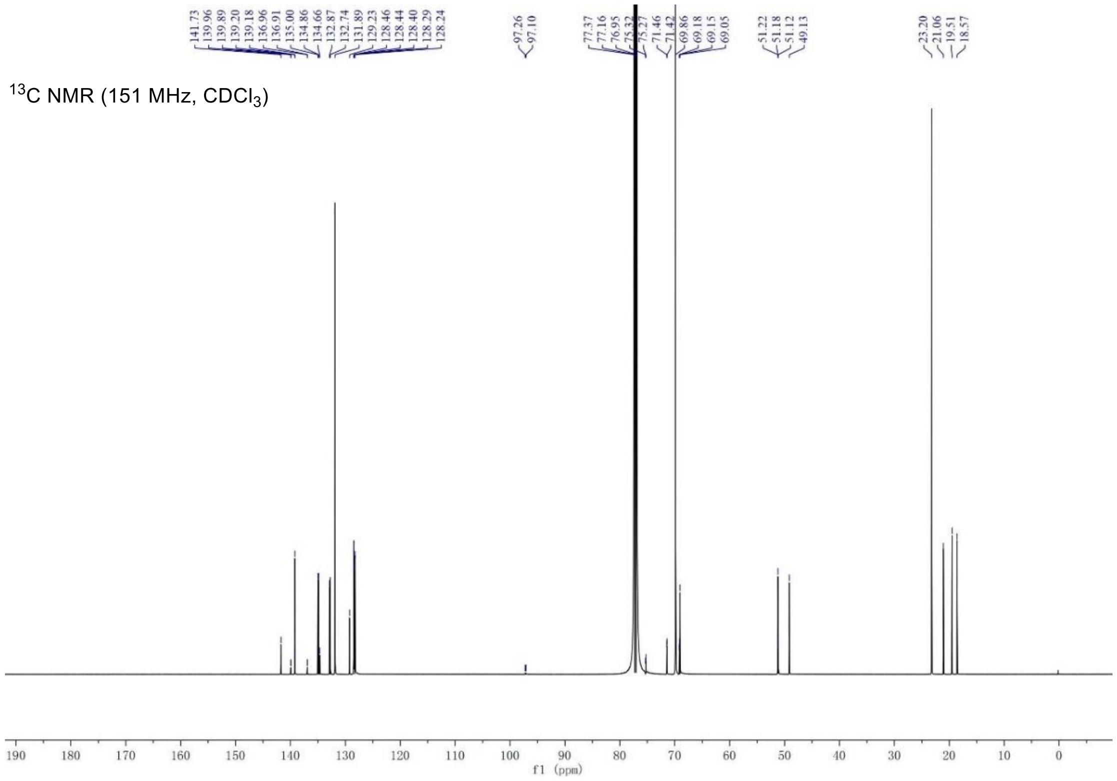

Supplementary Figure 17. ^{13}C NMR spectra of (R_C,S_P,R_C)-**L6**

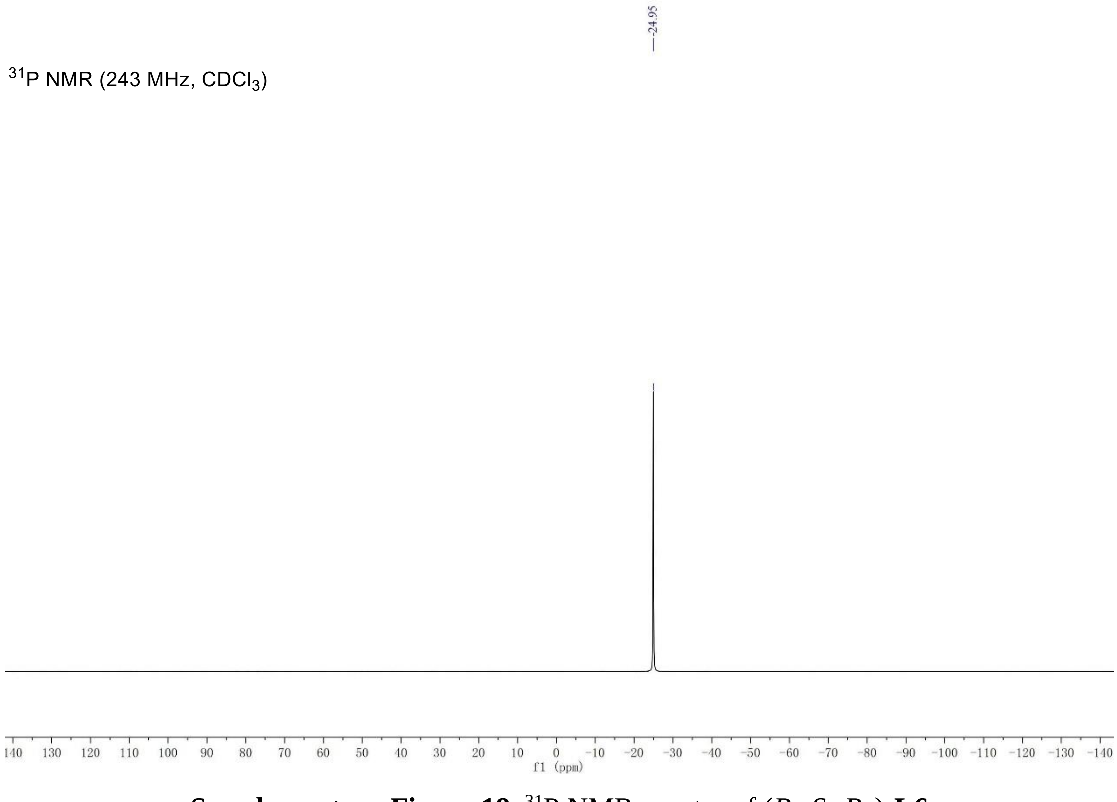

Supplementary Figure 18. ^{31}P NMR spectra of (R_C,S_P,R_C)-**L6**

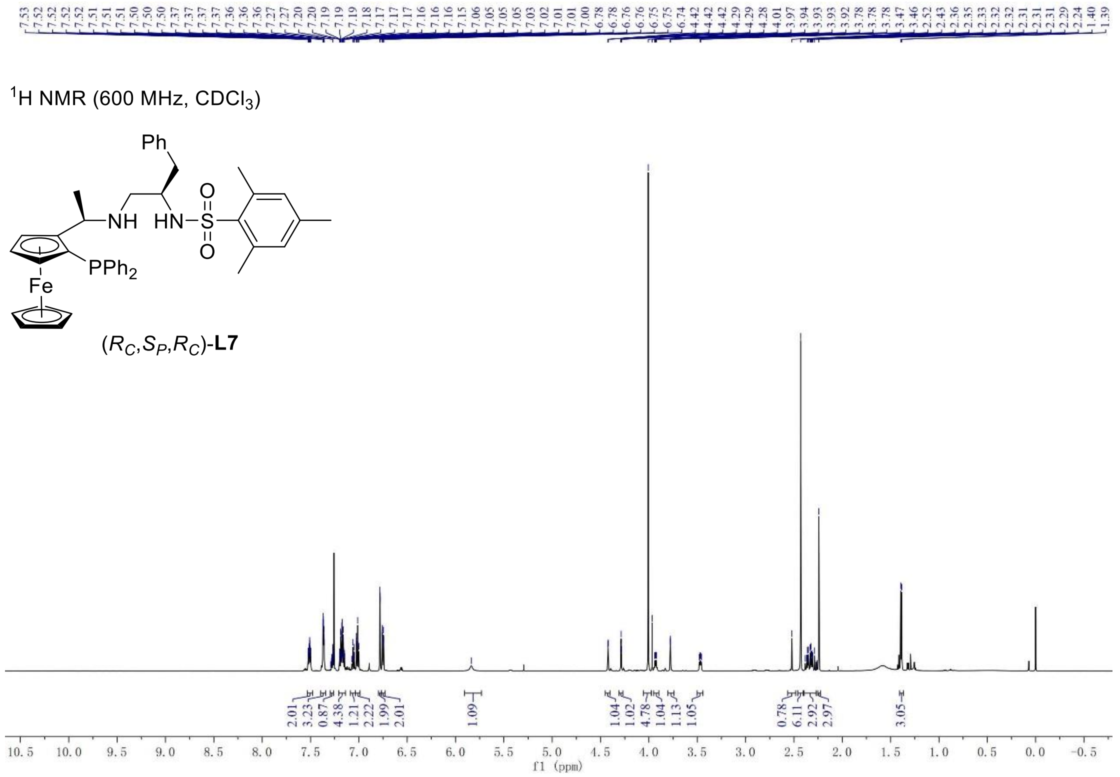

Supplementary Figure 19. ^{1}H NMR spectra of (R_C,S_P,R_C)-**L7**

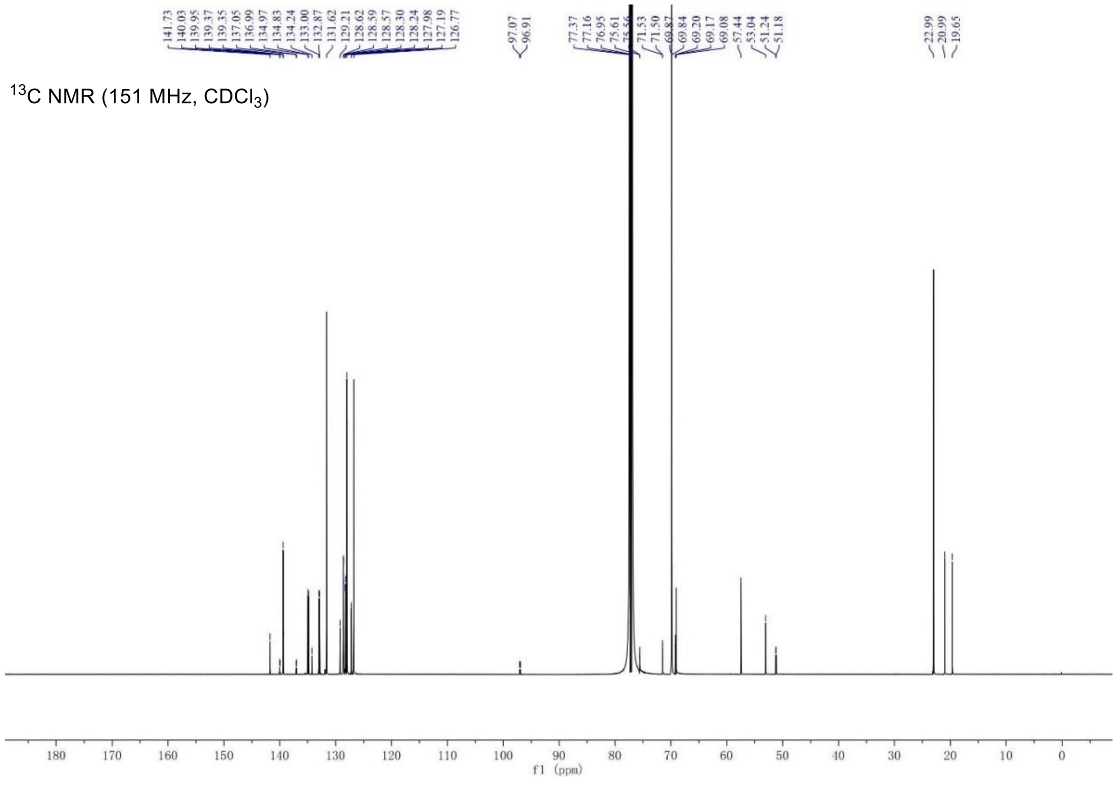

Supplementary Figure 20. ^{13}C NMR spectra of (R_C,S_P,R_C)-**L7**

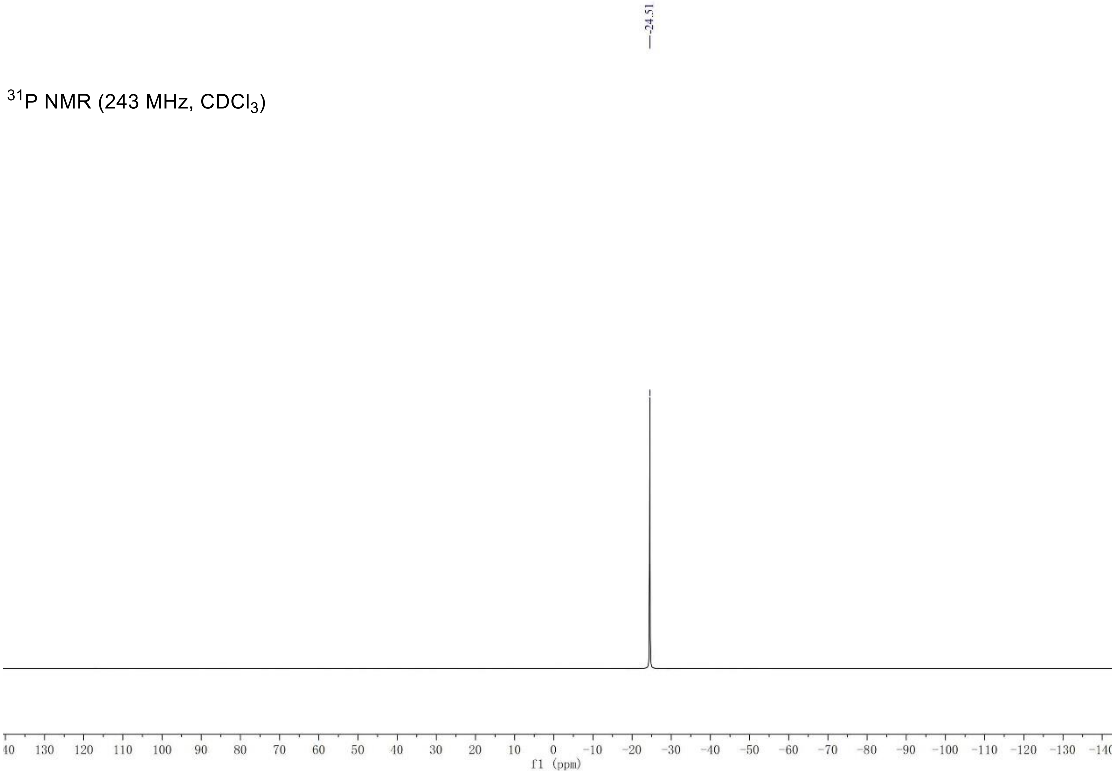

Supplementary Figure 21. ^{31}P NMR spectra of (R_C,S_P,R_C)-**L7**

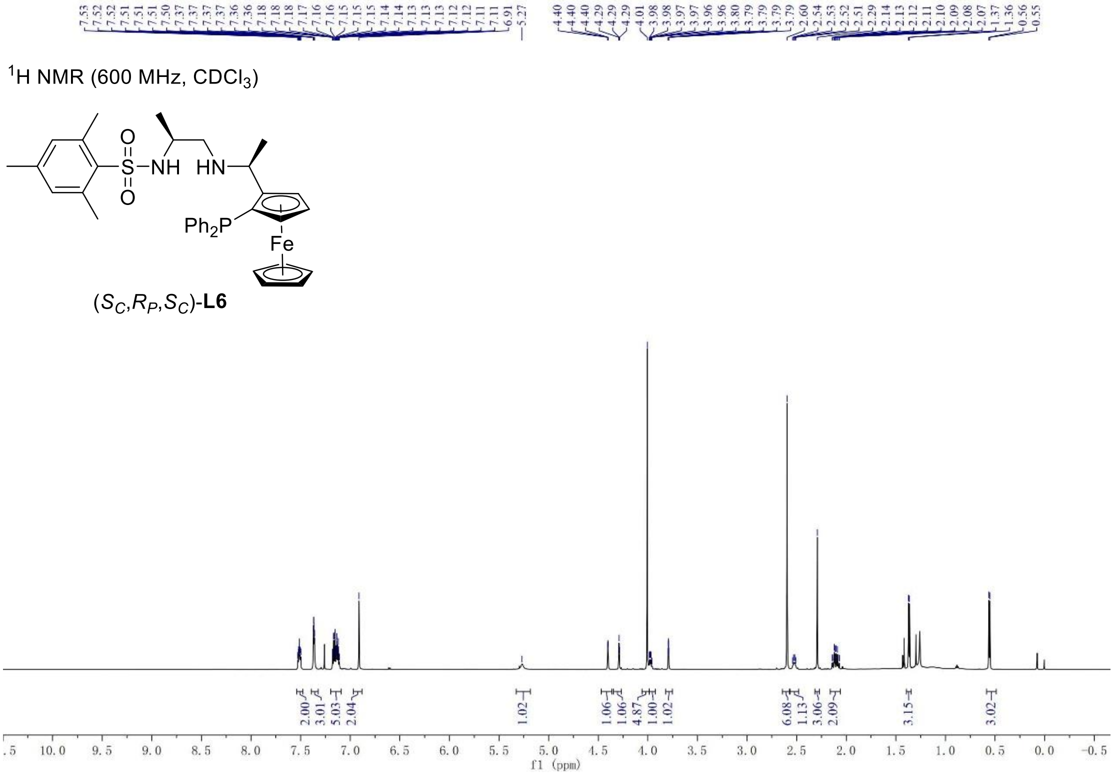

Supplementary Figure 22. ^{1}H NMR spectra of (S_C,R_P,S_C)-**L6**

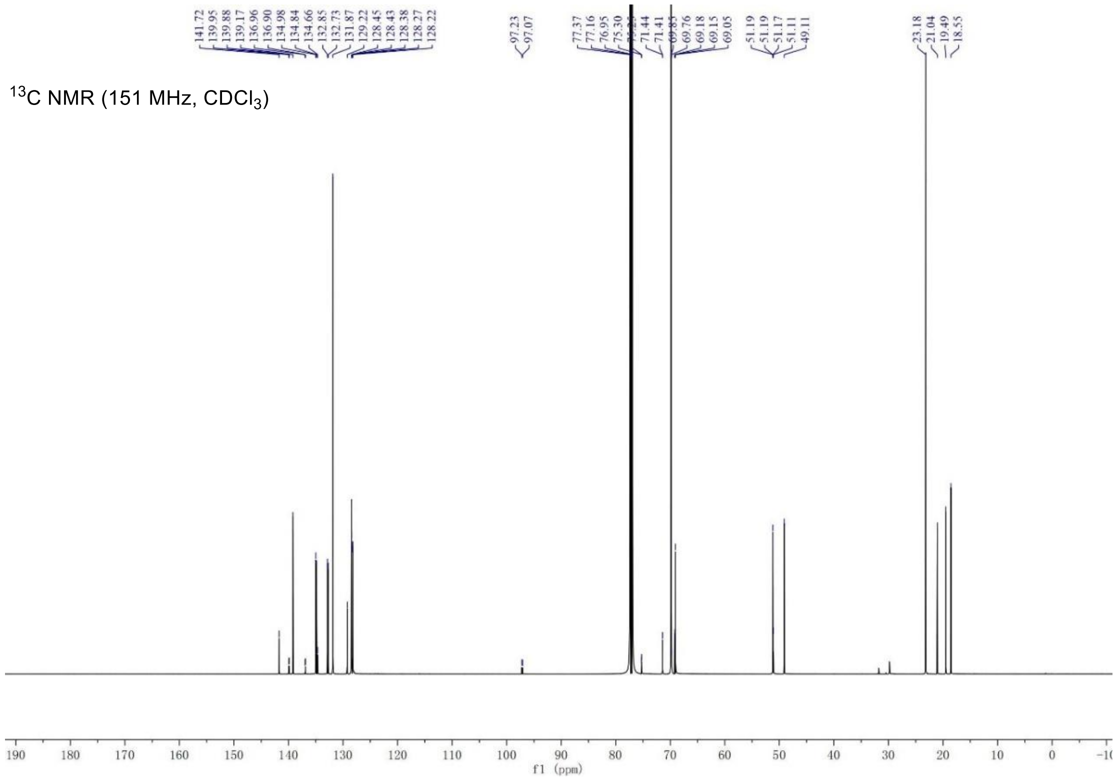

Supplementary Figure 23. ^{13}C NMR spectra of (S_C,R_P,S_C)-**L6**

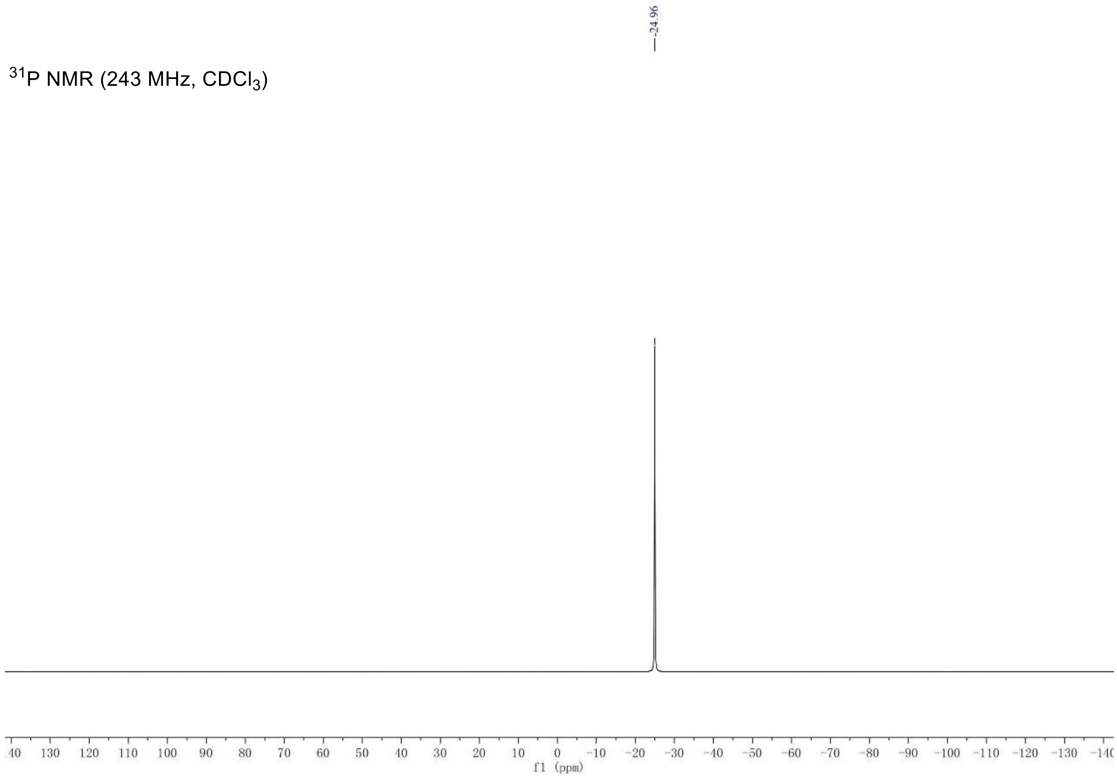

Supplementary Figure 24. ^{31}P NMR spectra of (S_C,R_P,S_C)-**L6**

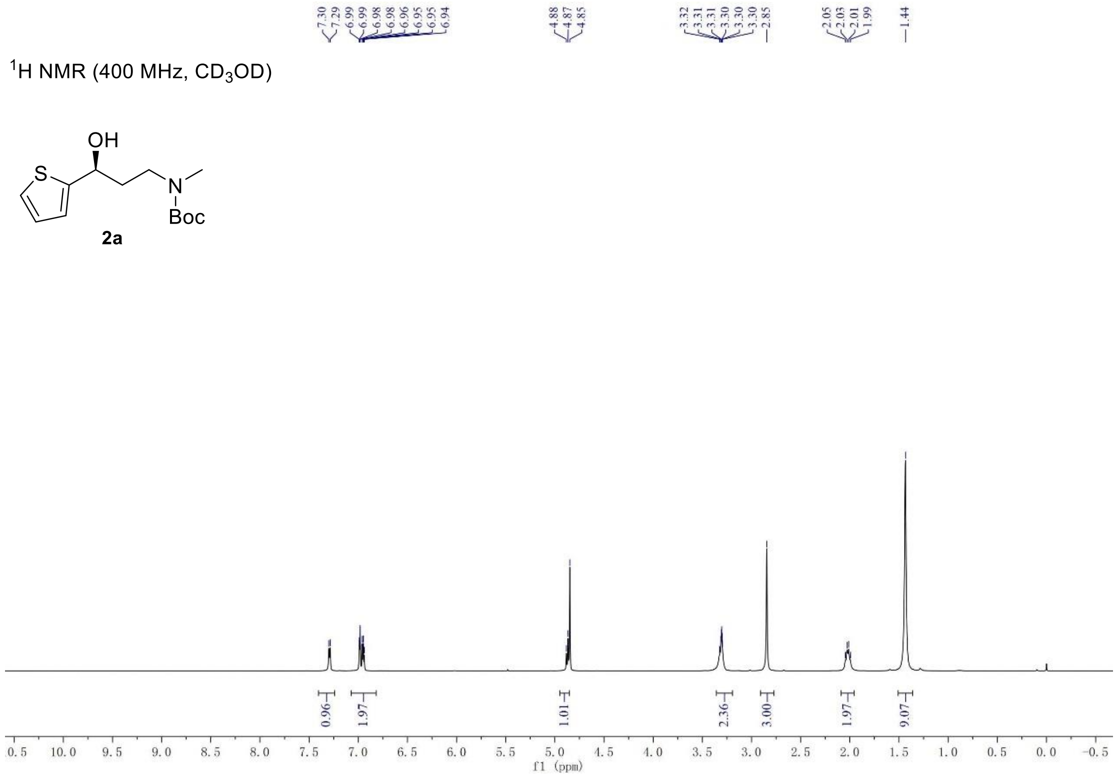

Supplementary Figure 25. ^{1}H NMR spectra of **2a**

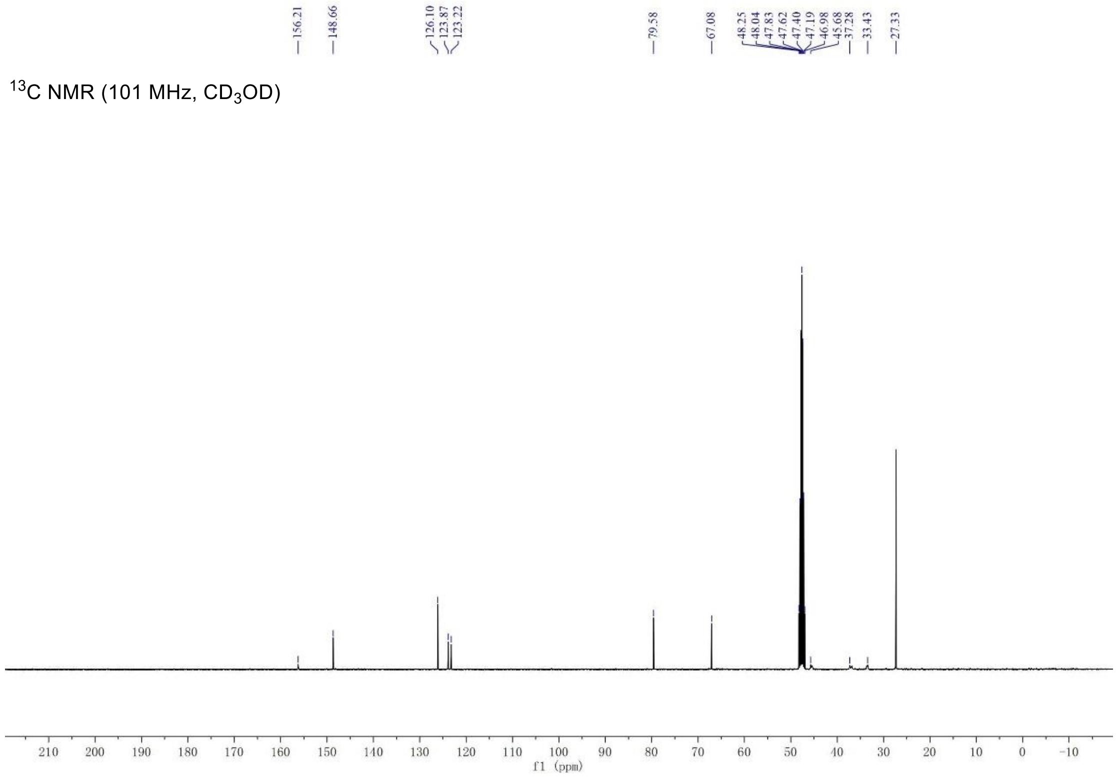

Supplementary Figure 26. ^{13}C NMR spectra of **2a**

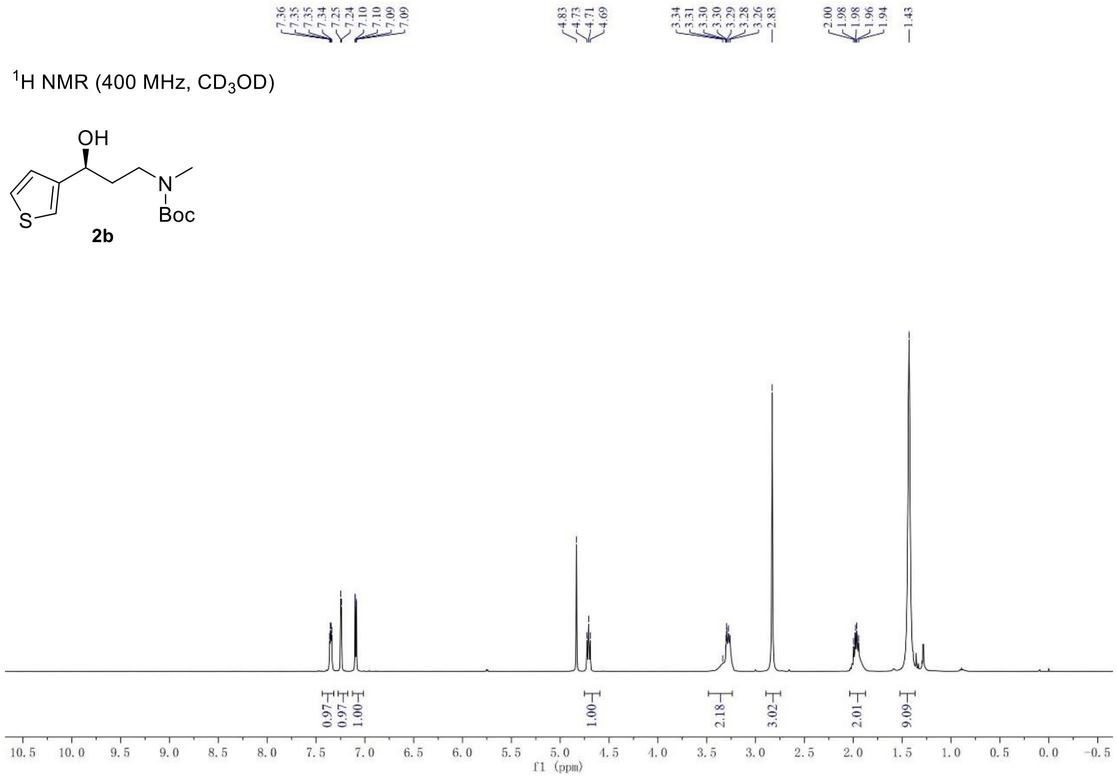

Supplementary Figure 27. ^{1}H NMR spectra of **2b**

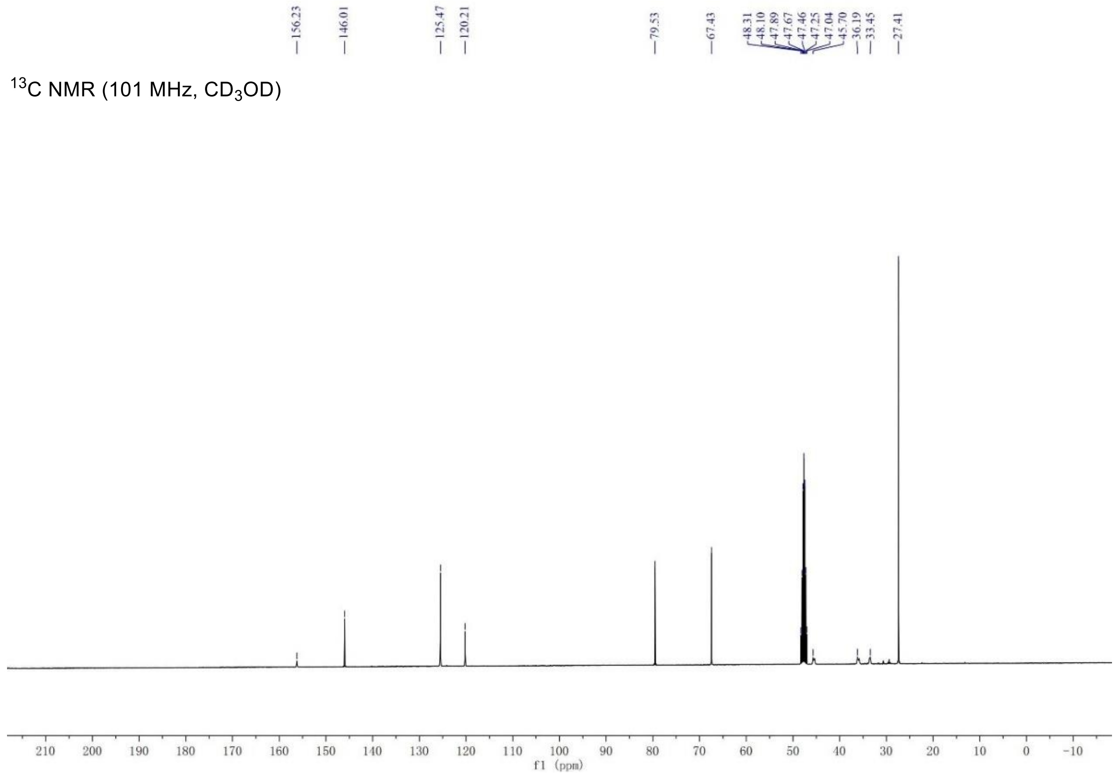

Supplementary Figure 28. ^{13}C NMR spectra of **2b**

^1H NMR (400 MHz, CD_3OD)

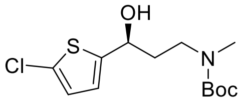

2c

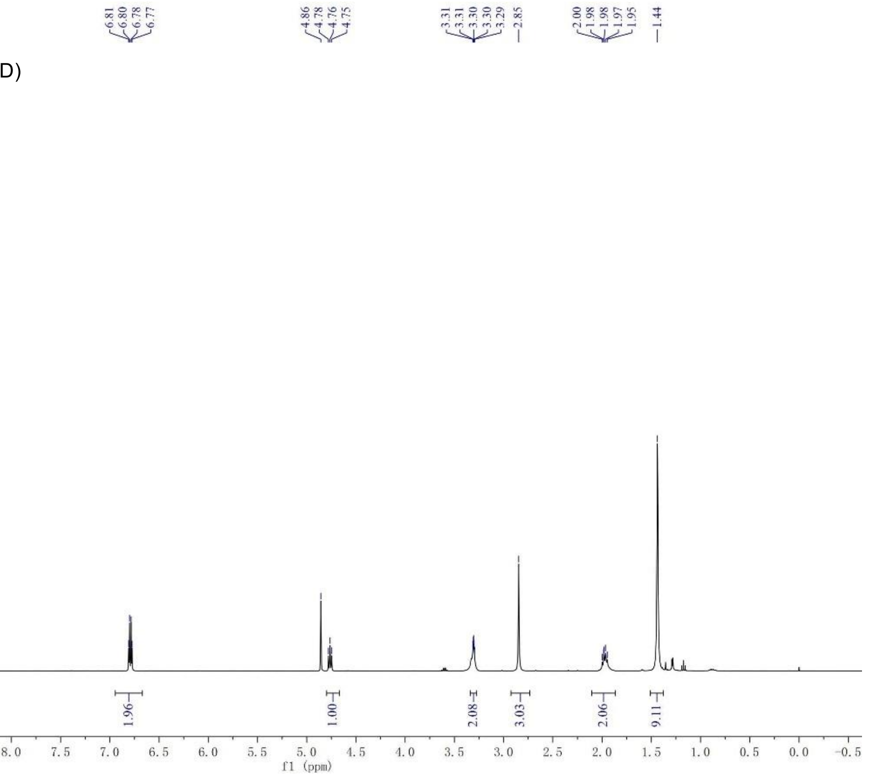

Supplementary Figure 29. ^1H NMR spectra of **2c**

^{13}C NMR (101 MHz, CD_3OD)

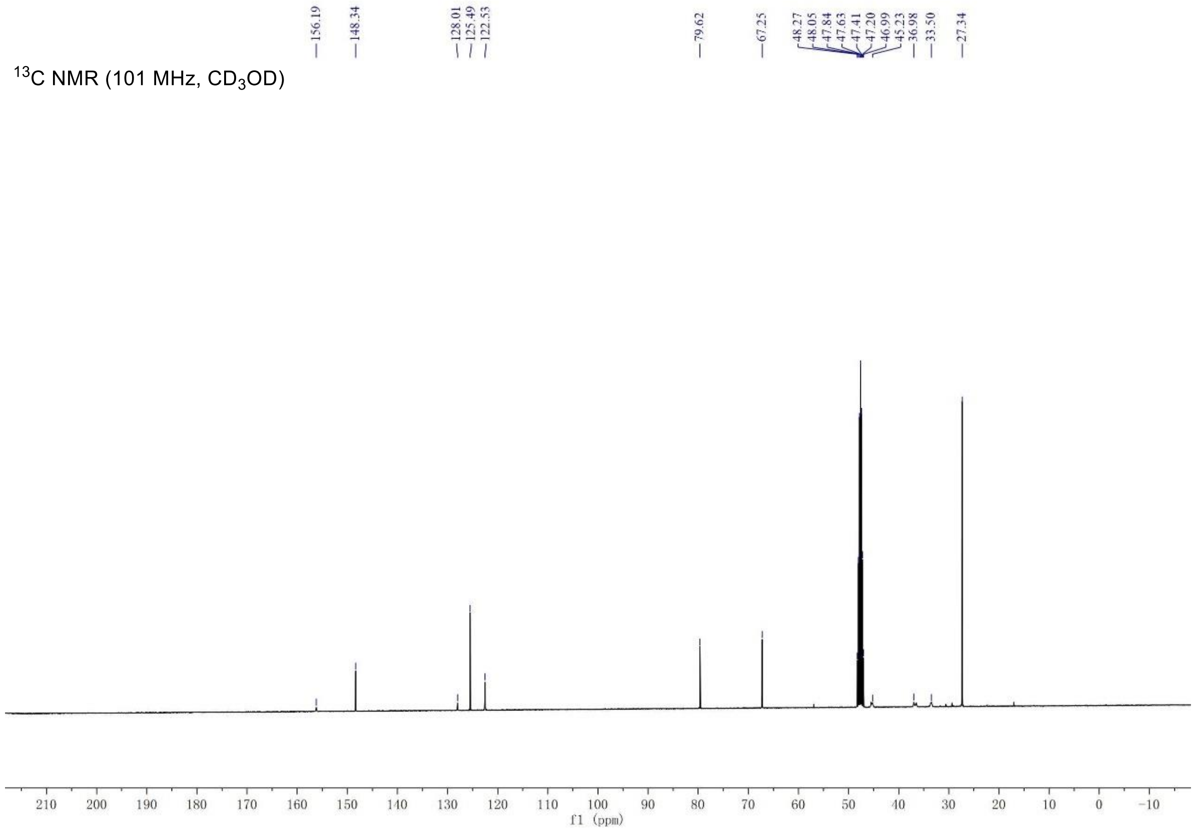

Supplementary Figure 30. ^{13}C NMR spectra of **2c**

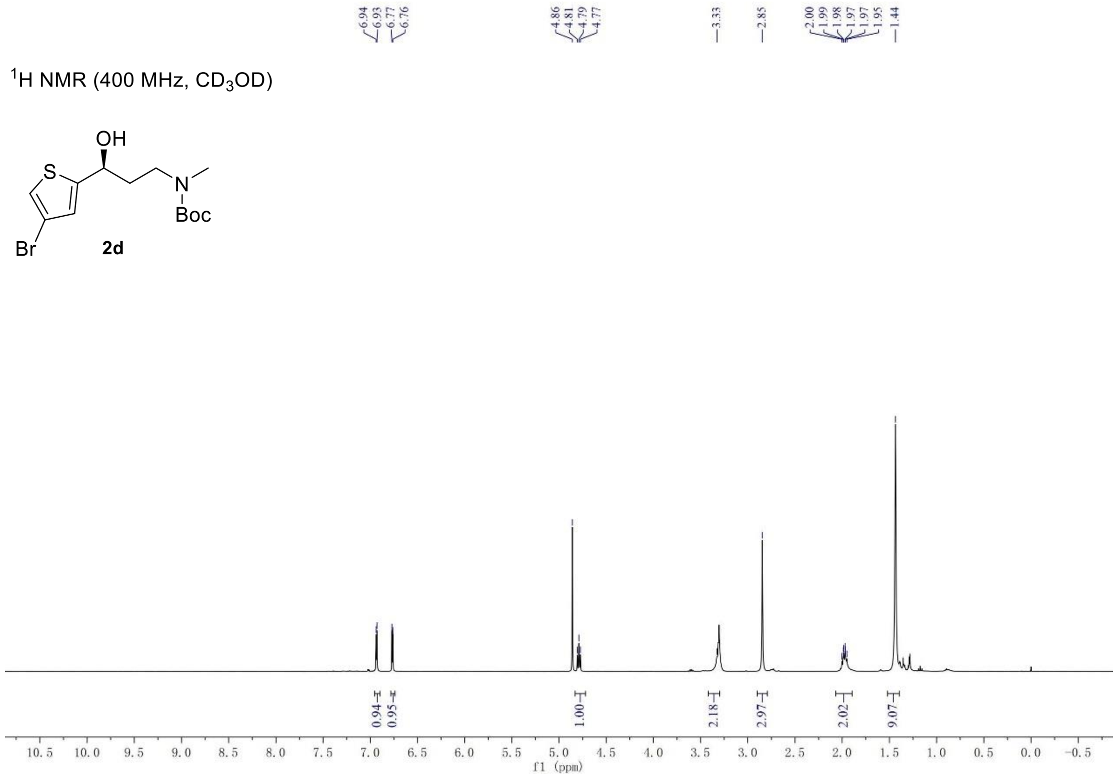

Supplementary Figure 31. ^{1}H NMR spectra of **2d**

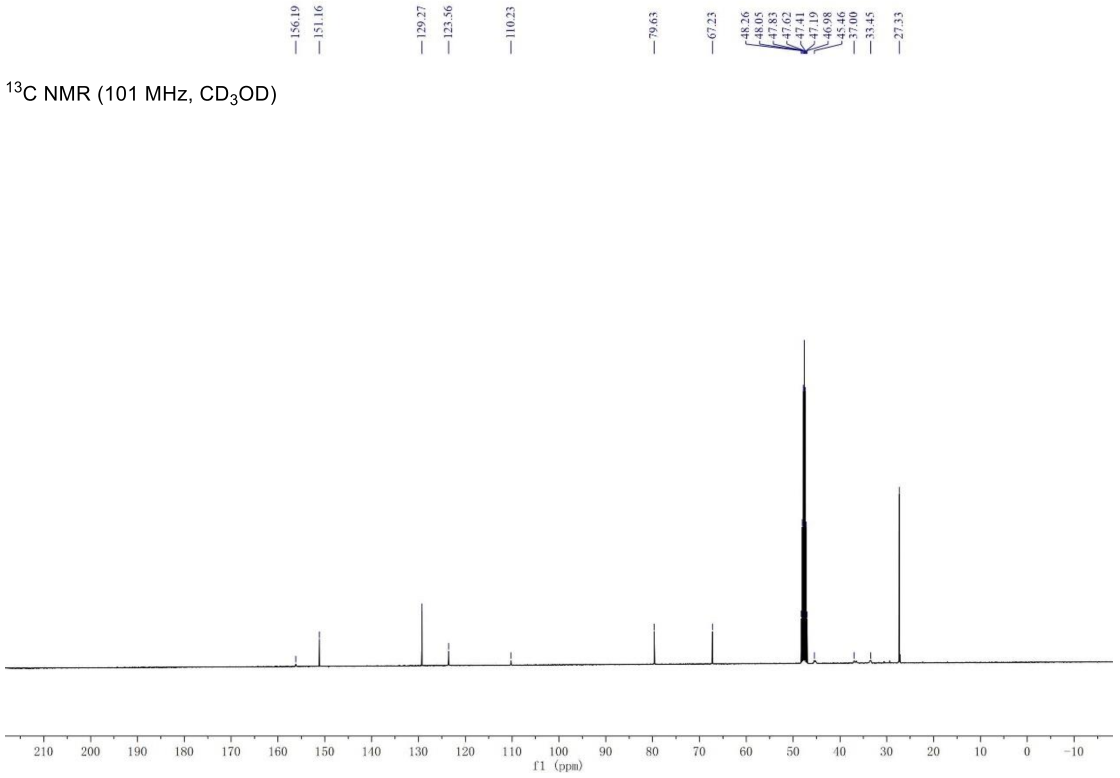

Supplementary Figure 32. ^{13}C NMR spectra of **2d**

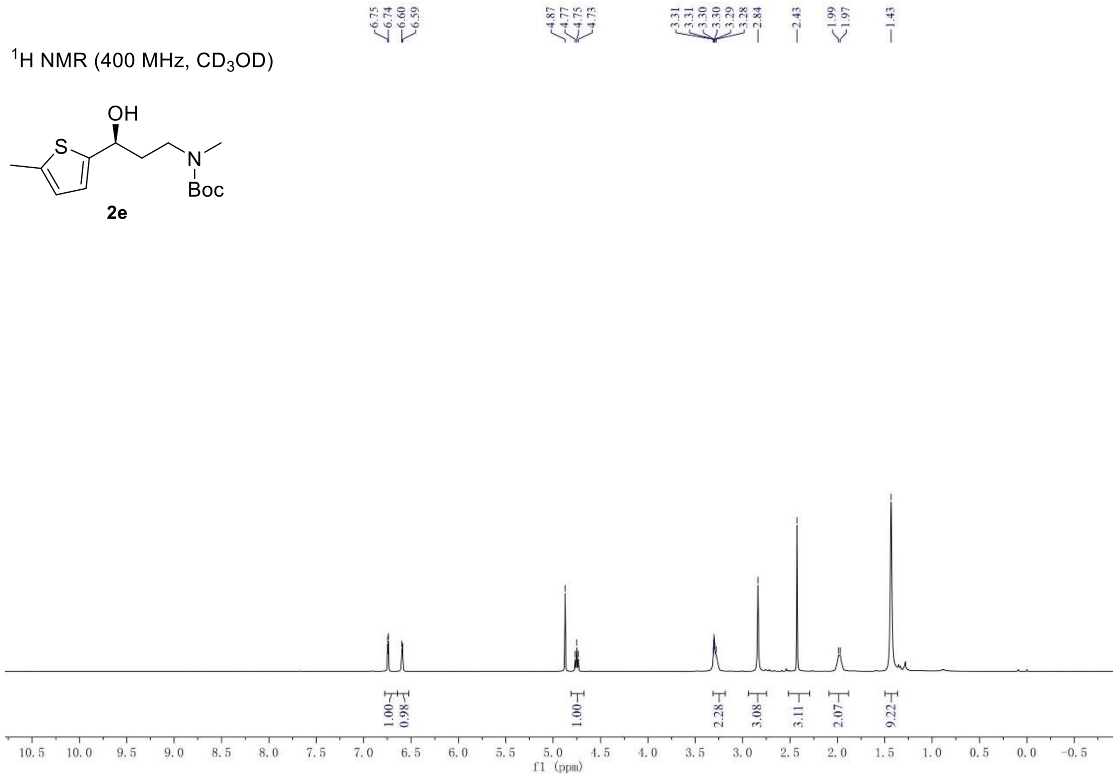

Supplementary Figure 33. ^{1}H NMR spectra of **2e**

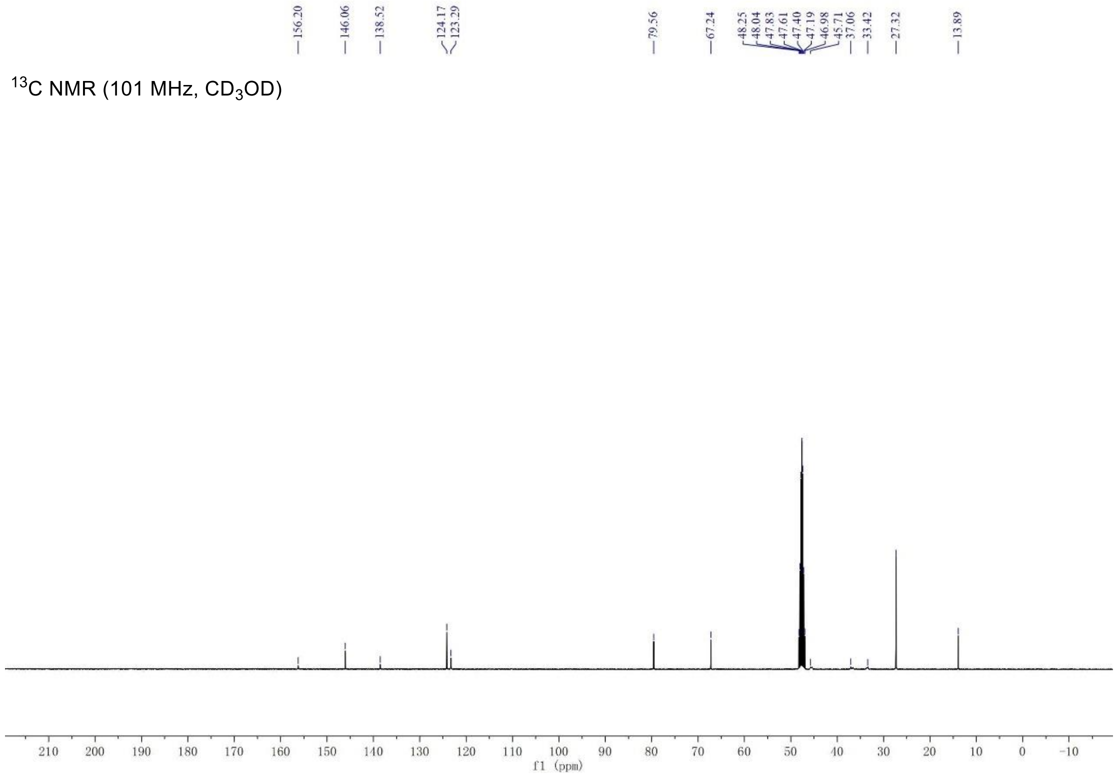

Supplementary Figure 34. ^{13}C NMR spectra of **2e**

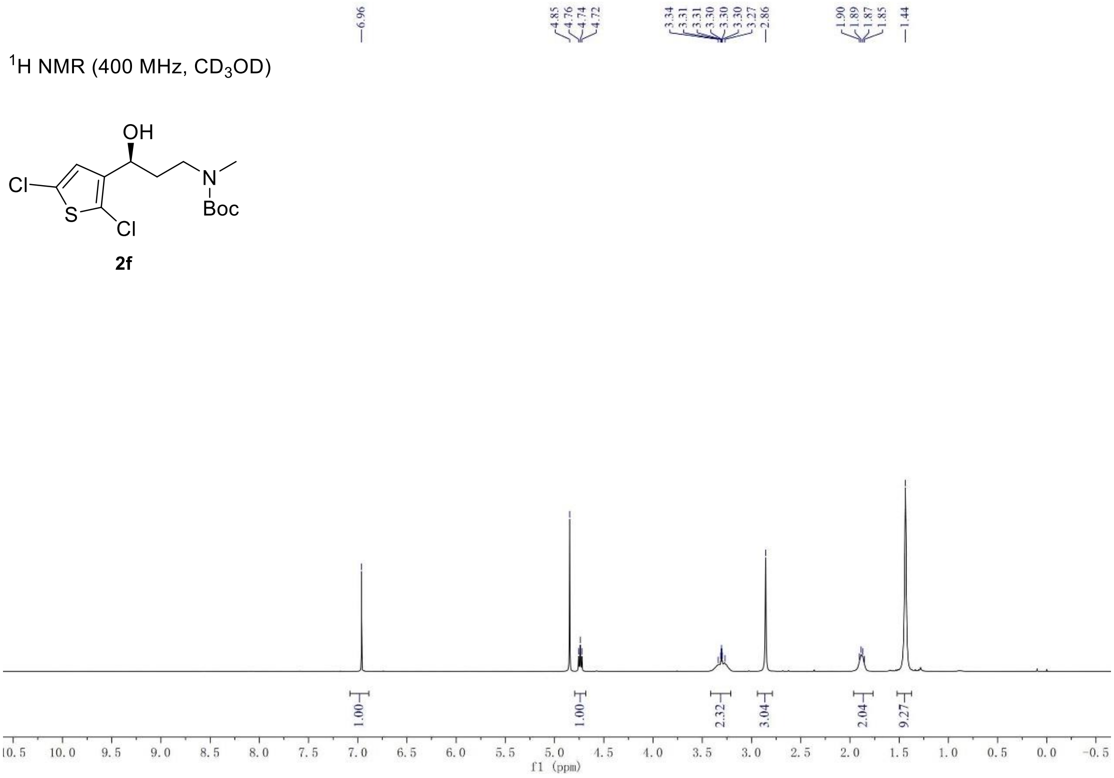

Supplementary Figure 35. ^{1}H NMR spectra of **2f**

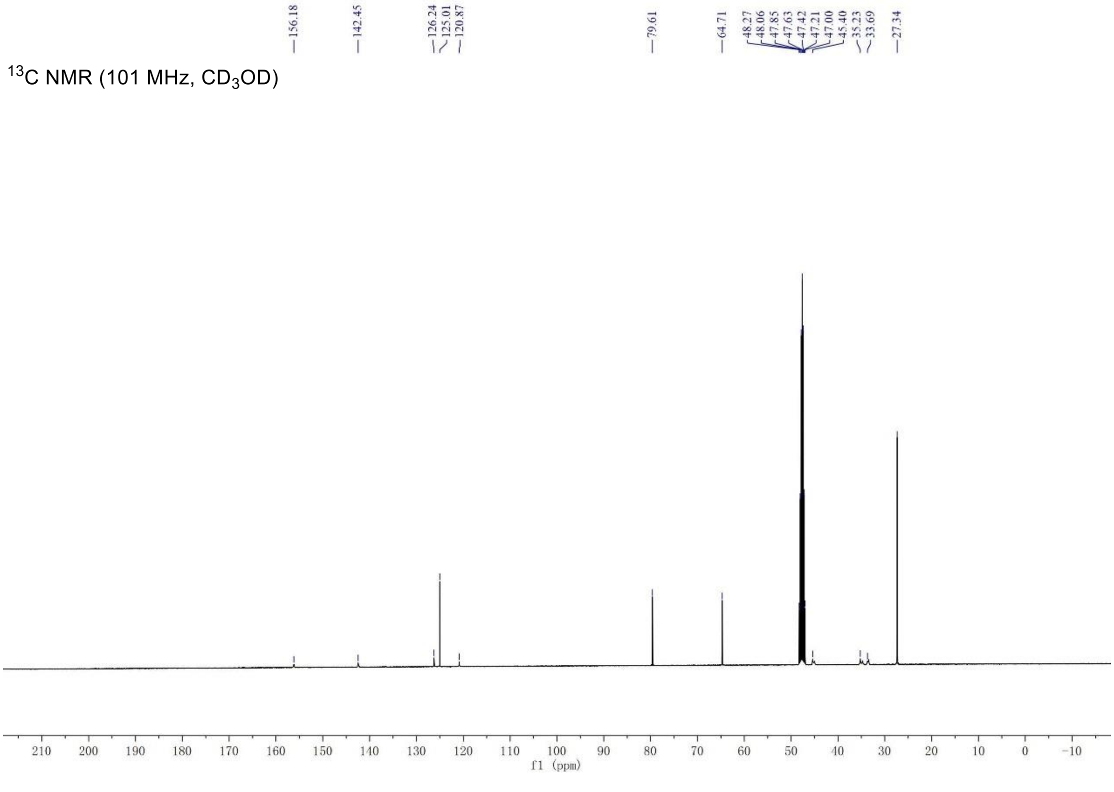

Supplementary Figure 36. ^{13}C NMR spectra of **2f**

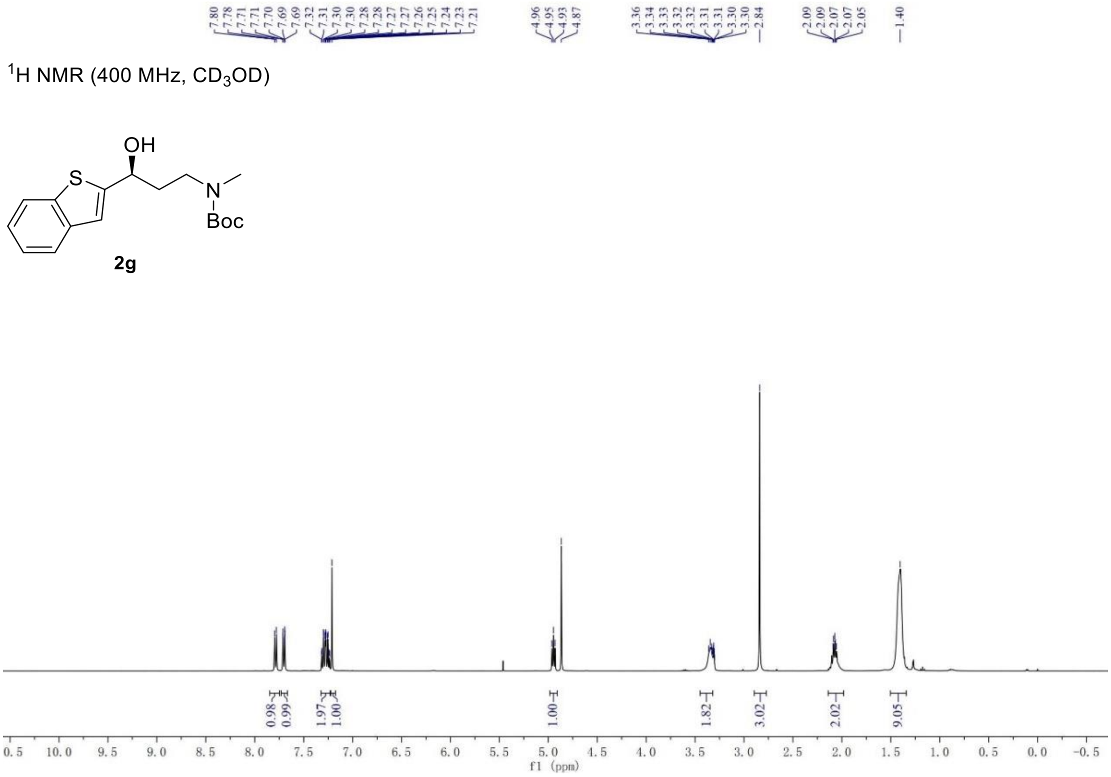

Supplementary Figure 37. ^{1}H NMR spectra of **2g**

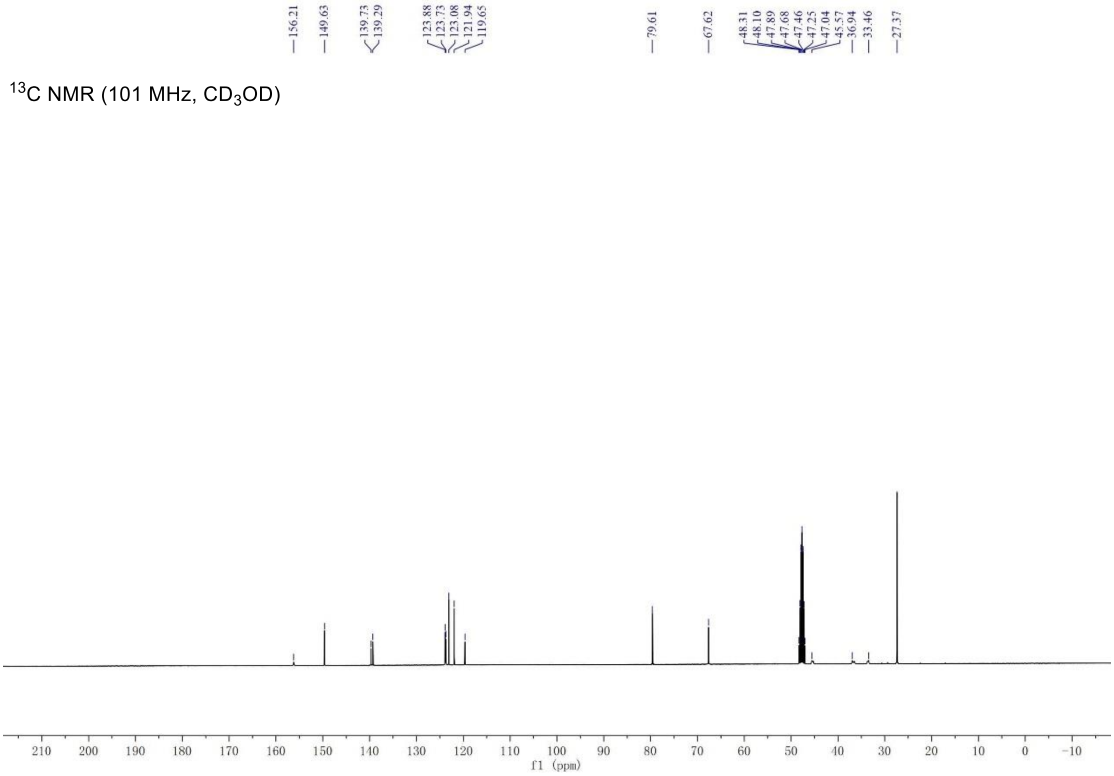

Supplementary Figure 38. ^{13}C NMR spectra of **2g**

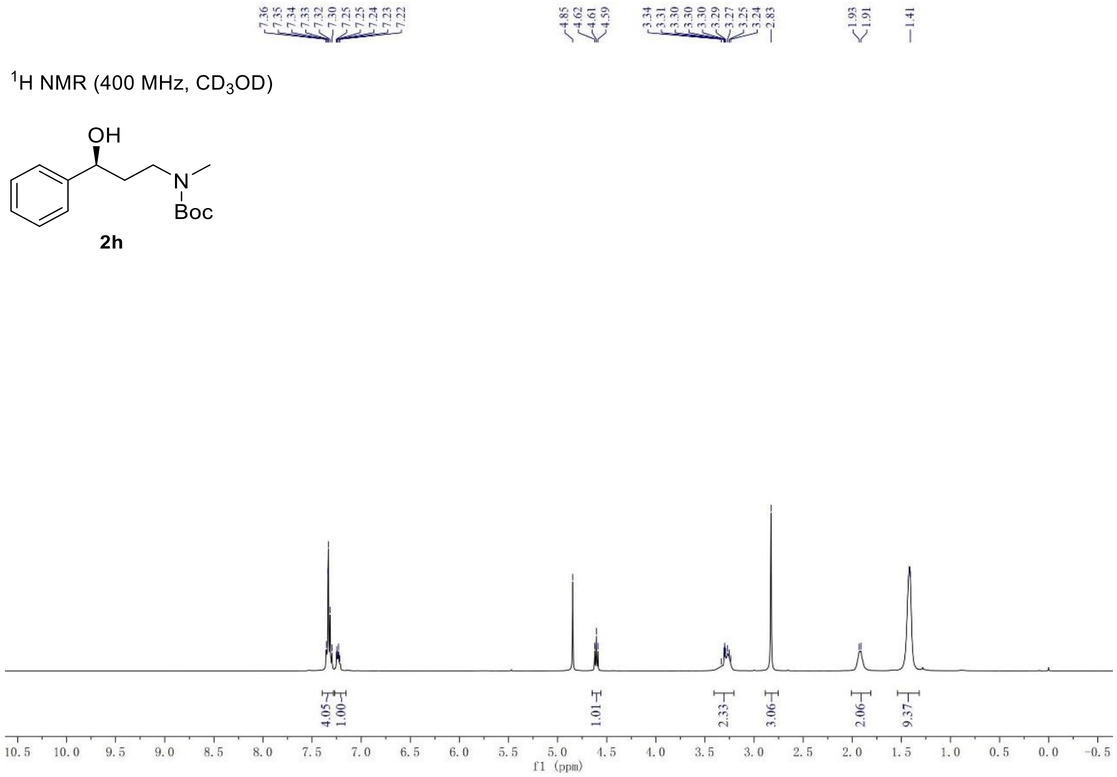

Supplementary Figure 39. ^{1}H NMR spectra of **2h**

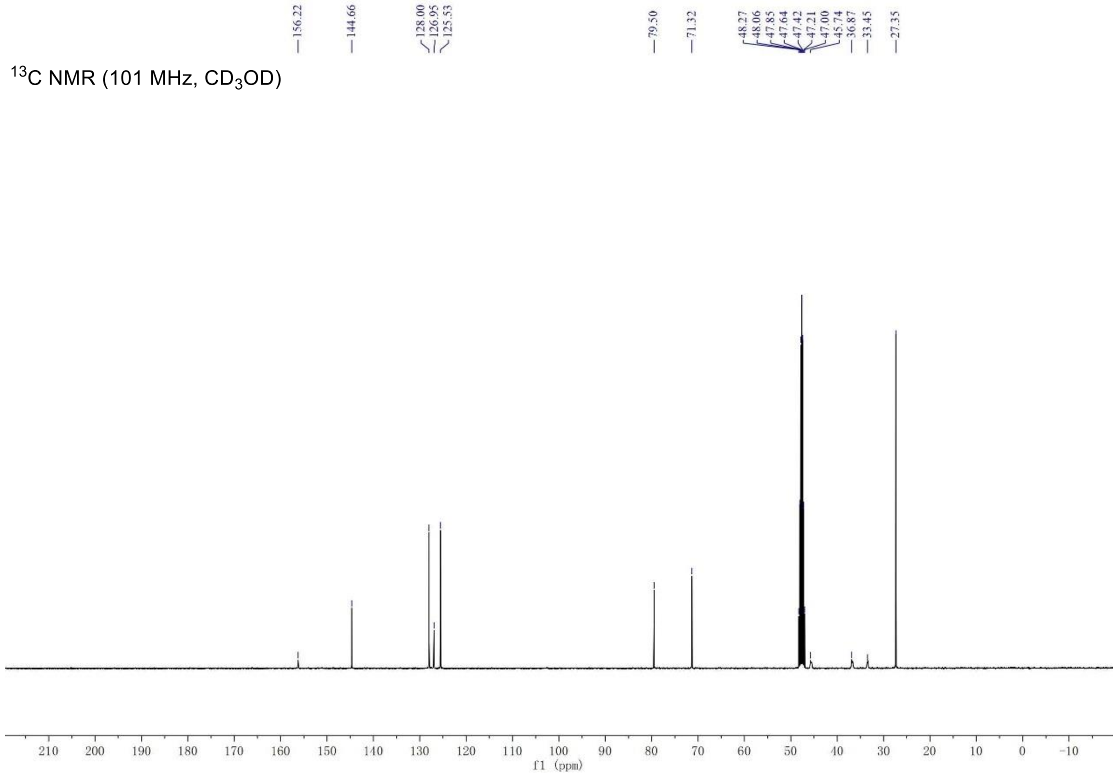

Supplementary Figure 40. ^{13}C NMR spectra of **2h**

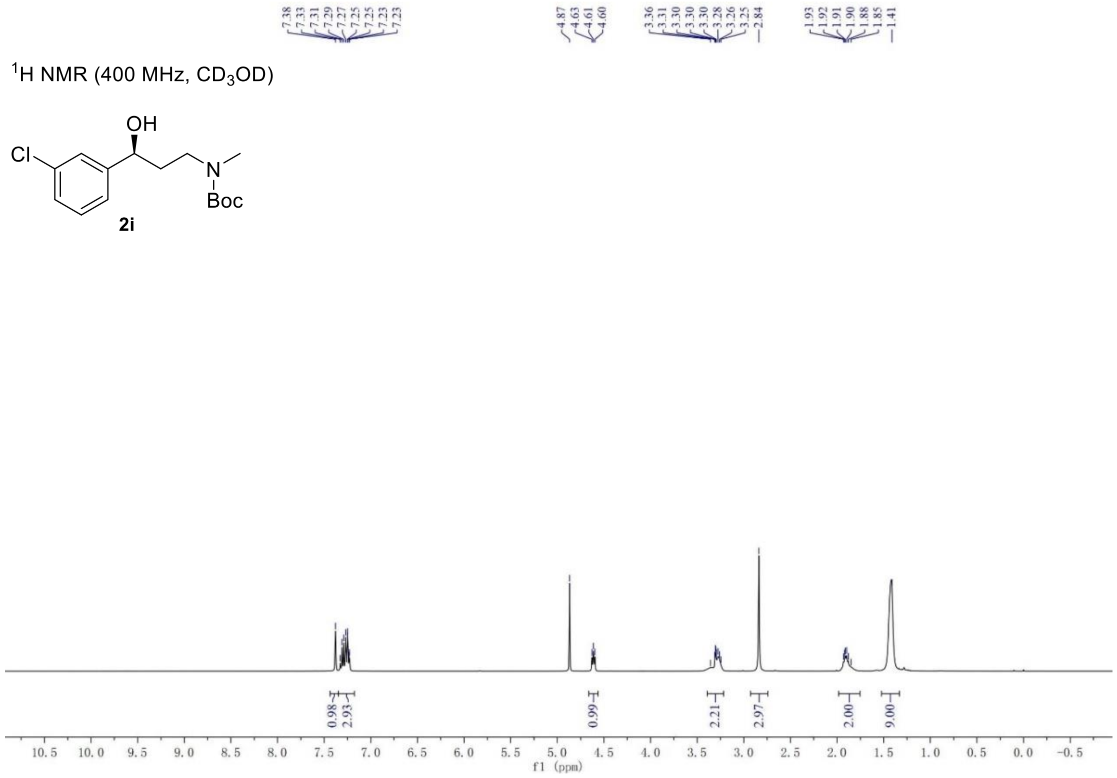

Supplementary Figure 41. ^{1}H NMR spectra of **2i**

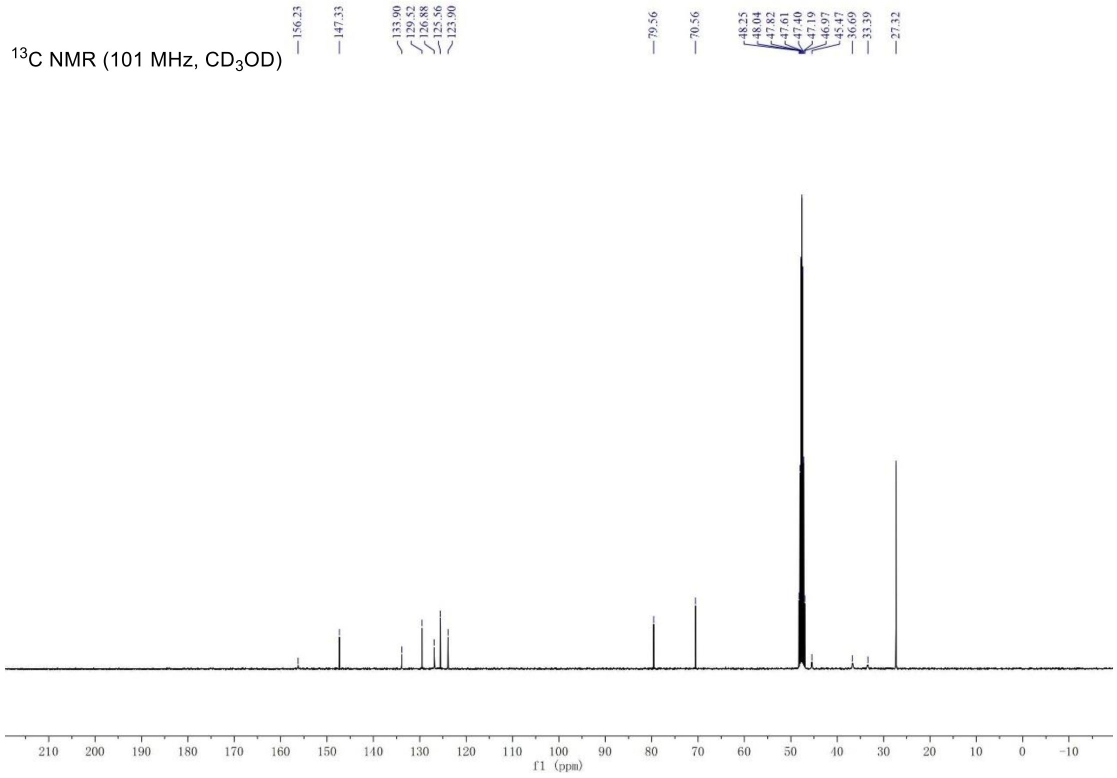

Supplementary Figure 42. ^{13}C NMR spectra of **2i**

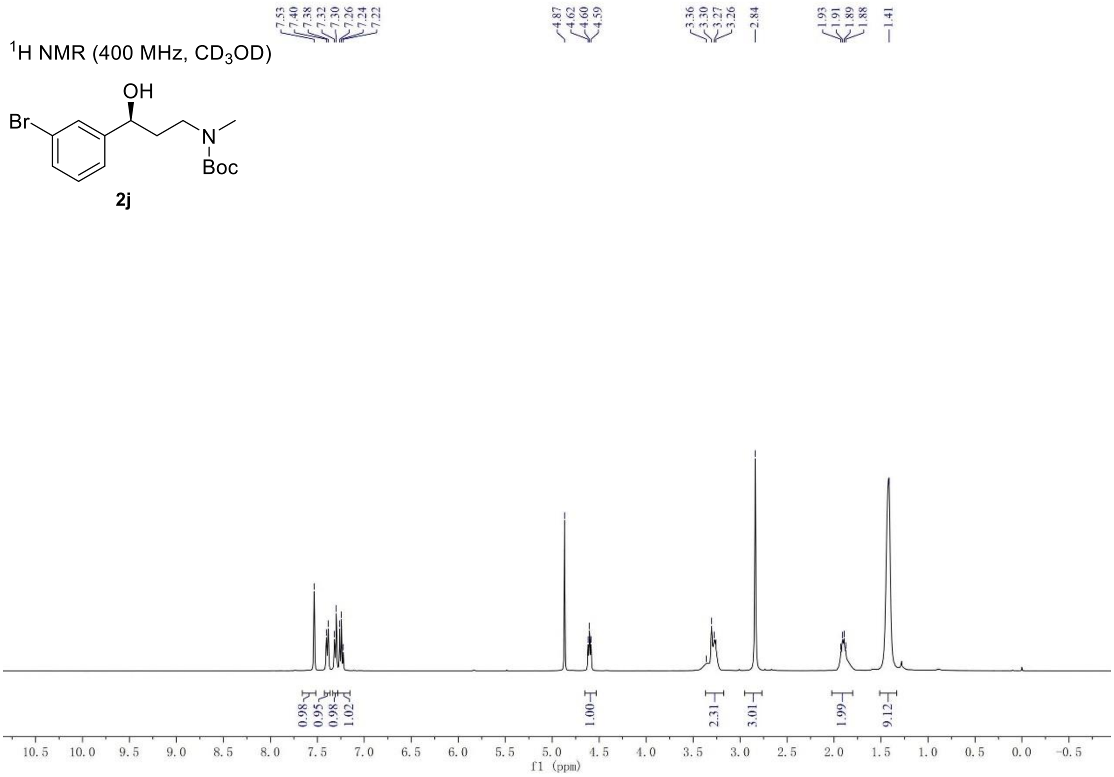

Supplementary Figure 43. ^{1}H NMR spectra of **2j**

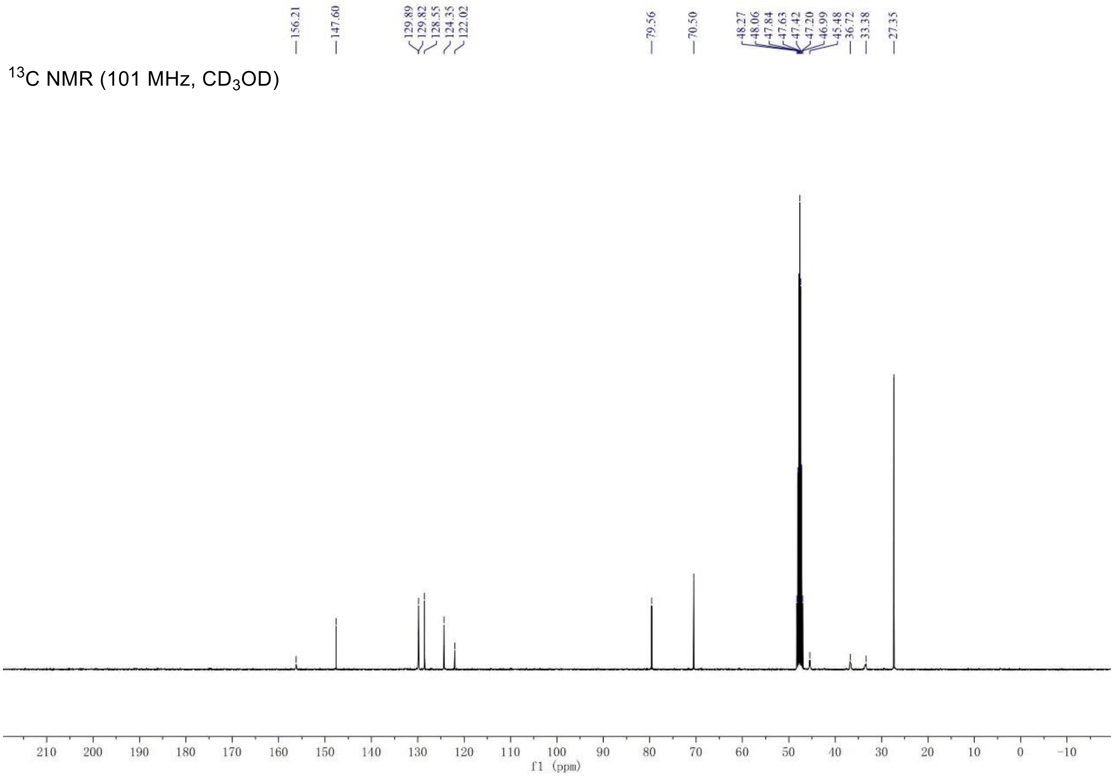

Supplementary Figure 44. ^{13}C NMR spectra of **2j**

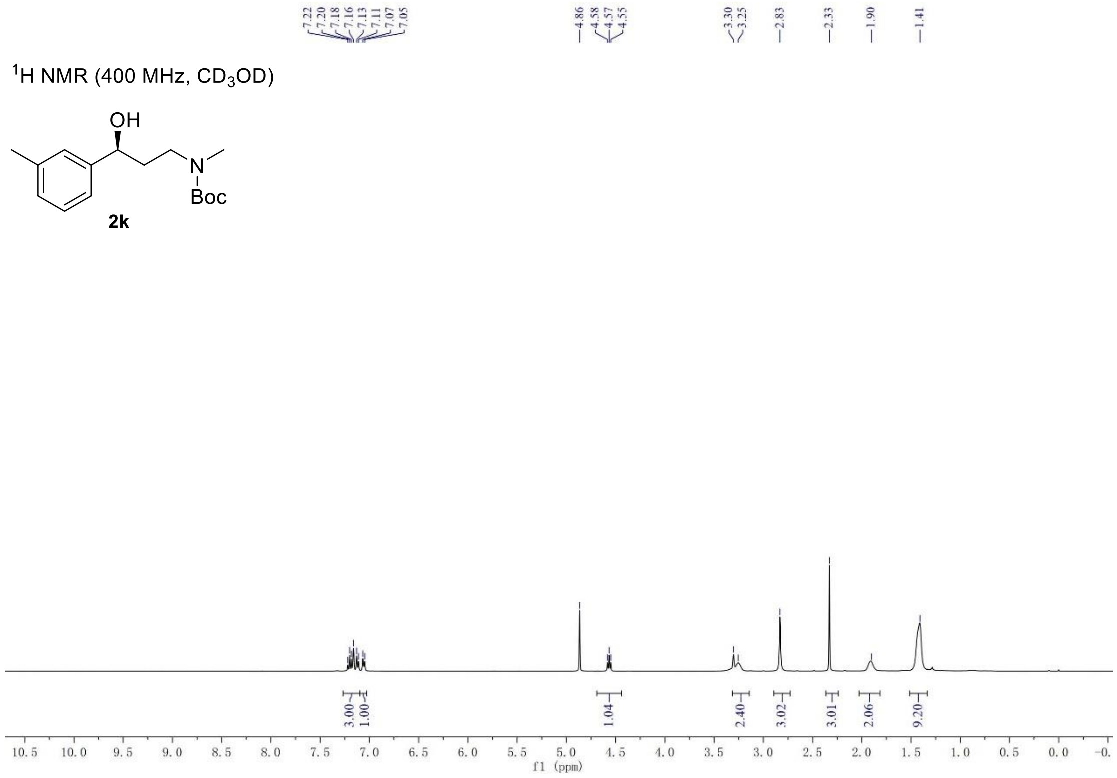

Supplementary Figure 45. ^{1}H NMR spectra of **2k**

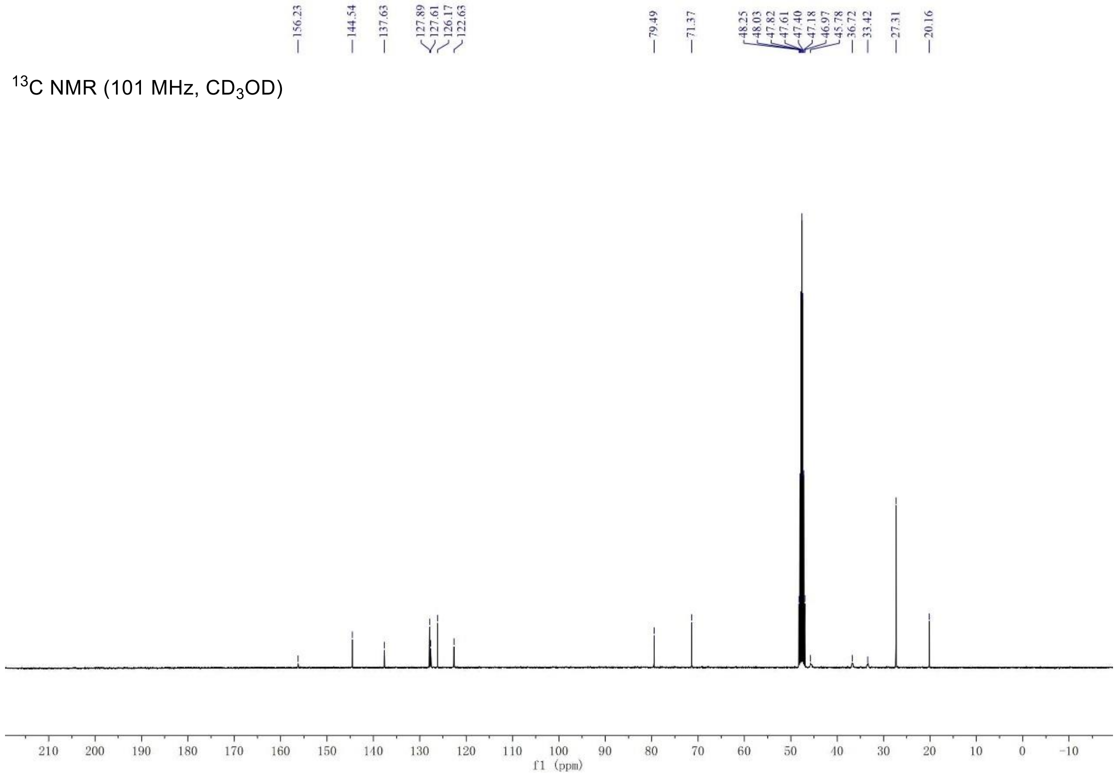

Supplementary Figure 46. ^{13}C NMR spectra of **2k**

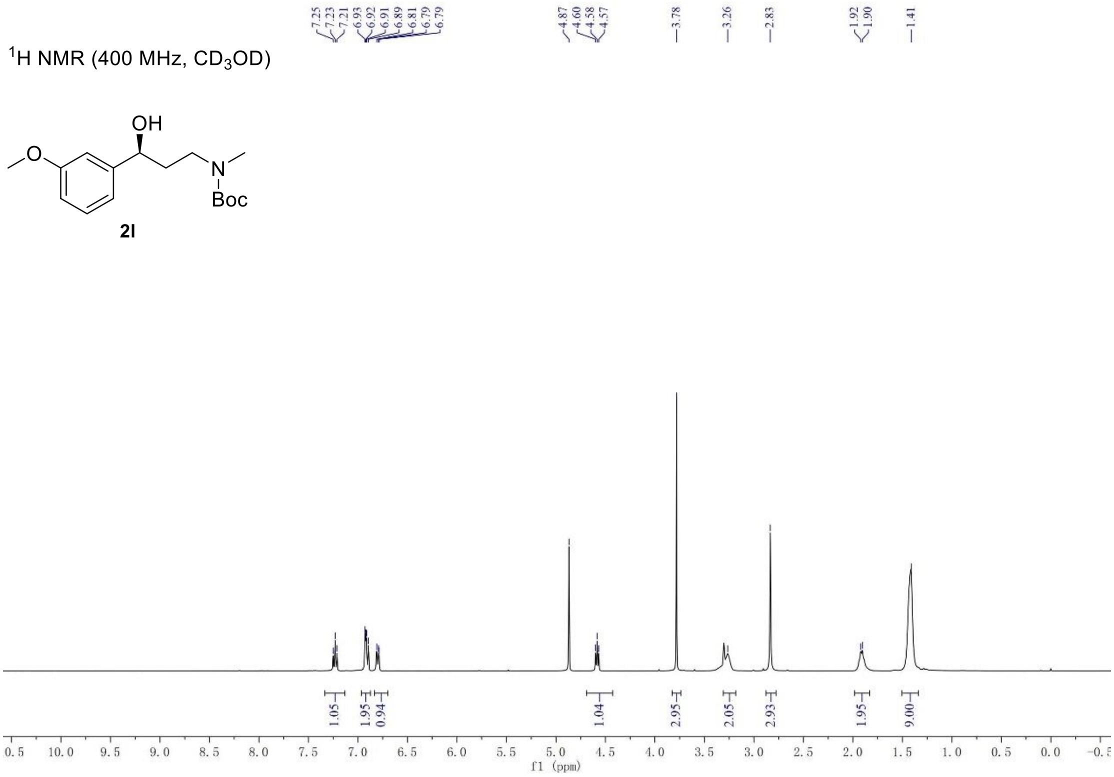

Supplementary Figure 47. ^{1}H NMR spectra of **2l**

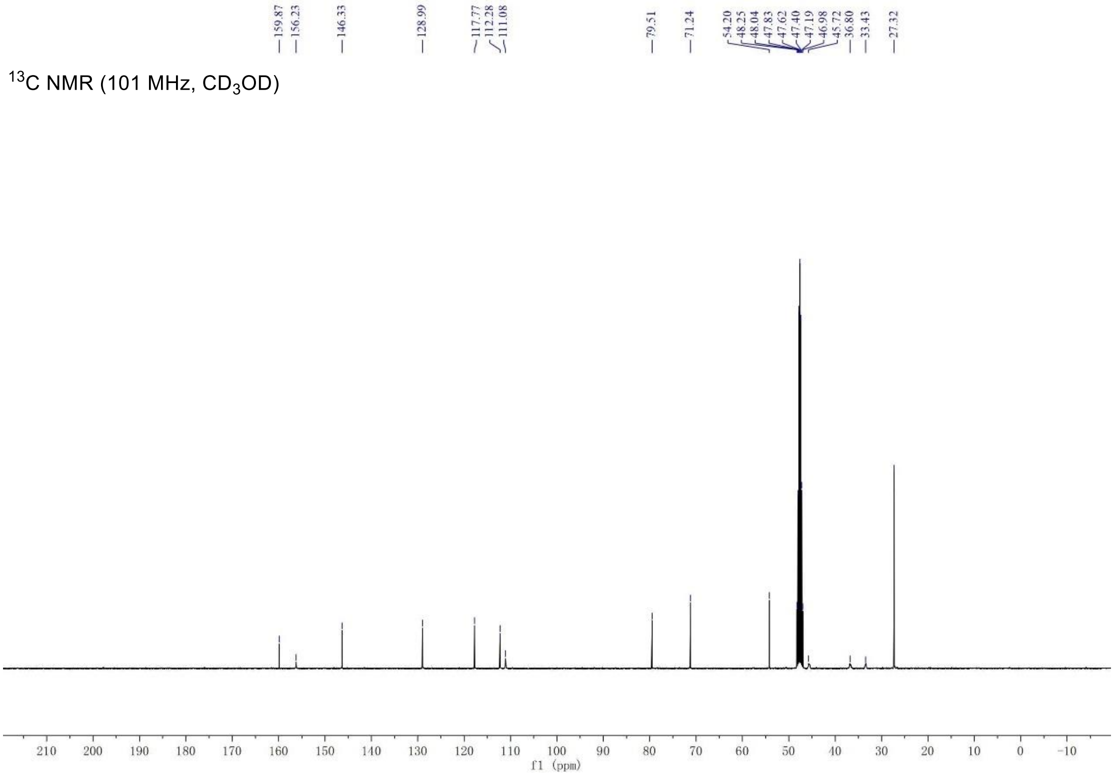

Supplementary Figure 48. ^{13}C NMR spectra of **2l**

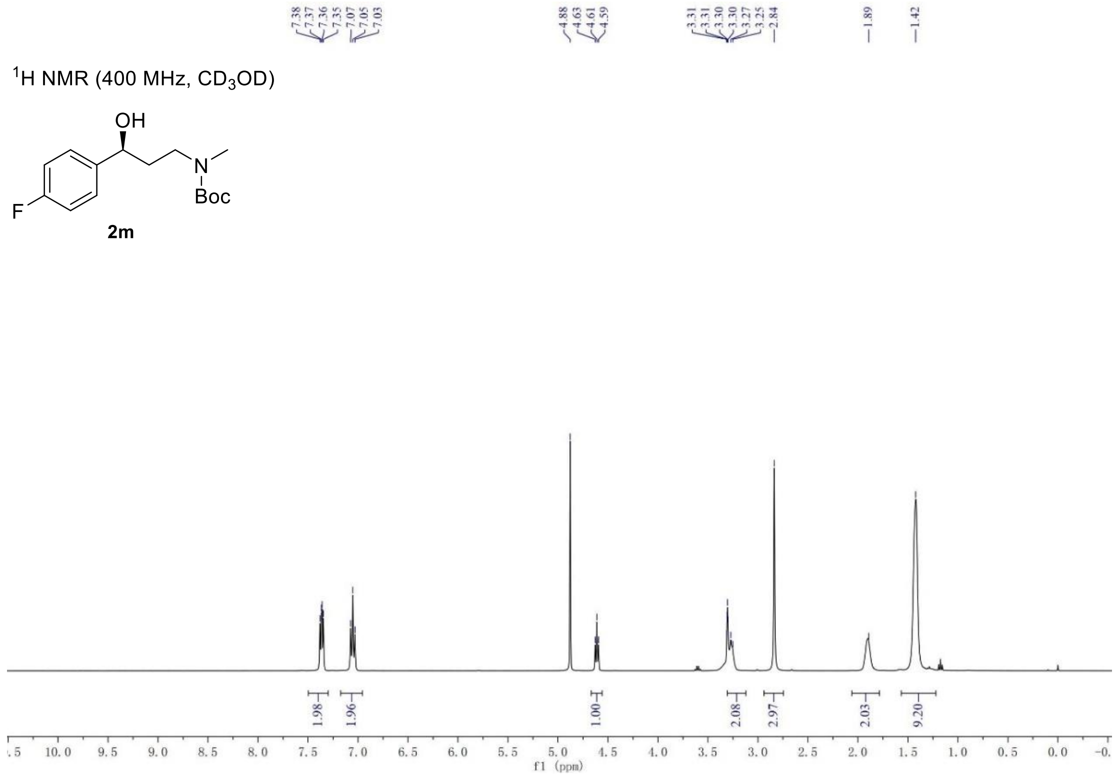

Supplementary Figure 49. ^{1}H NMR spectra of **2m**

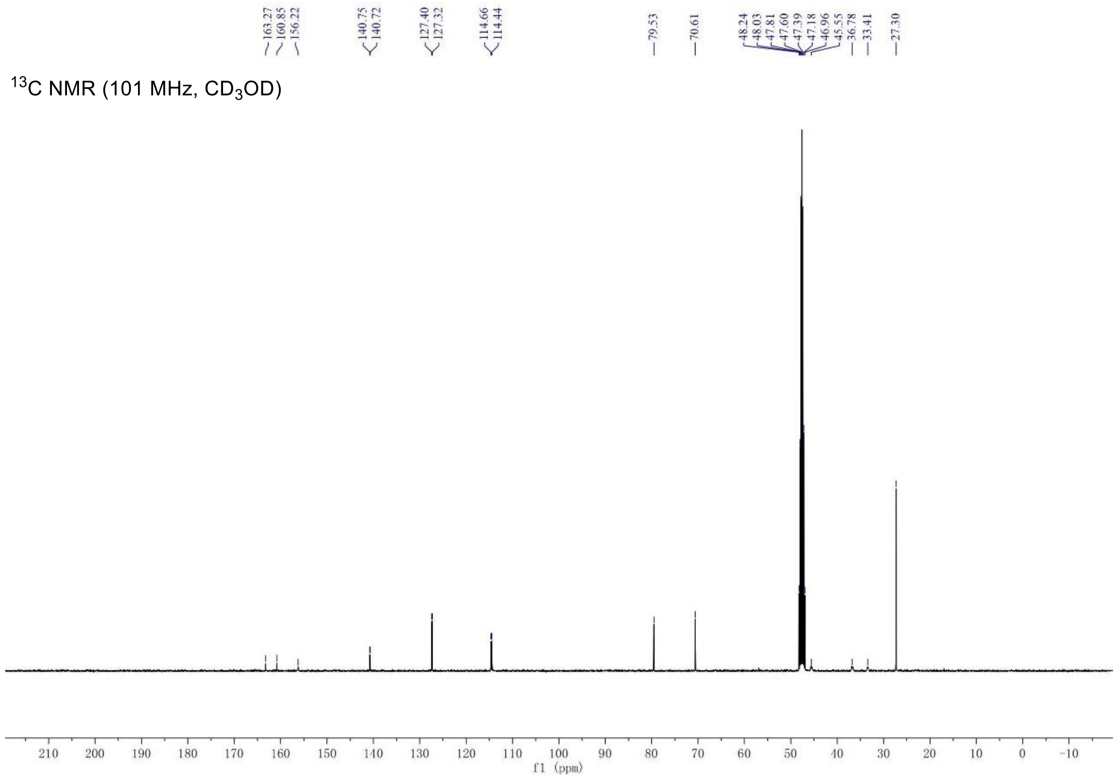

Supplementary Figure 50. ^{13}C NMR spectra of **2m**

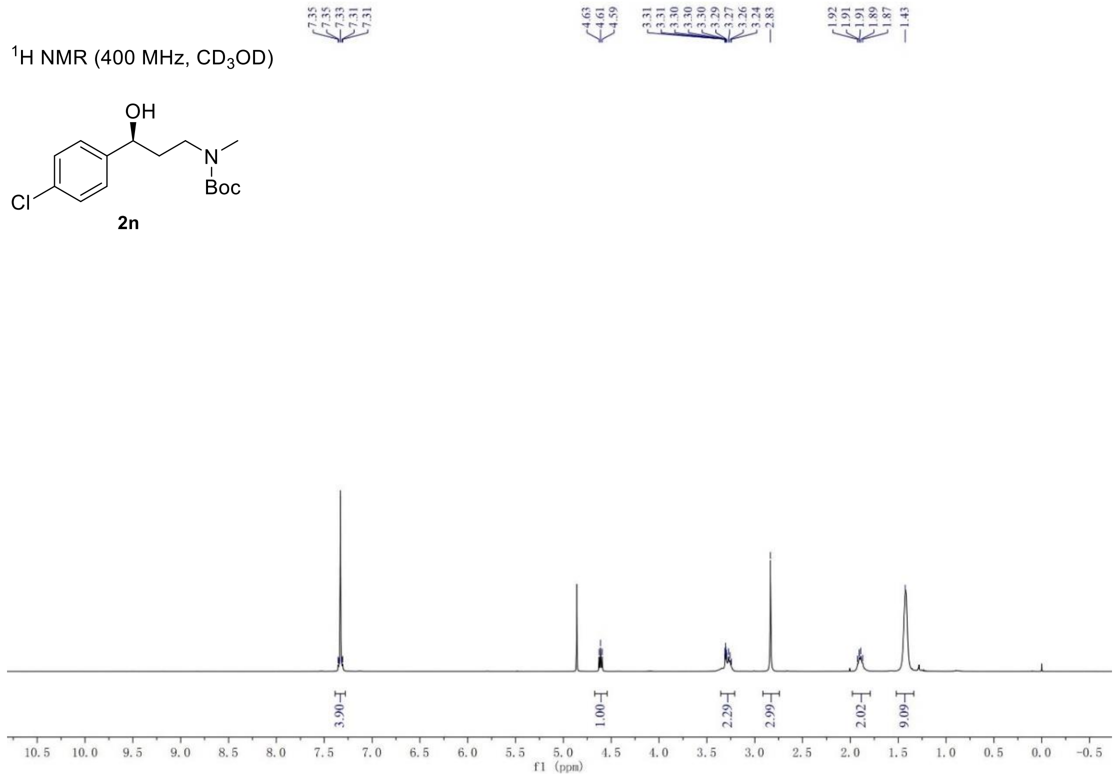

Supplementary Figure 51. ^{1}H NMR spectra of **2n**

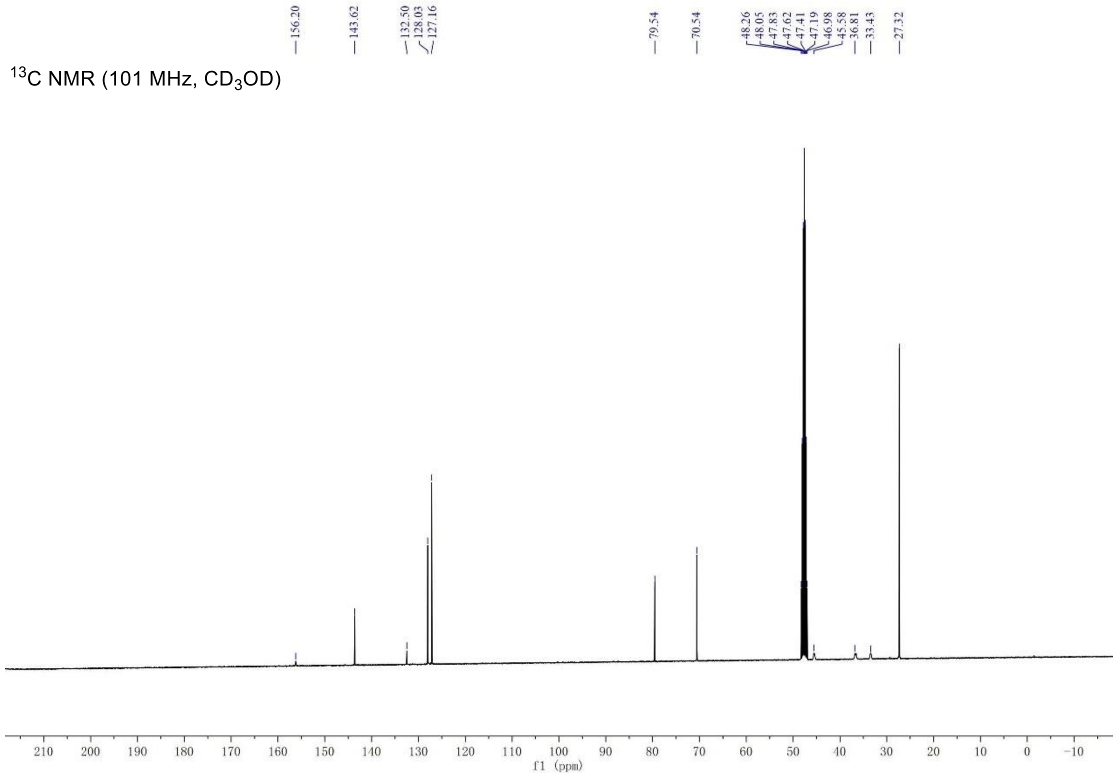

Supplementary Figure 52. ^{13}C NMR spectra of **2n**

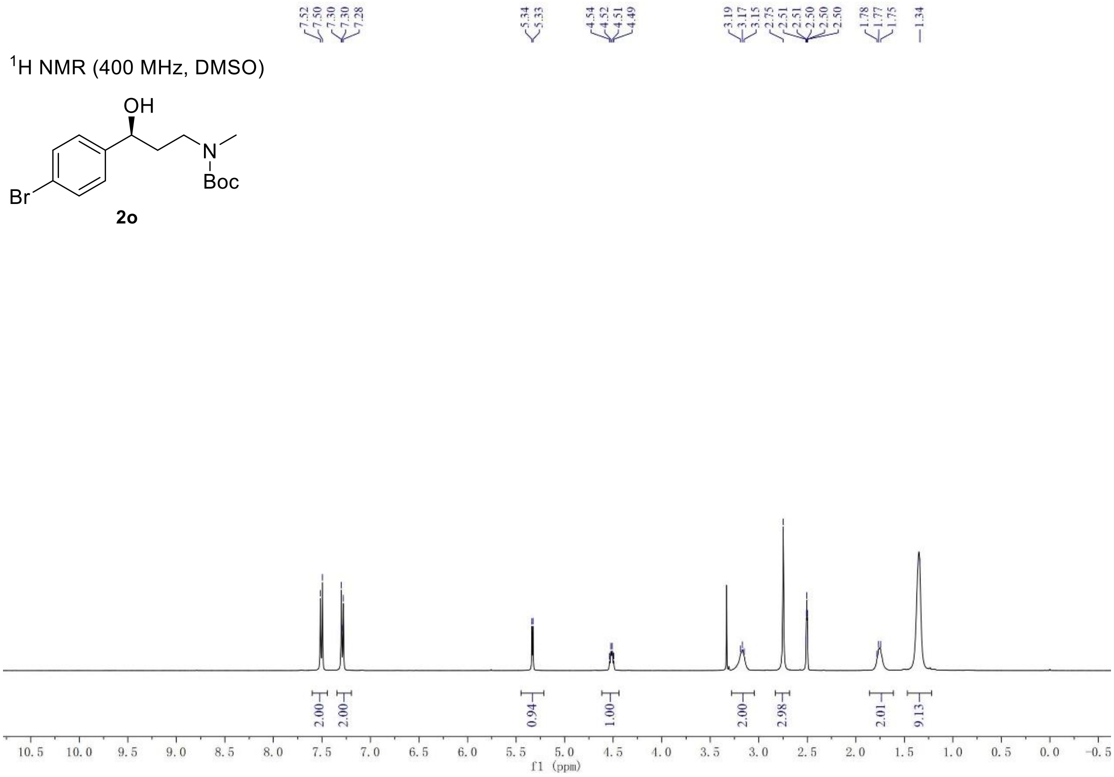

Supplementary Figure 53. ^{1}H NMR spectra of **2o**

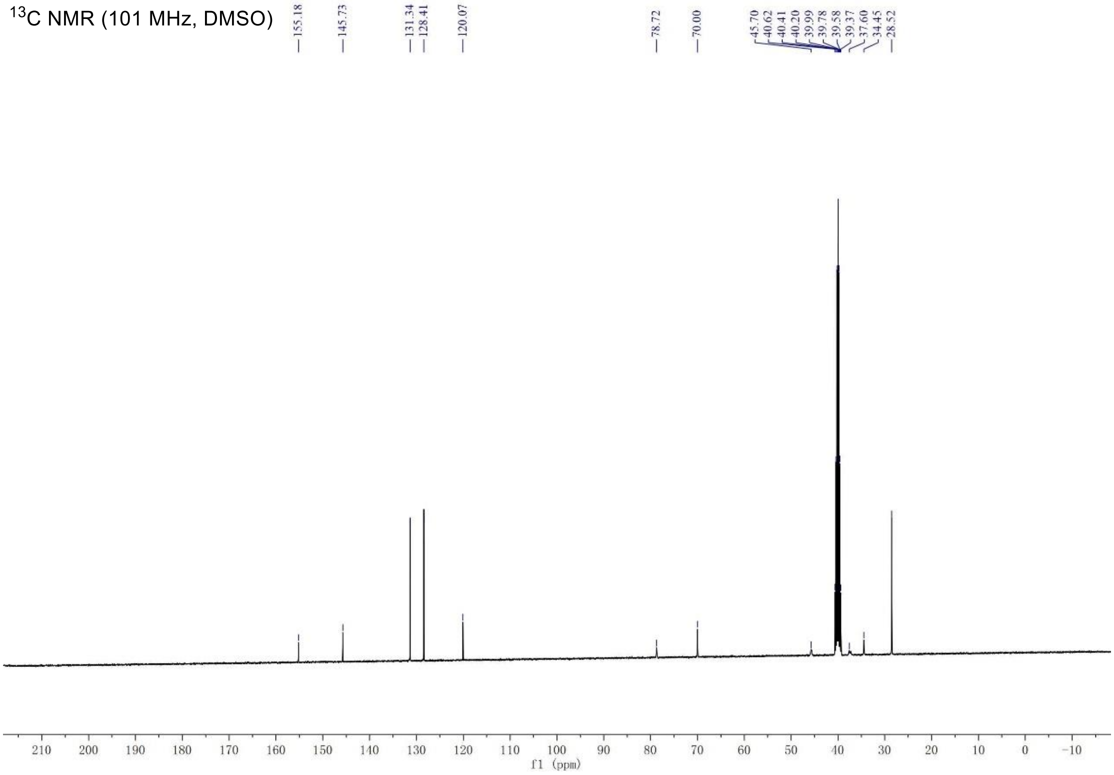

Supplementary Figure 54. ^{13}C NMR spectra of **2o**

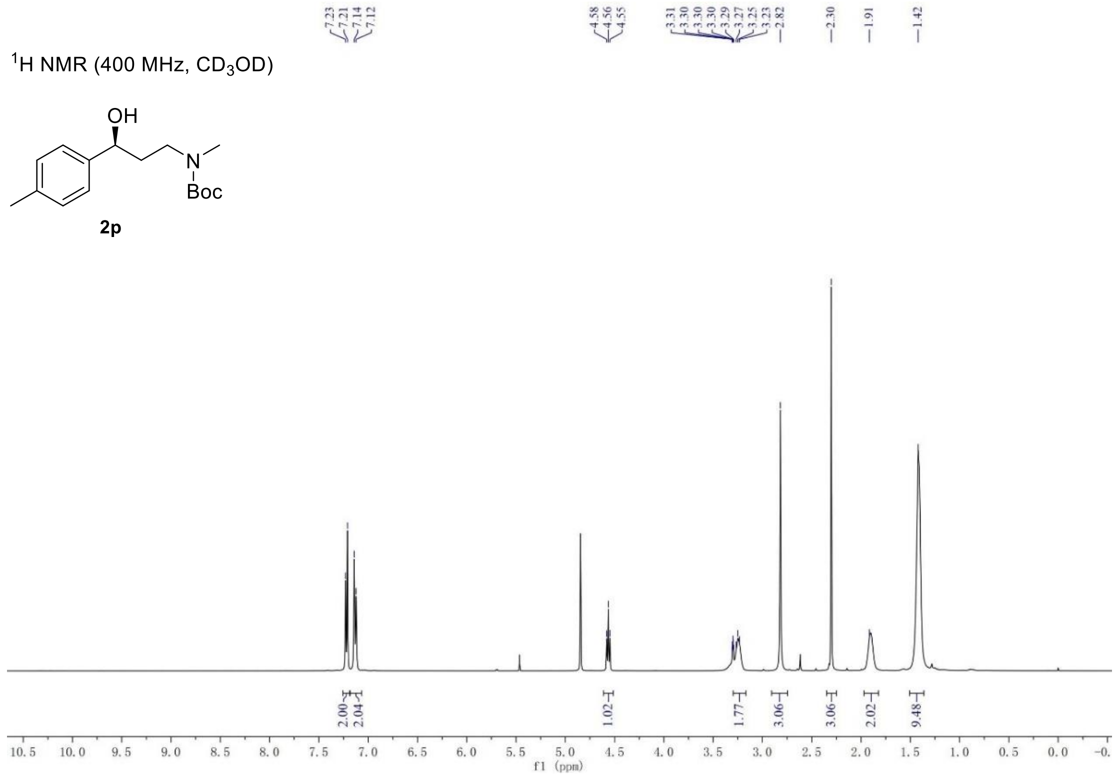

Supplementary Figure 55. ^{1}H NMR spectra of **2p**

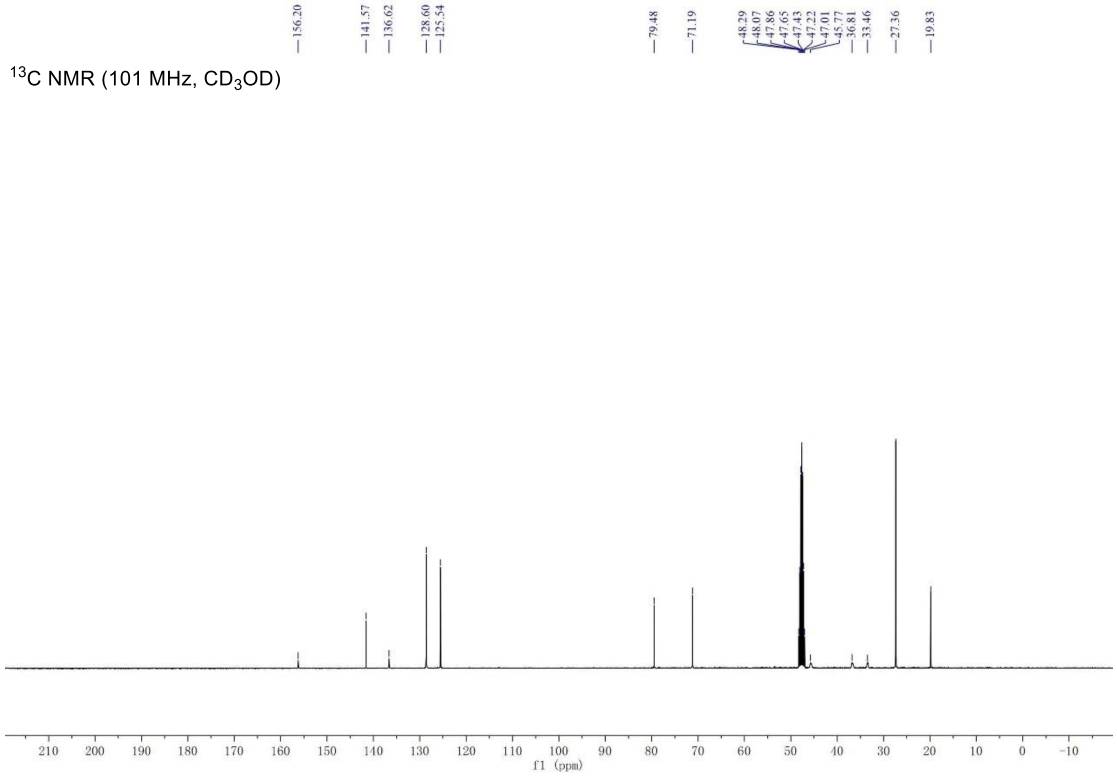

Supplementary Figure 56. ^{13}C NMR spectra of **2p**

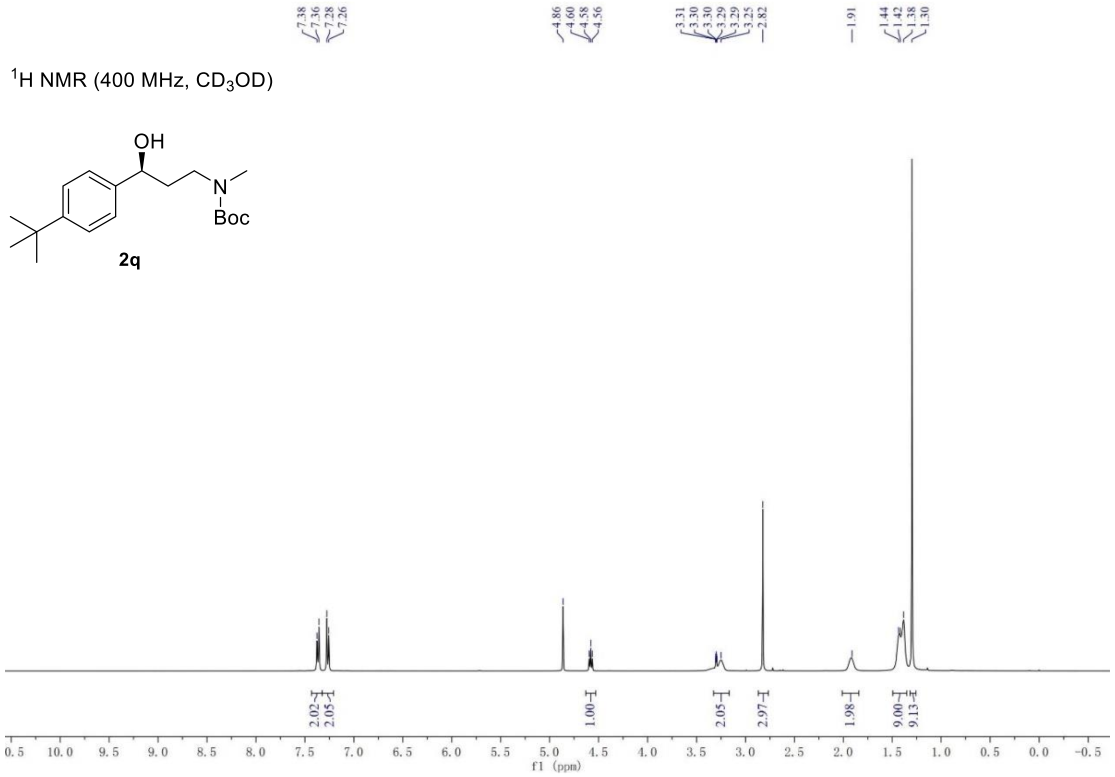

Supplementary Figure 57. ^{1}H NMR spectra of **2q**

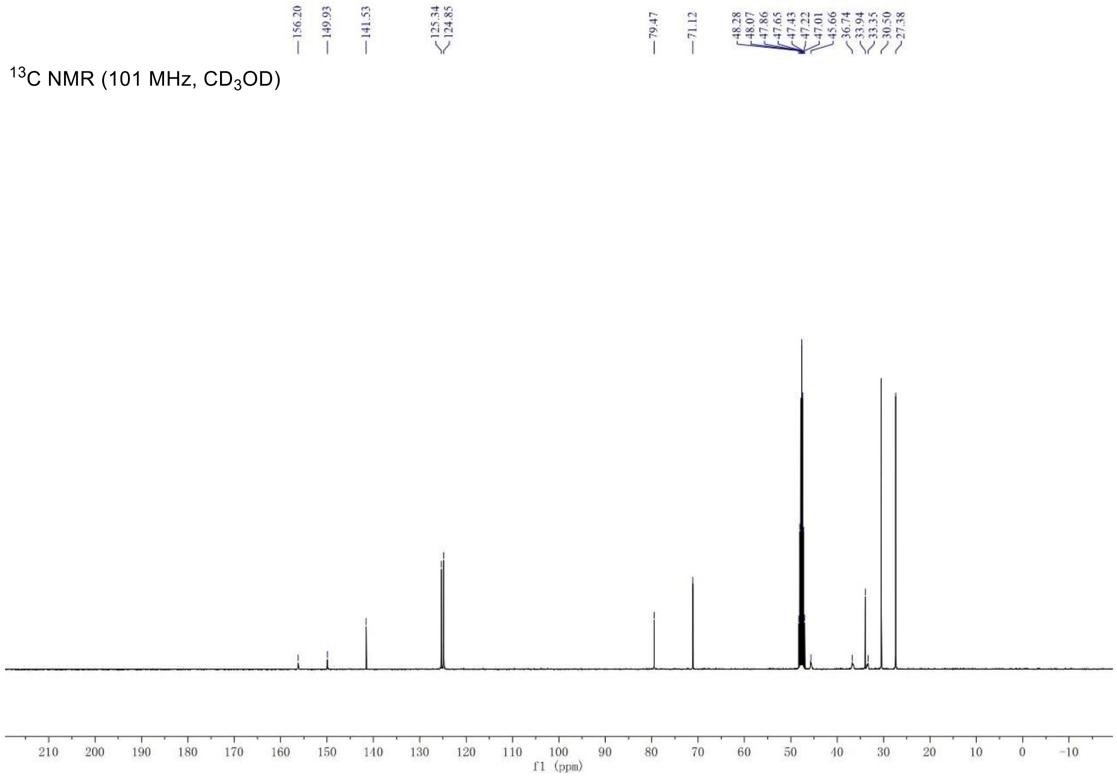

Supplementary Figure 58. ^{13}C NMR spectra of **2q**

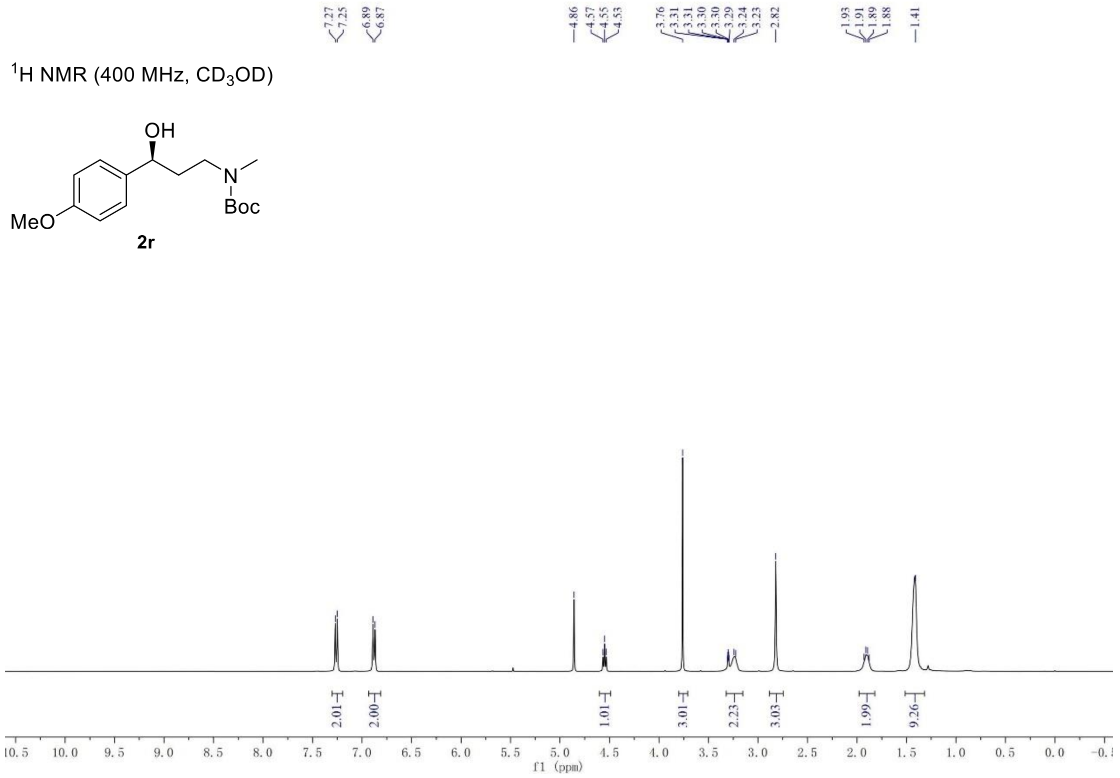

Supplementary Figure 59. ^{1}H NMR spectra of **2r**

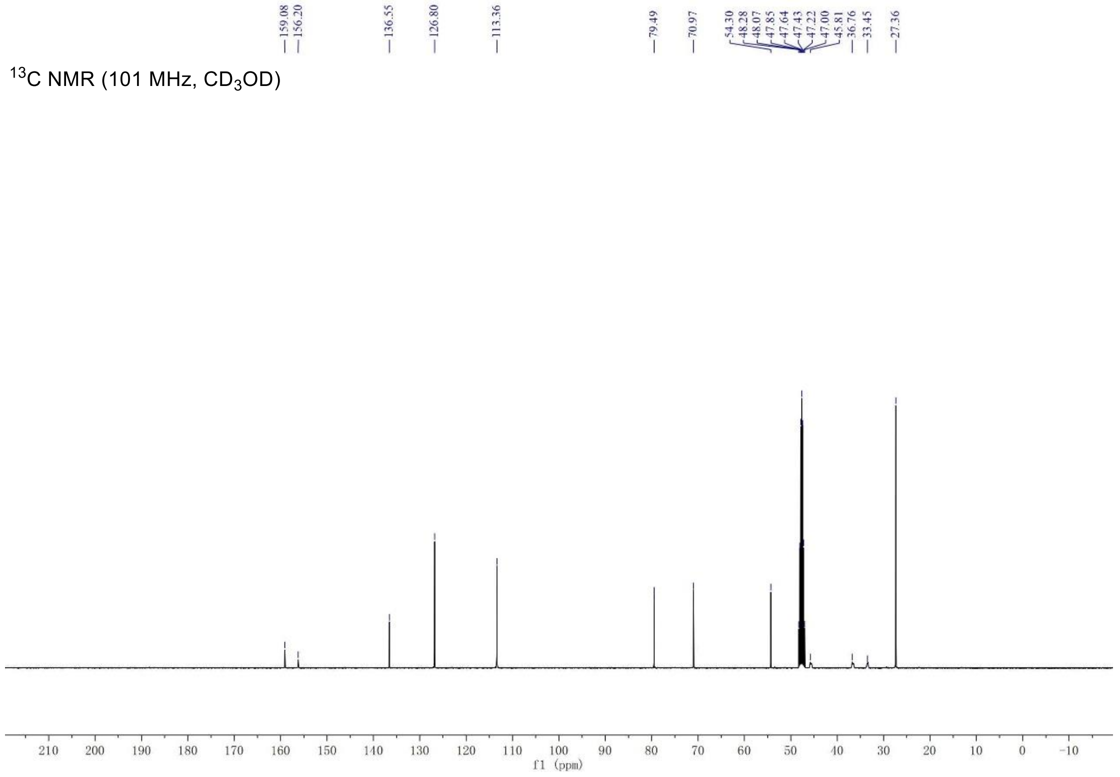

Supplementary Figure 60. ^{13}C NMR spectra of **2r**

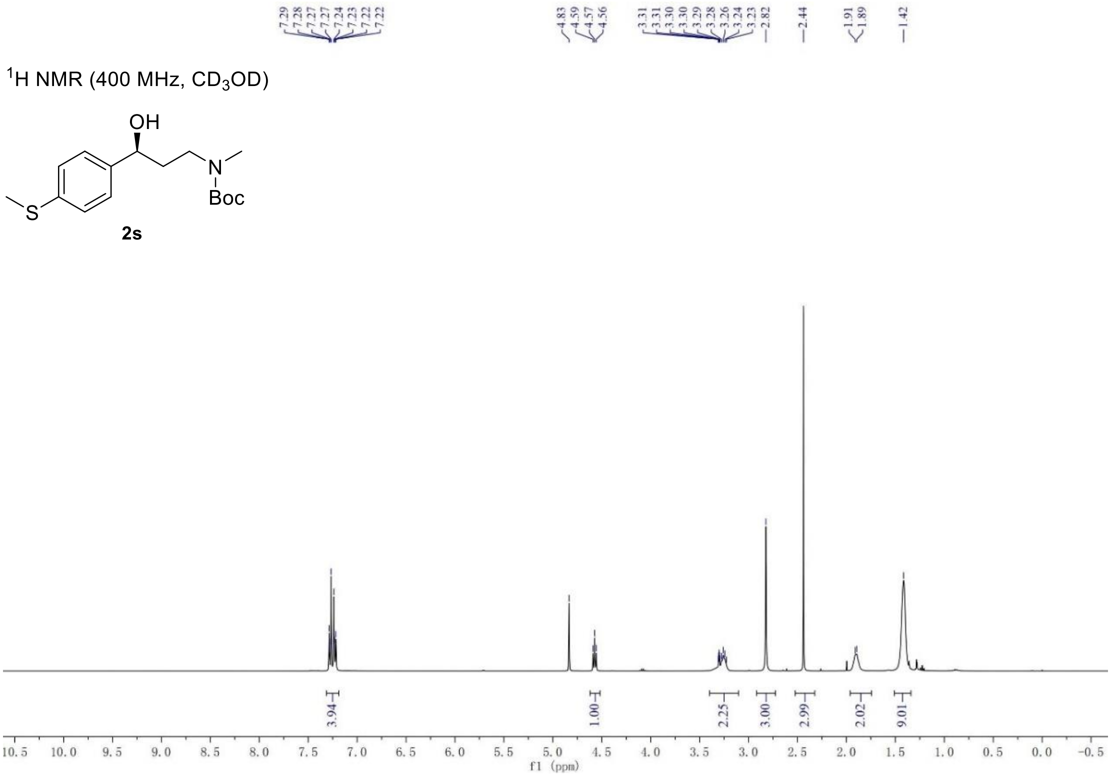

Supplementary Figure 61. ^{1}H NMR spectra of **2s**

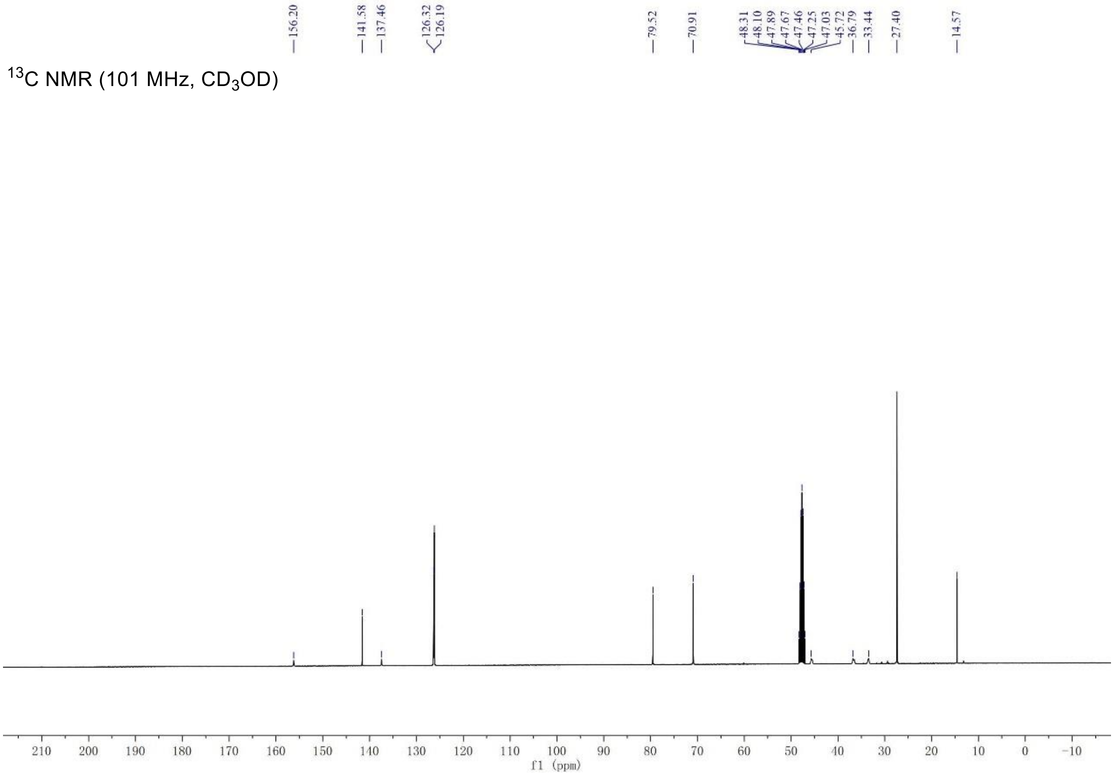

Supplementary Figure 62. ^{13}C NMR spectra of **2s**

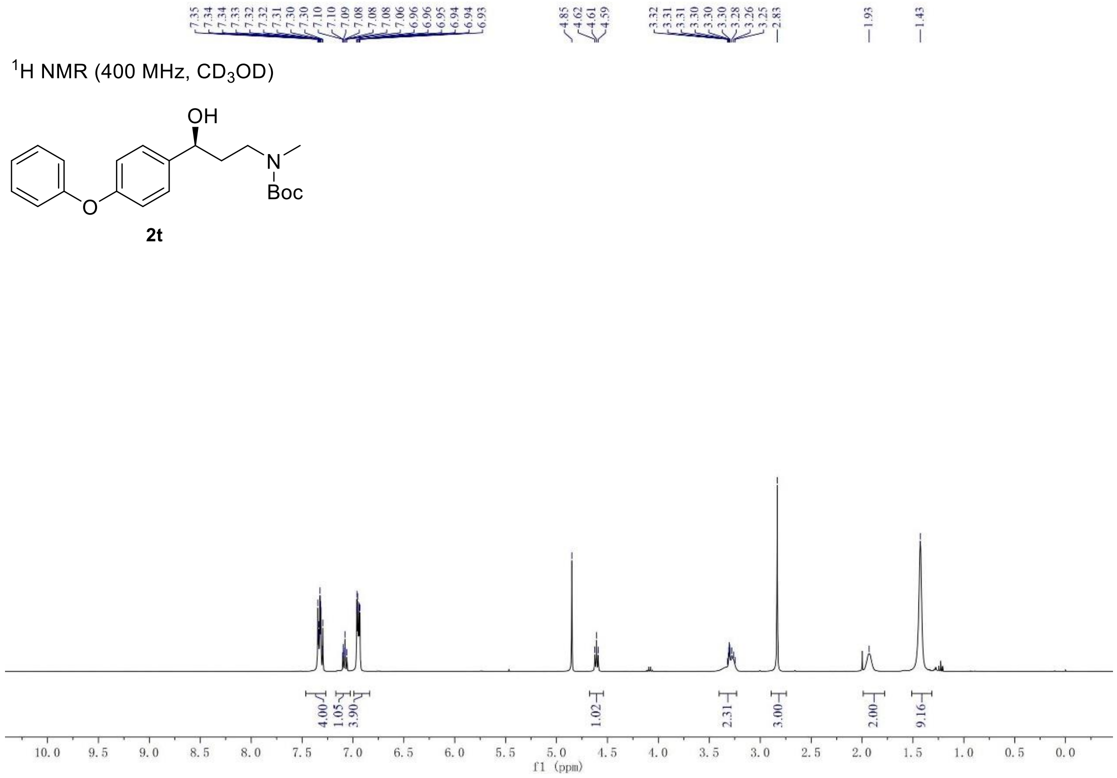

Supplementary Figure 63. ^{1}H NMR spectra of **2t**

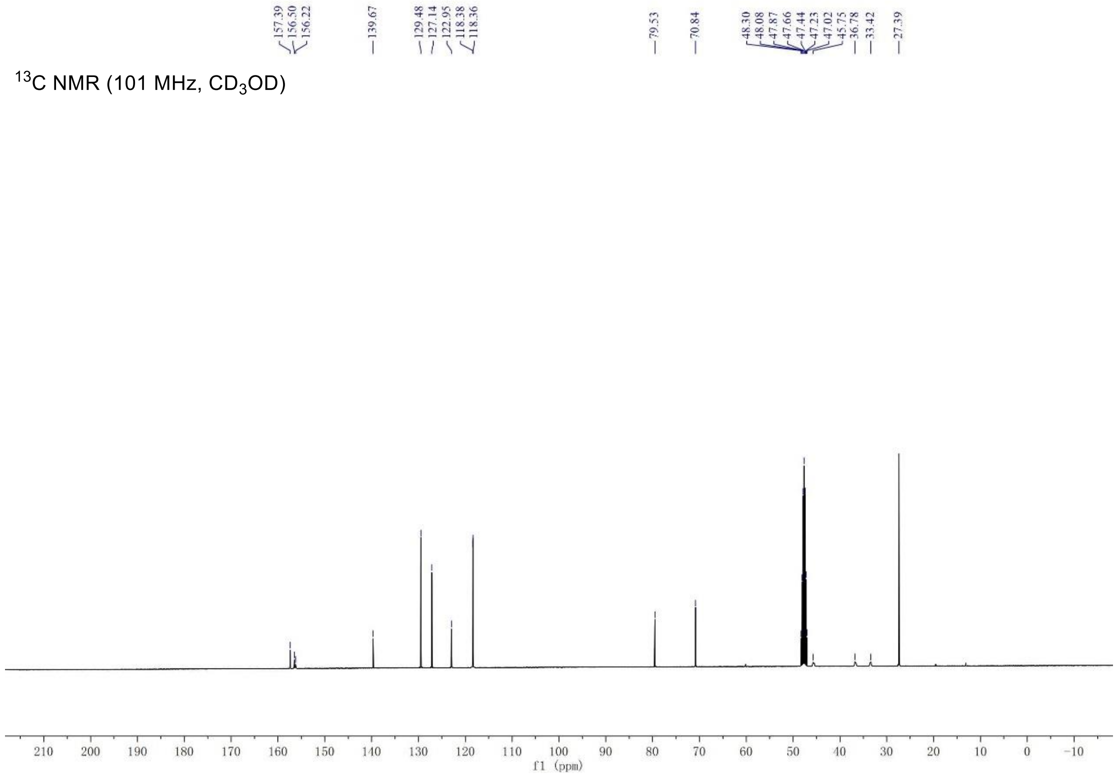

Supplementary Figure 64. ^{13}C NMR spectra of **2t**

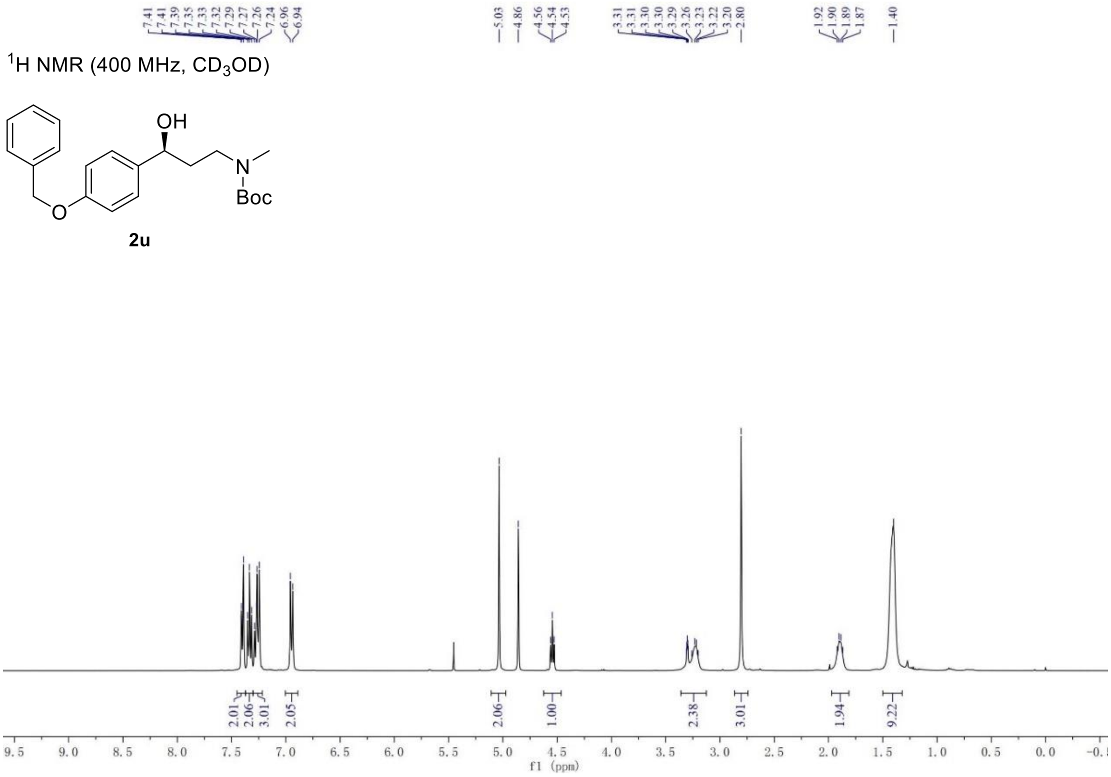

Supplementary Figure 65. ^{1}H NMR spectra of **2u**

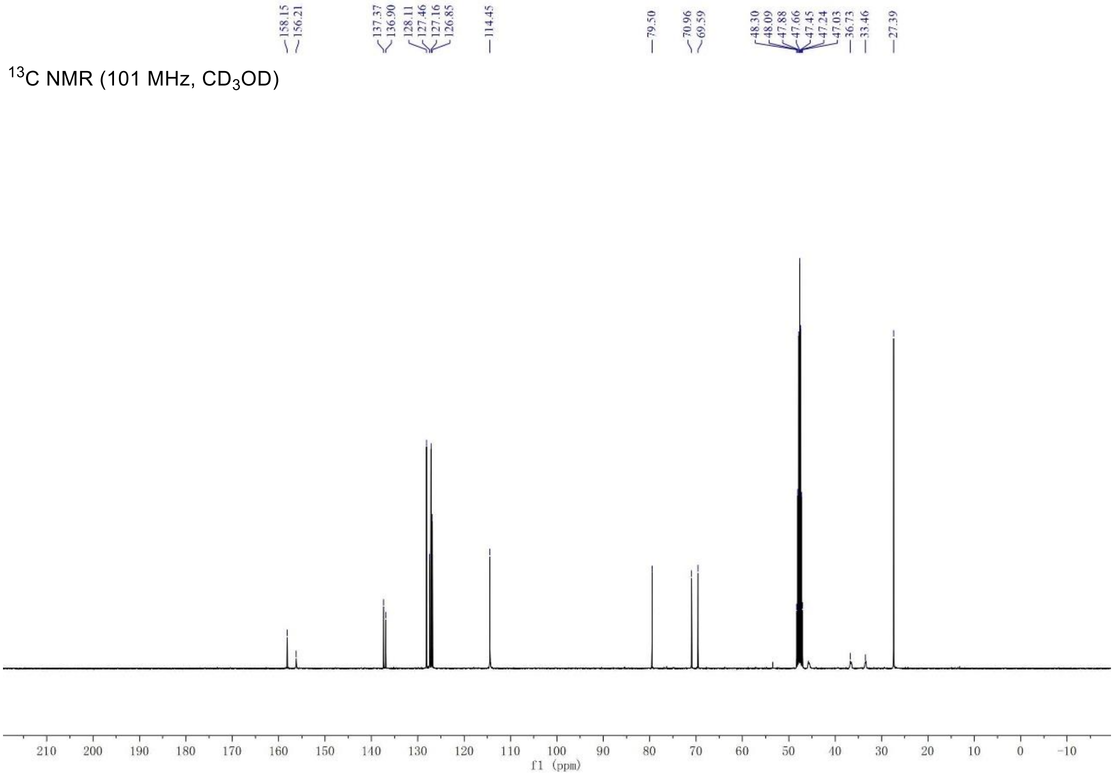

Supplementary Figure 66. ^{13}C NMR spectra of **2u**

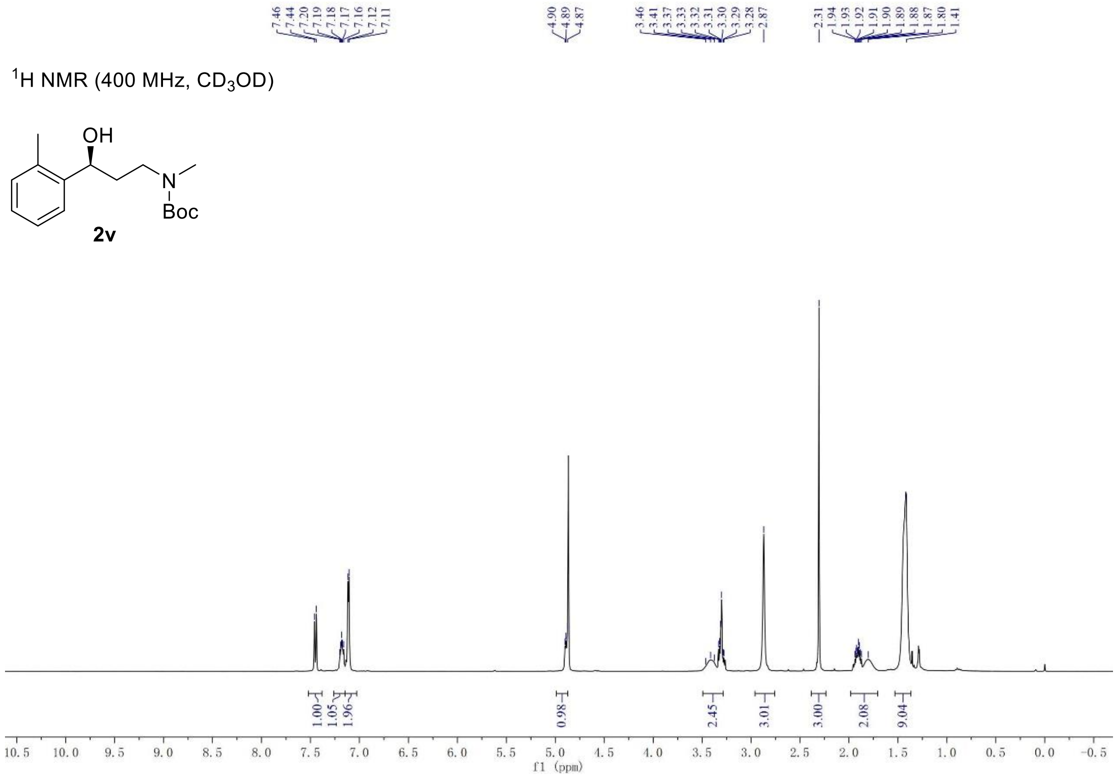

Supplementary Figure 67. ^{1}H NMR spectra of **2v**

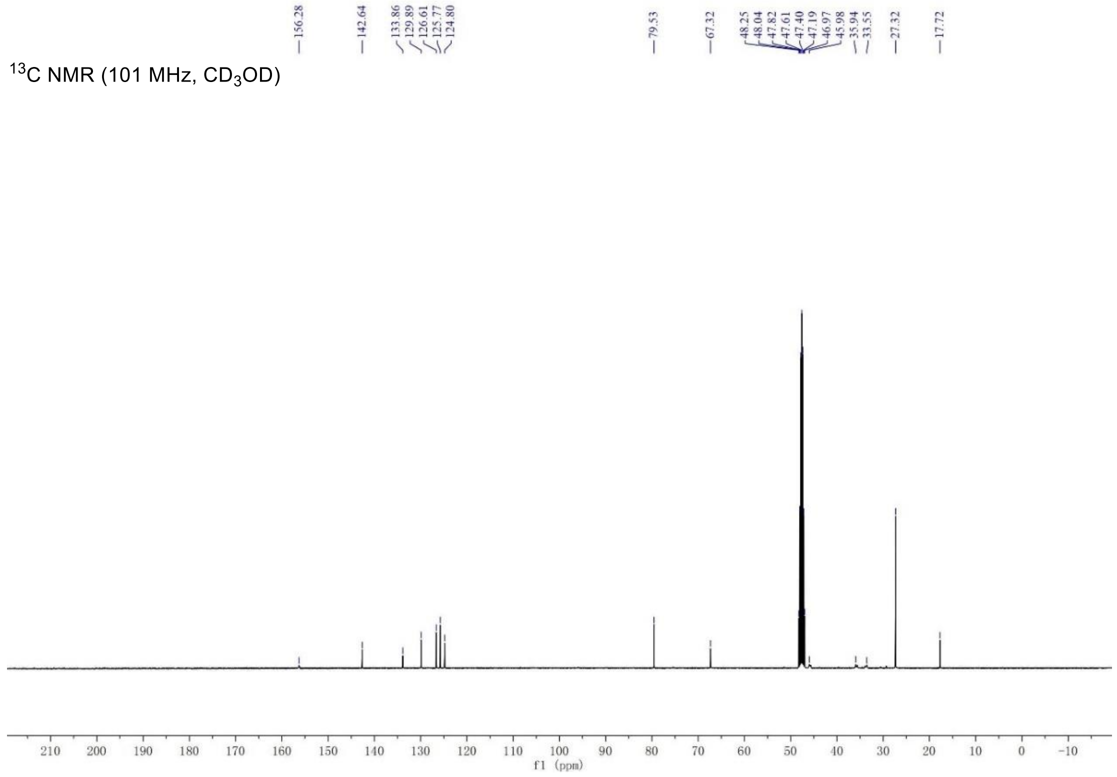

Supplementary Figure 68. ^{13}C NMR spectra of **2v**

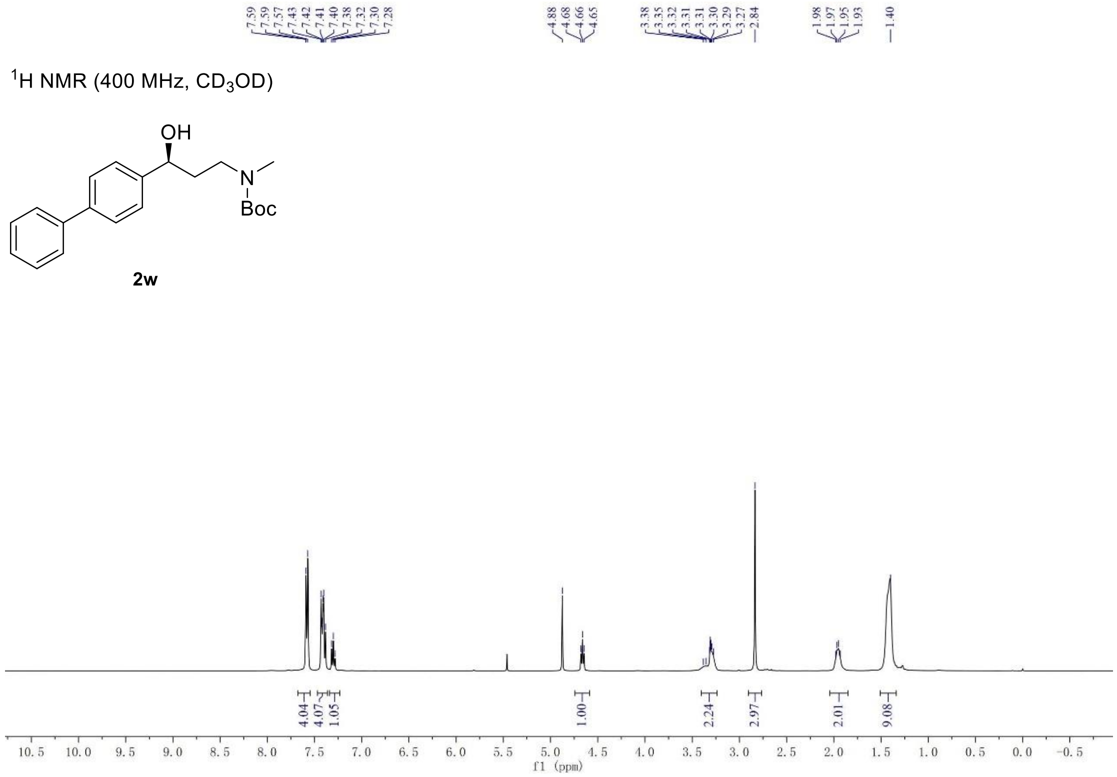

Supplementary Figure 69. ^{1}H NMR spectra of **2w**

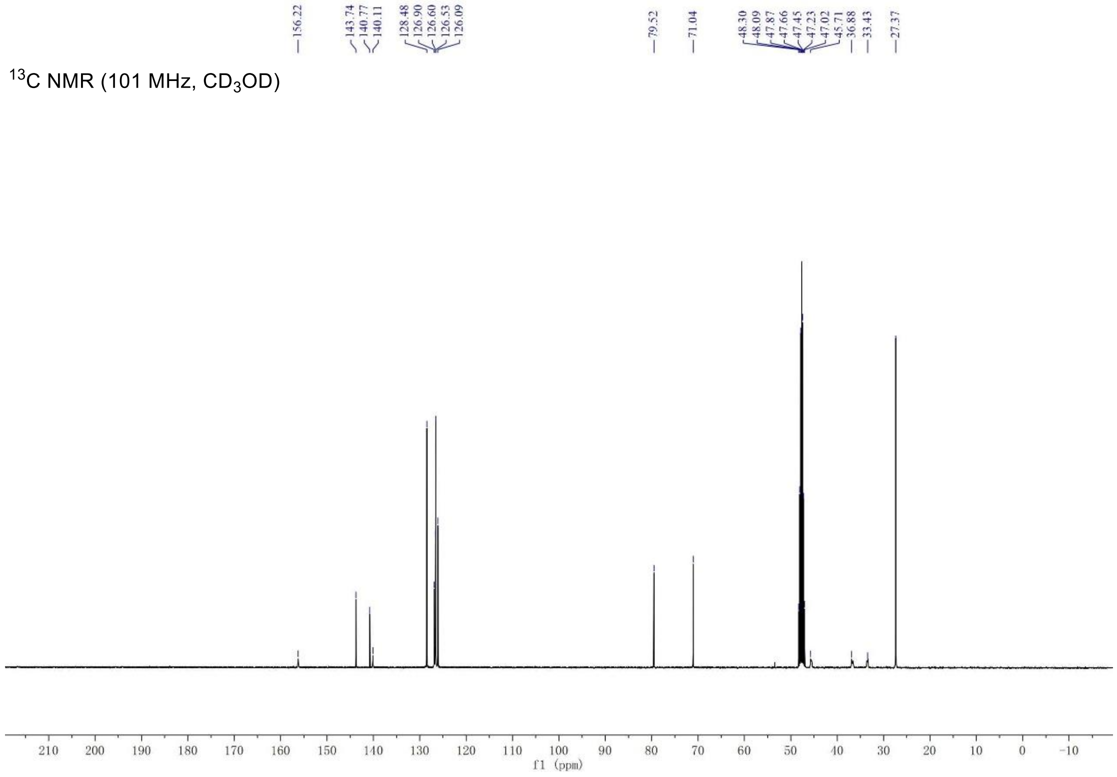

Supplementary Figure 70. ^{13}C NMR spectra of **2w**

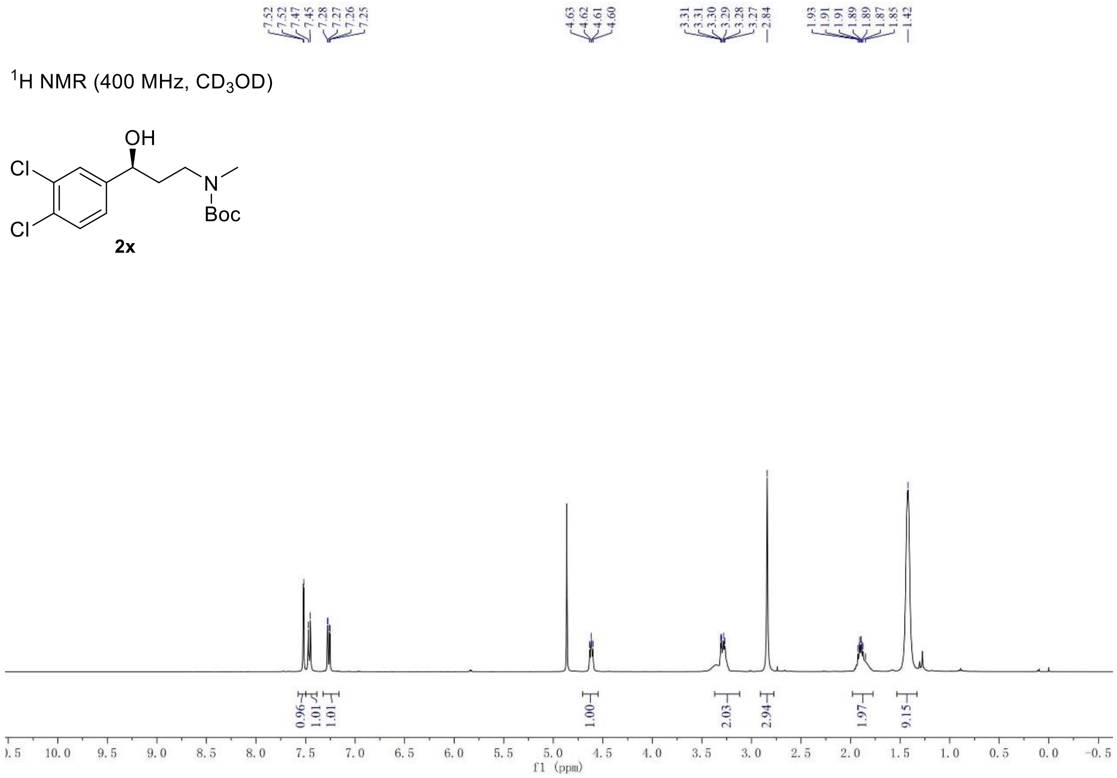

Supplementary Figure 71. ^{1}H NMR spectra of **2x**

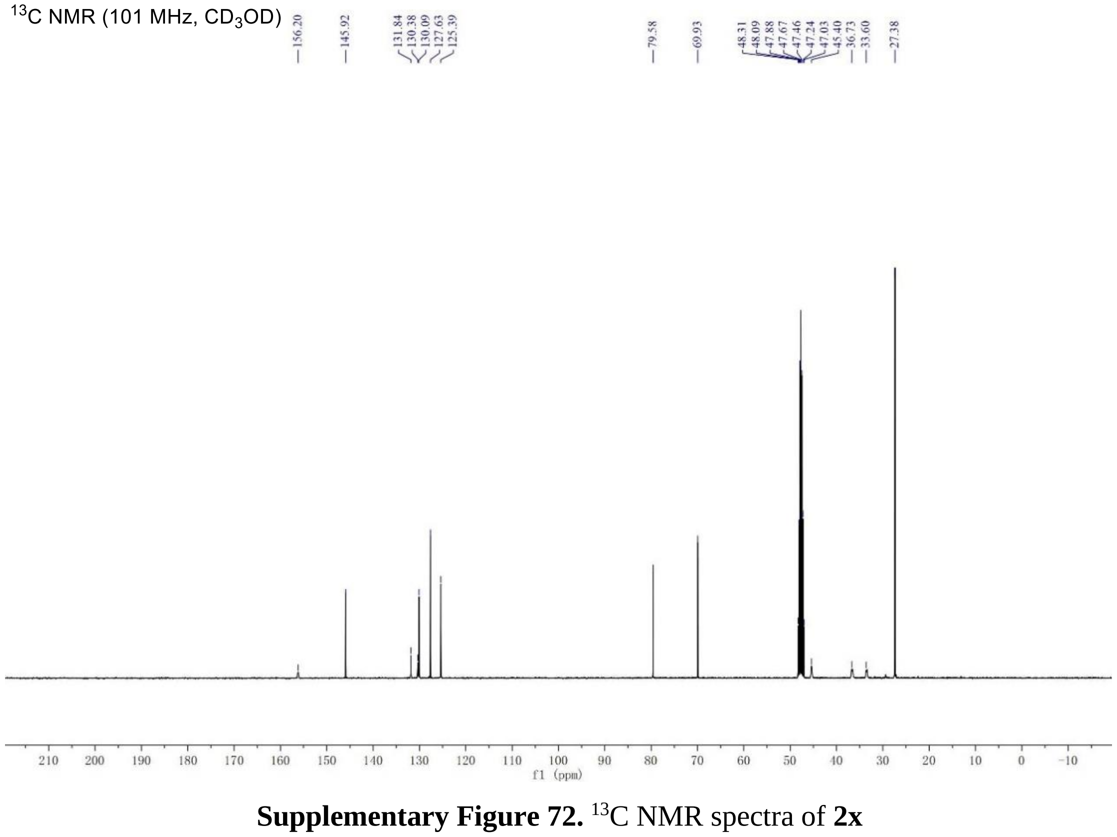

Supplementary Figure 72. ^{13}C NMR spectra of **2x**

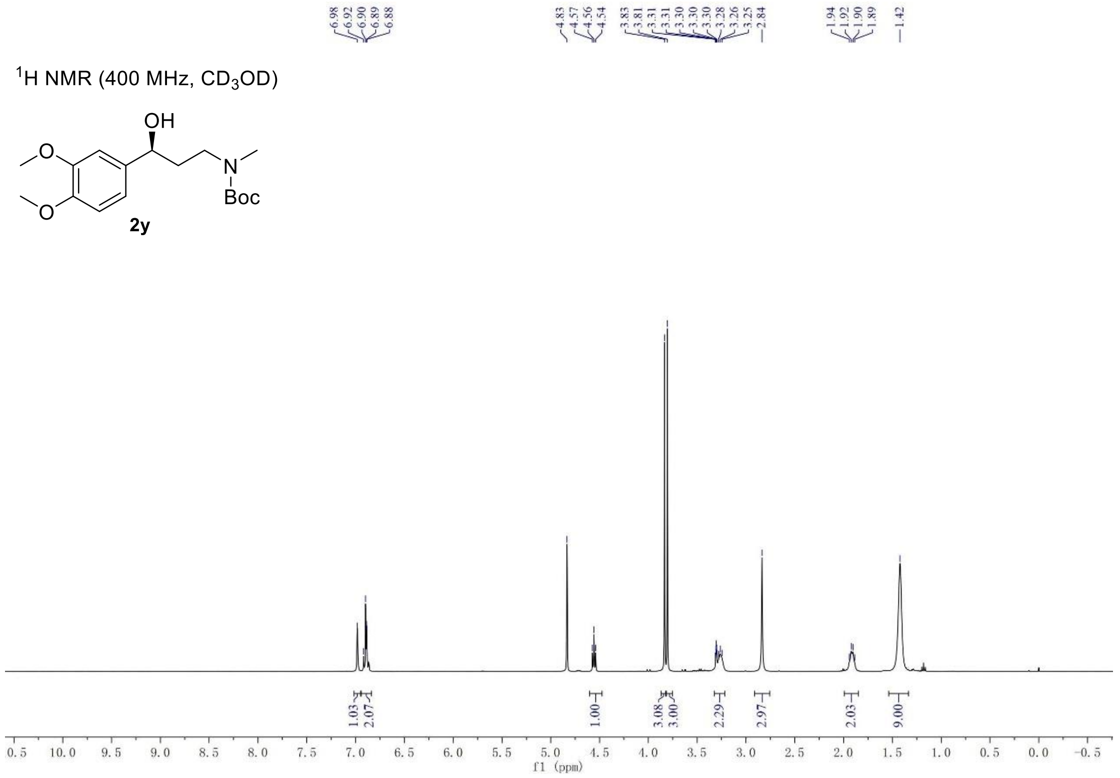

Supplementary Figure 73. ^{1}H NMR spectra of **2y**

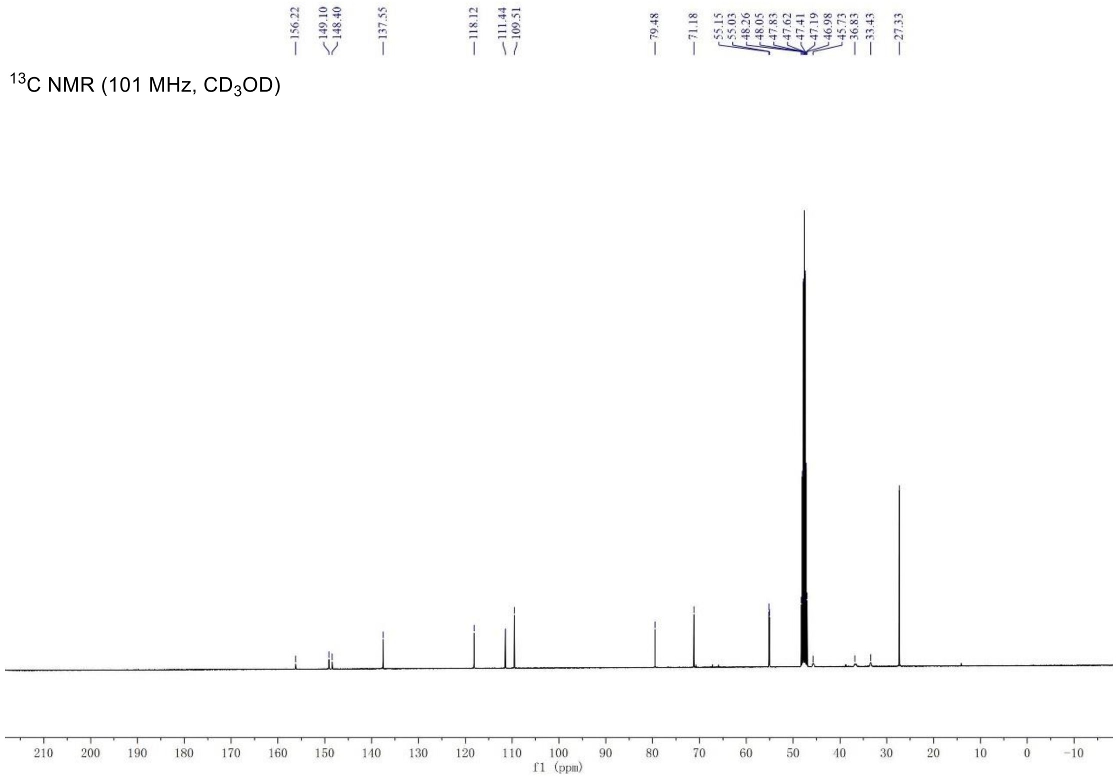

Supplementary Figure 74. ^{13}C NMR spectra of **2y**

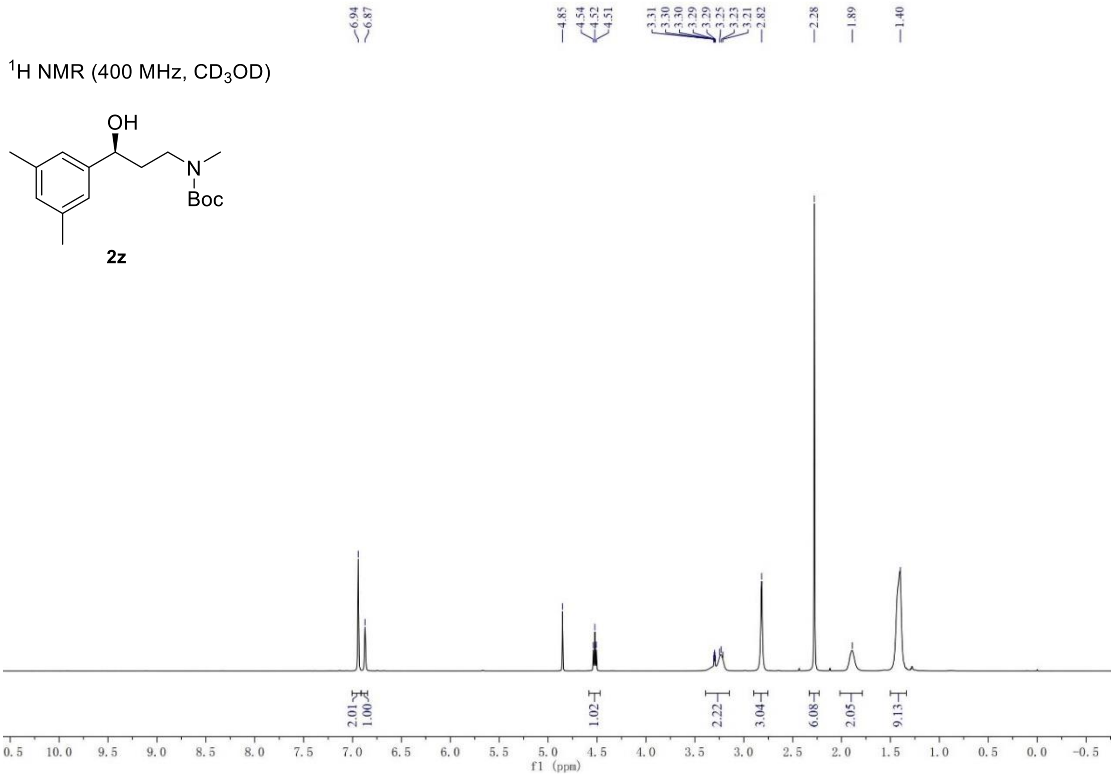

Supplementary Figure 75. ^{1}H NMR spectra of **2z**

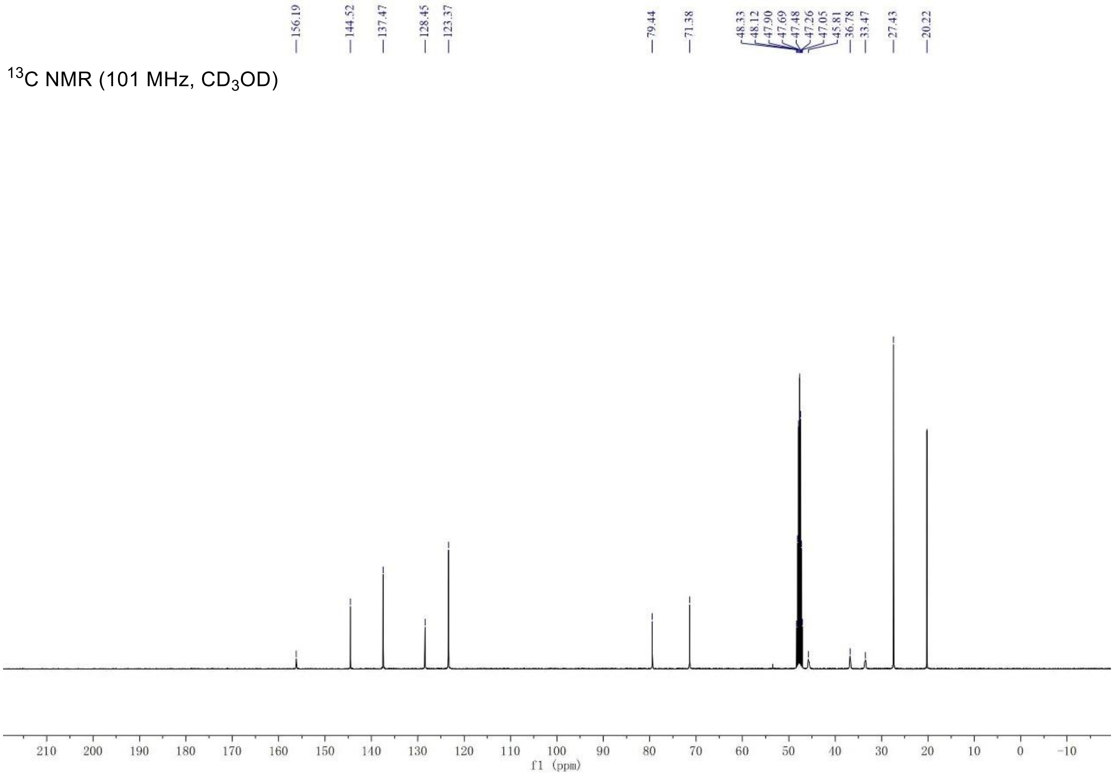

Supplementary Figure 76. ^{13}C NMR spectra of **2z**

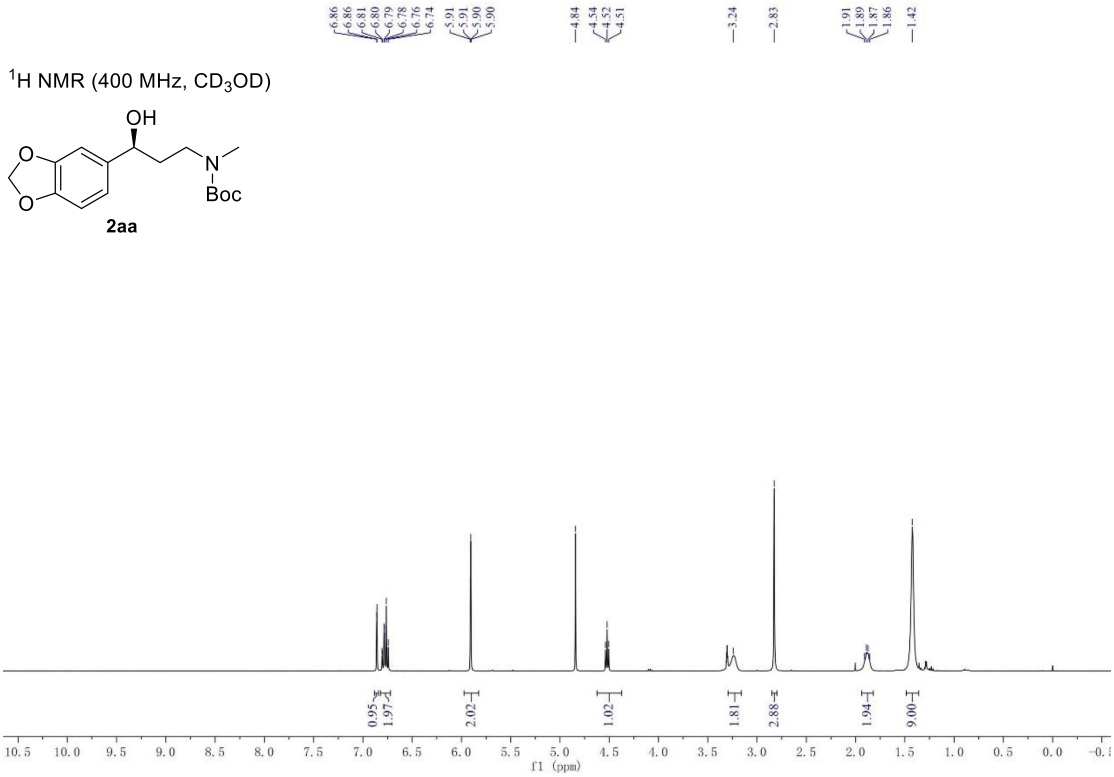

Supplementary Figure 77. ^{1}H NMR spectra of **2aa**

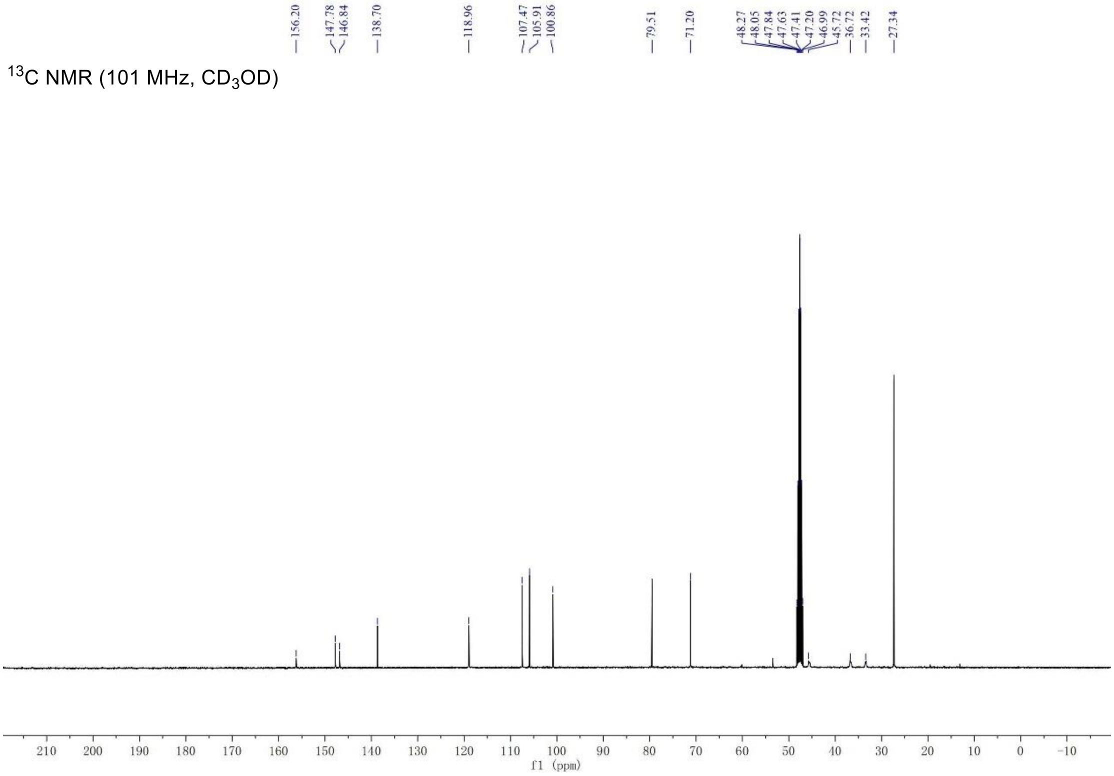

Supplementary Figure 78. ^{13}C NMR spectra of **2aa**

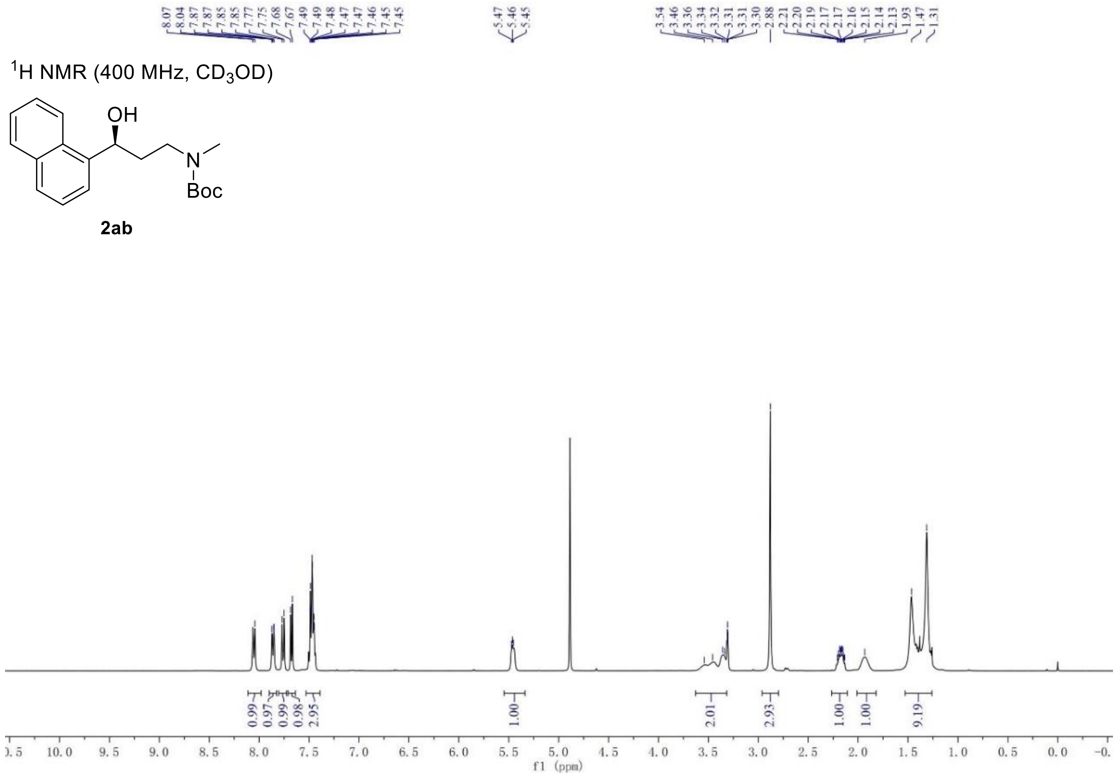

Supplementary Figure 79. ^{1}H NMR spectra of **2ab**

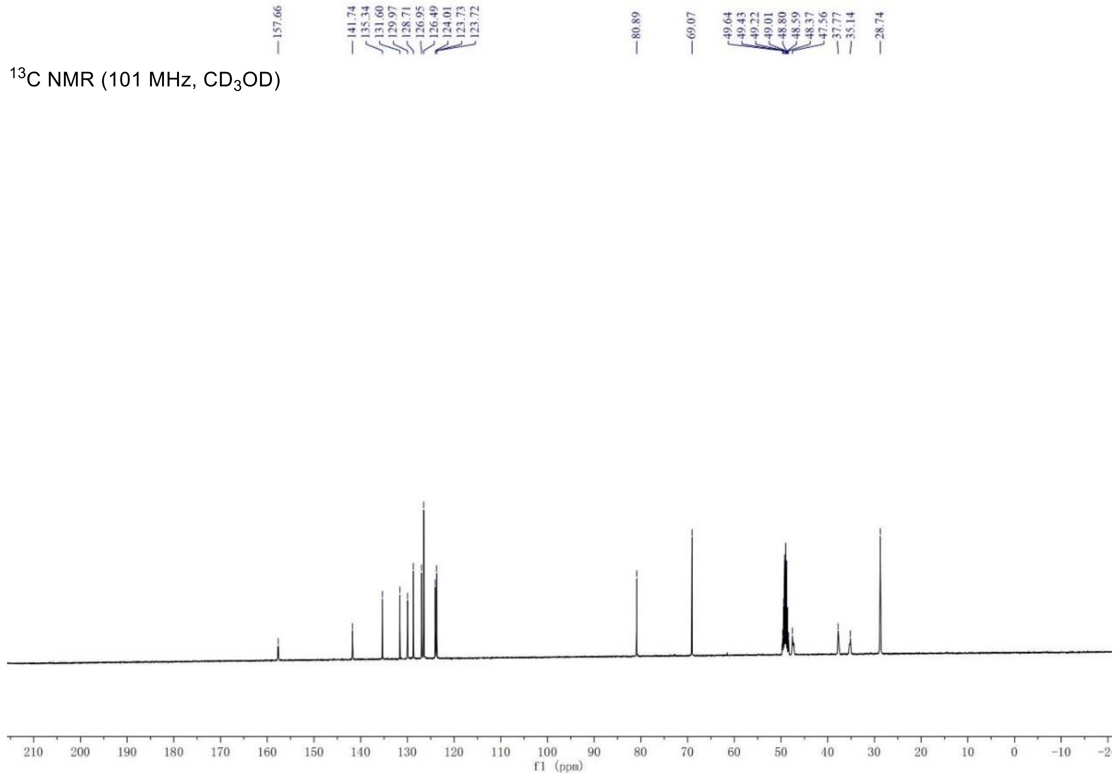

Supplementary Figure 80. ^{13}C NMR spectra of **2ab**

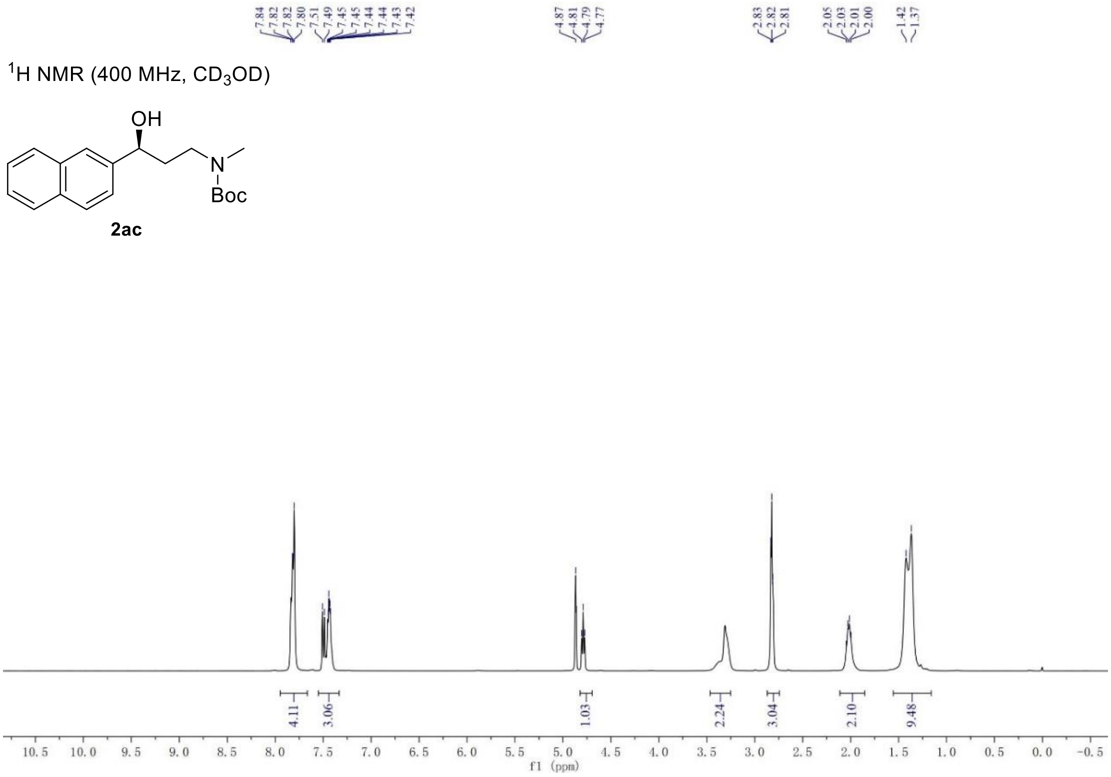

Supplementary Figure 81. ^{1}H NMR spectra of **2ac**

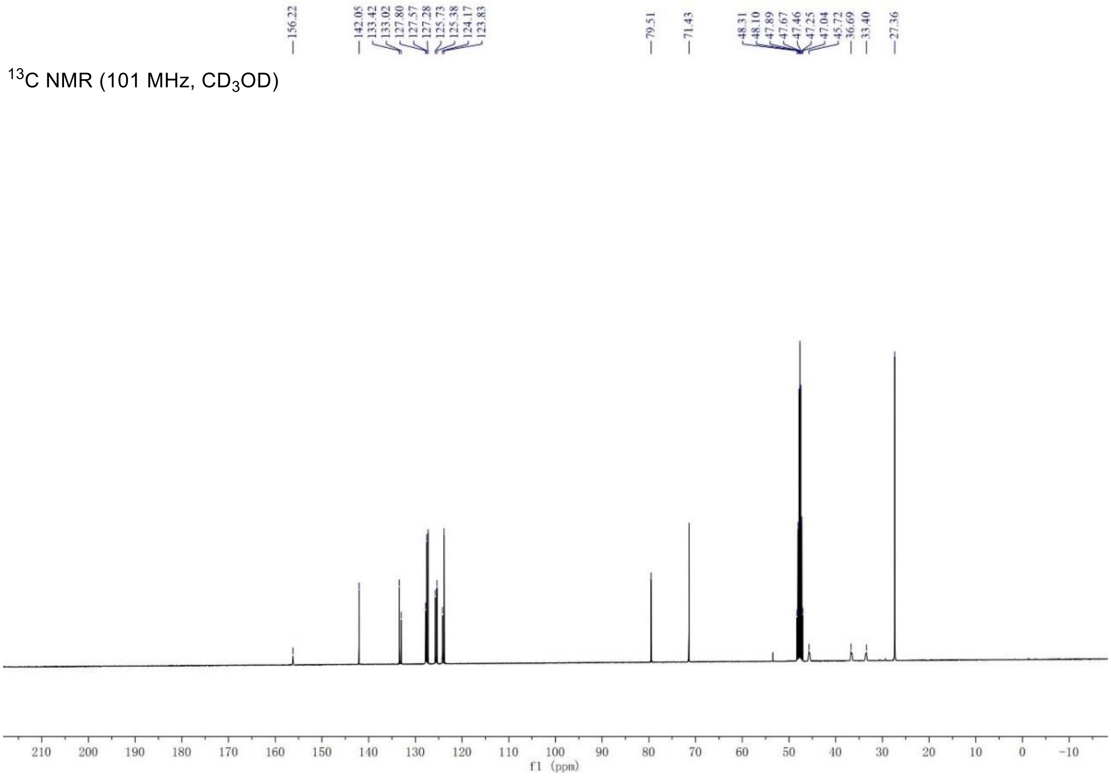

Supplementary Figure 82. ^{13}C NMR spectra of **2ac**

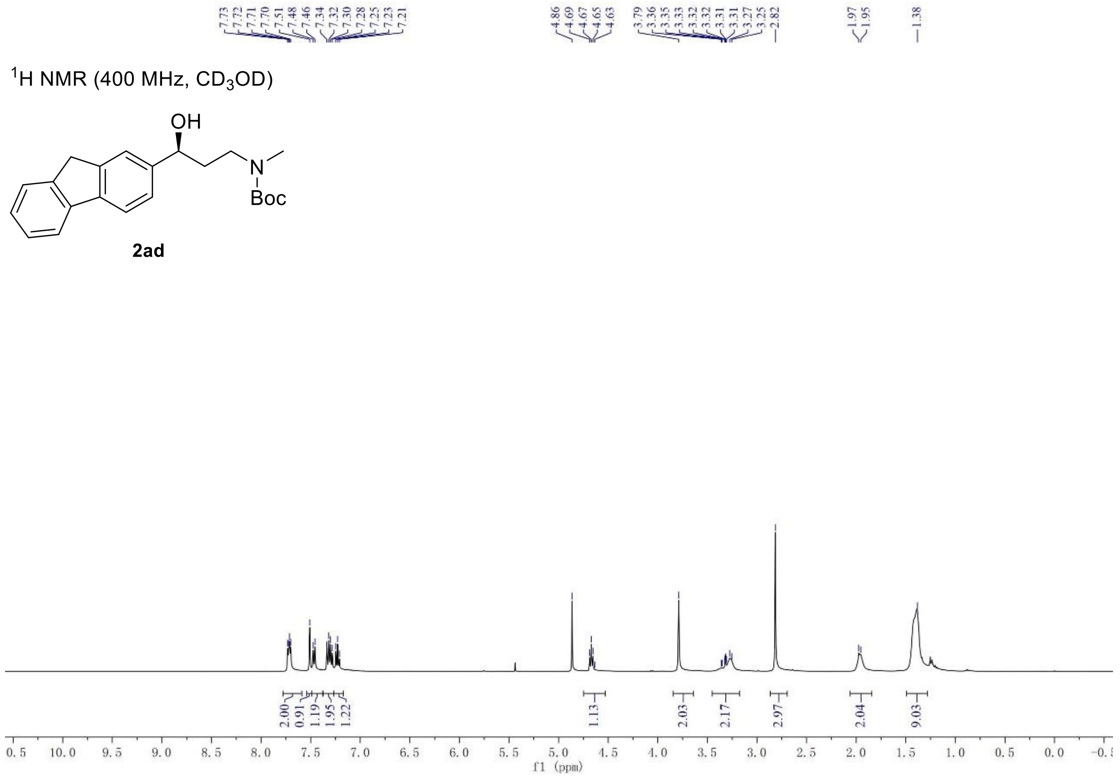

Supplementary Figure 83. ^{1}H NMR spectra of **2ad**

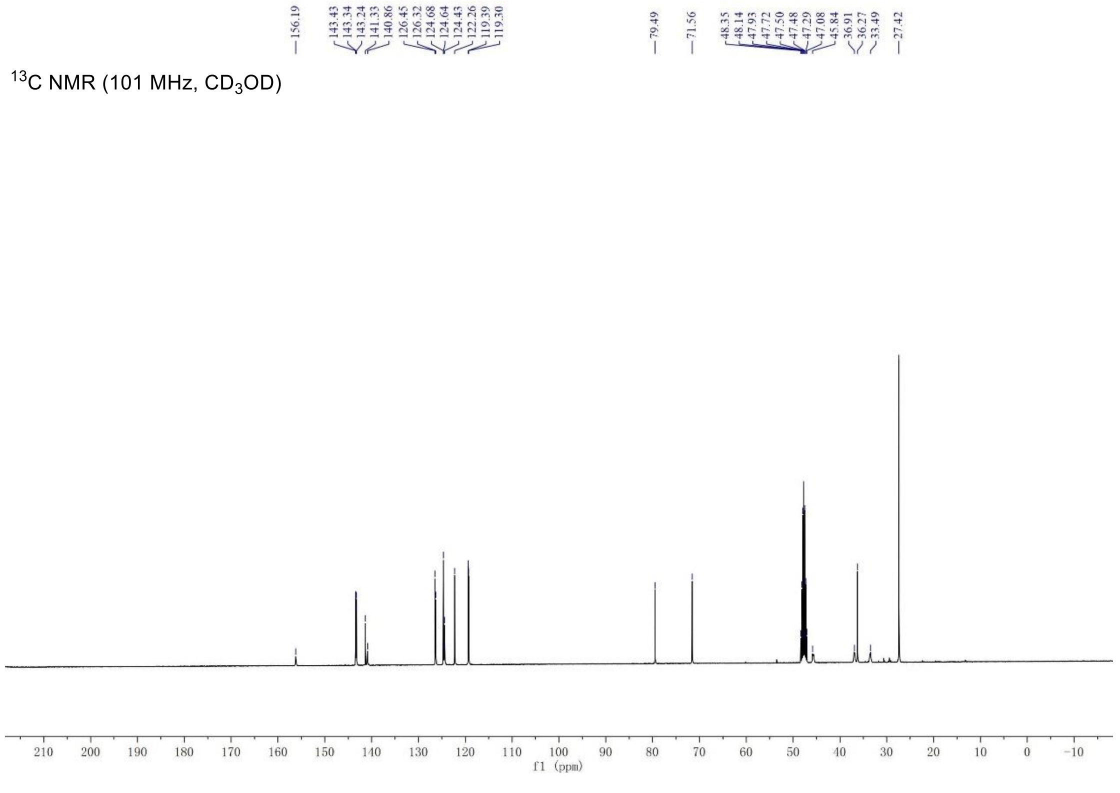

Supplementary Figure 84. ^{13}C NMR spectra of **2ad**

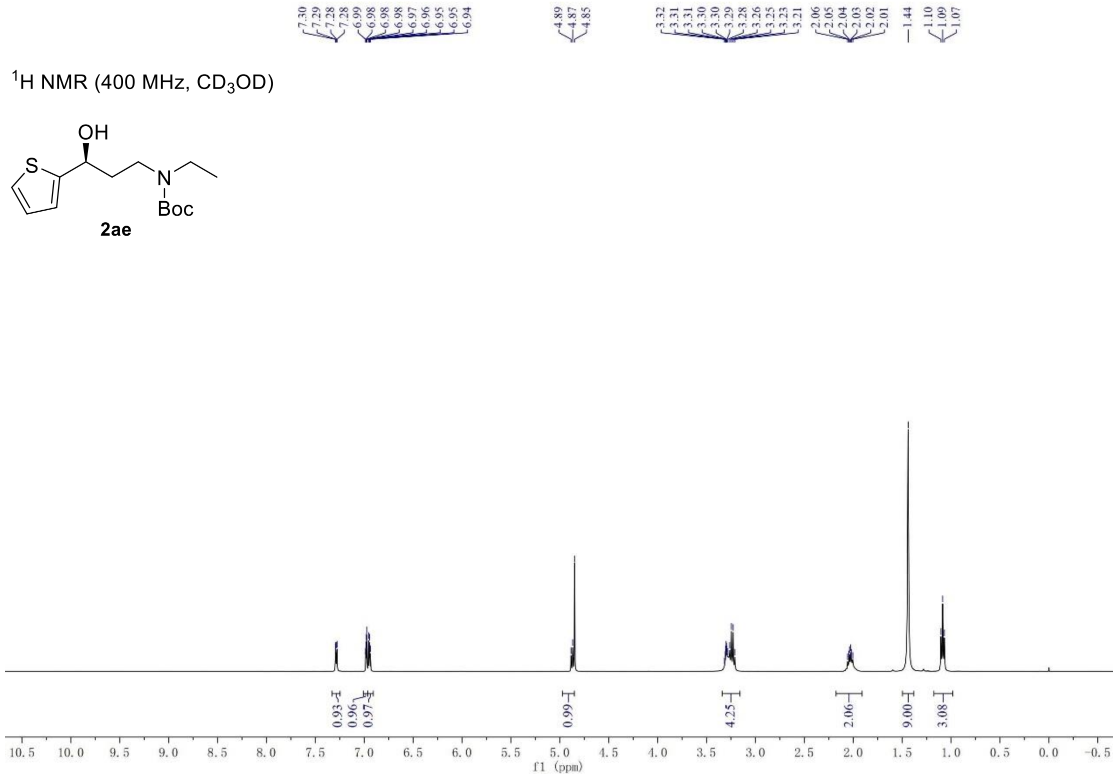

Supplementary Figure 85. ^{1}H NMR spectra of **2ae**

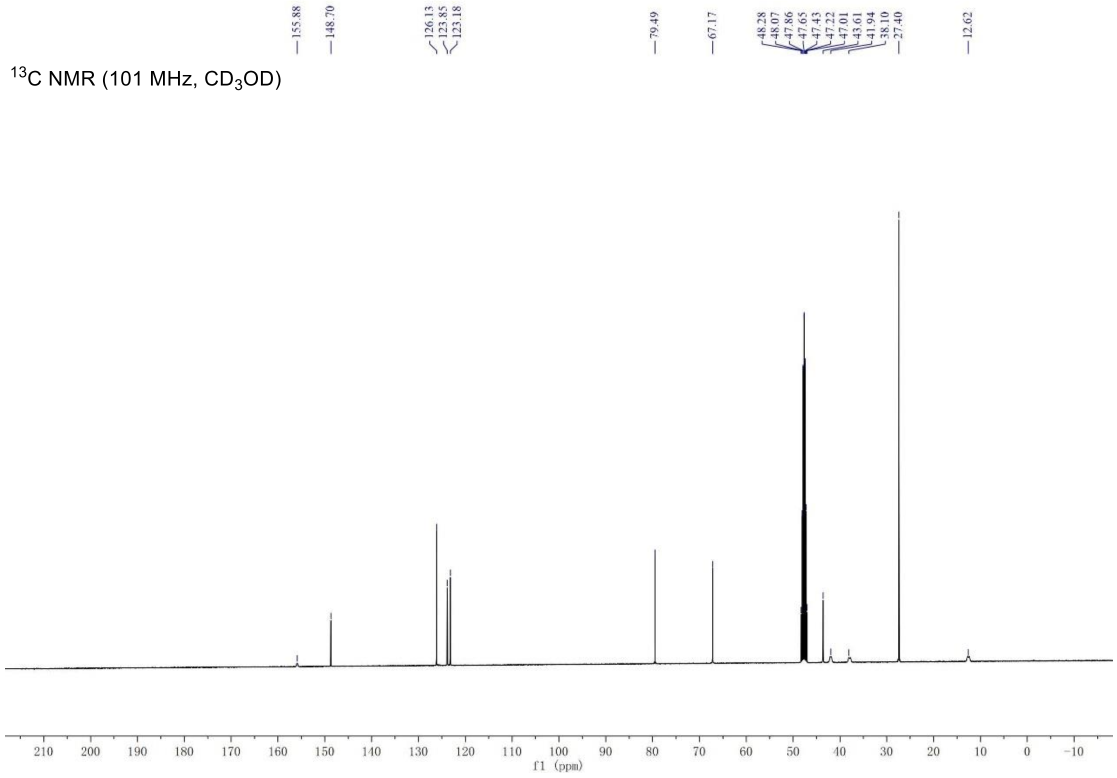

Supplementary Figure 86. ^{13}C NMR spectra of **2ae**

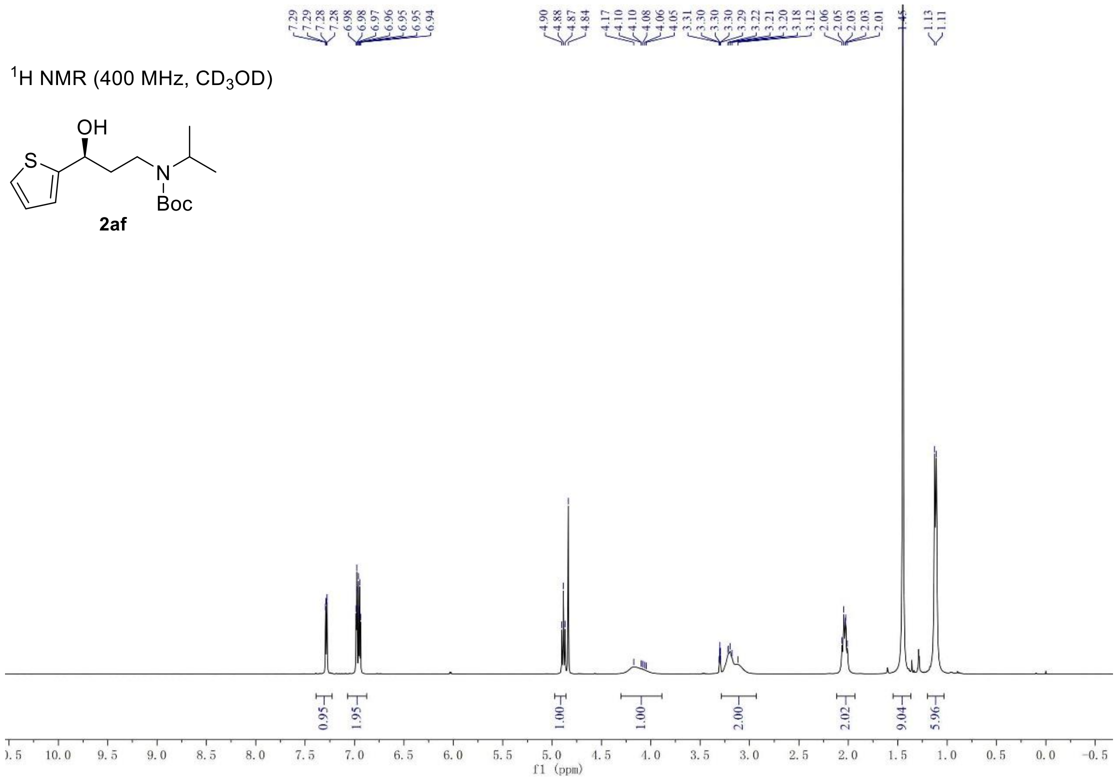

Supplementary Figure 87. ^{1}H NMR spectra of **2af**

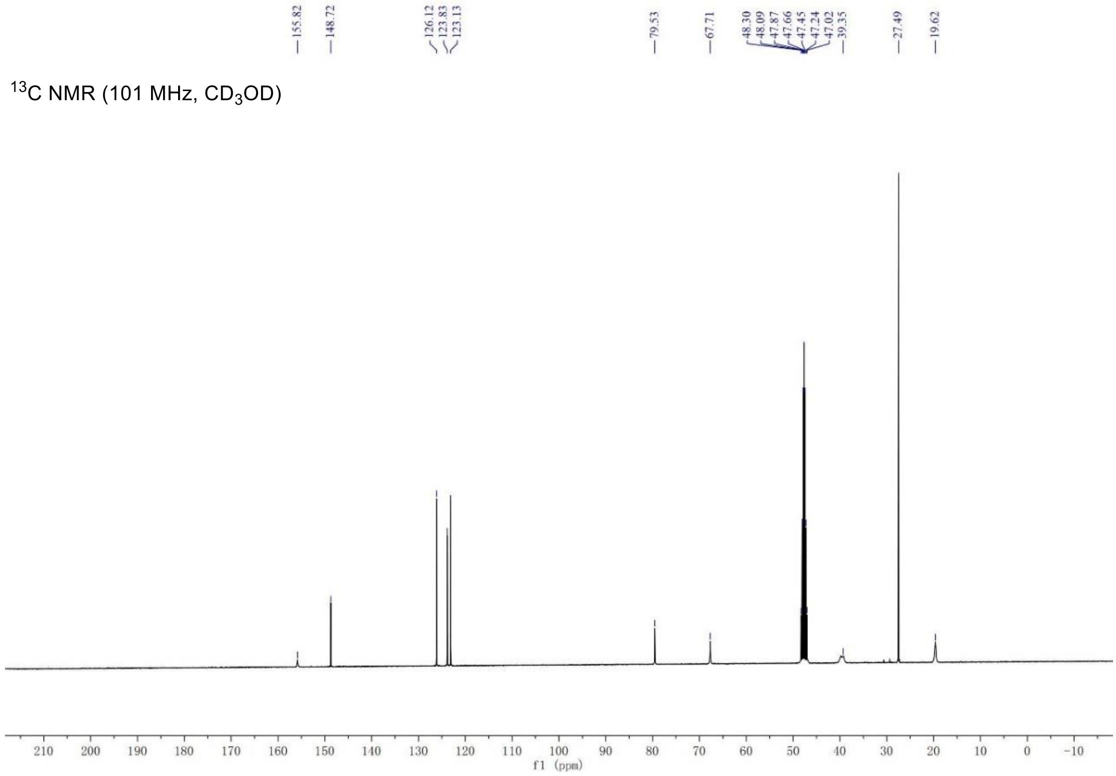

Supplementary Figure 88. ^{13}C NMR spectra of **2af**

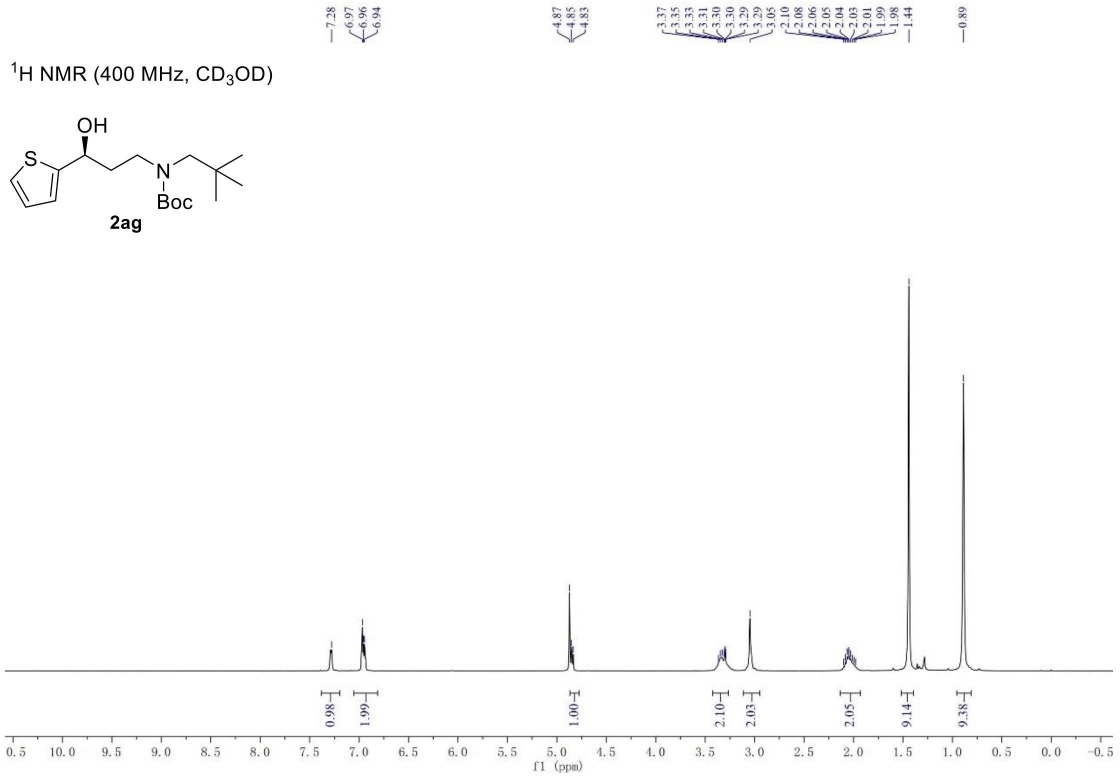

Supplementary Figure 89. ^{1}H NMR spectra of **2ag**

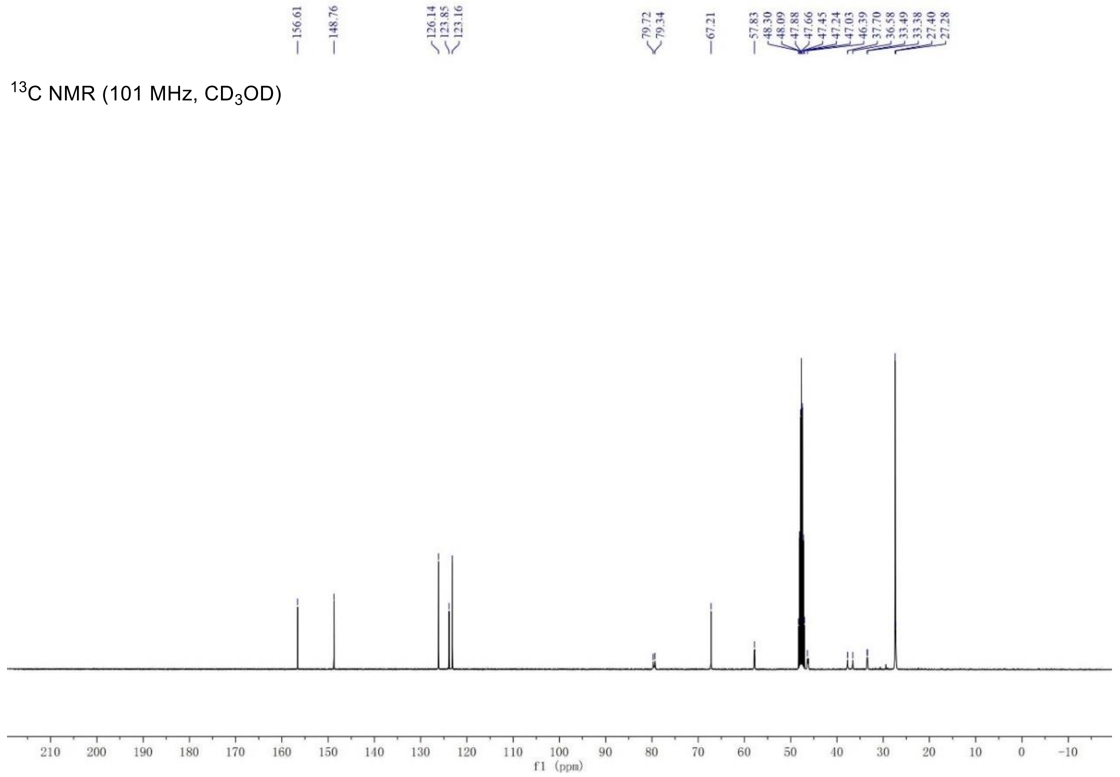

Supplementary Figure 90. ^{13}C NMR spectra of **2ag**

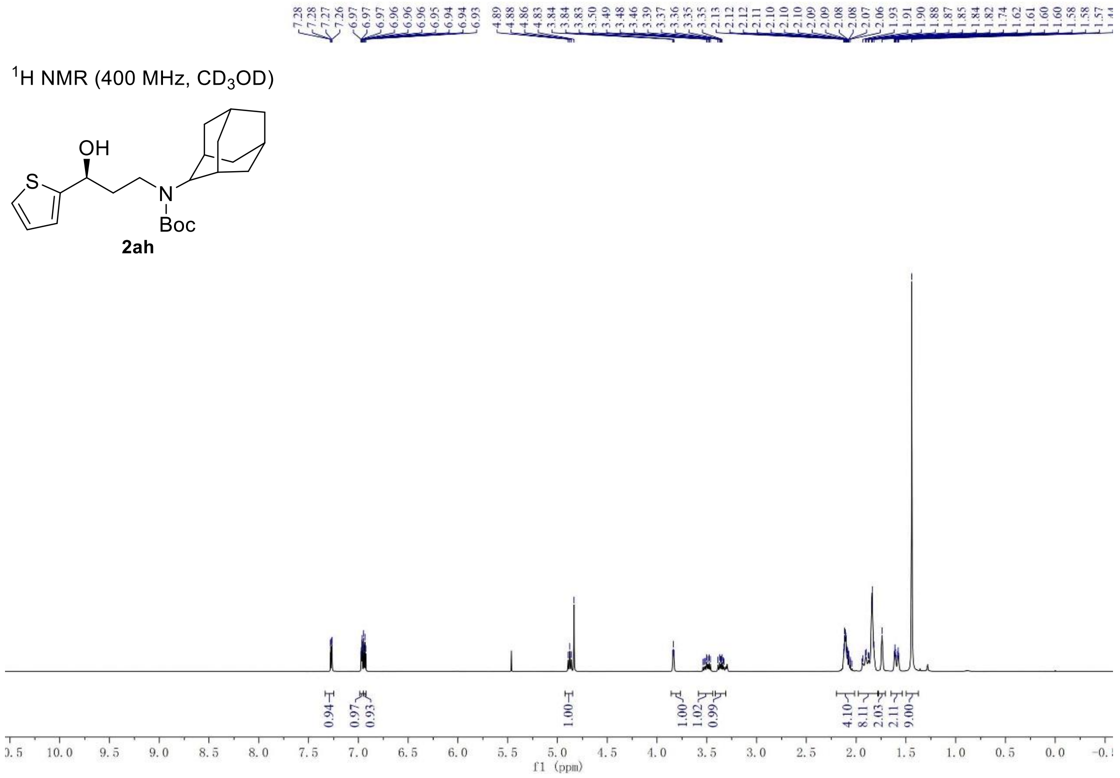

Supplementary Figure 91. ^{1}H NMR spectra of **2ah**

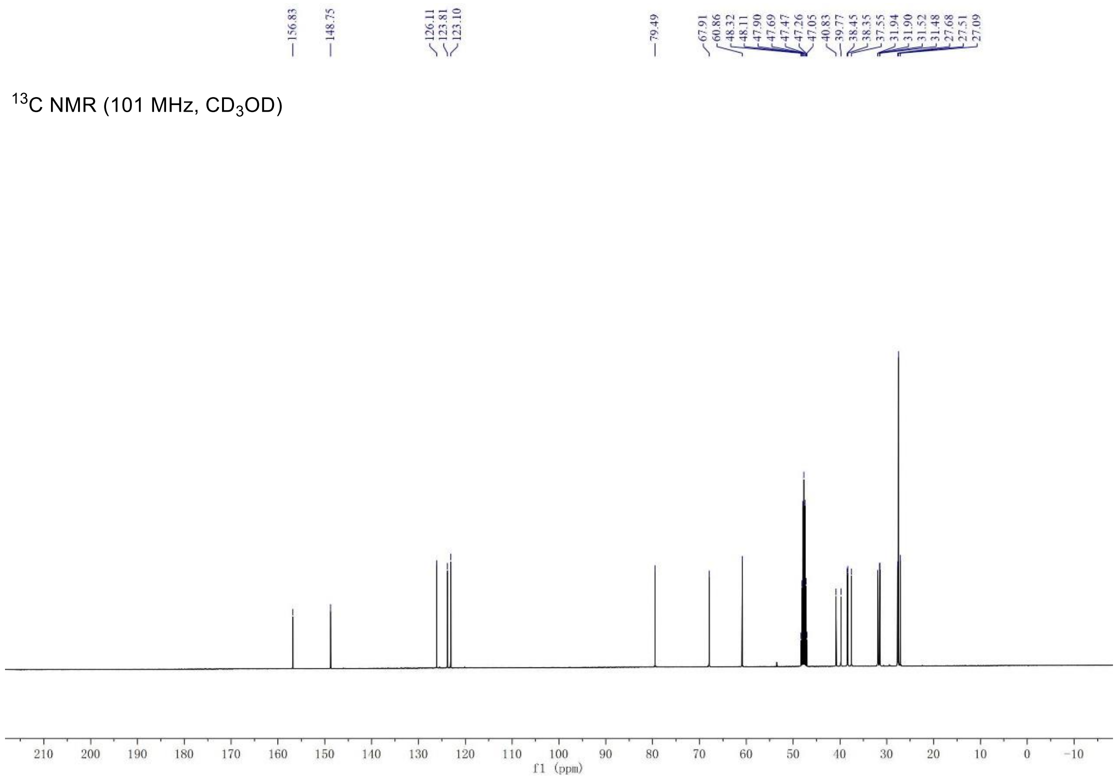

Supplementary Figure 92. ^{13}C NMR spectra of **2ah**

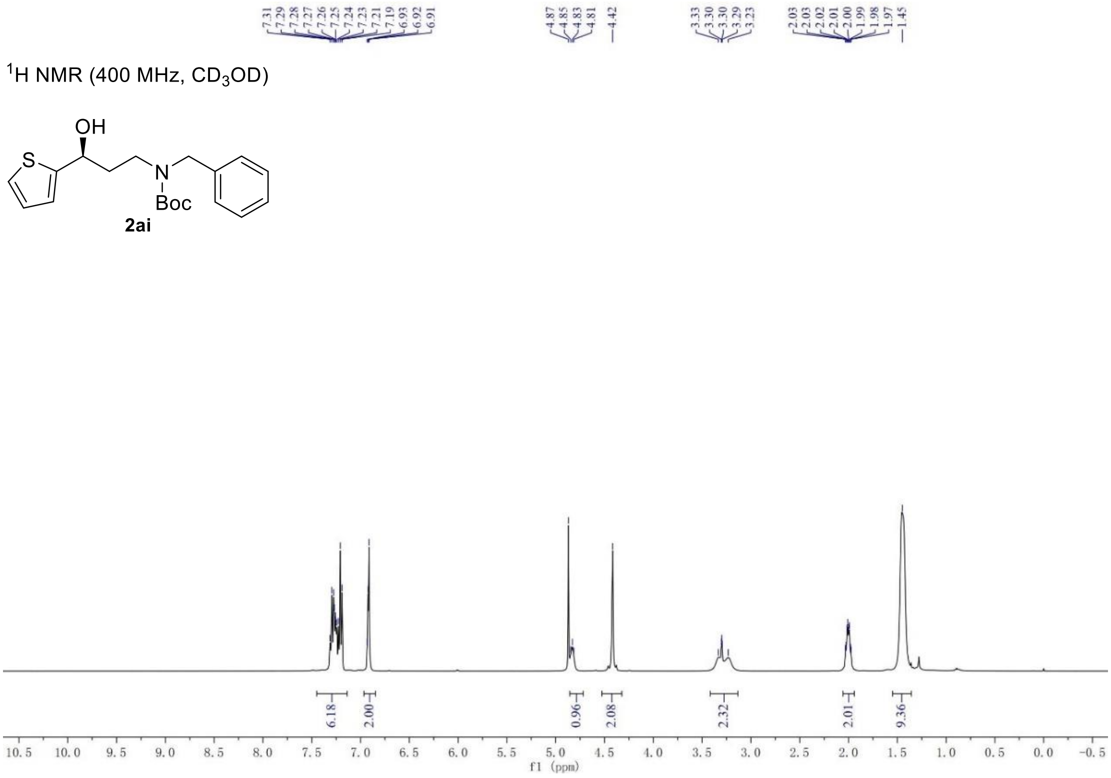

Supplementary Figure 93. ^{1}H NMR spectra of **2ai**

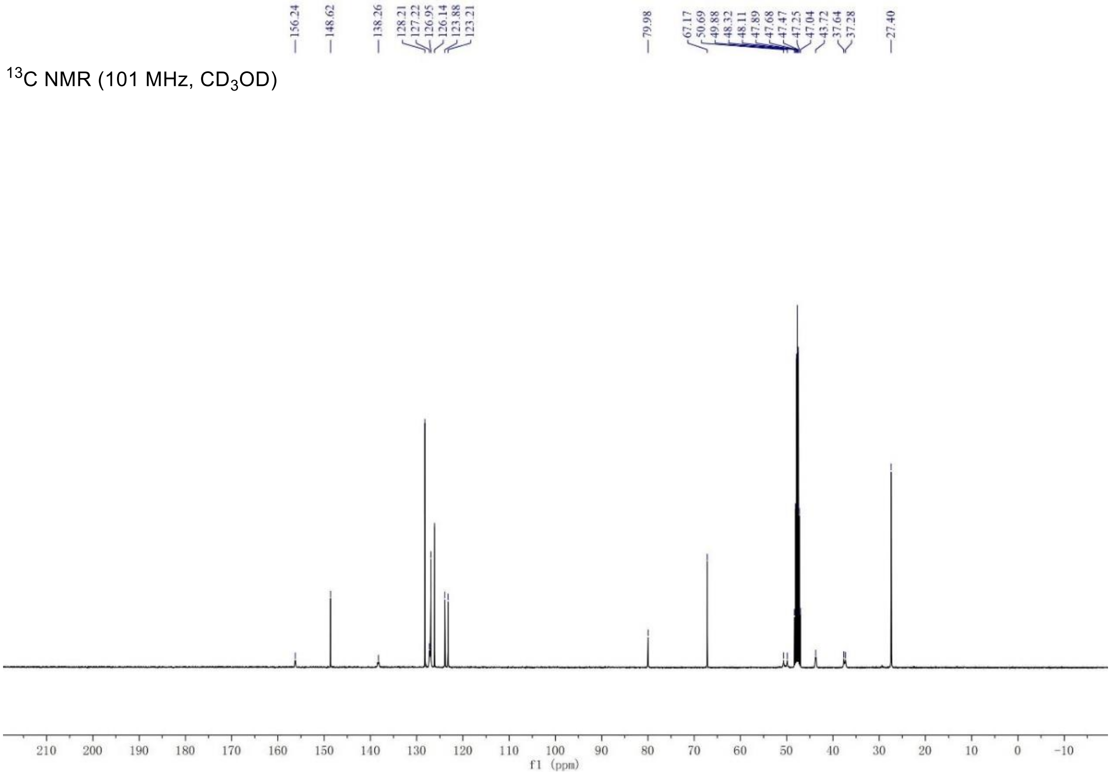

Supplementary Figure 94. ^{13}C NMR spectra of **2ai**

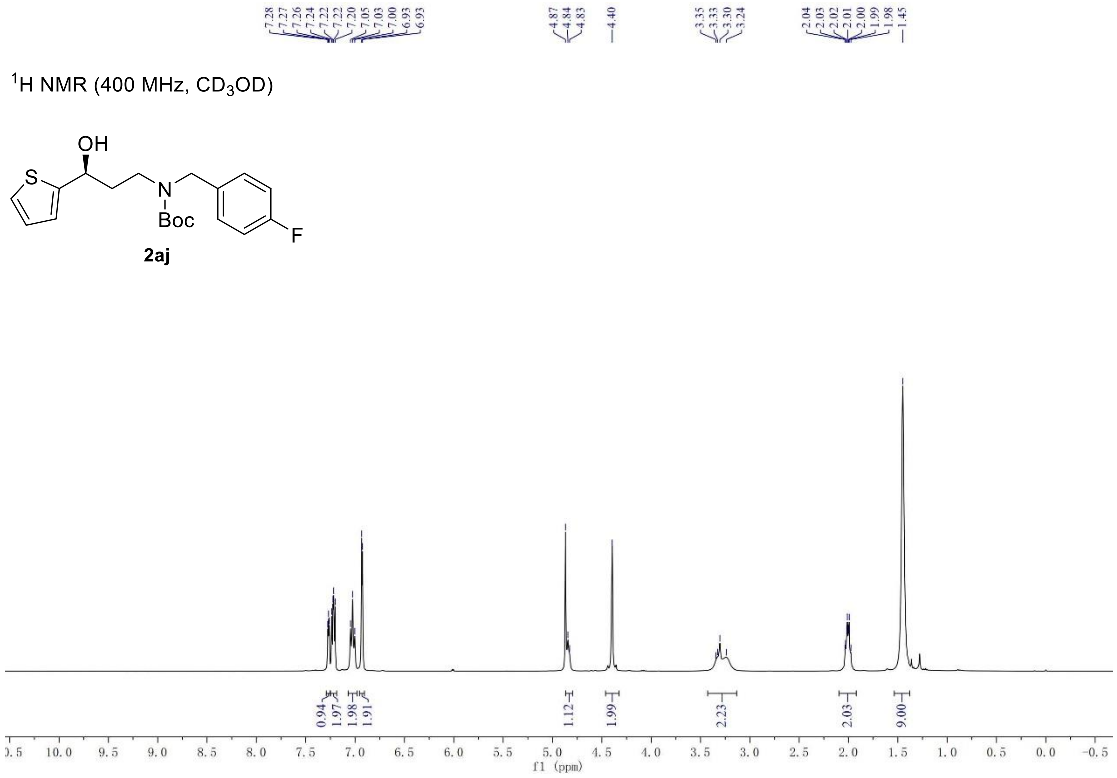

Supplementary Figure 95. ^{1}H NMR spectra of **2aj**

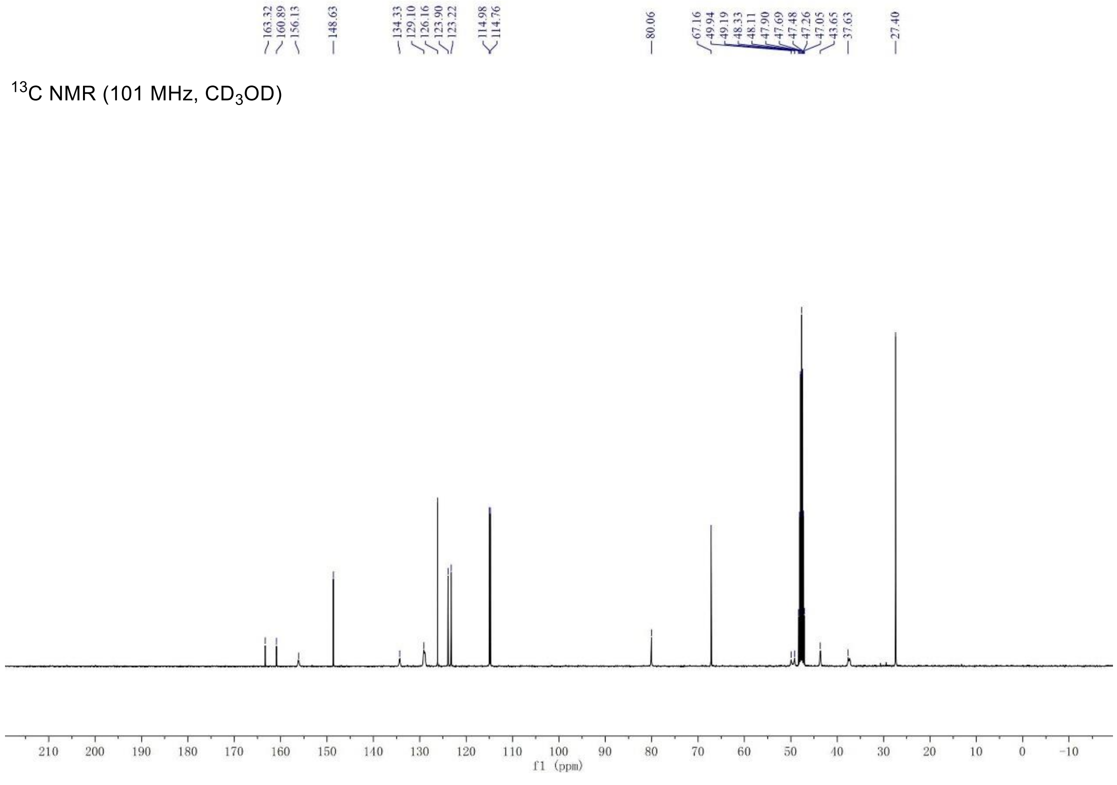

Supplementary Figure 96. ^{13}C NMR spectra of **2aj**

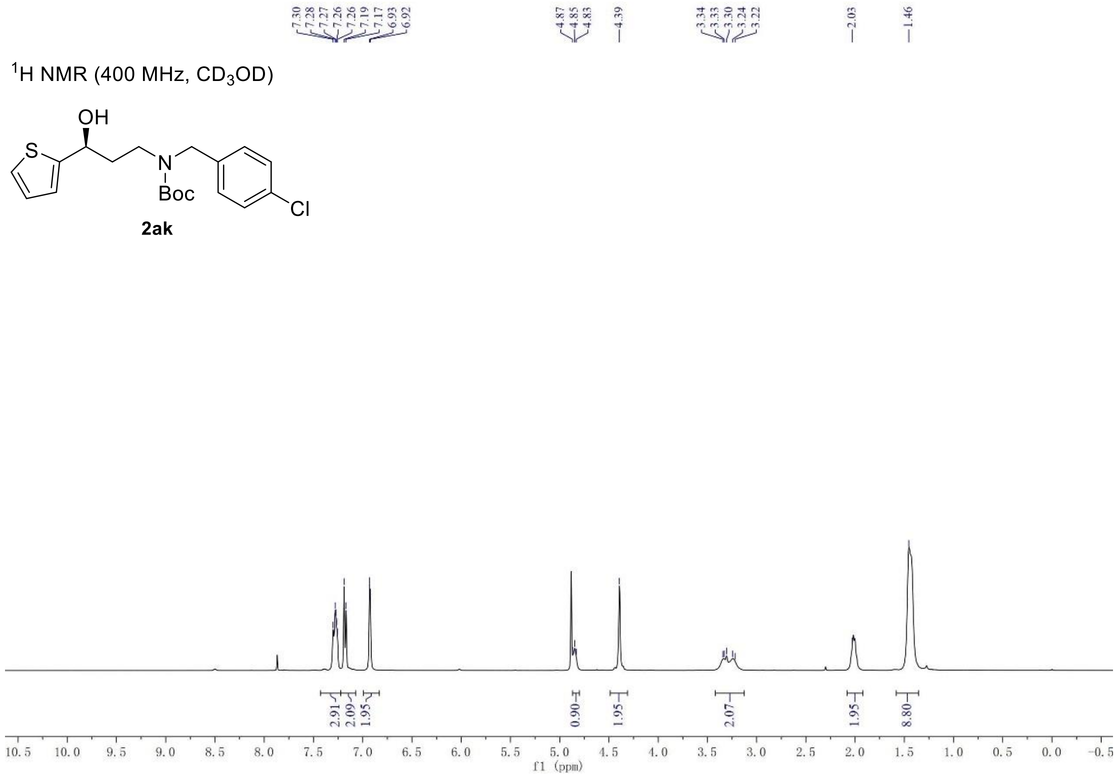

Supplementary Figure 97. ^{1}H NMR spectra of **2ak**

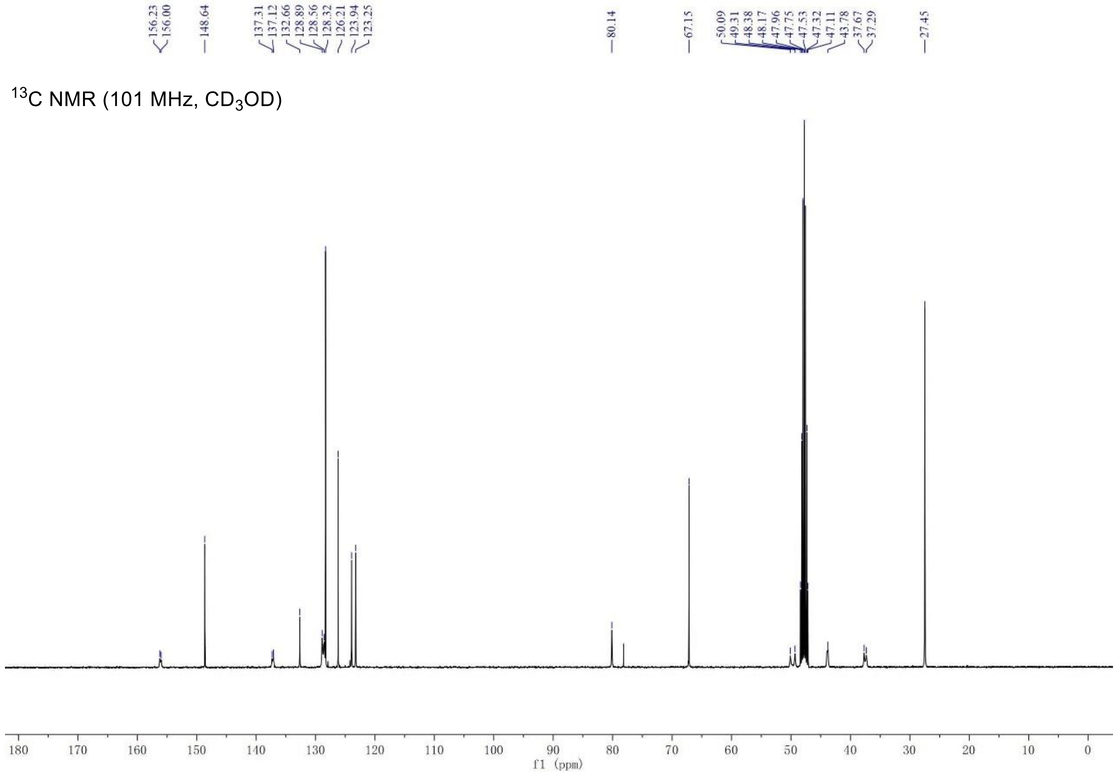

Supplementary Figure 98. ^{13}C NMR spectra of **2ak**

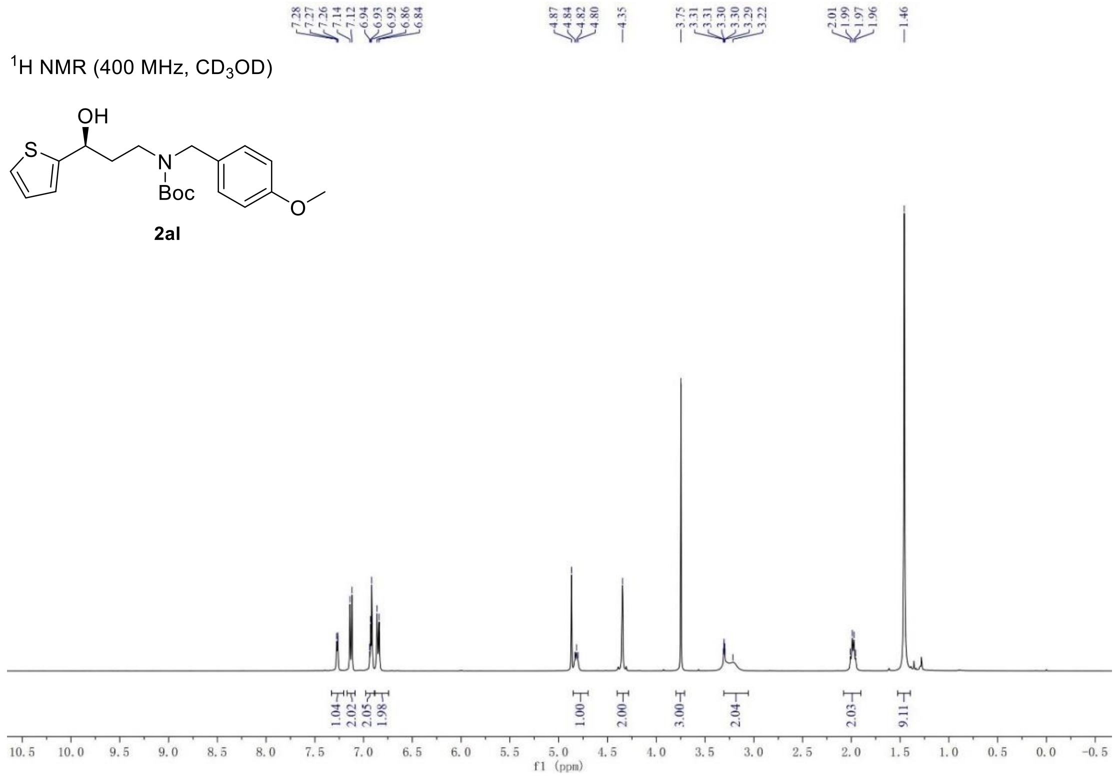

Supplementary Figure 99. ^{1}H NMR spectra of **2al**

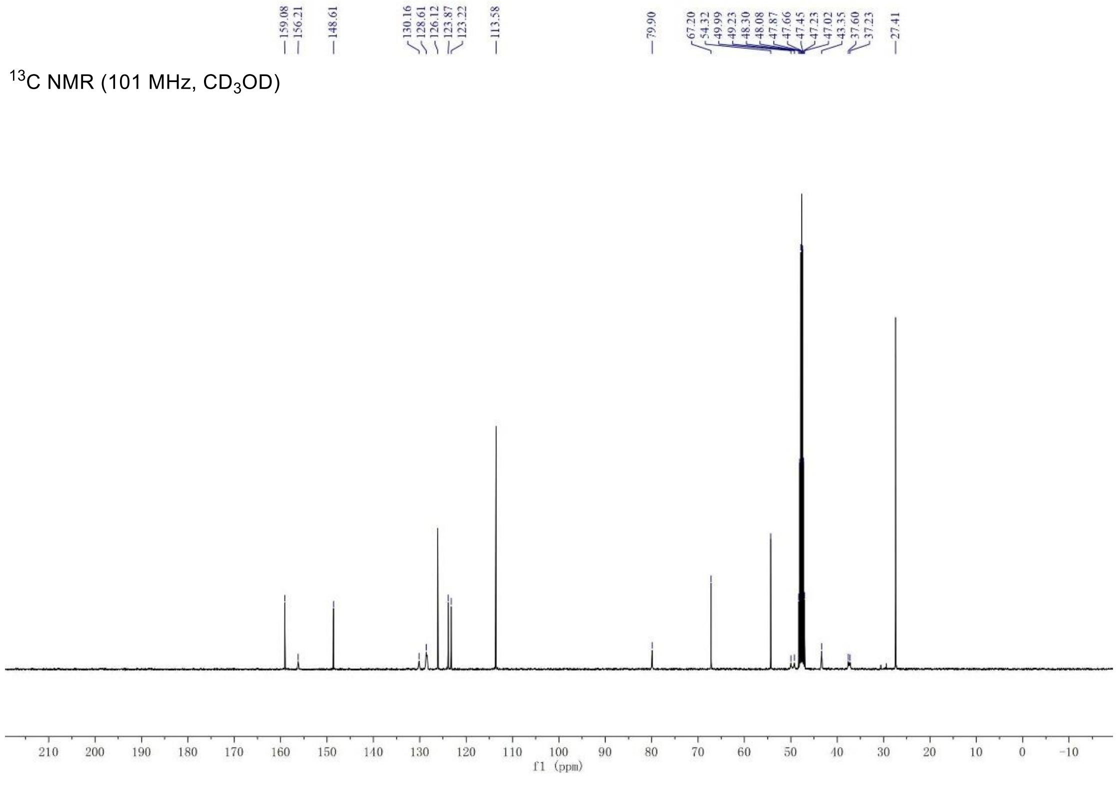

Supplementary Figure 100. ^{13}C NMR spectra of **2al**

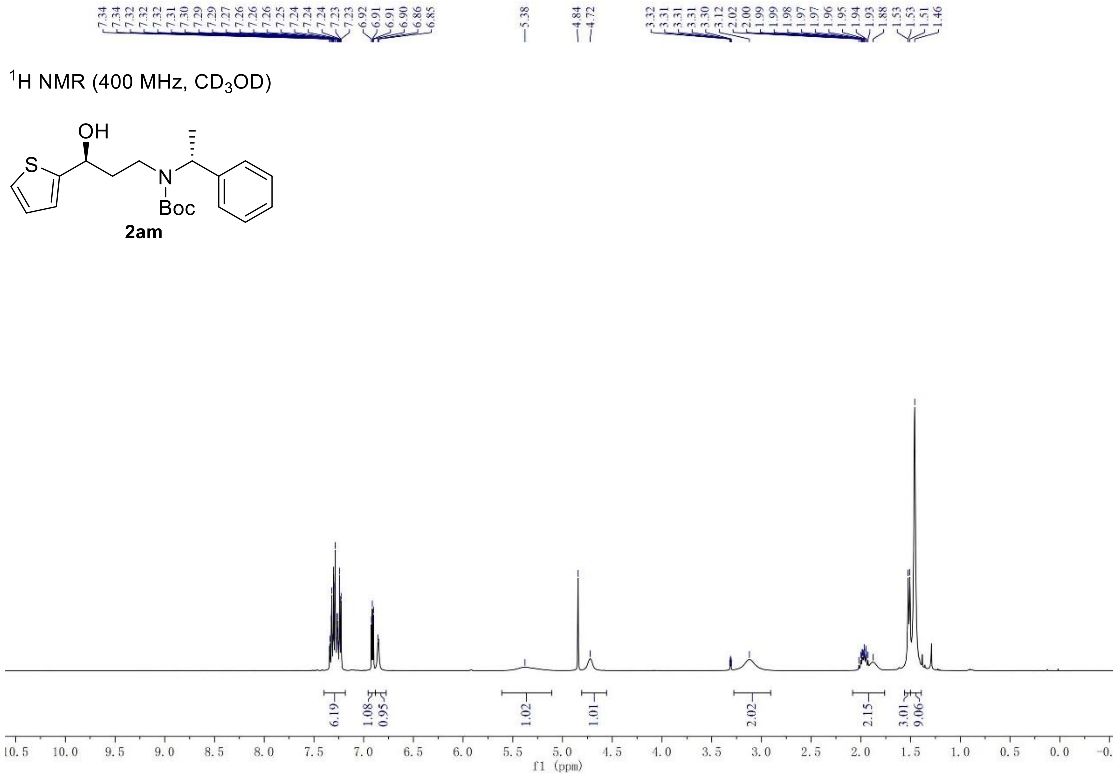

Supplementary Figure 101. ^{1}H NMR spectra of **2am**

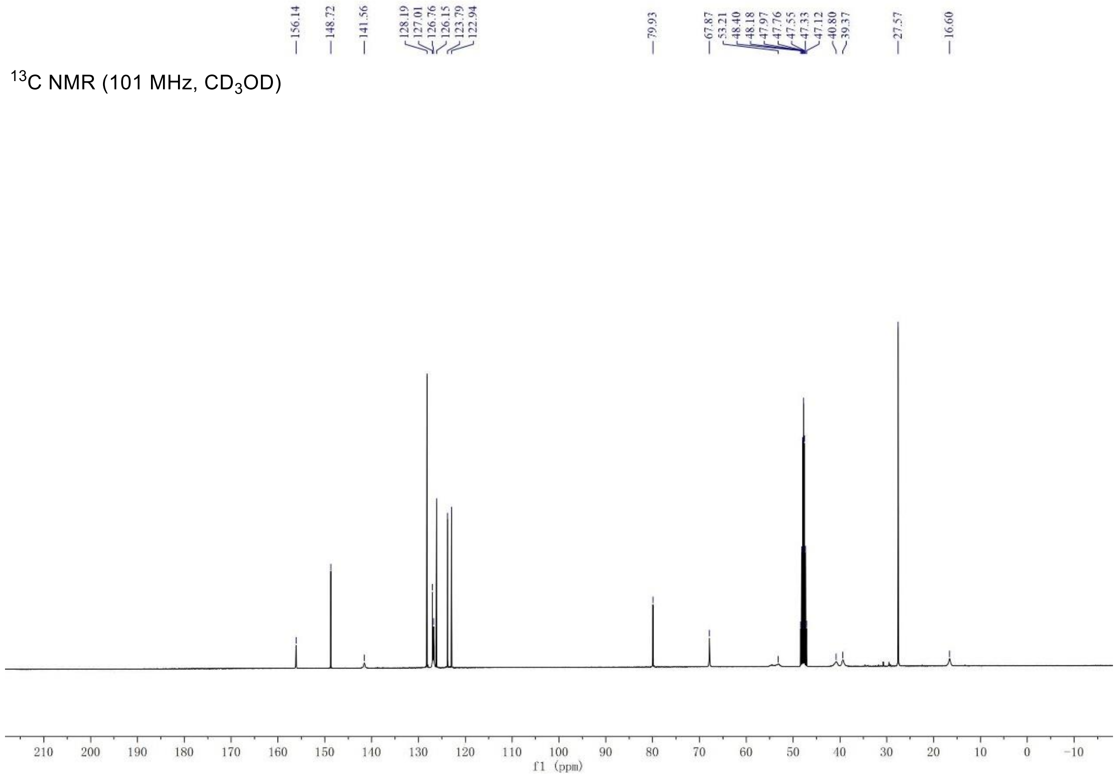

Supplementary Figure 102. ^{13}C NMR spectra of **2am**

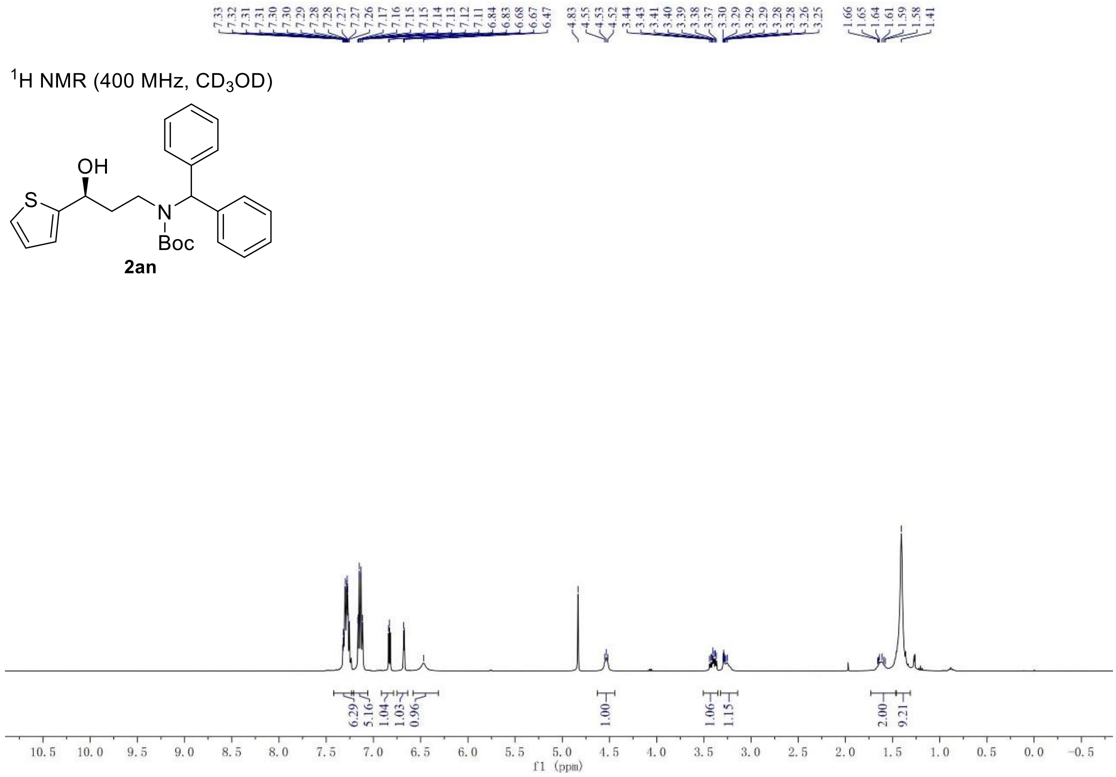

Supplementary Figure 103. ^{1}H NMR spectra of **2an**

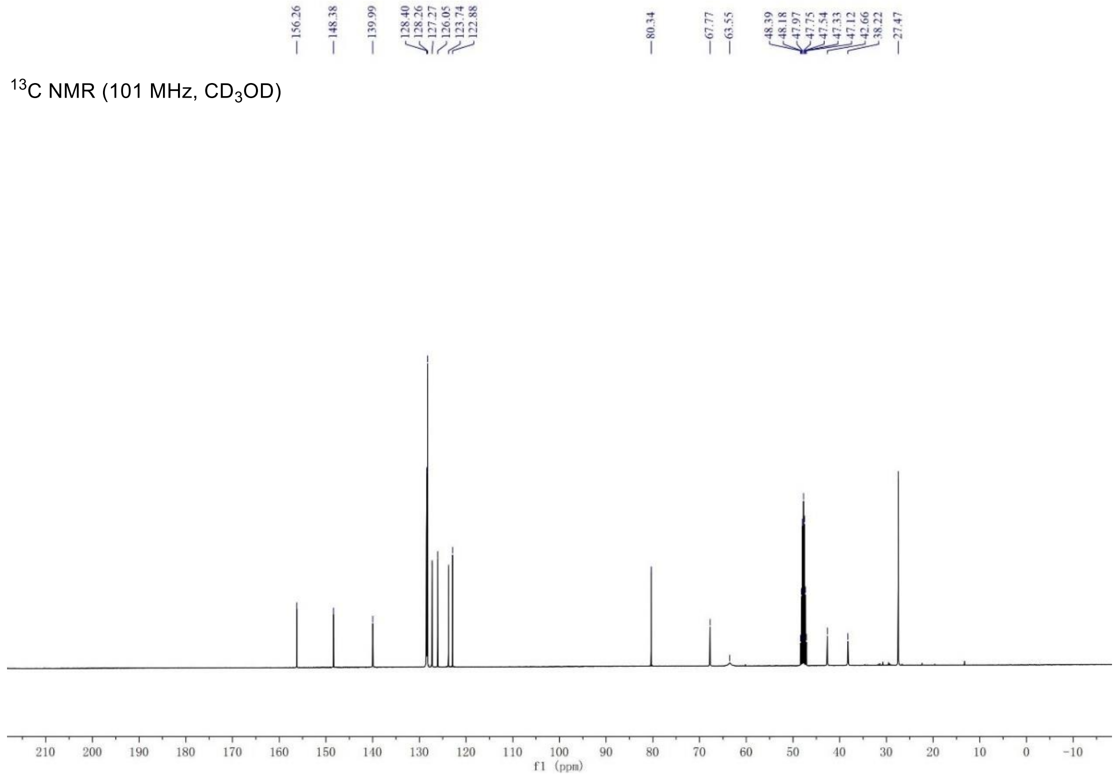

Supplementary Figure 104. ^{13}C NMR spectra of **2an**

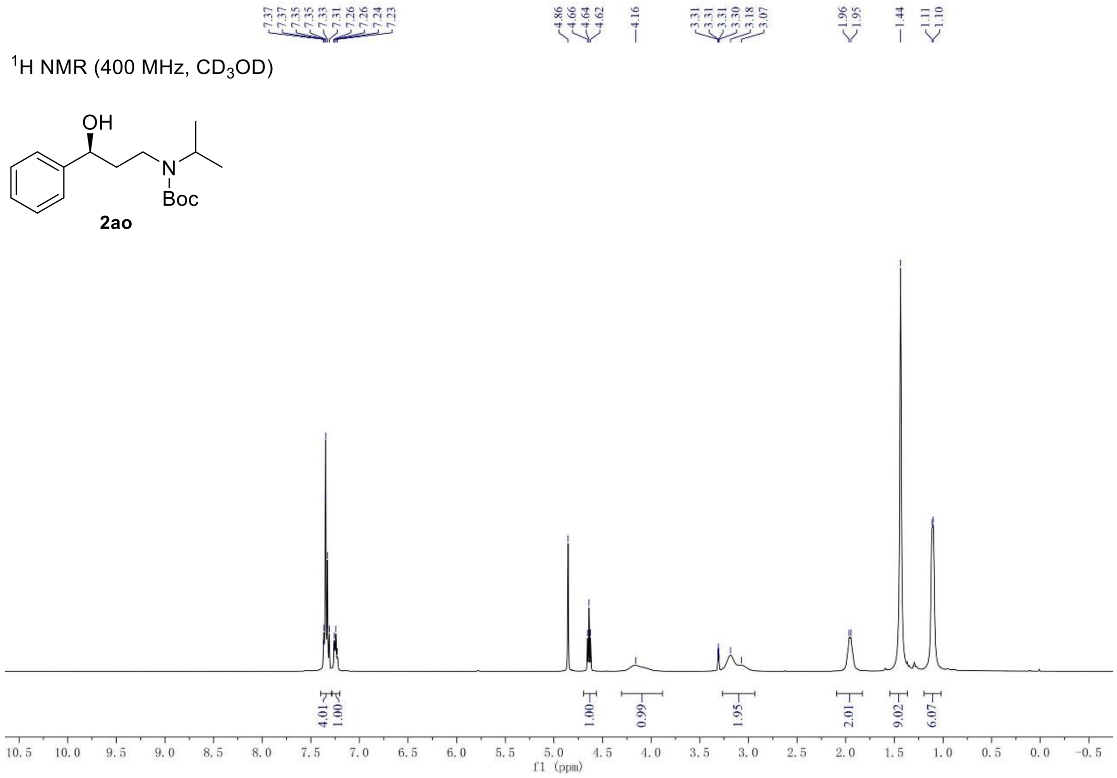

Supplementary Figure 105. ^{1}H NMR spectra of **2ao**

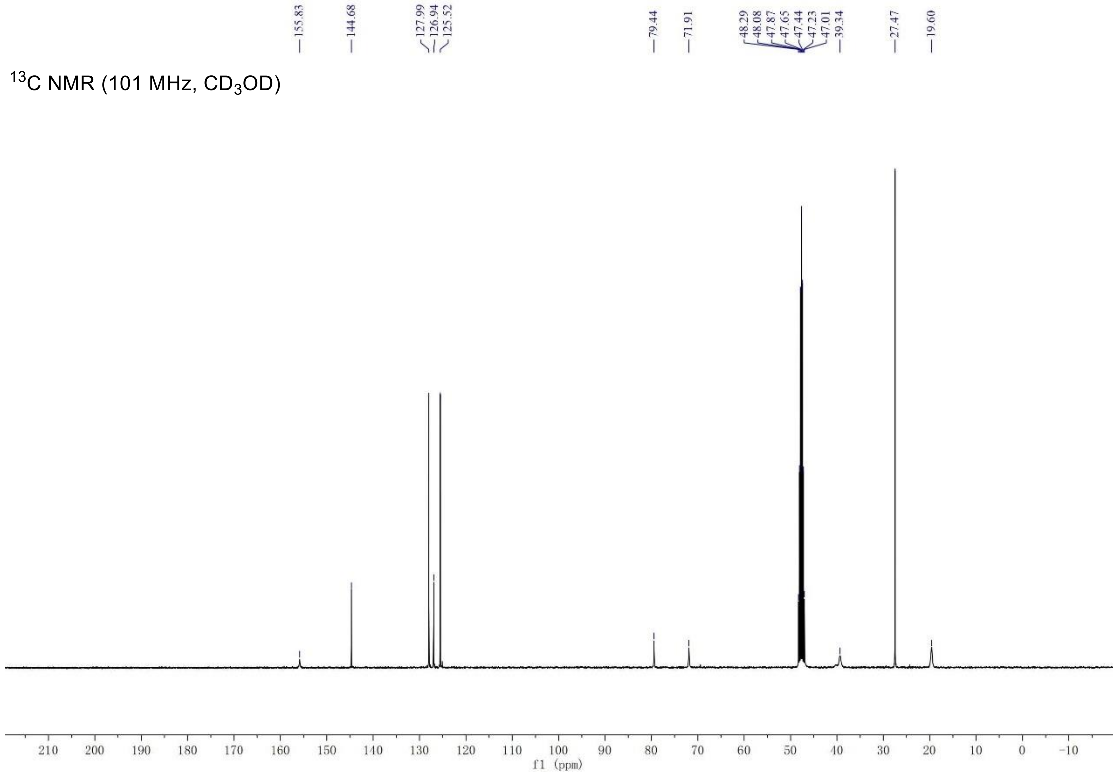

Supplementary Figure 106. ^{13}C NMR spectra of **2ao**

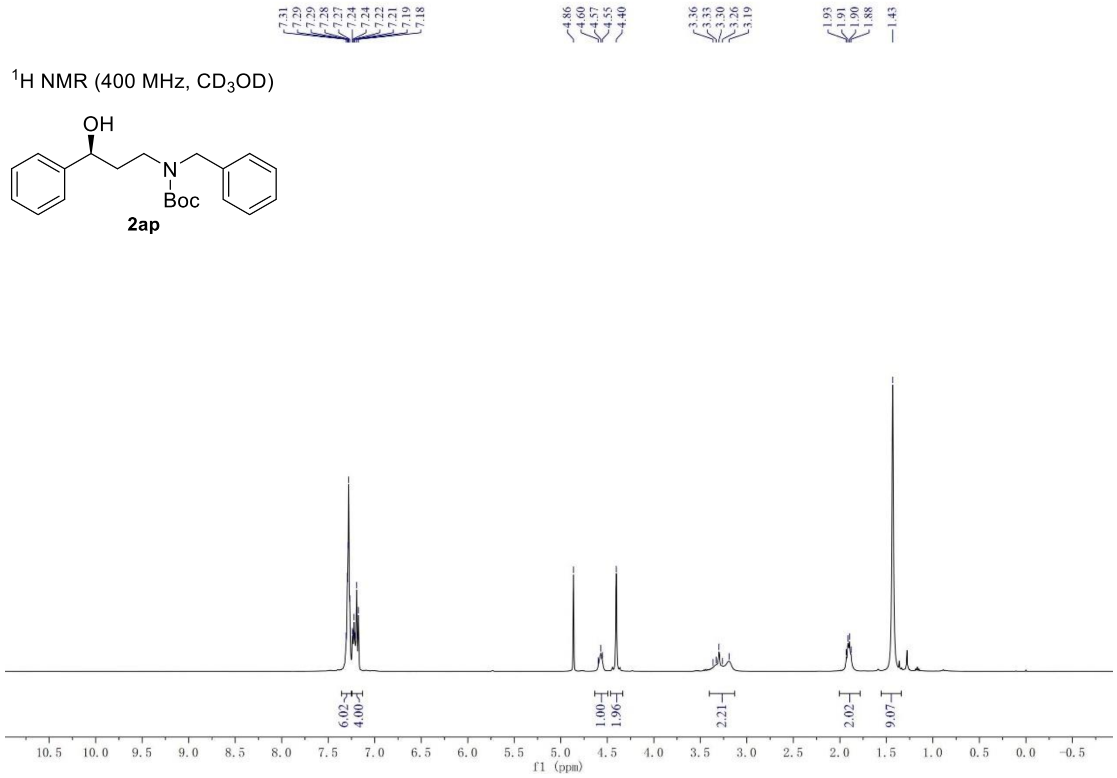

Supplementary Figure 107. ^{1}H NMR spectra of **2ap**

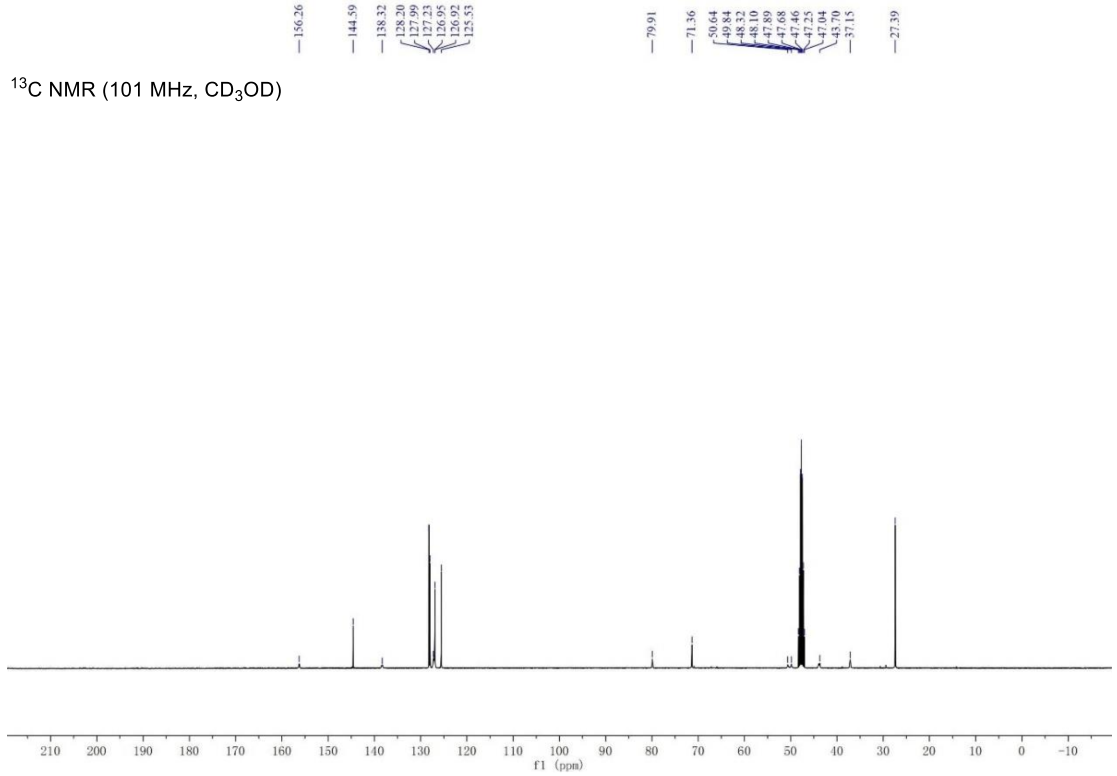

Supplementary Figure 108. ^{13}C NMR spectra of **2ap**

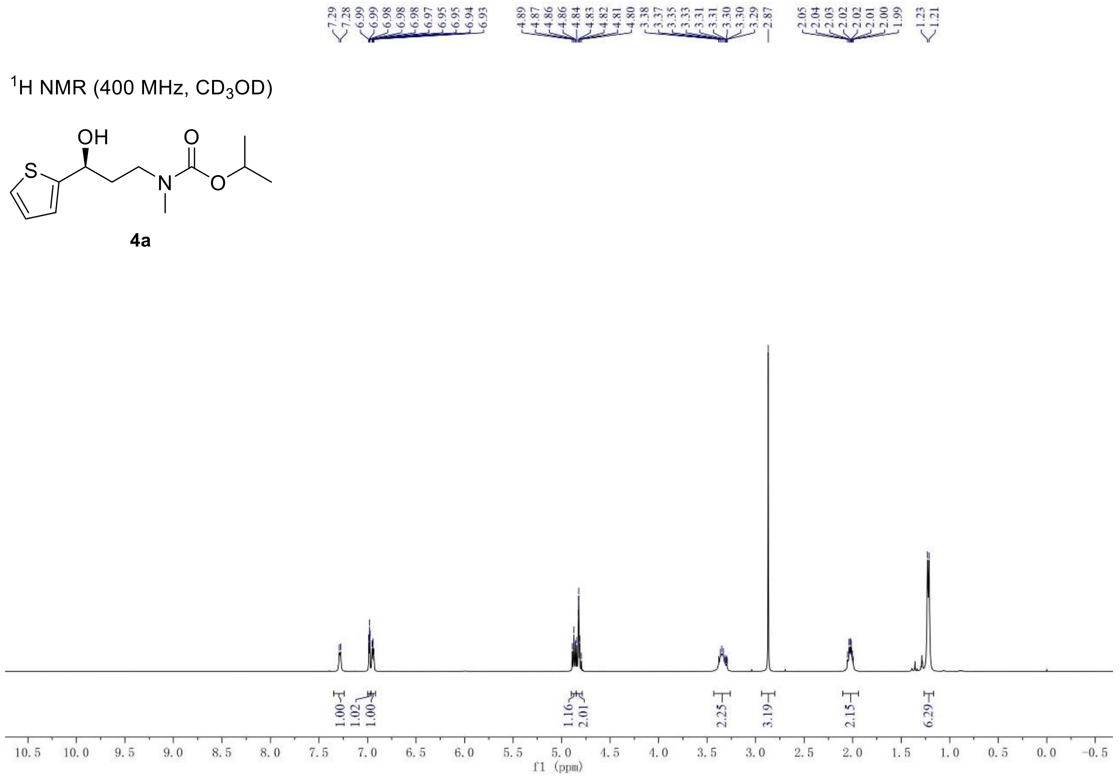

Supplementary Figure 109. ^{1}H NMR spectra of **4a**

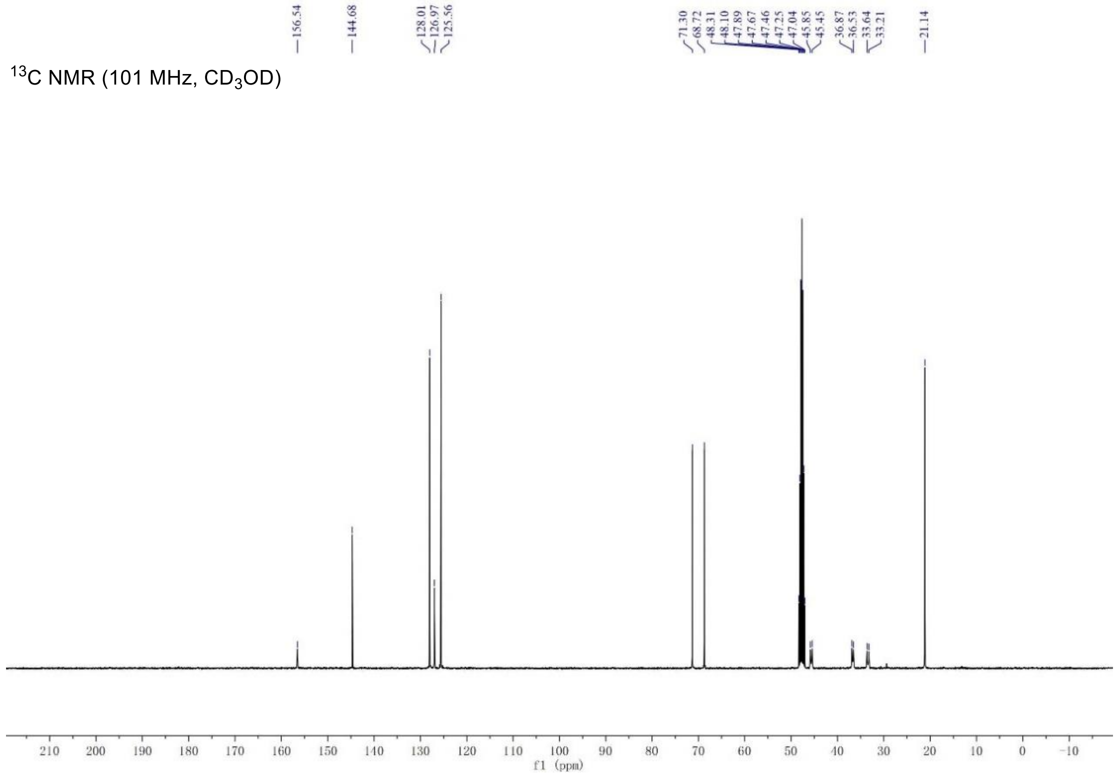

Supplementary Figure 110. ^{13}C NMR spectra of **4a**

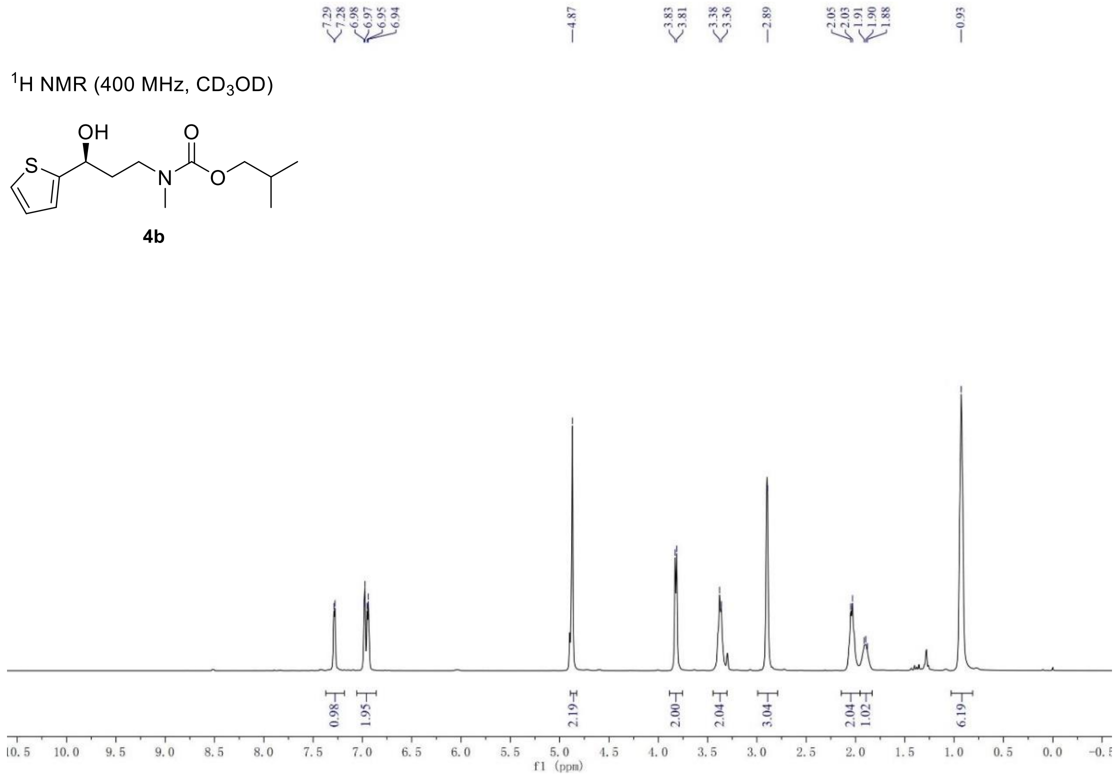

Supplementary Figure 111. ^{1}H NMR spectra of **4b**

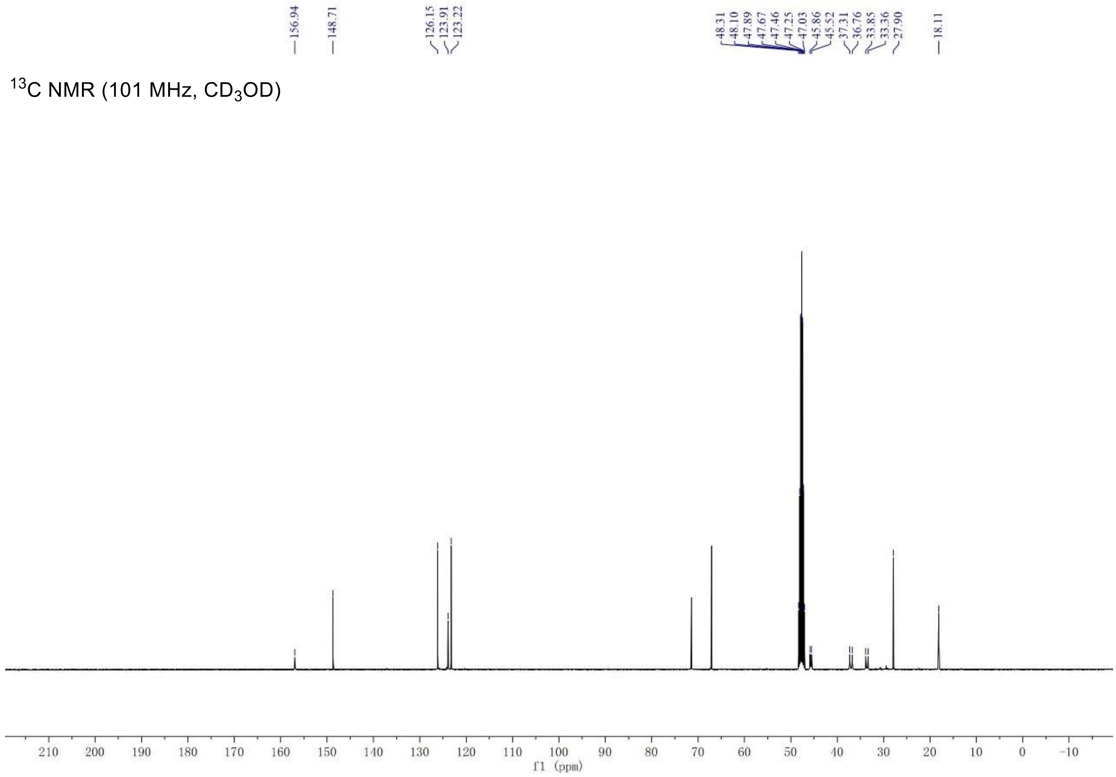

Supplementary Figure 112. ^{13}C NMR spectra of **4b**

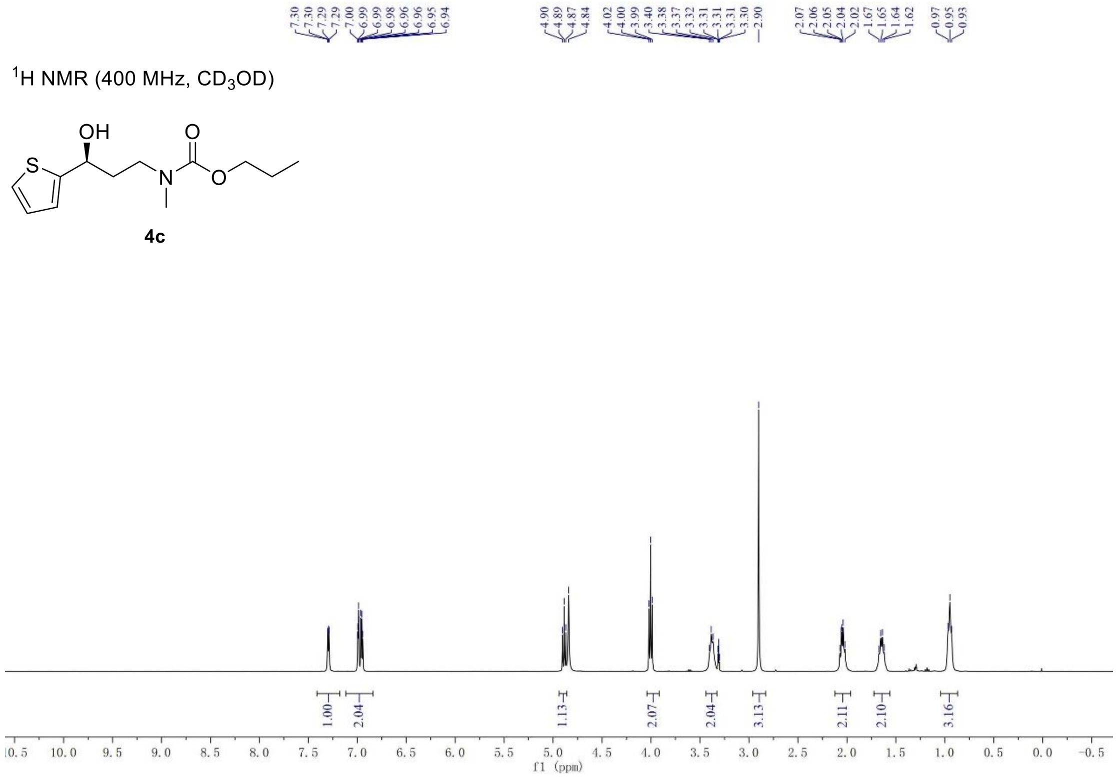

Supplementary Figure 113. ^{1}H NMR spectra of **4c**

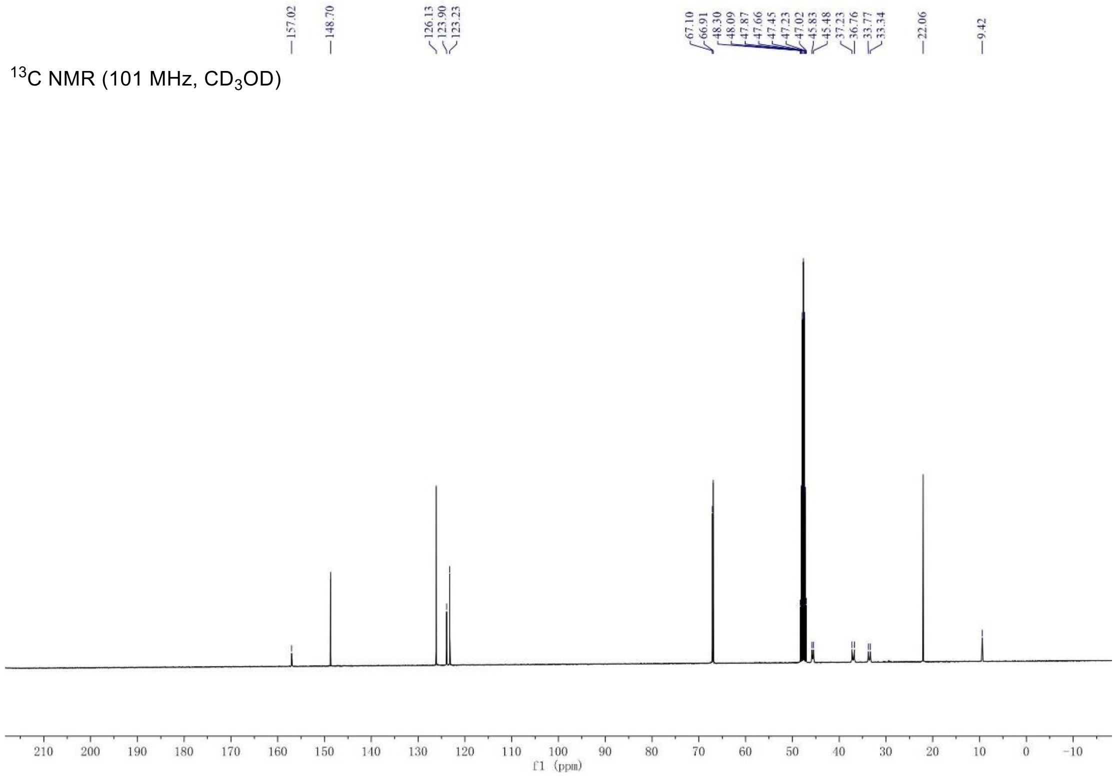

Supplementary Figure 114. ^{13}C NMR spectra of **4c**

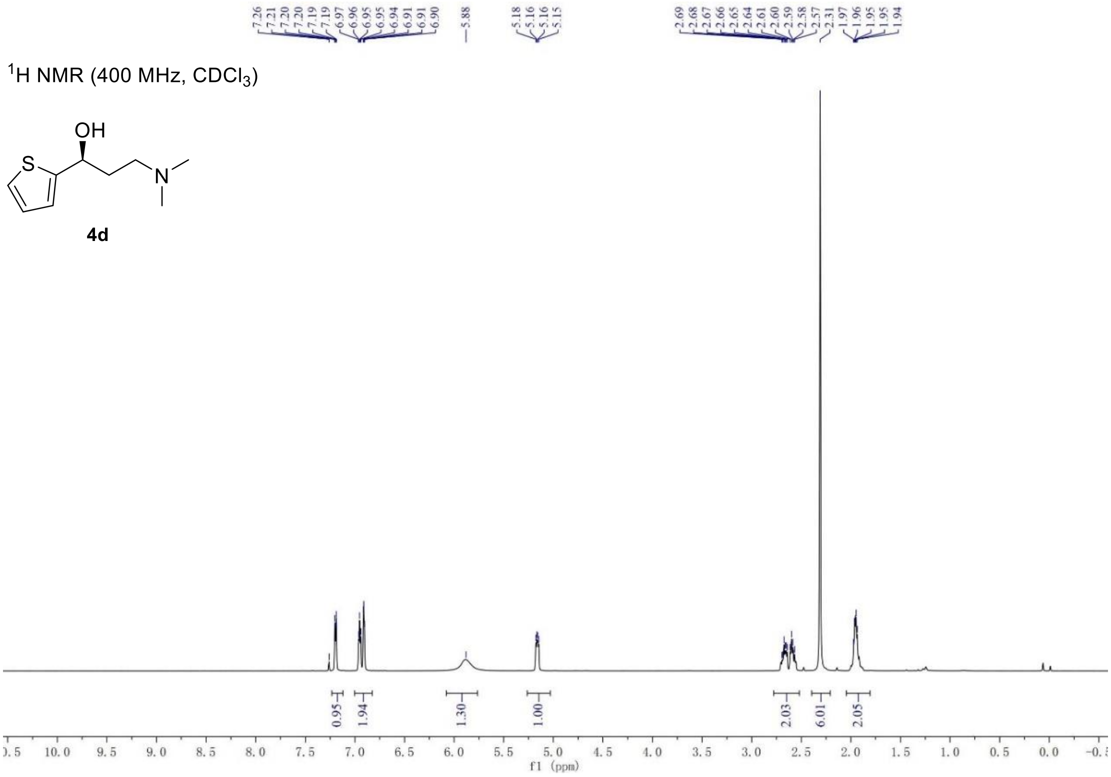

Supplementary Figure 115. ^{1}H NMR spectra of **4d**

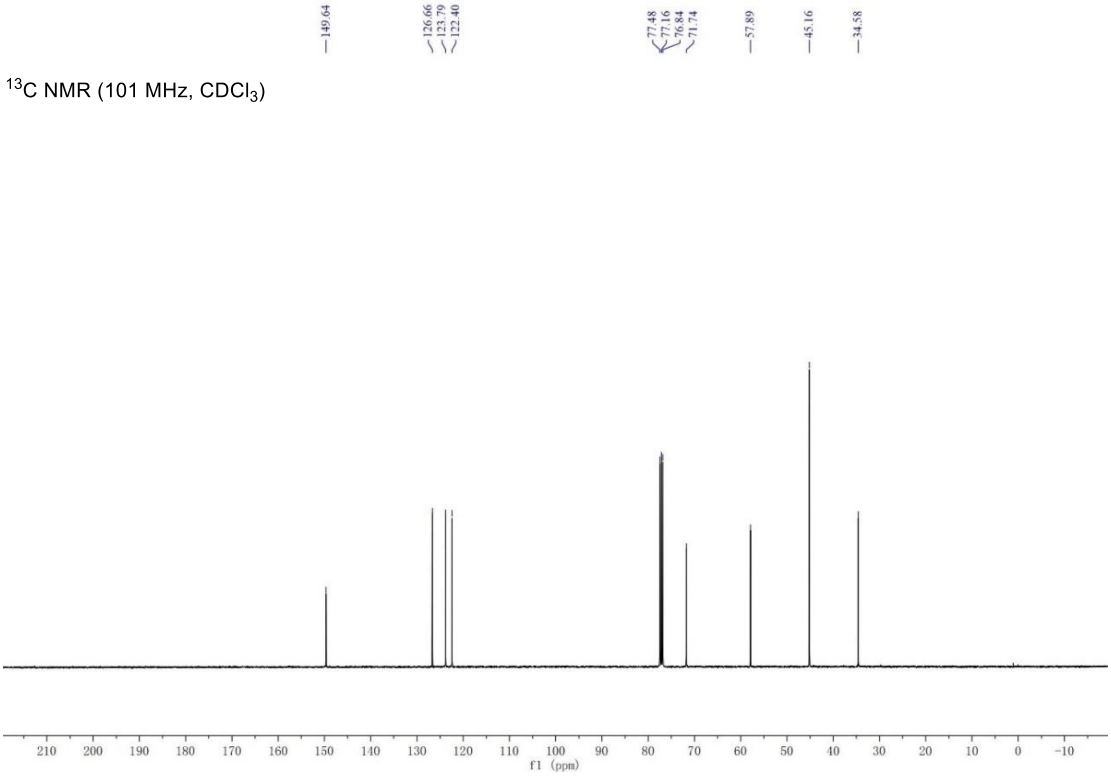

Supplementary Figure 116. ^{13}C NMR spectra of **4d**

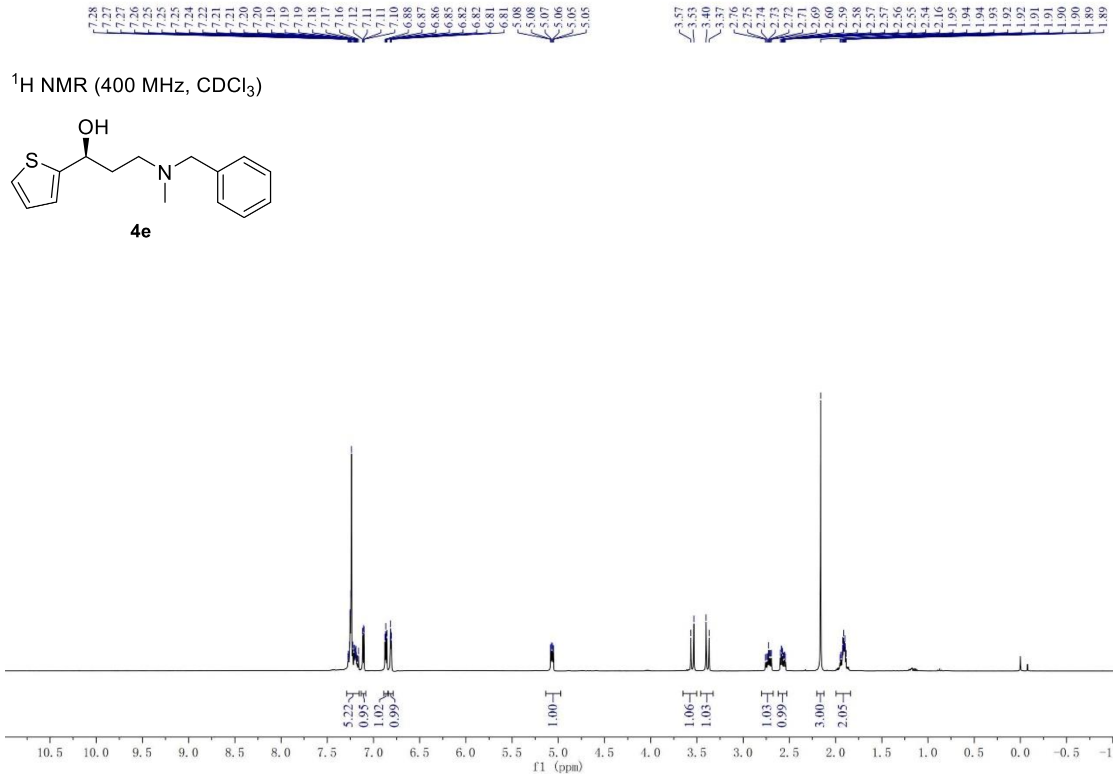

Supplementary Figure 117. ^{1}H NMR spectra of **4e**

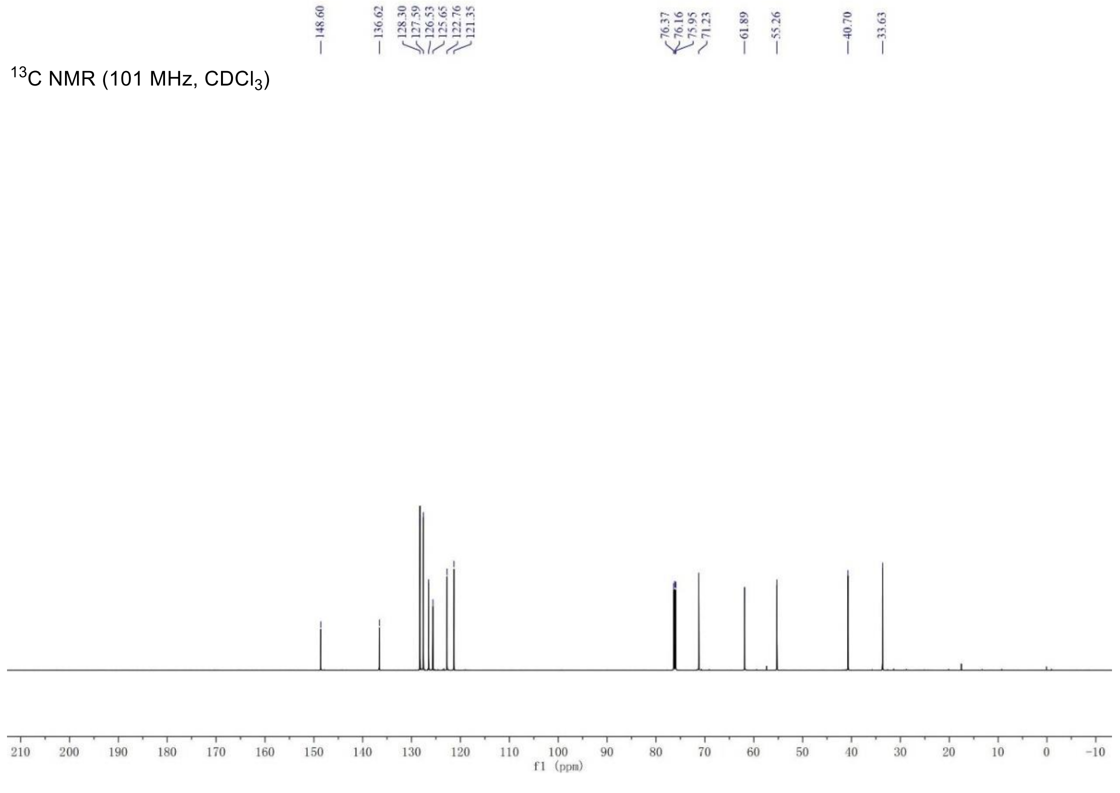

Supplementary Figure 118. ^{13}C NMR spectra of **4e**

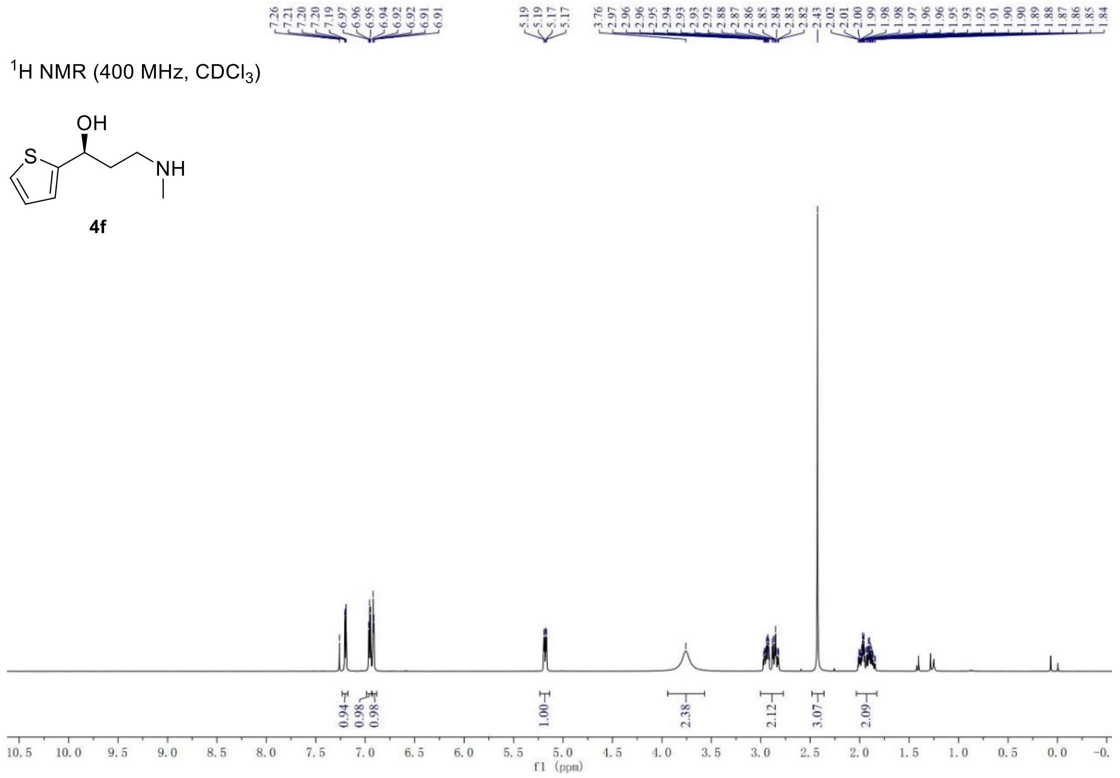

Supplementary Figure 119. ^{1}H NMR spectra of **4f**

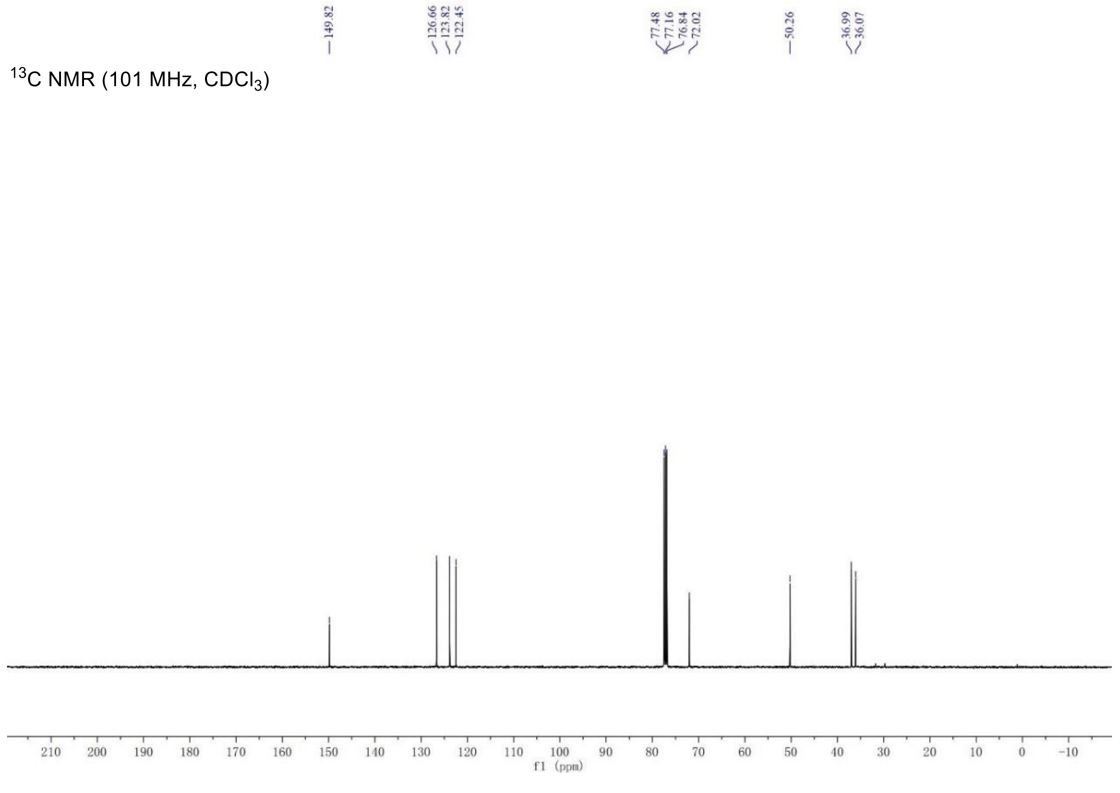

Supplementary Figure 120. ^{13}C NMR spectra of **4f**

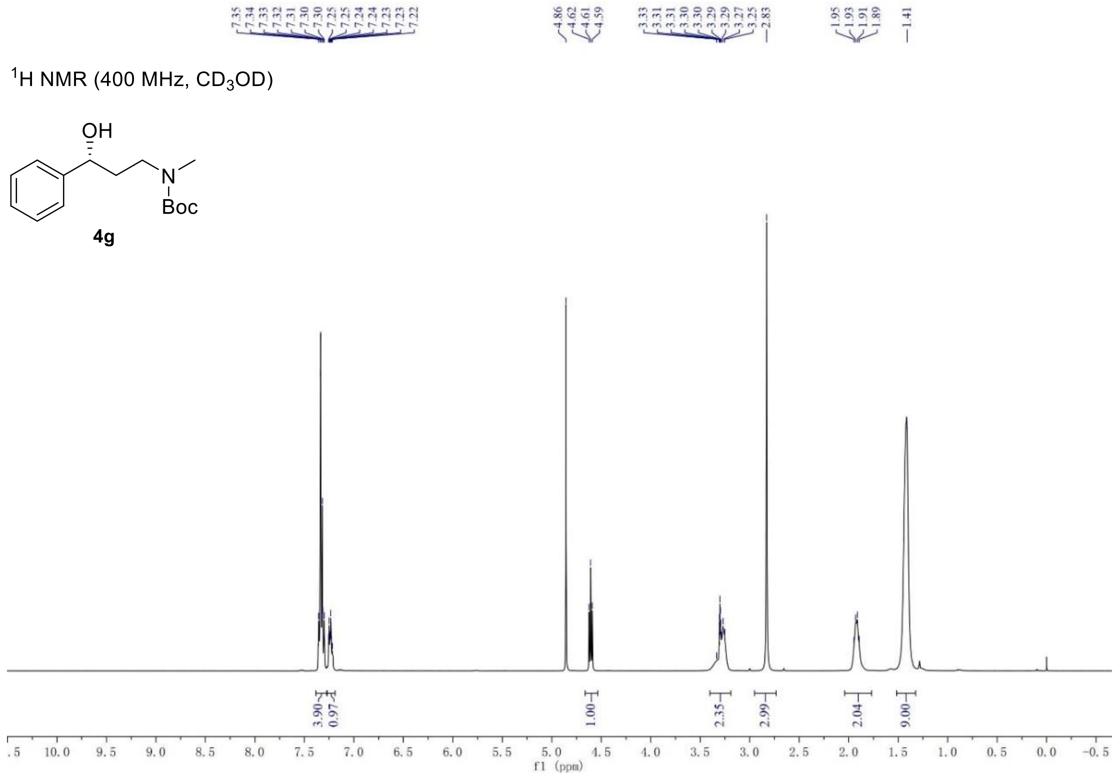

Supplementary Figure 121. ^{1}H NMR spectra of **4g**

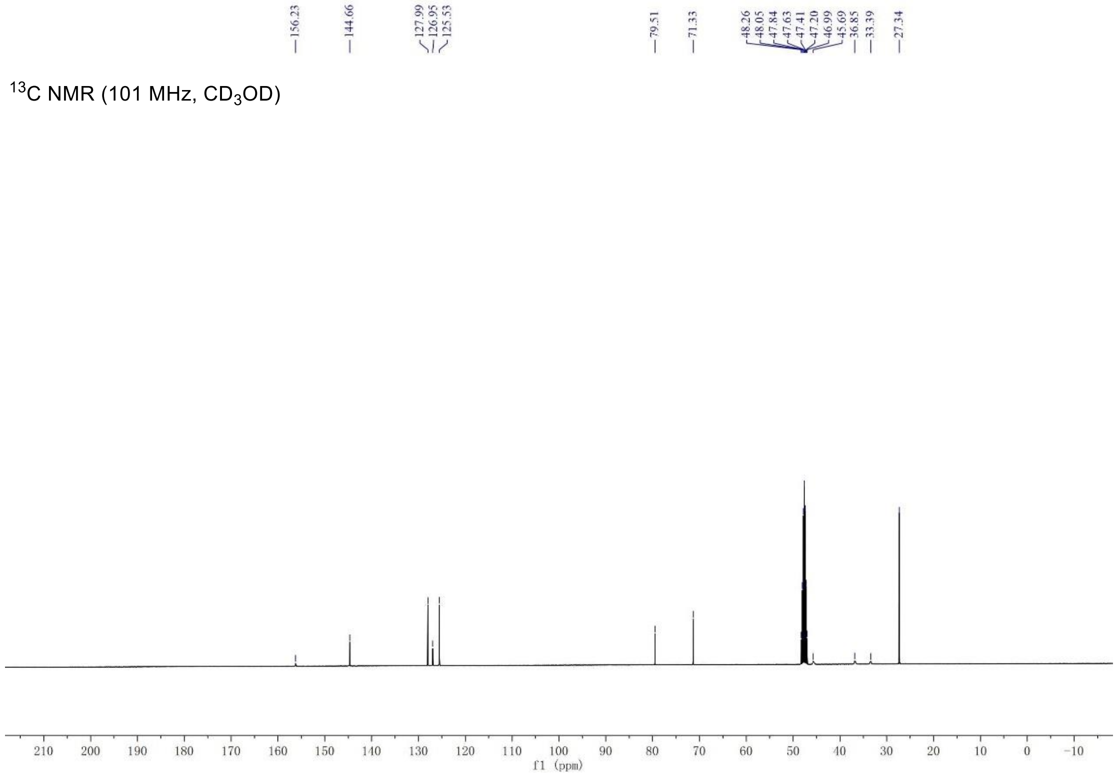

Supplementary Figure 122. ^{13}C NMR spectra of **4g**

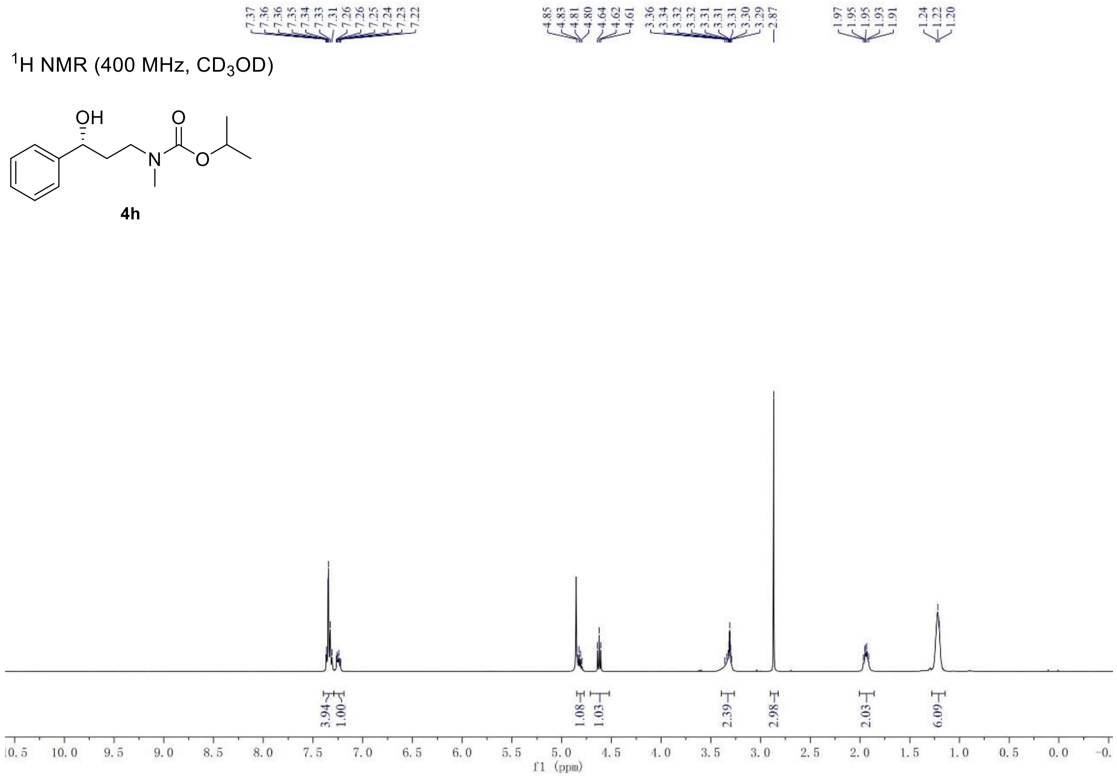

Supplementary Figure 123. ^{1}H NMR spectra of **4h**

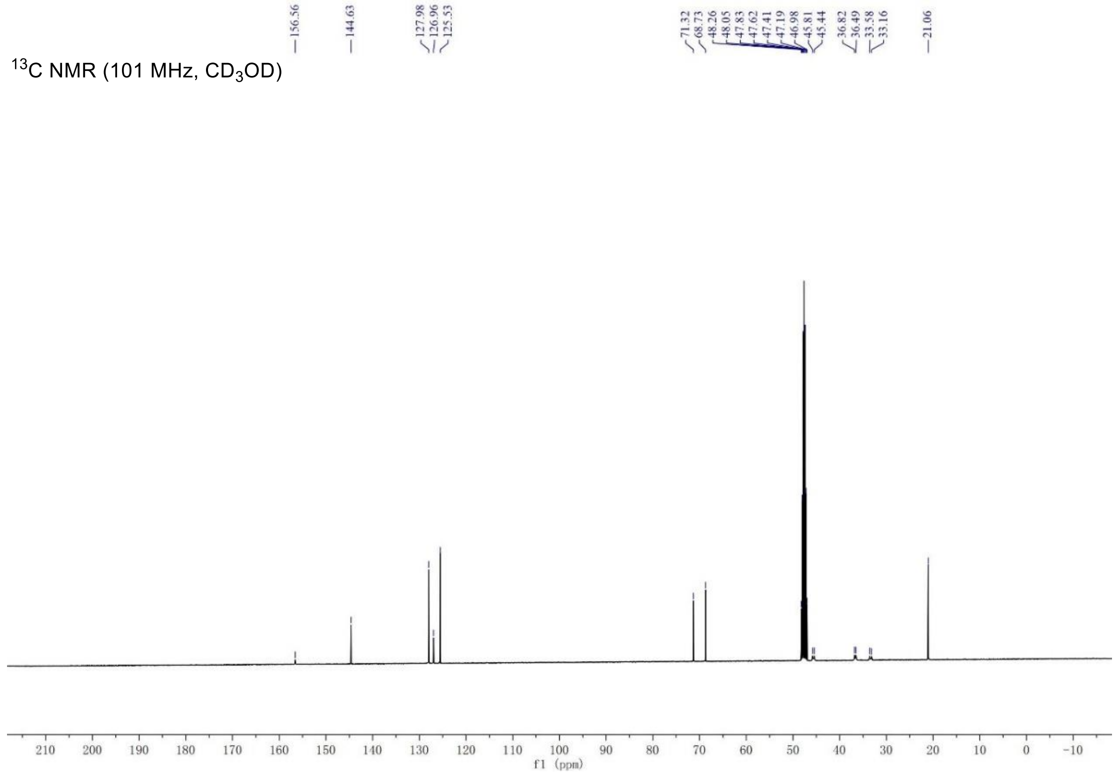

Supplementary Figure 124. ^{13}C NMR spectra of **4h**

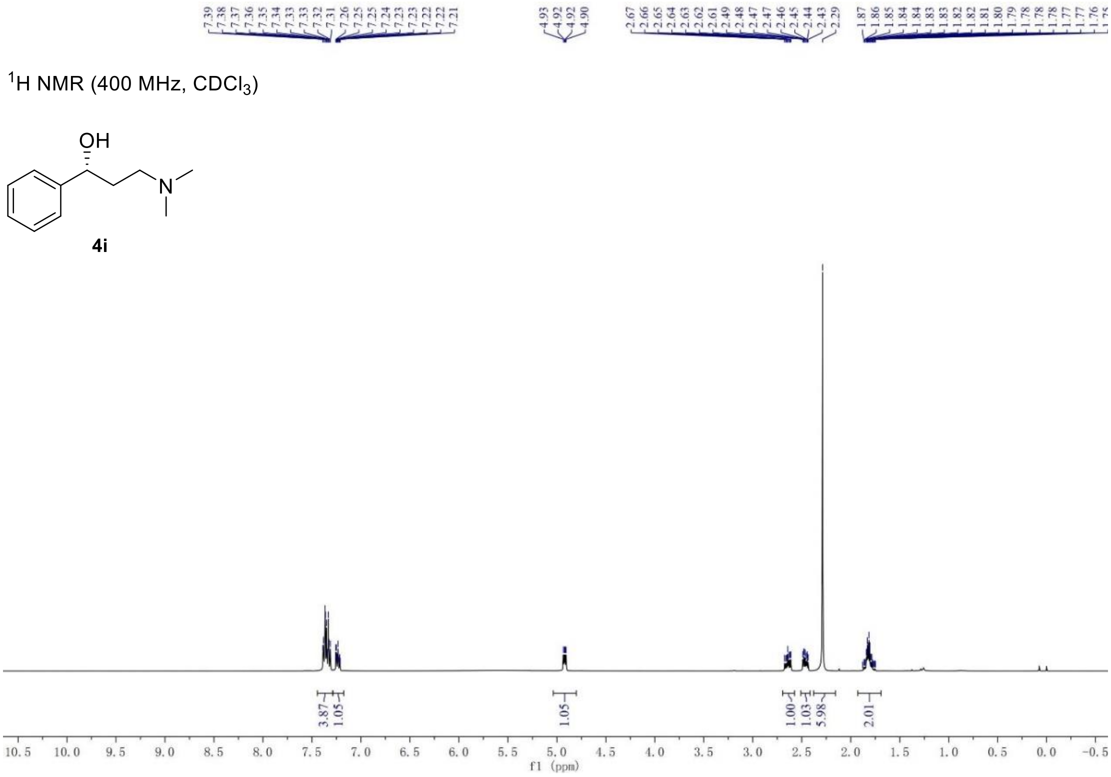

Supplementary Figure 125. ^{1}H NMR spectra of **4i**

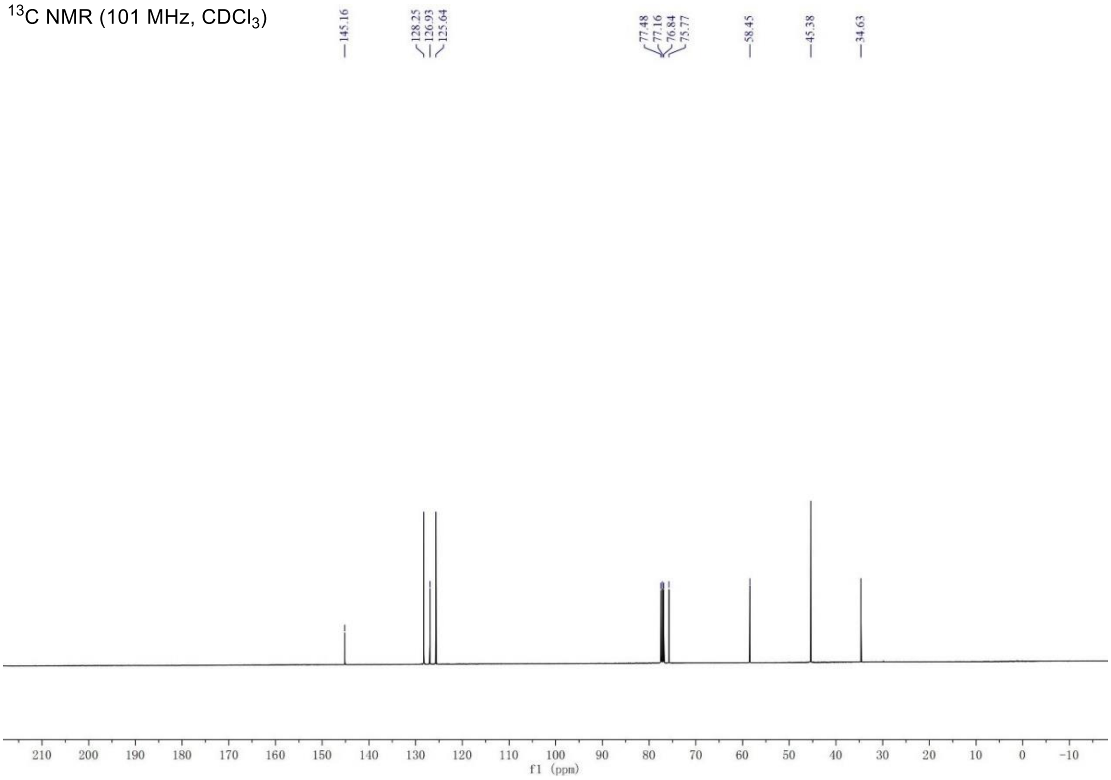

Supplementary Figure 126. ^{13}C NMR spectra of **4i**

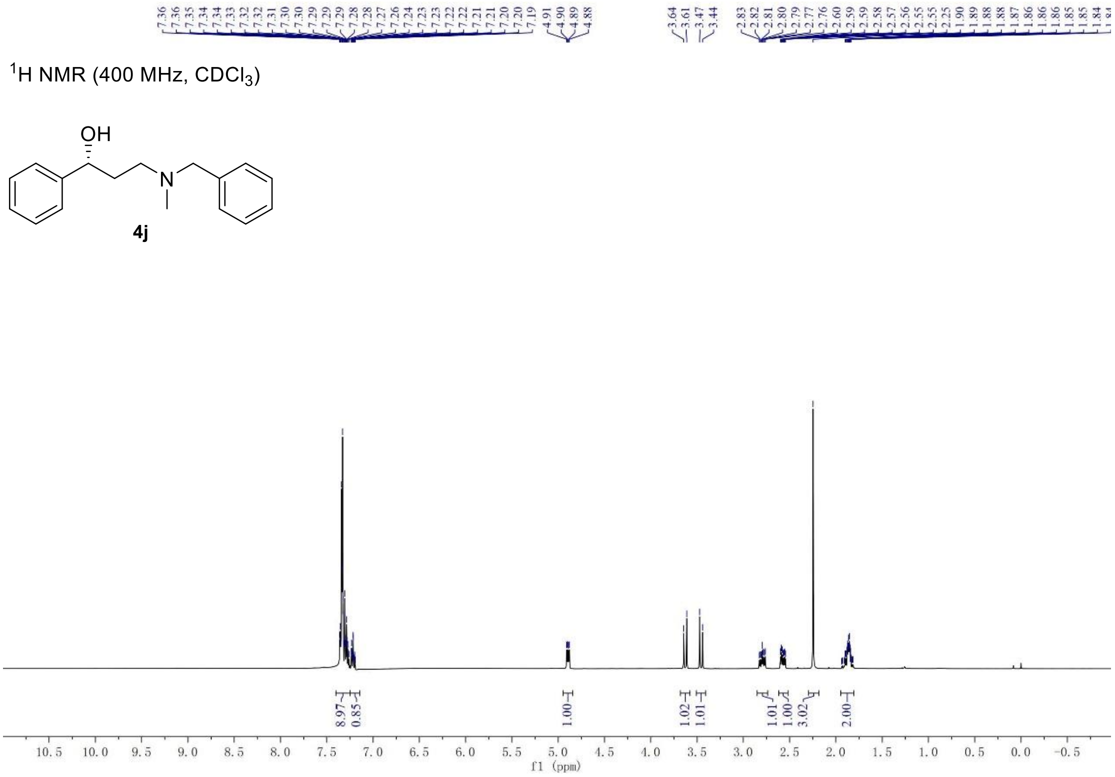

Supplementary Figure 127. ^{1}H NMR spectra of **4j**

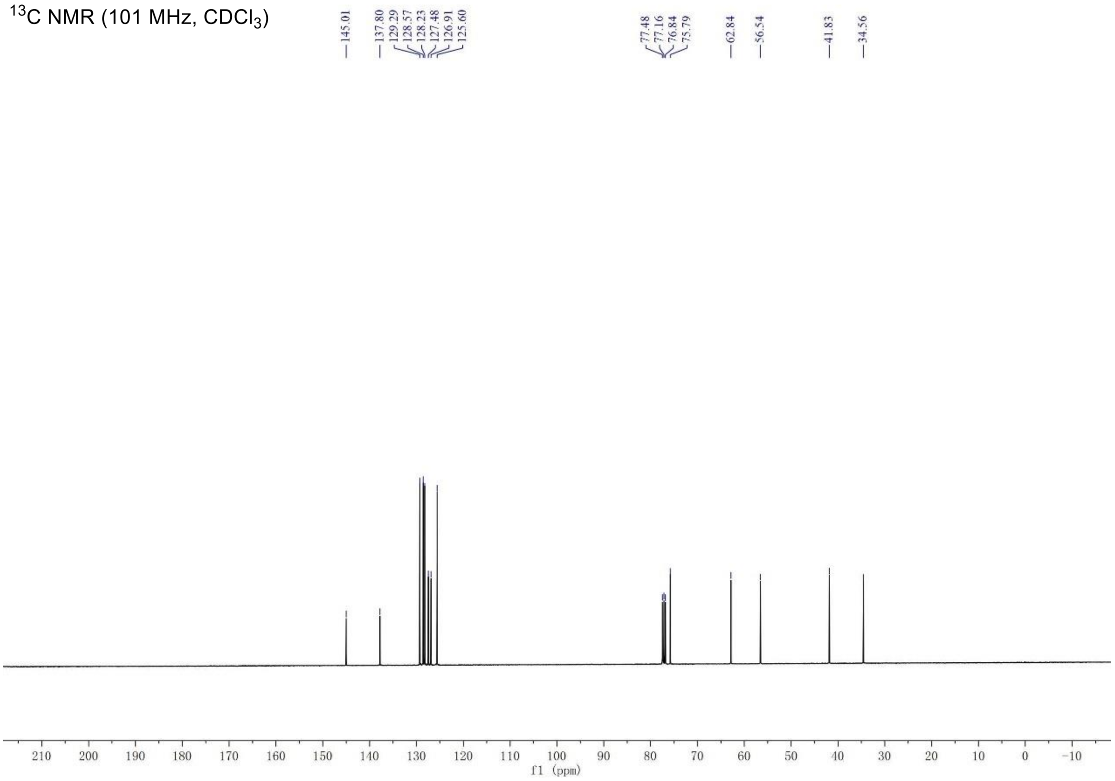

Supplementary Figure 128. ^{13}C NMR spectra of **4j**

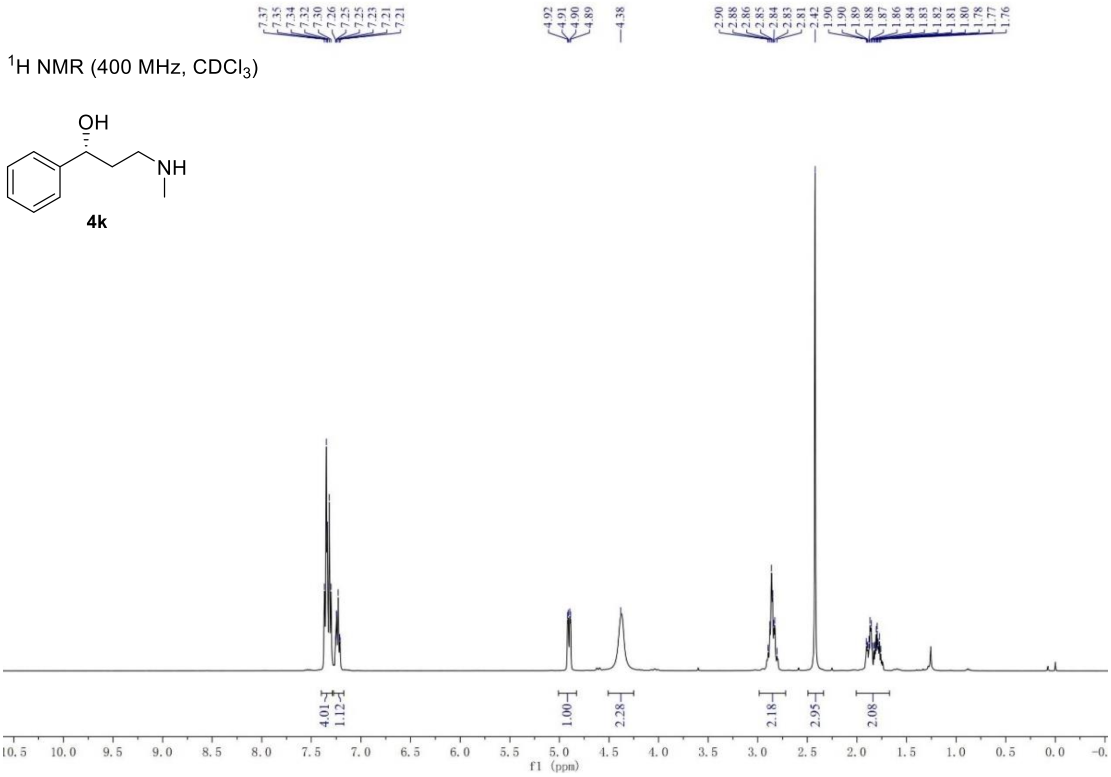

Supplementary Figure 129. ^{1}H NMR spectra of **4k**

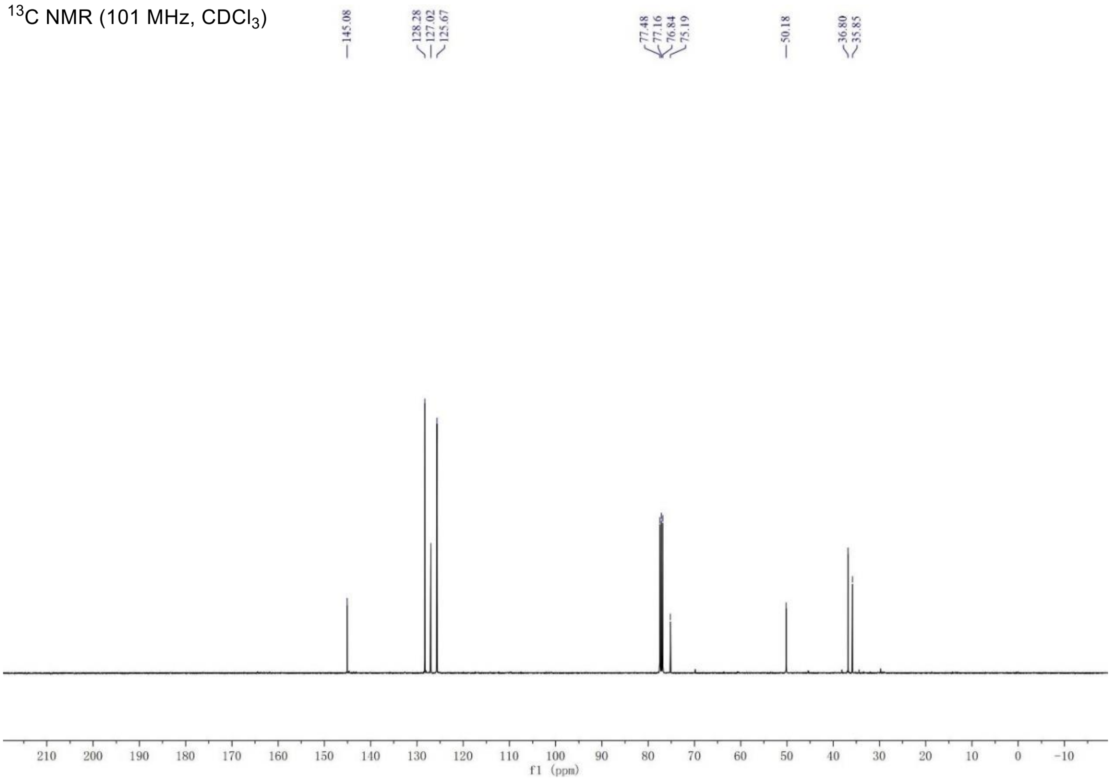

Supplementary Figure 130. ^{13}C NMR spectra of **4k**

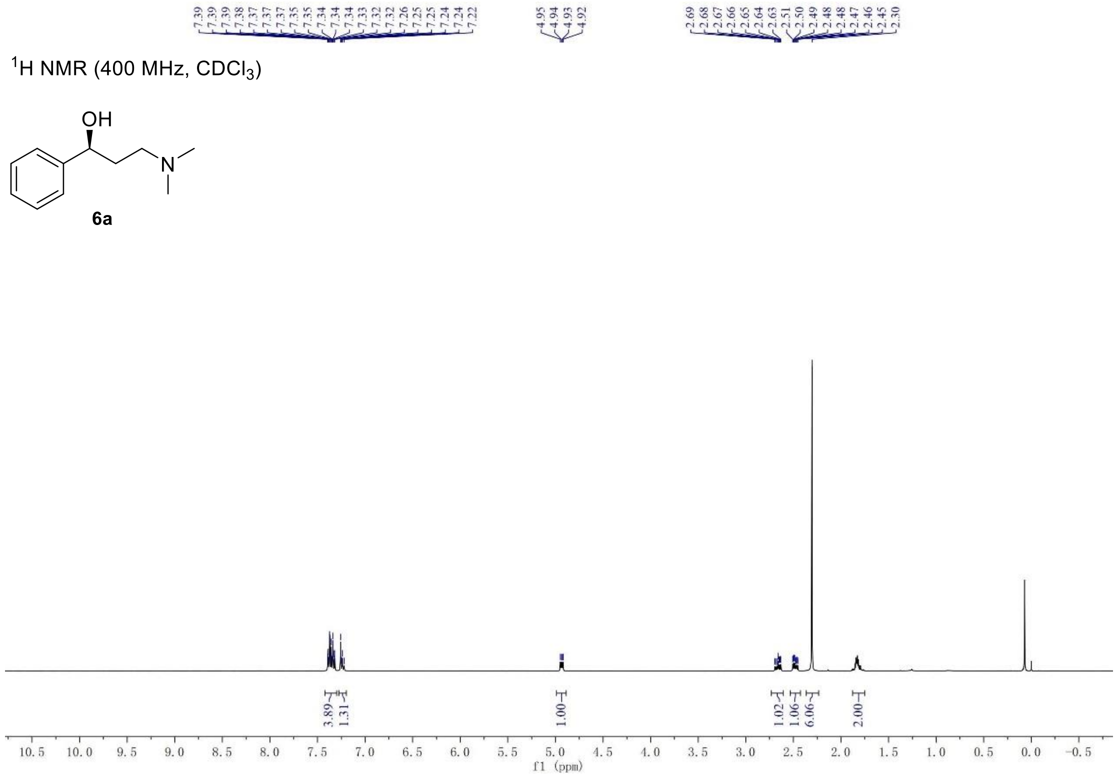

Supplementary Figure 131. ^{1}H NMR spectra of **6a**

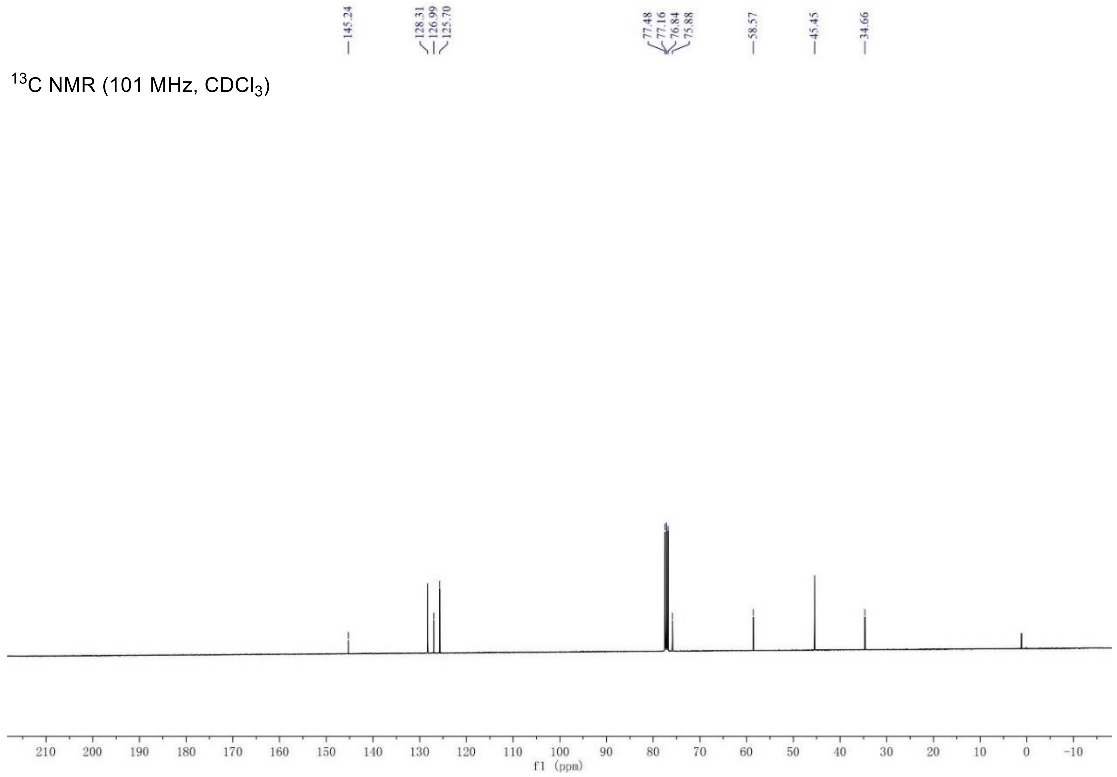

Supplementary Figure 132. ^{13}C NMR spectra of **6a**

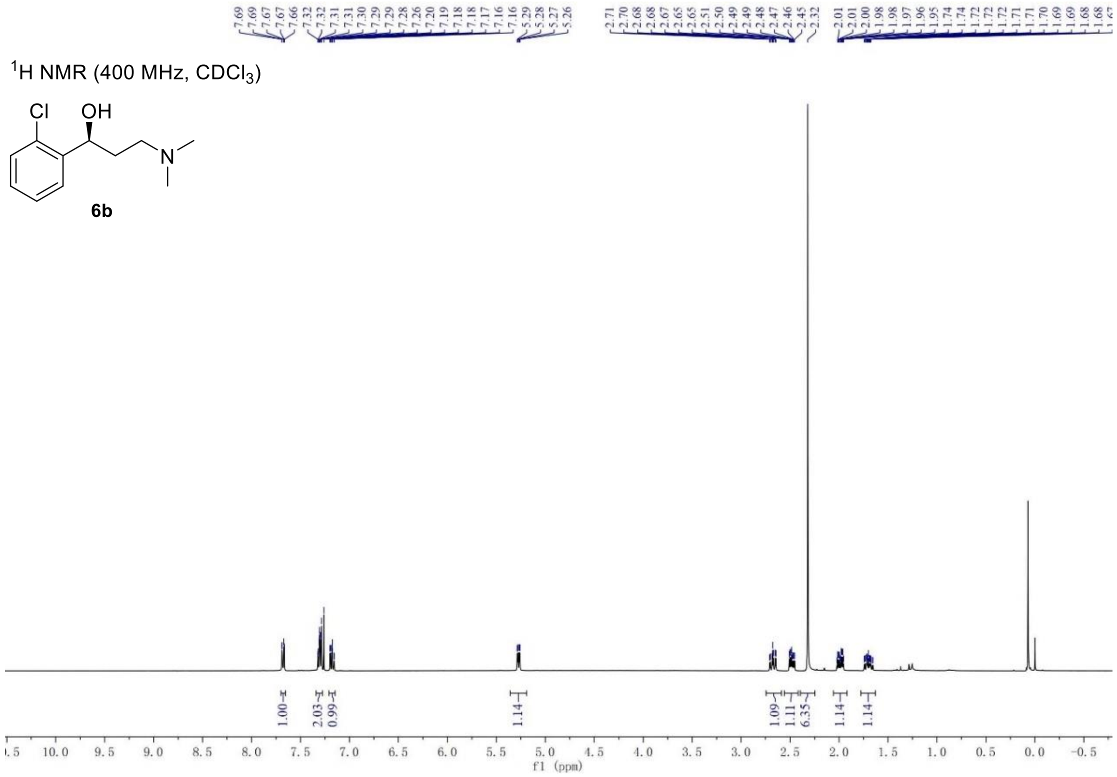

Supplementary Figure 133. ^{1}H NMR spectra of **6b**

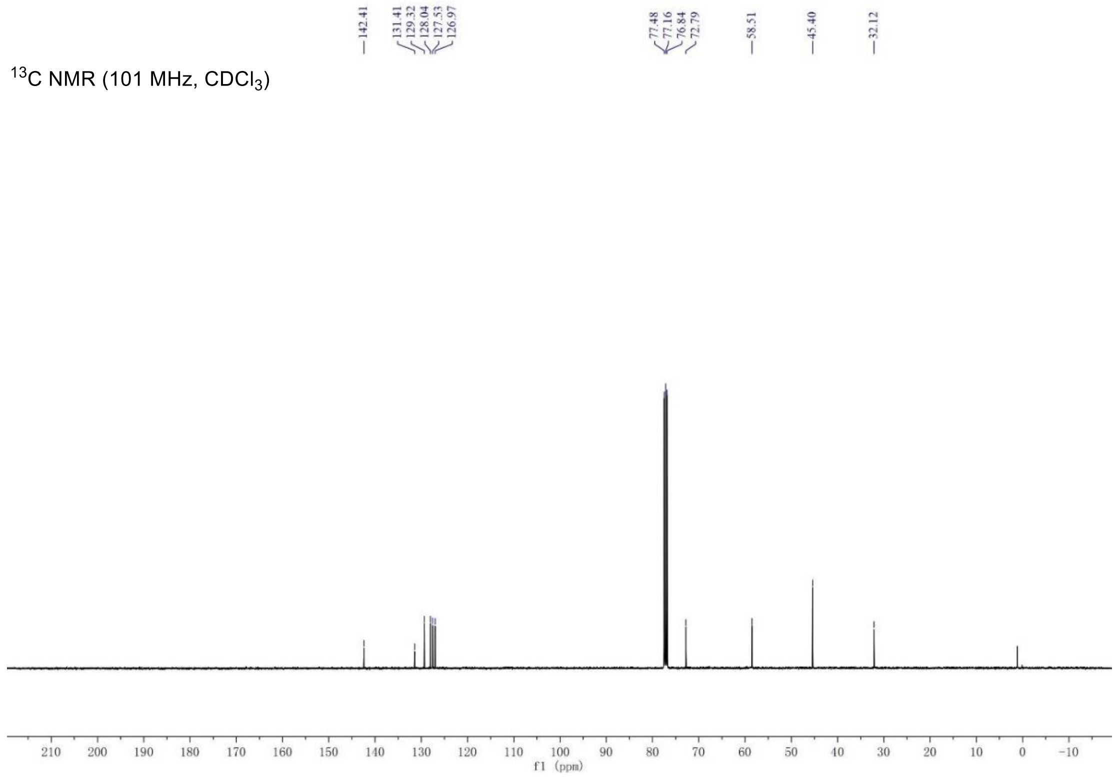

Supplementary Figure 134. ^{13}C NMR spectra of **6b**

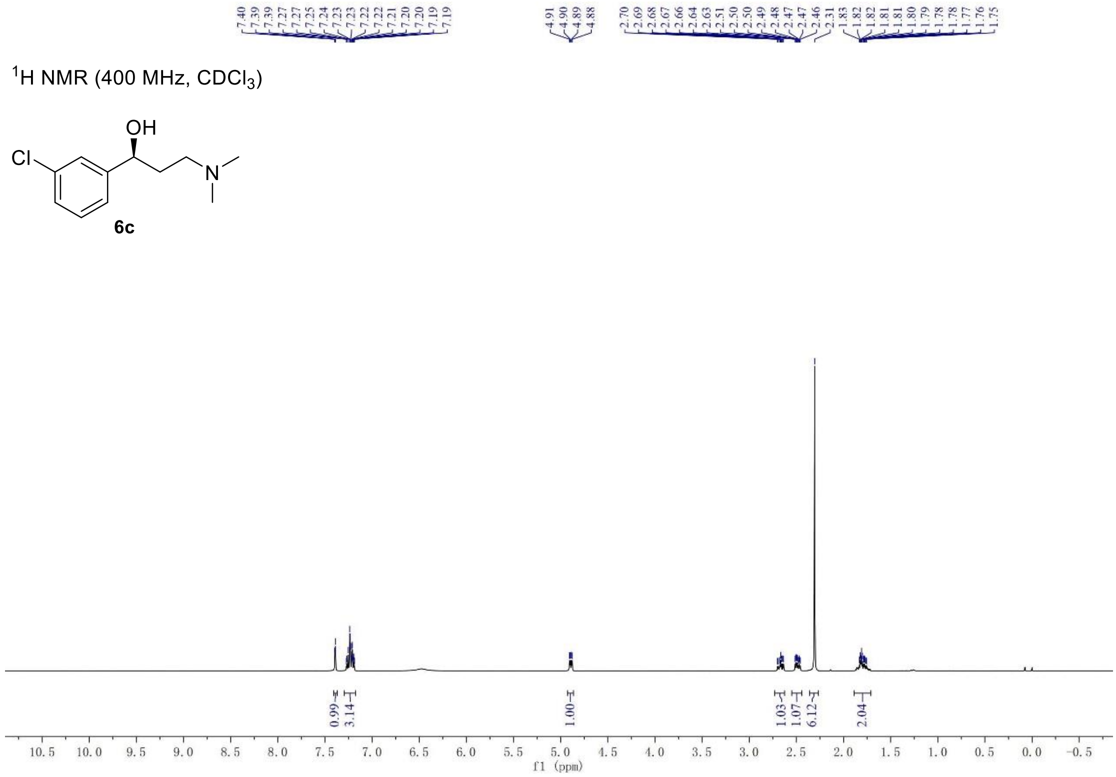

Supplementary Figure 135. ^{1}H NMR spectra of **6c**

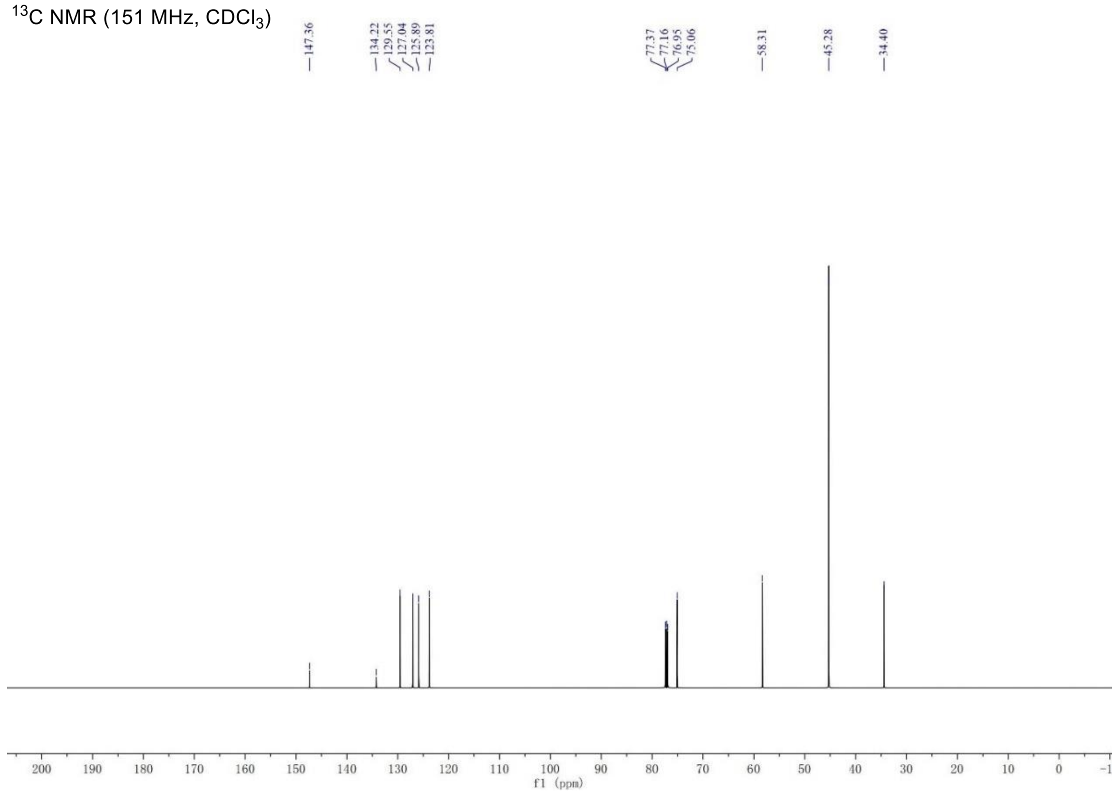

Supplementary Figure 136. ^{13}C NMR spectra of **6c**

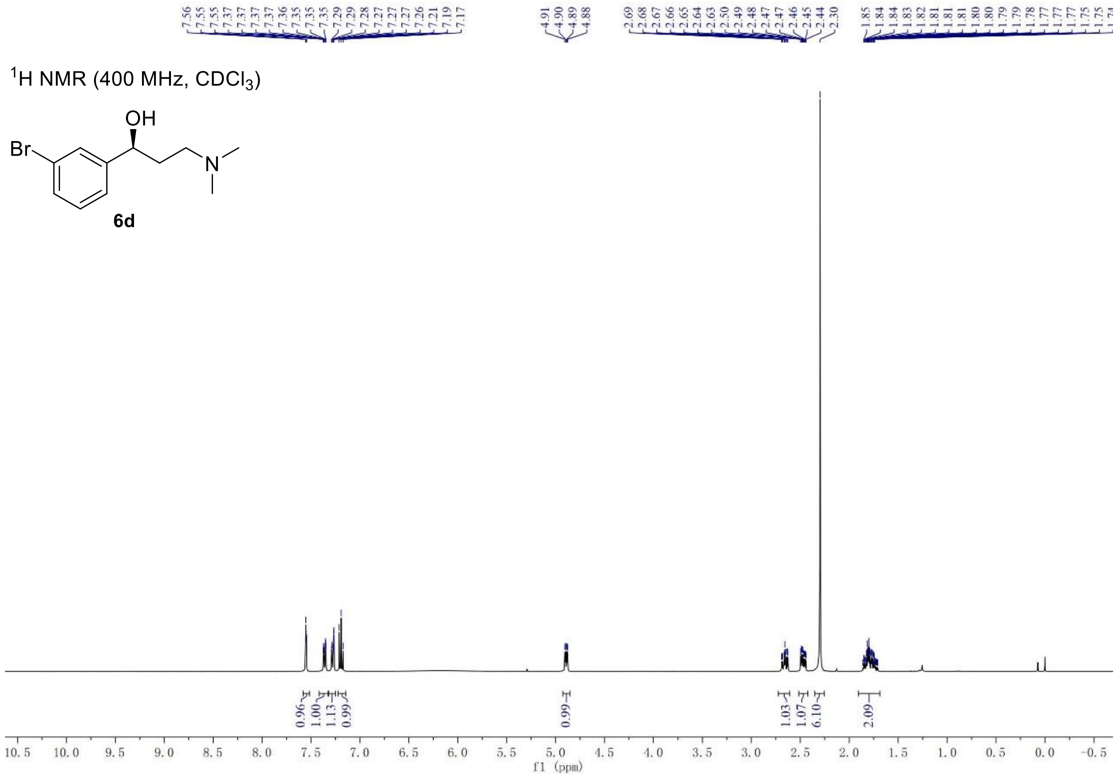

Supplementary Figure 137. ^{1}H NMR spectra of **6d**

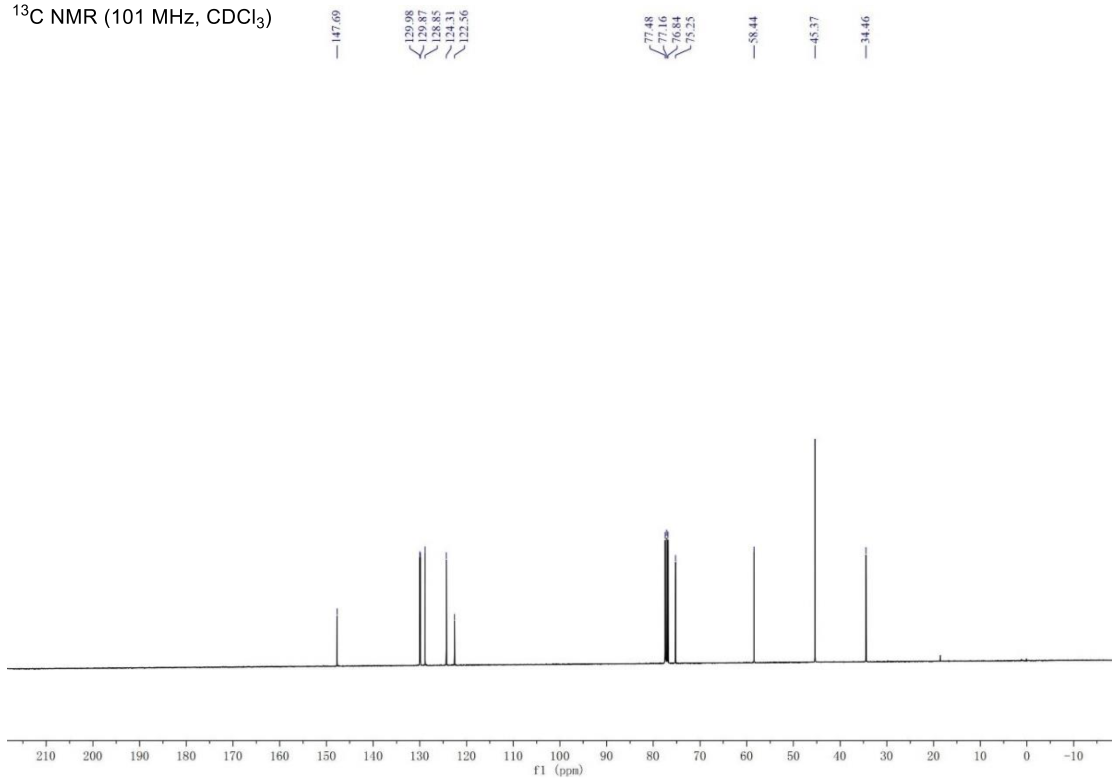

Supplementary Figure 138. ^{13}C NMR spectra of **6d**

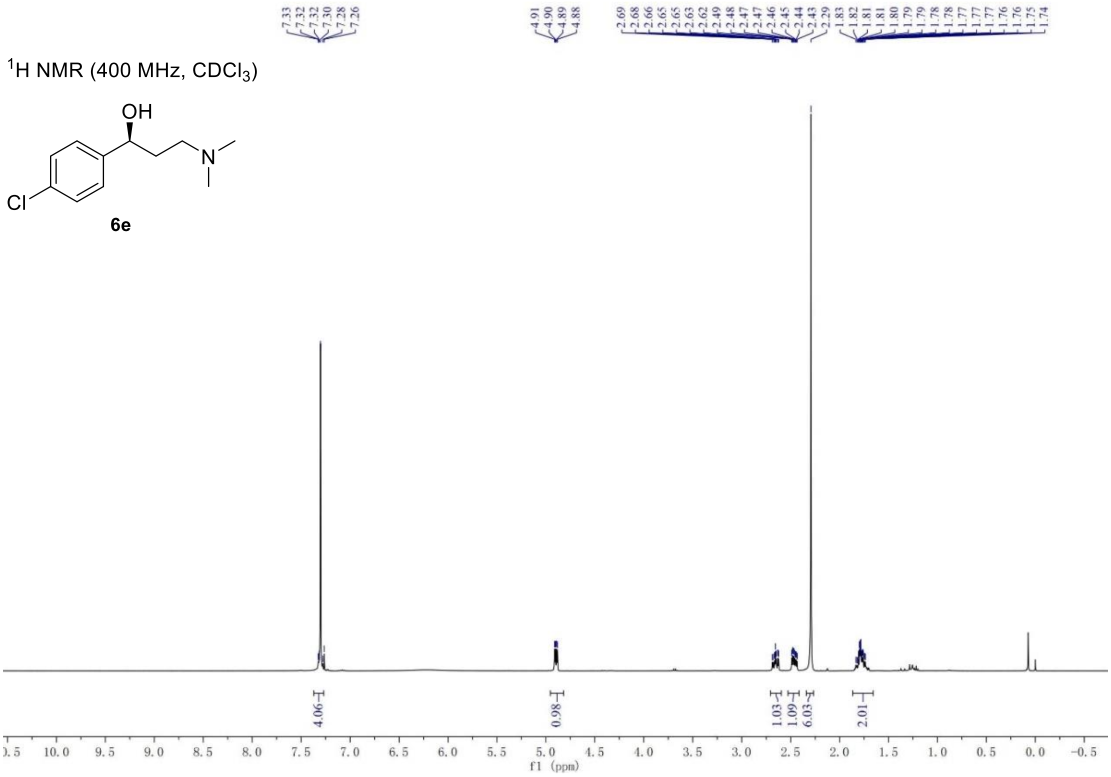

Supplementary Figure 139. ^{1}H NMR spectra of **6e**

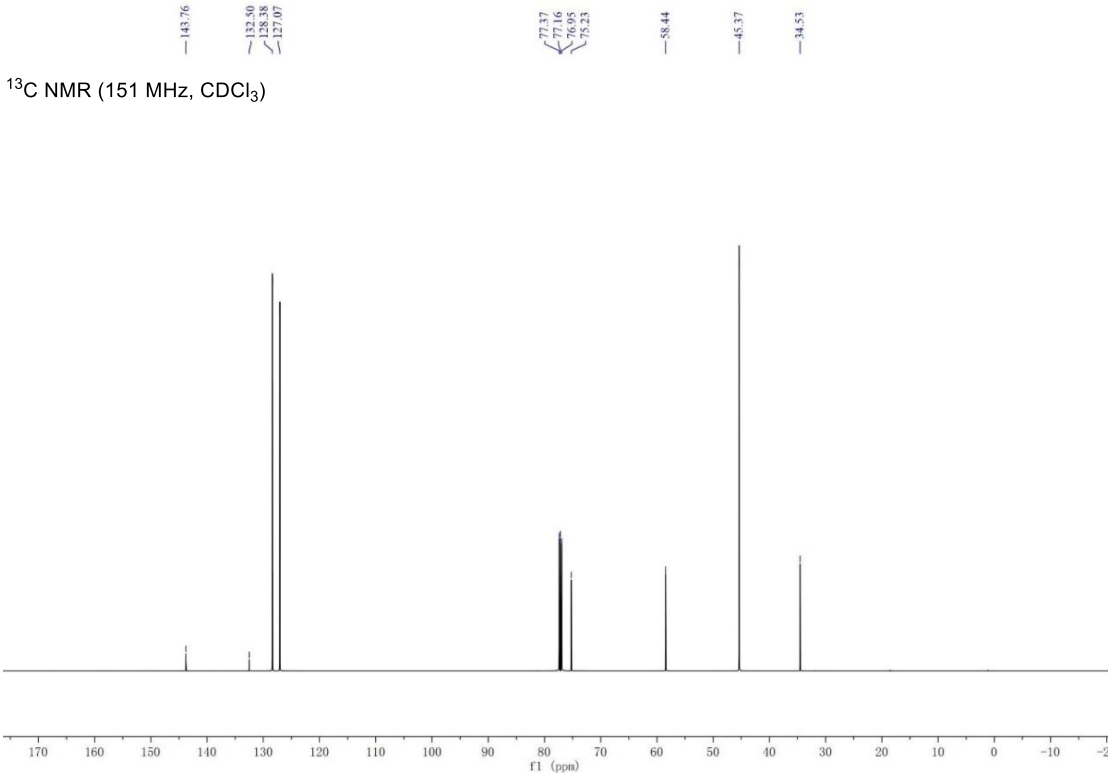

Supplementary Figure 140. ^{13}C NMR spectra of **6e**

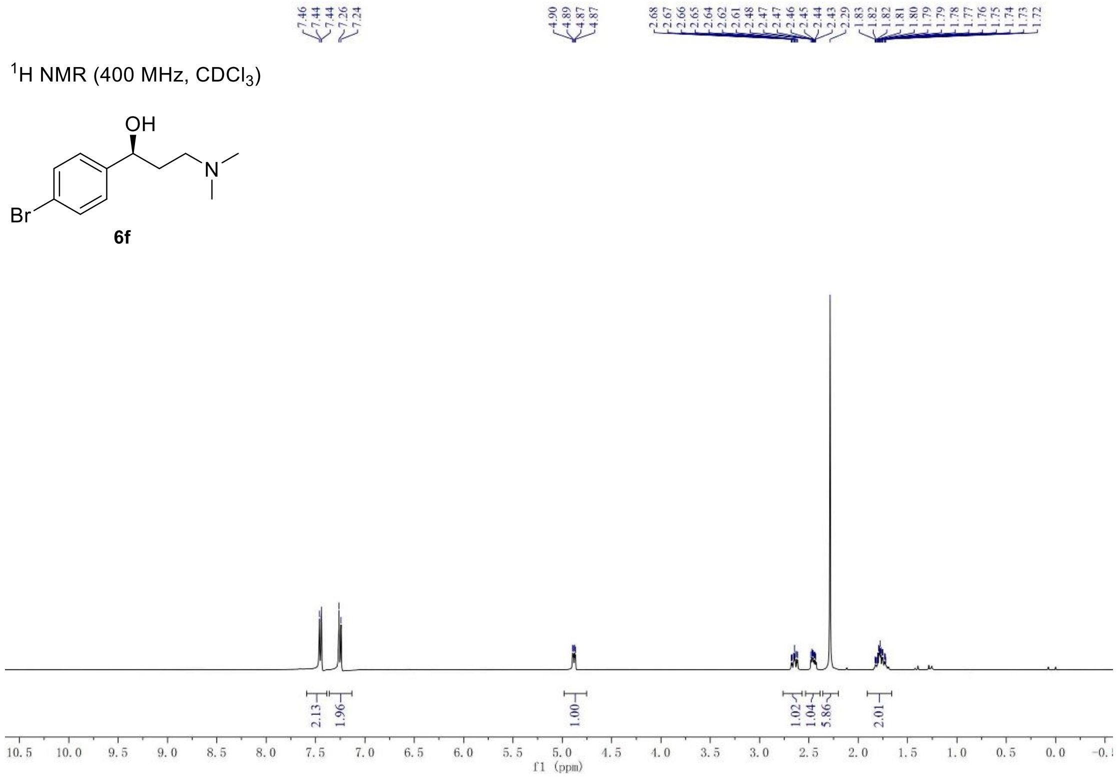

Supplementary Figure 141. ^{1}H NMR spectra of **6f**

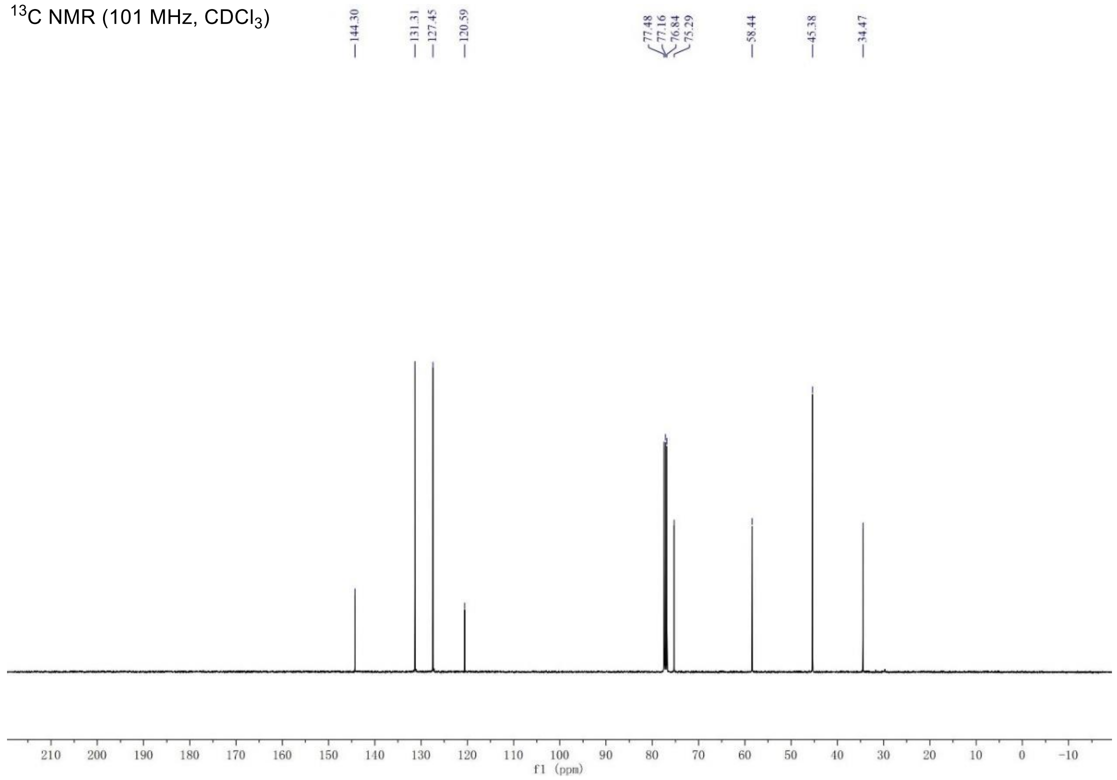

Supplementary Figure 142. ^{13}C NMR spectra of **6f**

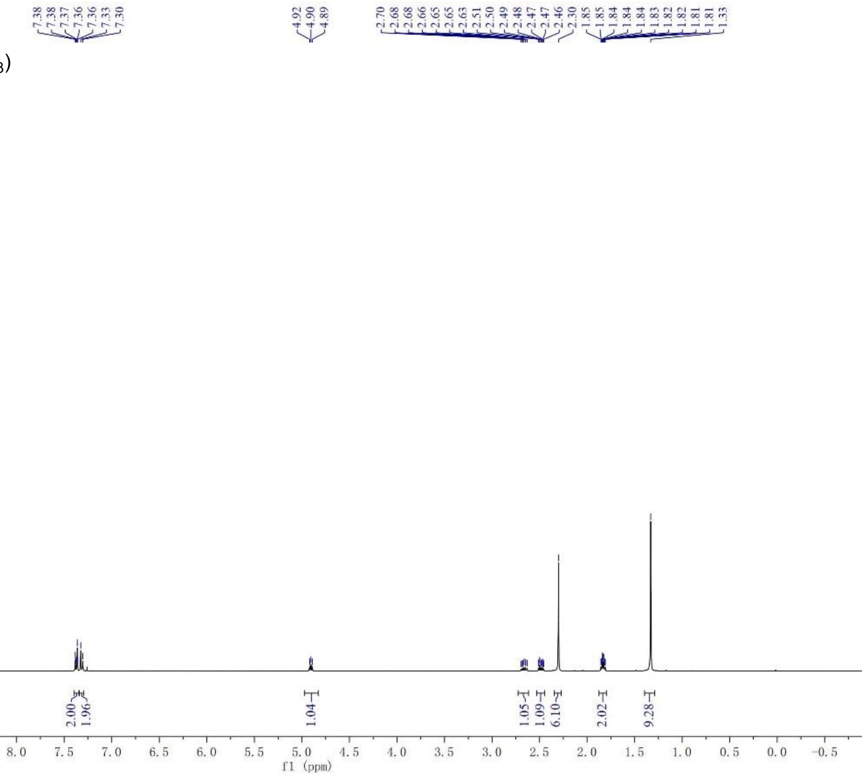

^{1}H NMR (400 MHz, $CDCl_3$)

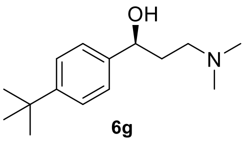

6g

Supplementary Figure 143. ^{1}H NMR spectra of **6g**

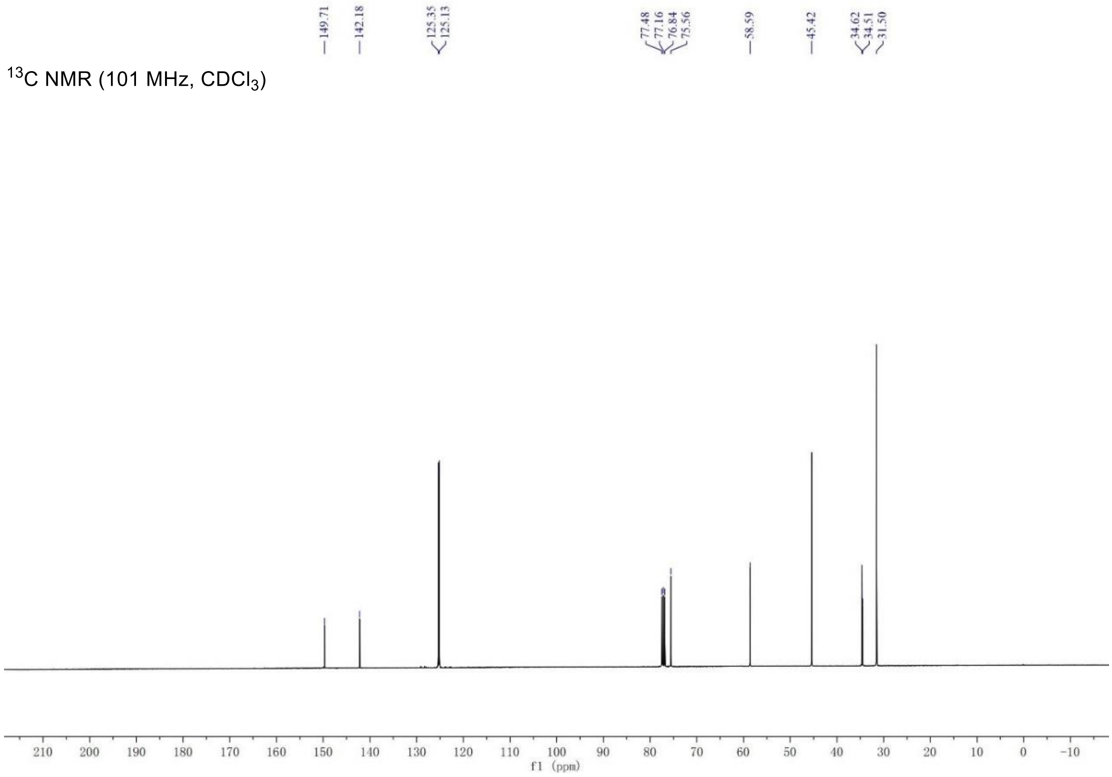

^{13}C NMR (101 MHz, $CDCl_3$)

Supplementary Figure 144. ^{13}C NMR spectra of **6g**

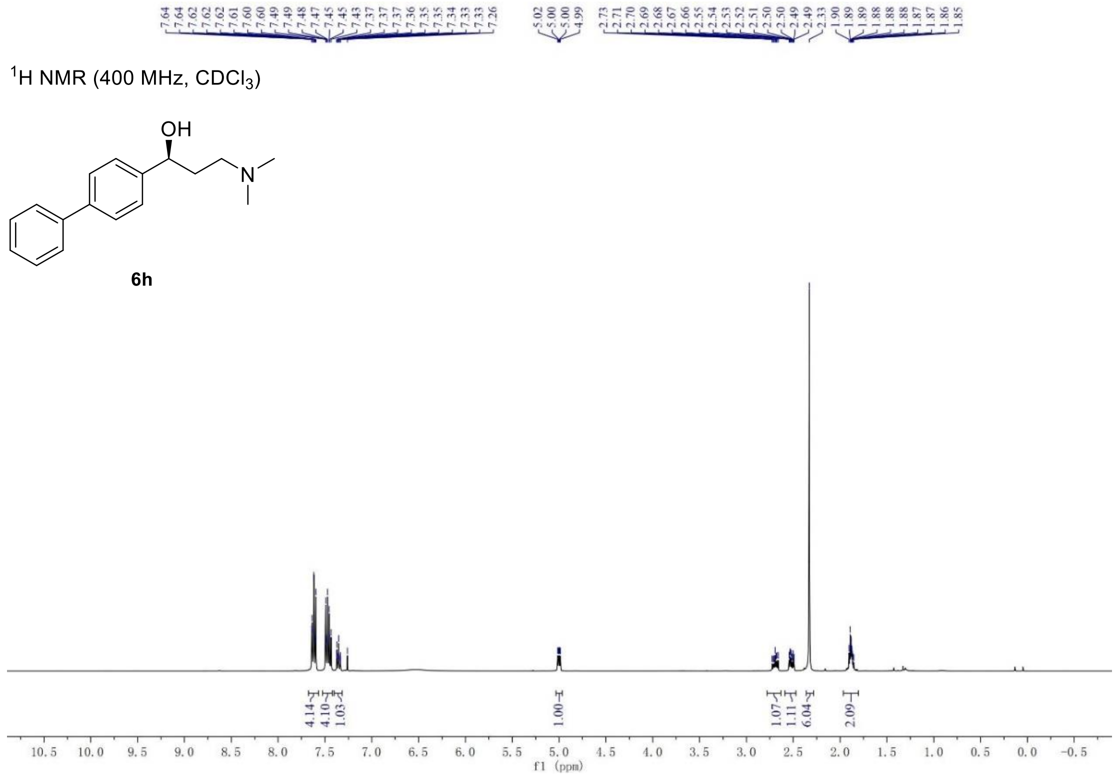

Supplementary Figure 145. ^{1}H NMR spectra of **6h**

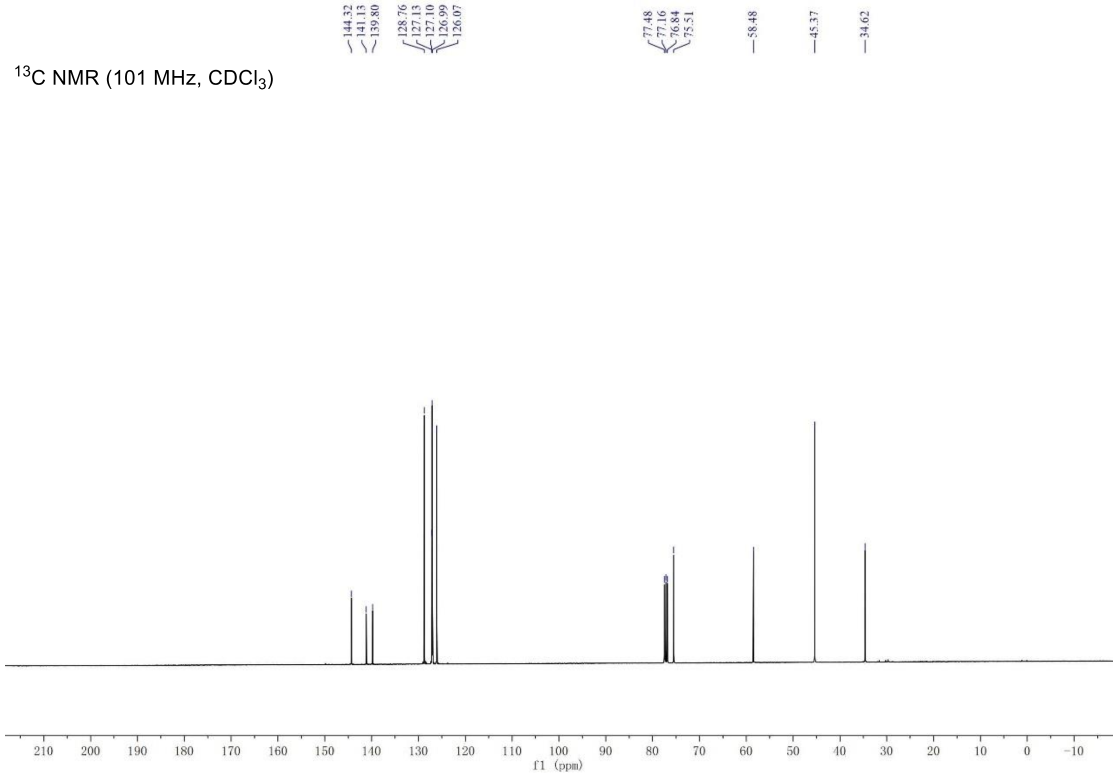

Supplementary Figure 146. ^{13}C NMR spectra of **6h**

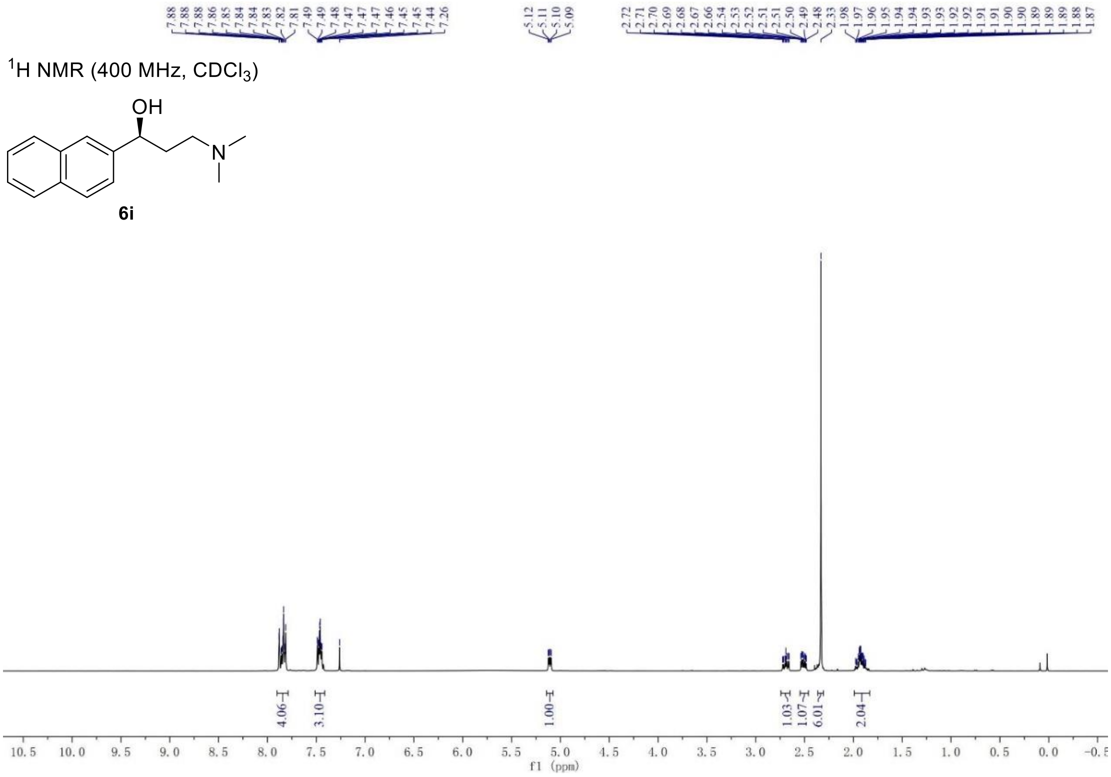

Supplementary Figure 147. ^{1}H NMR spectra of **6i**

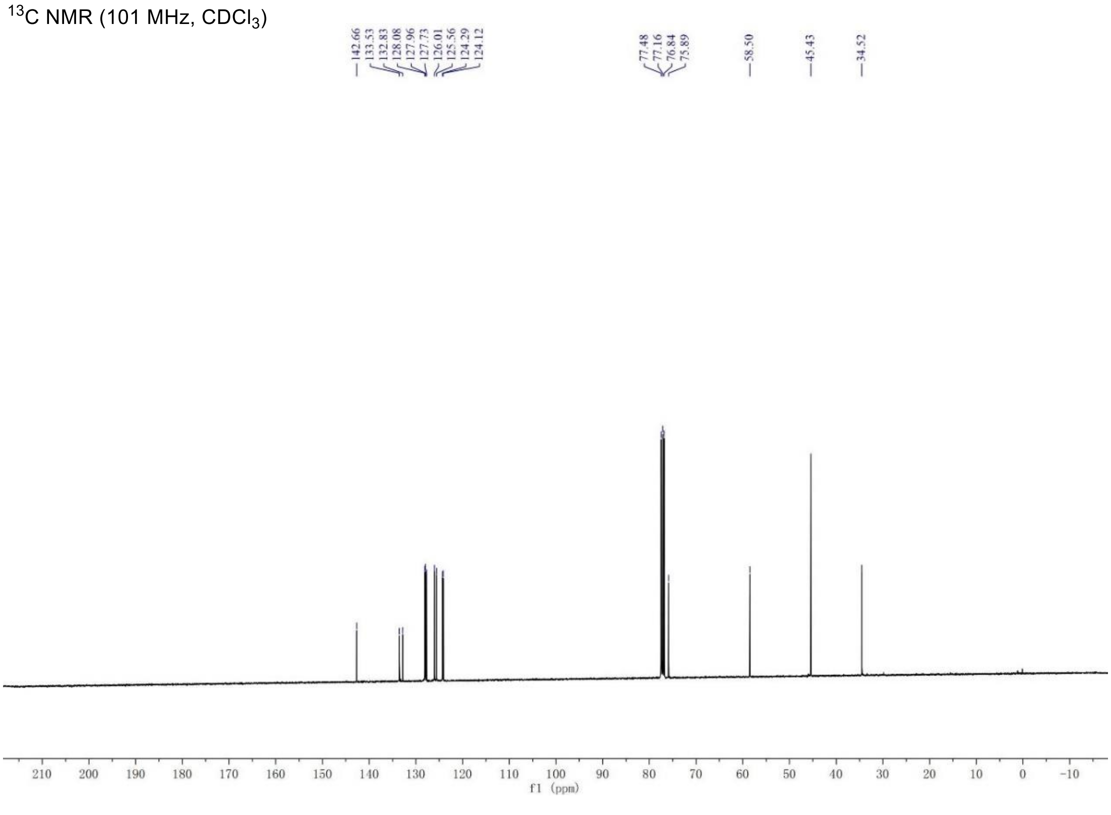

Supplementary Figure 148. ^{13}C NMR spectra of **6i**

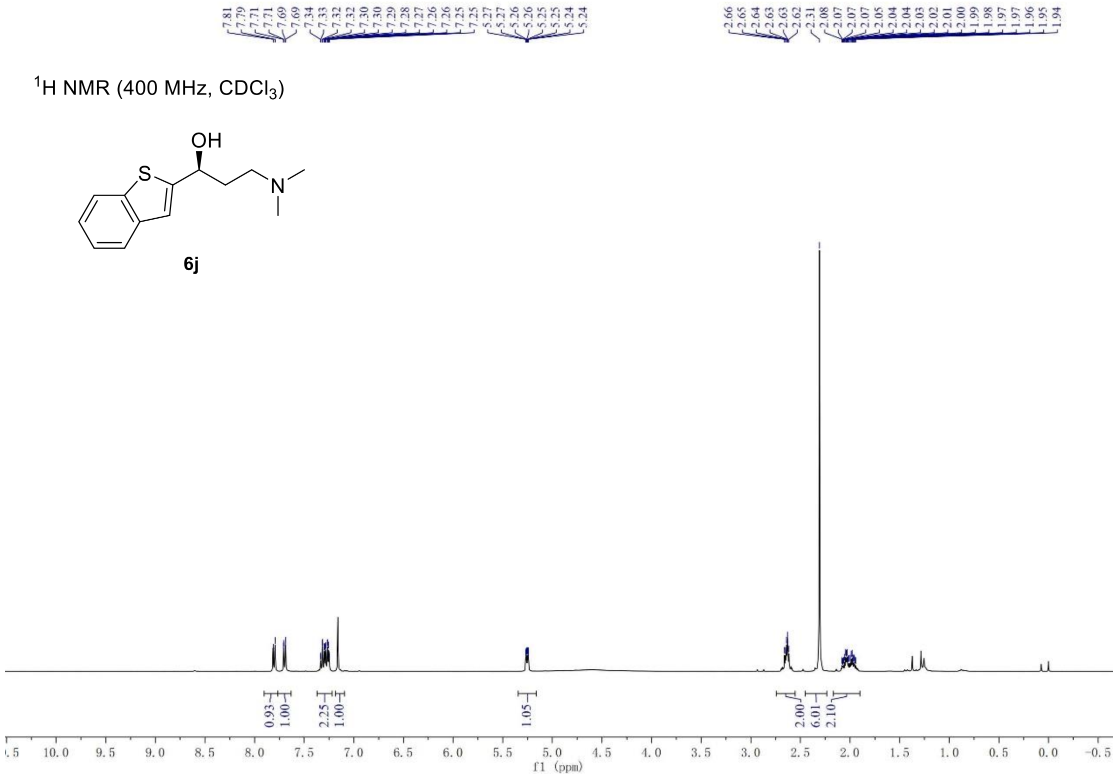

Supplementary Figure 149. ^{1}H NMR spectra of **6j**

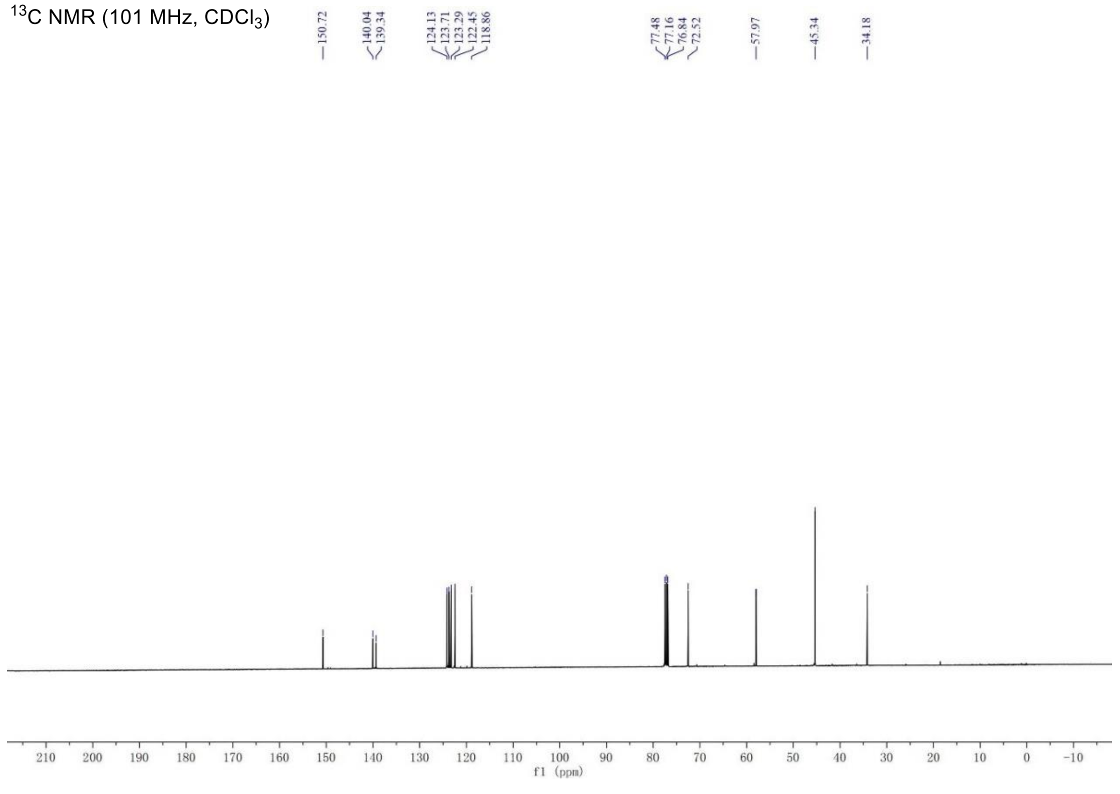

Supplementary Figure 150. ^{13}C NMR spectra of **6j**

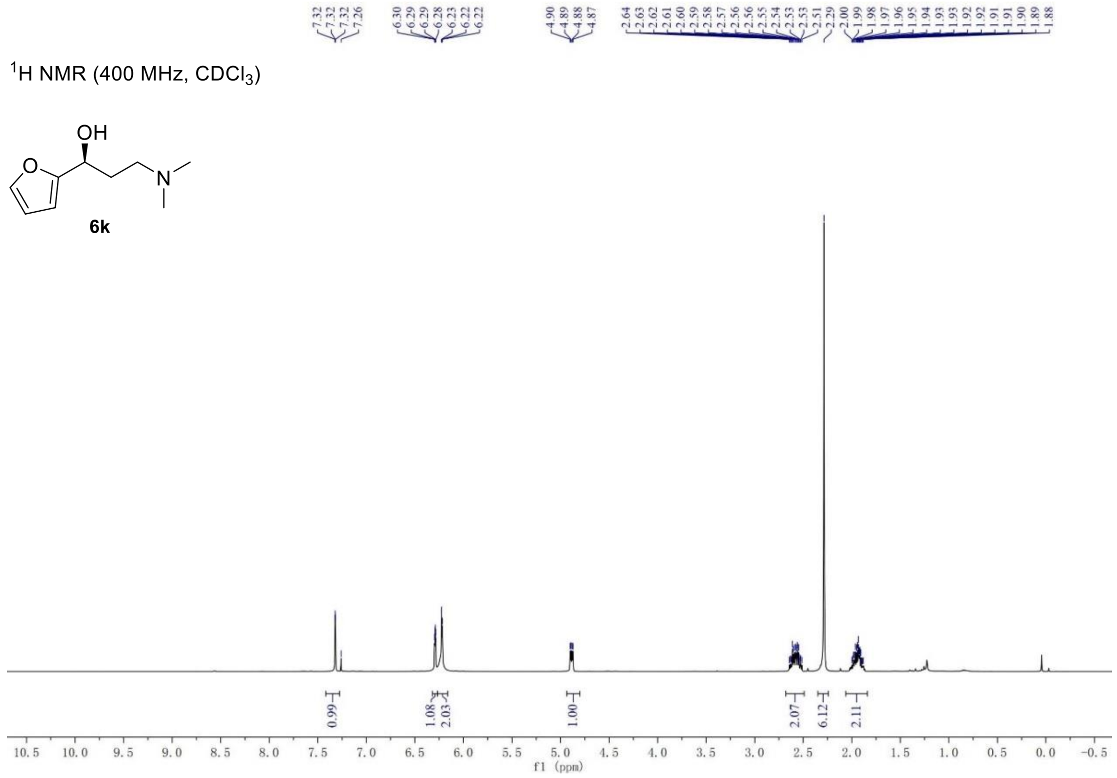

Supplementary Figure 151. ^{1}H NMR spectra of **6k**

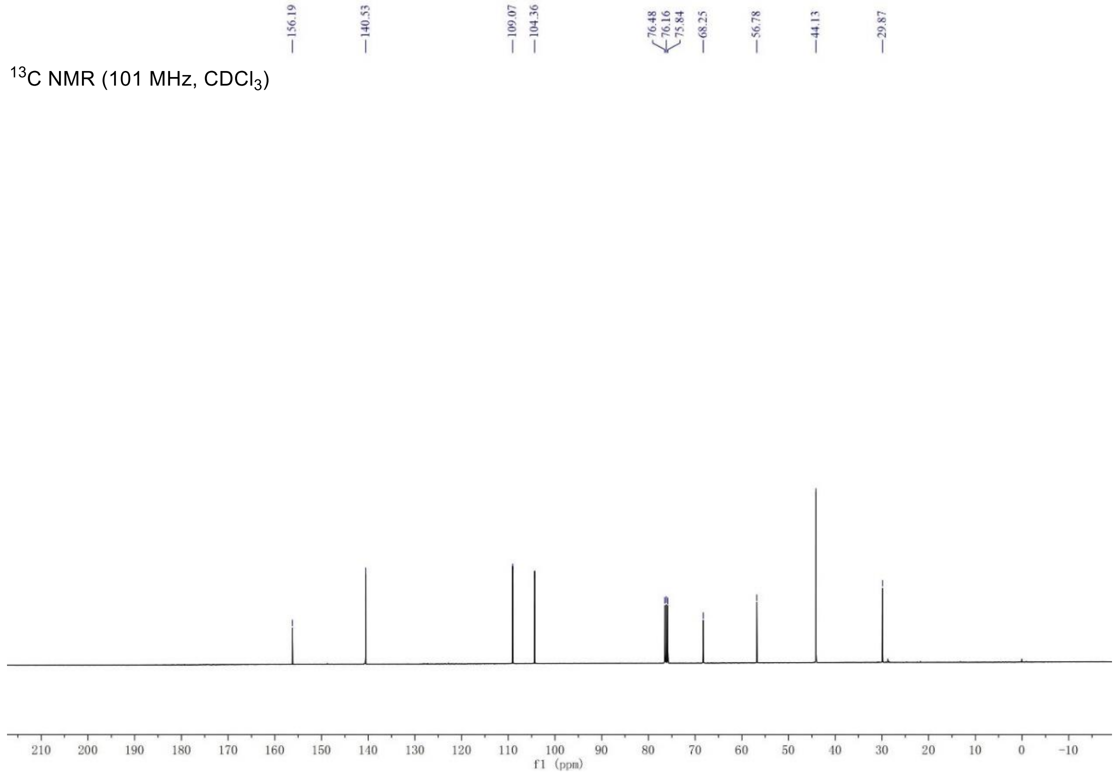

Supplementary Figure 152. ^{13}C NMR spectra of **6k**

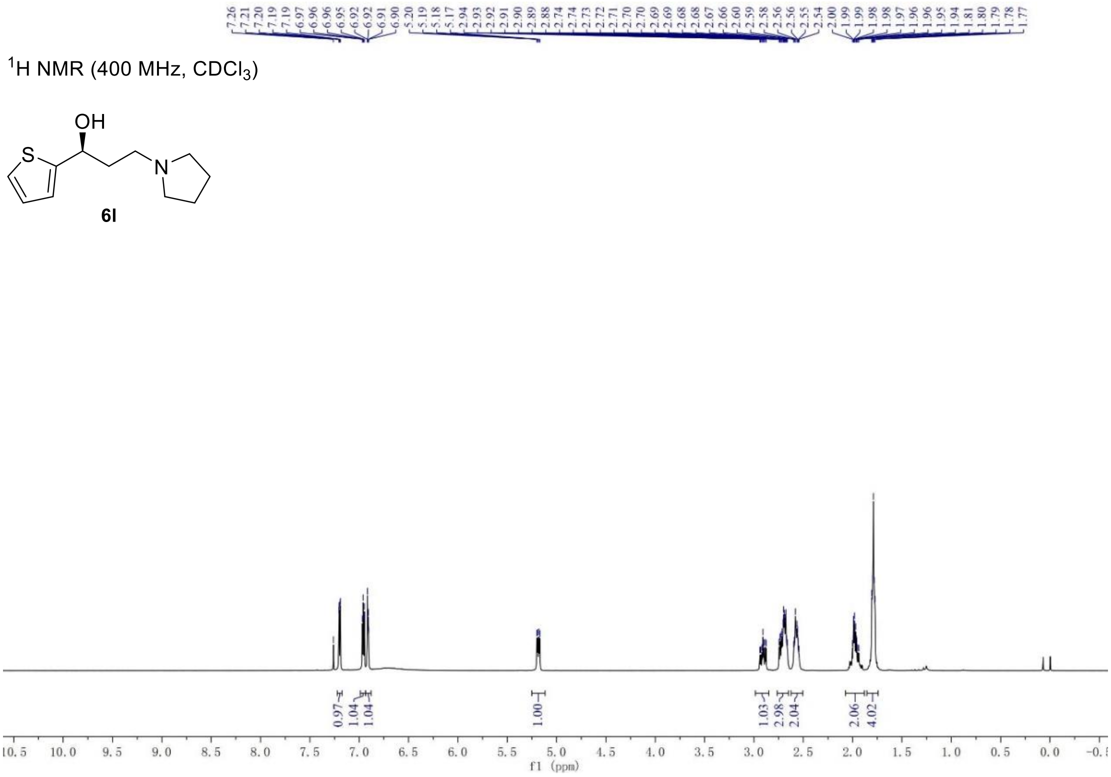

Supplementary Figure 153. ^{1}H NMR spectra of **6l**

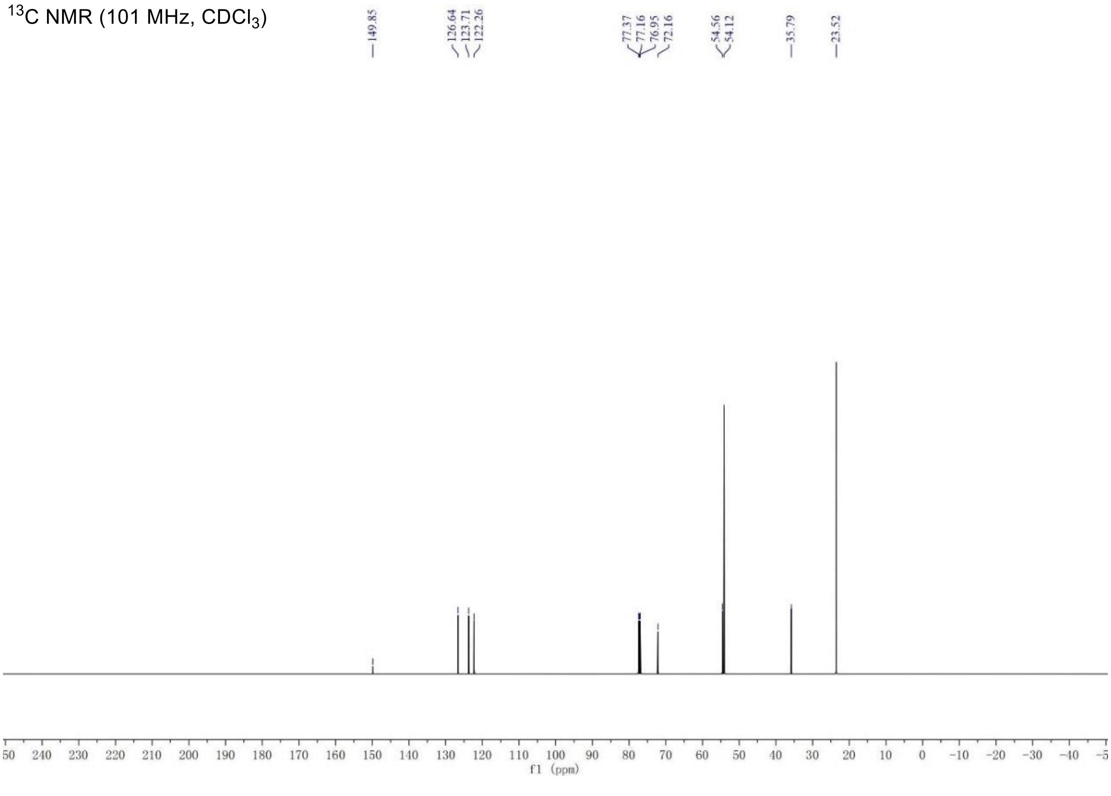

Supplementary Figure 154. ^{13}C NMR spectra of **6l**

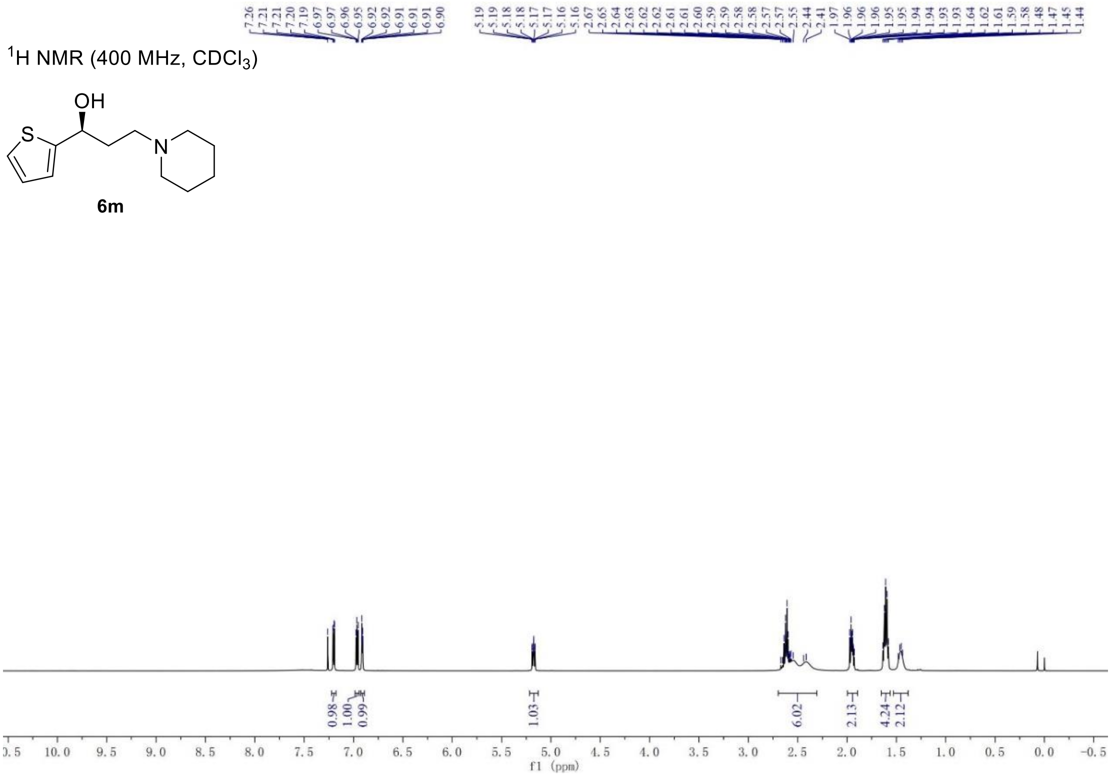

Supplementary Figure 155. ^{1}H NMR spectra of **6m**

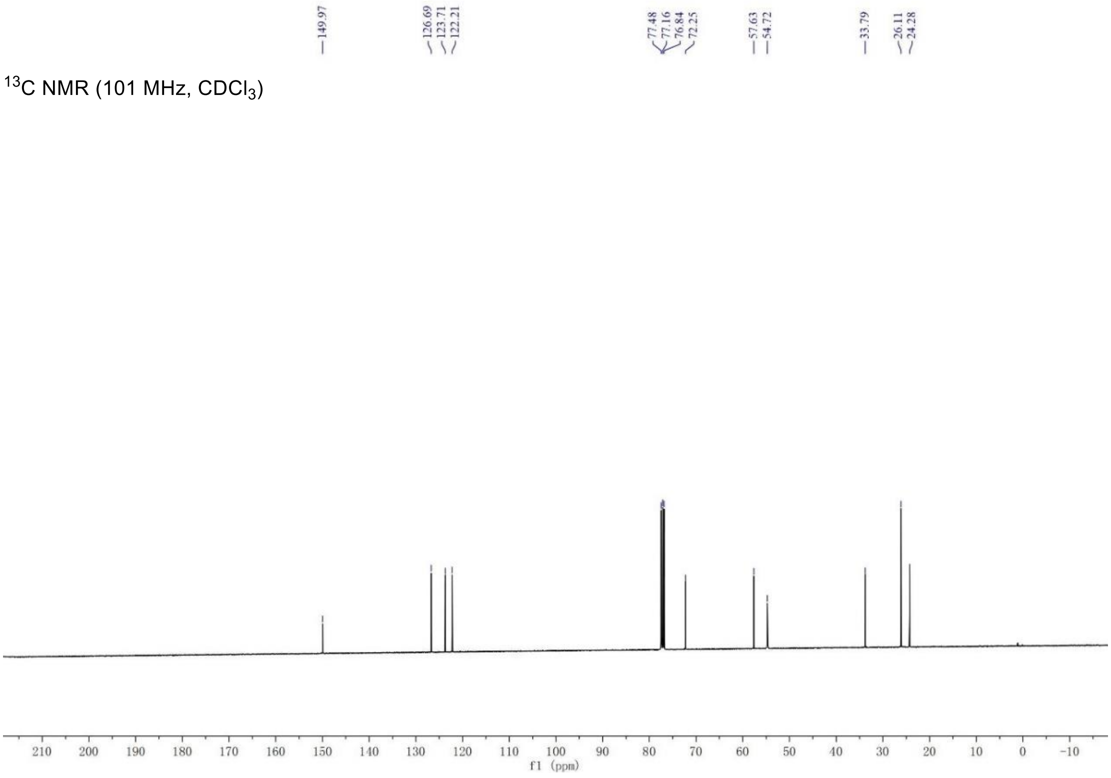

Supplementary Figure 156. ^{13}C NMR spectra of **6m**

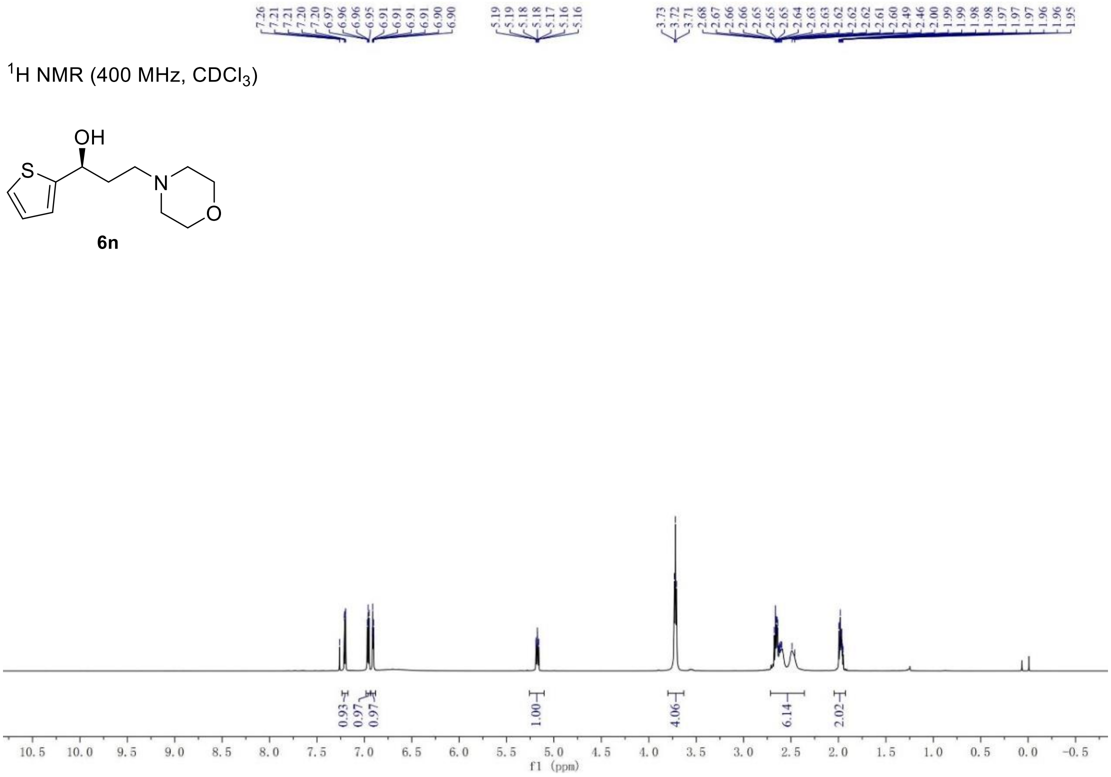

Supplementary Figure 157. ^{1}H NMR spectra of **6n**

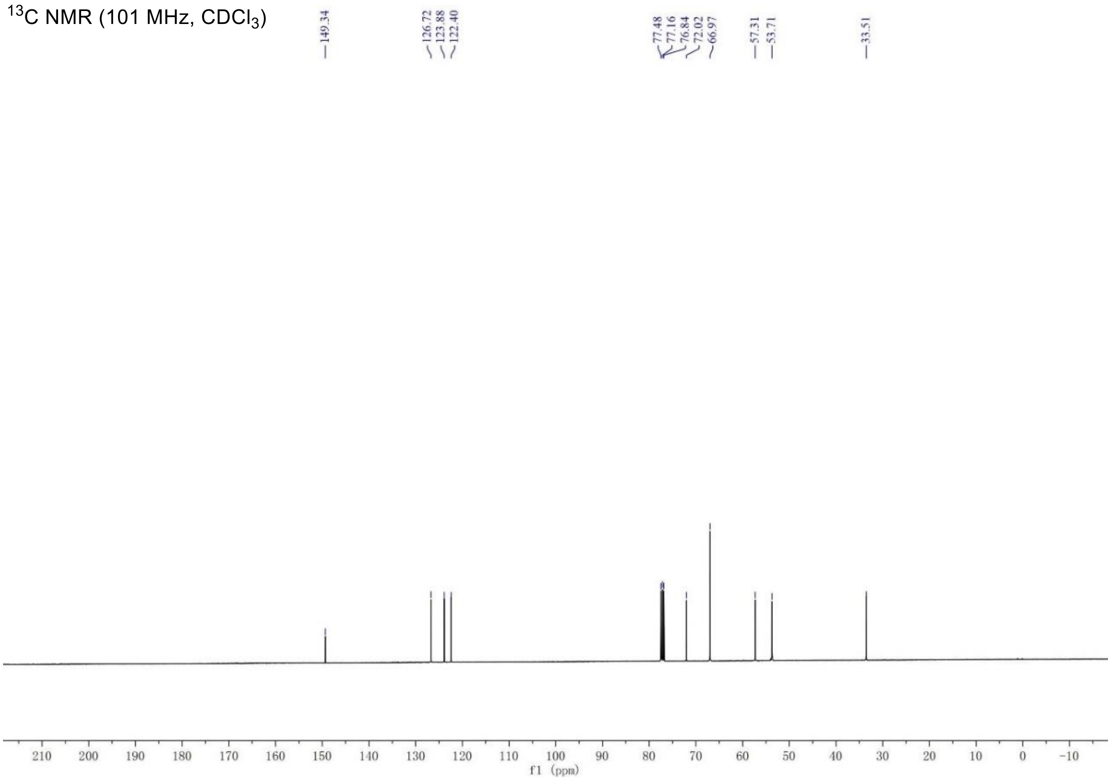

Supplementary Figure 158. ^{13}C NMR spectra of **6n**

^{1}H NMR (400 MHz, $CDCl_3$)

6o

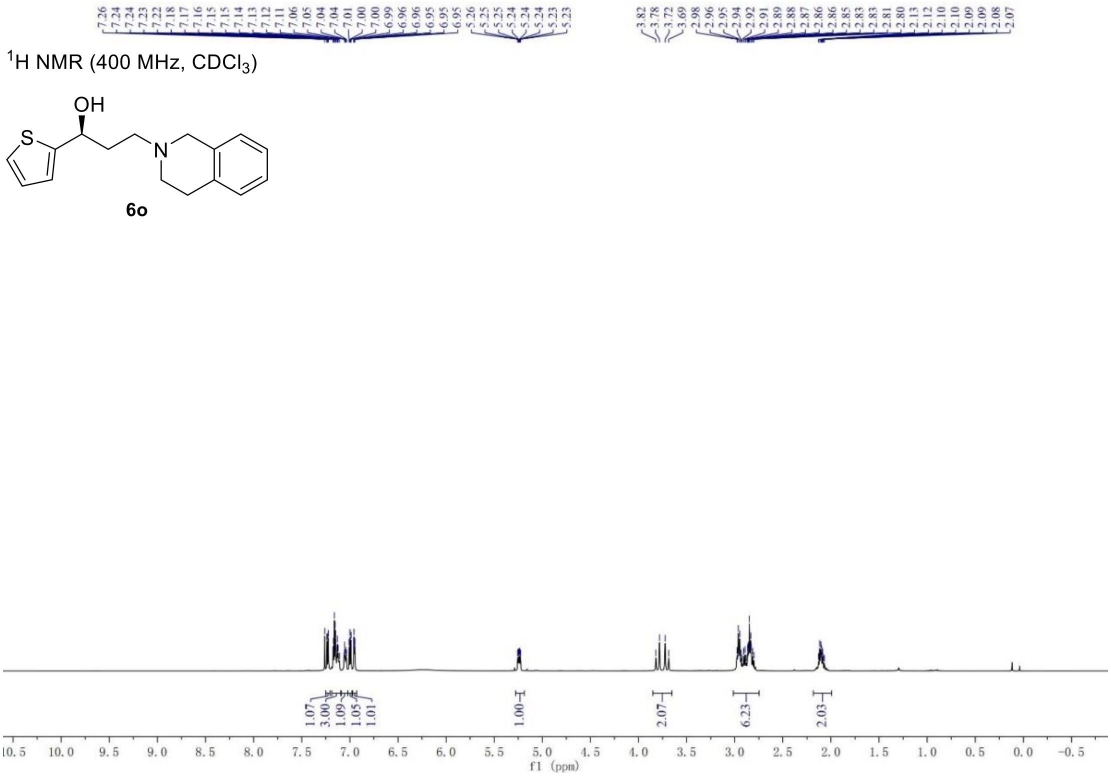

Supplementary Figure 159. ^{1}H NMR spectra of **6o**

^{13}C NMR (101 MHz, $CDCl_3$)

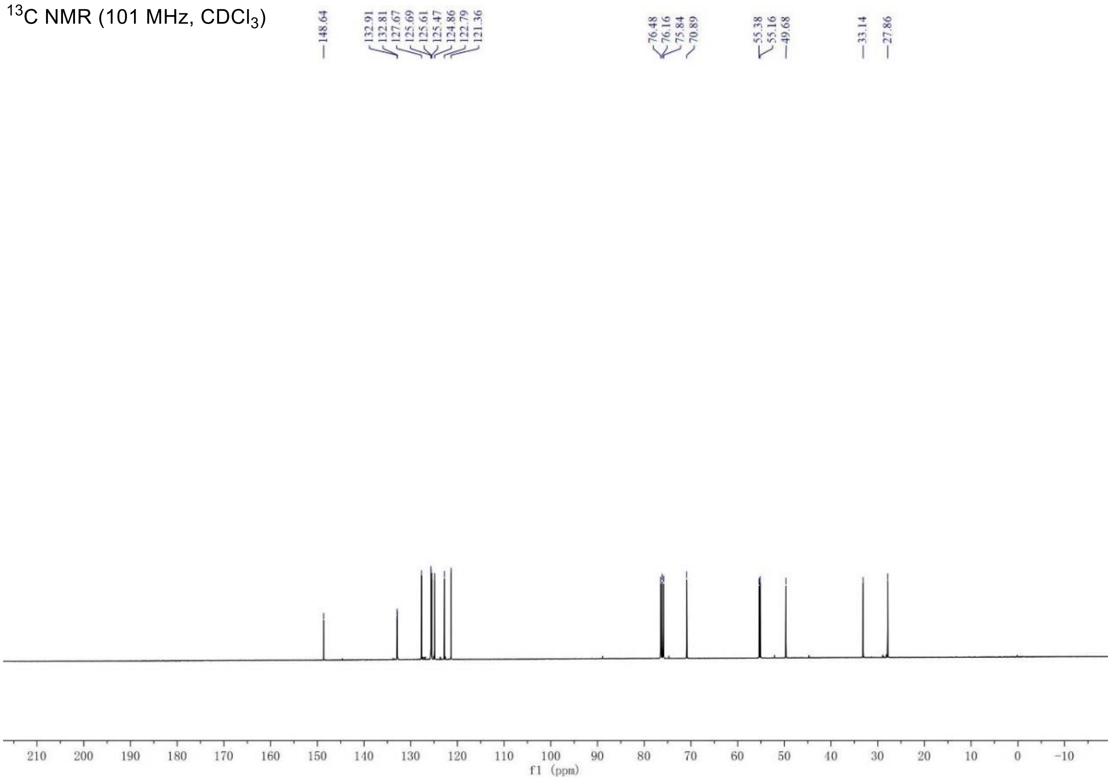

Supplementary Figure 160. ^{13}C NMR spectra of **6o**

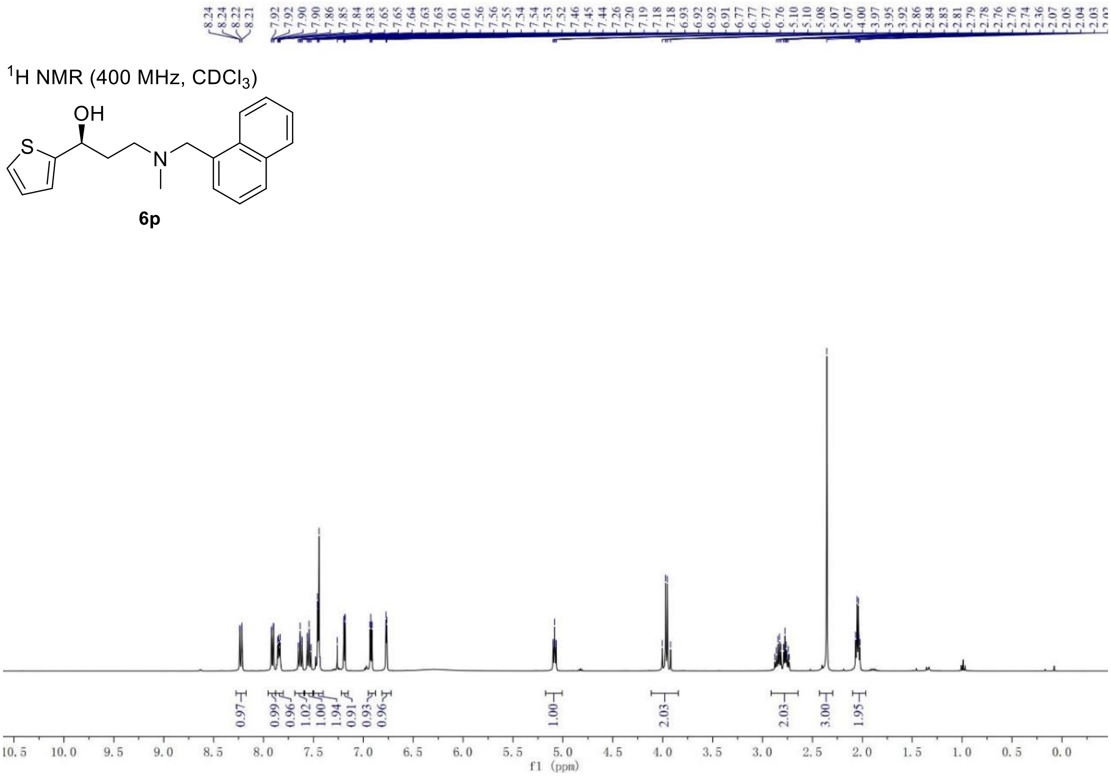

Supplementary Figure 161. ^{1}H NMR spectra of **6p**

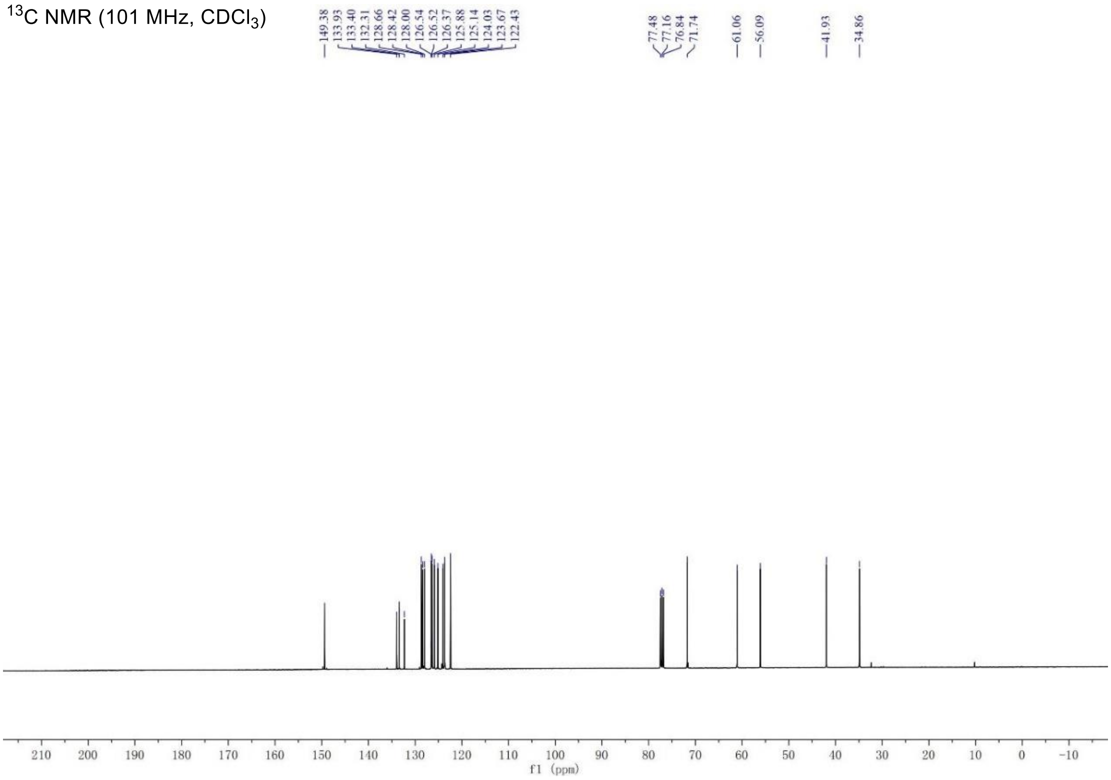

Supplementary Figure 162. ^{13}C NMR spectra of **6p**

^{1}H NMR (400 MHz, $CDCl_3$)

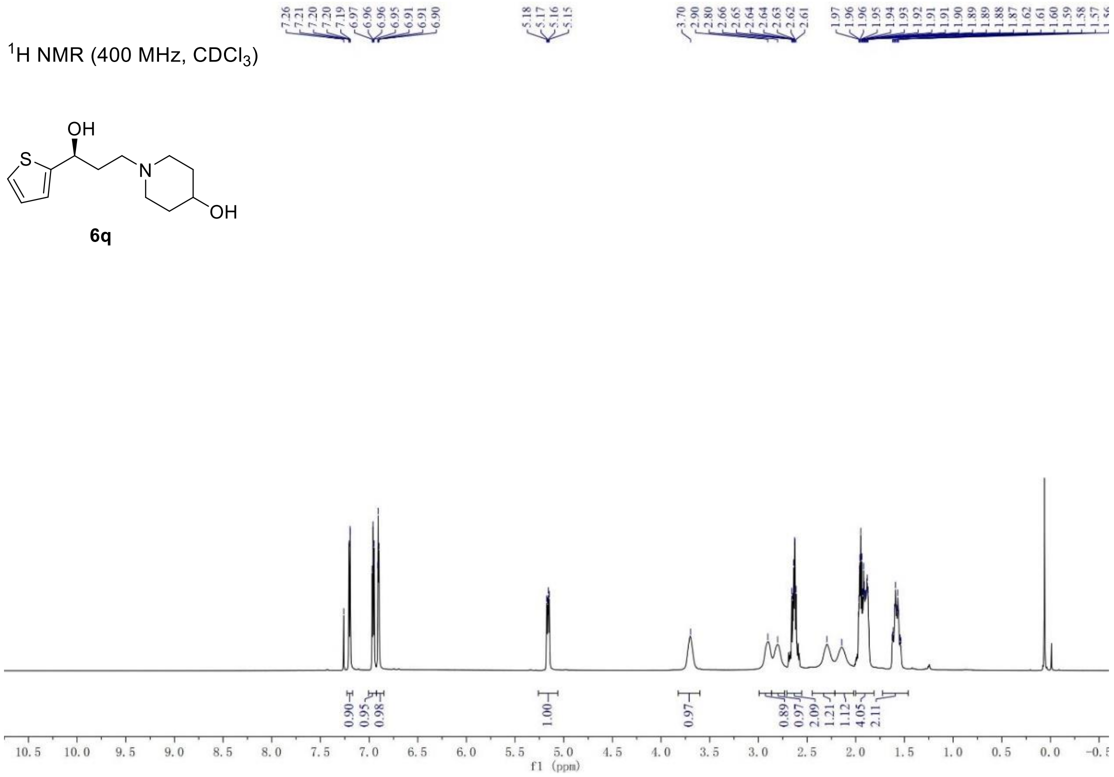

Supplementary Figure 163. ^{1}H NMR spectra of **6q**

^{13}C NMR (101 MHz, $CDCl_3$)

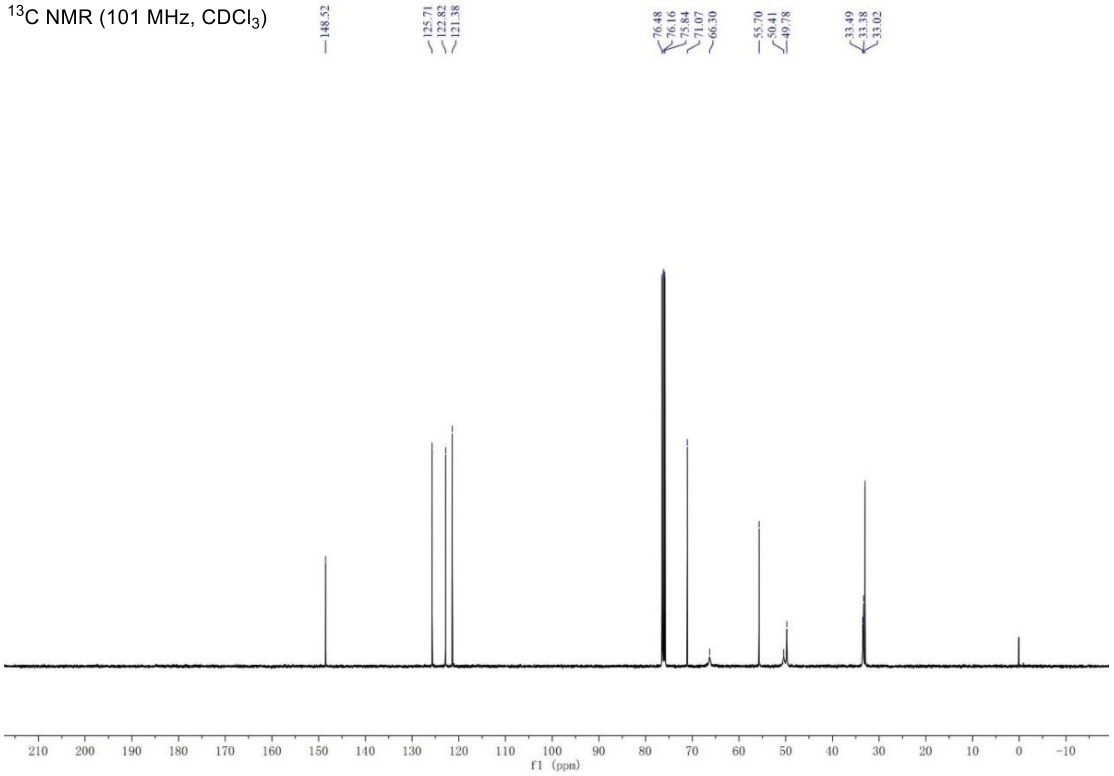

Supplementary Figure 164. ^{13}C NMR spectra of **6q**

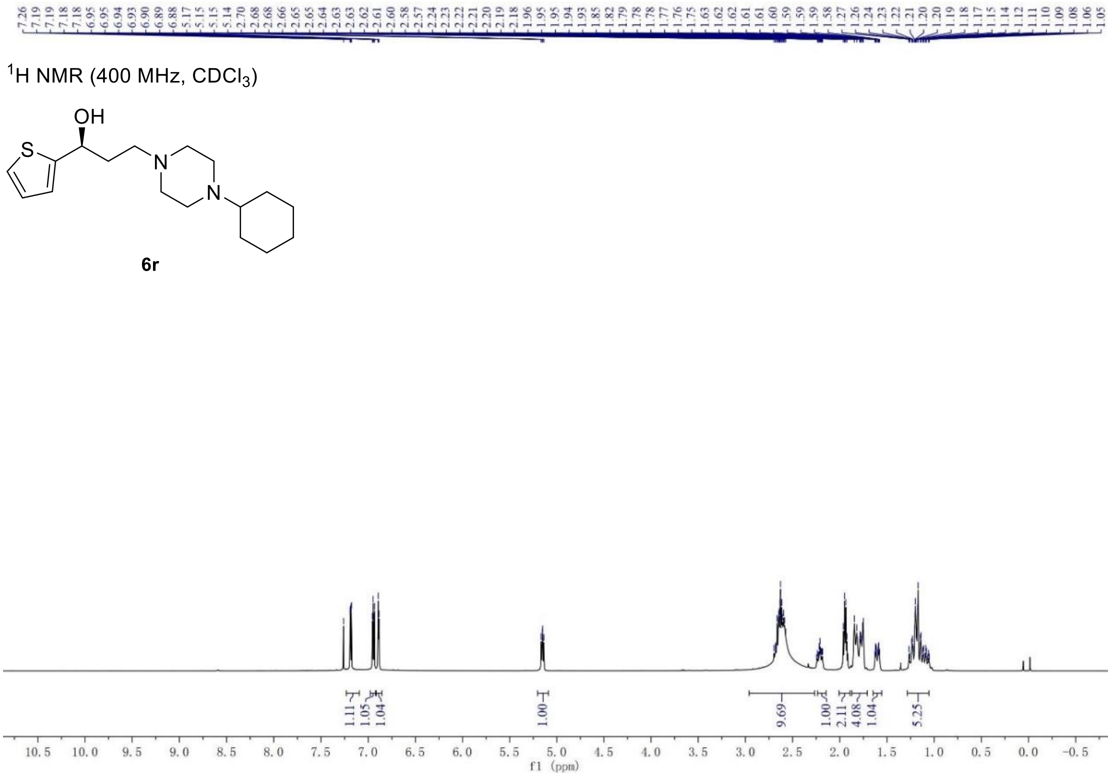

Supplementary Figure 165. ^{1}H NMR spectra of **6r**

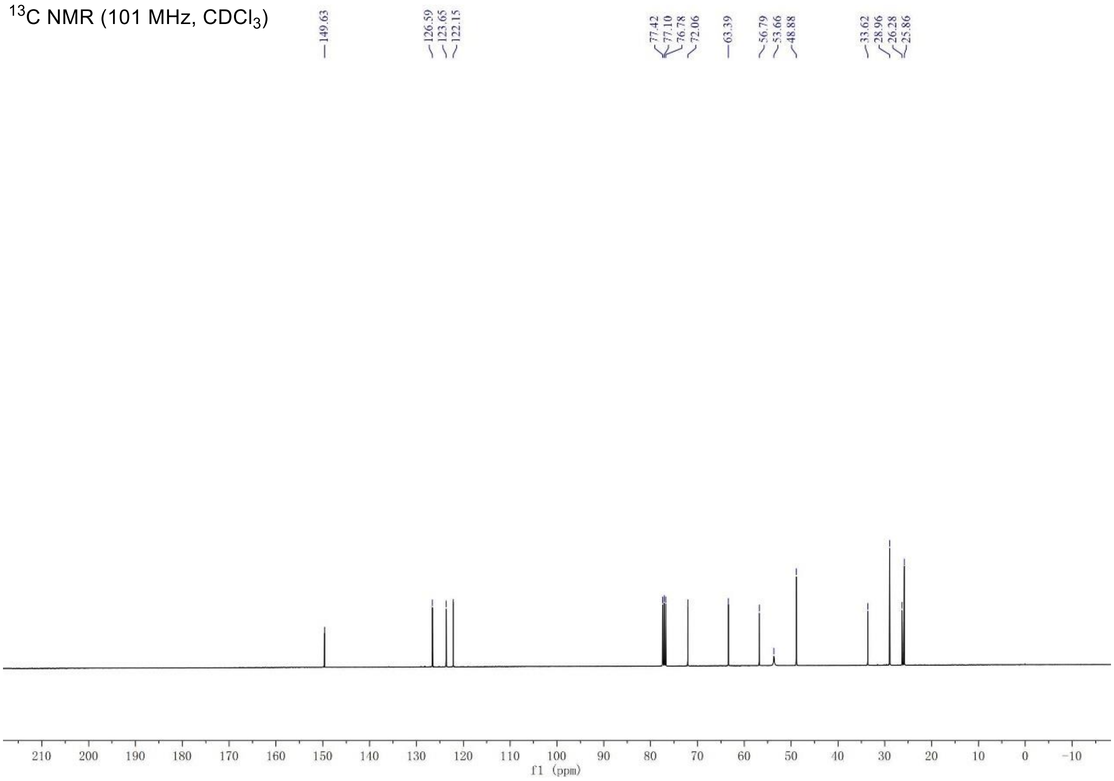

Supplementary Figure 166. ^{13}C NMR spectra of **6r**

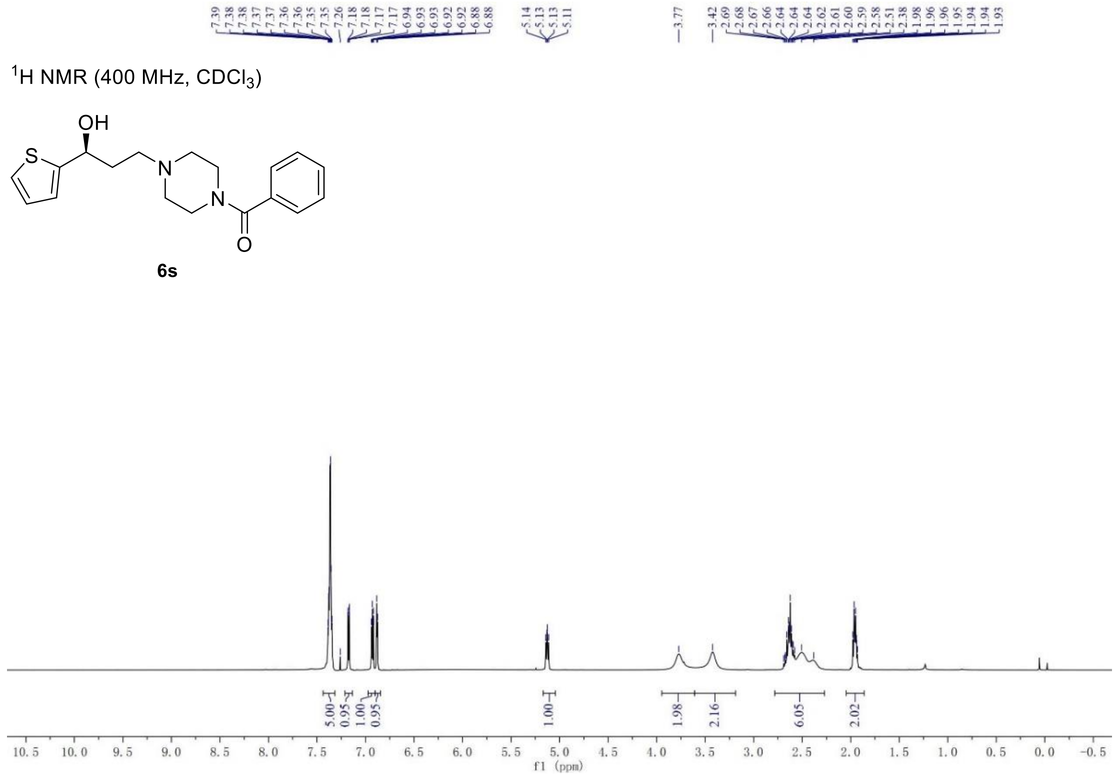

Supplementary Figure 167. ^{1}H NMR spectra of **6s**

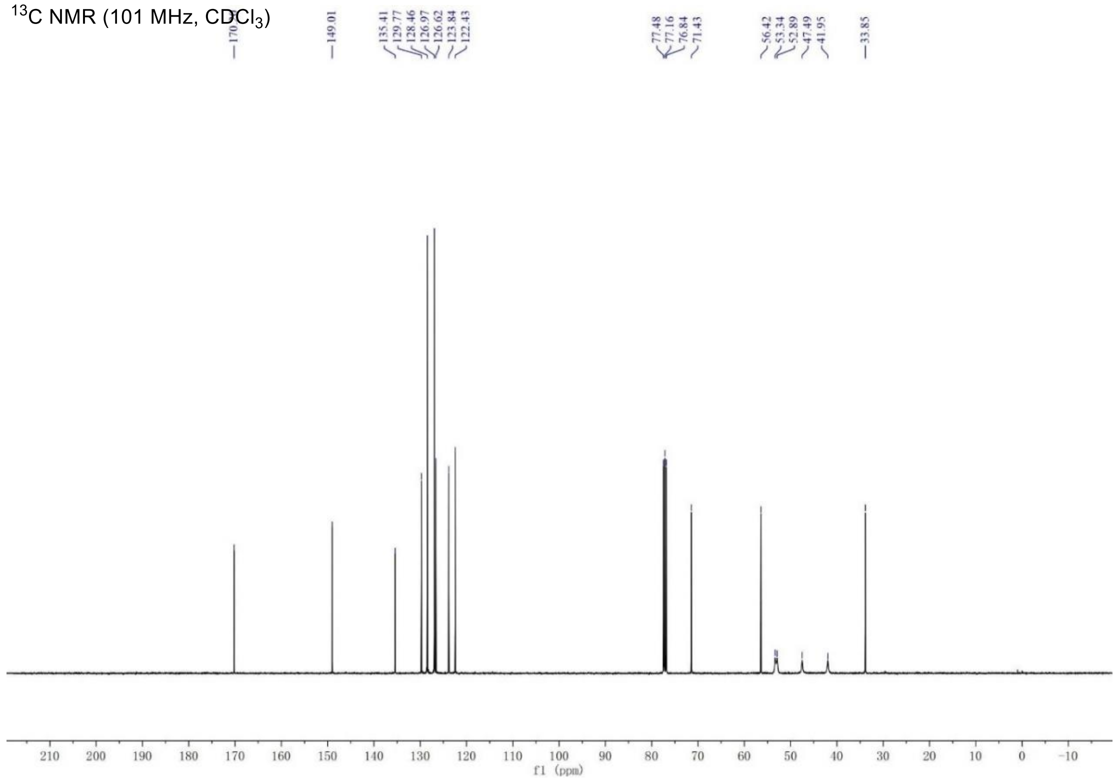

Supplementary Figure 168. ^{13}C NMR spectra of **6s**

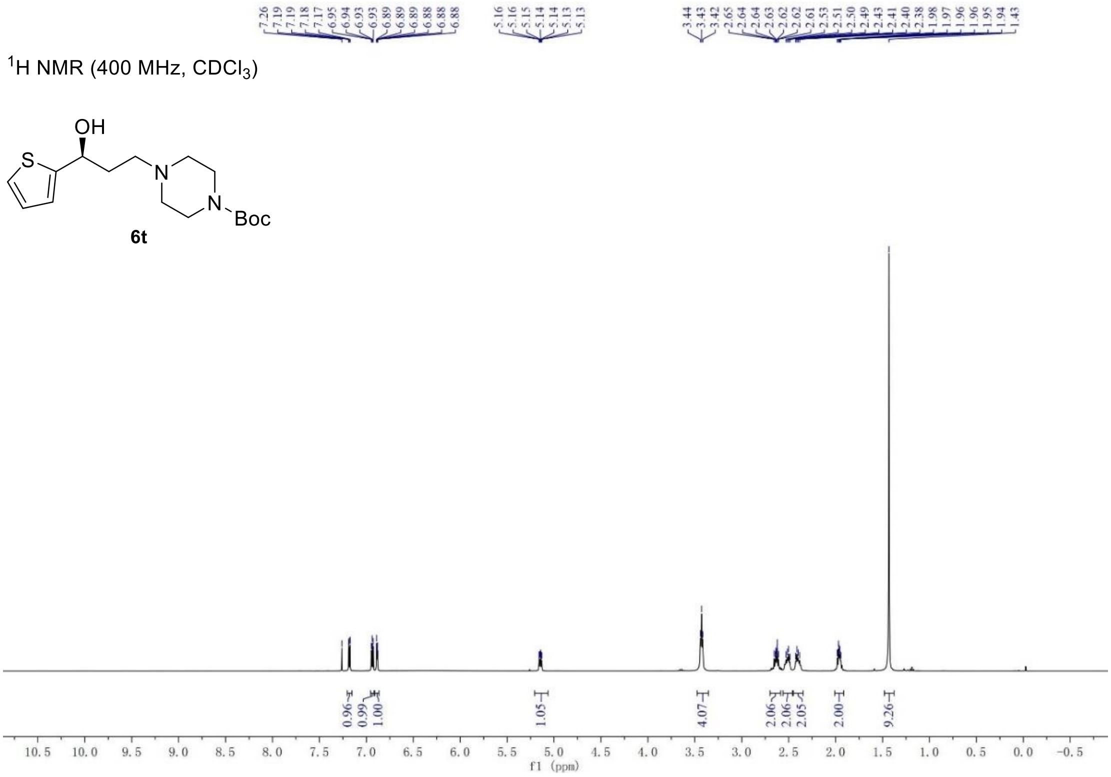

Supplementary Figure 169. ^{1}H NMR spectra of **6t**

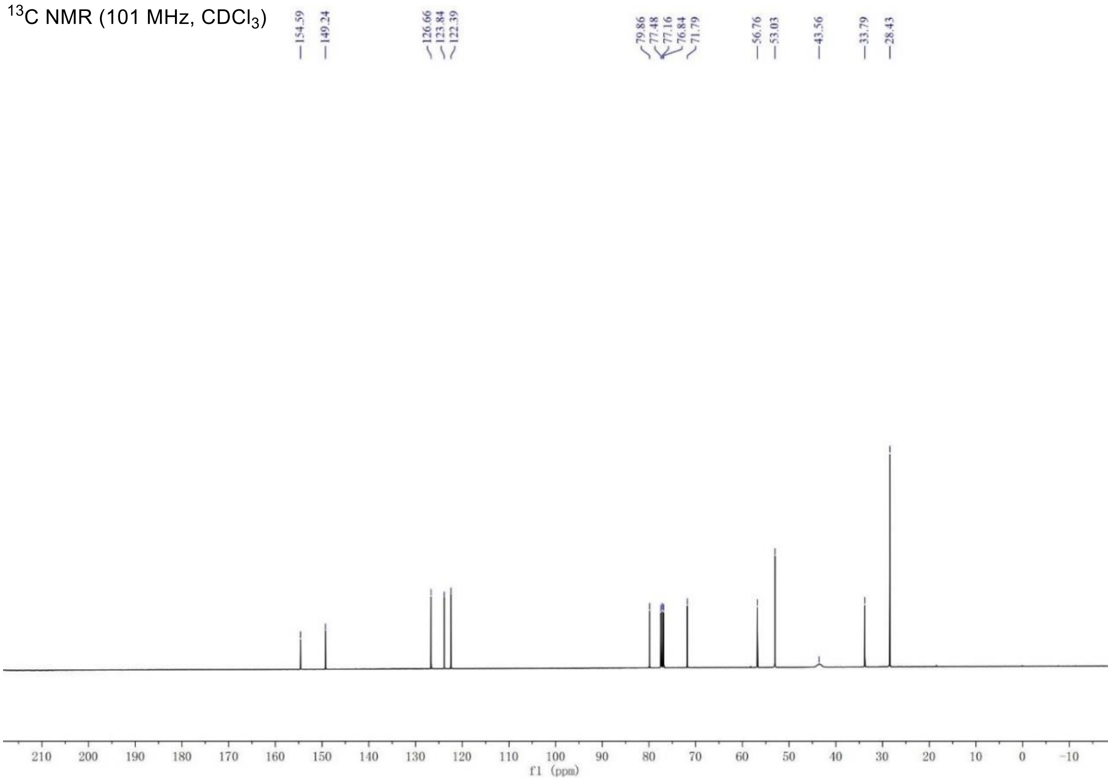

Supplementary Figure 170. ^{13}C NMR spectra of **6t**

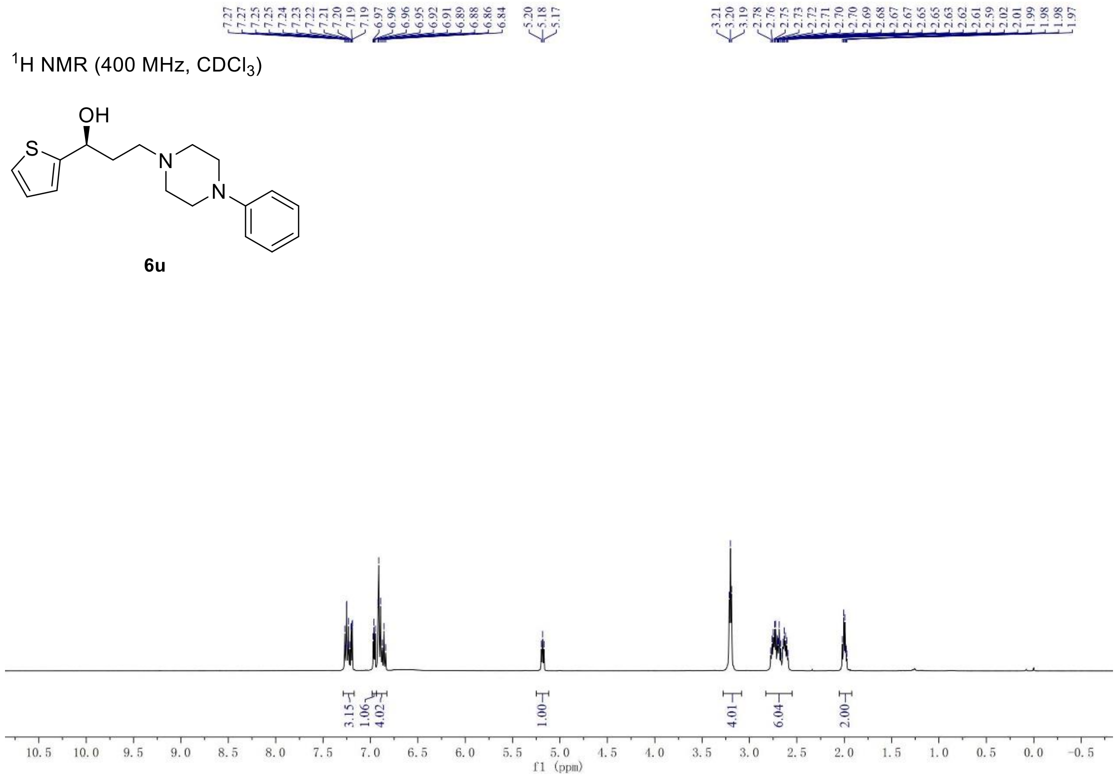

Supplementary Figure 171. ^{1}H NMR spectra of **6u**

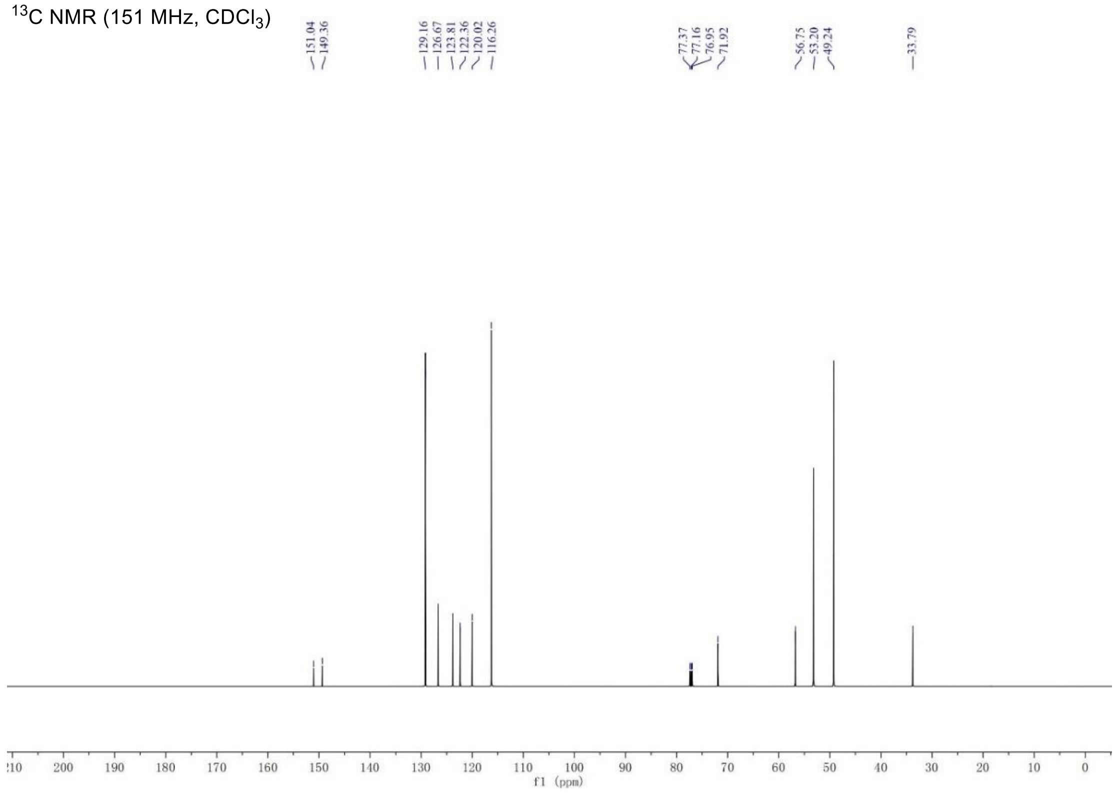

Supplementary Figure 172. ^{13}C NMR spectra of **6u**

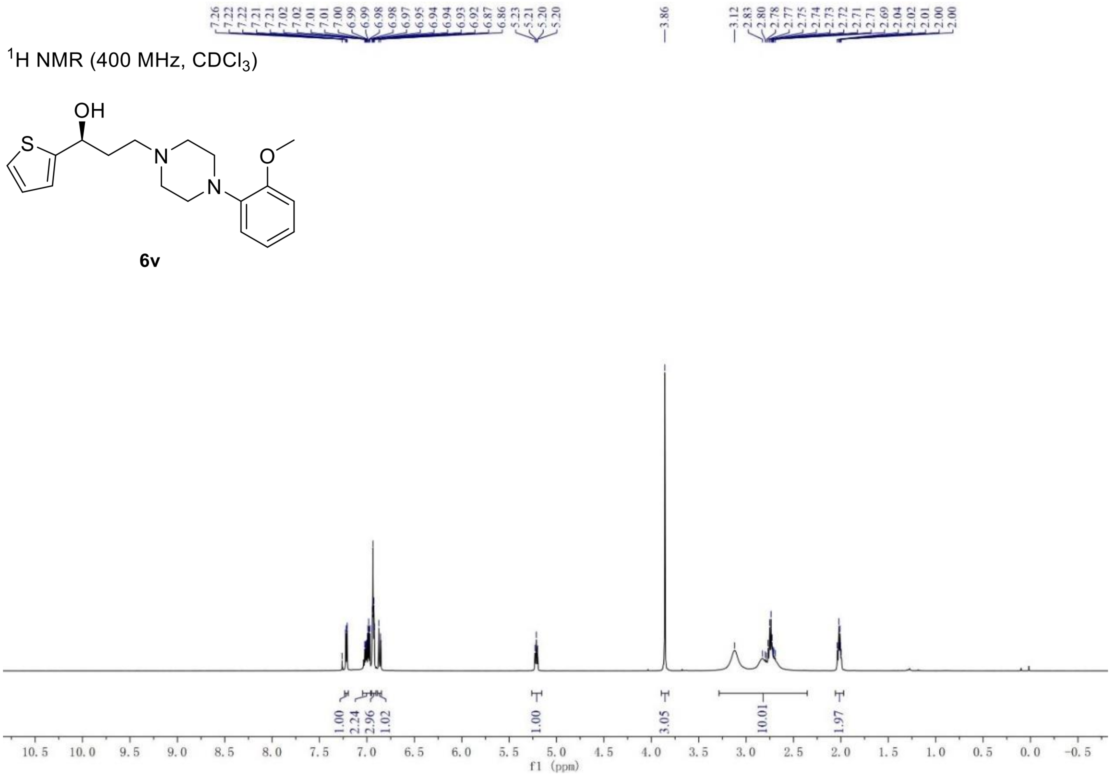

Supplementary Figure 173. ^{1}H NMR spectra of **6v**

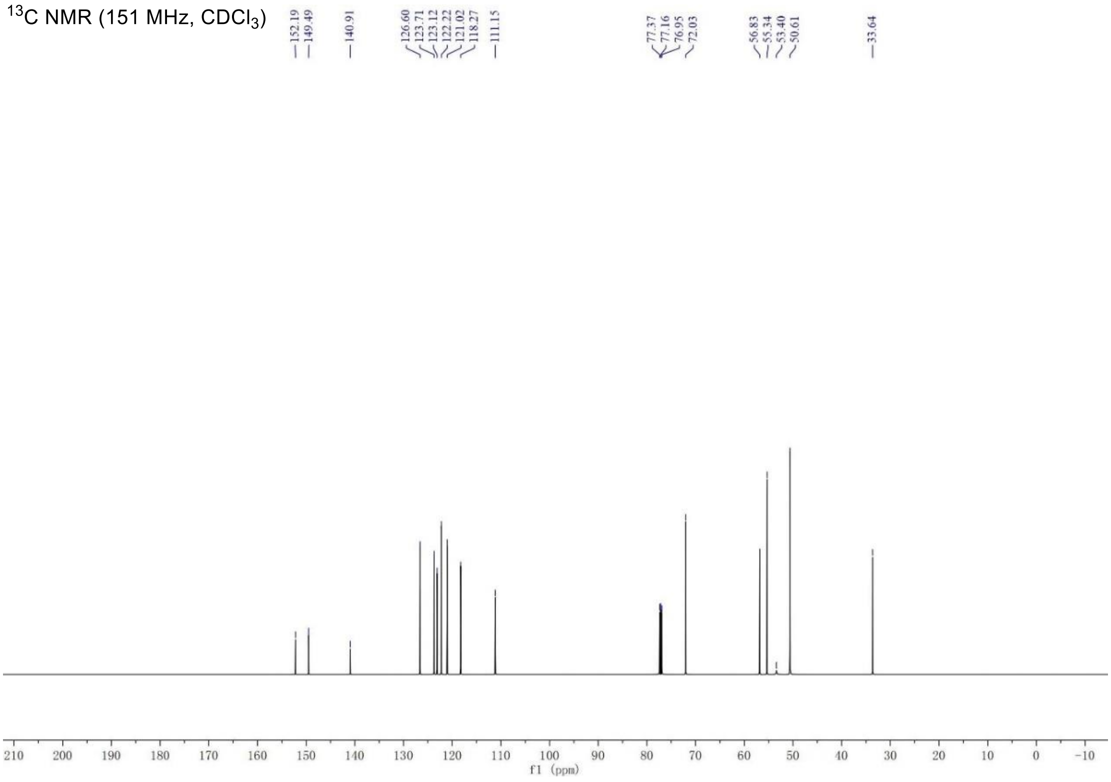

Supplementary Figure 174. ^{13}C NMR spectra of **6v**

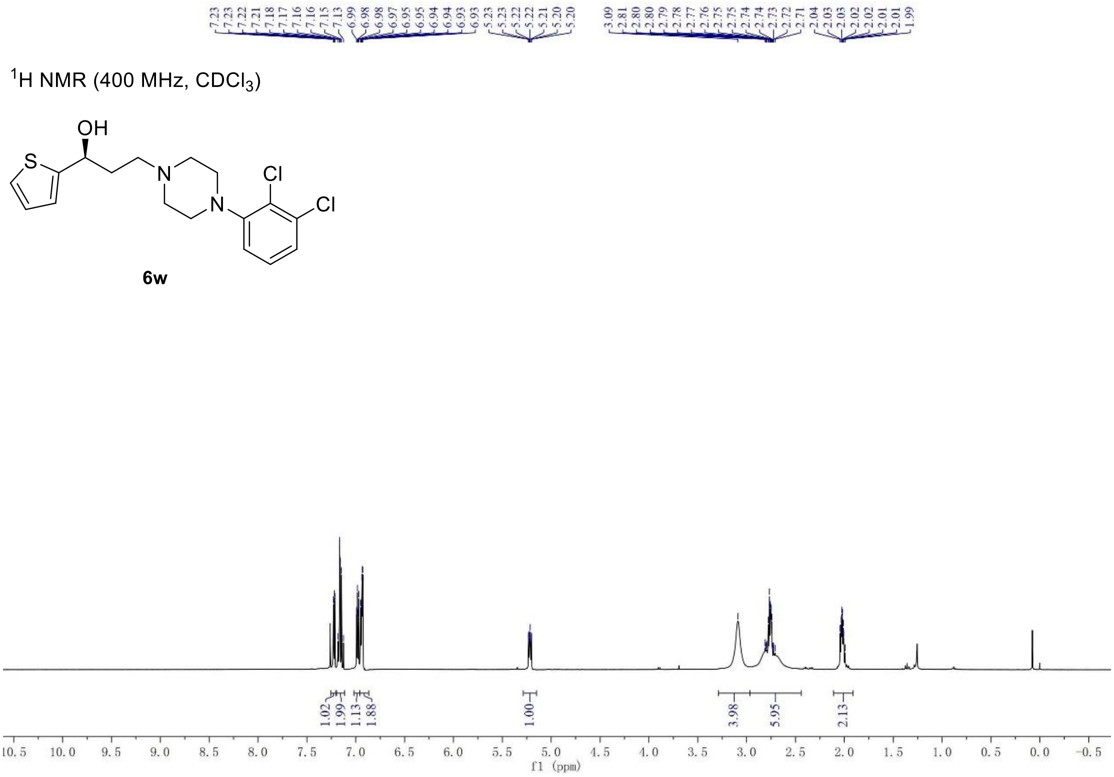

Supplementary Figure 175. ^{1}H NMR spectra of **6w**

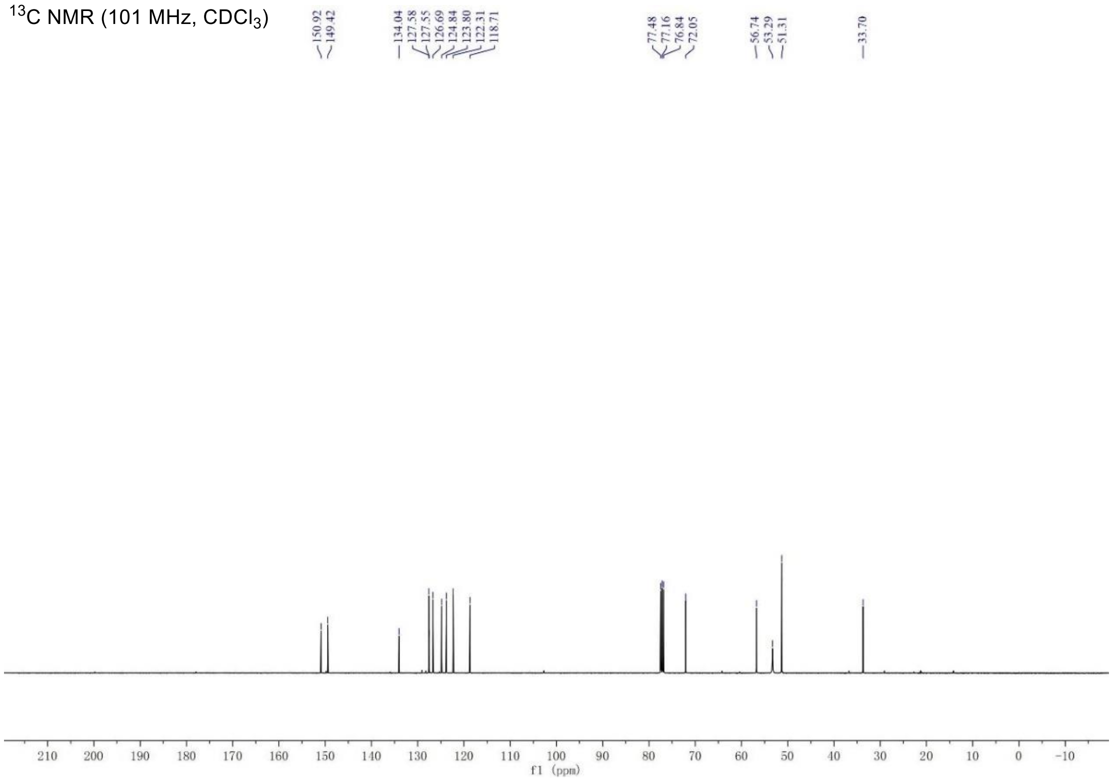

Supplementary Figure 176. ^{13}C NMR spectra of **6w**

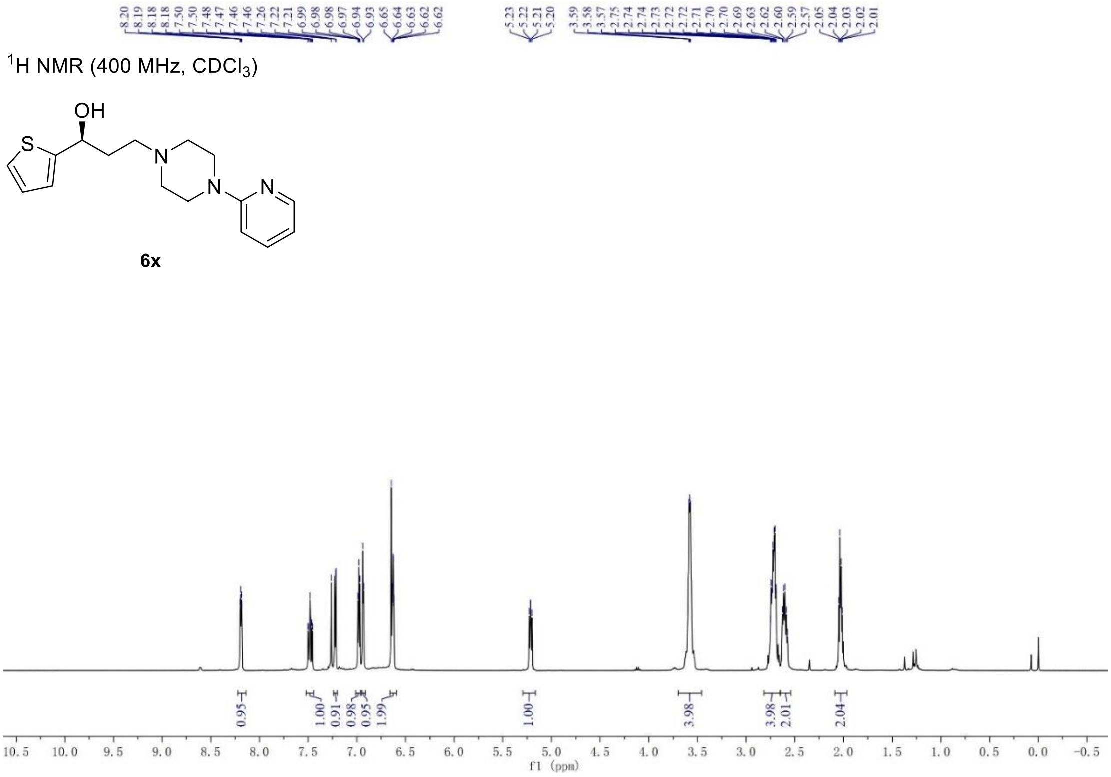

Supplementary Figure 177. ^{1}H NMR spectra of **6x**

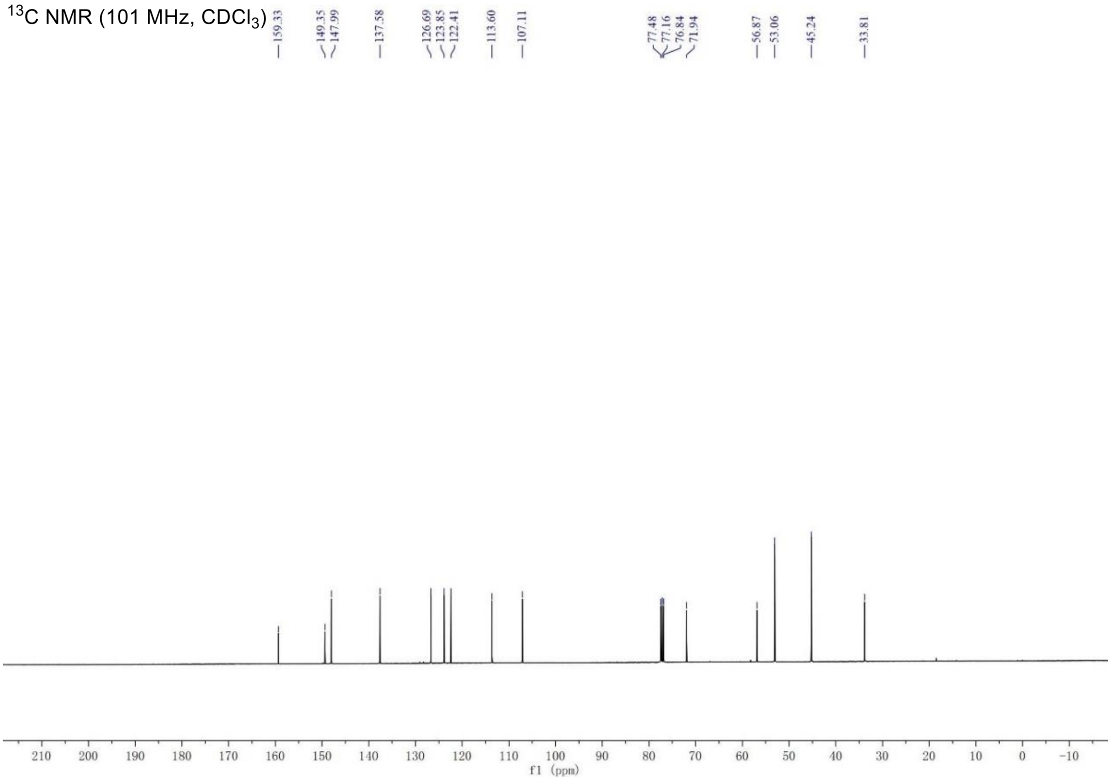

Supplementary Figure 178. ^{13}C NMR spectra of **6x**

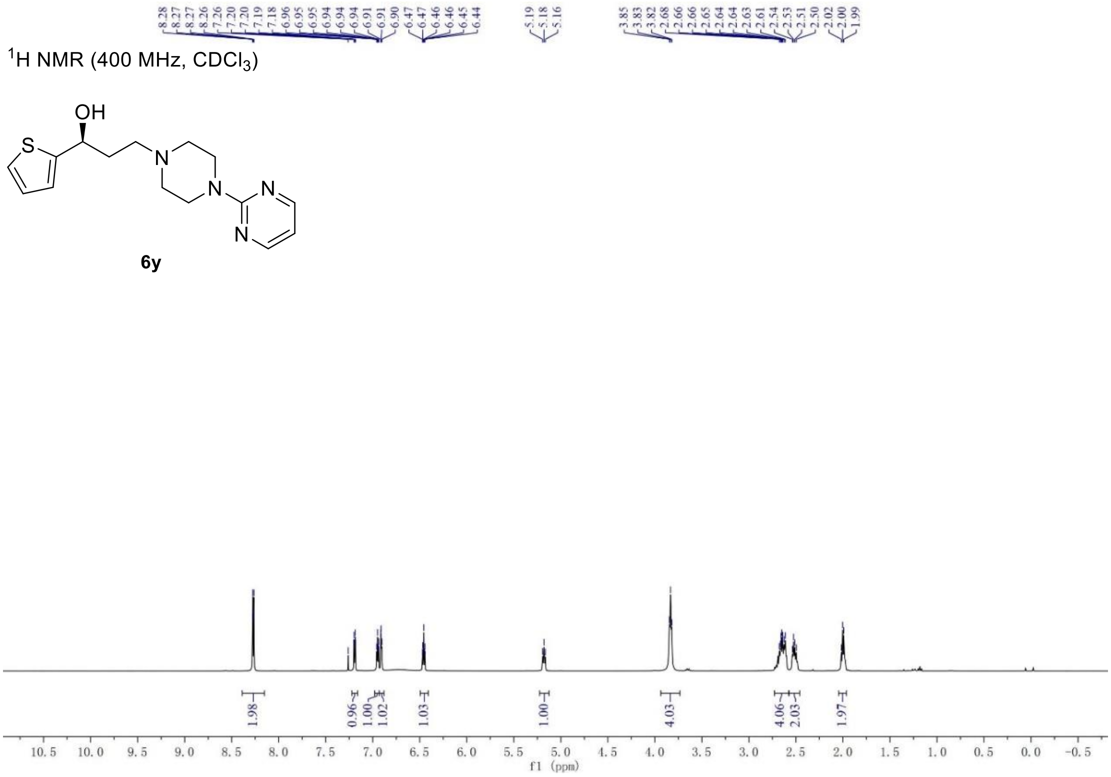

Supplementary Figure 179. ^{1}H NMR spectra of **6y**

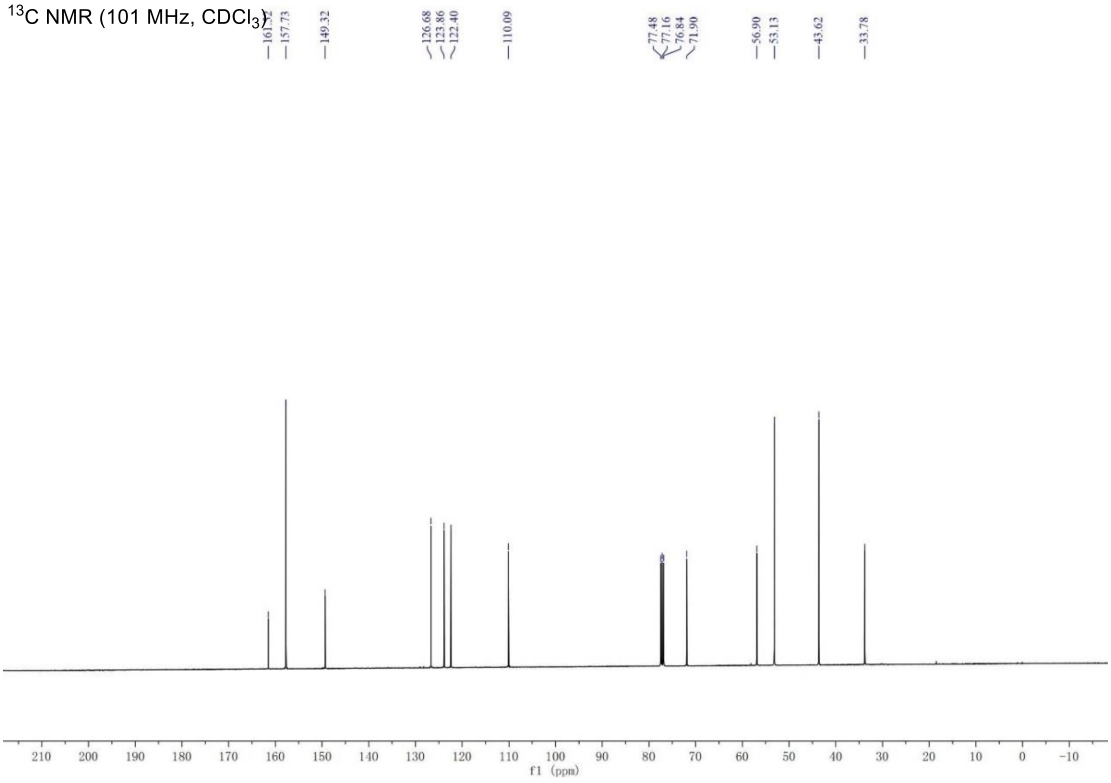

Supplementary Figure 180. ^{13}C NMR spectra of **6y**

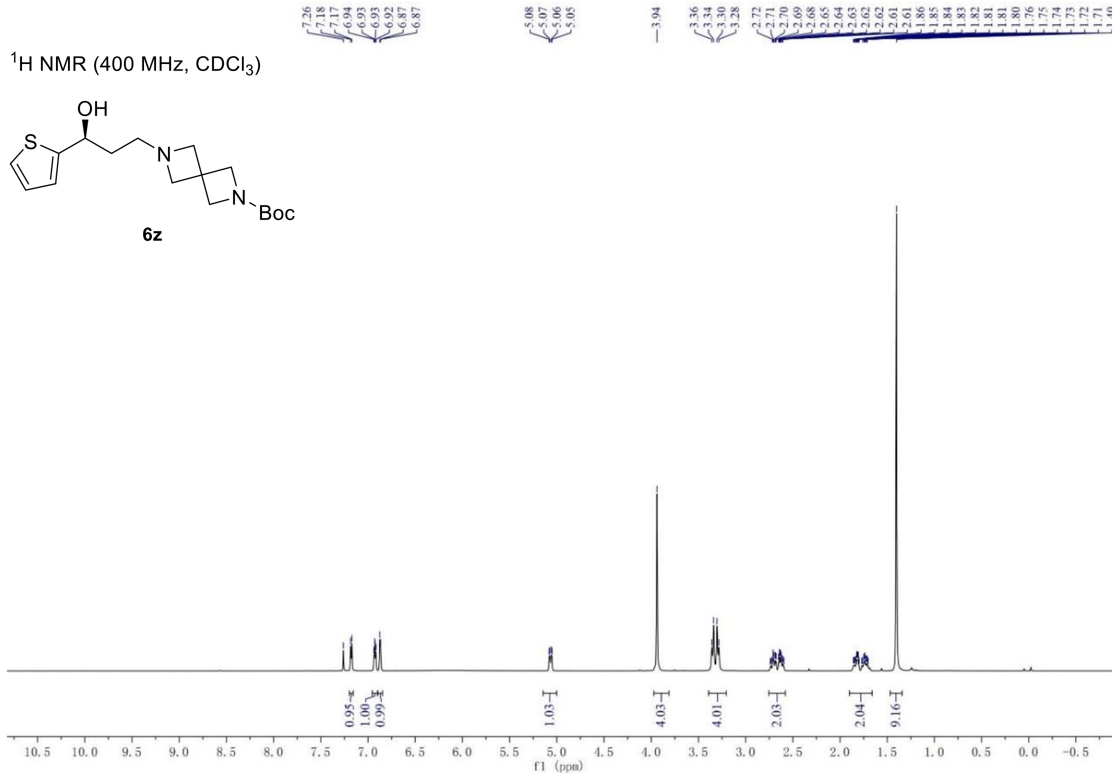

Supplementary Figure 181. ^{1}H NMR spectra of **6z**

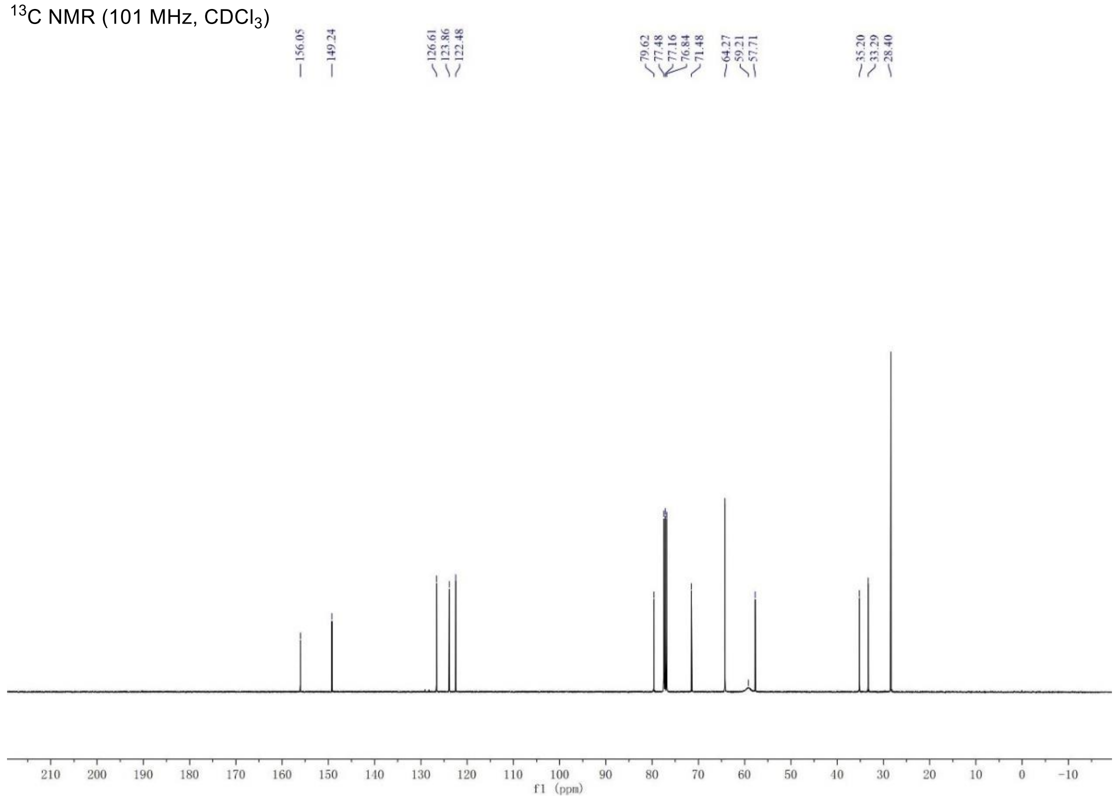

Supplementary Figure 182. ^{13}C NMR spectra of **6z**

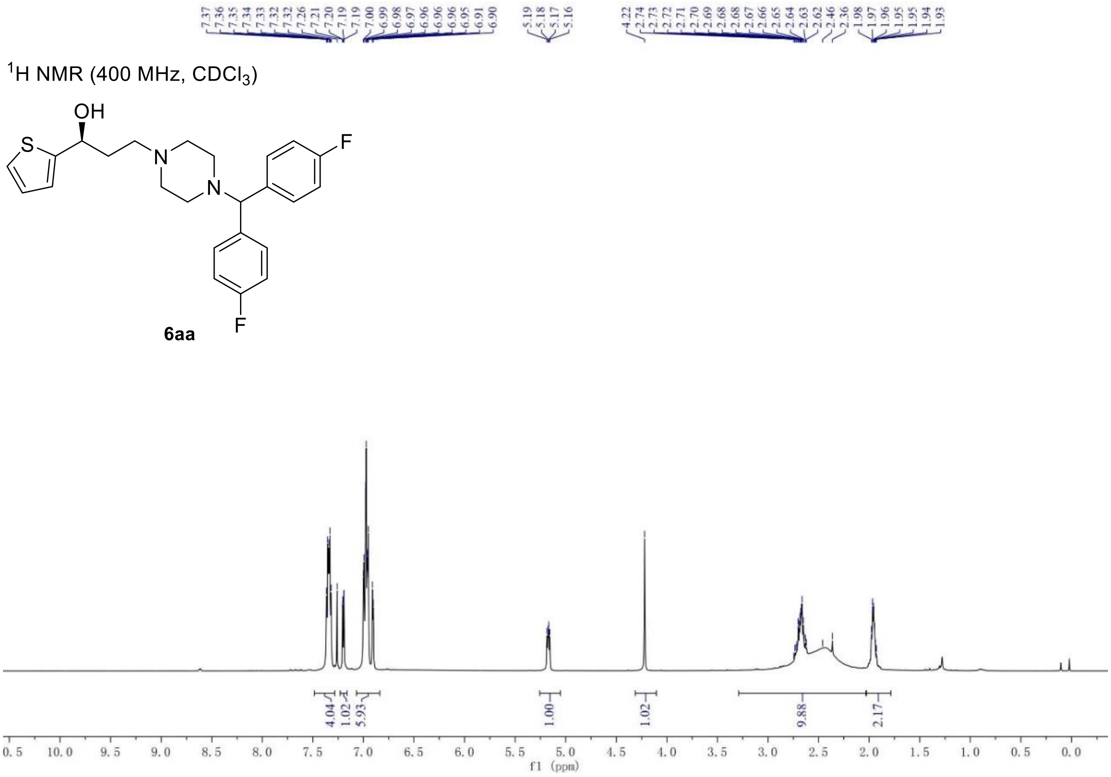

Supplementary Figure 183. ^{1}H NMR spectra of **6aa**

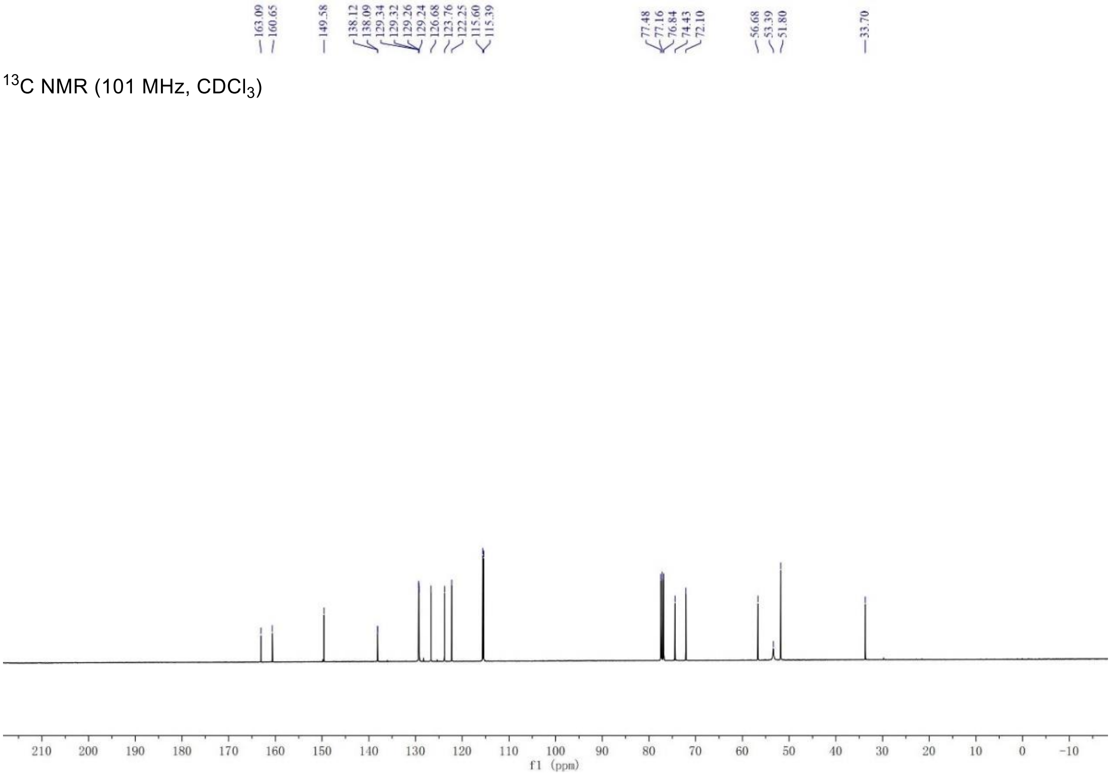

Supplementary Figure 184. ^{13}C NMR spectra of **6aa**

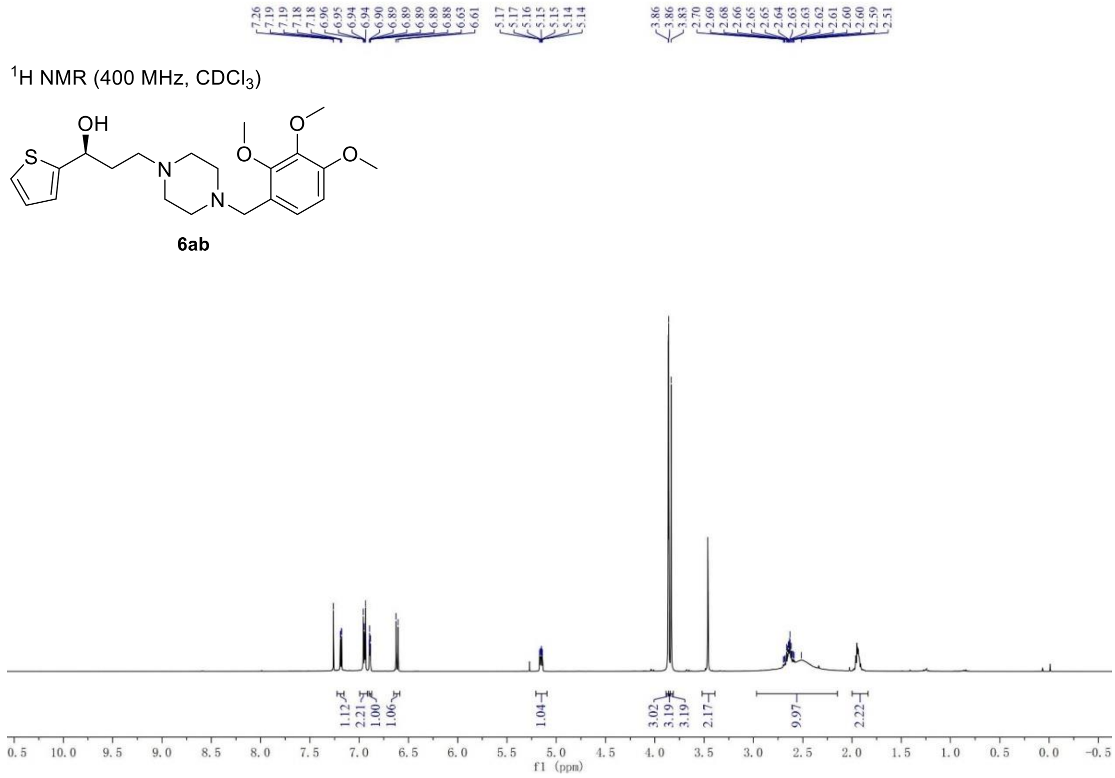

Supplementary Figure 185. ^{1}H NMR spectra of **6ab**

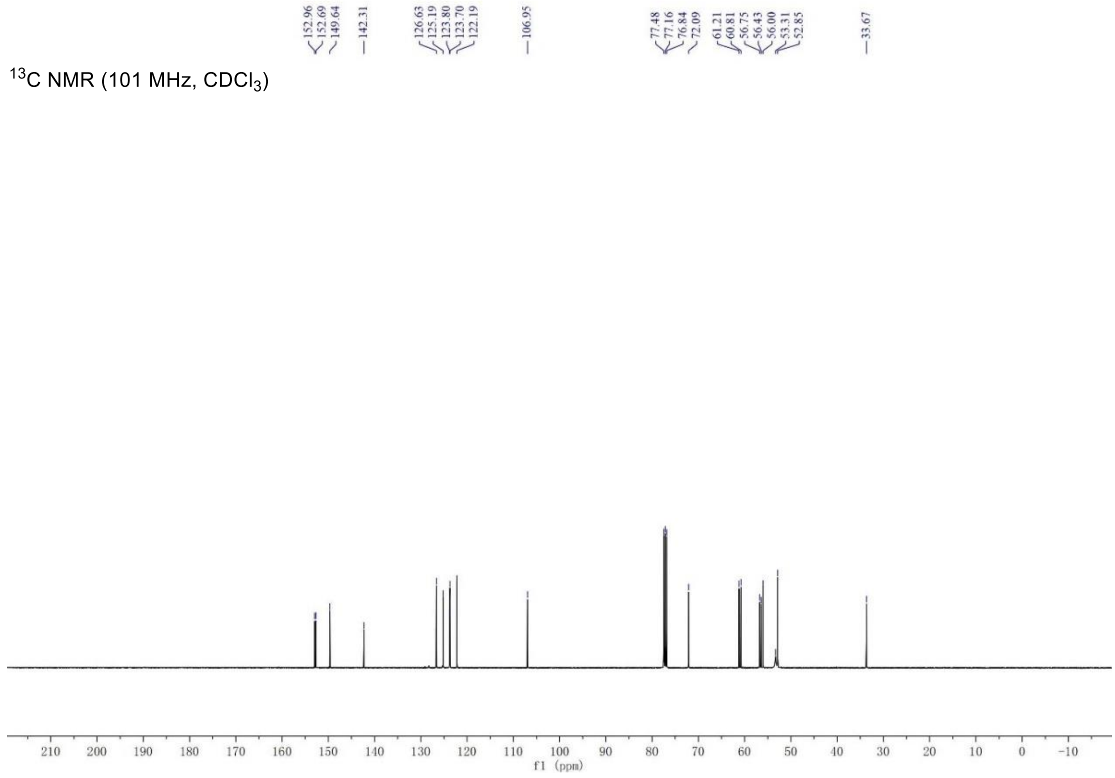

Supplementary Figure 186. ^{13}C NMR spectra of **6ab**

^{1}H NMR (400 MHz, $CDCl_3$)

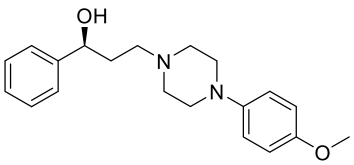

6ac

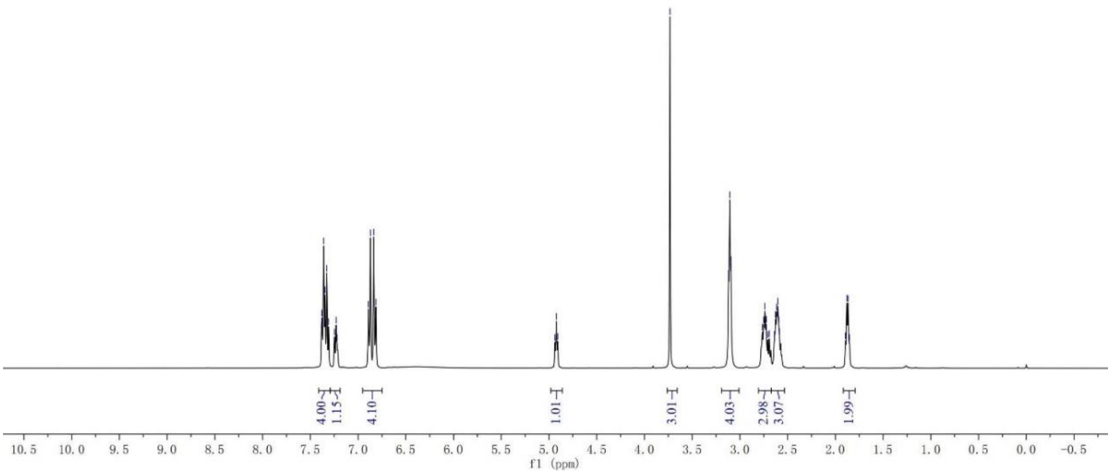

Supplementary Figure 187. ^{1}H NMR spectra of **6ac**

^{13}C NMR (101 MHz, $CDCl_3$)

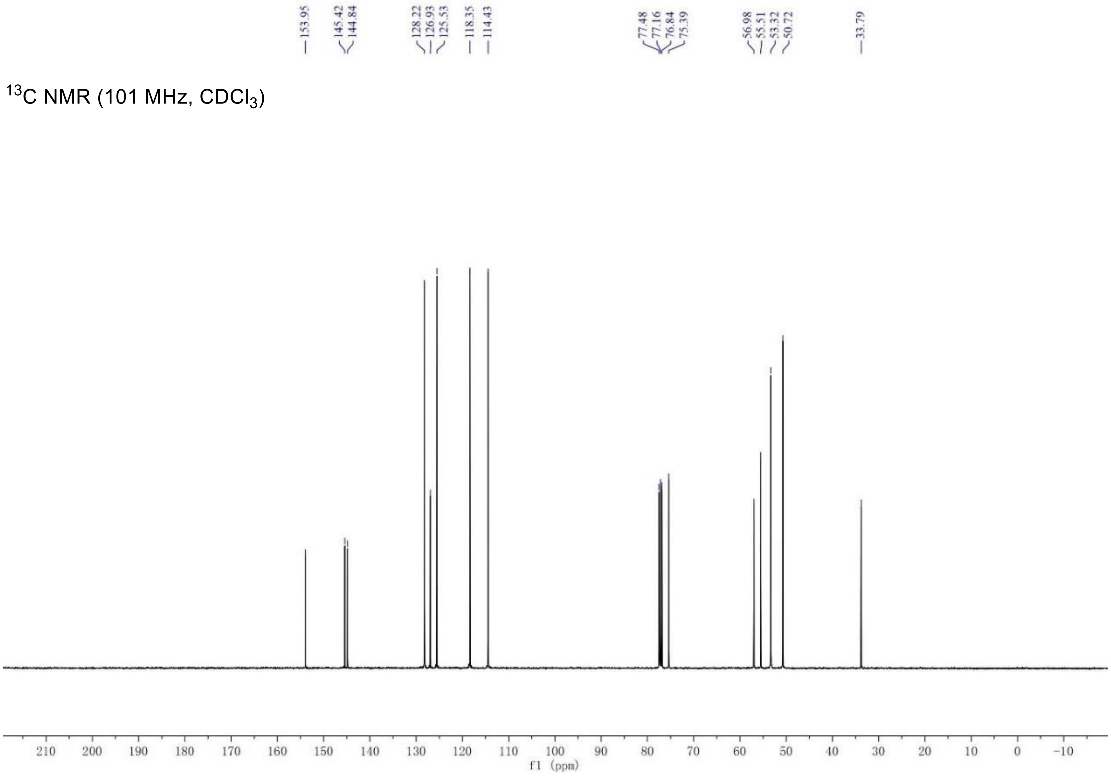

Supplementary Figure 188. ^{13}C NMR spectra of **6ac**

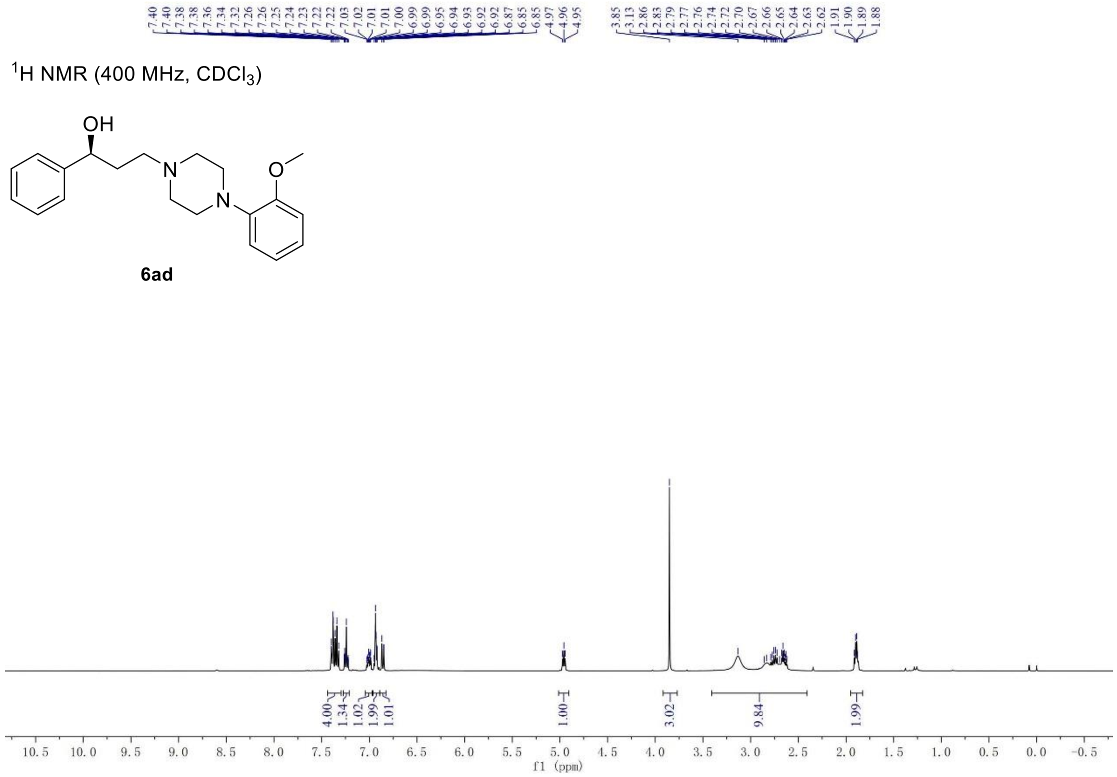

Supplementary Figure 189. ^{1}H NMR spectra of **6ad**

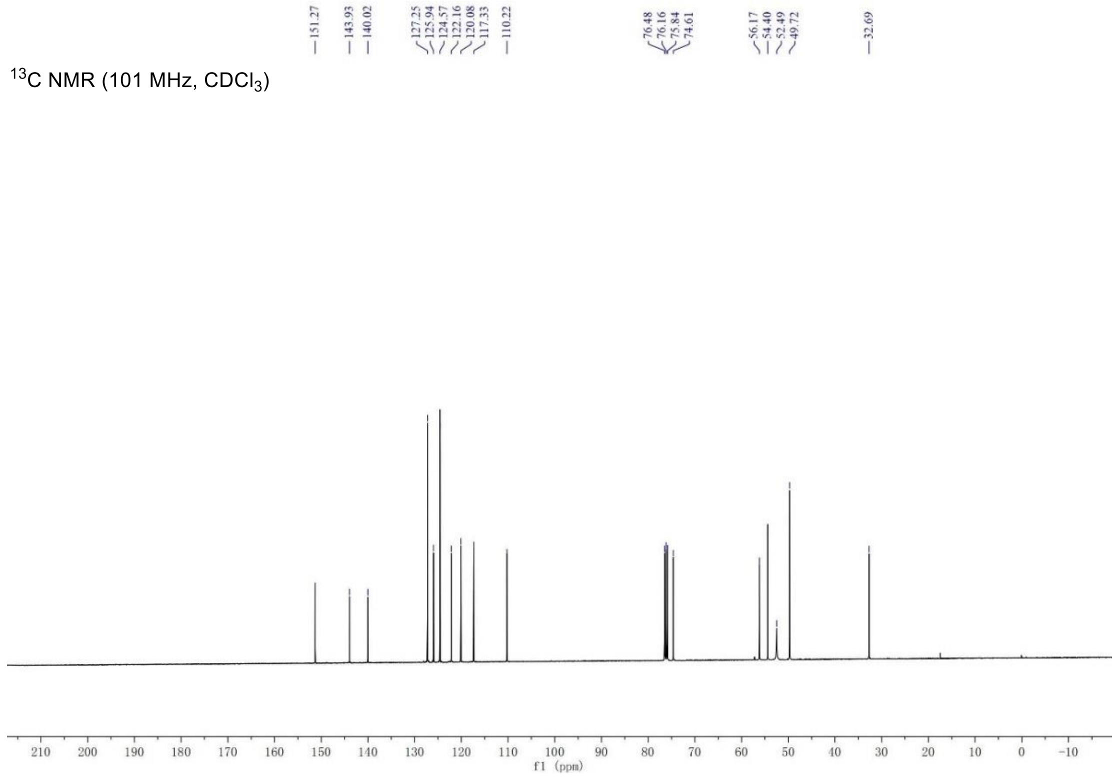

Supplementary Figure 190. ^{13}C NMR spectra of **6ad**

2af: The two carbon peaks of compound **2af** were not found in ^{13}C NMR spectra, and HMQC was used to confirm the structure.

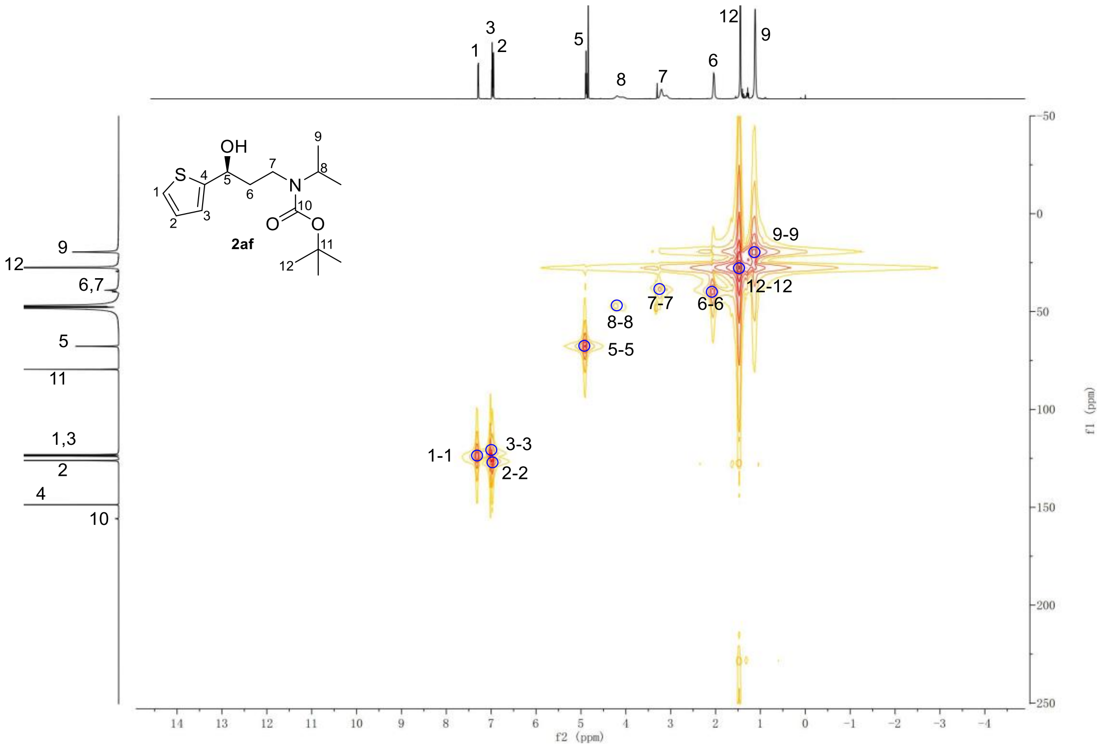

Supplementary Figure 191. NMR analysis for **2af**

4a: The splitting of three carbon peaks of compound **4a** was found in ^{13}C NMR spectra, which may be caused by the steric effect of the large sterically hindered alkoxycarbonyl group. The NMR heating experiment was carried out to confirm the structure.

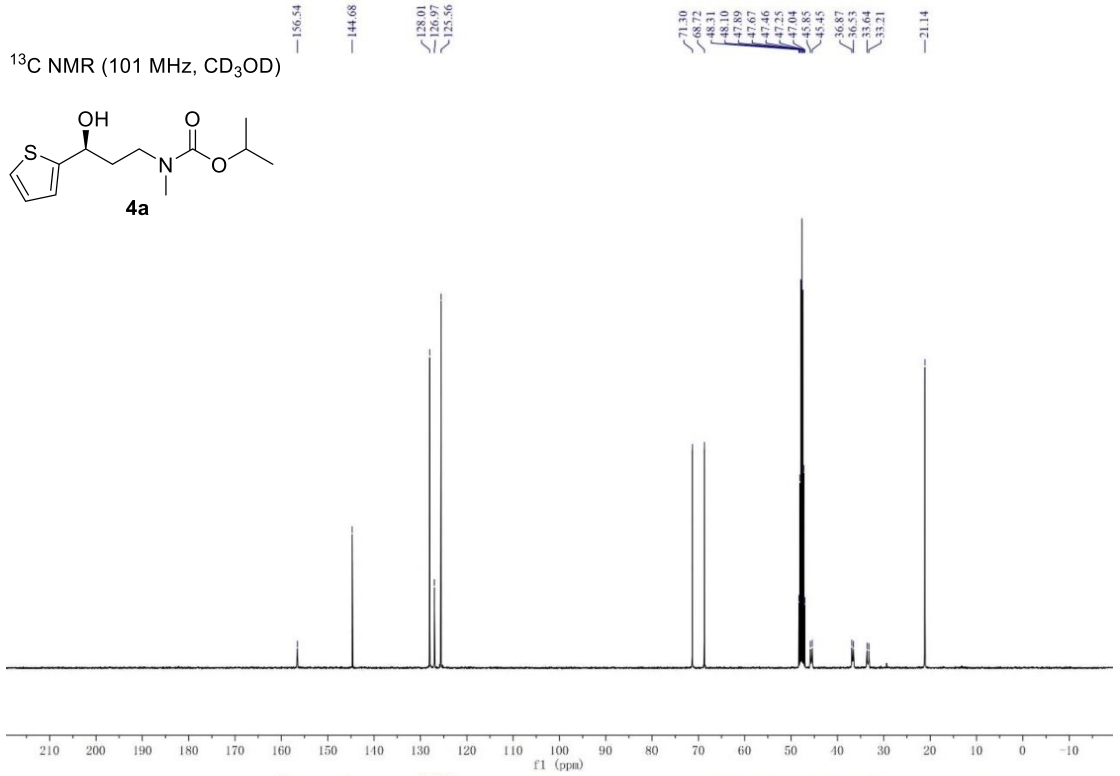

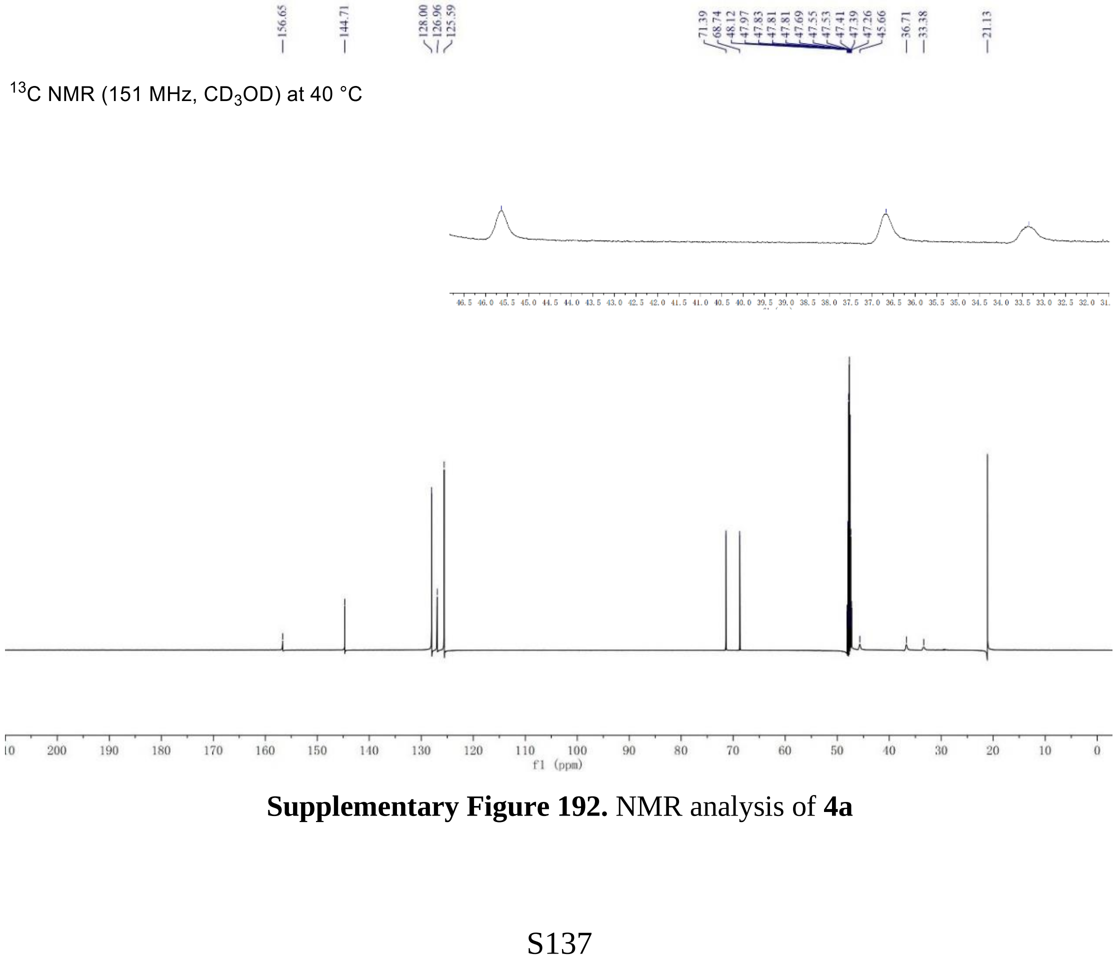

Supplementary Figure 192. NMR analysis of **4a**

2.5. HPLC spectra

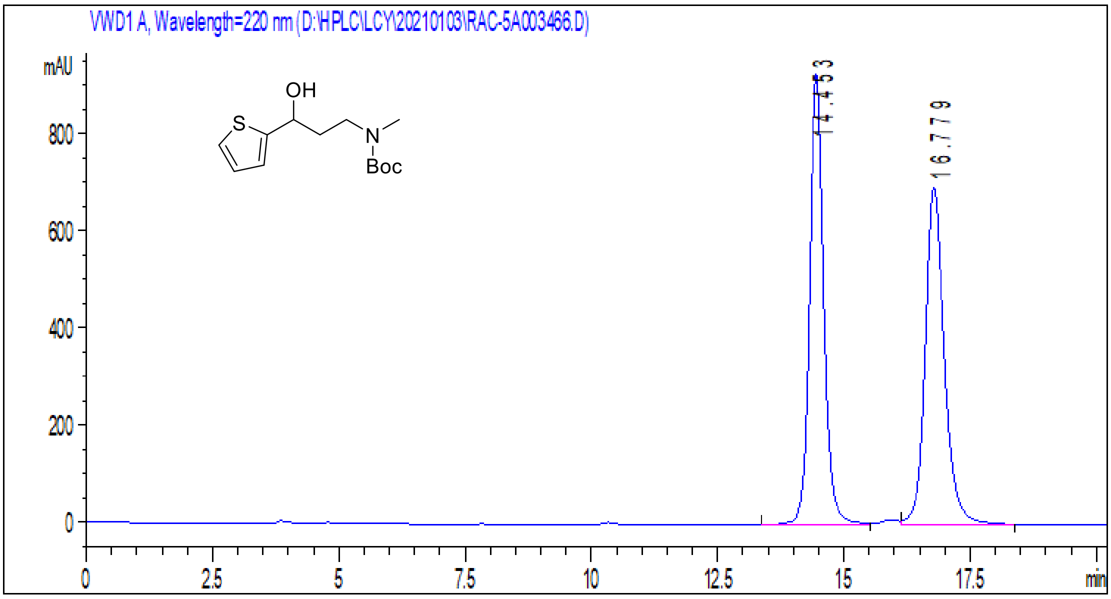

#	Time	Area	Height	Width	Symmetry	Area %
1	14.453	18066.5	927.2	0.2944	0.853	49.696
2	16.779	18287.8	693.9	0.4393	0.787	50.304

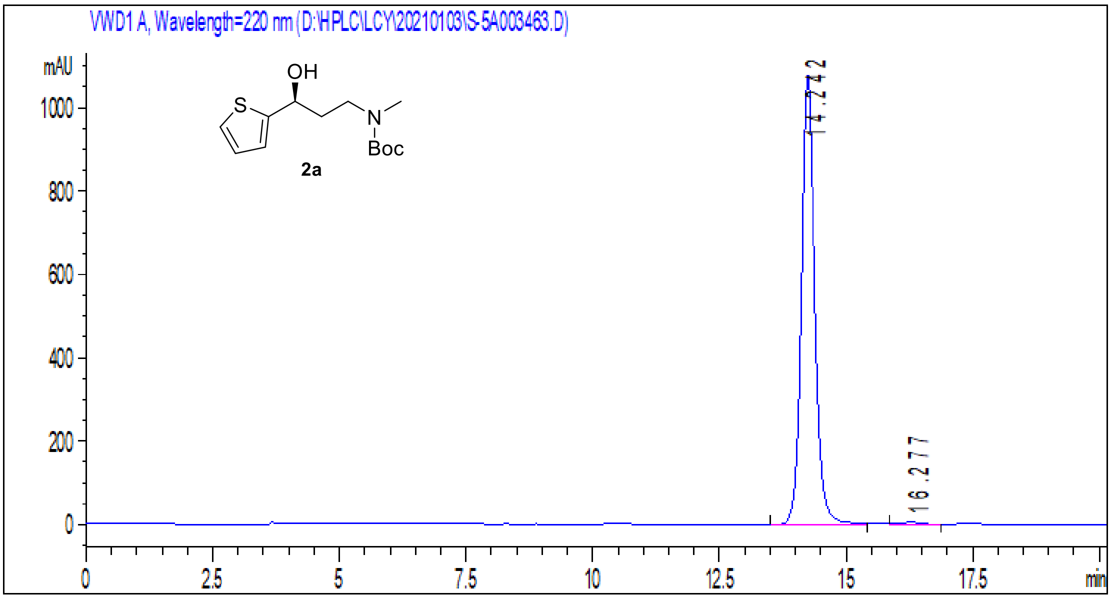

#	Time	Area	Height	Width	Symmetry	Area %
1	14.242	19637.1	1077.4	0.2768	0.849	99.459
2	16.277	106.9	4.1	0.3835	1.149	0.541

Supplementary Figure 193. HPLC spectra of **2a**

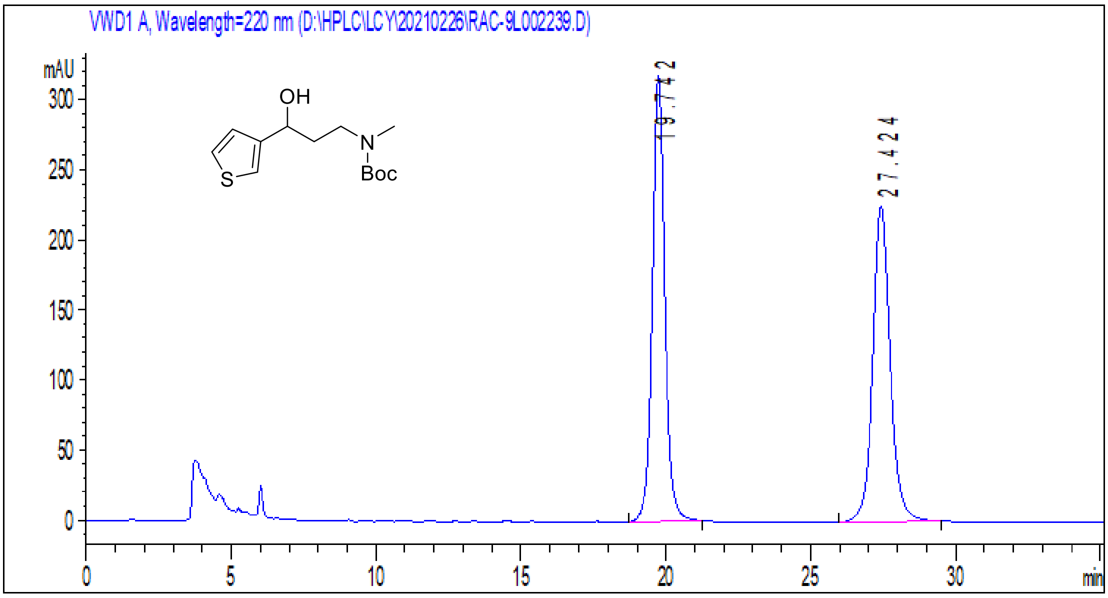

#	Time	Area	Height	Width	Symmetry	Area %
1	19.742	9401.5	318.1	0.4483	0.893	49.947
2	27.424	9421.4	225.2	0.628	0.846	50.053

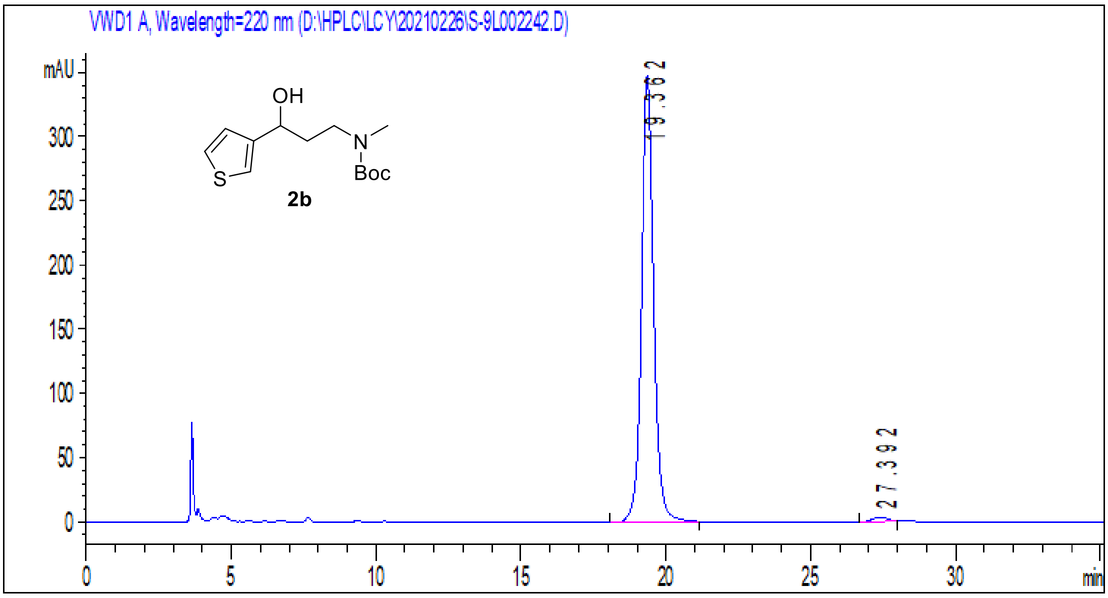

#	Time	Area	Height	Width	Symmetry	Area %
1	19.362	10130.7	347.9	0.4403	0.881	98.873
2	27.392	115.5	3.3	0.4272	1.006	1.127

Supplementary Figure 194. HPLC spectra of **2b**

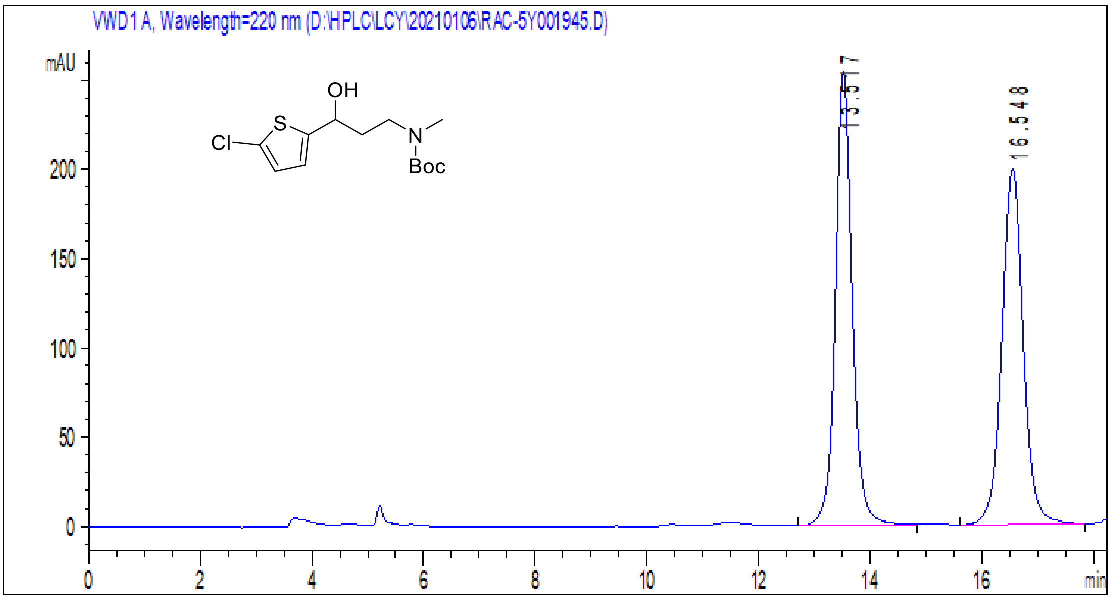

#	Time	Area	Height	Width	Symmetry	Area %
1	13.517	5341.9	253.6	0.3216	0.856	50.228
2	16.548	5293.3	199.1	0.4092	0.953	49.772

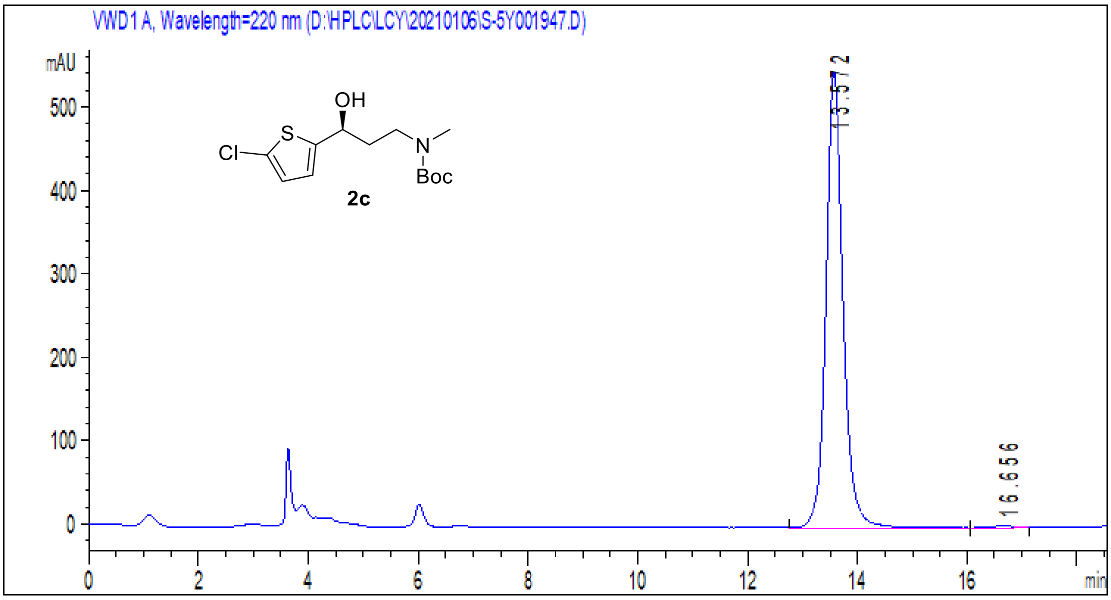

#	Time	Area	Height	Width	Symmetry	Area %
1	13.572	11837.8	545.9	0.3288	0.815	99.409
2	16.656	70.4	2.9	0.3804	0.927	0.591

Supplementary Figure 195. HPLC spectra of **2c**

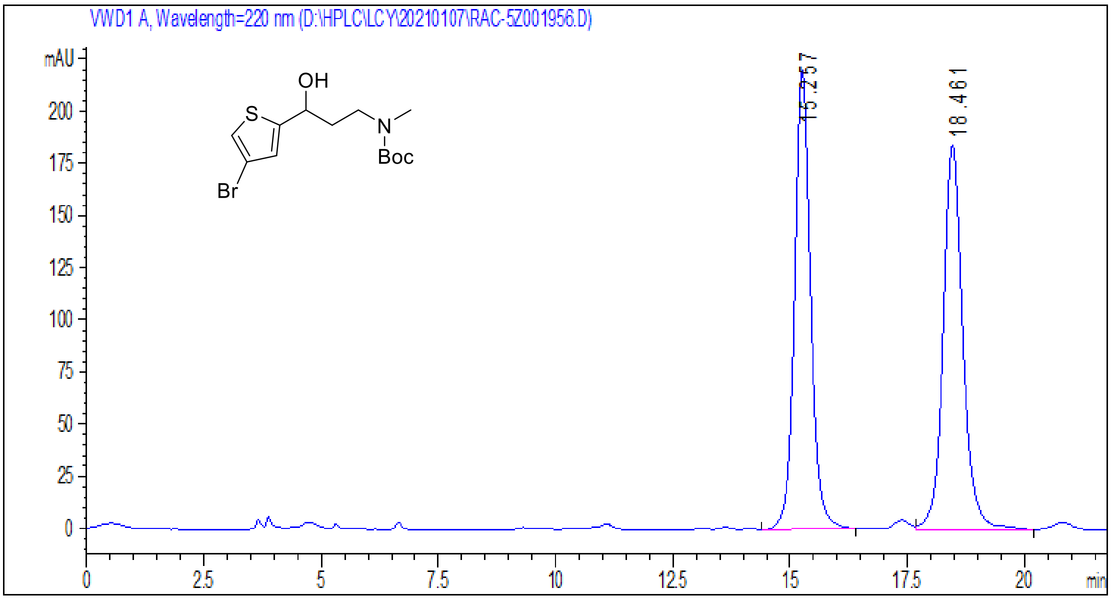

#	Time	Area	Height	Width	Symmetry	Area %
1	15.257	5205.9	220.1	0.3576	0.87	49.184
2	18.461	5378.7	184.3	0.4397	0.844	50.816

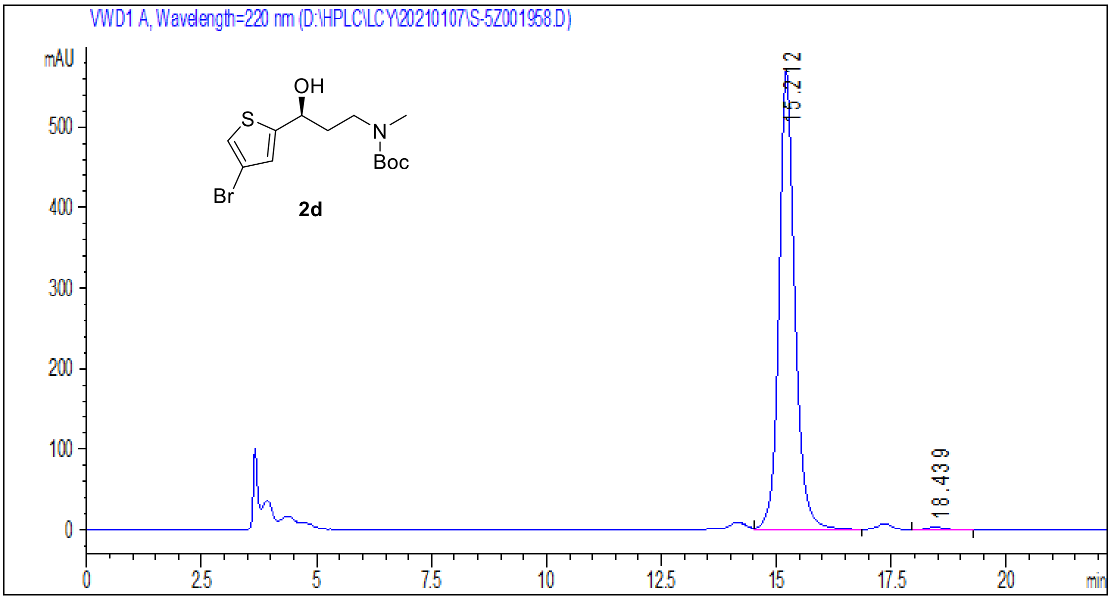

#	Time	Area	Height	Width	Symmetry	Area %
1	15.212	13847.2	570.6	0.3677	0.804	99.280
2	18.439	100.4	3.4	0.4188	0.727	0.720

Supplementary Figure 196. HPLC spectra of **2d**

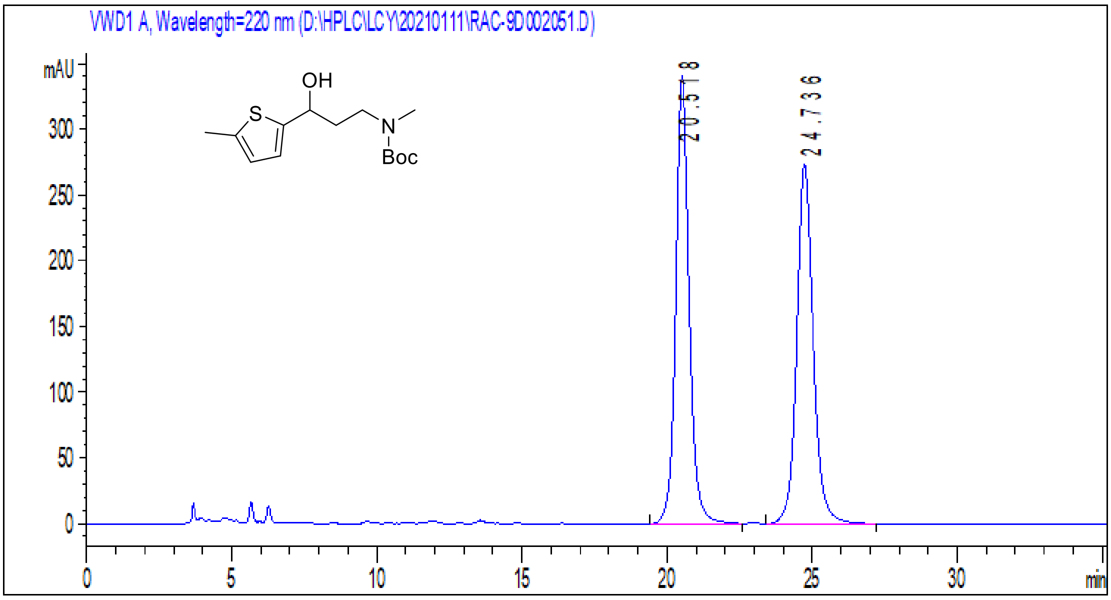

#	Time	Area	Height	Width	Symmetry	Area %
1	20.518	10985.2	340.7	0.4884	0.832	50.520
2	24.736	10759.2	273.1	0.5952	0.823	49.480

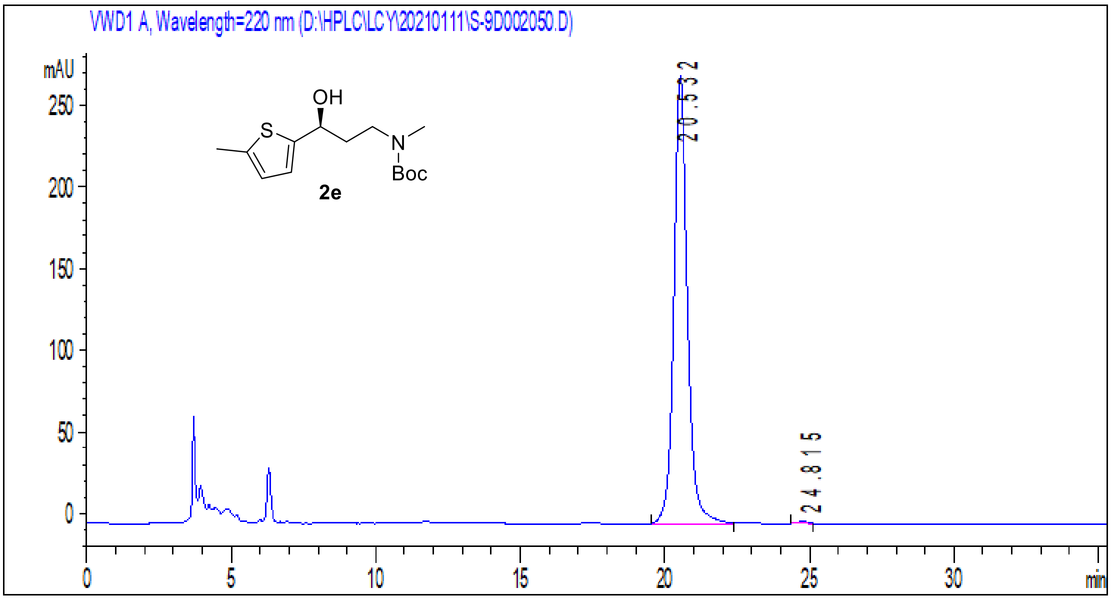

#	Time	Area	Height	Width	Symmetry	Area %
1	20.532	8944.3	274.2	0.4914	0.824	99.726
2	24.815	24.5	9.9E-1	0.4112	1.127	0.274

Supplementary Figure 197. HPLC spectra of **2e**

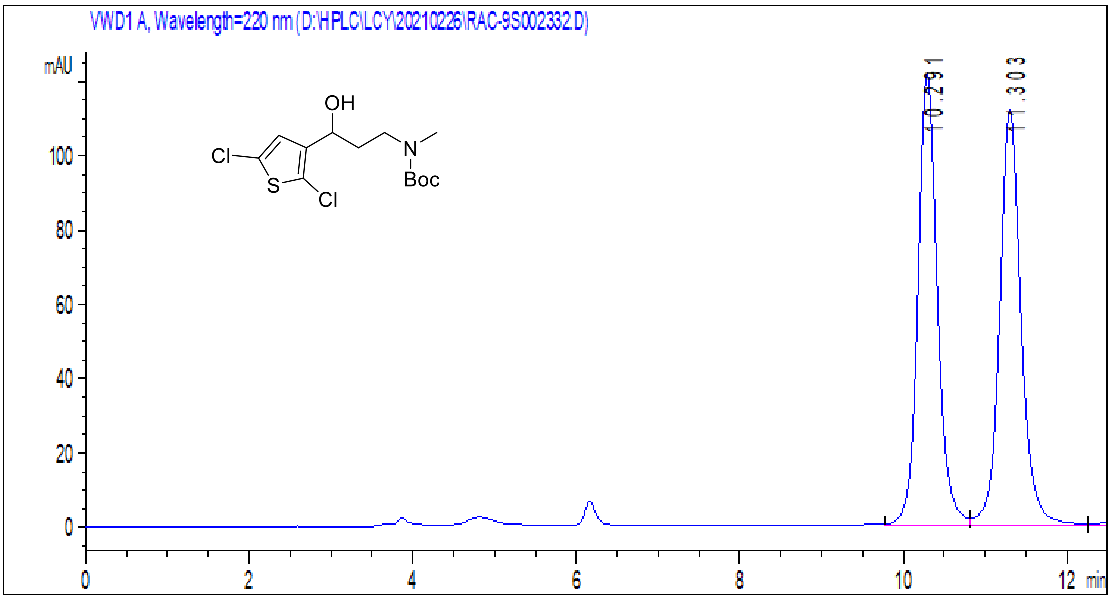

#	Time	Area	Height	Width	Symmetry	Area %
1	10.291	2010.7	121.7	0.2511	0.854	49.677
2	11.303	2036.8	111.6	0.2741	0.853	50.323

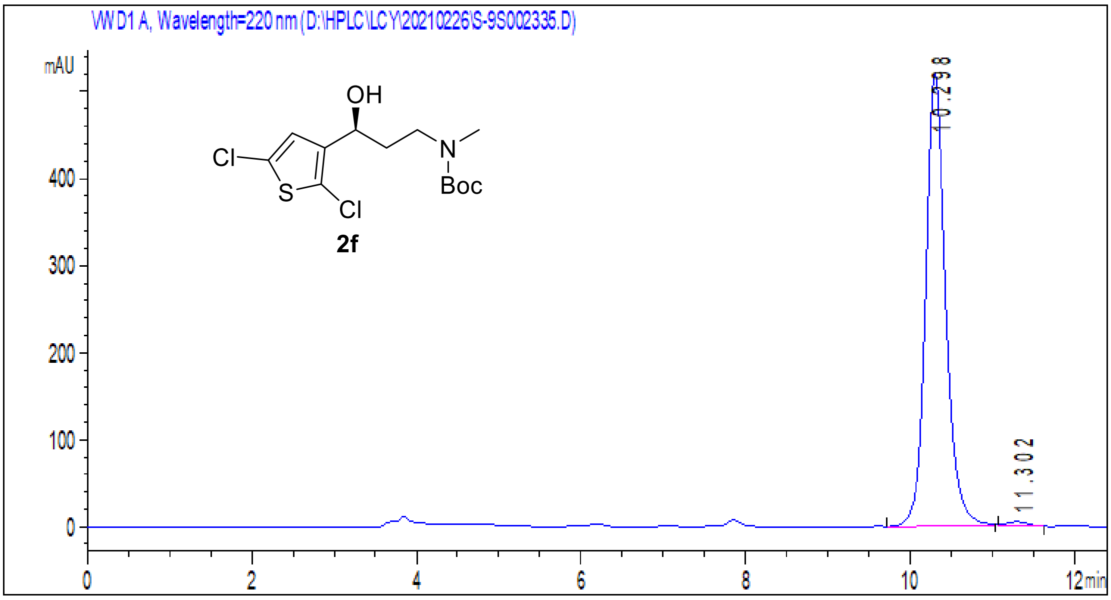

#	Time	Area	Height	Width	Symmetry	Area %
1	10.298	8768.5	521	0.2805	0.818	99.202
2	11.302	70.6	4.4	0.2676	0.845	0.798

Supplementary Figure 198. HPLC spectra of **2f**

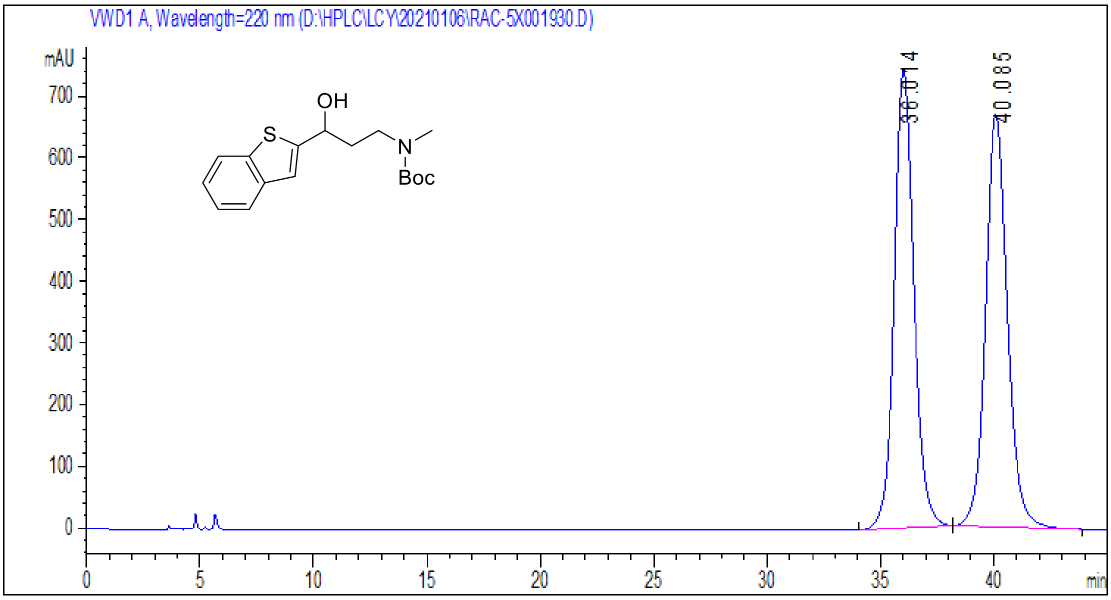

#	Time	Area	Height	Width	Symmetry	Area %
1	36.014	44511.4	742.9	0.909	0.853	49.938
2	40.085	44622.5	669.1	1.0011	0.847	50.062

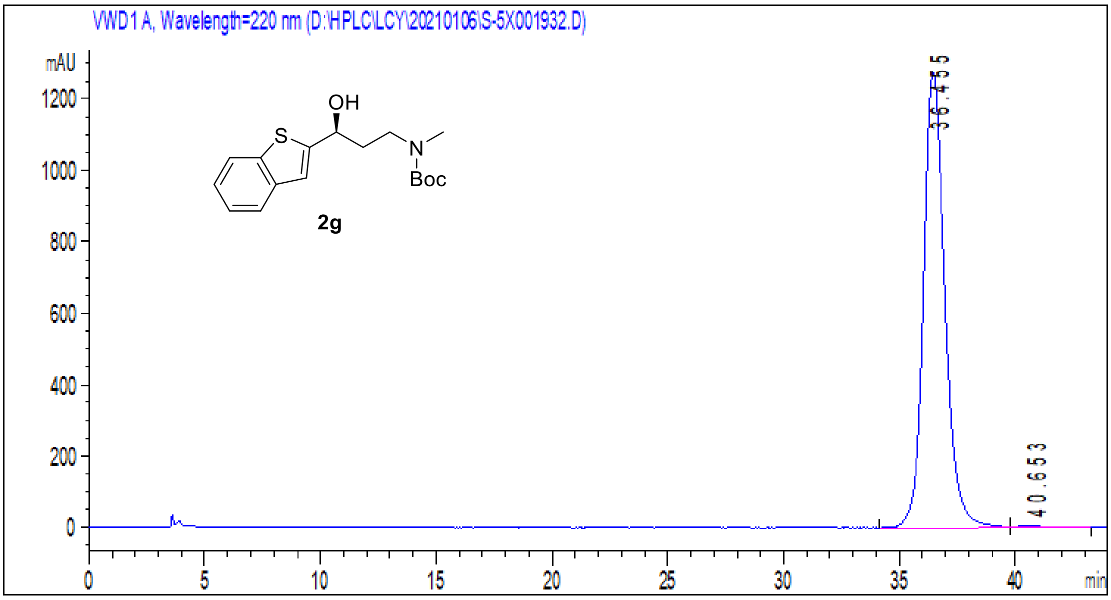

#	Time	Area	Height	Width	Symmetry	Area %
1	36.455	82025.2	1275.7	0.9834	0.765	99.375
2	40.653	515.9	6.8	0.9637	0.978	0.625

Supplementary Figure 199. HPLC spectra of **2g**

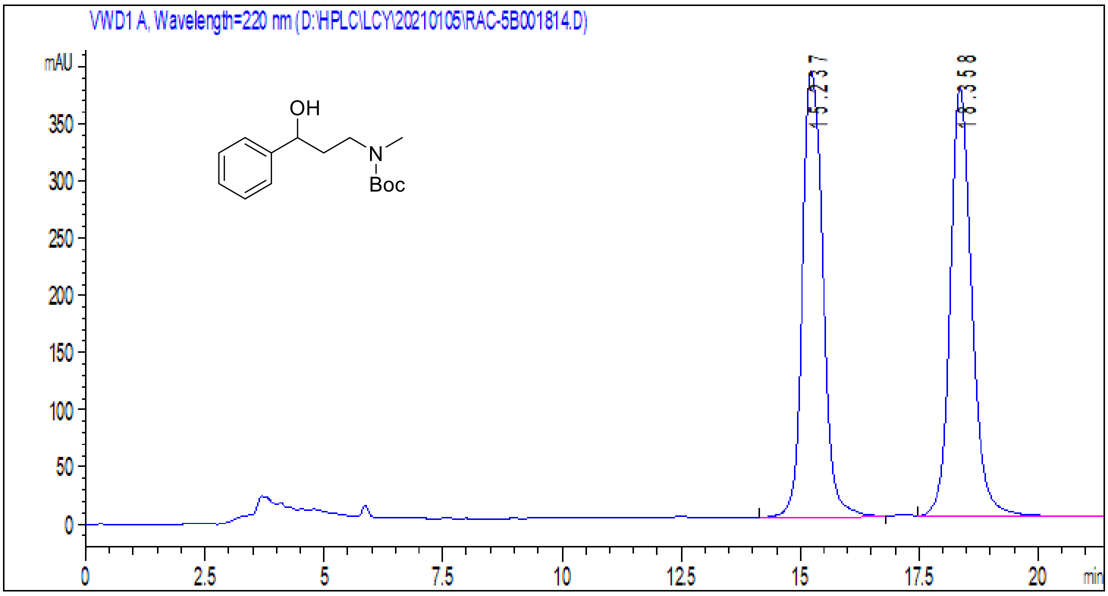

#	Time	Area	Height	Width	Symmetry	Area %
1	15.237	11740	389.2	0.4802	0.74	49.629
2	18.358	11915.6	376.3	0.4861	0.779	50.371

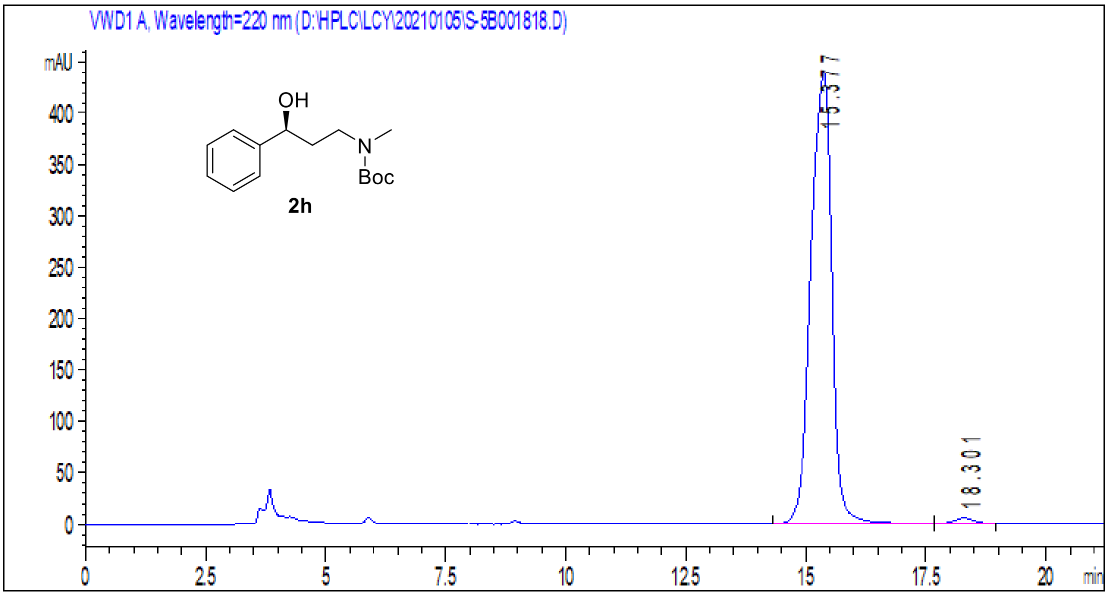

#	Time	Area	Height	Width	Symmetry	Area %
1	15.377	13395.7	439.8	0.4982	1.469	98.901
2	18.301	148.9	5.7	0.3701	0.949	1.099

Supplementary Figure 200. HPLC spectra of **2h**

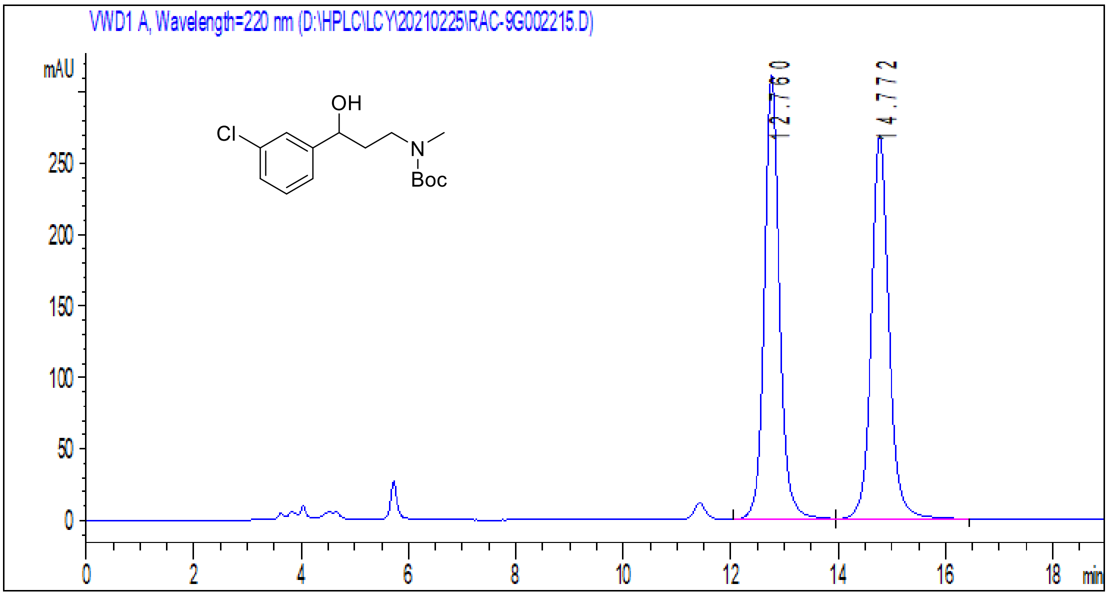

#	Time	Area	Height	Width	Symmetry	Area %
1	12.76	6072.6	310.9	0.2949	0.865	49.927
2	14.772	6090.3	268	0.3453	0.857	50.073

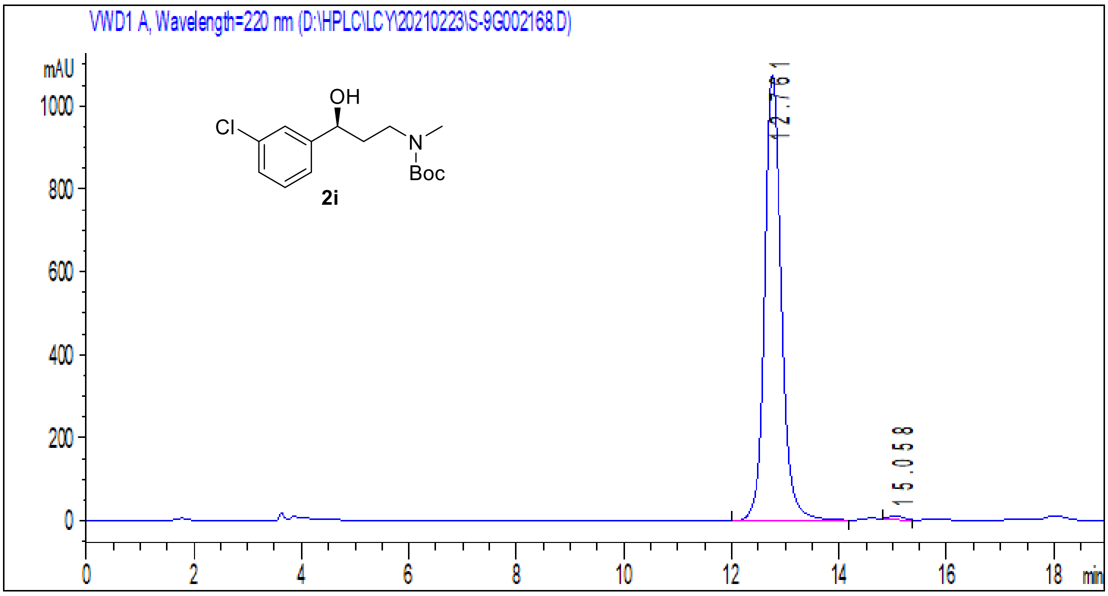

#	Time	Area	Height	Width	Symmetry	Area %
1	12.761	23125.2	1073	0.3316	0.812	99.291
2	15.058	165.1	8.8	0.313	0	0.709

Supplementary Figure 201. HPLC spectra of **2i**

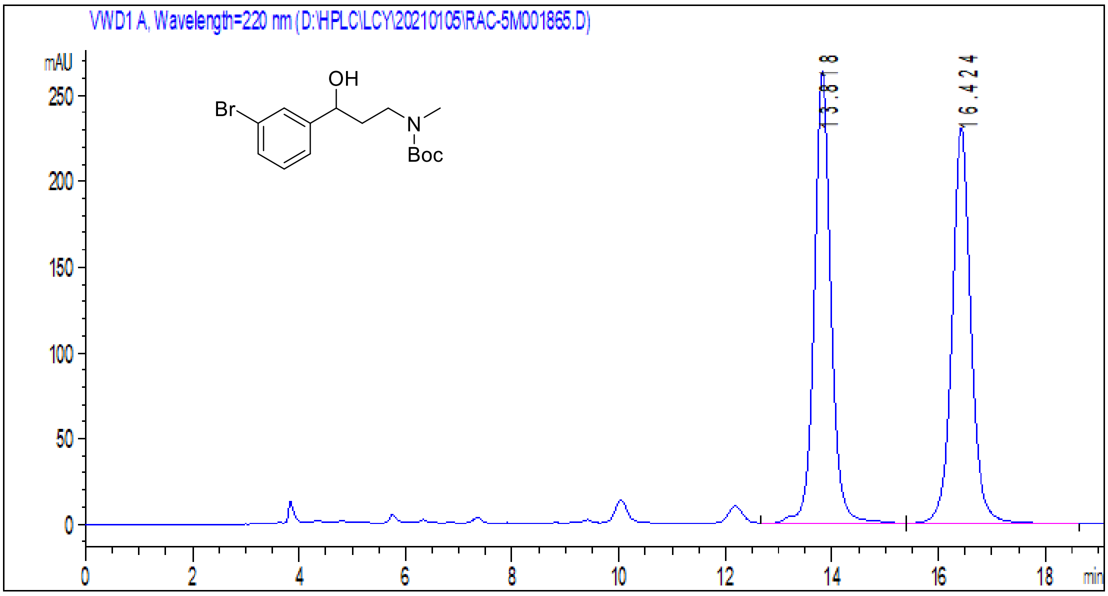

#	Time	Area	Height	Width	Symmetry	Area %
1	13.818	5745.4	263.2	0.3304	0.899	50.143
2	16.424	5712.6	231	0.3731	0.908	49.857

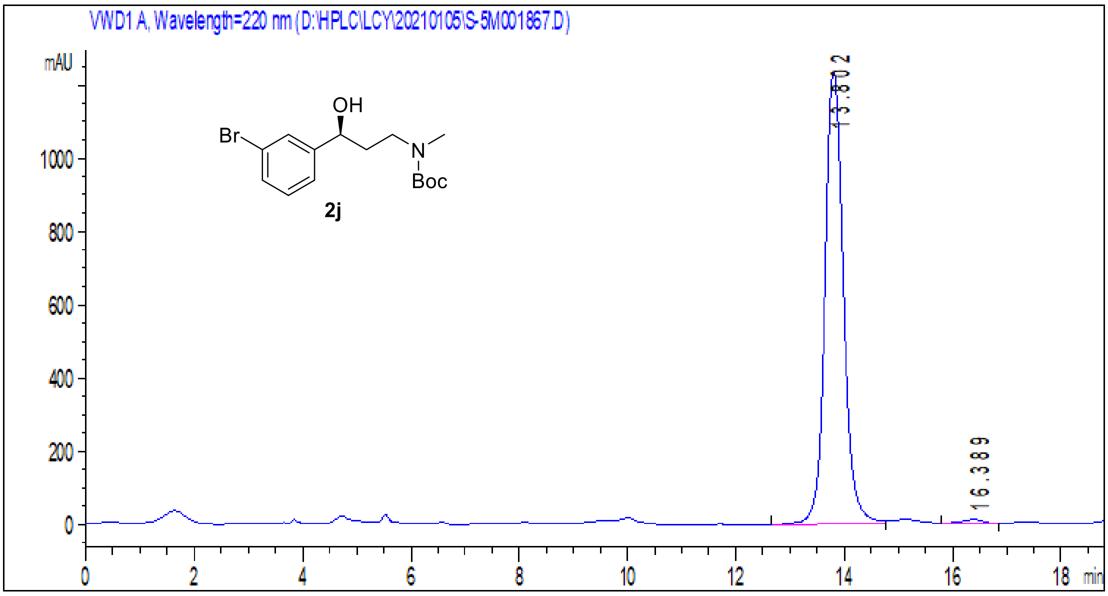

#	Time	Area	Height	Width	Symmetry	Area %
1	13.802	29152.7	1236	0.3626	0.83	98.835
2	16.389	343.5	12.1	0.42	1.502	1.165

Supplementary Figure 202. HPLC spectra of **2j**

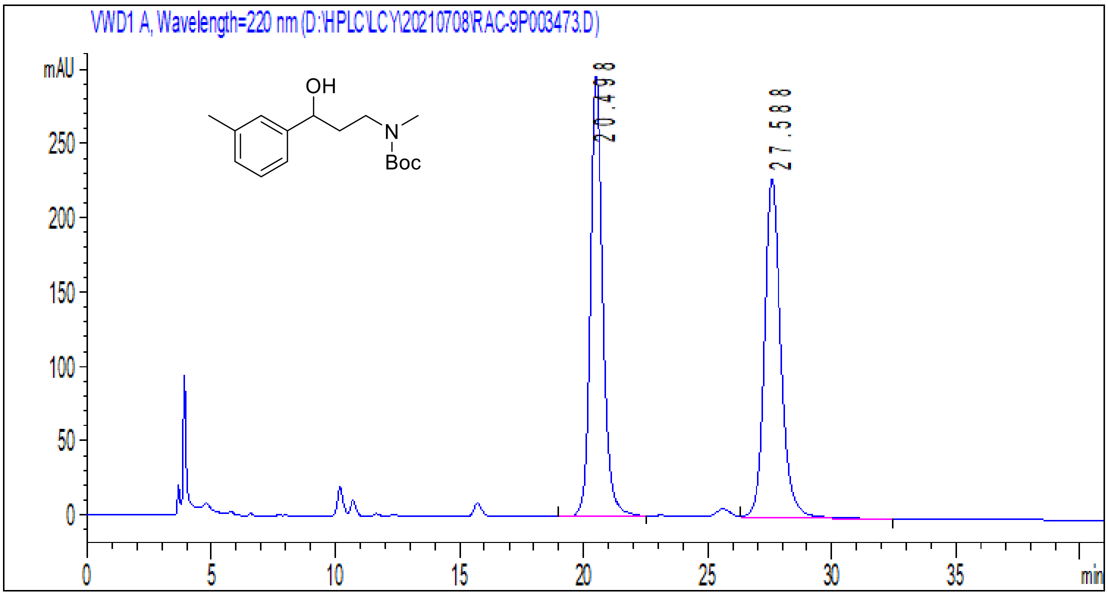

#	Time	Area	Height	Width	Symmetry	Area %
1	20.498	10346.1	296.5	0.5296	0.77	49.848
2	27.588	10409.3	228.2	0.6896	0.789	50.152

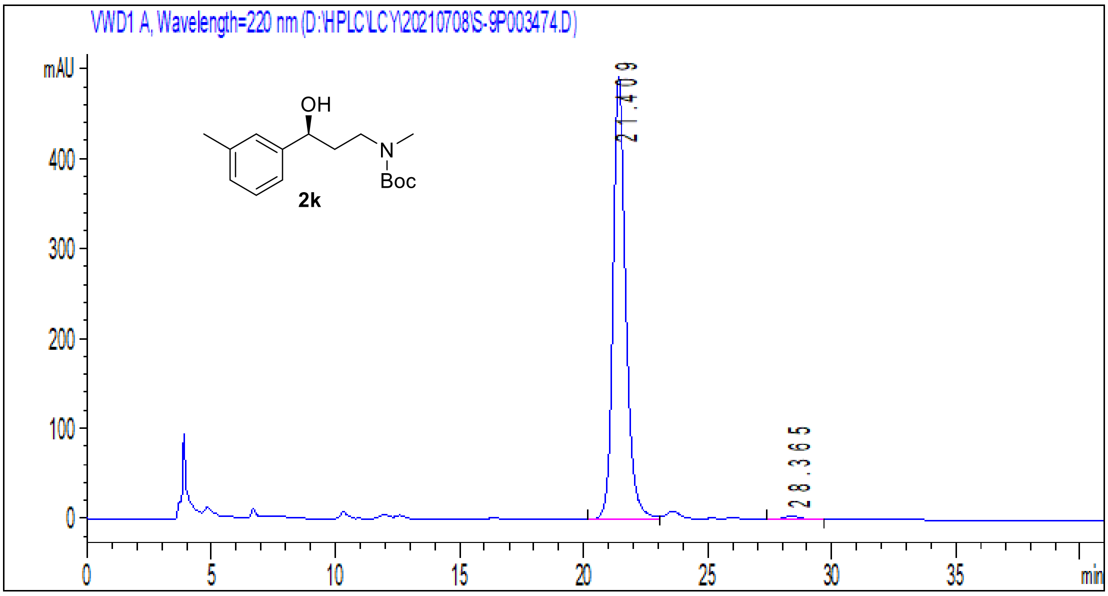

#	Time	Area	Height	Width	Symmetry	Area %
1	21.409	17633.2	492.2	0.5462	0.733	99.269
2	28.365	129.8	3	0.5743	0.839	0.731

Supplementary Figure 203. HPLC spectra of **2k**

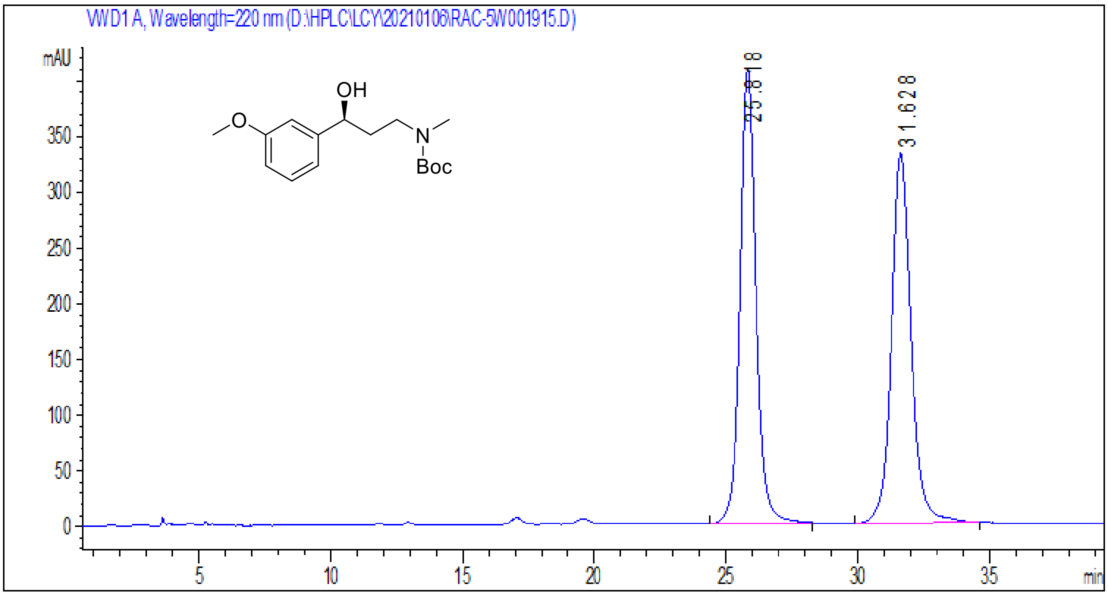

#	Time	Area	Height	Width	Symmetry	Area %
1	25.818	17081.5	407.5	0.6348	0.861	49.952
2	31.628	17114.6	331.5	0.7759	0.842	50.048

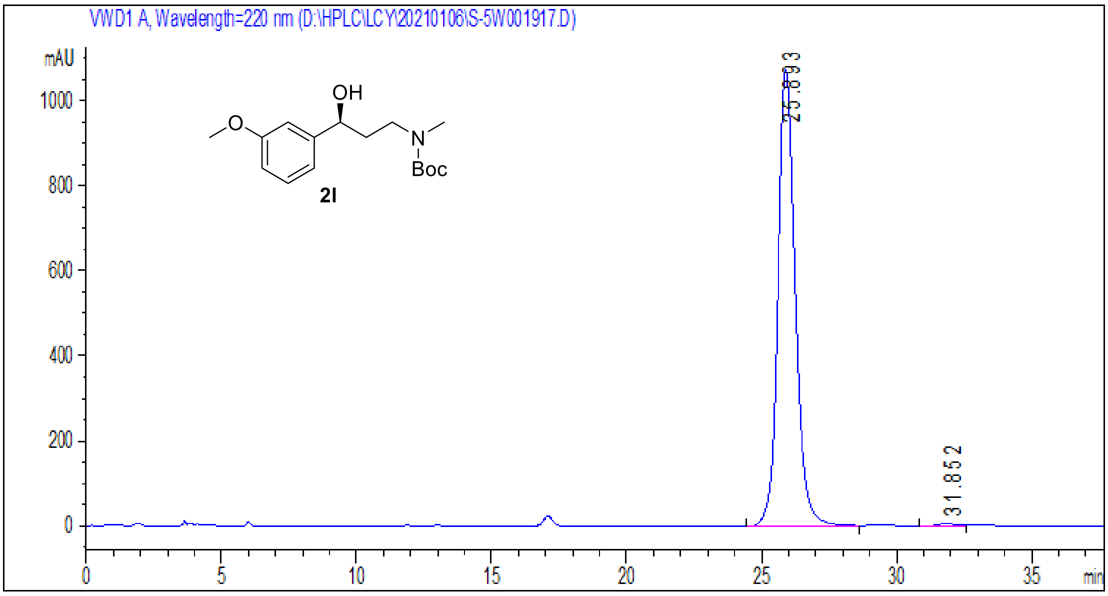

#	Time	Area	Height	Width	Symmetry	Area %
1	25.893	47704.7	1074.1	0.68	0.782	99.291
2	31.852	340.8	7	0.5877	0.921	0.709

Supplementary Figure 204. HPLC spectra of **2l**

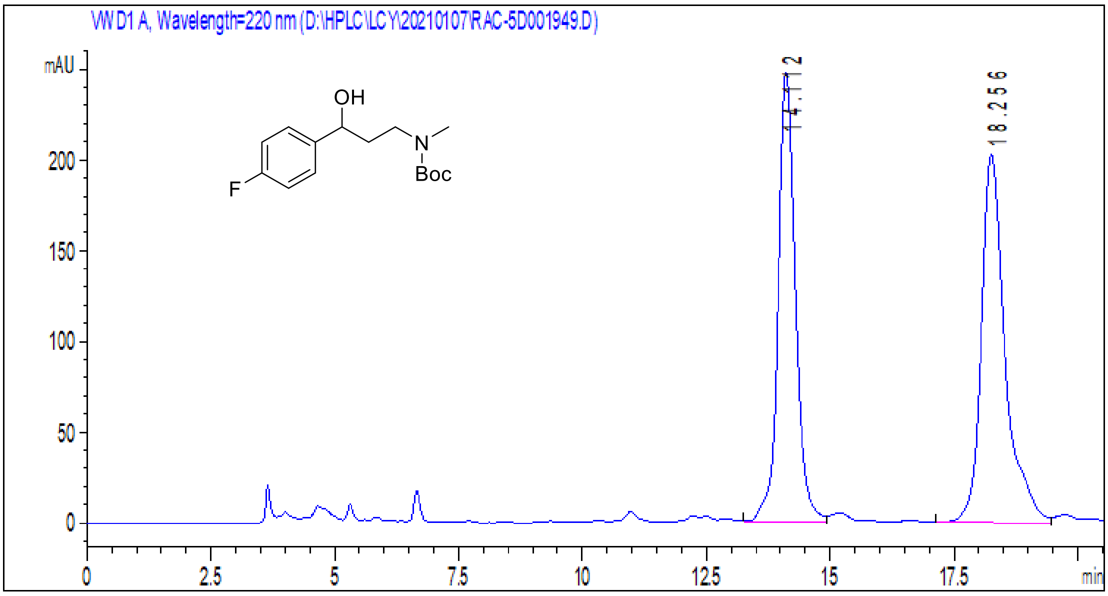

#	Time	Area	Height	Width	Symmetry	Area %
1	14.112	5980.3	247.5	0.3693	0.825	46.839
2	18.256	6787.5	202.7	0.5014	0.682	53.161

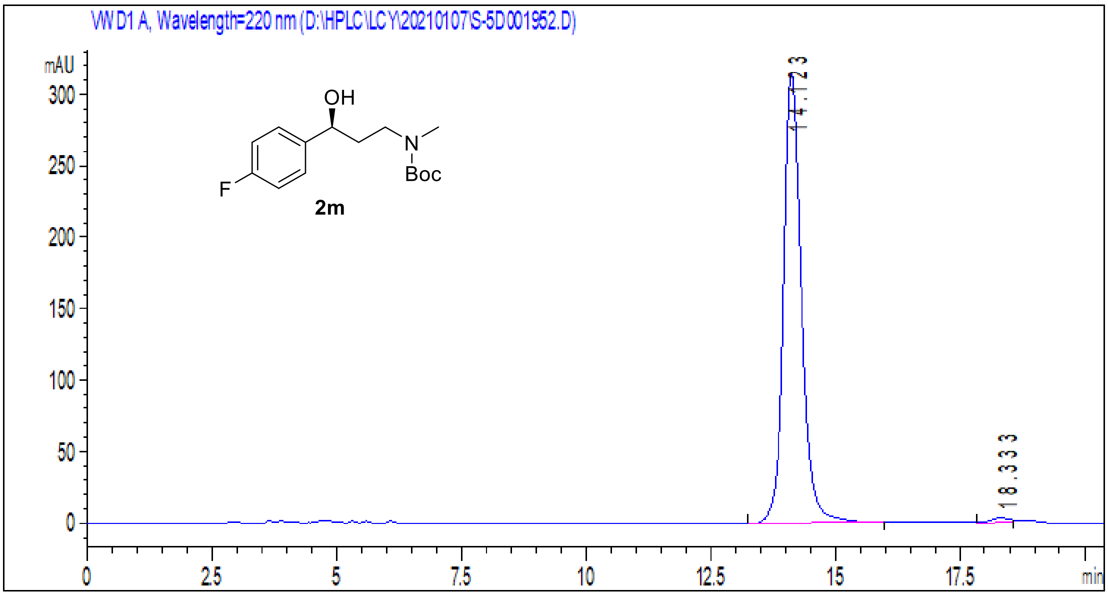

#	Time	Area	Height	Width	Symmetry	Area %
1	14.123	7841.9	315.4	0.3805	0.743	98.827
2	18.333	93.1	3.5	0.4385	1.173	1.173

Supplementary Figure 205. HPLC spectra of **2m**

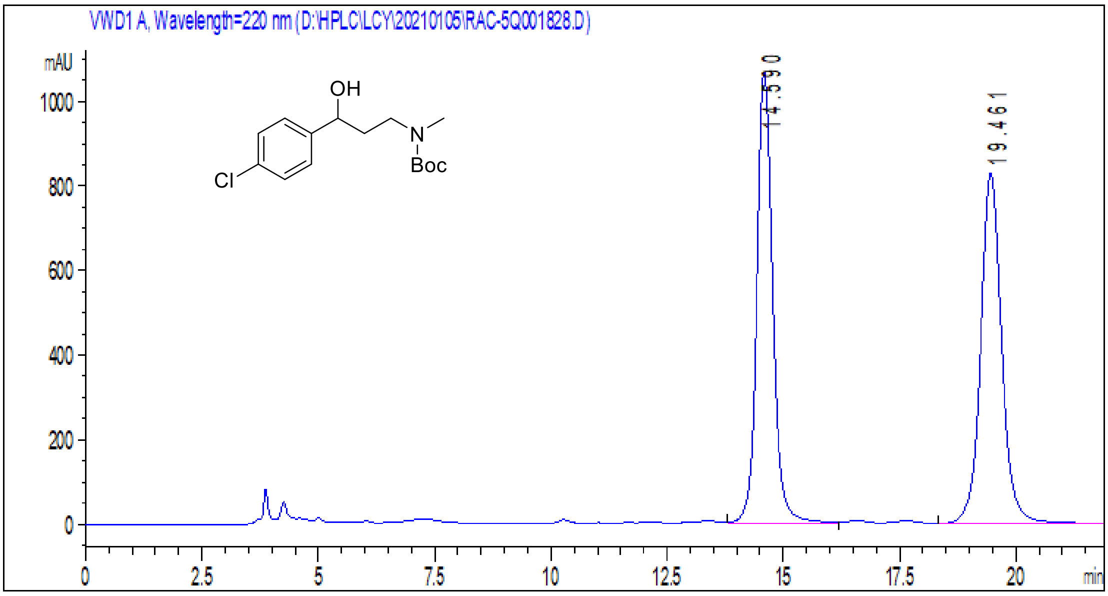

#	Time	Area	Height	Width	Symmetry	Area %
1	14.59	25409.1	1068.5	0.3649	0.83	49.507
2	19.461	25915.4	828	0.476	0.879	50.493

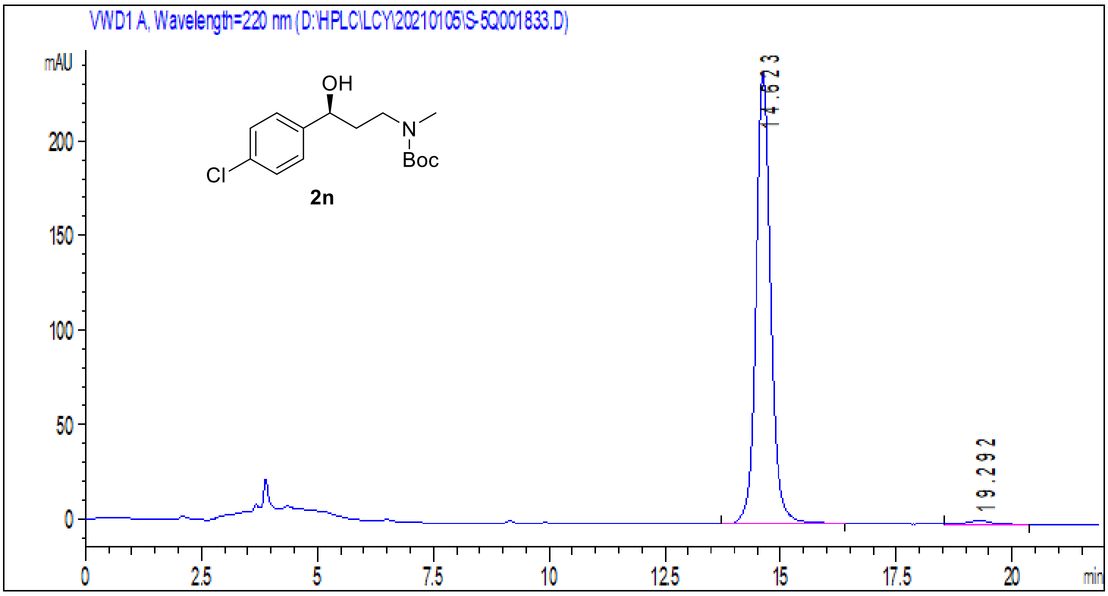

#	Time	Area	Height	Width	Symmetry	Area %
1	14.623	5235.6	238.8	0.3316	0.868	98.731
2	19.292	67.3	2.1	0.48	0.983	1.269

Supplementary Figure 206. HPLC spectra of **2n**

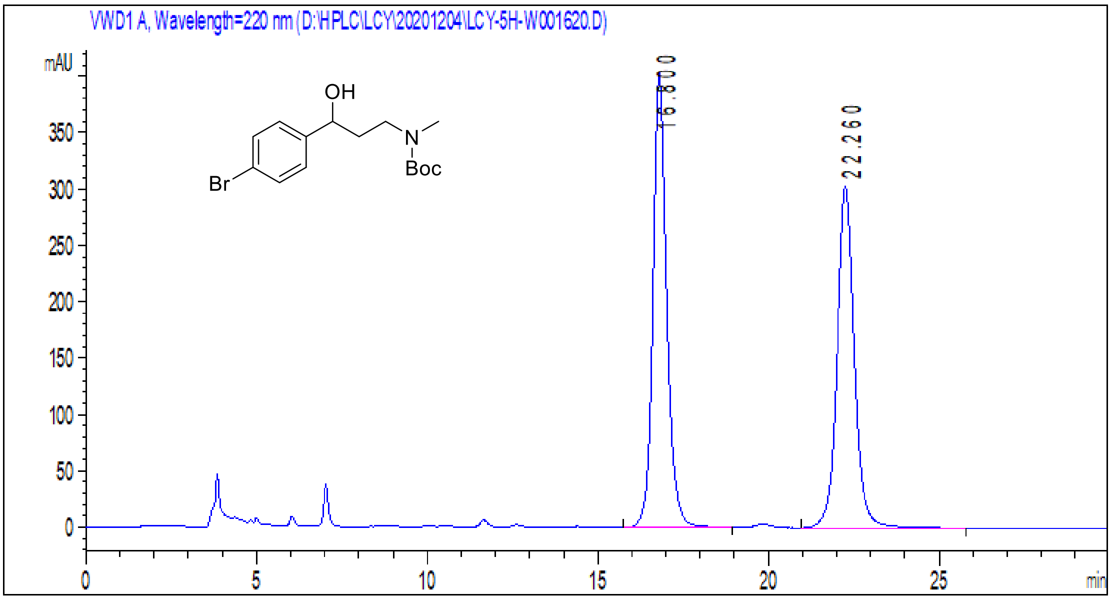

#	Time	Area	Height	Width	Symmetry	Area %
1	16.8	11421.2	403.8	0.4262	0.791	51.918
2	22.26	10577.2	302.2	0.5309	0.893	48.082

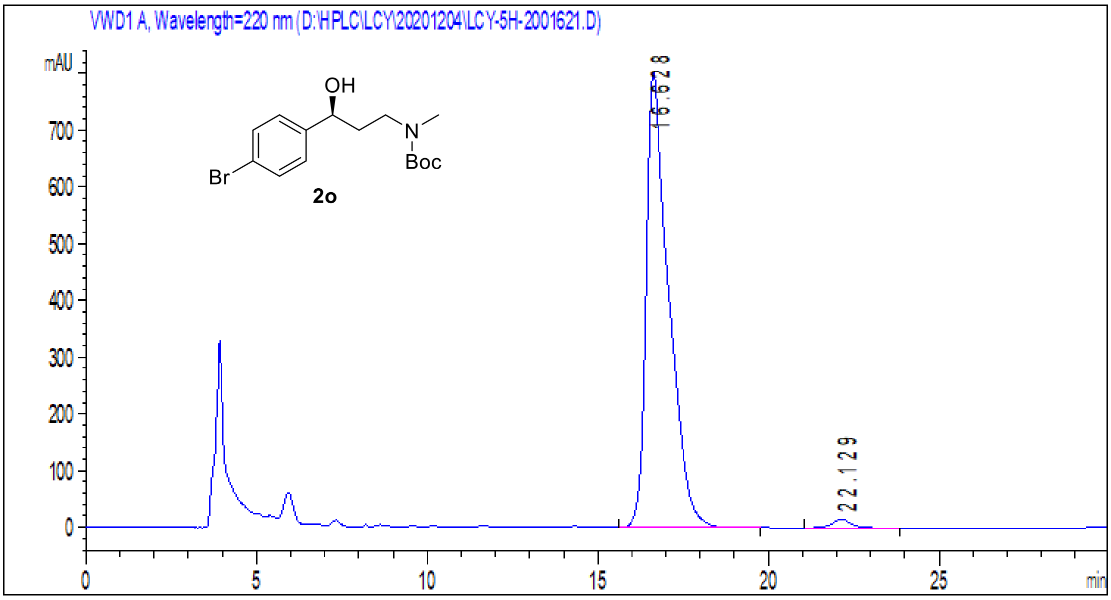

#	Time	Area	Height	Width	Symmetry	Area %
1	16.628	36744.8	802.4	0.6421	0.44	98.514
2	22.129	554.4	15.1	0.558	0.891	1.486

Supplementary Figure 207. HPLC spectra of **2o**

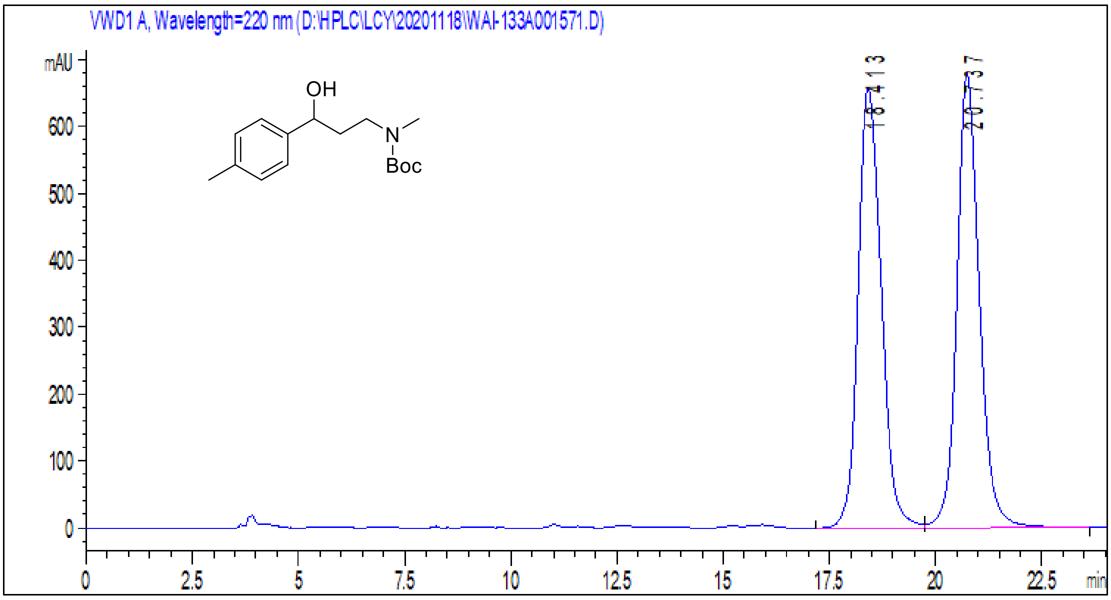

#	Time	Area	Height	Width	Symmetry	Area %
1	18.413	24889.3	657.4	0.5992	0.75	49.959
2	20.737	24930.5	681.3	0.5581	0.777	50.041

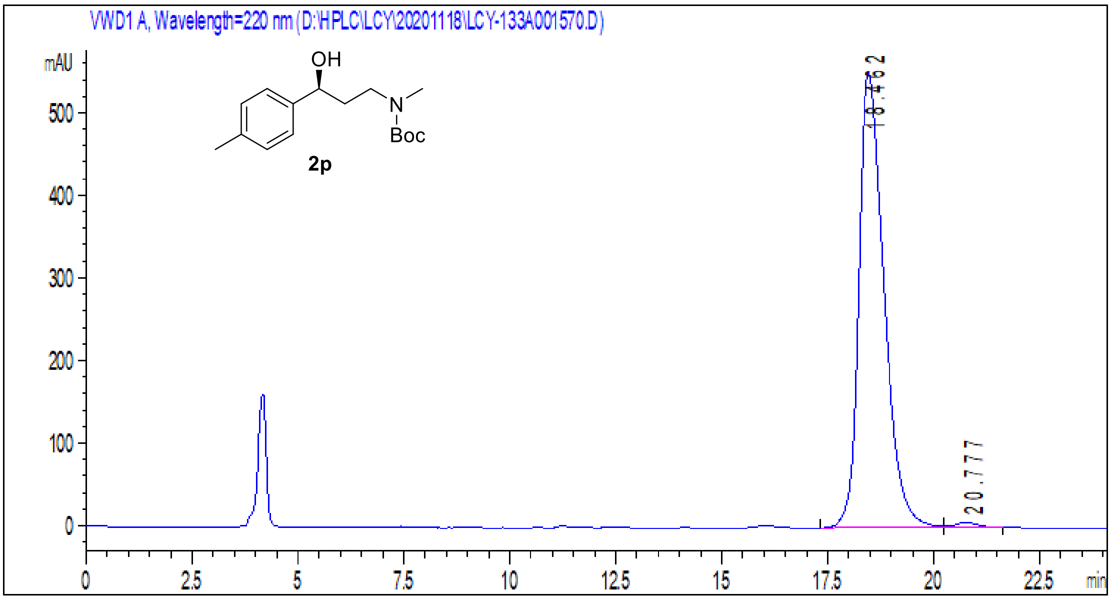

#	Time	Area	Height	Width	Symmetry	Area %
1	18.462	22577.4	551.3	0.6294	0.592	99.244
2	20.777	172	5.5	0.4414	0.759	0.756

Supplementary Figure 208. HPLC spectra of **2p**

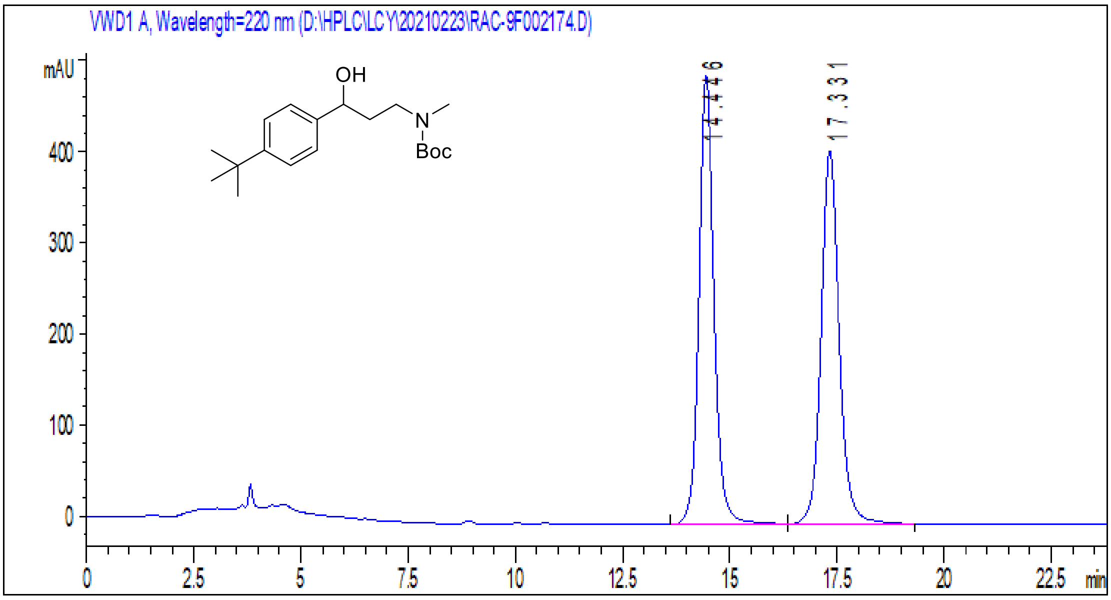

#	Time	Area	Height	Width	Symmetry	Area %
1	14.446	11971.1	491.9	0.3714	0.835	50.058
2	17.331	11943.4	409.4	0.4439	0.854	49.942

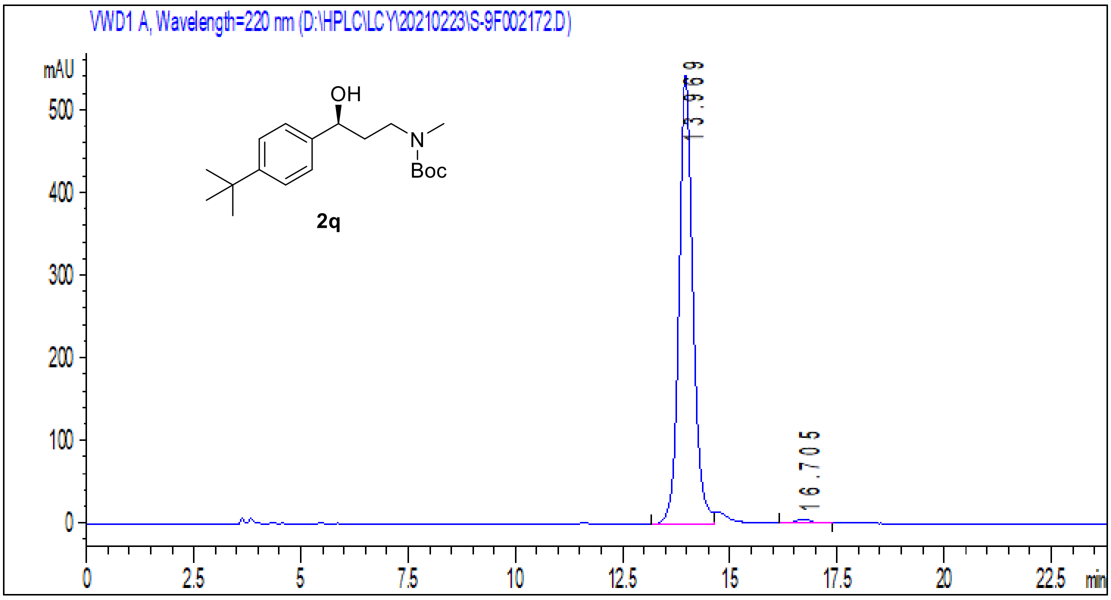

#	Time	Area	Height	Width	Symmetry	Area %
1	13.969	12399.5	541.6	0.3473	0.847	99.088
2	16.705	114.1	4.3	0.3233	0.879	0.912

Supplementary Figure 209. HPLC spectra of **2q**

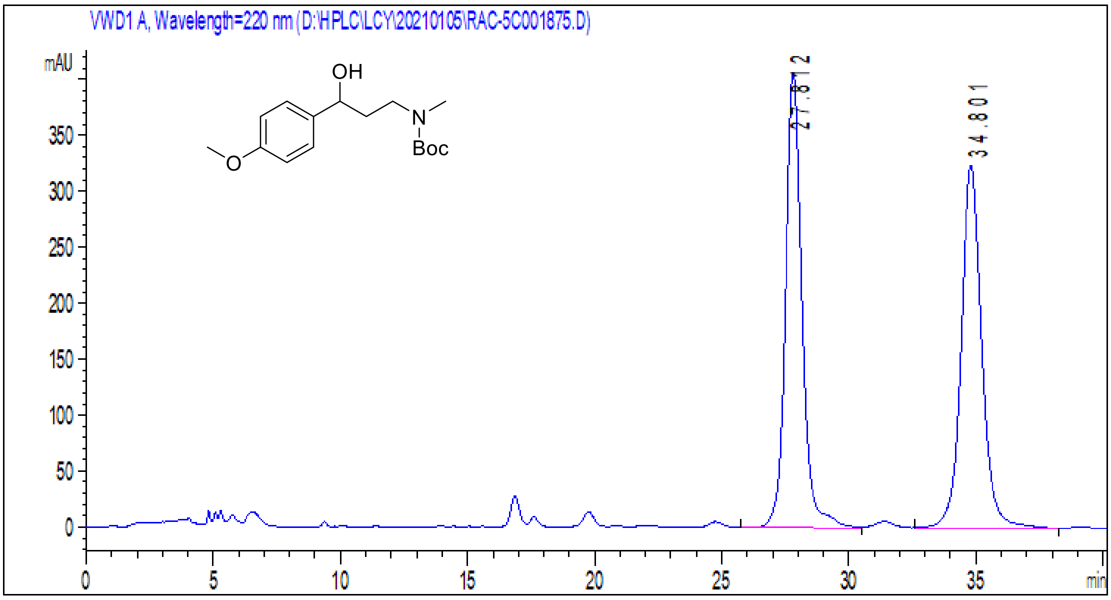

#	Time	Area	Height	Width	Symmetry	Area %
1	27.812	18315.7	406.9	0.6767	0.828	50.204
2	34.801	18167	323.5	0.8417	0.869	49.796

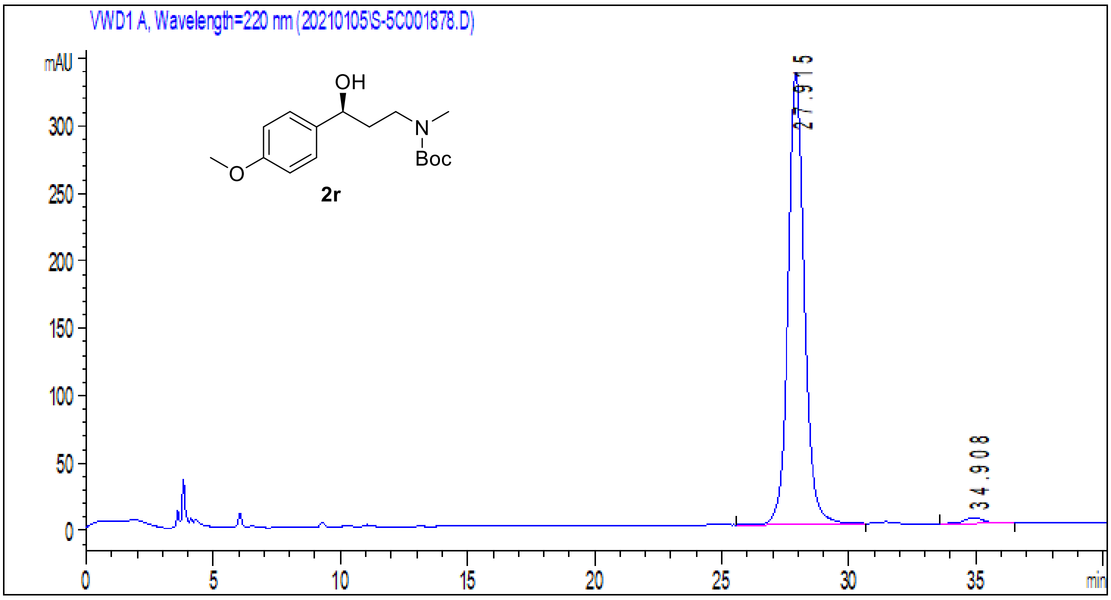

#	Time	Area	Height	Width	Symmetry	Area %
1	27.915	14881	335.3	0.6752	0.859	98.518
2	34.908	223.8	4.2	0.764	0.98	1.482

Supplementary Figure 210. HPLC spectra of **2r**

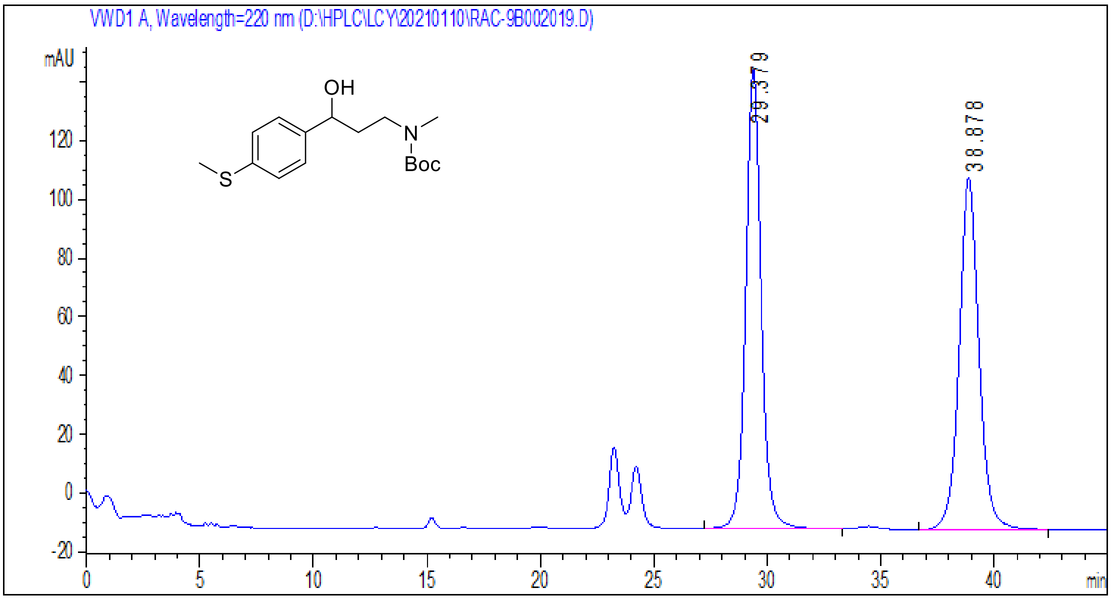

#	Time	Area	Height	Width	Symmetry	Area %
1	29.379	7387.9	156.2	0.7208	0.883	50.292
2	38.878	7302	119.7	0.9157	0.896	49.708

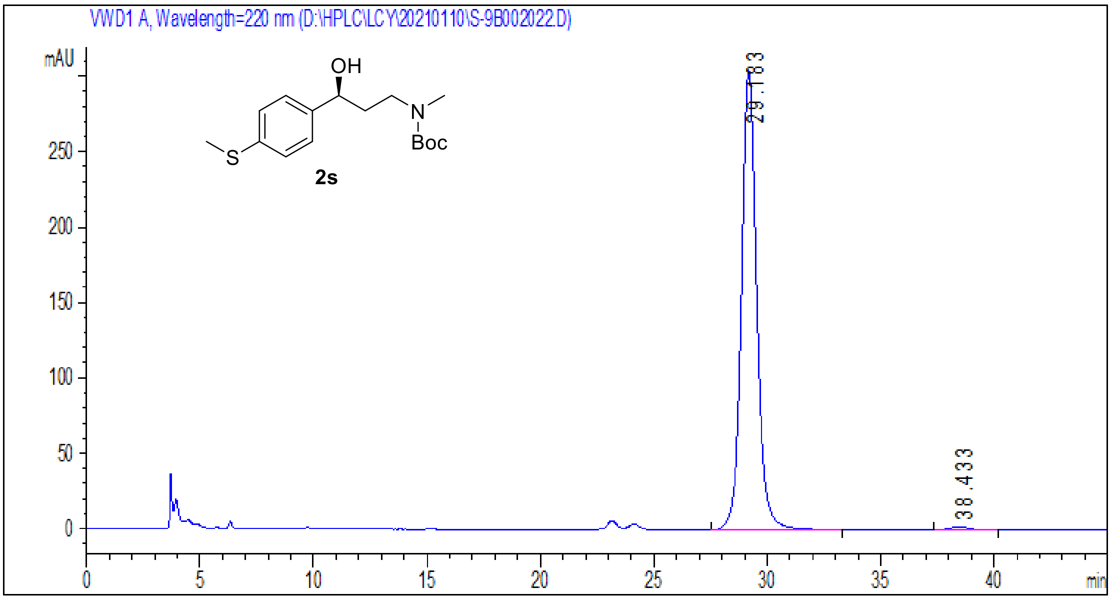

#	Time	Area	Height	Width	Symmetry	Area %
1	29.183	14149.6	304.9	0.7017	0.862	99.031
2	38.433	138.4	2.4	0.705	0.84	0.969

Supplementary Figure 211. HPLC spectra of **2s**

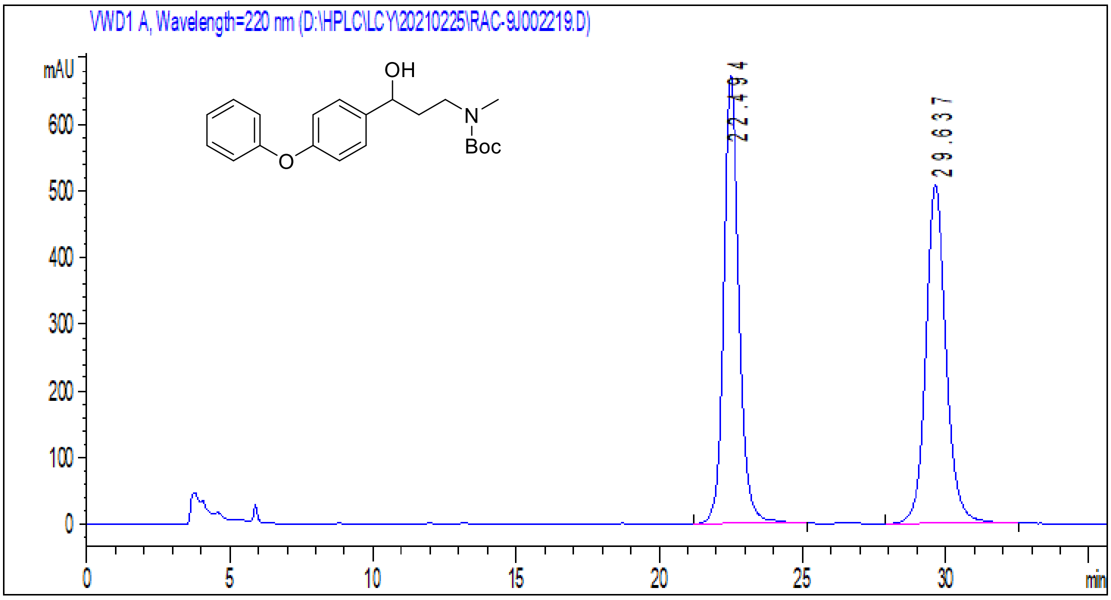

#	Time	Area	Height	Width	Symmetry	Area %
1	22.494	24778.4	671.4	0.5574	0.798	49.987
2	29.637	24791.2	507.1	0.7438	0.831	50.013

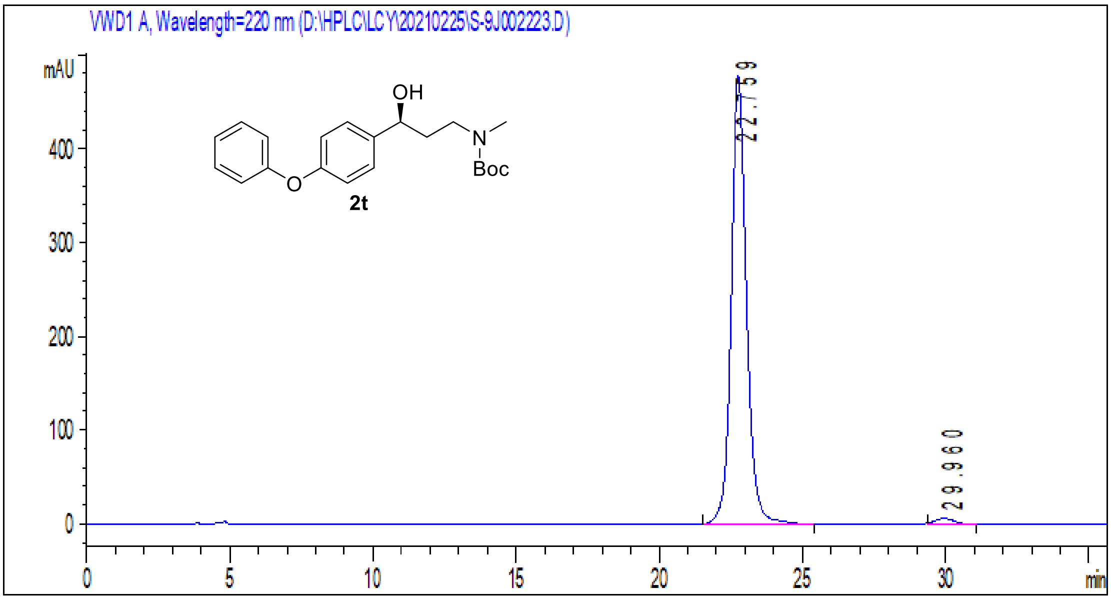

#	Time	Area	Height	Width	Symmetry	Area %
1	22.759	17647.2	477.6	0.5579	0.834	98.397
2	29.96	287.4	6.3	0.7639	0.98	1.603

Supplementary Figure 212. HPLC spectra of **2t**

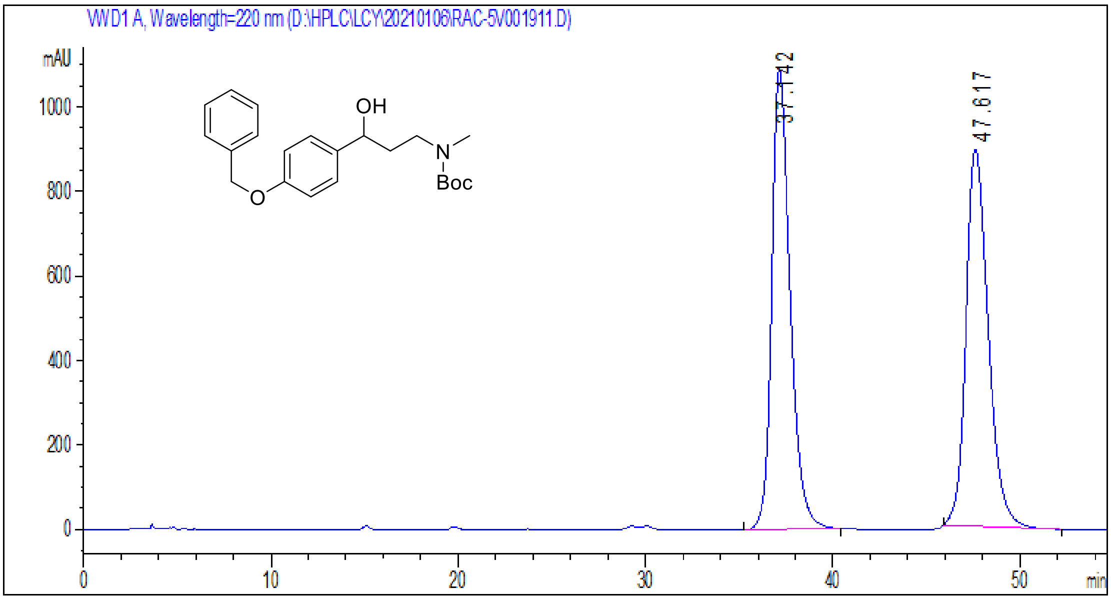

#	Time	Area	Height	Width	Symmetry	Area %
1	37.142	74092.2	1087.7	1.0251	0.65	50.254
2	47.617	73343.5	892.4	1.1848	0.703	49.746

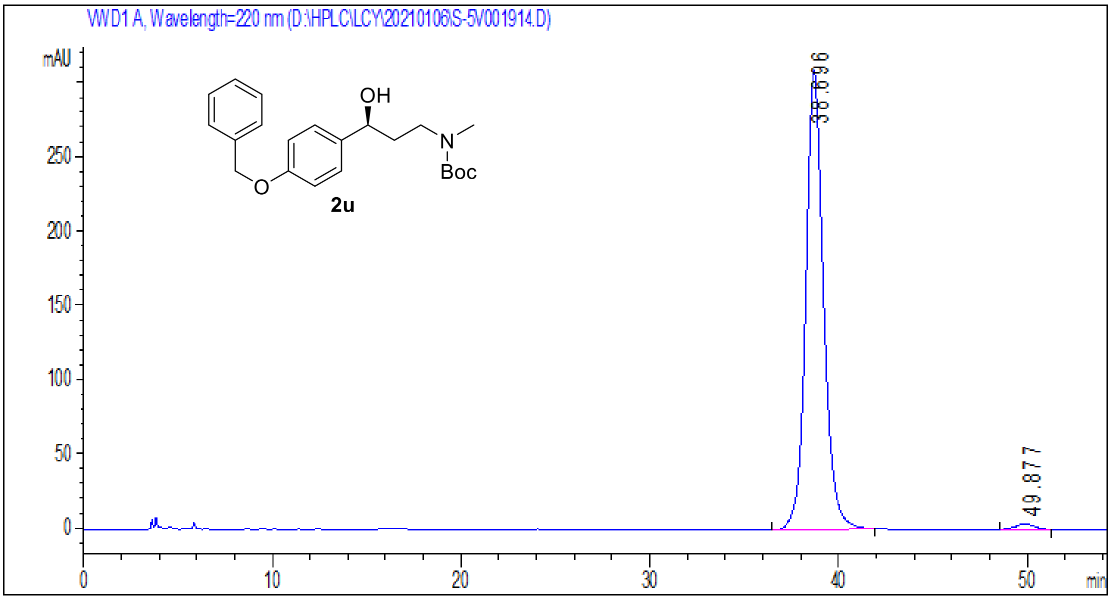

#	Time	Area	Height	Width	Symmetry	Area %
1	38.696	20371.3	309.6	0.9994	0.819	98.725
2	49.877	263.1	3.7	0.8541	0.992	1.275

Supplementary Figure 213. HPLC spectra of **2u**

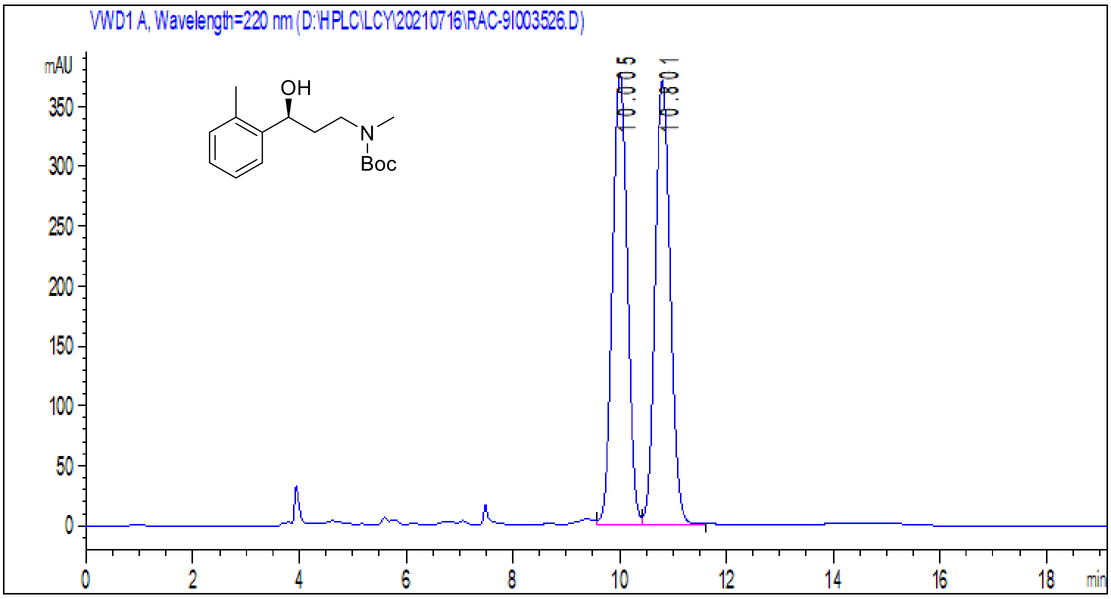

#	Time	Area	Height	Width	Symmetry	Area %
1	10.005	7266	377	0.3036	0.963	49.740
2	10.801	7342.1	370.4	0.3129	0.884	50.260

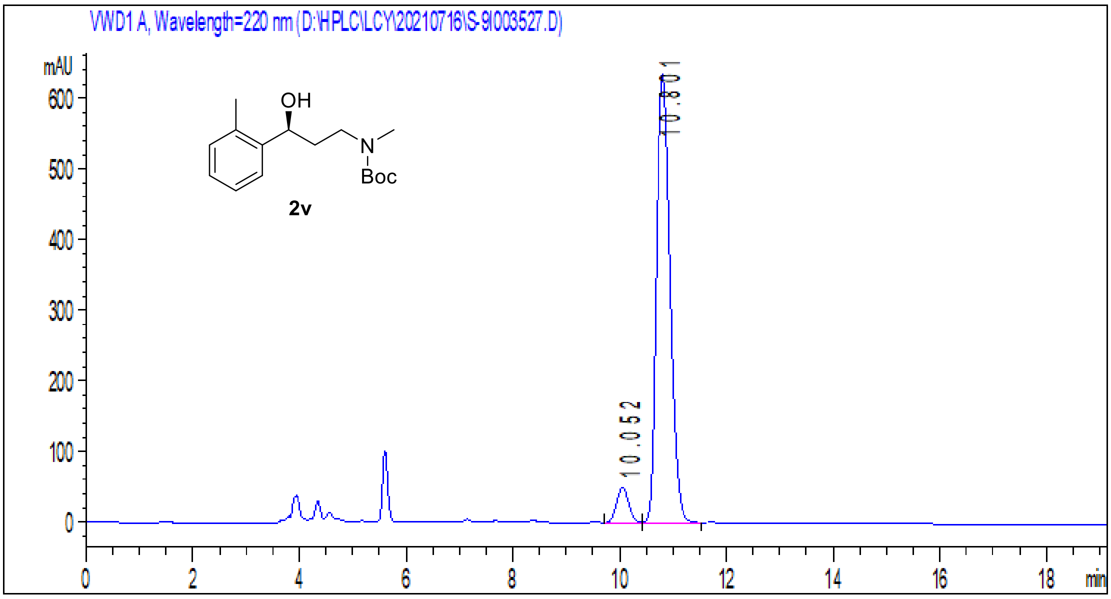

#	Time	Area	Height	Width	Symmetry	Area %
1	10.052	793.3	51	0.2429	0.913	6.594
2	10.801	11237.8	635.4	0.2792	0.717	93.406

Supplementary Figure 214. HPLC spectra of **2v**

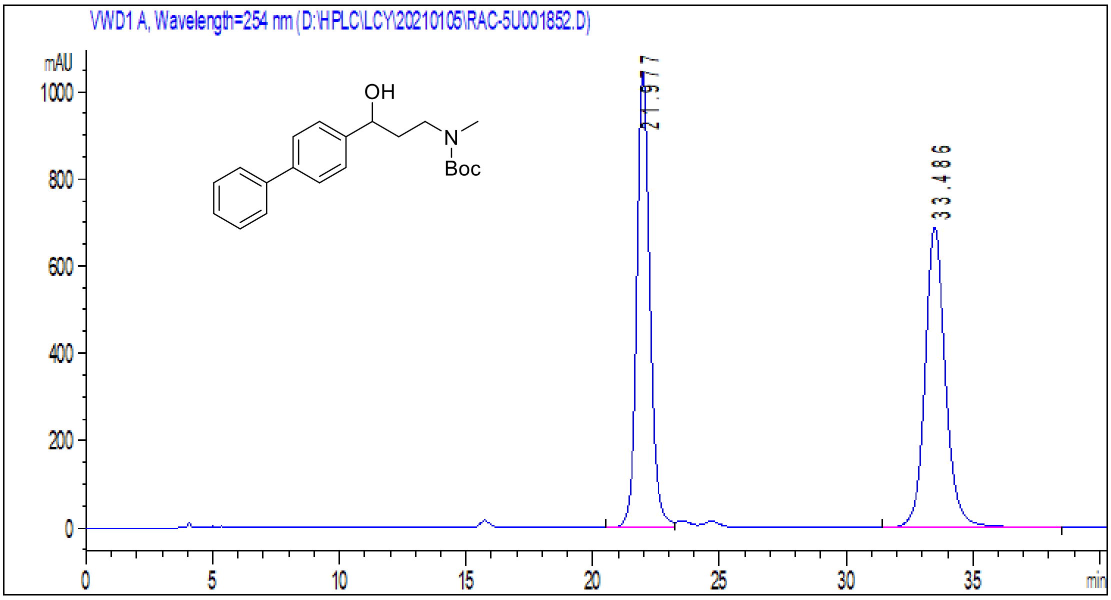

#	Time	Area	Height	Width	Symmetry	Area %
1	21.977	37256.5	1043	0.545	0.827	49.248
2	33.486	38393.7	687.1	0.8502	0.83	50.752

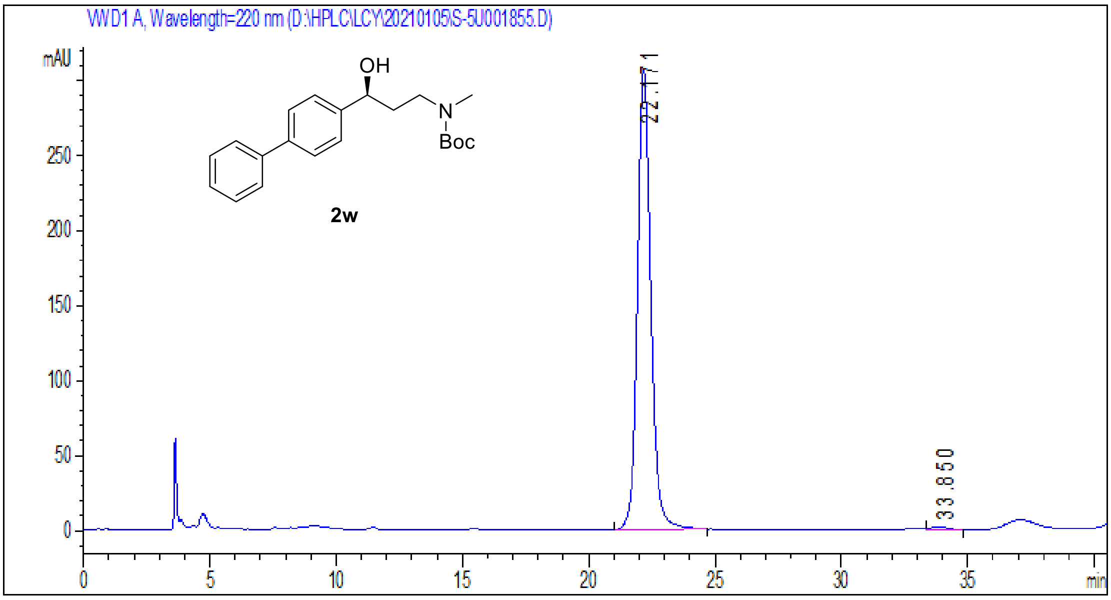

#	Time	Area	Height	Width	Symmetry	Area %
1	22.171	11177.5	306.4	0.5568	0.811	99.038
2	33.85	108.6	2.2	0.5807	0.902	0.962

Supplementary Figure 215. HPLC spectra of **2w**

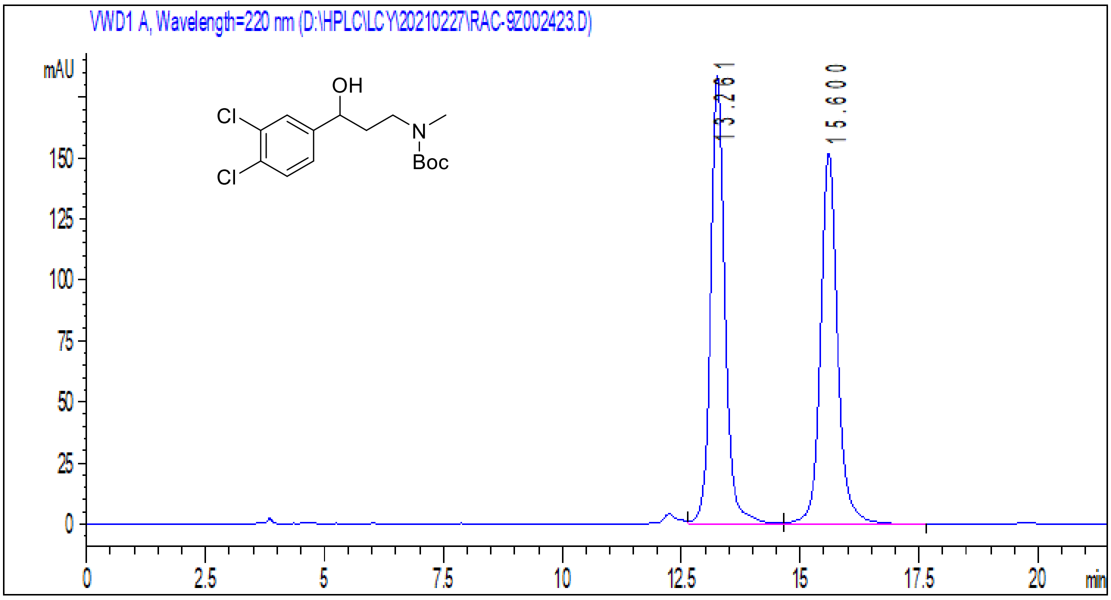

#	Time	Area	Height	Width	Symmetry	Area %
1	13.261	3837.8	183.7	0.3137	0.841	50.469
2	15.6	3766.4	151.8	0.3741	0.861	49.531

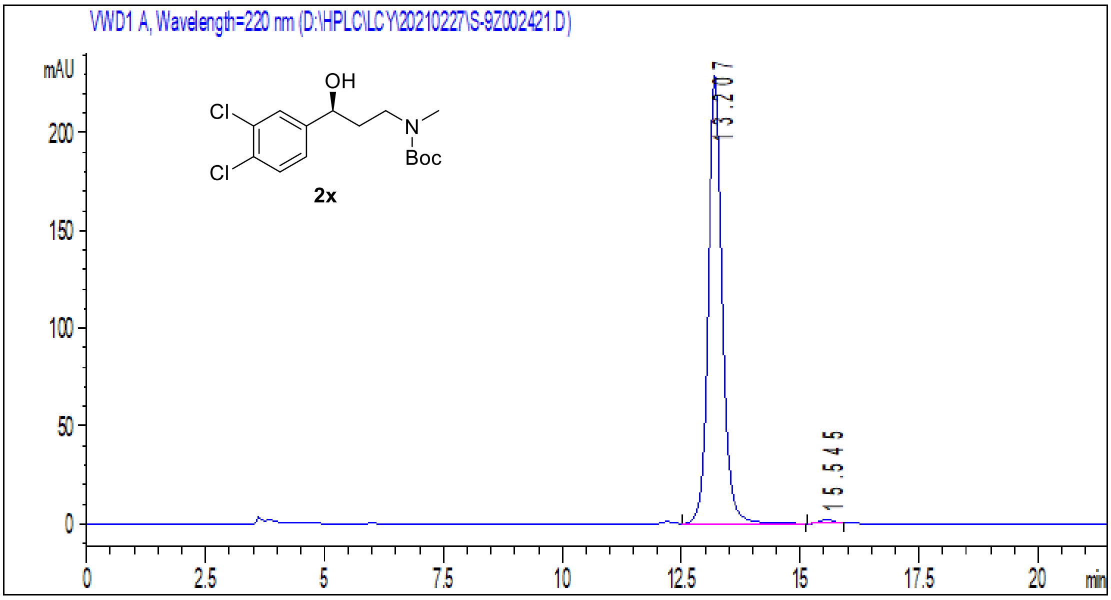

#	Time	Area	Height	Width	Symmetry	Area %
1	13.207	4701.6	229.2	0.3093	0.853	99.321
2	15.545	32.1	1.7	0.2935	0.973	0.679

Supplementary Figure 216. HPLC spectra of **2x**

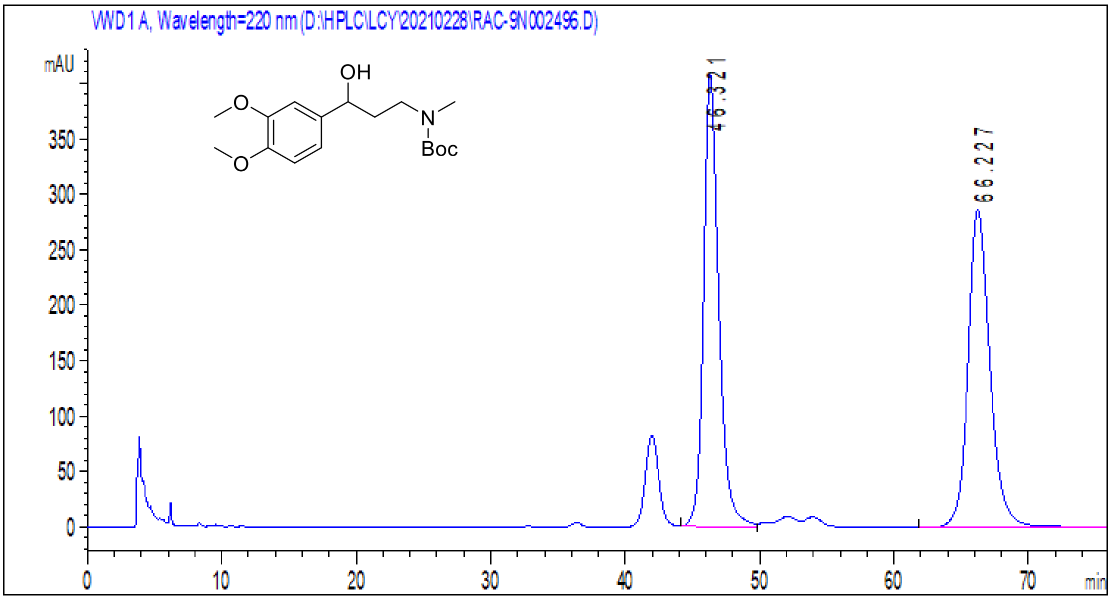

#	Time	Area	Height	Width	Symmetry	Area %
1	46.321	32152	409	1.1853	0.71	49.831
2	66.227	32370.5	286.2	1.7045	0.754	50.169

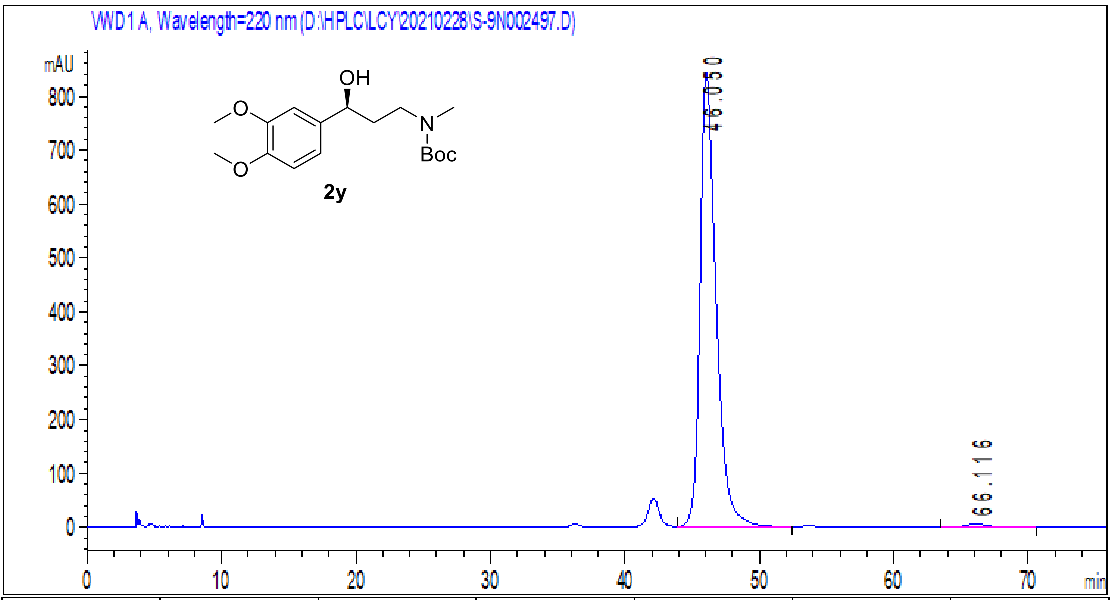

#	Time	Area	Height	Width	Symmetry	Area %
1	46.05	70337.4	842.6	1.2537	0.585	99.016
2	66.116	699.2	6.4	1.5442	0.896	0.984

Supplementary Figure 217. HPLC spectra of **2y**

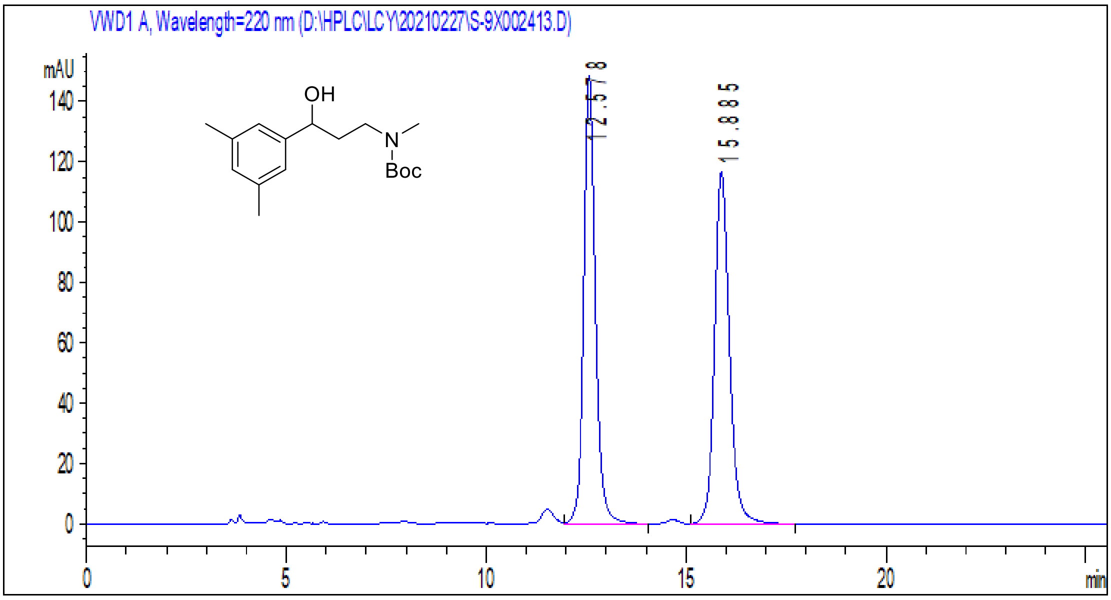

#	Time	Area	Height	Width	Symmetry	Area %
1	12.454	3546.9	174	0.3078	0.818	49.923
2	15.664	3557.8	138.3	0.3906	0.799	50.077

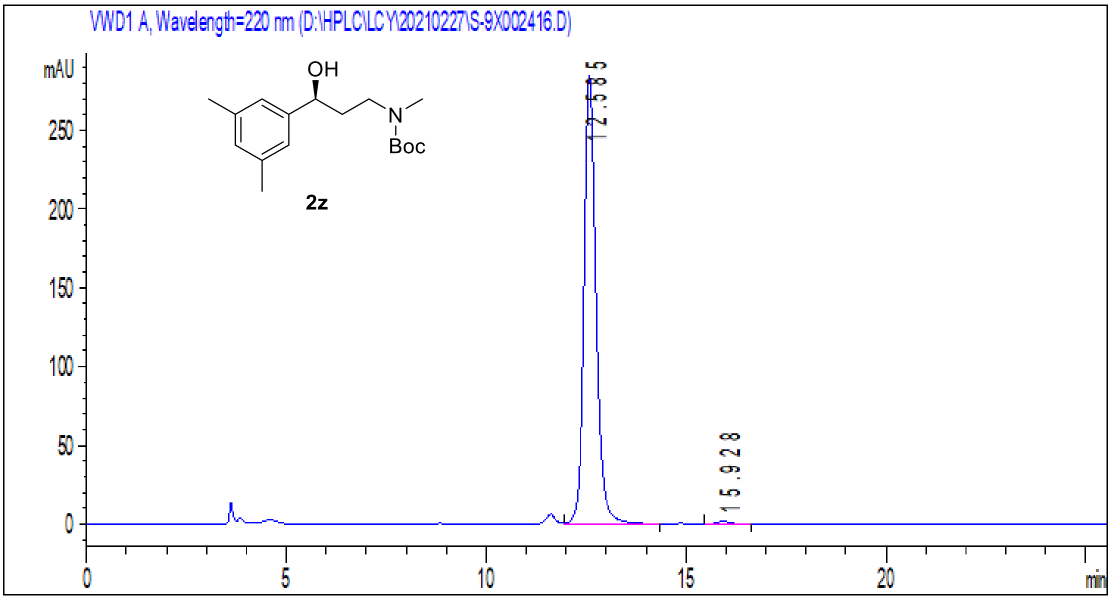

#	Time	Area	Height	Width	Symmetry	Area %
1	12.585	5897.8	284.5	0.3147	0.779	99.424
2	15.928	34.1	1.5	0.3456	0.897	0.576

Supplementary Figure 218. HPLC spectra of **2z**

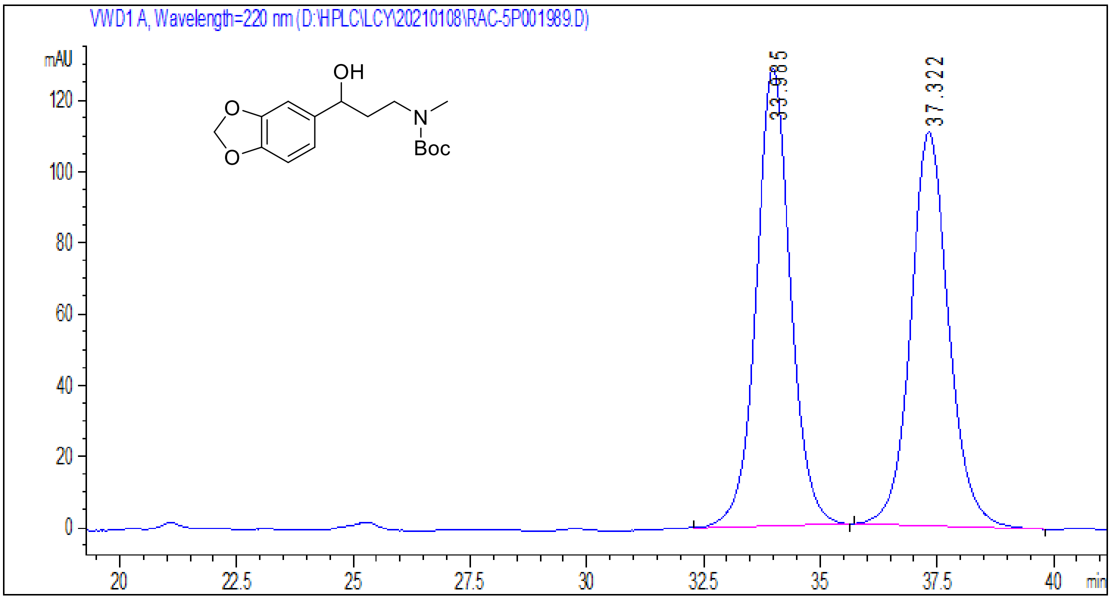

#	Time	Area	Height	Width	Symmetry	Area %
1	33.985	6576.5	128.4	0.7725	0.938	51.314
2	37.321	6239.6	110.4	0.8576	0.871	48.686

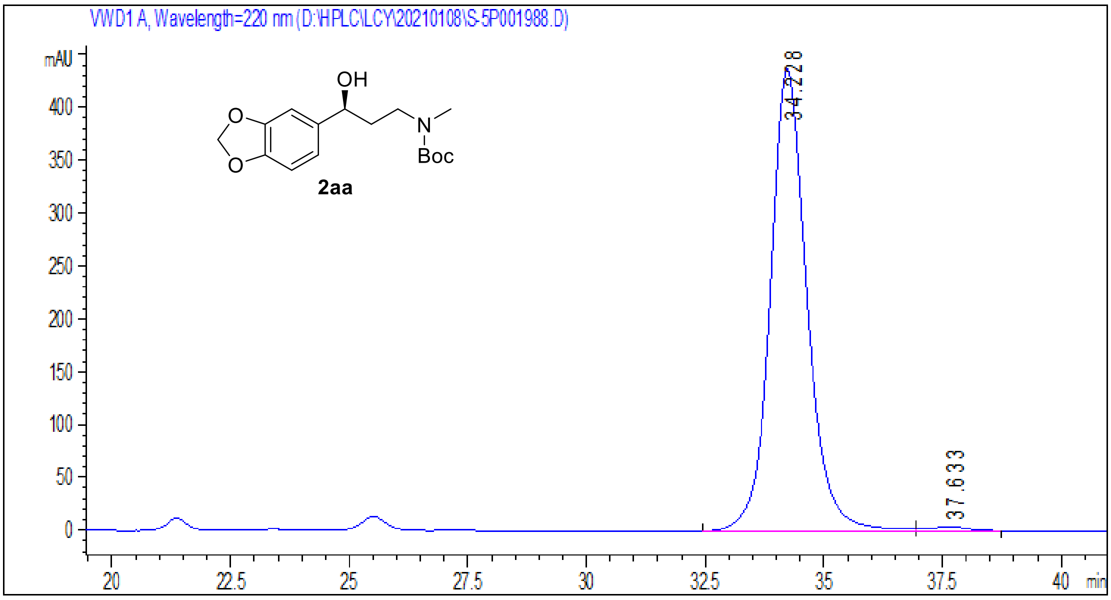

#	Time	Area	Height	Width	Symmetry	Area %
1	34.228	23223.2	436.3	0.8075	0.833	99.475
2	37.63	122.6	2.6	0.6044	0.795	0.525

Supplementary Figure 219. HPLC spectra of **2aa**

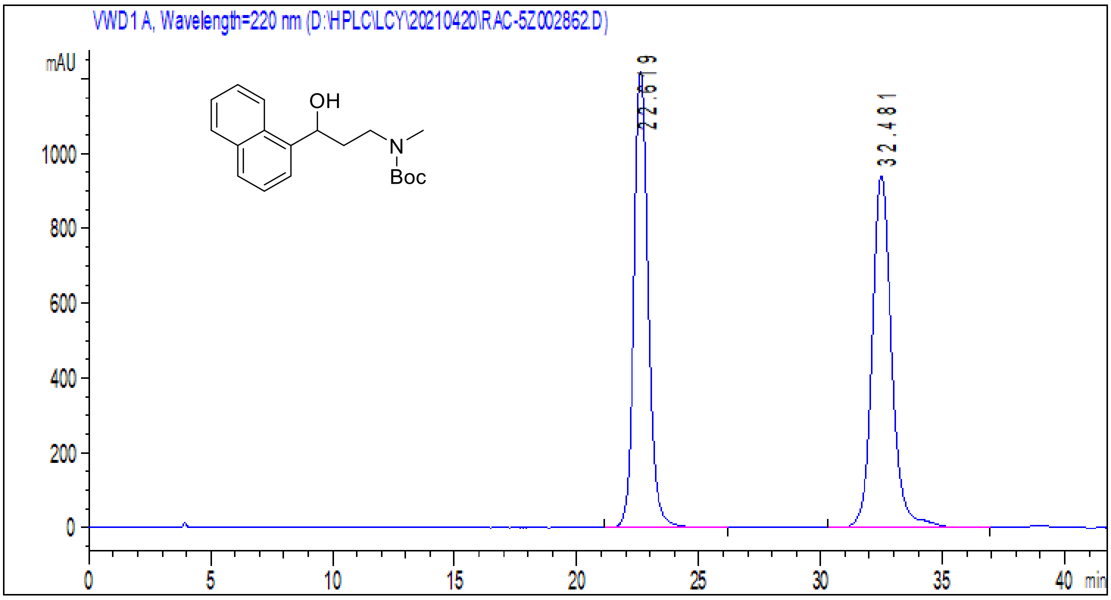

#	Time	Area	Height	Width	Symmetry	Area %
1	22.619	49608.8	1217.7	0.6269	0.82	48.626
2	32.481	52411.6	942.6	0.8528	0.807	51.374

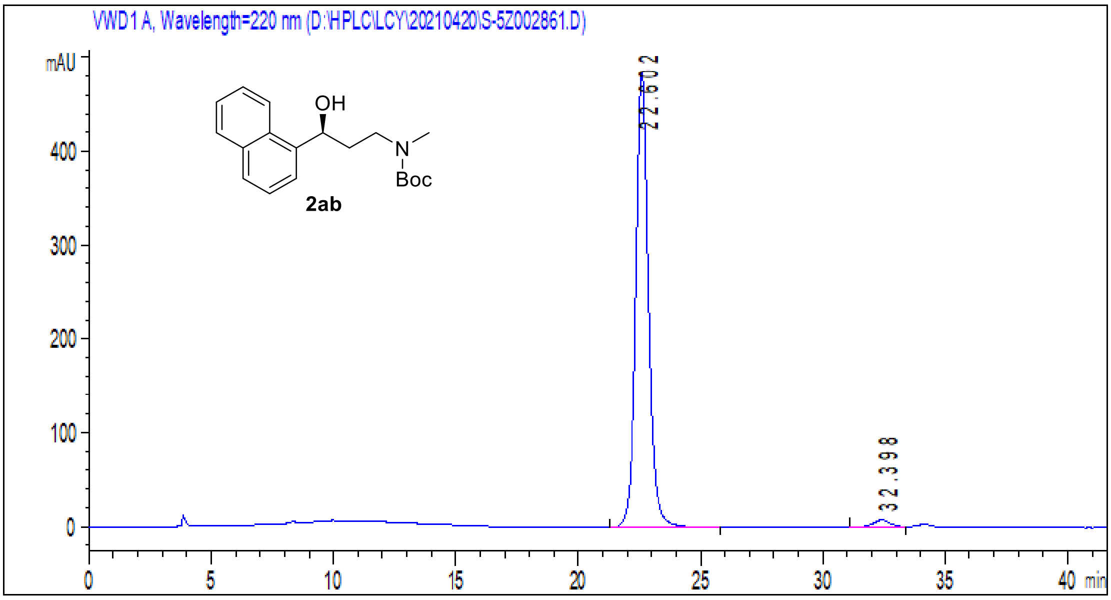

#	Time	Area	Height	Width	Symmetry	Area %
1	22.602	17465.6	485.1	0.5483	0.86	97.870
2	32.398	380	7.4	0.6785	0.928	2.130

Supplementary Figure 220. HPLC spectra of **2ab**

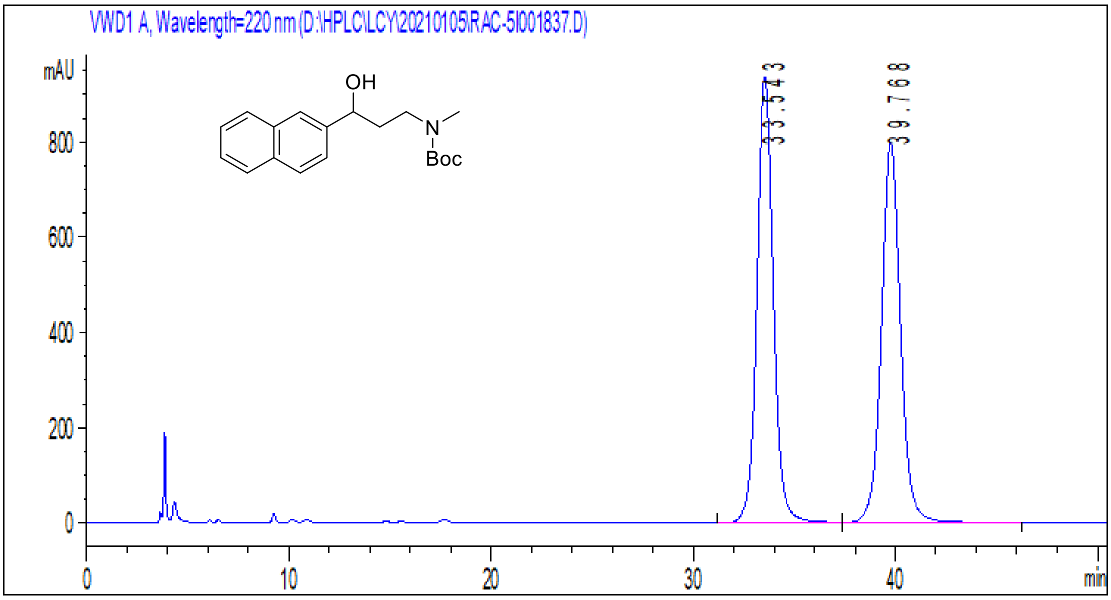

#	Time	Area	Height	Width	Symmetry	Area %
1	33.543	52963.3	935.8	0.8702	0.891	50.111
2	39.768	52728.8	799.8	1.0024	0.906	49.889

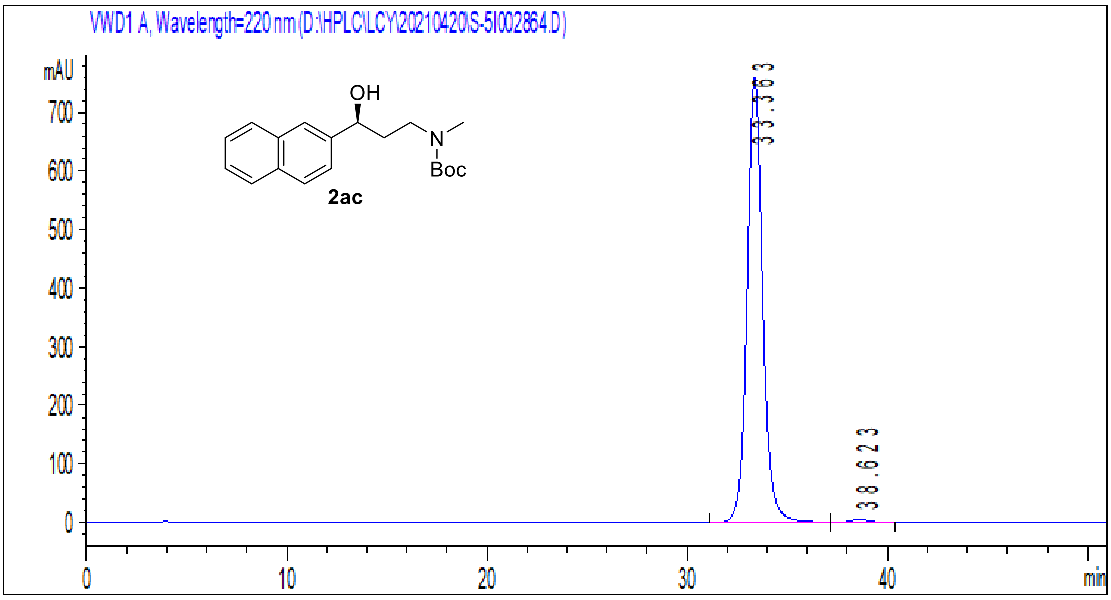

#	Time	Area	Height	Width	Symmetry	Area %
1	33.363	41146.7	759.6	0.8302	0.85	98.907
2	38.623	454.6	6.7	0.9739	0.995	1.093

Supplementary Figure 221. HPLC spectra of **2ac**

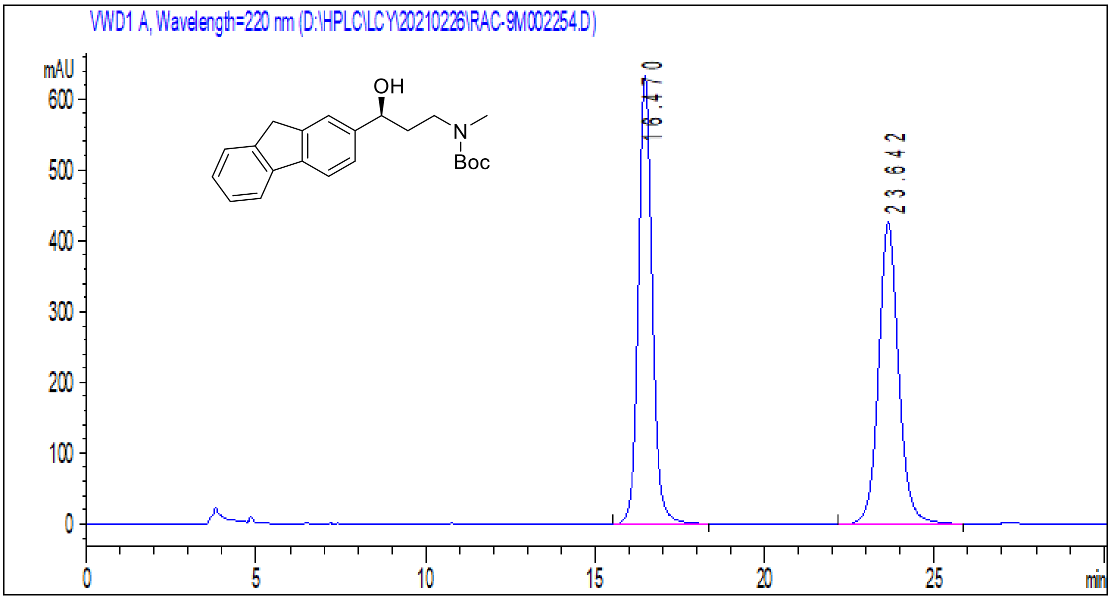

#	Time	Area	Height	Width	Symmetry	Area %
1	16.47	17849.1	632.8	0.4311	0.867	50.493
2	23.642	17500.5	425.7	0.6268	0.873	49.507

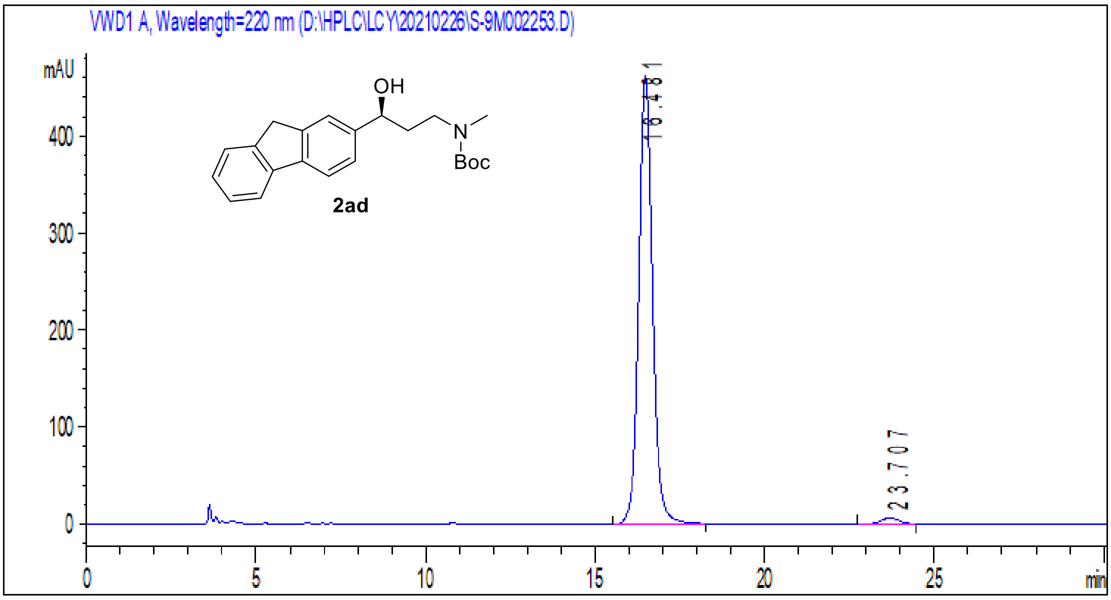

#	Time	Area	Height	Width	Symmetry	Area %
1	16.481	12903.7	461.8	0.4251	0.88	98.272
2	23.707	226.9	6	0.4483	1.157	1.728

Supplementary Figure 222. HPLC spectra of **2ad**

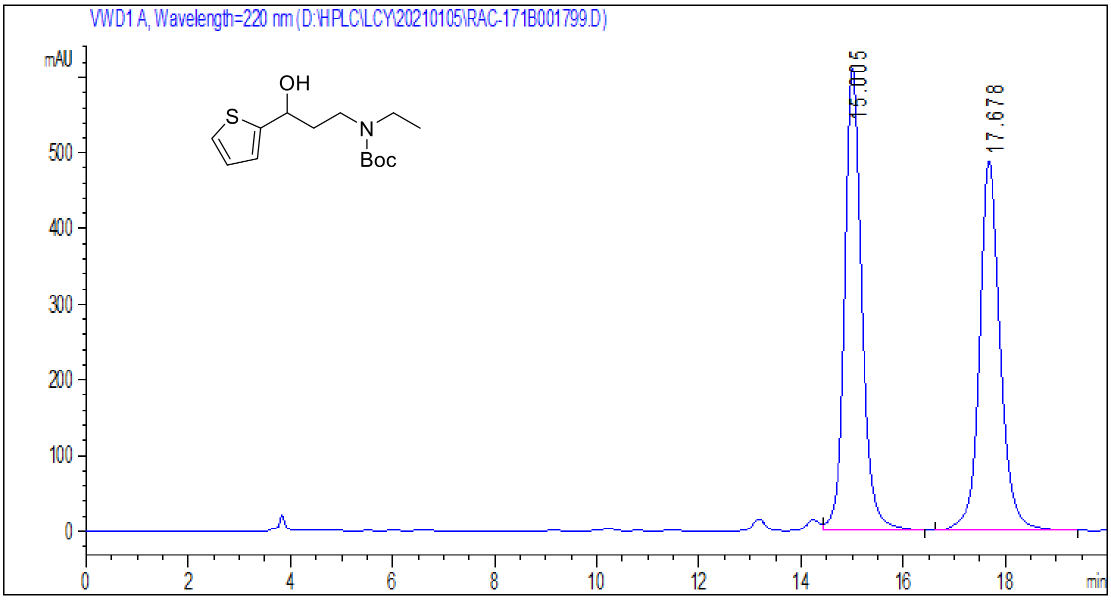

#	Time	Area	Height	Width	Symmetry	Area %
1	15.005	14299.6	610.1	0.3551	0.792	51.057
2	17.678	13707.7	488.1	0.4239	0.807	48.943

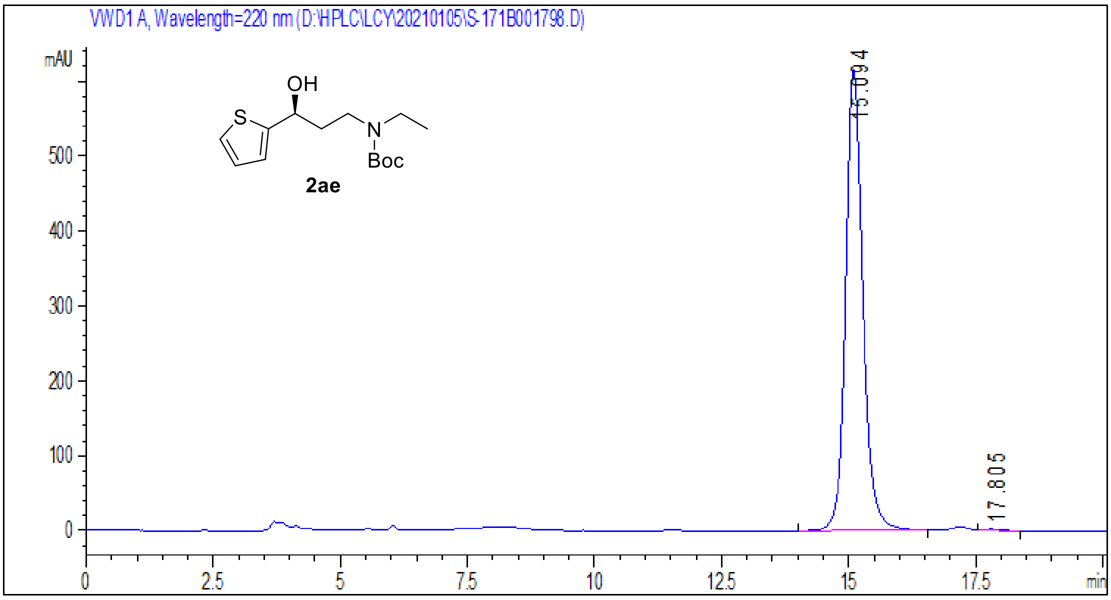

#	Time	Area	Height	Width	Symmetry	Area %
1	15.094	14420.4	616.2	0.3532	0.793	99.659
2	17.805	49.3	2.1	0.3011	0.826	0.341

Supplementary Figure 223. HPLC spectra of **2ae**

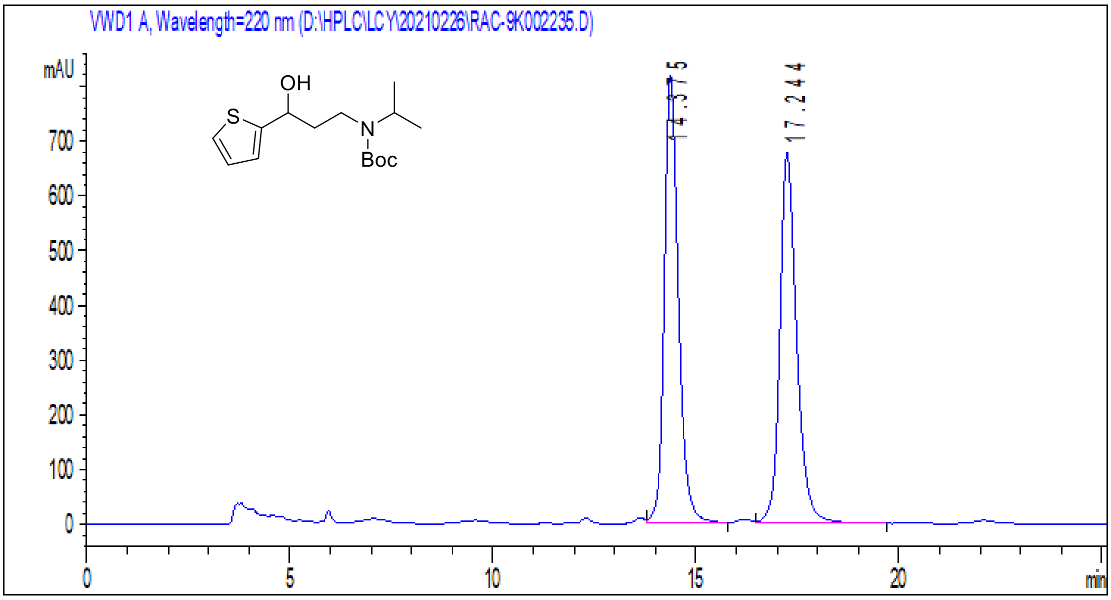

#	Time	Area	Height	Width	Symmetry	Area %
1	14.375	19462.2	817	0.3595	0.728	49.992
2	17.244	19468.2	676.5	0.4349	0.709	50.008

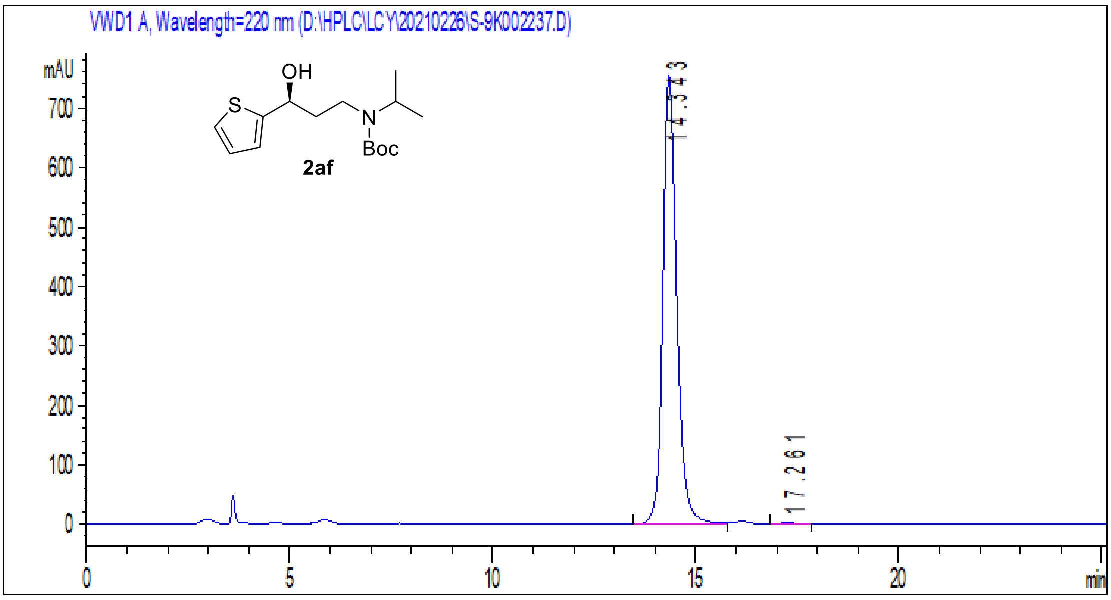

#	Time	Area	Height	Width	Symmetry	Area %
1	14.343	17742.4	753.9	0.3562	0.737	99.637
2	17.261	64.6	2.3	0.3381	0.846	0.363

Supplementary Figure 224. HPLC spectra of **2af**

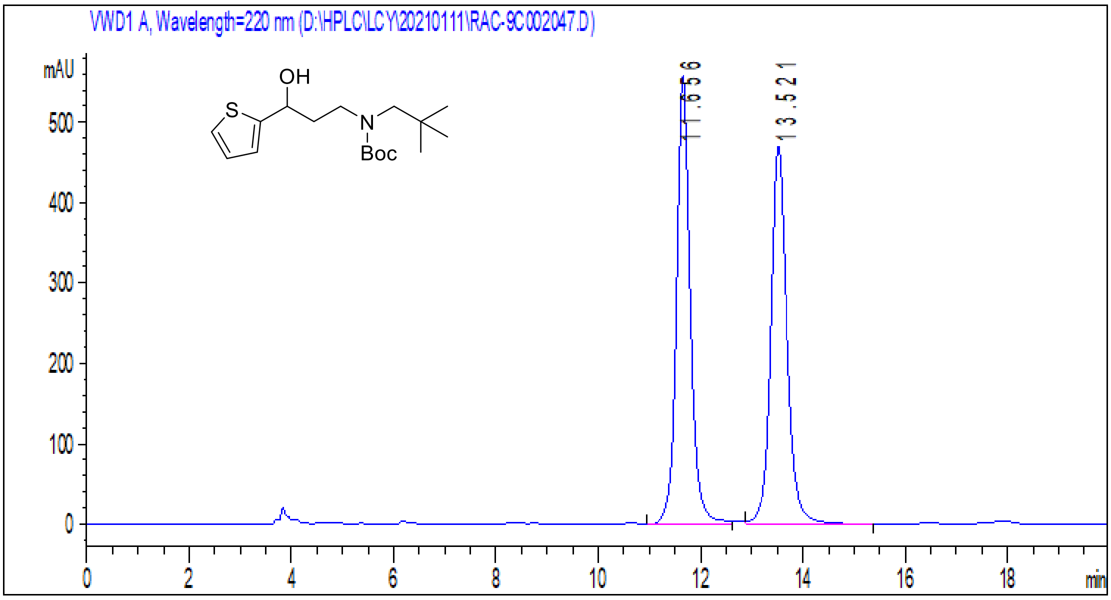

#	Time	Area	Height	Width	Symmetry	Area %
1	11.656	10515.2	557.5	0.2842	0.832	50.581
2	13.522	10273.6	469.4	0.3312	0.827	49.419

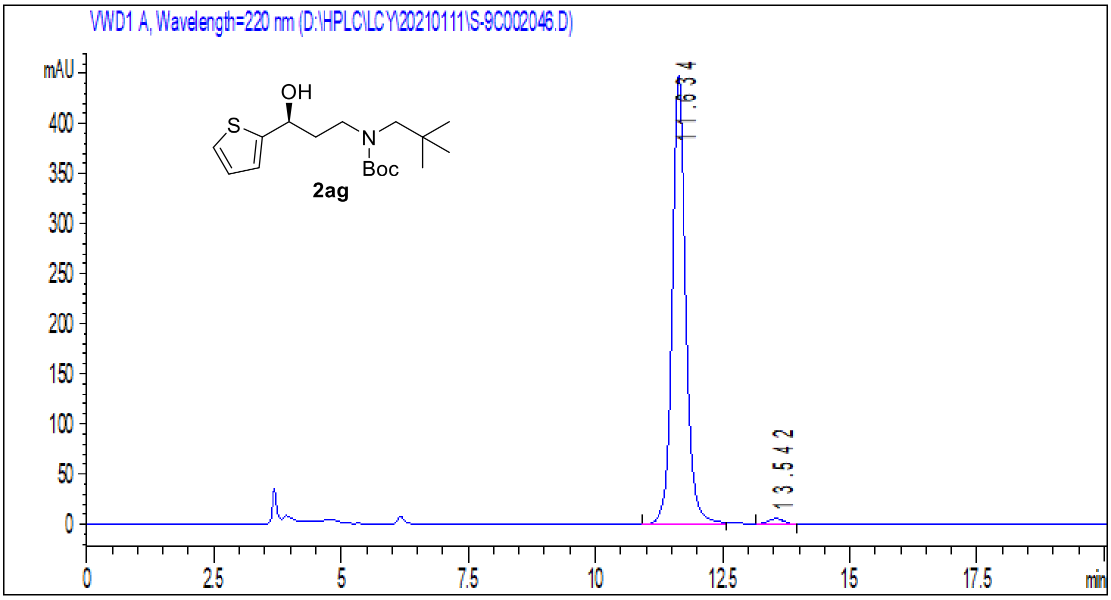

#	Time	Area	Height	Width	Symmetry	Area %
1	11.634	8294.2	447.6	0.2803	0.844	98.860
2	13.542	95.6	5	0.3189	0.912	1.140

Supplementary Figure 225. HPLC spectra of **2ag**

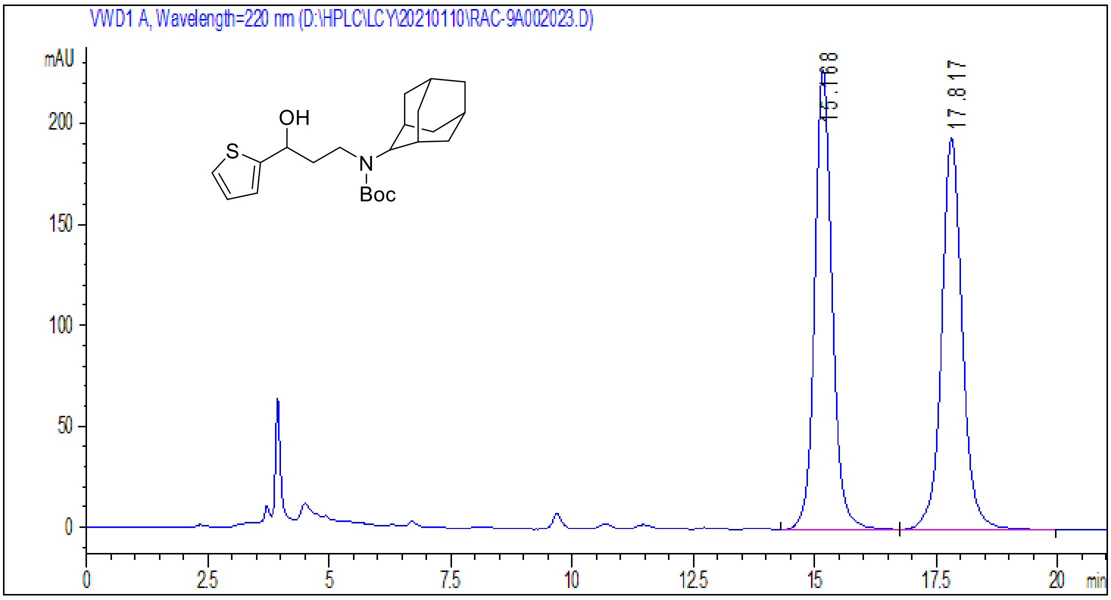

#	Time	Area	Height	Width	Symmetry	Area %
1	15.168	5666.3	227.4	0.3781	0.852	49.736
2	17.818	5726.5	193.8	0.4467	0.878	50.264

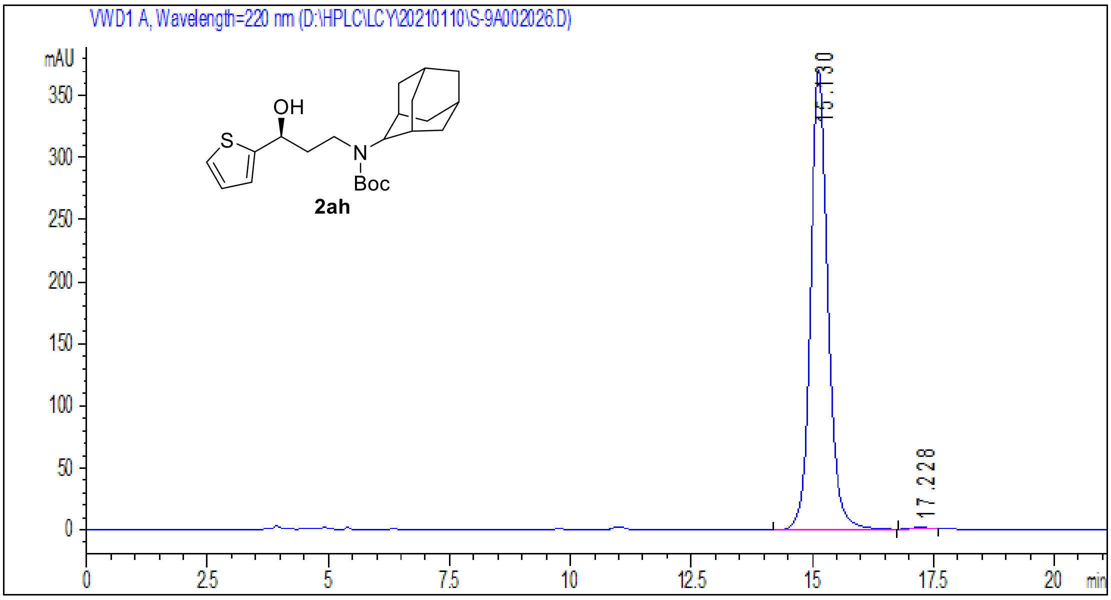

#	Time	Area	Height	Width	Symmetry	Area %
1	15.13	9236.5	370.7	0.3781	0.826	99.597
2	17.228	37.4	1.7	0.3423	1.064	0.403

Supplementary Figure 226. HPLC spectra of **2ah**

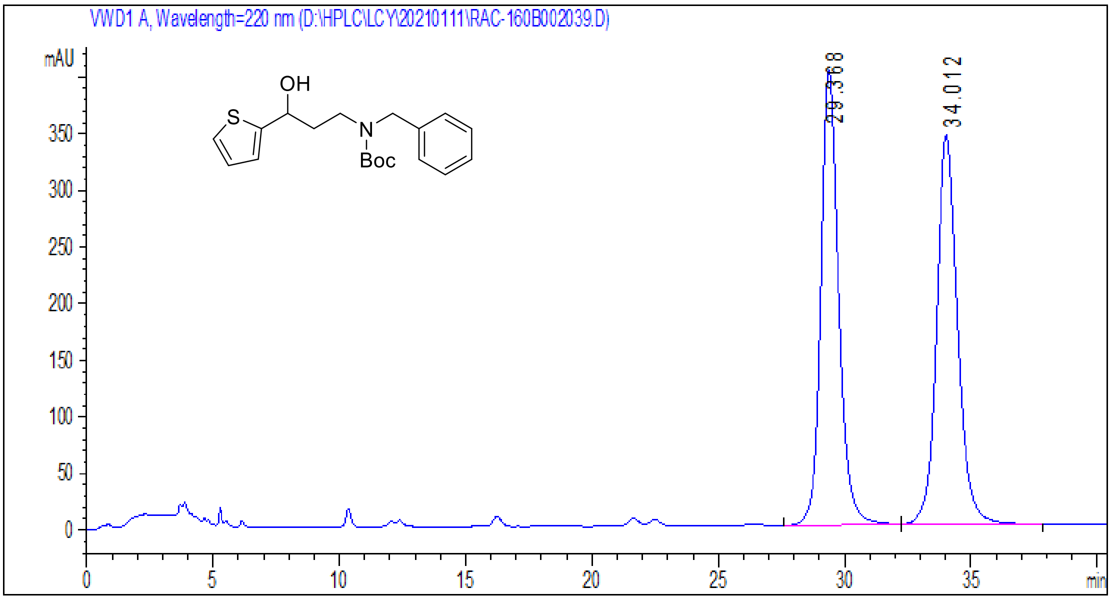

#	Time	Area	Height	Width	Symmetry	Area %
1	29.368	19592.5	401.7	0.7381	0.762	49.759
2	34.012	19782.3	343.3	0.8677	0.8	50.241

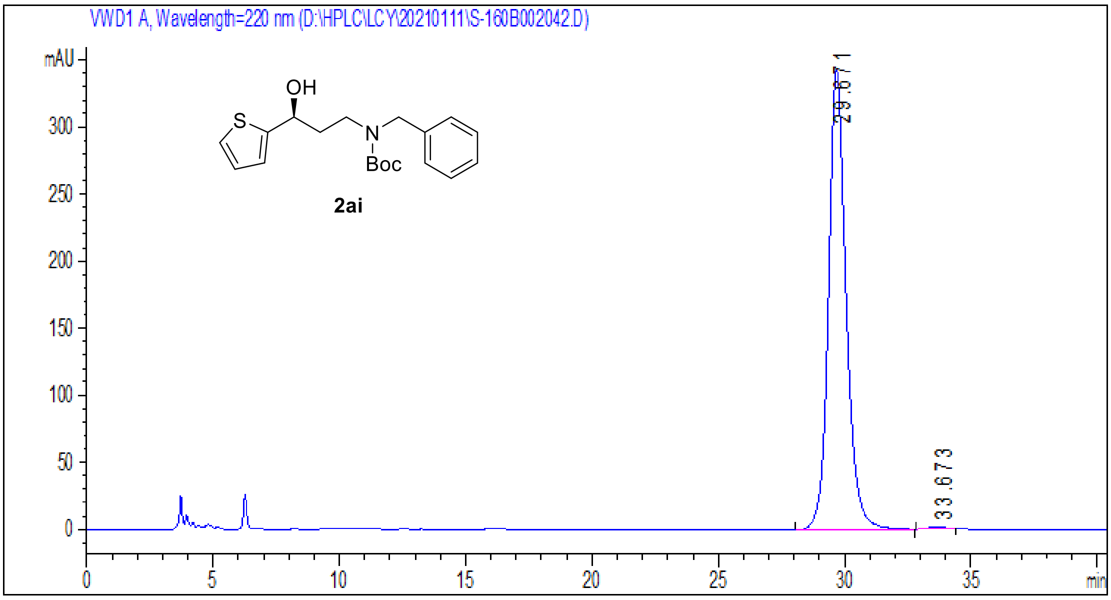

#	Time	Area	Height	Width	Symmetry	Area %
1	29.671	16676.7	343	0.7393	0.778	99.666
2	33.673	55.9	1.3	0.5359	1.594	0.334

Supplementary Figure 227. HPLC spectra of **2ai**

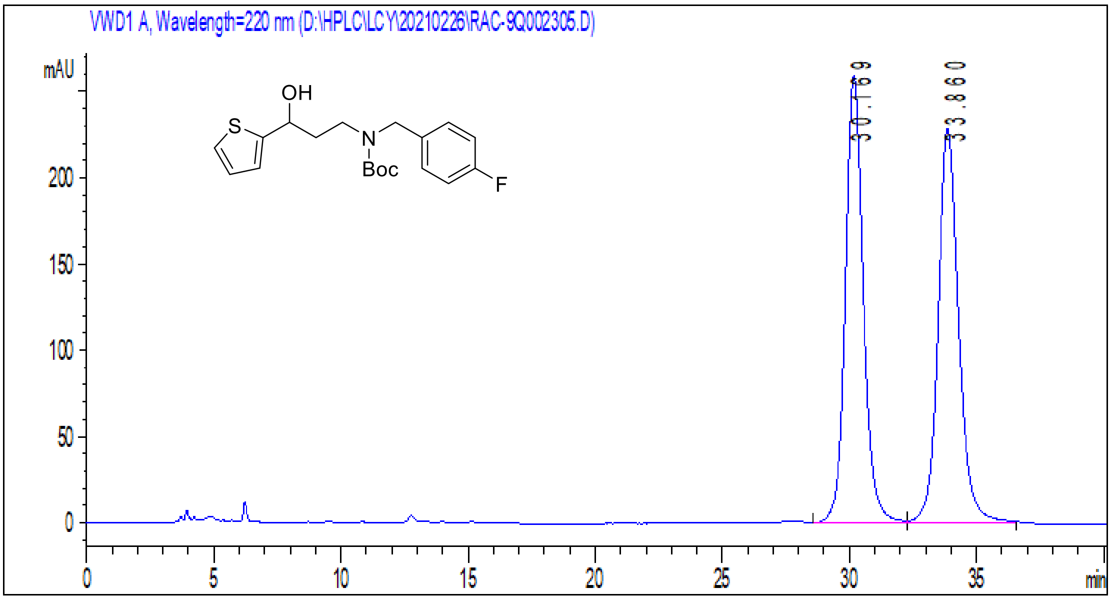

#	Time	Area	Height	Width	Symmetry	Area %
1	30.169	12642.4	259.2	0.7367	0.833	49.529
2	33.86	12882.8	229.2	0.8467	0.821	50.471

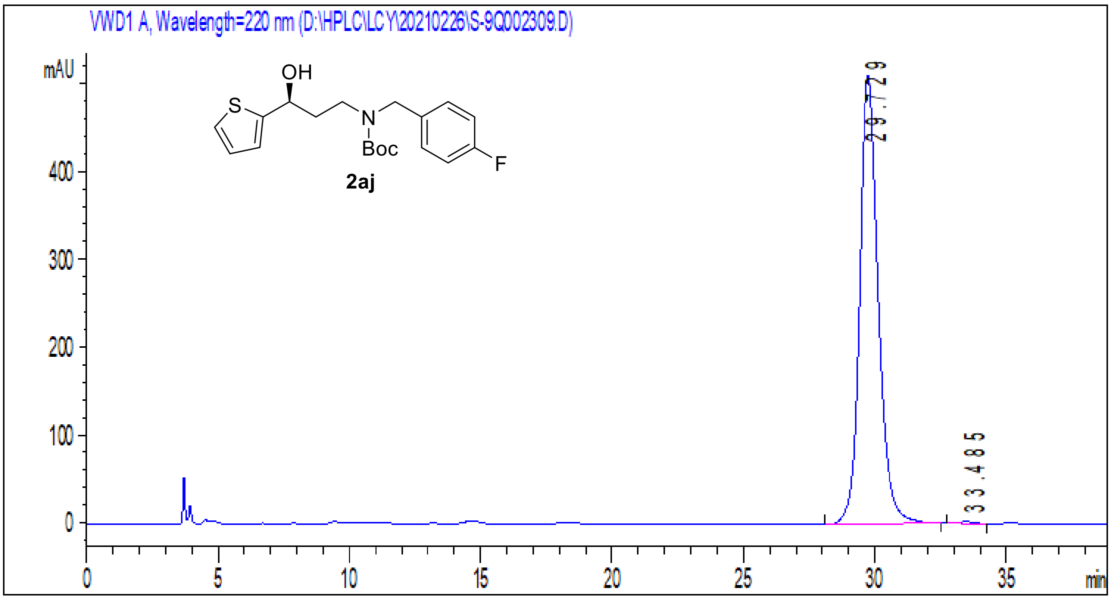

#	Time	Area	Height	Width	Symmetry	Area %
1	29.729	24966.8	510	0.7344	0.761	99.675
2	33.485	81.3	1.9	0.5144	0.908	0.325

Supplementary Figure 228. HPLC spectra of **2aj**

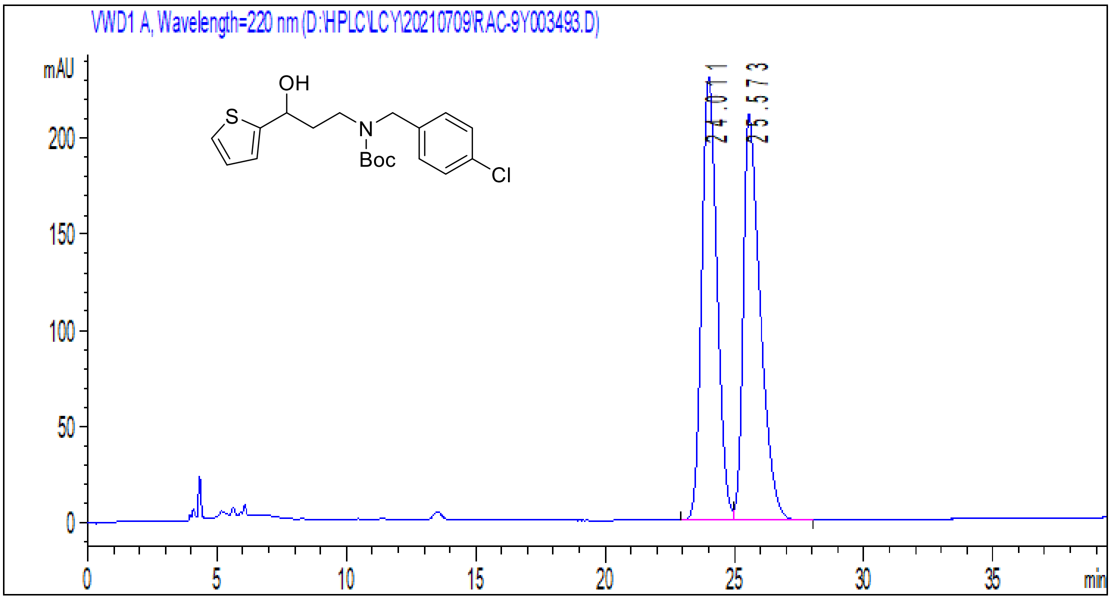

#	Time	Area	Height	Width	Symmetry	Area %
1	24.011	9555.1	231	0.6458	0.801	49.941
2	25.573	9577.8	211.7	0.6591	0.484	50.059

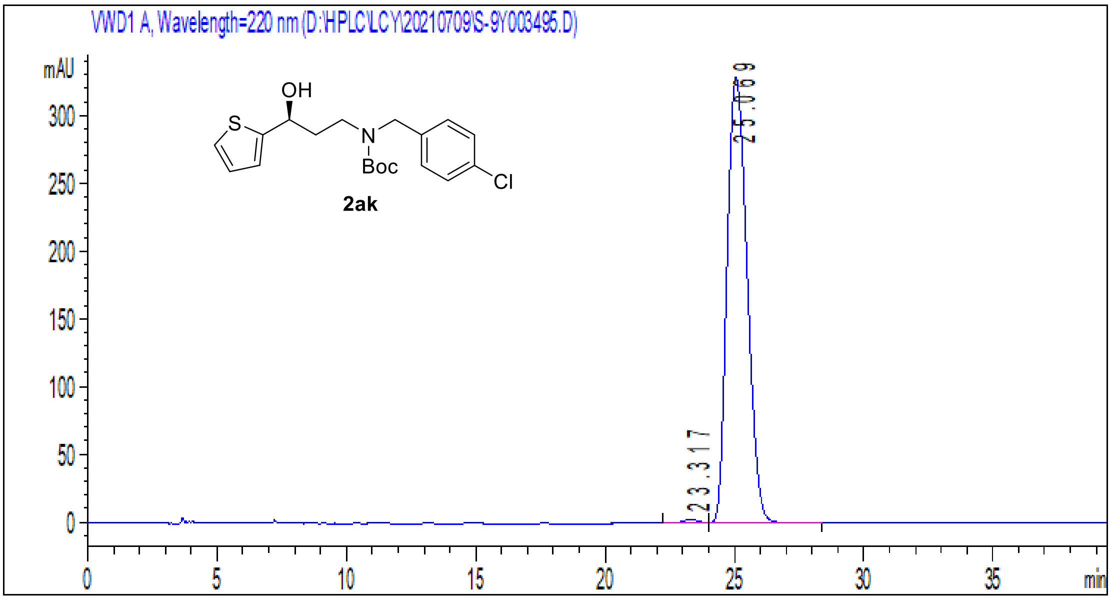

#	Time	Area	Height	Width	Symmetry	Area %
1	23.317	106.2	2.4	0.5913	0.978	0.596
2	25.069	17724.4	329.4	0.8668	0.773	99.404

Supplementary Figure 229. HPLC spectra of **2ak**

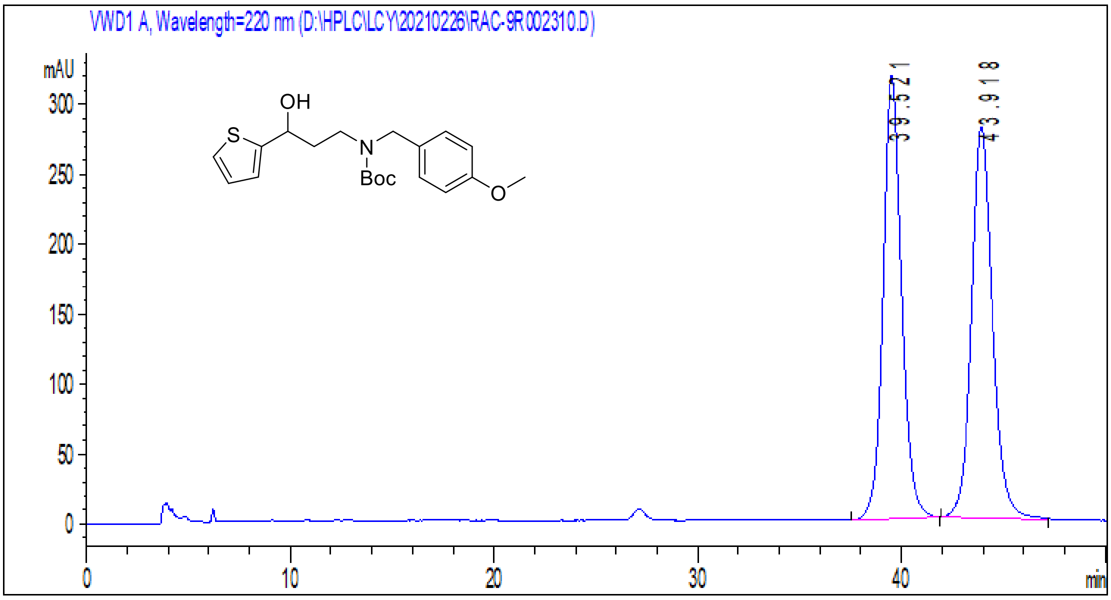

#	Time	Area	Height	Width	Symmetry	Area %
1	39.521	19942.6	316.8	0.9545	0.85	49.944
2	43.918	19987.7	279.7	1.0758	0.83	50.056

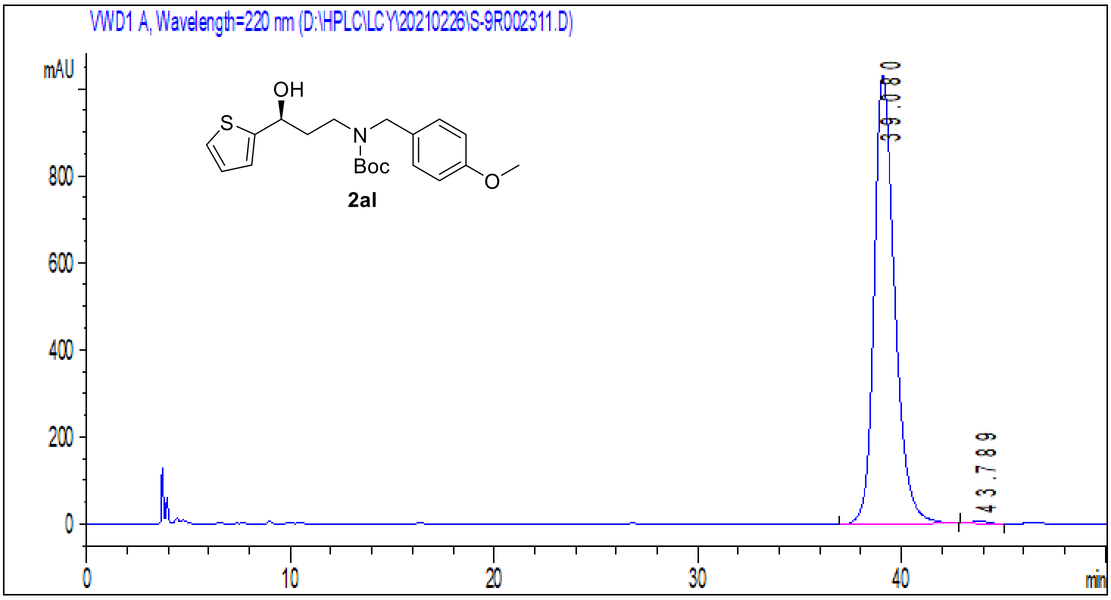

#	Time	Area	Height	Width	Symmetry	Area %
1	39.08	72277	1030.2	1.0634	0.678	99.598
2	43.789	291.9	5	0.6948	0.746	0.402

Supplementary Figure 230. HPLC spectra of **2al**

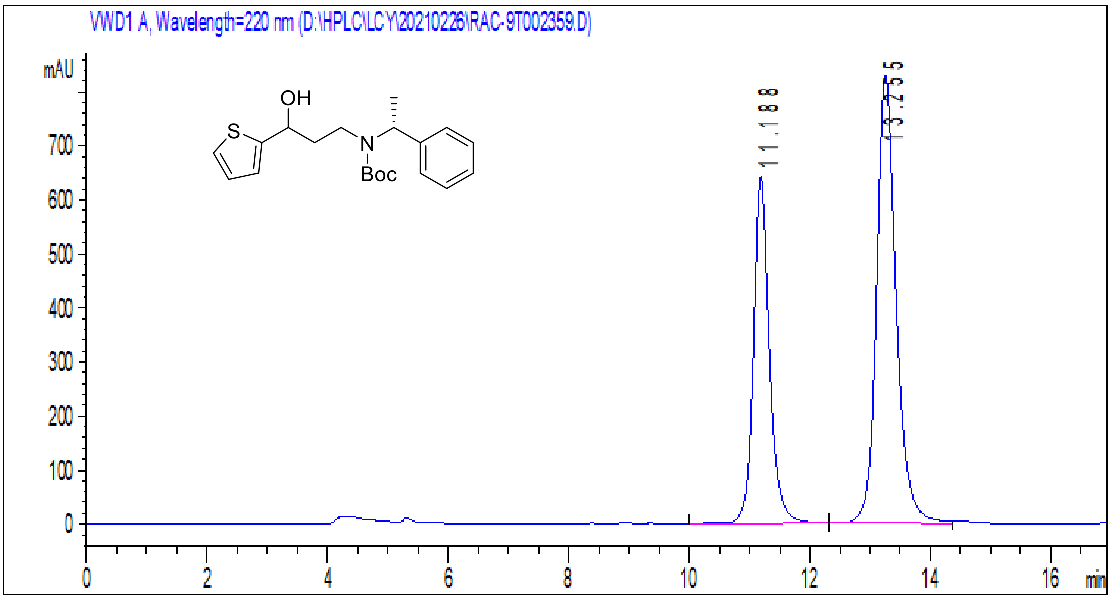

#	Time	Area	Height	Width	Symmetry	Area %
1	11.188	11830.8	641.1	0.2765	0.804	38.719
2	13.255	18724.7	827.3	0.3428	0.77	61.281

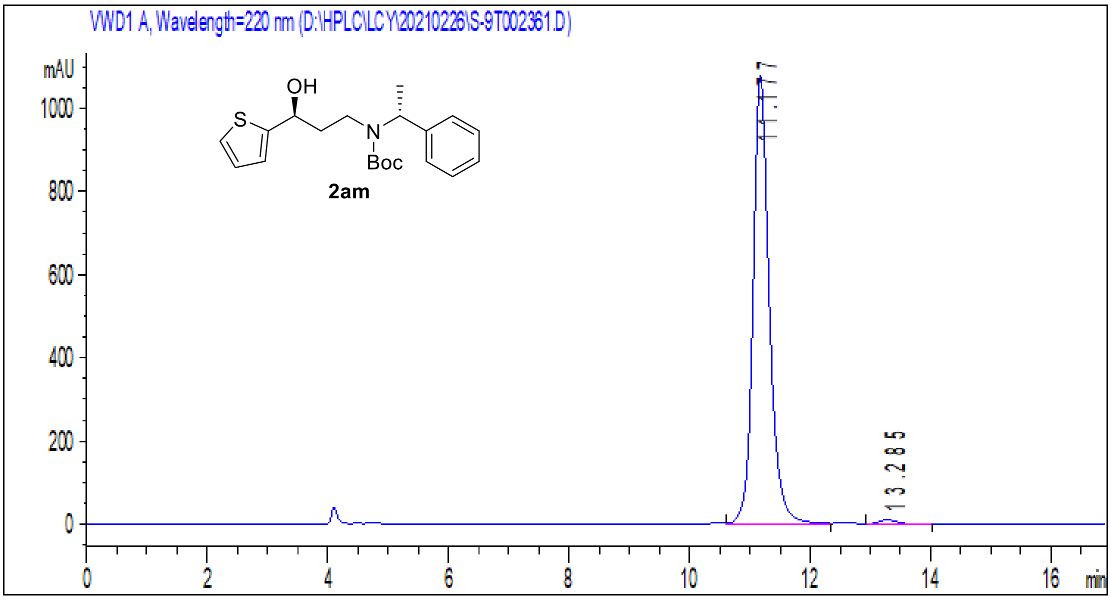

#	Time	Area	Height	Width	Symmetry	Area %
1	11.177	20339.4	1077.4	0.2873	0.772	99.123
2	13.285	179.9	9.1	0.2996	0.792	0.877

Supplementary Figure 231. HPLC spectra of **2am**

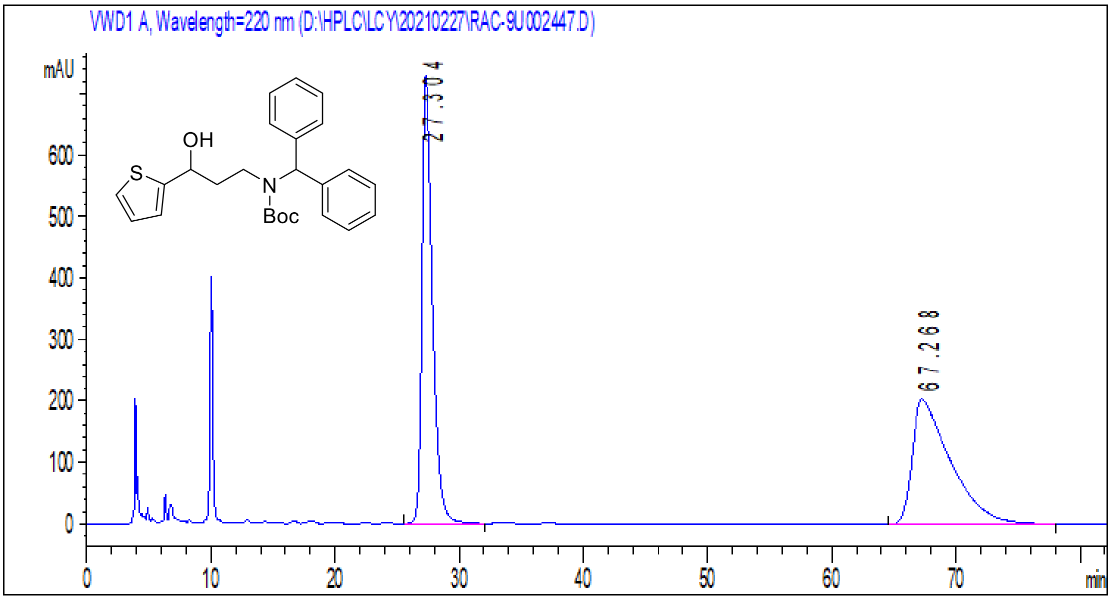

#	Time	Area	Height	Width	Symmetry	Area %
1	27.304	43048.1	728.6	0.8876	0.522	49.811
2	67.268	43374.5	203.3	2.7837	0.325	50.189

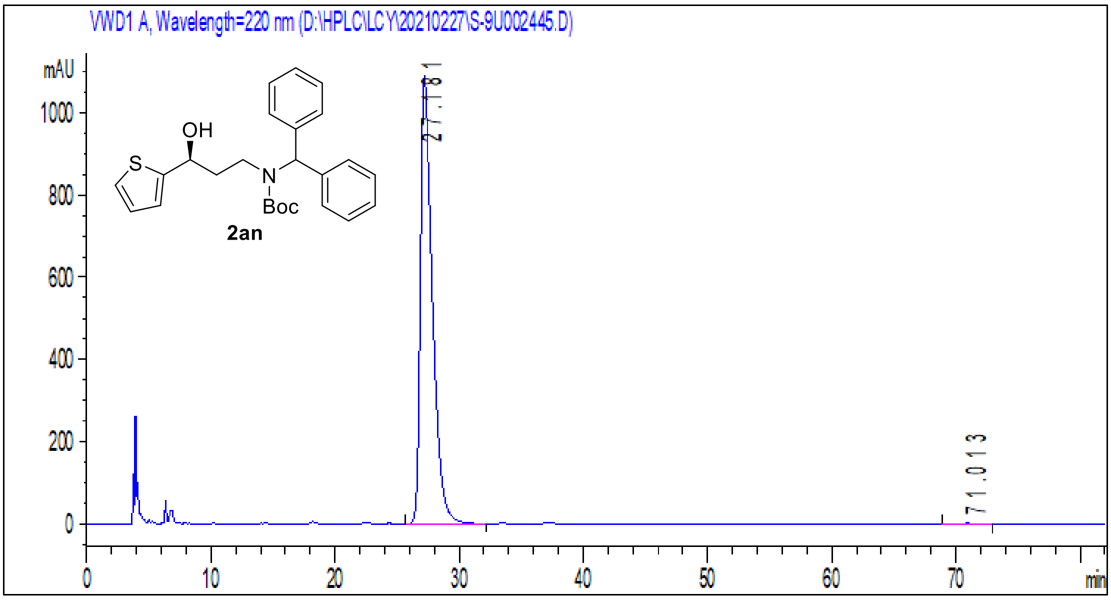

#	Time	Area	Height	Width	Symmetry	Area %
1	27.181	73346.1	1089.7	1.0068	0.436	99.631
2	71.013	271.5	2.2	1.4848	1.218	0.369

Supplementary Figure 232. HPLC spectra of **2an**

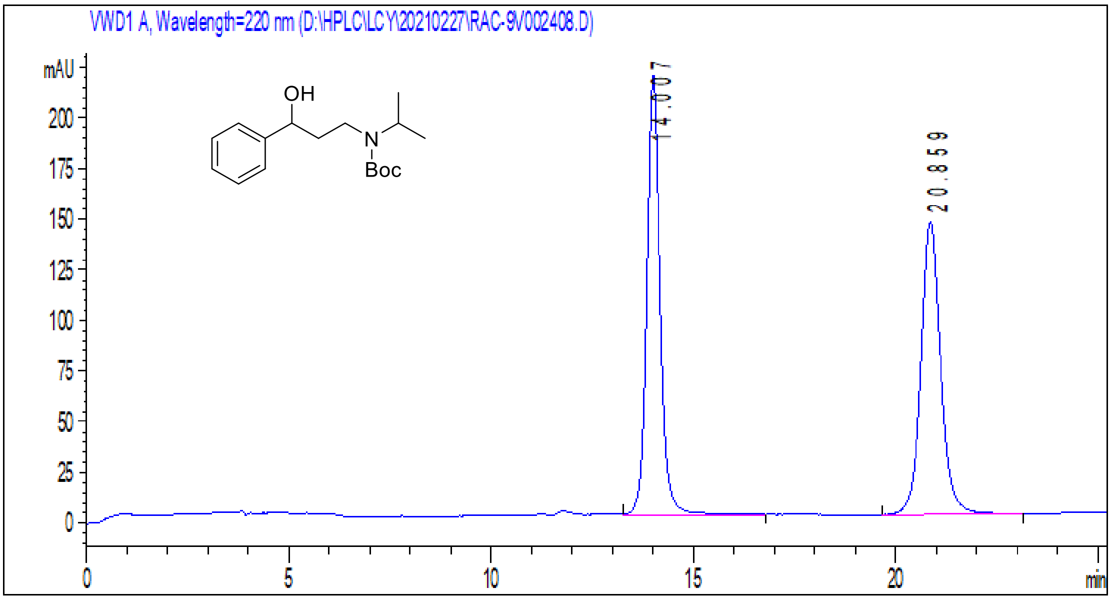

#	Time	Area	Height	Width	Symmetry	Area %
1	14.007	4935.5	216.6	0.3446	0.839	50.212
2	20.859	4893.9	144.5	0.5146	0.869	49.788

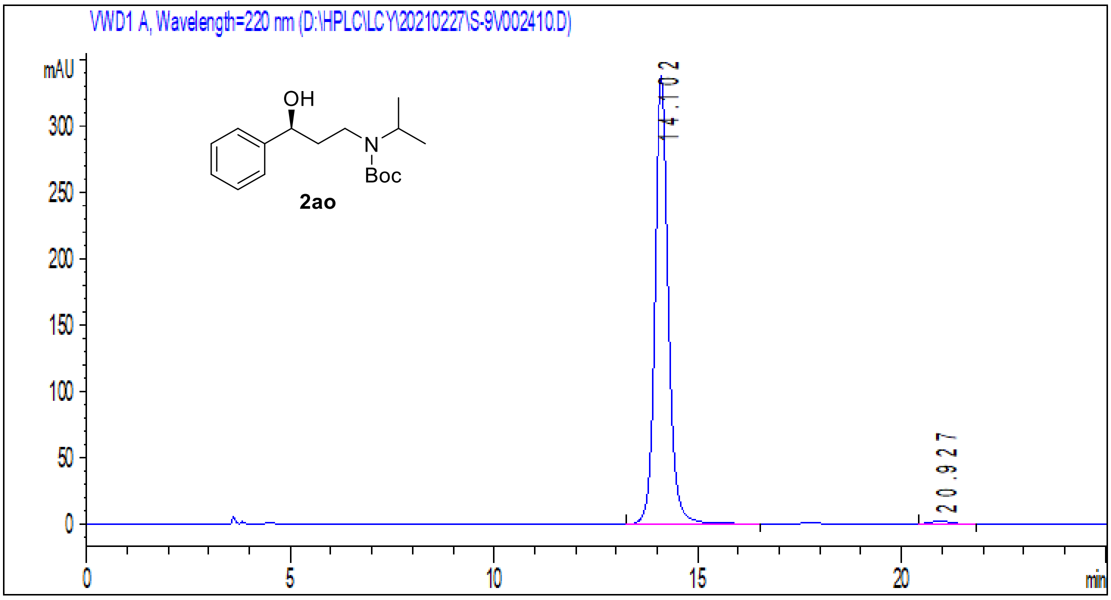

#	Time	Area	Height	Width	Symmetry	Area %
1	14.102	7708.2	337.8	0.3449	0.834	99.278
2	20.927	56	1.8	0.438	0.752	0.722

Supplementary Figure 233. HPLC spectra of **2ao**

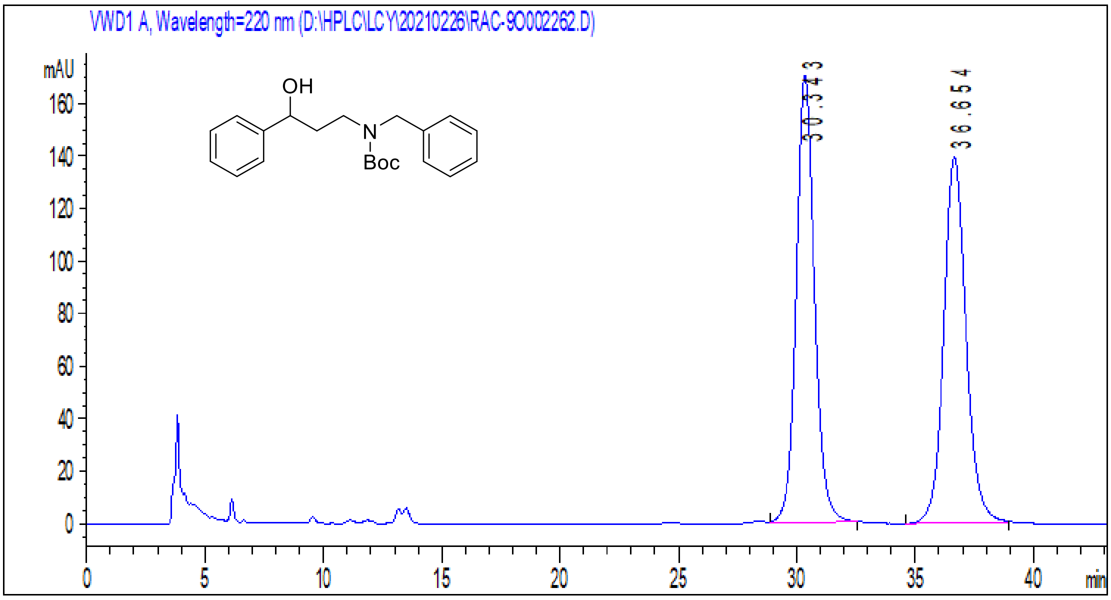

#	Time	Area	Height	Width	Symmetry	Area %
1	30.343	9064	170	0.797	0.852	49.851
2	36.654	9118.2	139.3	0.9852	0.853	50.149

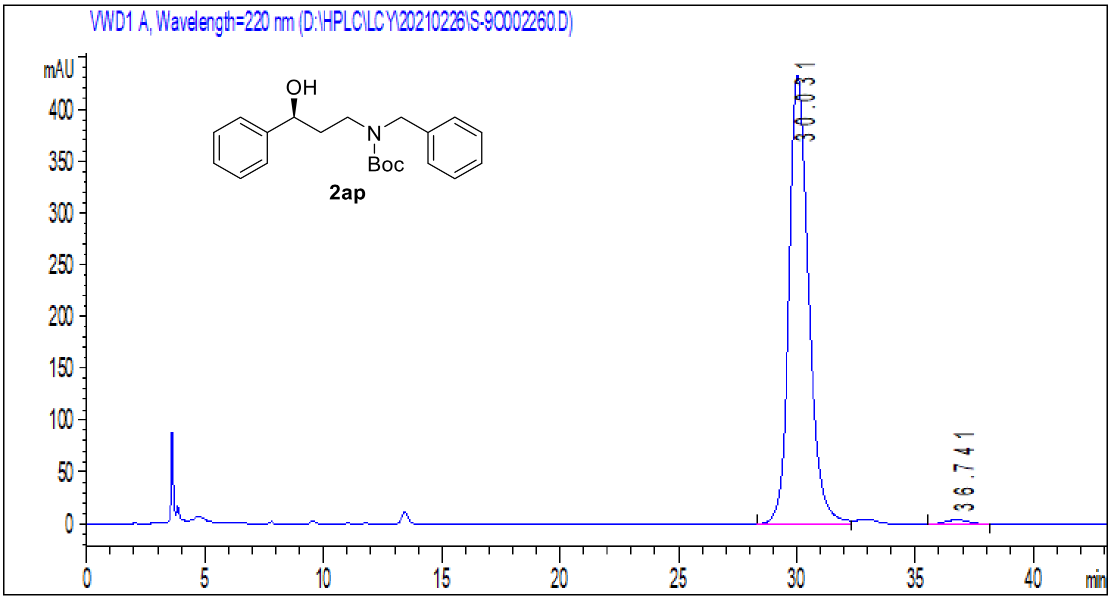

#	Time	Area	Height	Width	Symmetry	Area %
1	30.031	24882.8	432.4	0.8652	0.726	98.952
2	36.741	263.6	3.8	0.8189	0.852	1.048

Supplementary Figure 234. HPLC spectra of **2ap**

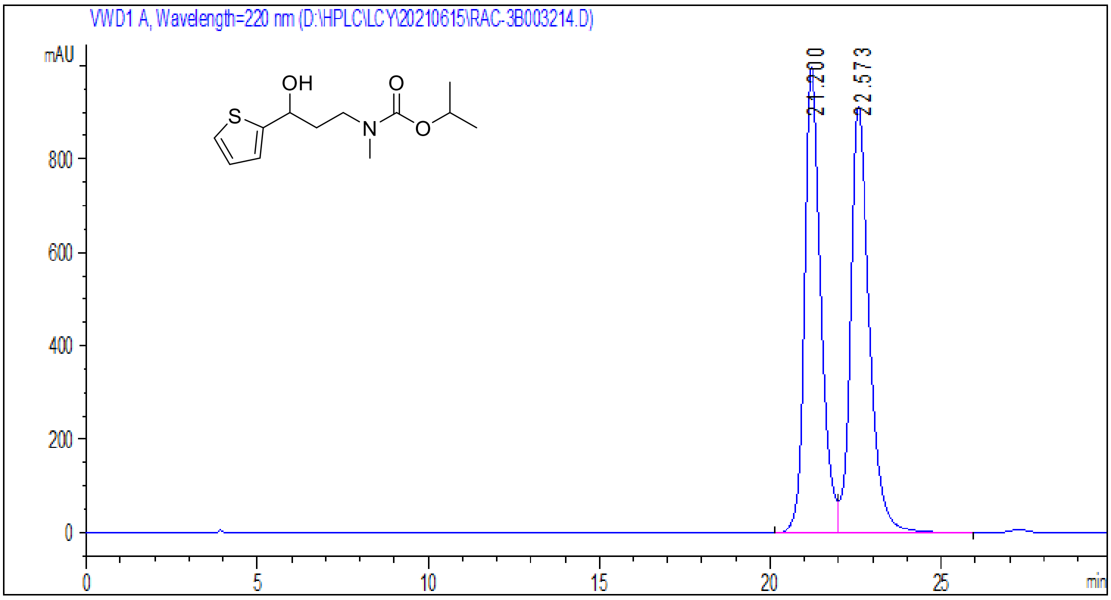

#	Time	Area	Height	Width	Symmetry	Area %
1	21.2	32978.1	997.2	0.4995	0.695	48.907
2	22.573	34452.4	913.6	0.5653	0.654	51.093

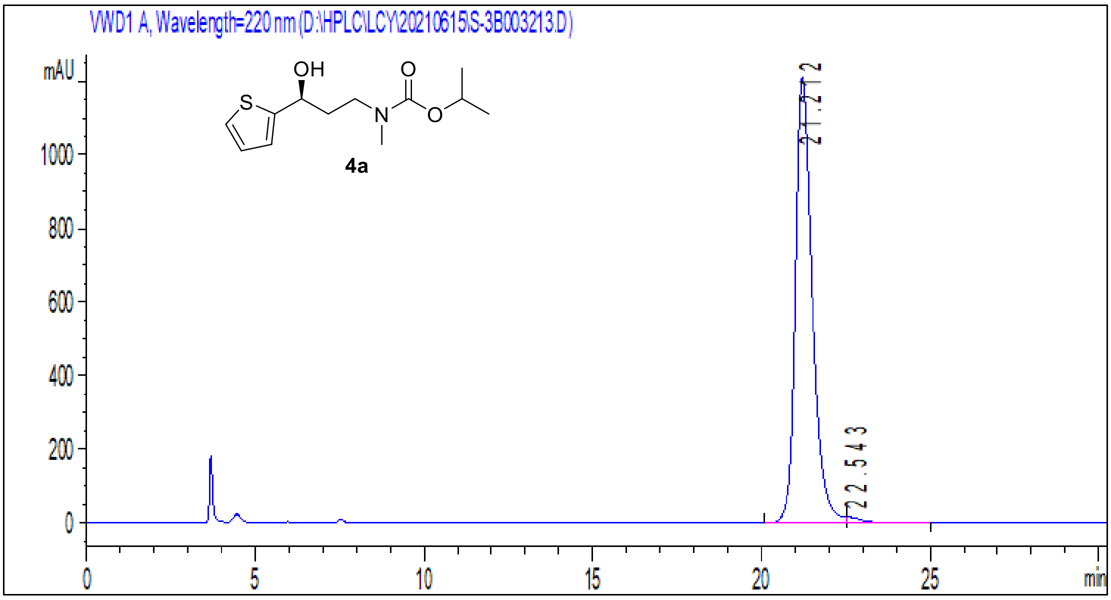

#	Time	Area	Height	Width	Symmetry	Area %
1	21.212	41994.7	1213.9	0.5766	0.602	98.811
2	22.543	505.3	15	0.5626	0	1.189

Supplementary Figure 235. HPLC spectra of **4a**

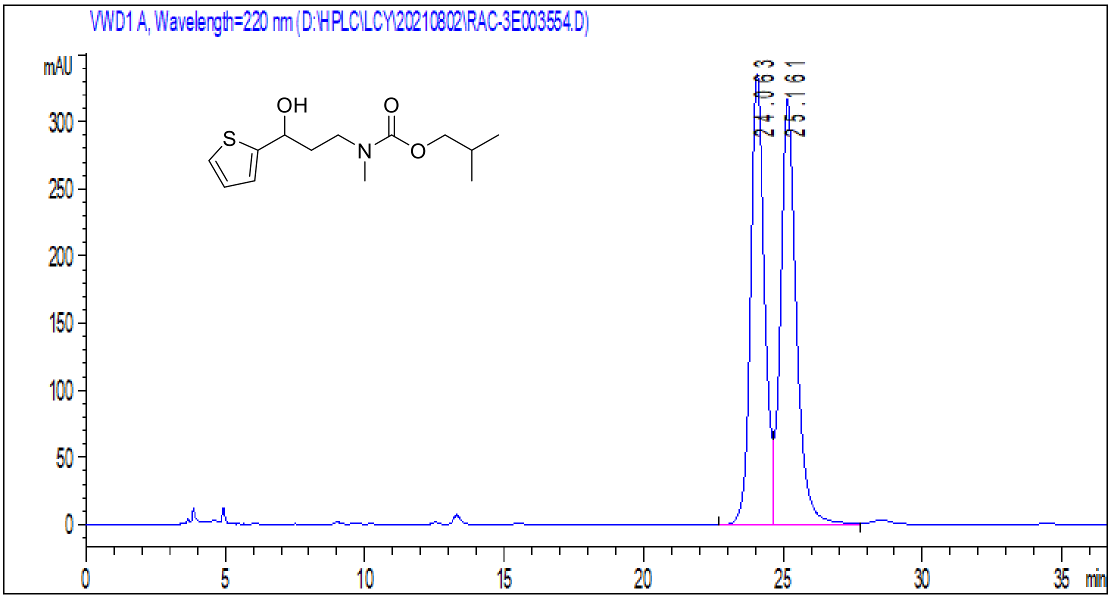

#	Time	Area	Height	Width	Symmetry	Area %
1	24.063	11671.7	334.9	0.5261	0.82	48.284
2	25.161	12501.3	317	0.5884	0.745	51.716

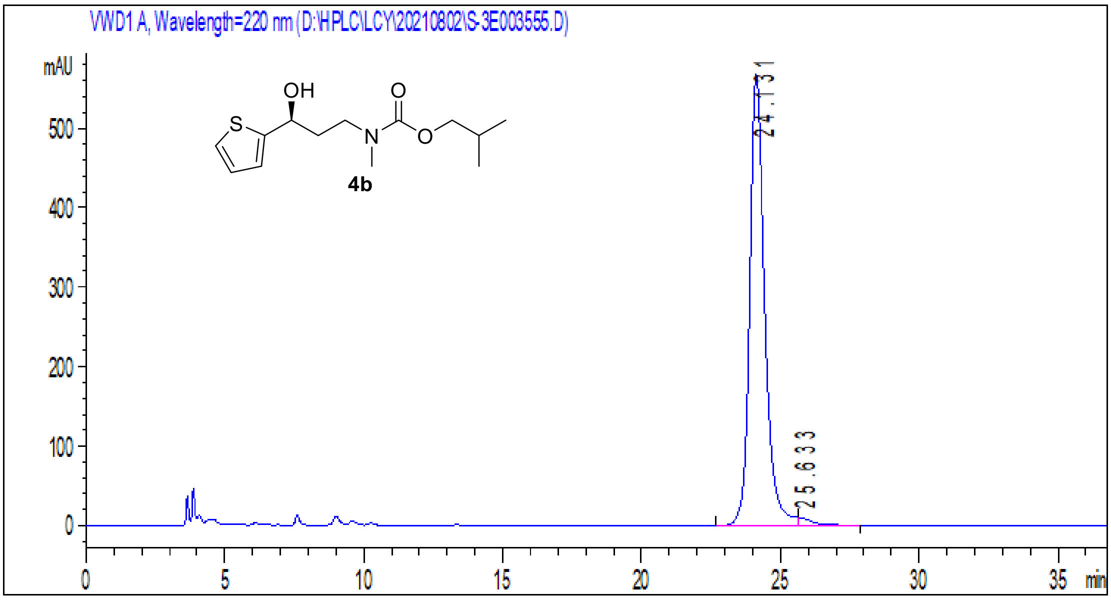

#	Time	Area	Height	Width	Symmetry	Area %
1	24.131	21245.8	567.2	0.6243	0.732	98.489
2	25.633	325.9	9.6	0.5655	0	1.511

Supplementary Figure 236. HPLC spectra of **4b**

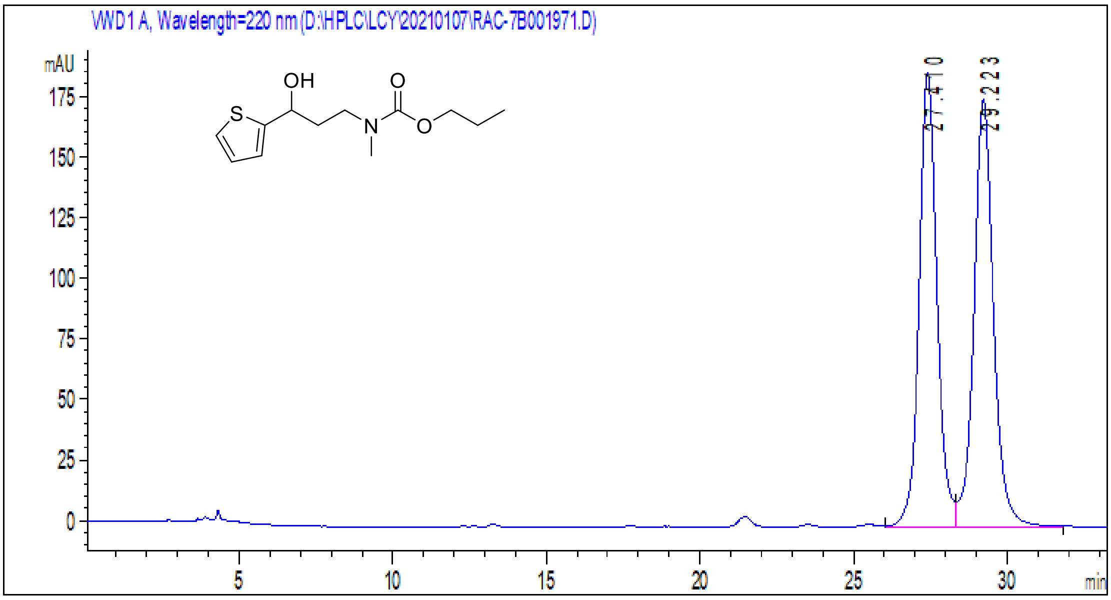

#	Time	Area	Height	Width	Symmetry	Area %
1	27.41	7511.2	187	0.6087	0.874	49.249
2	29.223	7740.2	175.7	0.6626	0.867	50.751

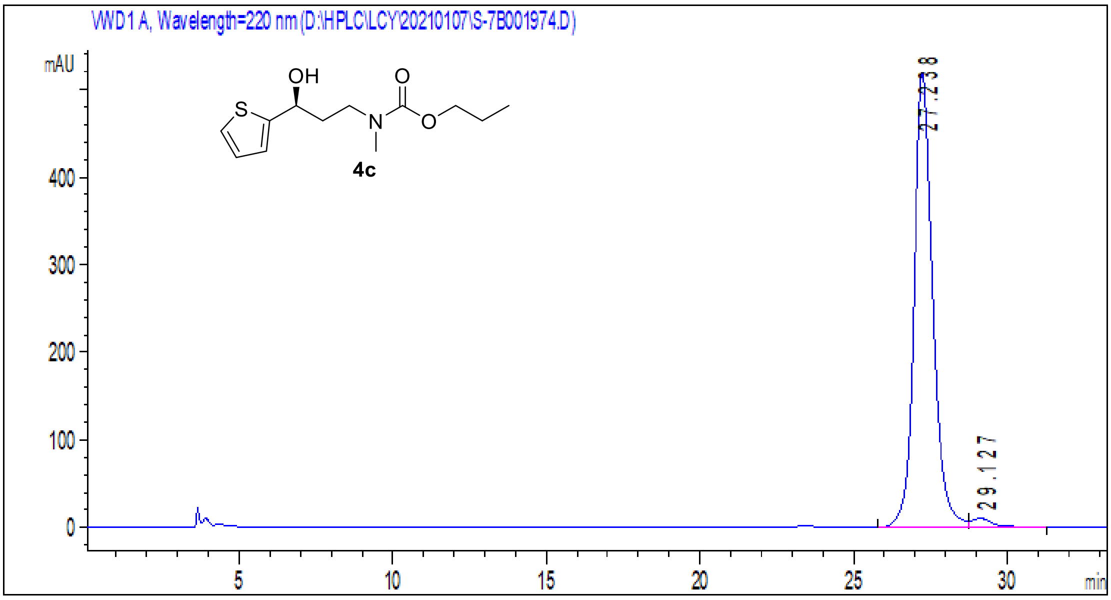

#	Time	Area	Height	Width	Symmetry	Area %
1	27.238	21785.5	518.5	0.6302	0.76	97.986
2	29.127	447.8	9.4	0.6446	0.691	2.014

Supplementary Figure 237. HPLC spectra of **4c**

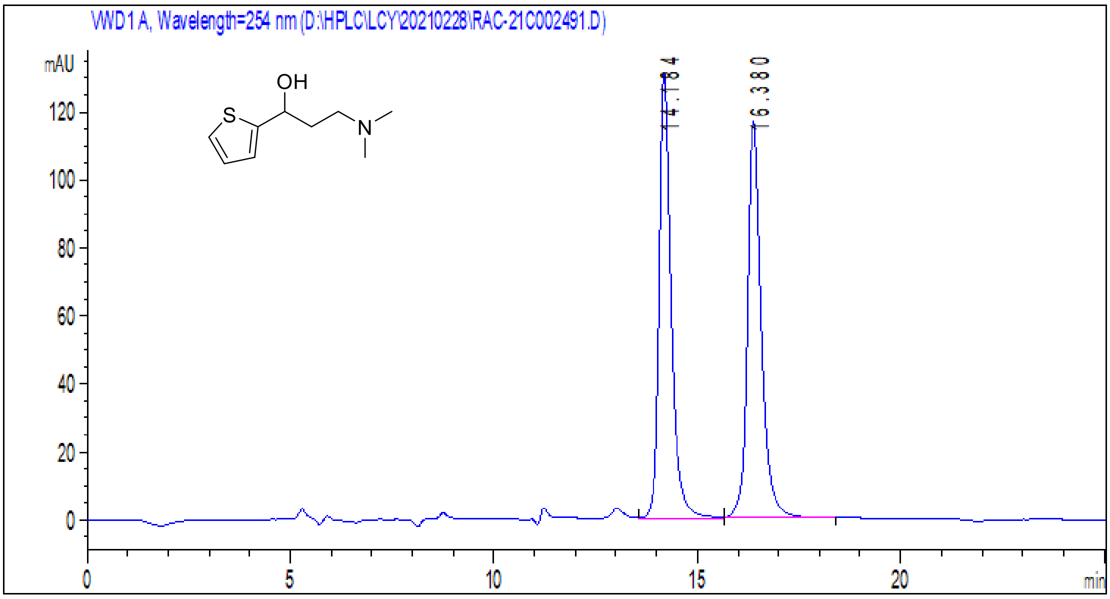

#	Time	Area	Height	Width	Symmetry	Area %
1	14.184	2697.1	131.2	0.3099	0.653	49.427
2	16.38	2759.7	116.8	0.3516	0.713	50.573

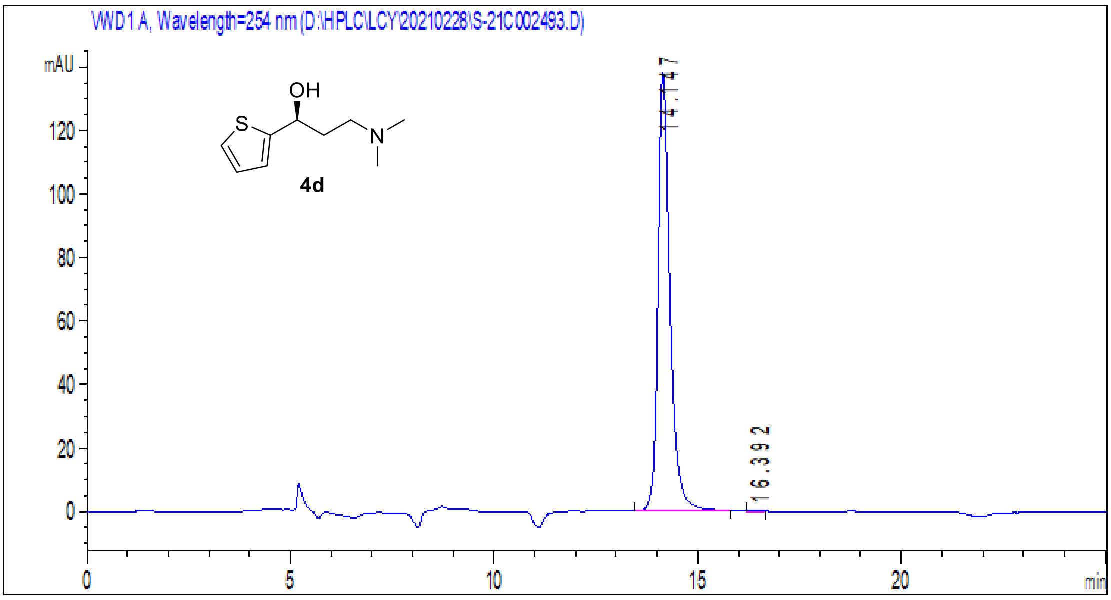

#	Time	Area	Height	Width	Symmetry	Area %
1	14.147	2831.1	138.4	0.3106	0	99.720
2	16.392	7.9	4.1E-1	0.3197	0.85	0.280

Supplementary Figure 238. HPLC spectra of **4d**

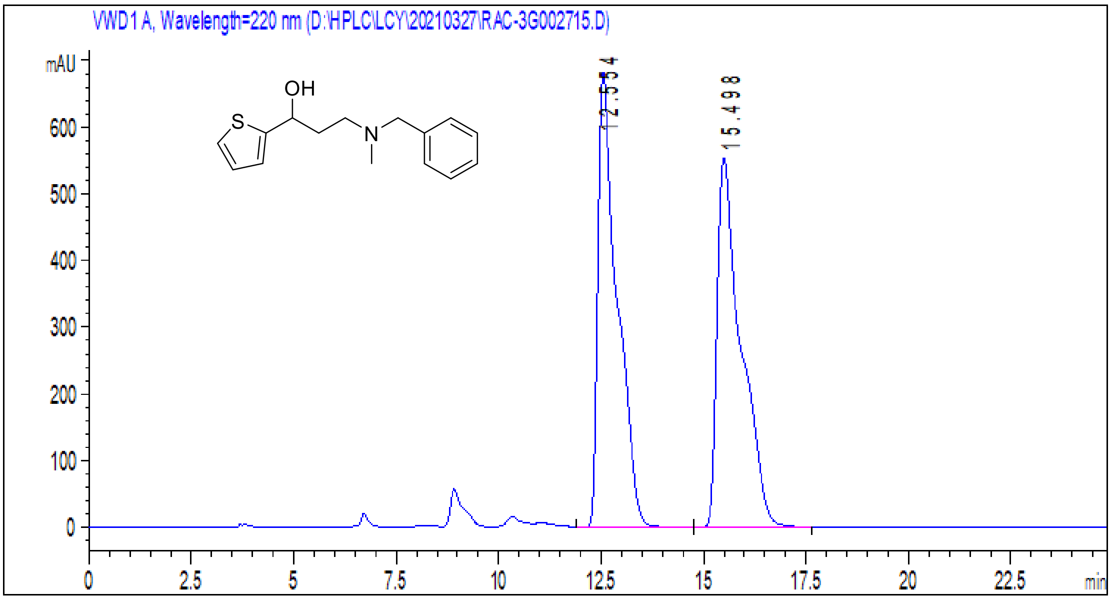

#	Time	Area	Height	Width	Symmetry	Area %
1	12.554	22301.1	680.6	0.4581	0.349	49.854
2	15.498	22431.5	553	0.5724	0.358	50.146

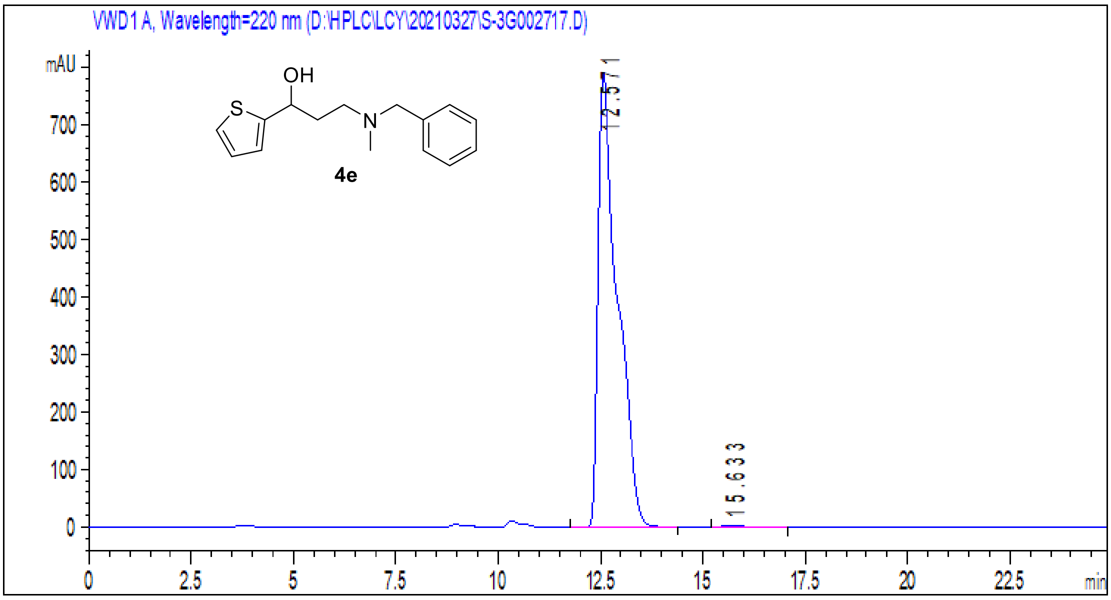

#	Time	Area	Height	Width	Symmetry	Area %
1	12.571	25949.7	791.1	0.4585	0.342	99.492
2	15.633	132.6	3.5	0.4996	0.406	0.508

Supplementary Figure 239. HPLC spectra of **4e**

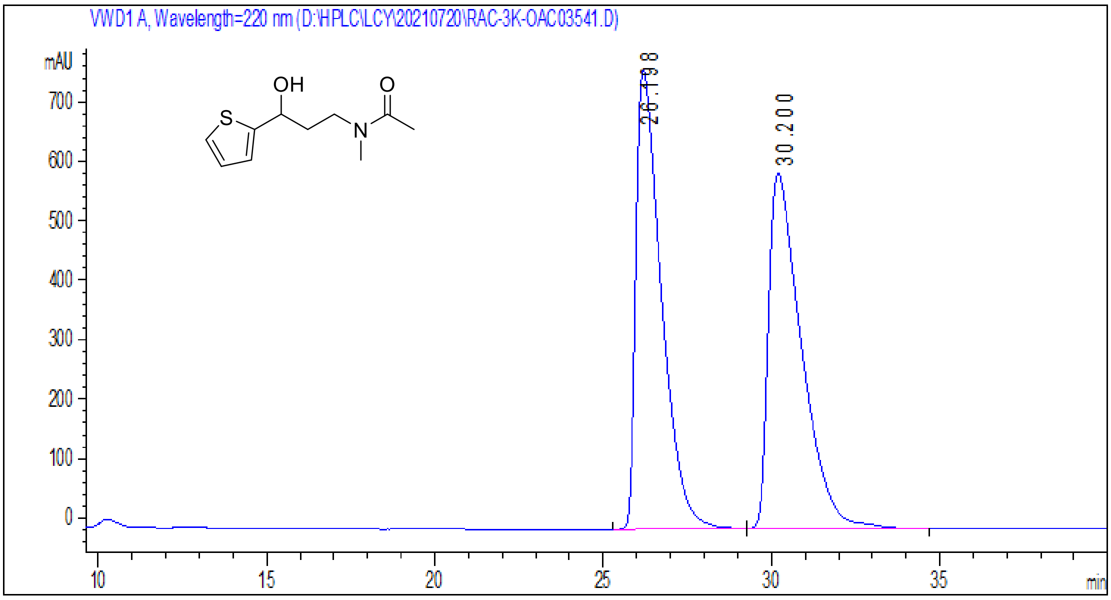

#	Time	Area	Height	Width	Symmetry	Area %
1	26.198	40339.5	771.5	0.7805	0.384	49.871
2	30.2	40549	597.1	0.995	0.379	50.129

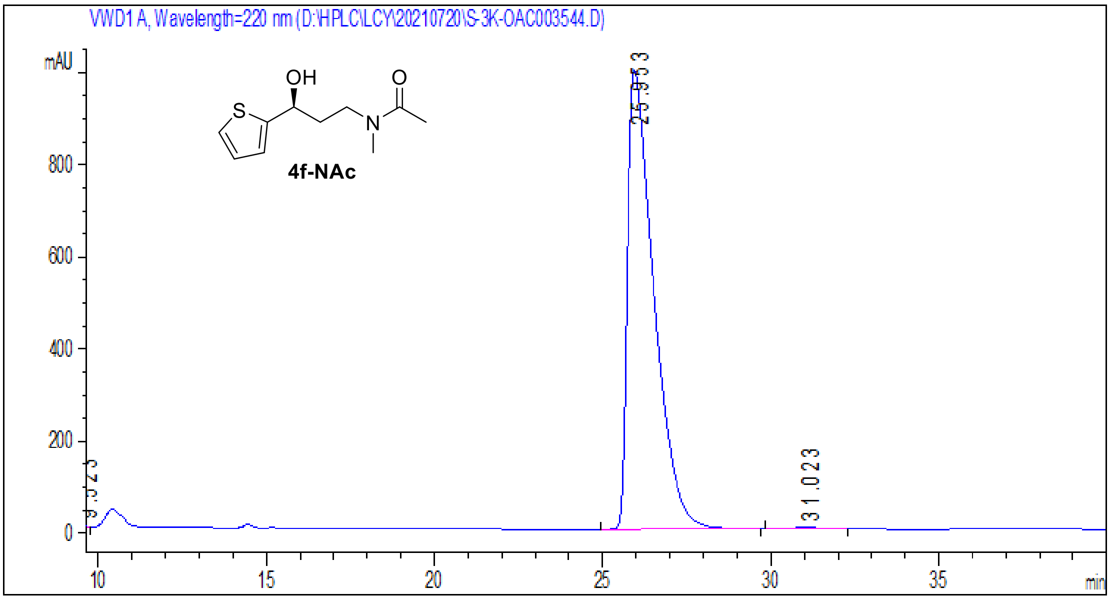

#	Time	Area	Height	Width	Symmetry	Area %
1	25.953	54270.1	996.1	0.8004	0.342	99.588
2	31.023	224.7	3.5	0.7792	0.996	0.412

Supplementary Figure 240. HPLC spectra of **4f-NAc**

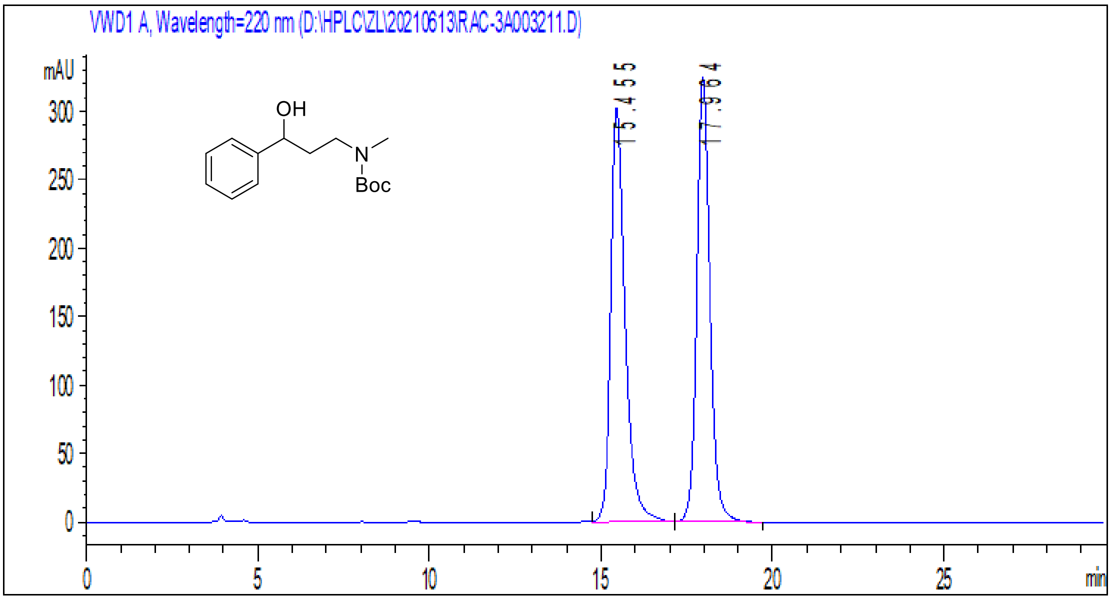

#	Time	Area	Height	Width	Symmetry	Area %
1	15.455	8758.8	303	0.4306	0.648	50.365
2	17.964	8631.8	324.8	0.4063	0.785	49.635

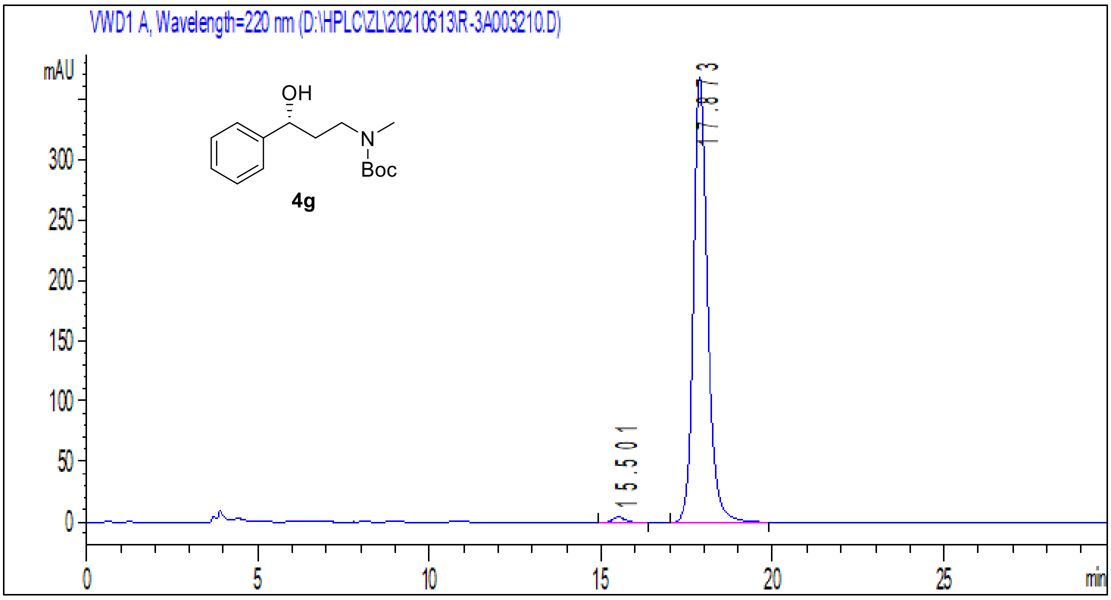

#	Time	Area	Height	Width	Symmetry	Area %
1	15.501	105.9	4.3	0.3702	0.787	1.003
2	17.873	10462.1	368.7	0.4302	0.738	98.997

Supplementary Figure 241. HPLC spectra of **4g**

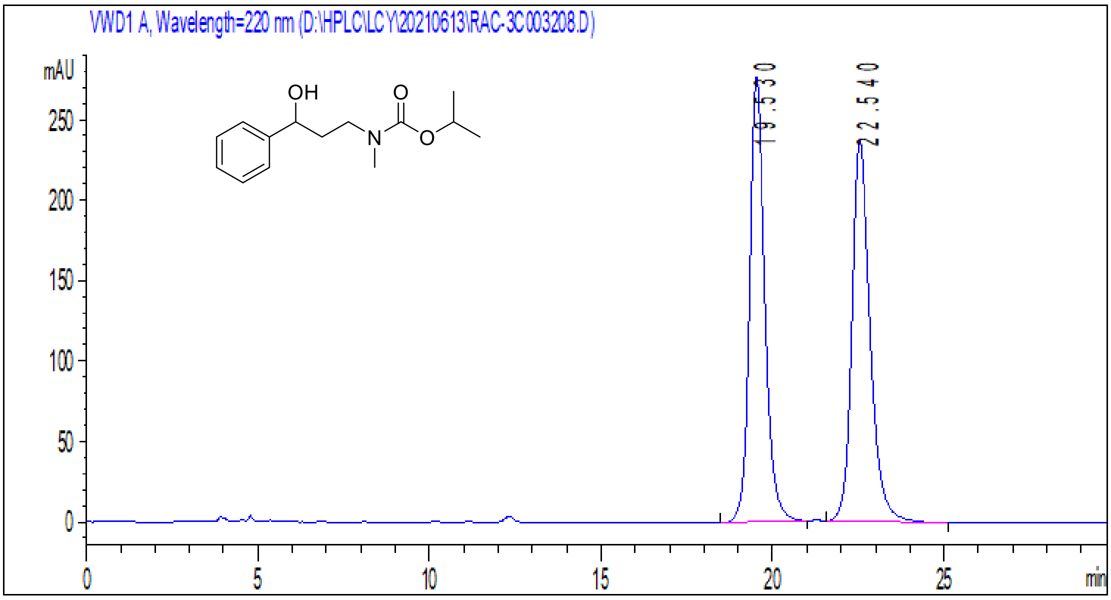

#	Time	Area	Height	Width	Symmetry	Area %
1	19.53	8510.6	276.4	0.4685	0.777	49.469
2	22.54	8693.3	236.6	0.5585	0.71	50.531

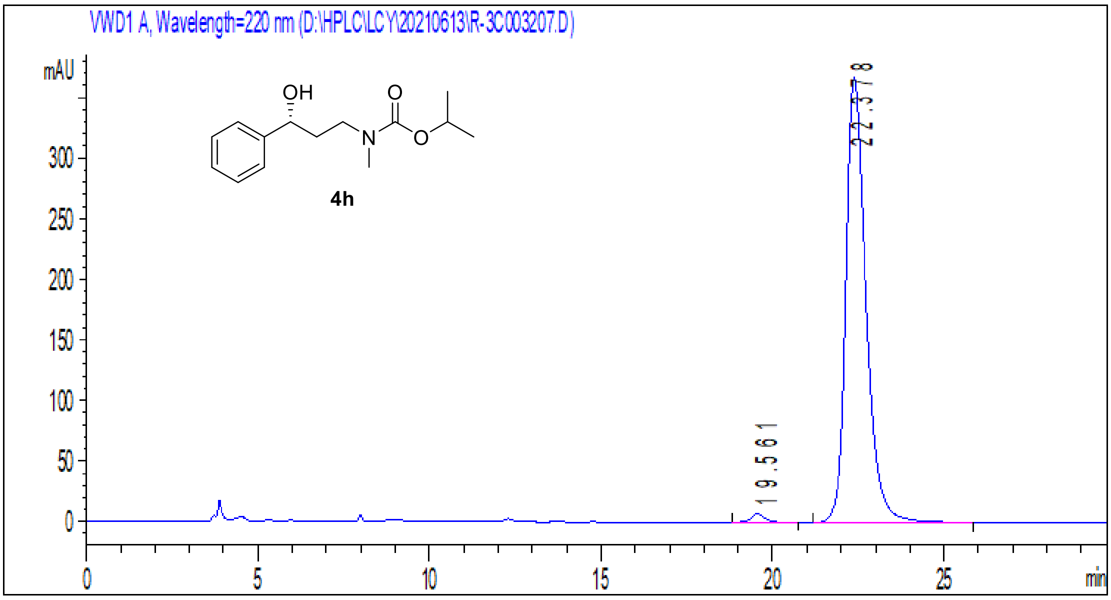

#	Time	Area	Height	Width	Symmetry	Area %
1	19.561	180.8	6.7	0.4018	0.863	1.203
2	22.378	14844.7	367.2	0.6174	0.653	98.797

Supplementary Figure 242. HPLC spectra of **4h**

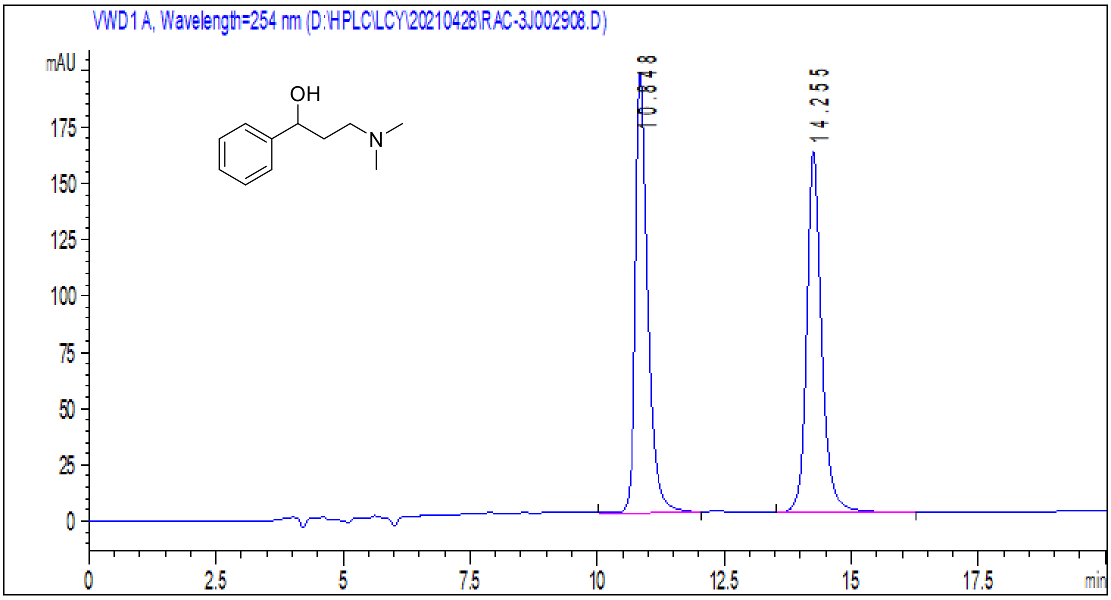

#	Time	Area	Height	Width	Symmetry	Area %
1	10.848	3320.1	195.3	0.2509	0.59	50.148
2	14.255	3300.5	160.5	0.304	0.767	49.852

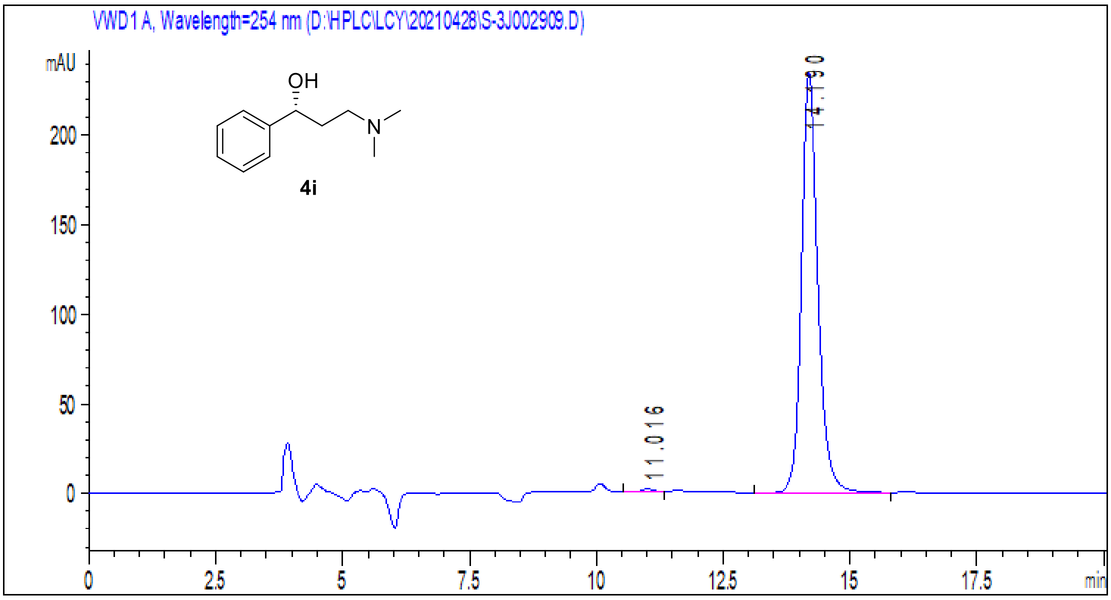

#	Time	Area	Height	Width	Symmetry	Area %
1	11.016	35.8	1.9	0.322	1.23	0.672
2	14.19	5294.1	234.9	0.3446	0.77	99.328

Supplementary Figure 243. HPLC spectra of **4i**

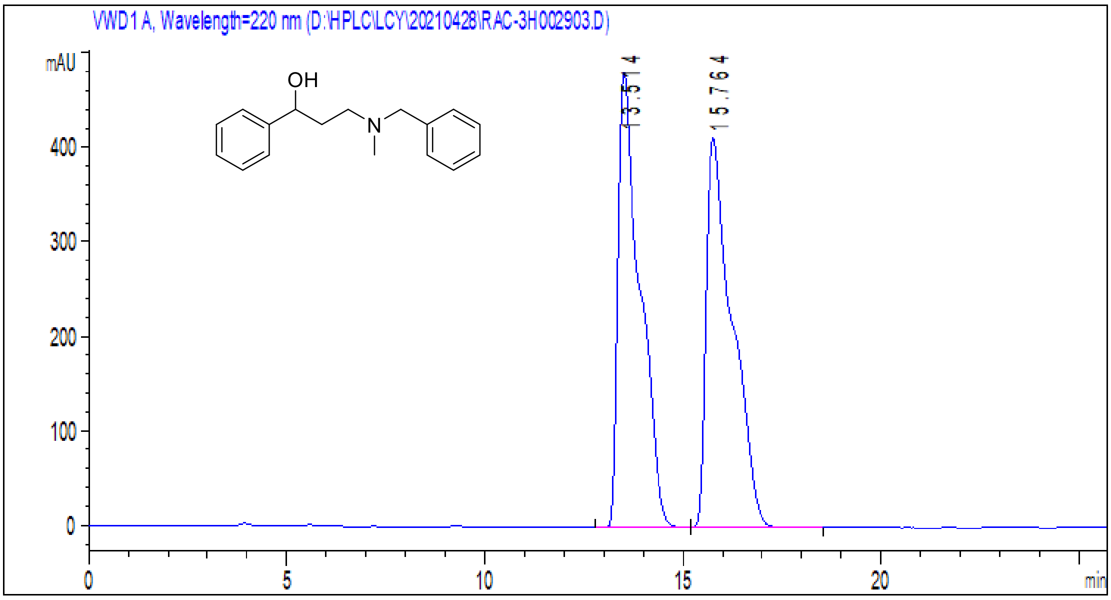

#	Time	Area	Height	Width	Symmetry	Area %
1	13.514	18536.1	481.4	0.5425	0.347	49.584
2	15.764	18846.9	411.4	0.6453	0.34	50.416

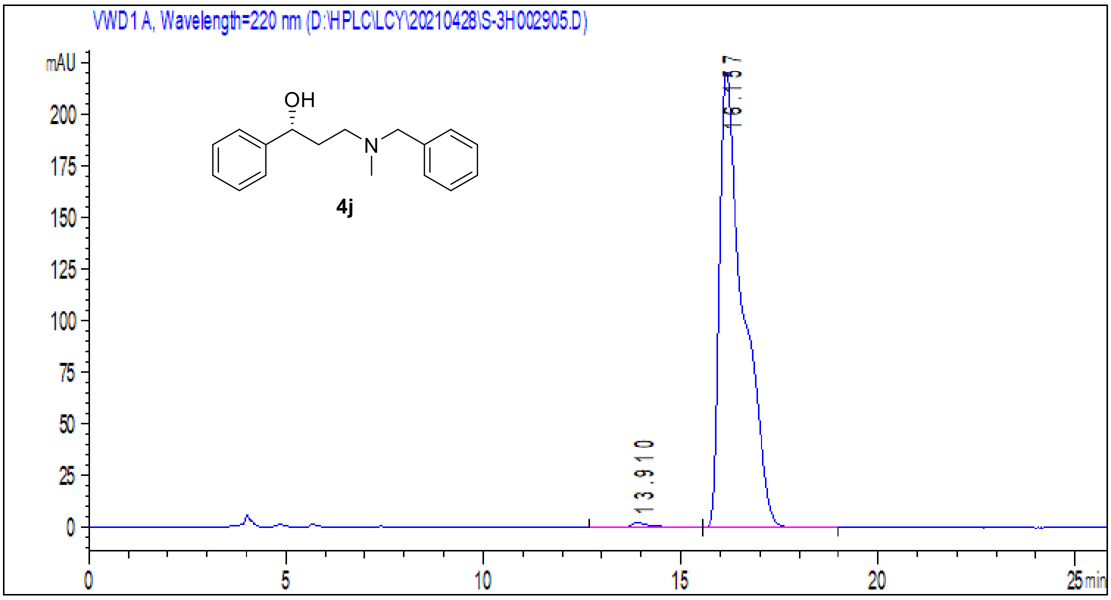

#	Time	Area	Height	Width	Symmetry	Area %
1	13.91	87.3	2.4	0.5092	0.588	0.937
2	16.157	9228.4	220.5	0.5932	0.367	99.063

Supplementary Figure 244. HPLC spectra of **4j**

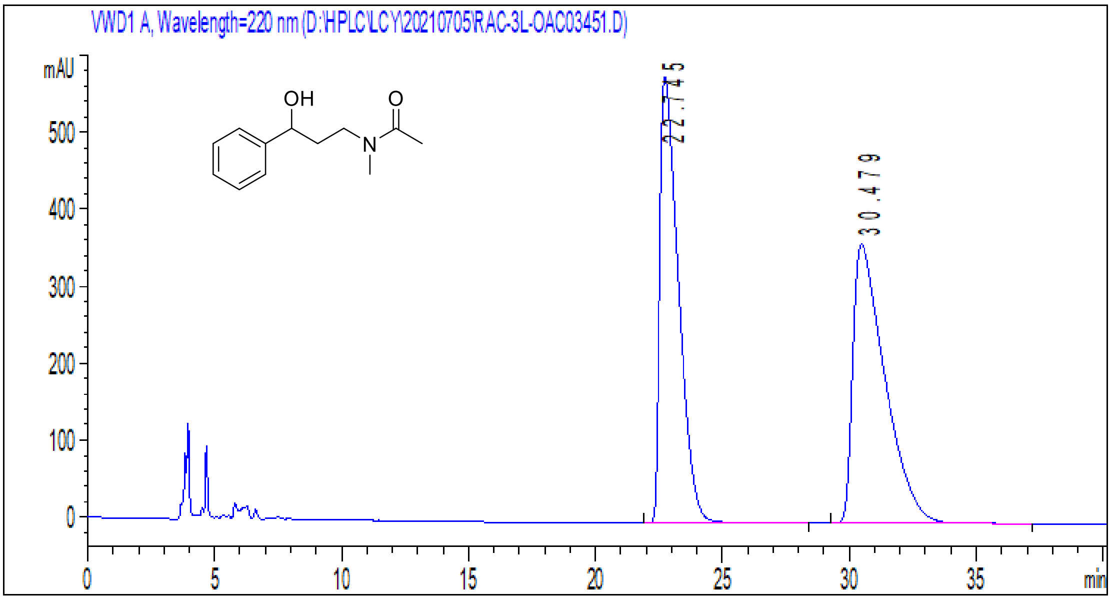

#	Time	Area	Height	Width	Symmetry	Area %
1	22.745	29492	579.7	0.7743	0.366	48.128
2	30.479	31785.7	362.2	1.3008	0.343	51.872

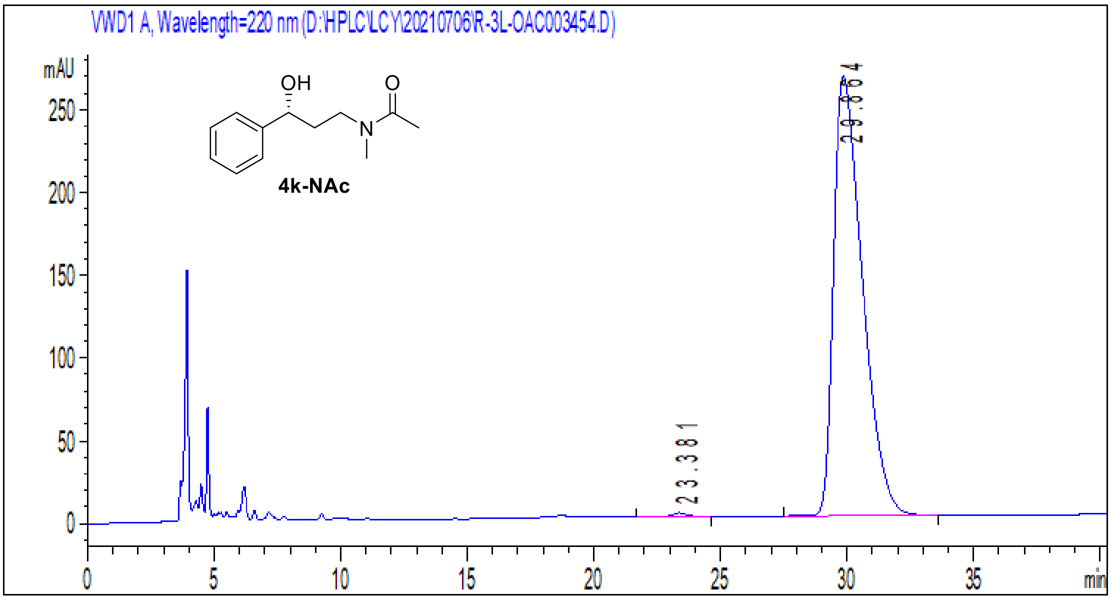

#	Time	Area	Height	Width	Symmetry	Area %
1	23.381	103.9	2.4	0.6346	0.996	0.494
2	29.864	20933.7	265.4	1.206	0.494	99.506

Supplementary Figure 245. HPLC spectra of **4k**

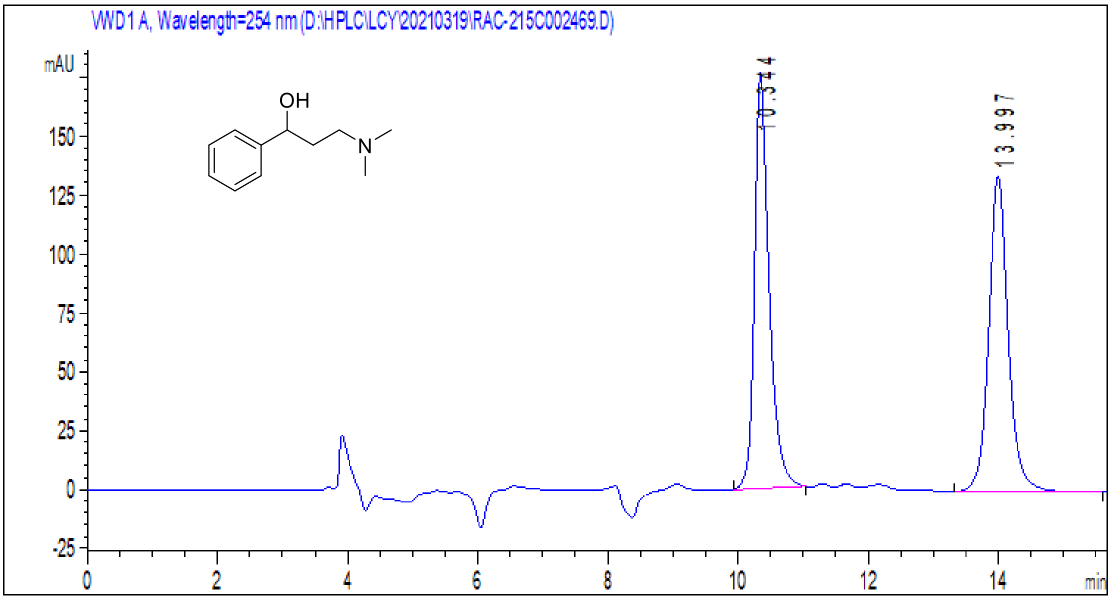

#	Time	Area	Height	Width	Symmetry	Area %
1	10.344	2760.4	176.9	0.26	0.594	49.720
2	13.997	2791.5	133.6	0.3137	0.883	50.280

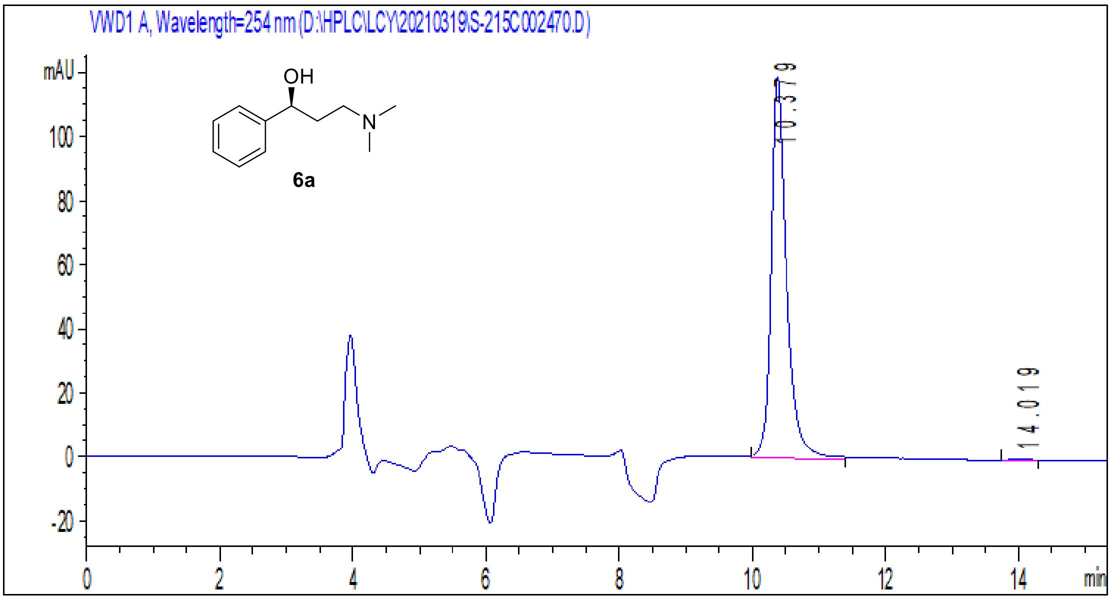

#	Time	Area	Height	Width	Symmetry	Area %
1	10.379	1902.4	119.3	0.2658	0.588	99.529
2	14.019	9	4.8E-1	0.3113	1.15	0.471

Supplementary Figure 246. HPLC spectra of **6a**

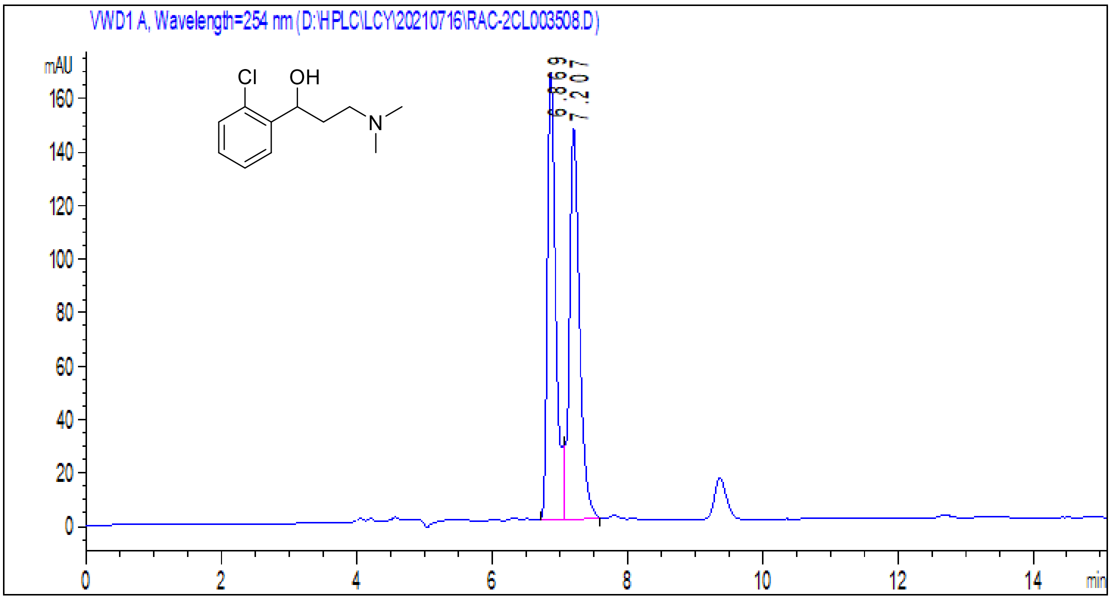

#	Time	Area	Height	Width	Symmetry	Area %
1	6.869	1461.2	167.7	0.1452	0	48.979
2	7.207	1522.1	146.3	0.1734	0.696	51.021

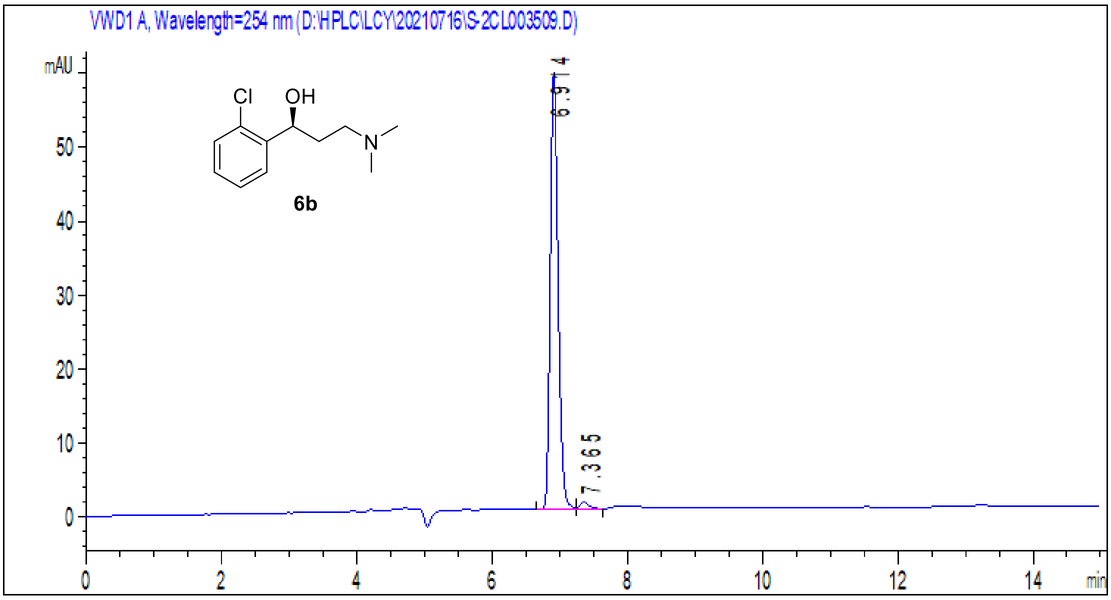

#	Time	Area	Height	Width	Symmetry	Area %
1	6.914	486.6	59	0.1282	0.758	98.112
2	7.365	9.4	9.3E-1	0.1496	0.796	1.888

Supplementary Figure 247. HPLC spectra of **6b**

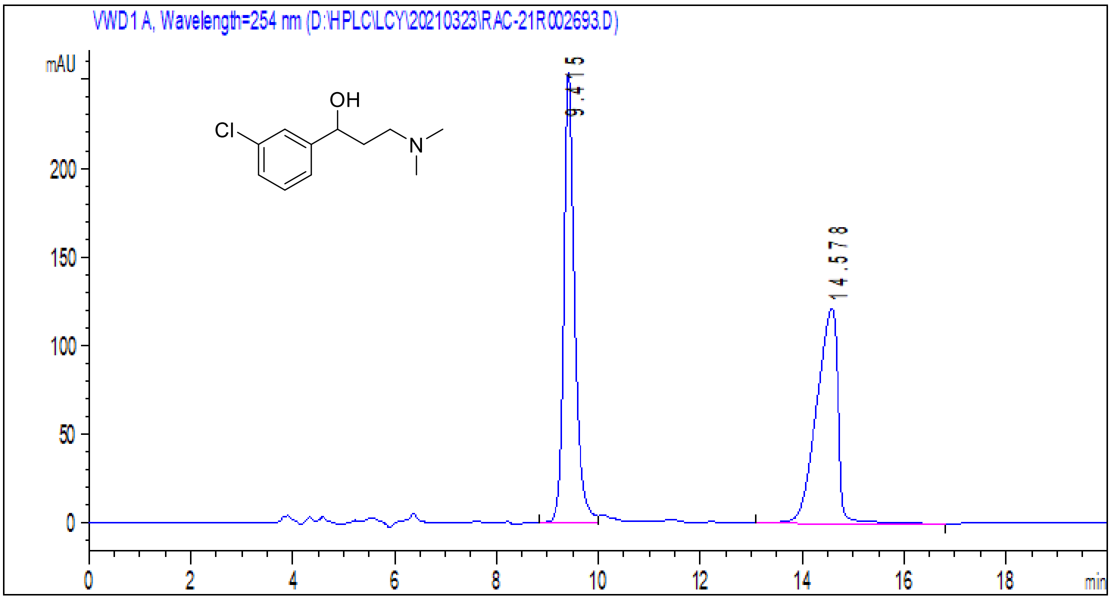

#	Time	Area	Height	Width	Symmetry	Area %
1	9.415	3683.9	253.1	0.2164	0.689	50.602
2	14.578	3596.2	121.2	0.4627	2.091	49.398

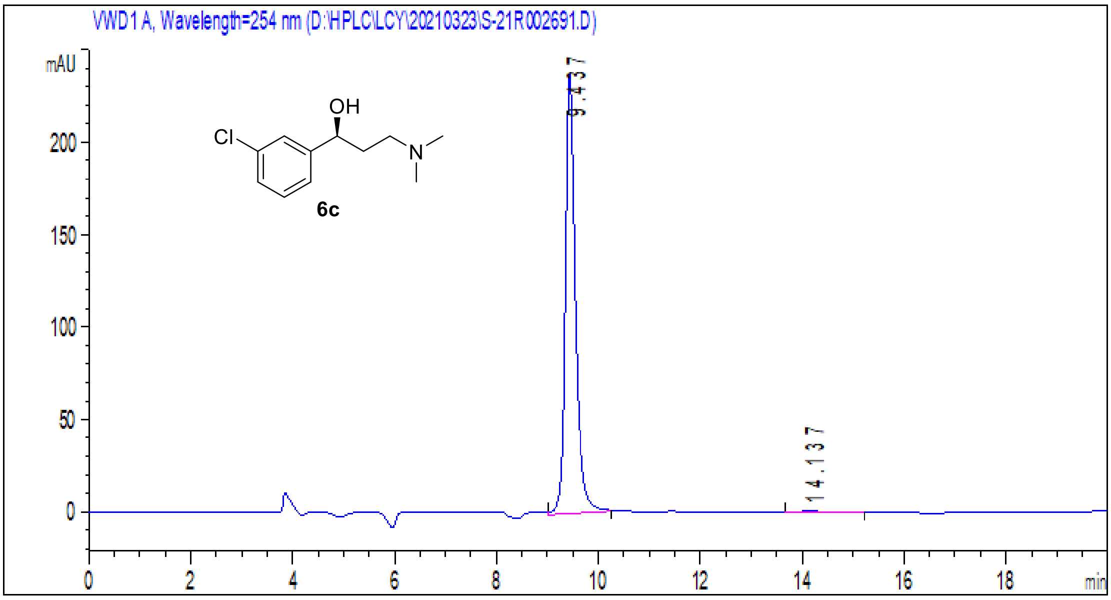

#	Time	Area	Height	Width	Symmetry	Area %
1	9.437	3246.4	239.4	0.226	0.649	99.406
2	14.137	19.4	8.6E-1	0.3456	0.706	0.594

Supplementary Figure 248. HPLC spectra of **6c**

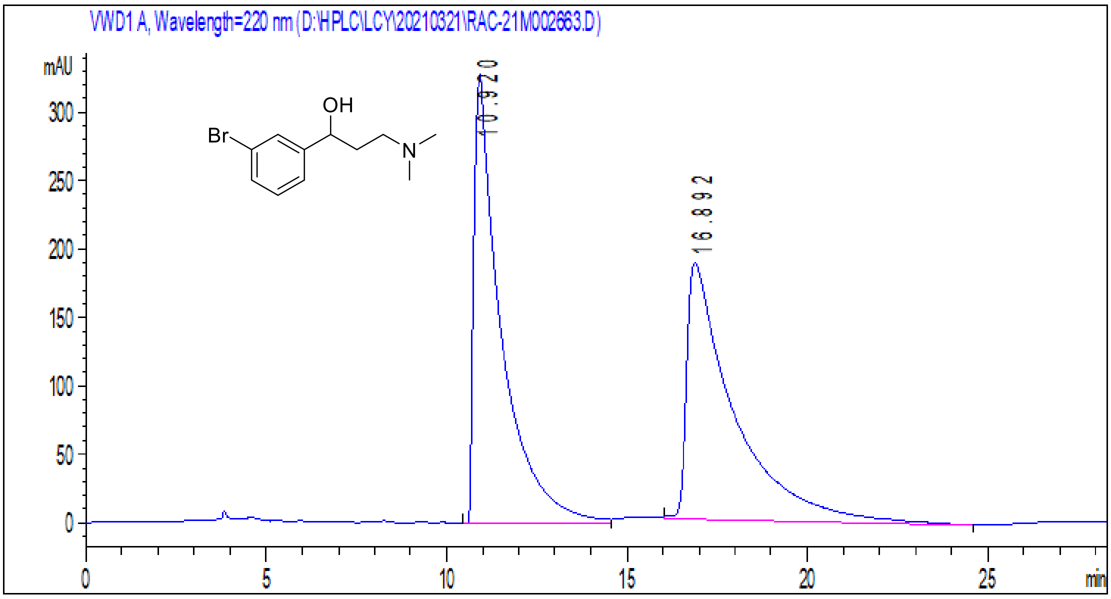

#	Time	Area	Height	Width	Symmetry	Area %
1	10.92	17242.9	327.7	0.7188	0.253	49.063
2	16.894	17901.2	190.4	1.2544	0.207	50.937

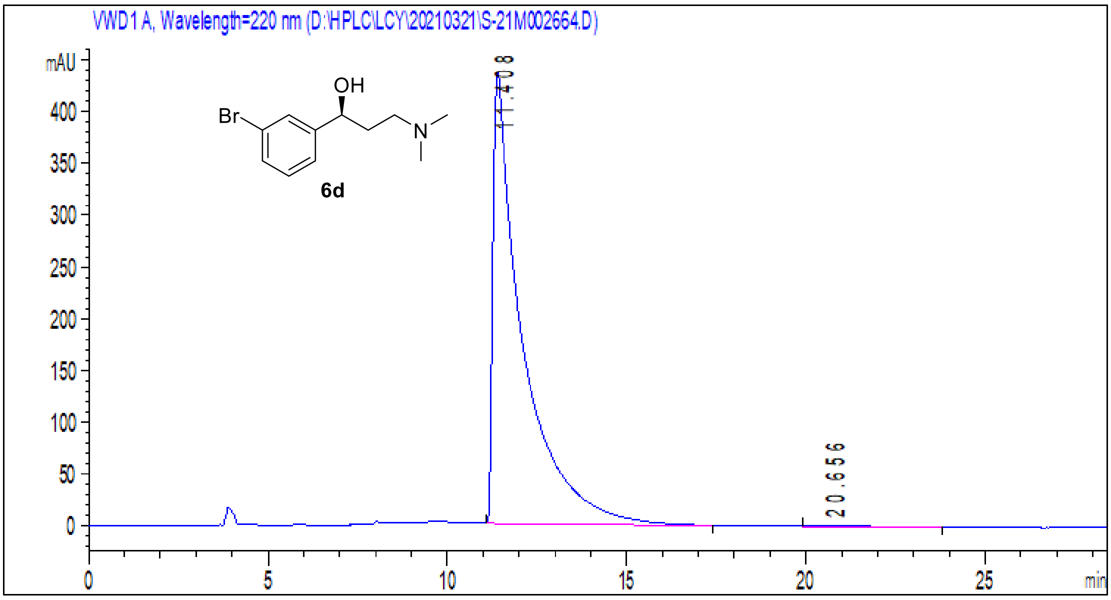

#	Time	Area	Height	Width	Symmetry	Area %
1	11.408	24805.1	435	0.7528	0.177	99.492
2	20.656	126.6	1.2	1.2913	0.366	0.508

Supplementary Figure 249. HPLC spectra of **6d**

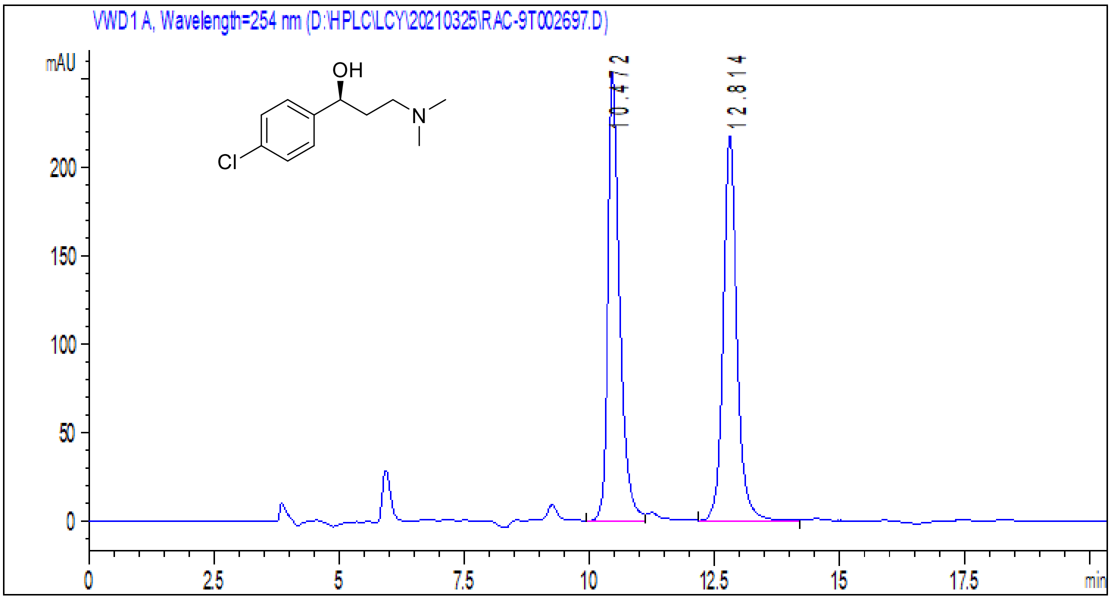

#	Time	Area	Height	Width	Symmetry	Area %
1	10.472	4153.5	253.4	0.2437	0.603	49.856
2	12.814	4177.6	217.7	0.288	0.837	50.144

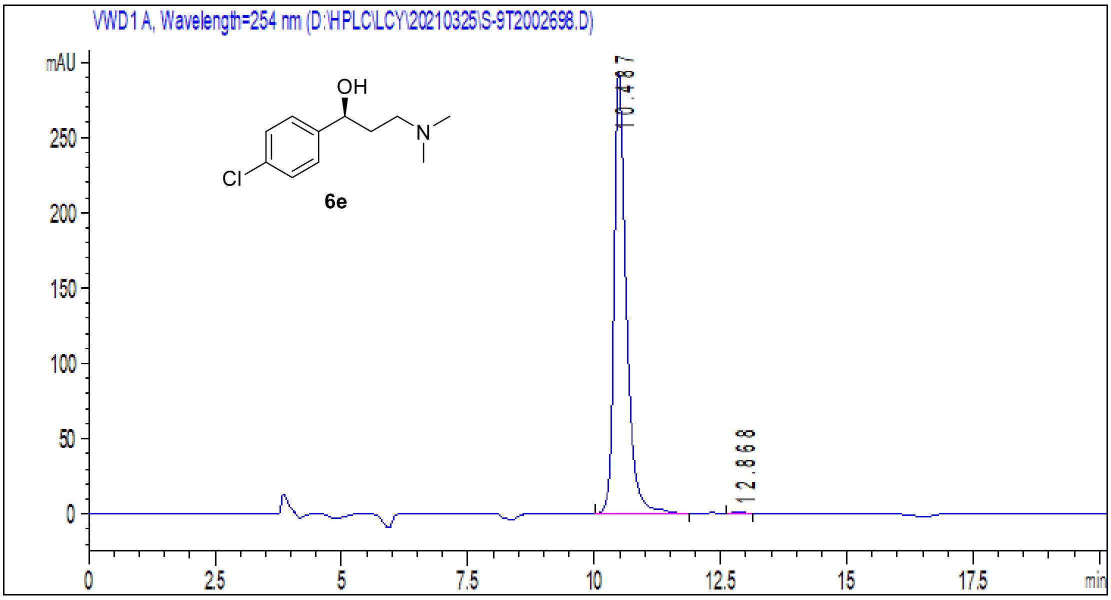

#	Time	Area	Height	Width	Symmetry	Area %
1	10.487	4893.7	293.5	0.2693	0	99.459
2	12.868	26.6	1.3	0.3353	1.22	0.541

Supplementary Figure 250. HPLC spectra of **6e**

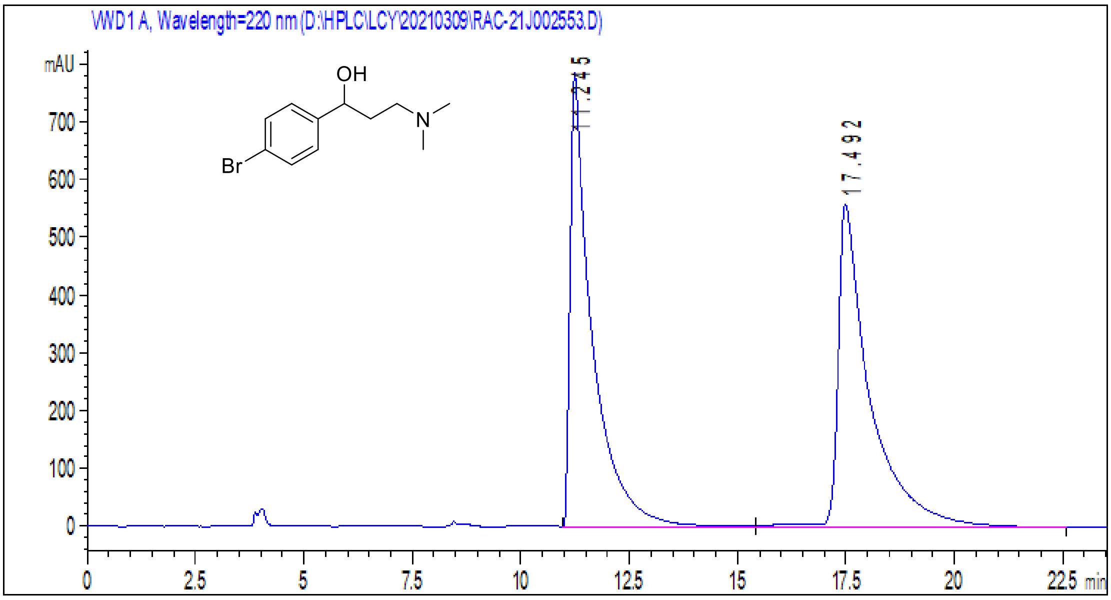

#	Time	Area	Height	Width	Symmetry	Area %
1	11.245	27675.2	785.5	0.4694	0.237	49.406
2	17.492	28340.4	559.9	0.6926	0.289	50.594

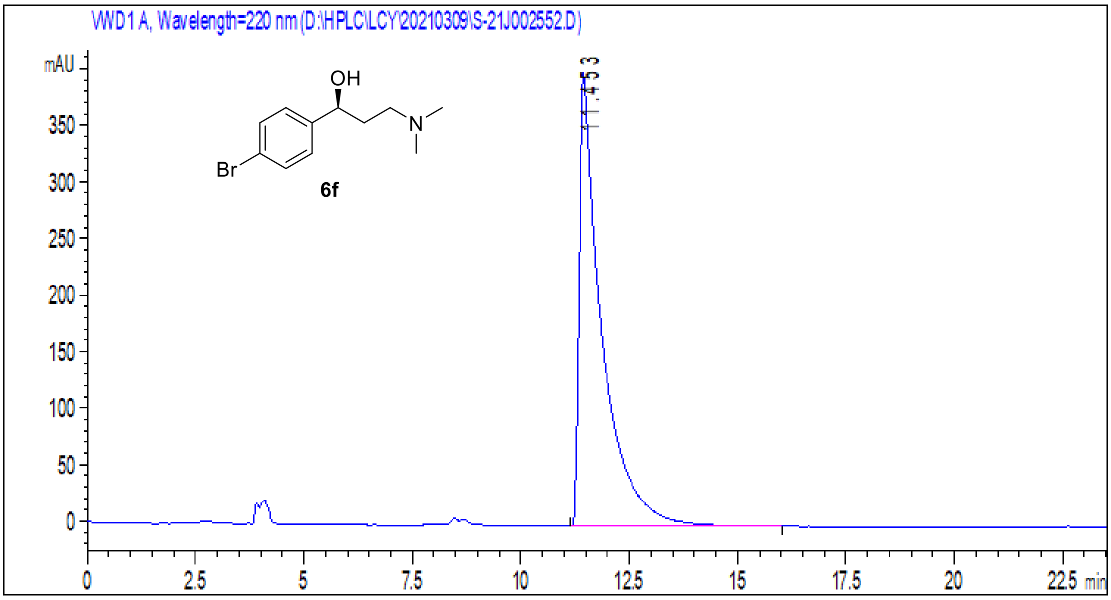

#	Time	Area	Height	Width	Symmetry	Area %
1	11.453	14407.7	400.4	0.4723	0.223	100.000

Supplementary Figure 251. HPLC spectra of **6f**

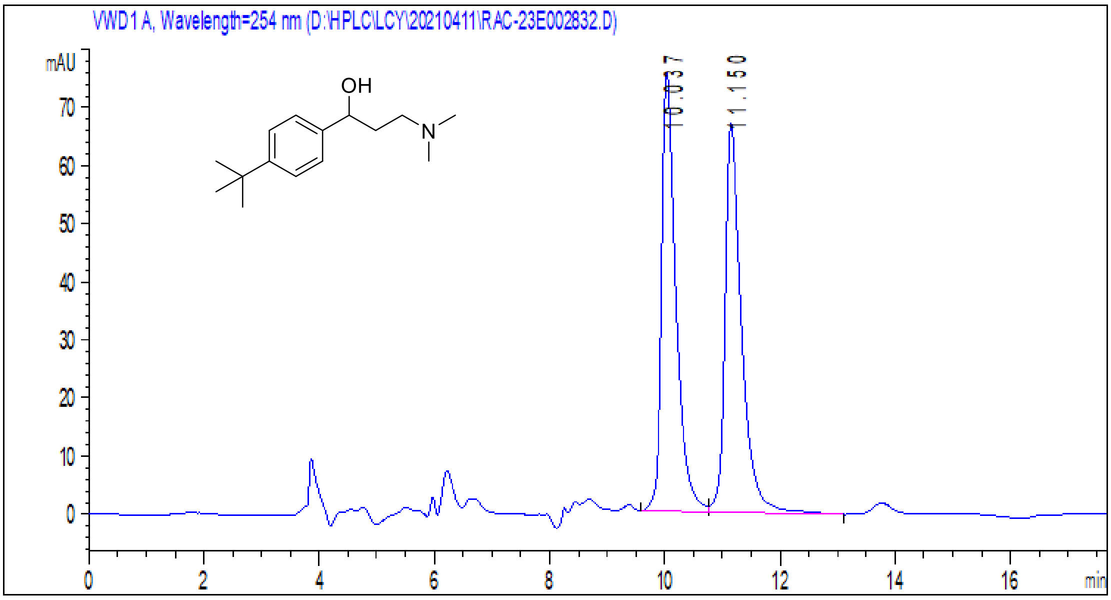

#	Time	Area	Height	Width	Symmetry	Area %
1	10.037	1303.6	75.4	0.2541	0.553	49.272
2	11.15	1342.1	66.9	0.2923	0.527	50.728

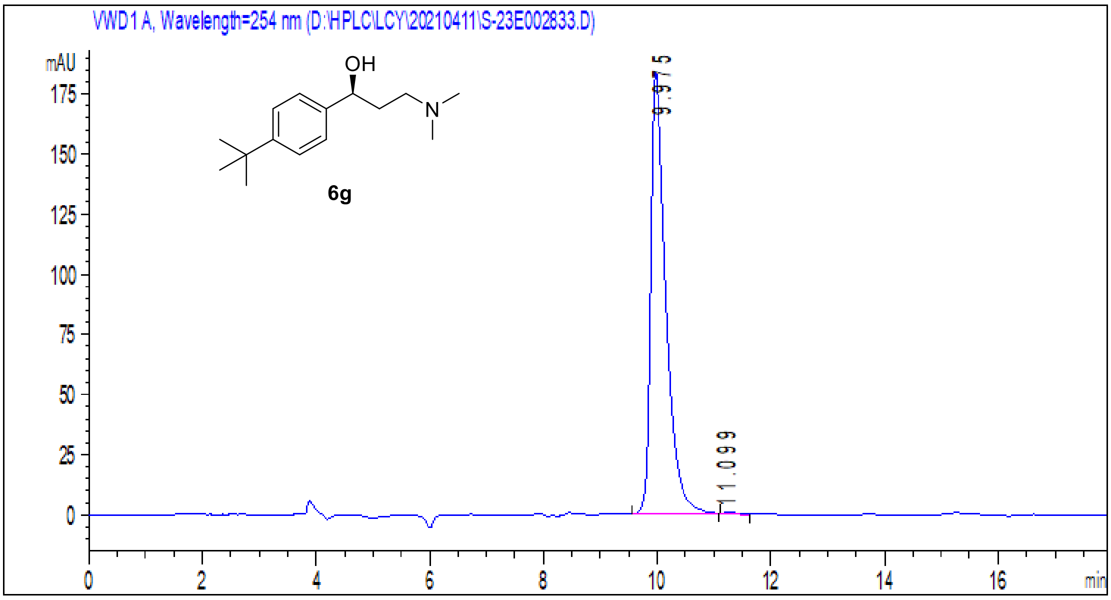

#	Time	Area	Height	Width	Symmetry	Area %
1	9.975	3381.8	184.2	0.306	0.435	99.633
2	11.099	12.5	6.8E-2	0.289	0.465	0.367

Supplementary Figure 252. HPLC spectra of **6g**

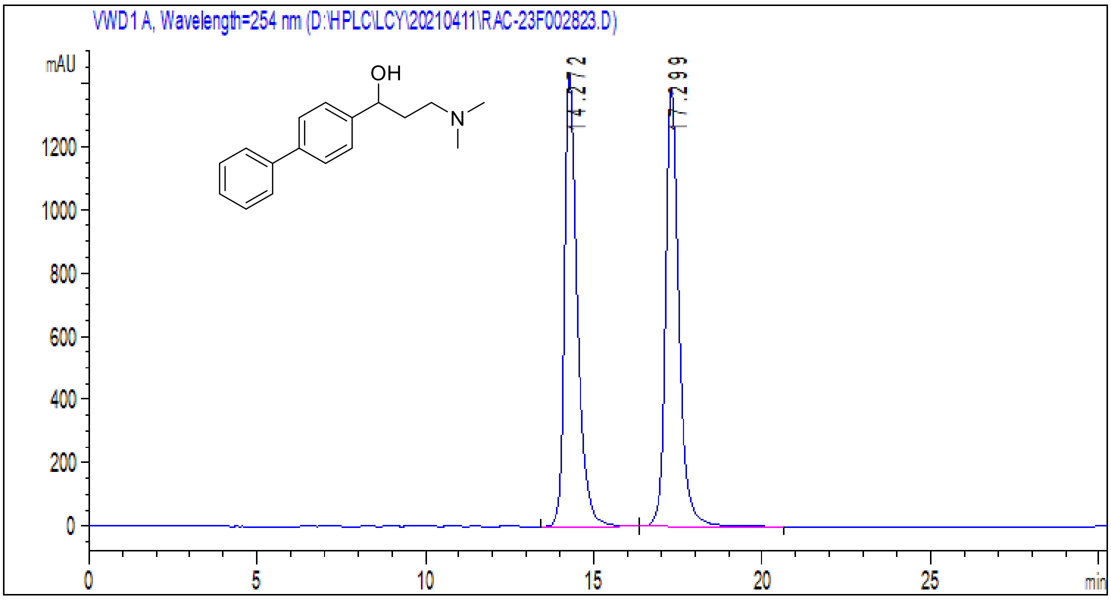

#	Time	Area	Height	Width	Symmetry	Area %
1	14.272	37626	1433.9	0.3936	0.574	49.855
2	17.299	37844.9	1385.3	0.412	0.702	50.145

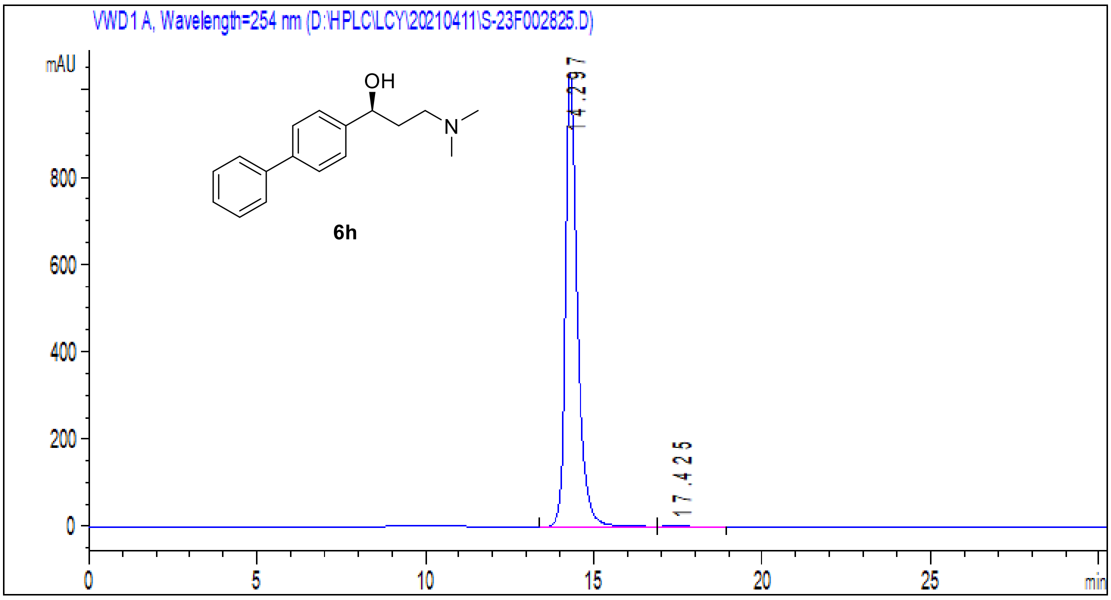

#	Time	Area	Height	Width	Symmetry	Area %
1	14.297	25443.5	1039.1	0.3673	0.61	99.520
2	17.425	122.8	4.2	0.438	0.823	0.480

Supplementary Figure 253. HPLC spectra of **6h**

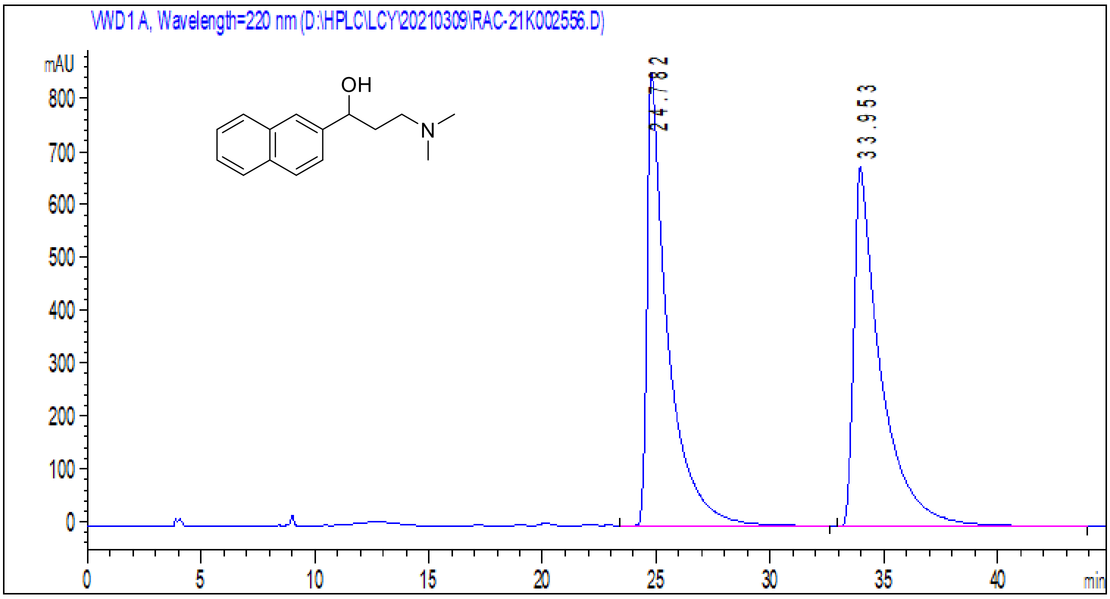

#	Time	Area	Height	Width	Symmetry	Area %
1	24.782	54205.3	857.7	0.8627	0.243	49.583
2	33.953	55117.5	678.7	1.0815	0.281	50.417

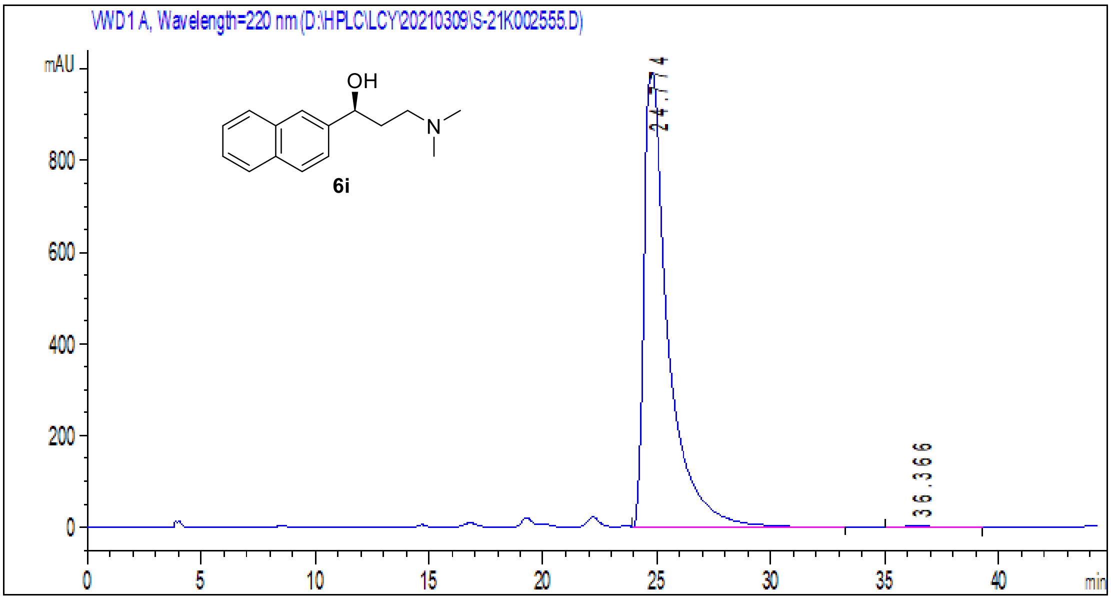

#	Time	Area	Height	Width	Symmetry	Area %
1	24.774	72518	991.5	1.0516	0.408	99.567
2	36.366	315.1	3.6	1.0711	0.614	0.433

Supplementary Figure 254. HPLC spectra of **6i**

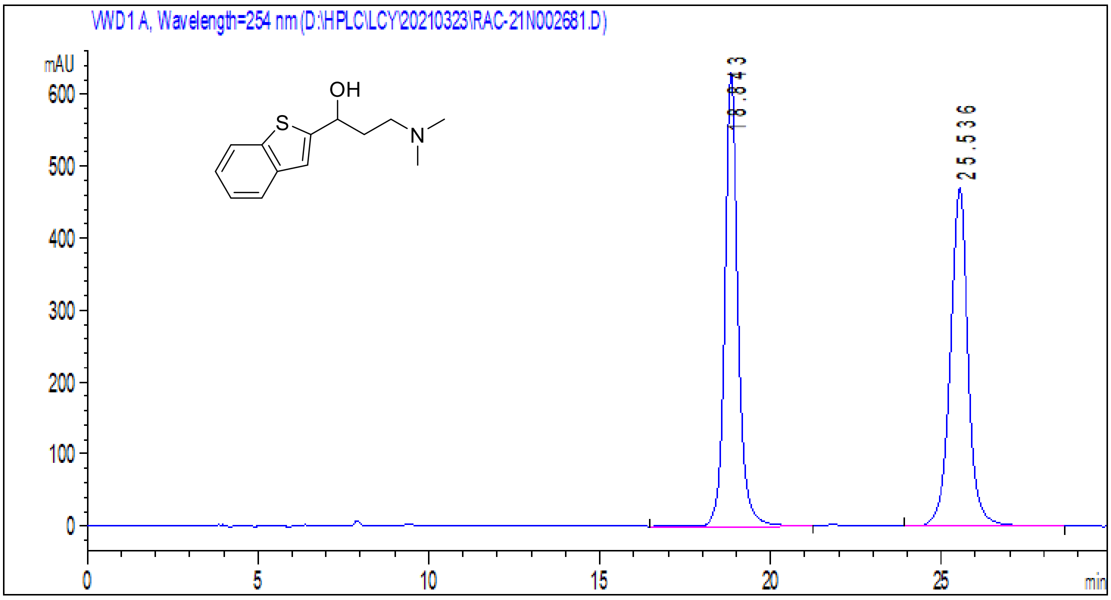

#	Time	Area	Height	Width	Symmetry	Area %
1	18.843	17117.6	630.7	0.4041	0.821	50.030
2	25.536	17096.8	469.8	0.6066	1.045	49.970

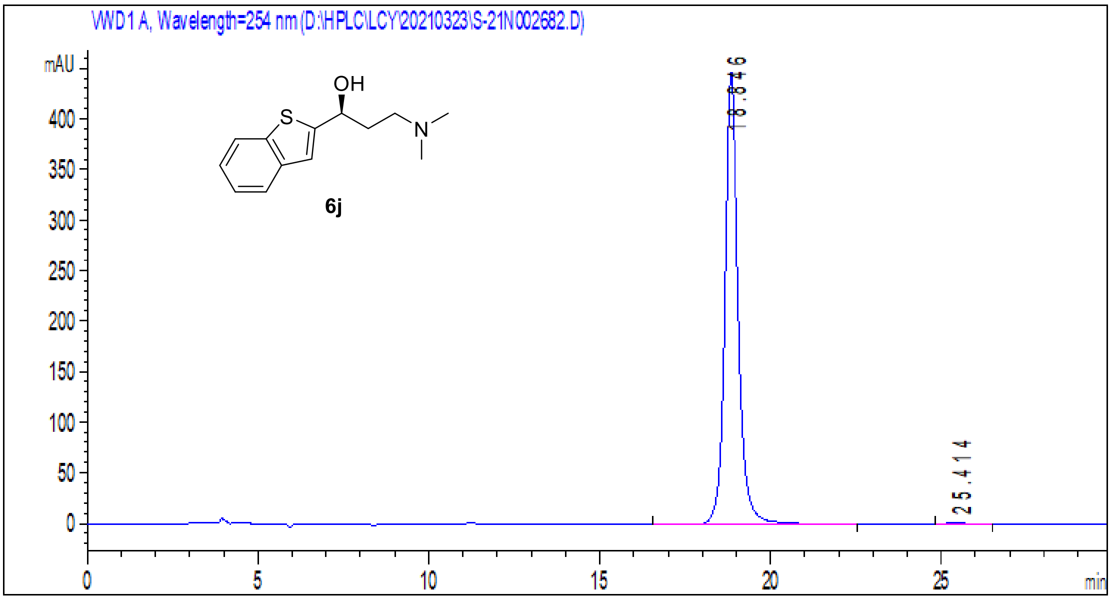

#	Time	Area	Height	Width	Symmetry	Area %
1	18.846	12109.2	446.1	0.4041	0.823	99.715
2	25.414	34.6	1	0.4092	0.843	0.285

Supplementary Figure 255. HPLC spectra of **6j**

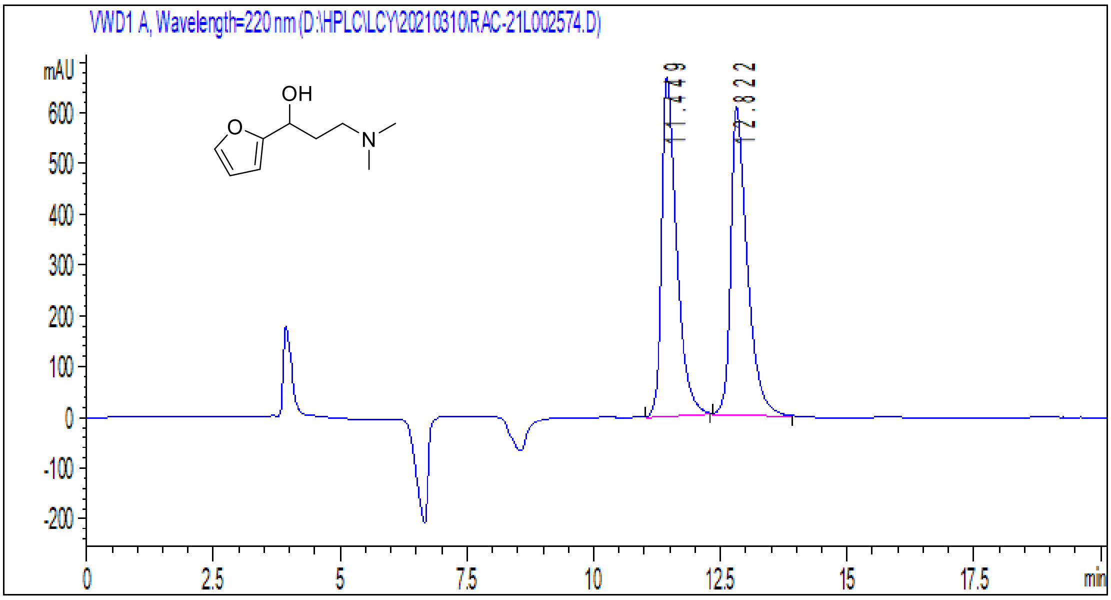

#	Time	Area	Height	Width	Symmetry	Area %
1	11.449	13627	670.1	0.3389	0.616	49.543
2	12.822	13878.7	609.1	0.3797	0.572	50.457

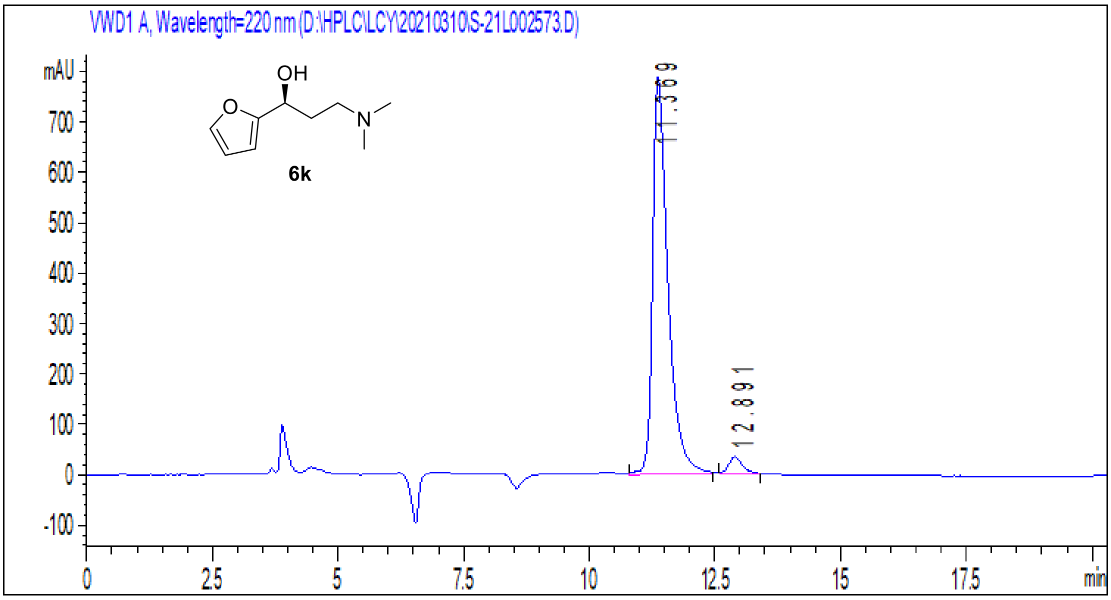

#	Time	Area	Height	Width	Symmetry	Area %
1	11.369	16820.1	789.9	0.3549	0.587	96.302
2	12.891	646	33.5	0.3214	0.844	3.698

Supplementary Figure 256. HPLC spectra of **6k**

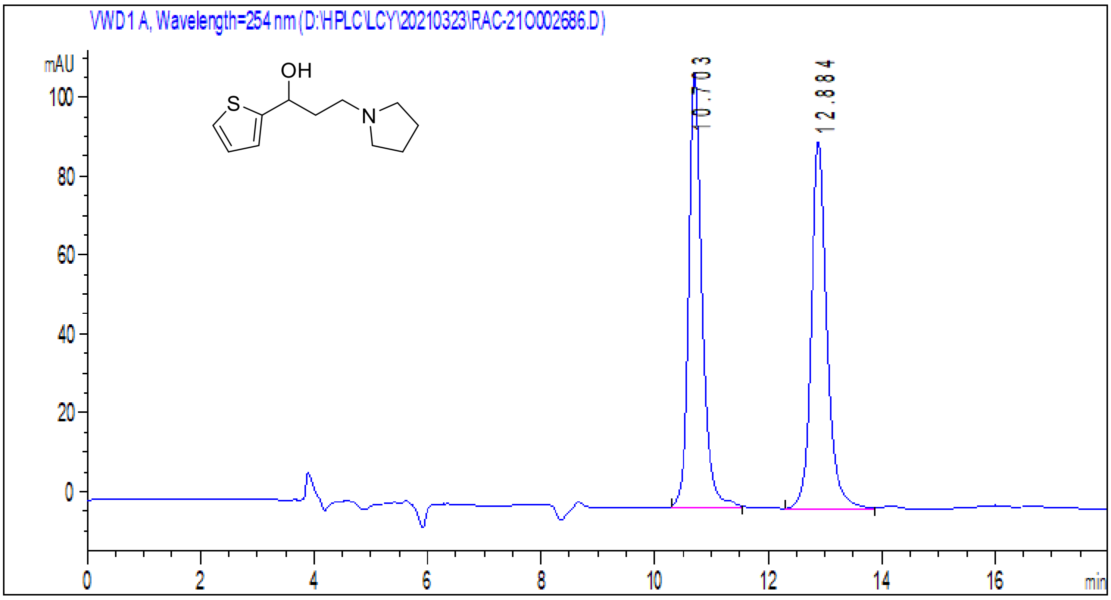

#	Time	Area	Height	Width	Symmetry	Area %
1	10.703	1787.8	110.5	0.2697	0.749	50.098
2	12.884	1780.8	92.9	0.2878	0.77	49.902

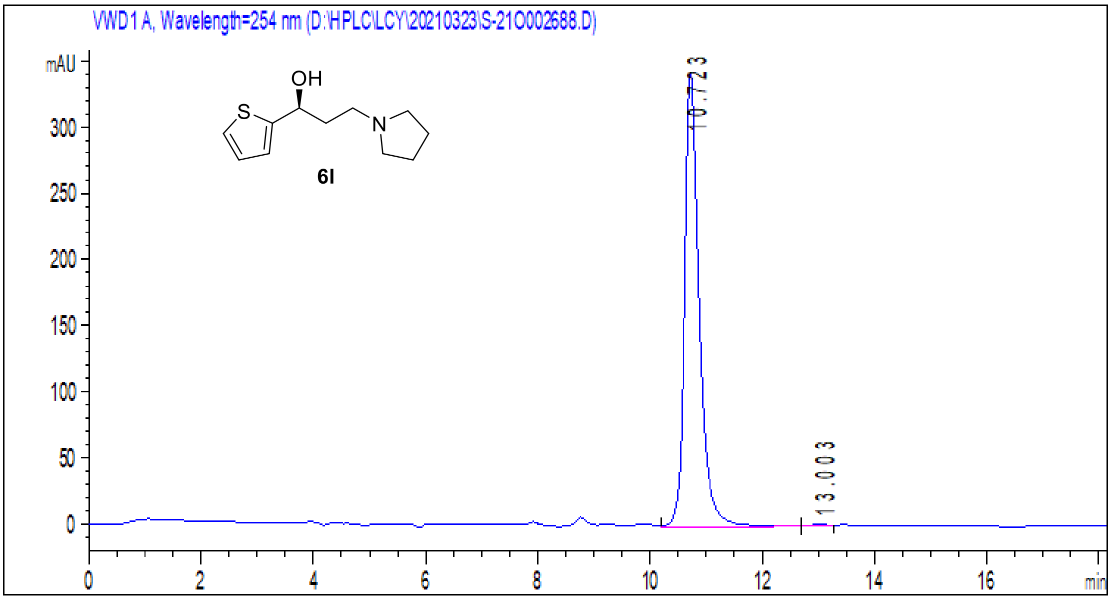

#	Time	Area	Height	Width	Symmetry	Area %
1	10.723	6166.6	342.5	0.2684	0.64	99.473
2	13.003	32.7	1.4	0.3335	0.946	0.527

Supplementary Figure 257. HPLC spectra of **6l**

(*S*)-3-(piperidin-1-yl)-1-(thiophen-2-yl)propan-1-ol (6m)

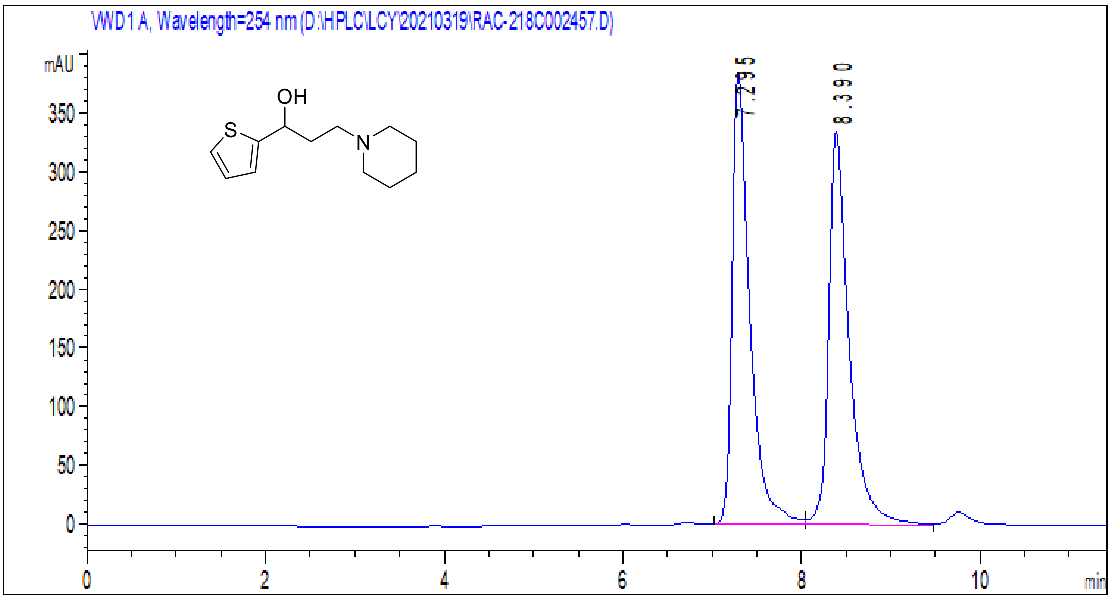

#	Time	Area	Height	Width	Symmetry	Area %
1	7.295	5265.9	384.2	0.2007	0.509	49.852
2	8.39	5297.1	334.3	0.2344	0.548	50.148

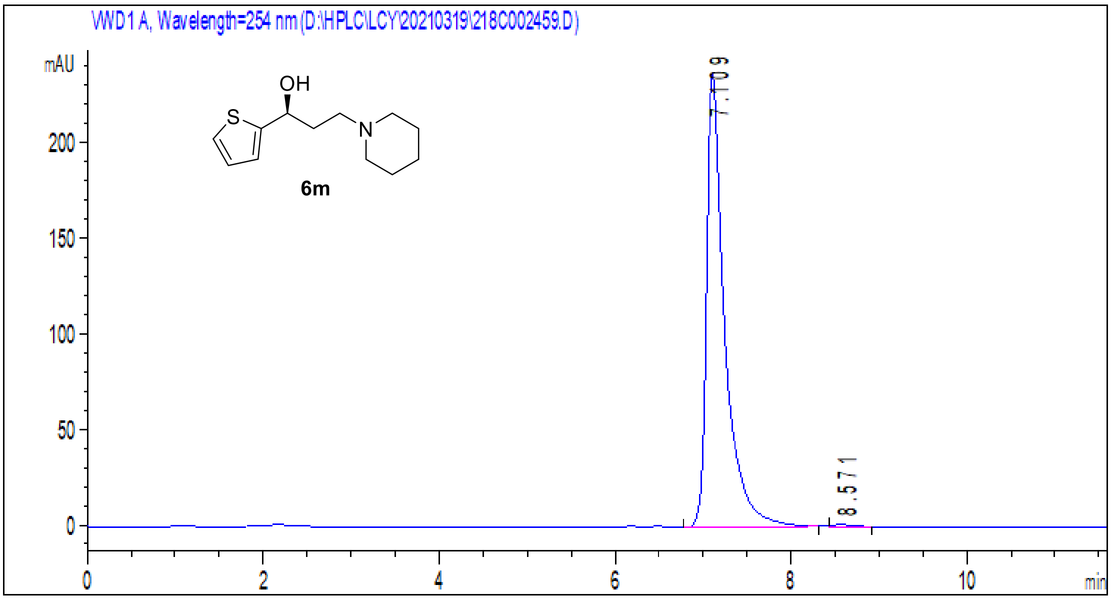

#	Time	Area	Height	Width	Symmetry	Area %
1	7.109	3417.7	238.8	0.2385	0.447	99.430
2	8.571	19.6	1.1	0.2871	0.421	0.570

Supplementary Figure 258. HPLC spectra of **6m**

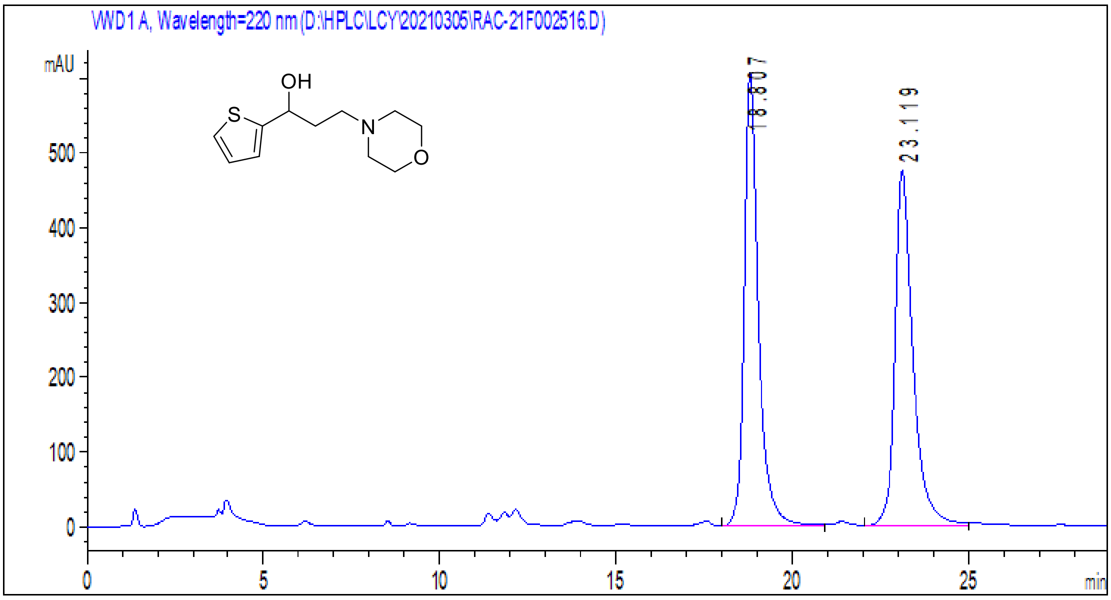

#	Time	Area	Height	Width	Symmetry	Area %
1	18.807	16701.3	605.1	0.4153	0.646	50.099
2	23.119	16635	474.1	0.5259	0.615	49.901

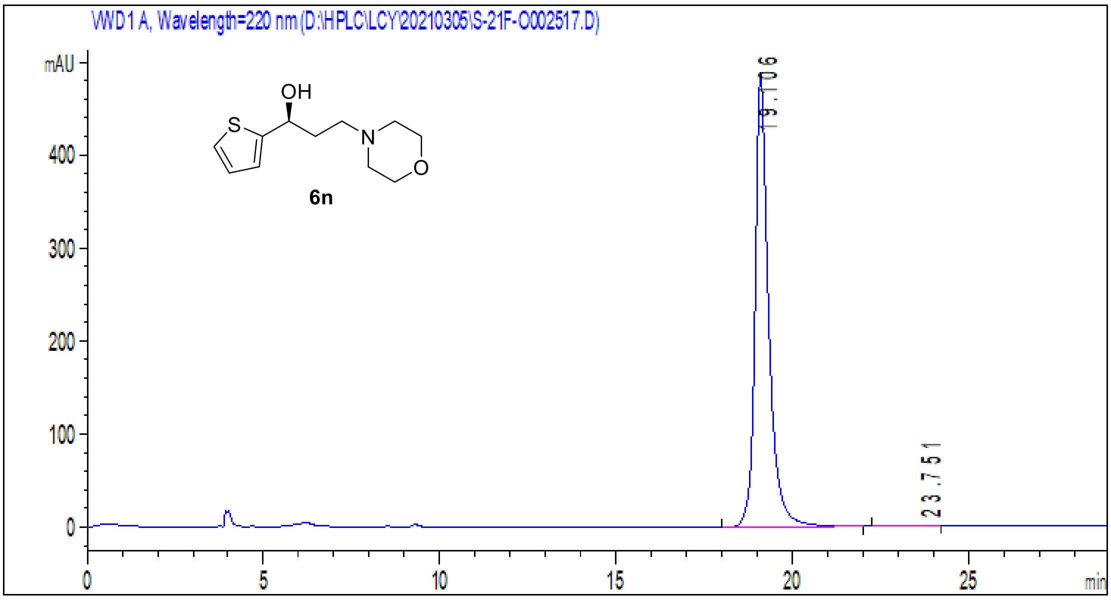

#	Time	Area	Height	Width	Symmetry	Area %
1	19.106	13163.4	486.6	0.3972	0.653	99.649
2	23.751	46.4	1.3	0.5183	0.988	0.351

Supplementary Figure 259. HPLC spectra of **6n**

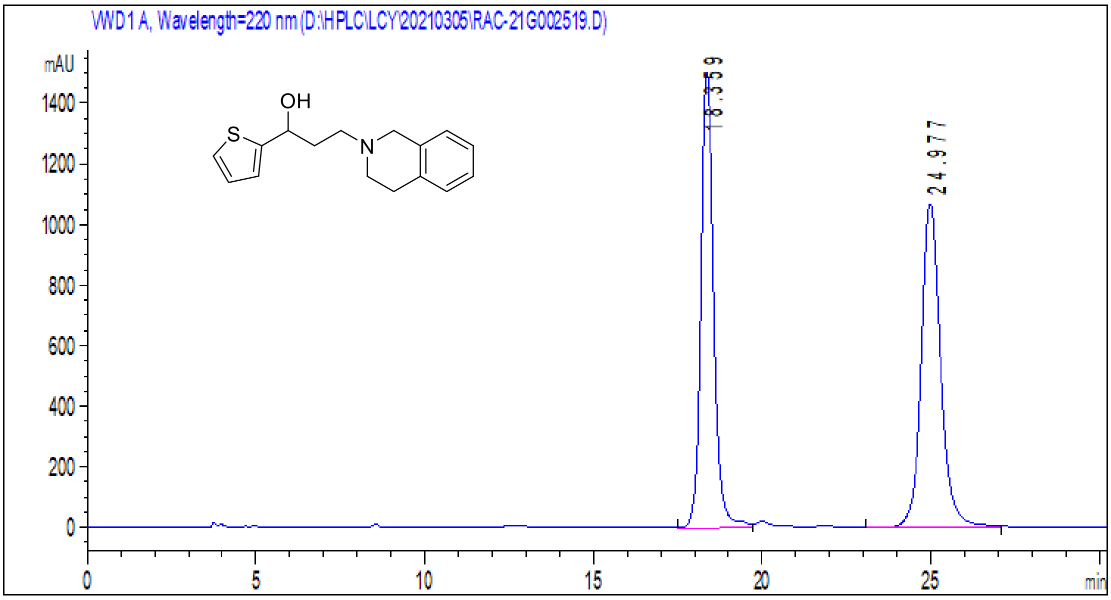

#	Time	Area	Height	Width	Symmetry	Area %
1	18.359	40030.5	1503.7	0.4437	0.808	48.425
2	24.977	42635	1070	0.6641	0.816	51.575

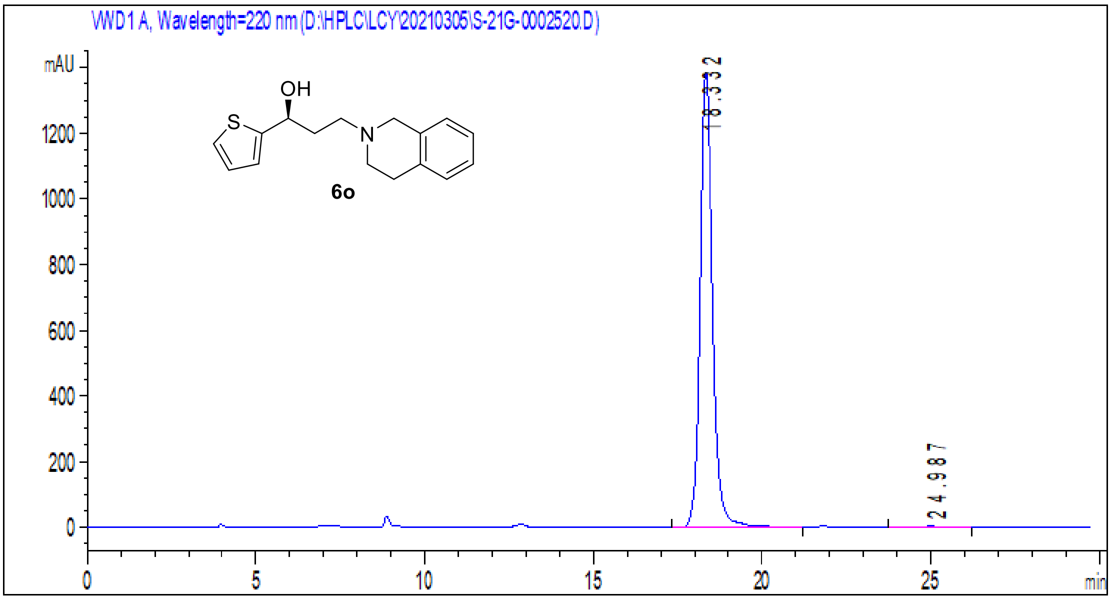

#	Time	Area	Height	Width	Symmetry	Area %
1	18.332	35441.6	1385.1	0.3918	0.778	99.600
2	24.987	142.4	3.5	0.552	0.97	0.400

Supplementary Figure 260. HPLC spectra of **6o**

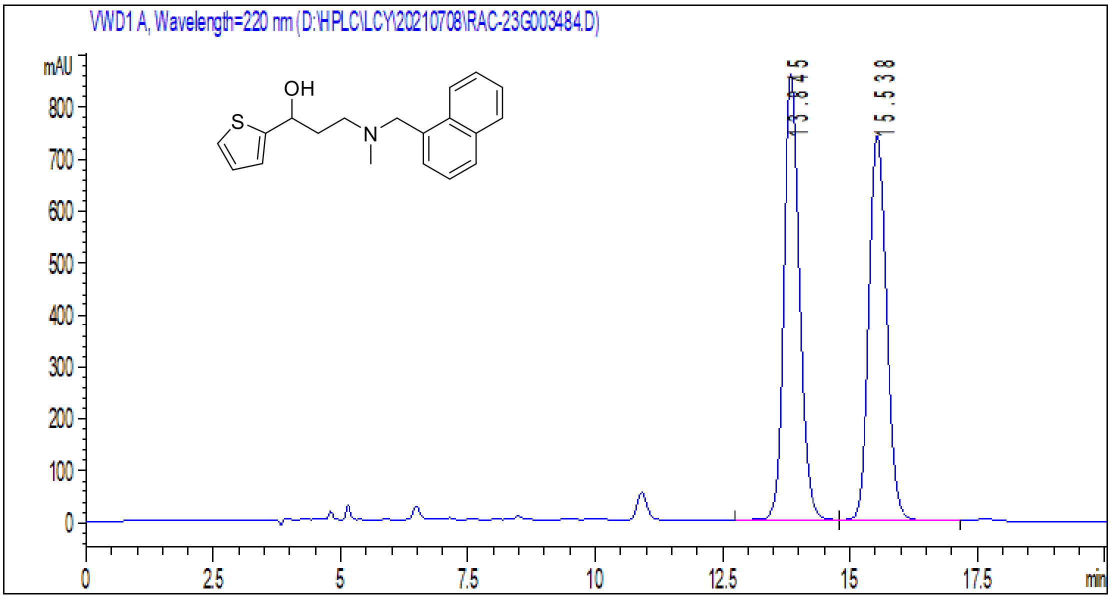

#	Time	Area	Height	Width	Symmetry	Area %
1	13.845	18360.9	858	0.3284	0.8	50.763
2	15.538	17809.1	740.7	0.3767	0.848	49.237

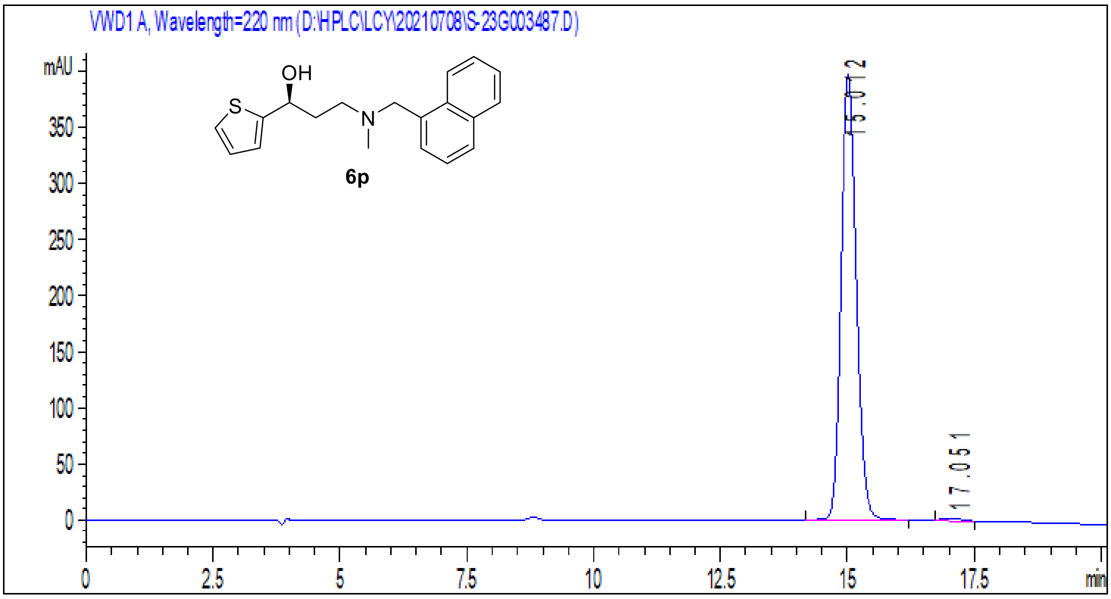

#	Time	Area	Height	Width	Symmetry	Area %
1	15.012	8297.6	397.5	0.3479	0.848	99.241
2	17.051	63.5	2.2	0.4718	0.627	0.759

Supplementary Figure 261. HPLC spectra of **6p**

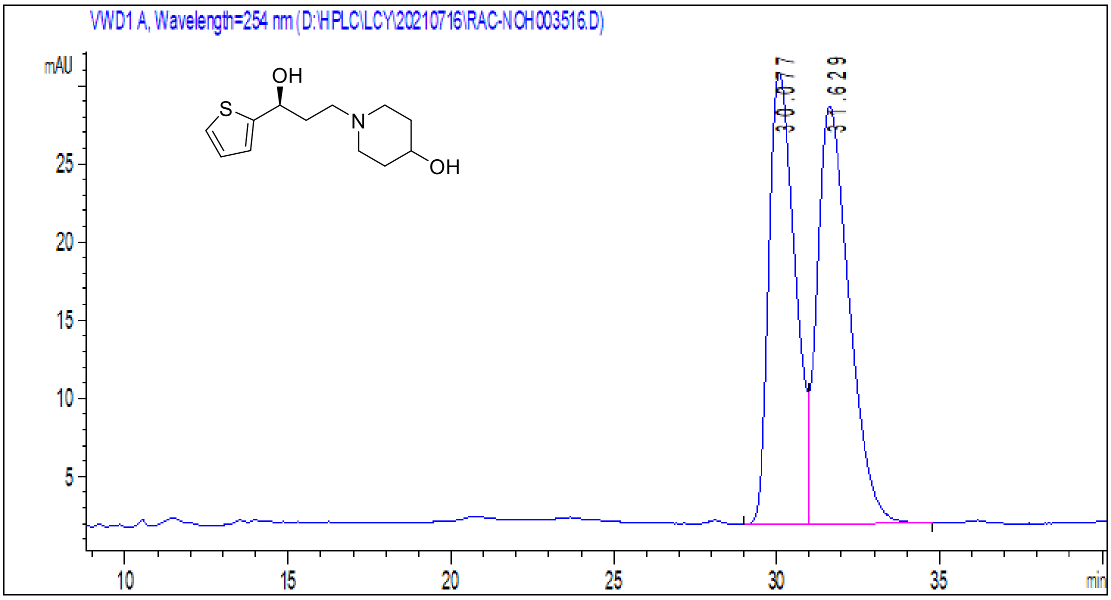

#	Time	Area	Height	Width	Symmetry	Area %
1	30.077	1604	28.9	0.8638	0.625	46.636
2	31.629	1835.5	26.7	1.0032	0.604	53.364

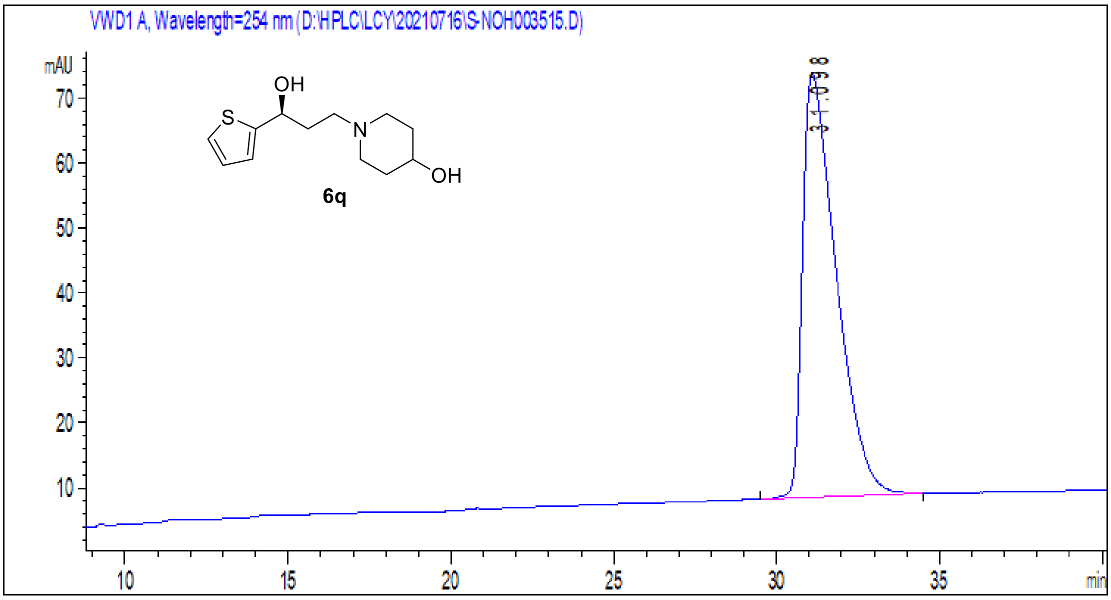

#	Time	Area	Height	Width	Symmetry	Area %
1	31.098	4684	65.2	1.027	0.384	100.000

Supplementary Figure 262. HPLC spectra of **6q**

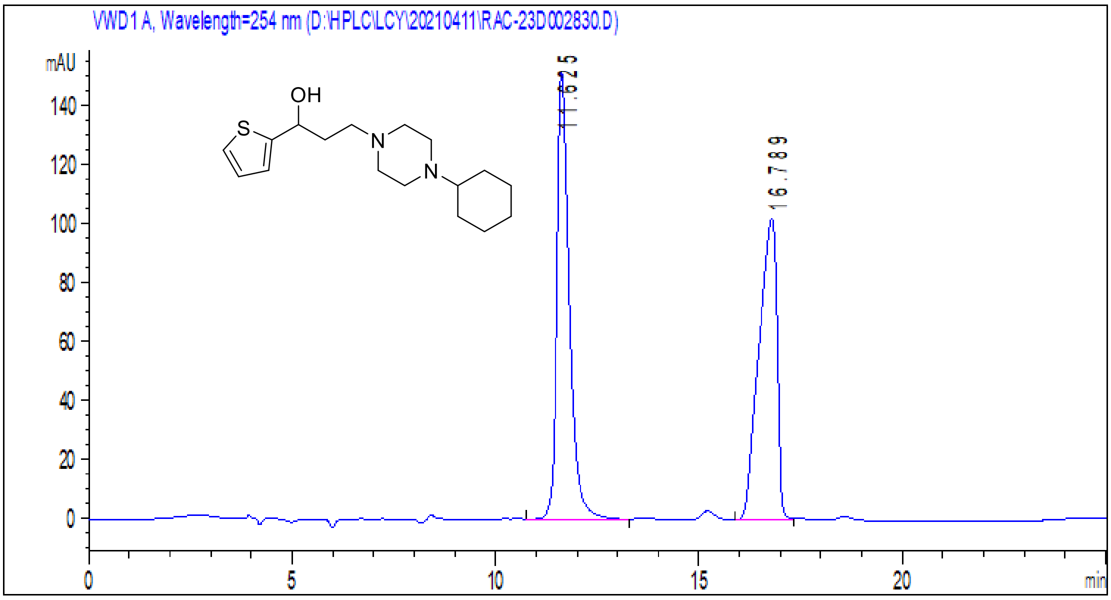

#	Time	Area	Height	Width	Symmetry	Area %
1	11.625	3245.9	151.2	0.3204	0.569	50.495
2	16.789	3182.3	101.9	0.475	2.294	49.505

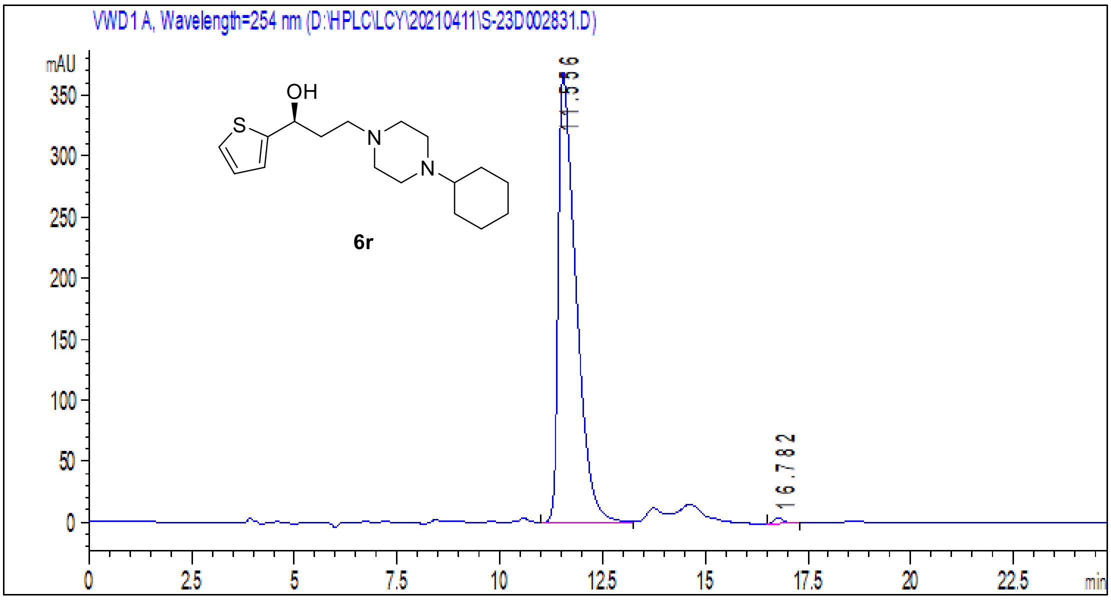

#	Time	Area	Height	Width	Symmetry	Area %
1	11.556	11017.2	368.6	0.4479	0.371	99.319
2	16.782	75.5	4.9	0.2582	0.672	0.681

Supplementary Figure 263. HPLC spectra of **6r**

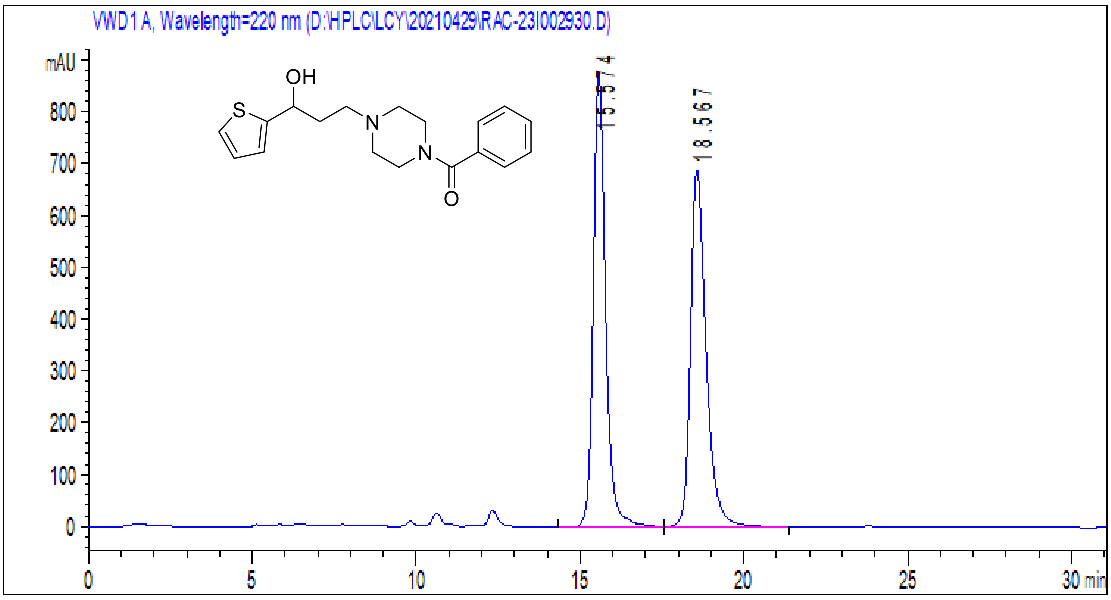

#	Time	Area	Height	Width	Symmetry	Area %
1	15.574	22830.4	877.7	0.3909	0.74	50.086
2	18.567	22752.3	690	0.4955	0.643	49.914

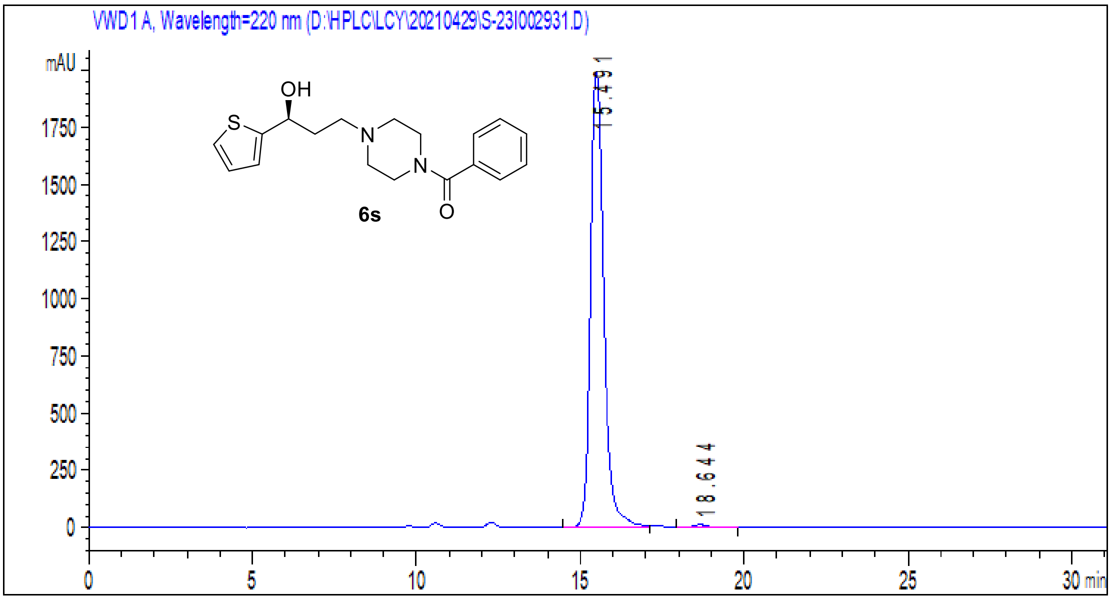

#	Time	Area	Height	Width	Symmetry	Area %
1	15.491	55894.8	1990.5	0.4326	0.706	99.397
2	18.644	338.9	11	0.4642	0.953	0.603

Supplementary Figure 264. HPLC spectra of **6s**

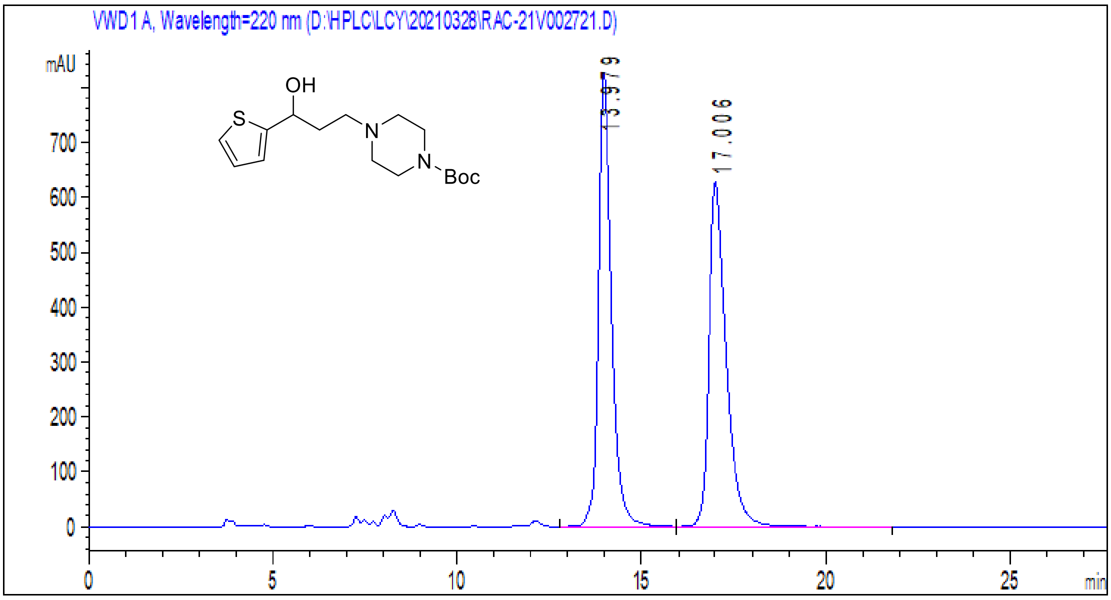

#	Time	Area	Height	Width	Symmetry	Area %
1	13.979	20307.1	825.4	0.3687	0.655	50.016
2	17.006	20294	626.2	0.483	0.544	49.984

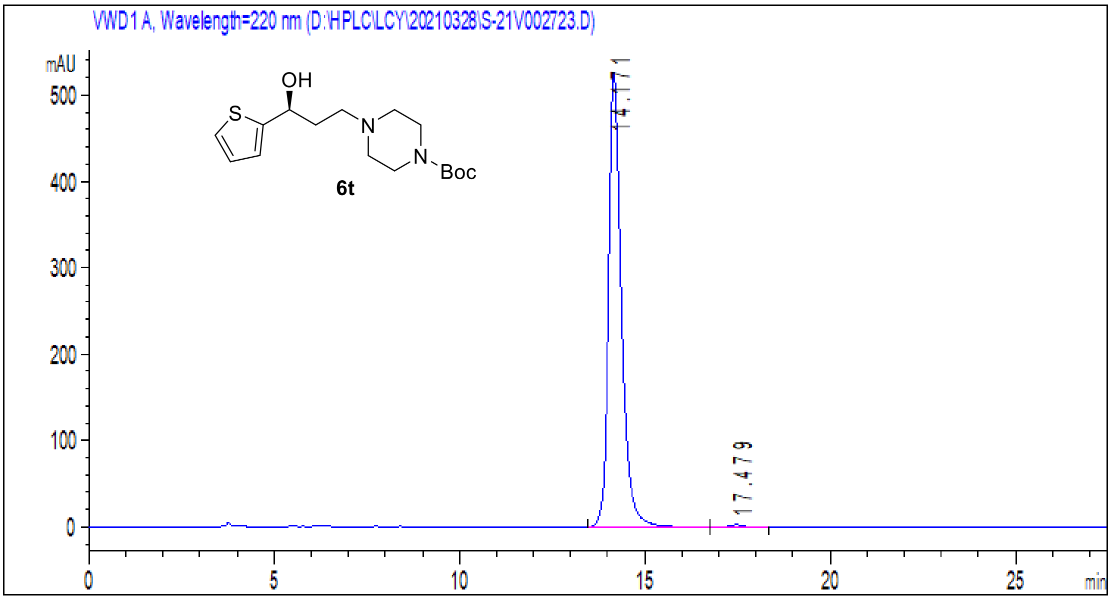

#	Time	Area	Height	Width	Symmetry	Area %
1	14.171	12707.2	525.9	0.3577	0.659	99.264
2	17.479	94.2	3.1	0.5121	1.046	0.736

Supplementary Figure 265. HPLC spectra of **6t**

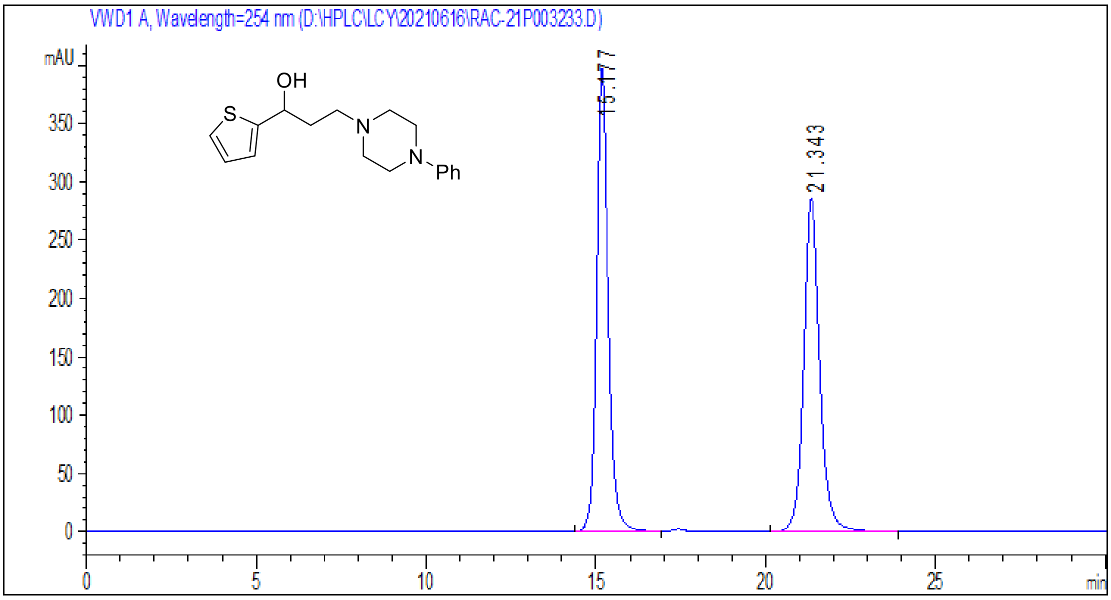

#	Time	Area	Height	Width	Symmetry	Area %
1	15.177	9370.3	397.6	0.3537	0.785	49.950
2	21.343	9389.1	285.9	0.4954	0.846	50.050

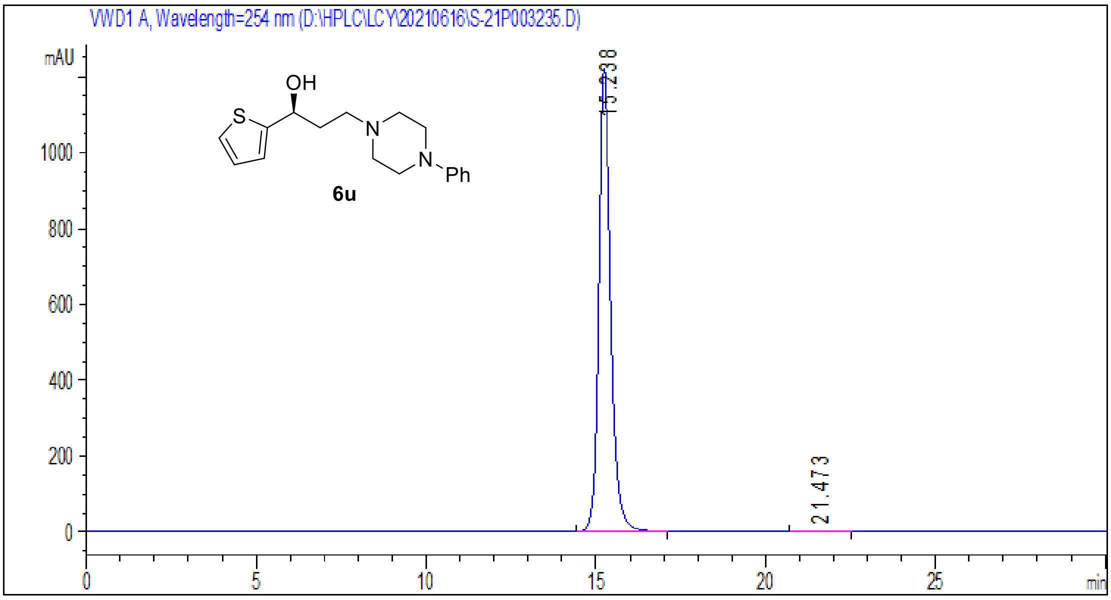

#	Time	Area	Height	Width	Symmetry	Area %
1	15.238	29858.7	1222.8	0.3665	0.739	99.664
2	21.473	100.8	3.1	0.4715	0.85	0.336

Supplementary Figure 266. HPLC spectra of **6u**

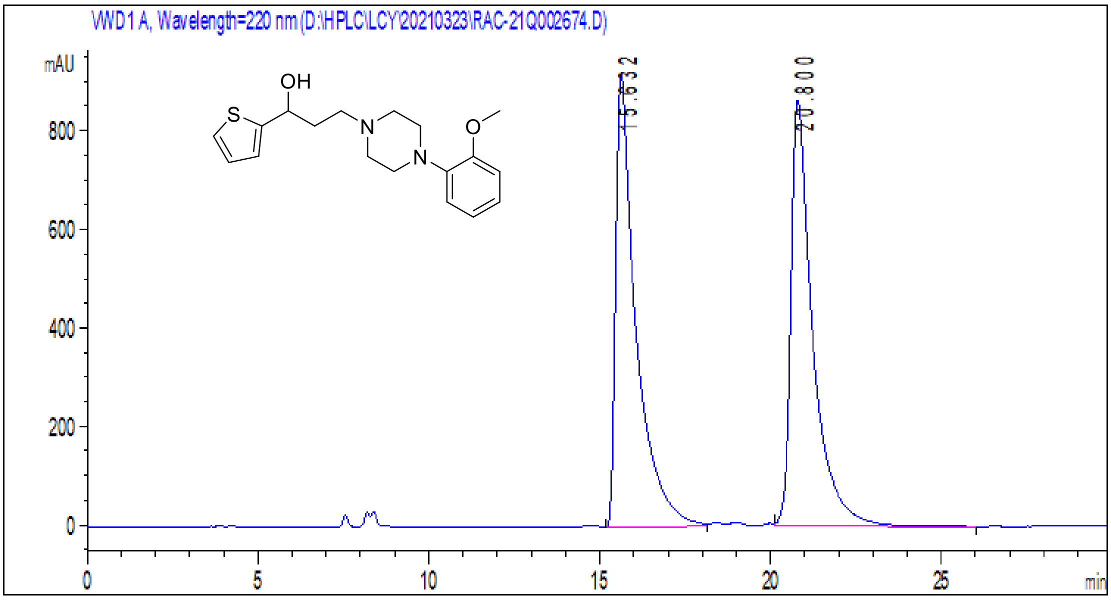

#	Time	Area	Height	Width	Symmetry	Area %
1	15.632	37660.3	920.8	0.5763	0.33	49.718
2	20.8	38088	863.7	0.6369	0.426	50.282

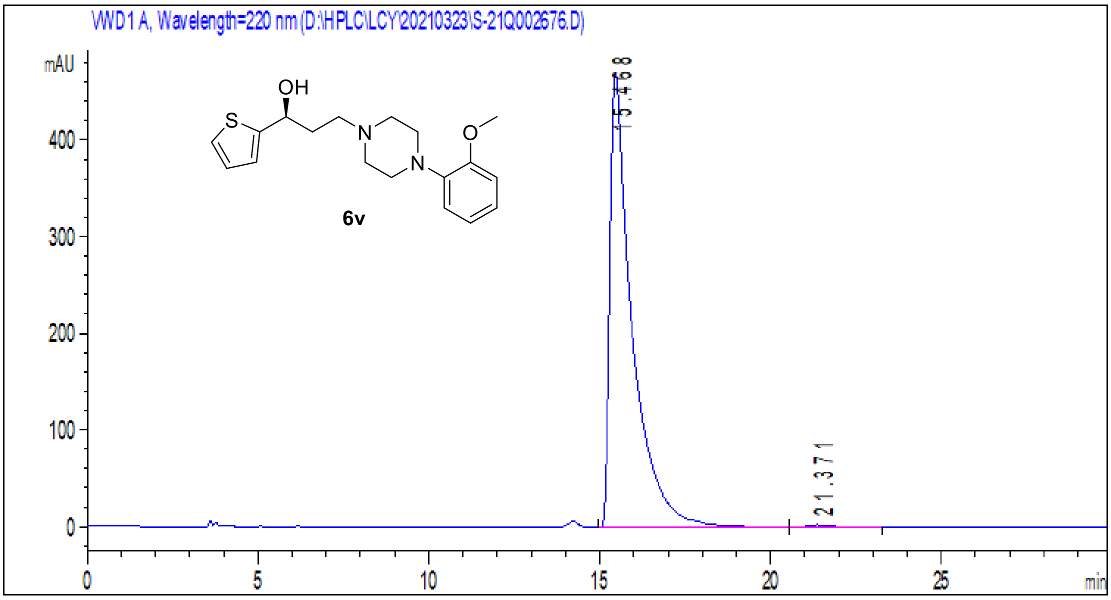

#	Time	Area	Height	Width	Symmetry	Area %
1	15.468	21185.6	470.5	0.6245	0.309	99.372
2	21.371	133.8	2.6	0.7155	0.618	0.628

Supplementary Figure 267. HPLC spectra of **6v**

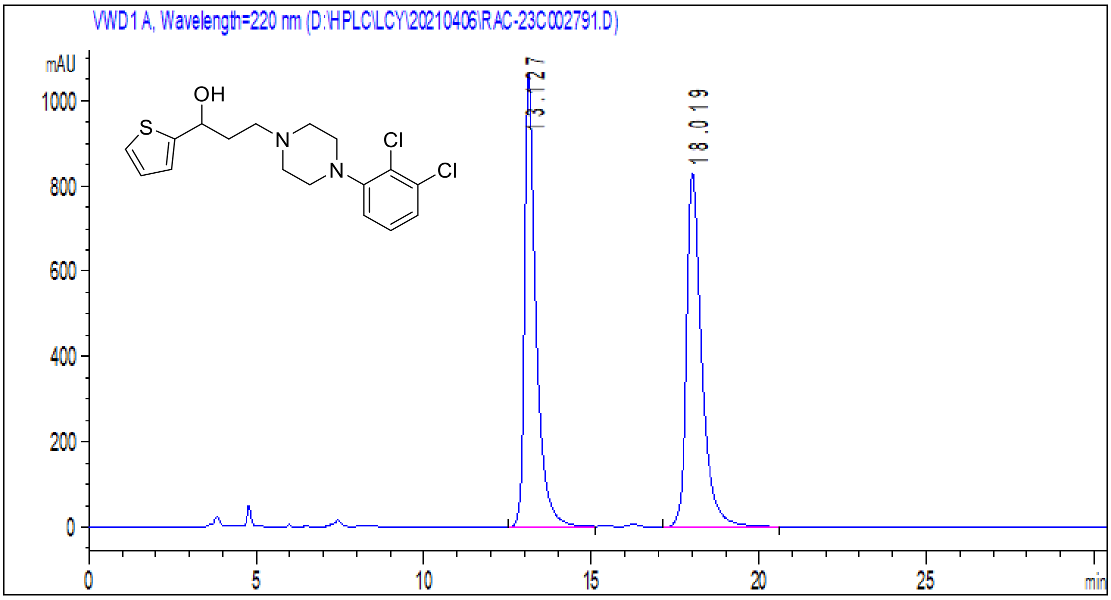

#	Time	Area	Height	Width	Symmetry	Area %
1	13.127	25797.1	1064.5	0.3585	0.505	49.841
2	18.019	25961.4	830	0.464	0.584	50.159

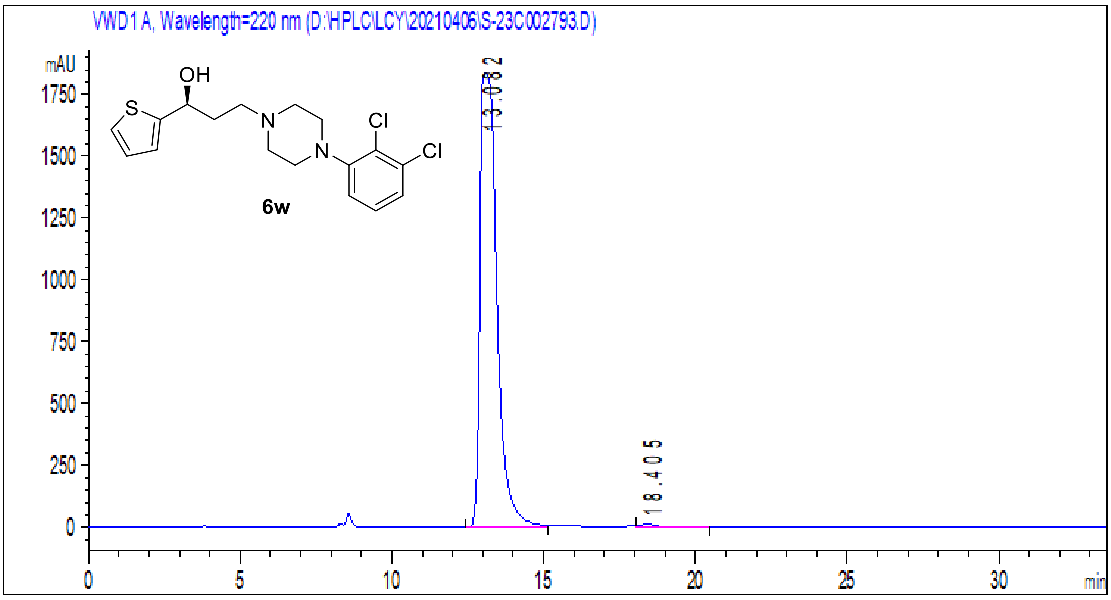

#	Time	Area	Height	Width	Symmetry	Area %
1	13.082	71156	1836.8	0.5977	0.447	99.399
2	18.405	430	13.6	0.4727	0.727	0.601

Supplementary Figure 268. HPLC spectra of **6w**

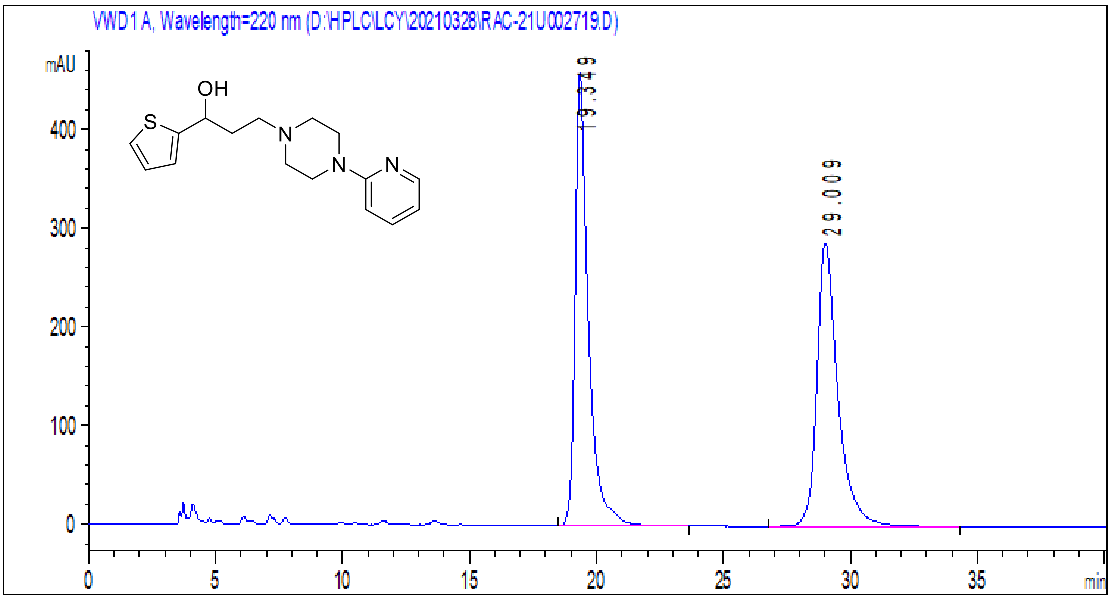

#	Time	Area	Height	Width	Symmetry	Area %
1	19.349	16699.8	458.8	0.5296	0.518	50.272
2	29.009	16518.9	286.8	0.847	0.618	49.728

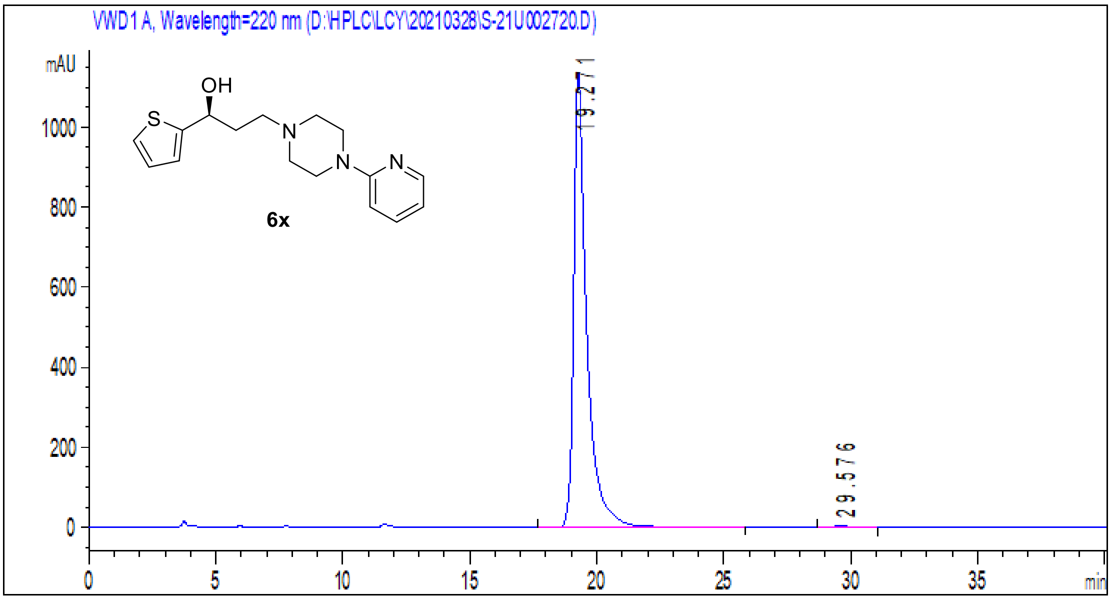

#	Time	Area	Height	Width	Symmetry	Area %
1	19.271	40902.8	1136.9	0.5247	0.478	99.566
2	29.576	178.5	3.4	0.7027	0.655	0.434

Supplementary Figure 269. HPLC spectra of **6x**

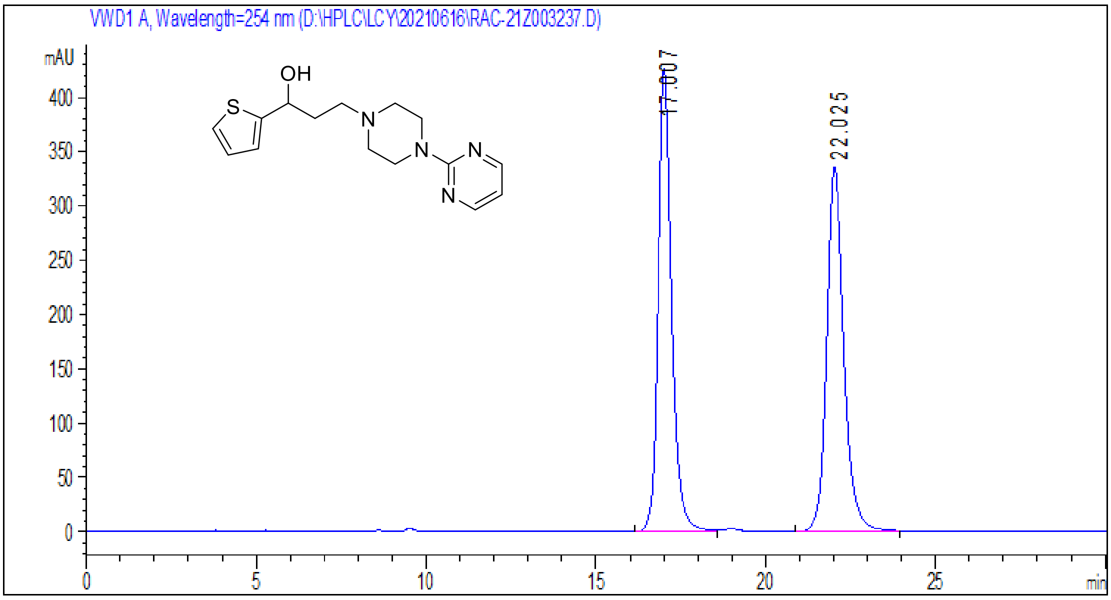

#	Time	Area	Height	Width	Symmetry	Area %
1	17.007	11647.5	426.8	0.4117	0.743	49.806
2	22.025	11738	335.7	0.5275	0.784	50.194

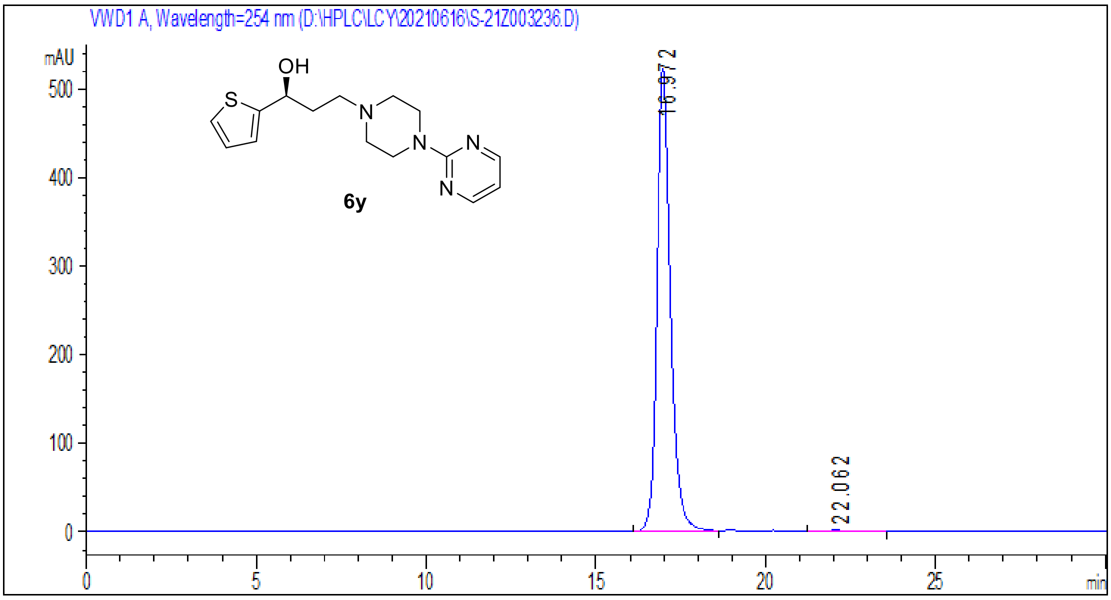

#	Time	Area	Height	Width	Symmetry	Area %
1	16.972	14469.4	523.5	0.4157	0.726	99.634
2	22.062	53.1	1.5	0.5073	0.849	0.366

Supplementary Figure 270. HPLC spectra of **6y**

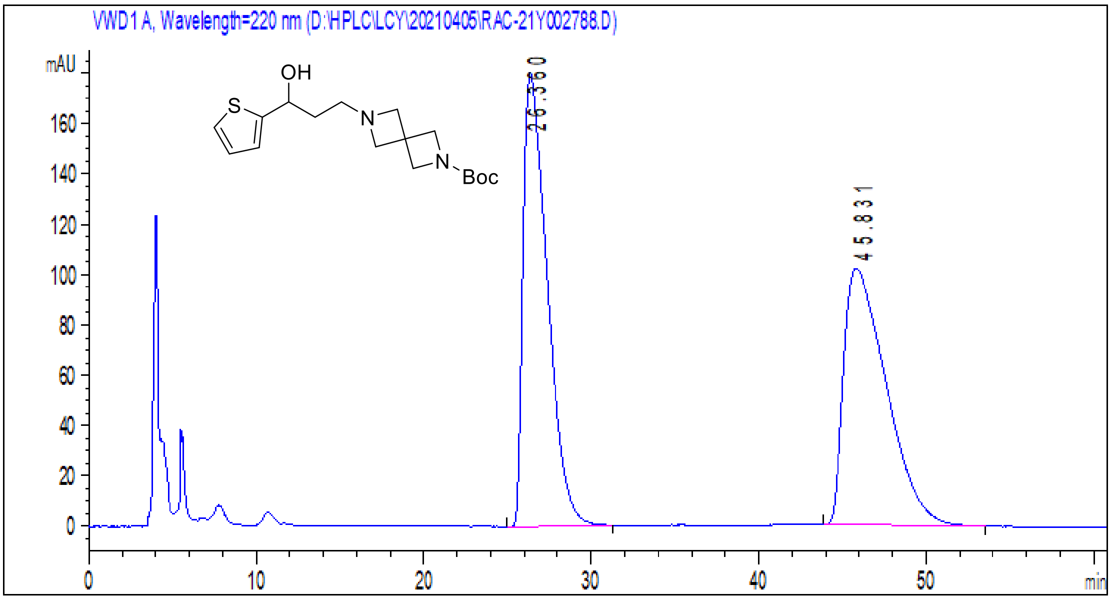

#	Time	Area	Height	Width	Symmetry	Area %
1	26.36	18144.9	180.5	1.5055	0.396	50.283
2	45.831	17940.3	101.7	2.3547	0.39	49.717

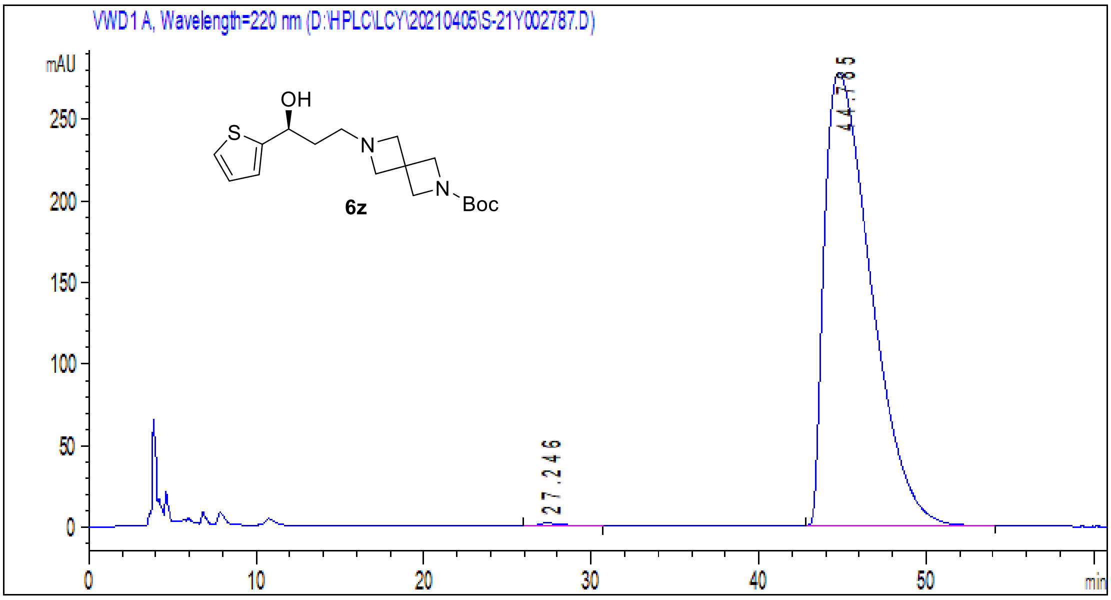

#	Time	Area	Height	Width	Symmetry	Area %
1	27.246	201.8	1.9	1.2545	0.363	0.376
2	44.785	53430.3	277.2	2.6392	0.414	99.624

Supplementary Figure 271. HPLC spectra of **6z**

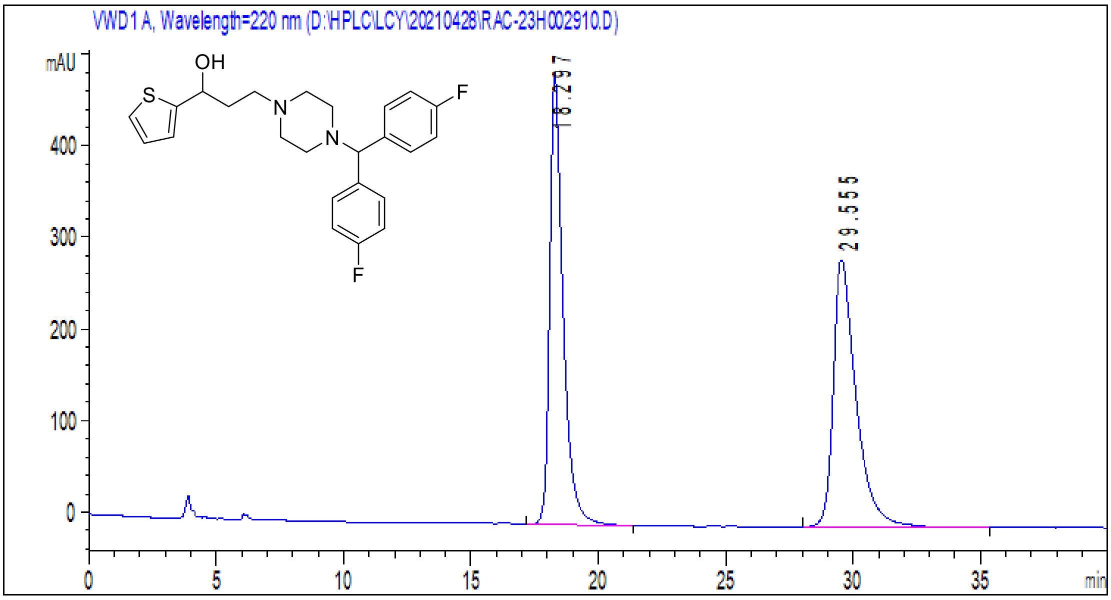

#	Time	Area	Height	Width	Symmetry	Area %
1	18.297	18060.9	493.4	0.5437	0.57	49.822
2	29.555	18189.8	292.4	0.9182	0.535	50.178

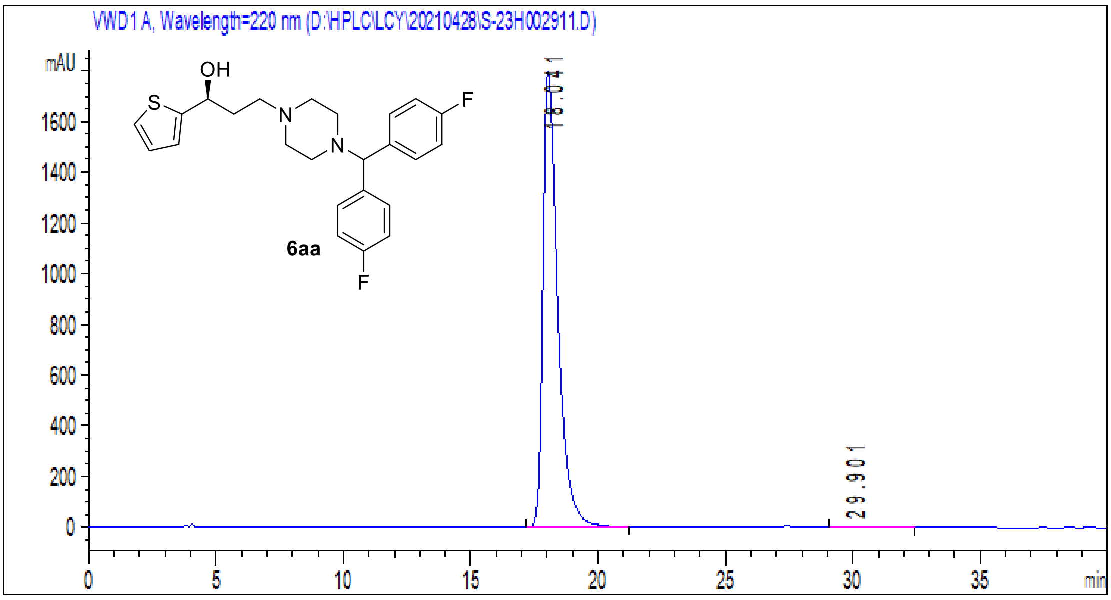

#	Time	Area	Height	Width	Symmetry	Area %
1	18.041	73066	1793.1	0.6095	0.486	99.558
2	29.901	324.2	5	0.9522	0.808	0.442

Supplementary Figure 272. HPLC spectra of **6aa**

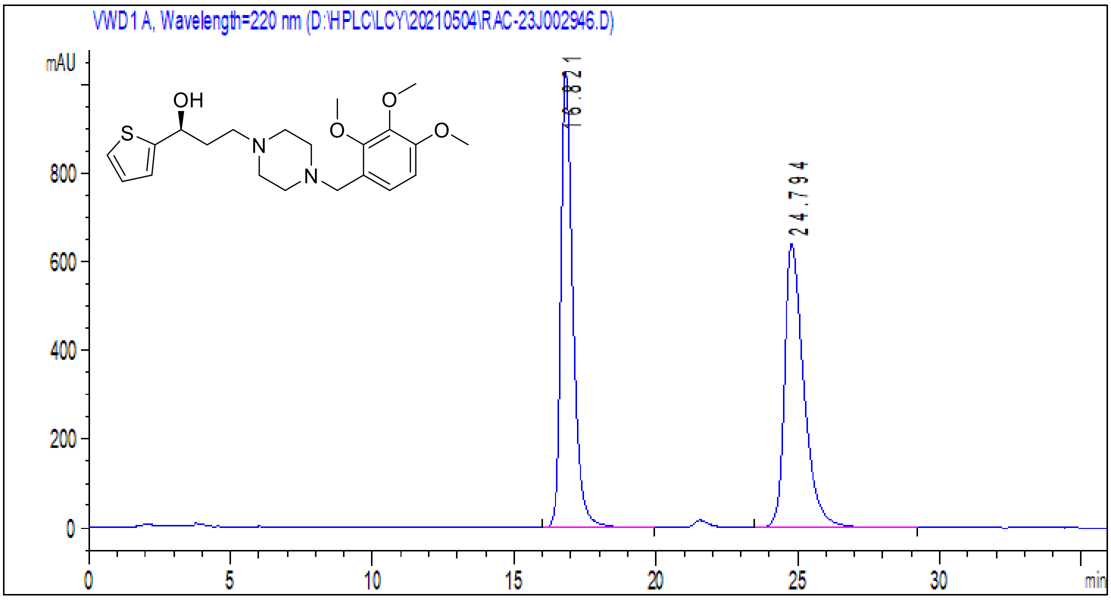

#	Time	Area	Height	Width	Symmetry	Area %
1	16.821	30105.6	1026.8	0.4412	0.626	49.801
2	24.794	30346.1	641.1	0.7097	0.573	50.199

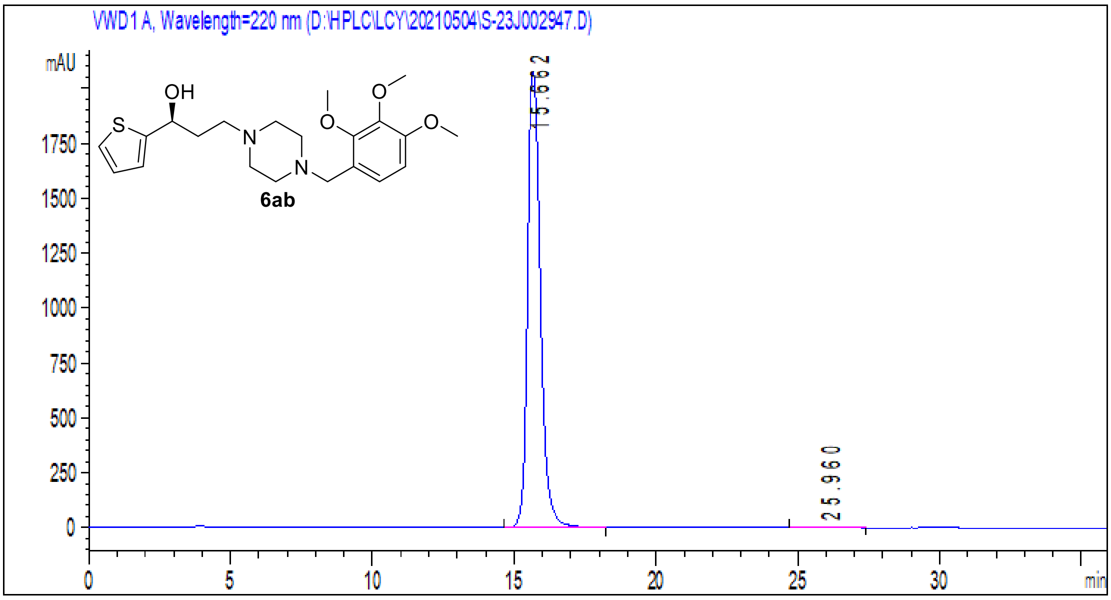

#	Time	Area	Height	Width	Symmetry	Area %
1	15.662	66543.1	2074.6	0.4967	0.673	99.935
2	25.96	43	5.4E-1	1.0088	1.779	0.065

Supplementary Figure 273. HPLC spectra of **6ab**

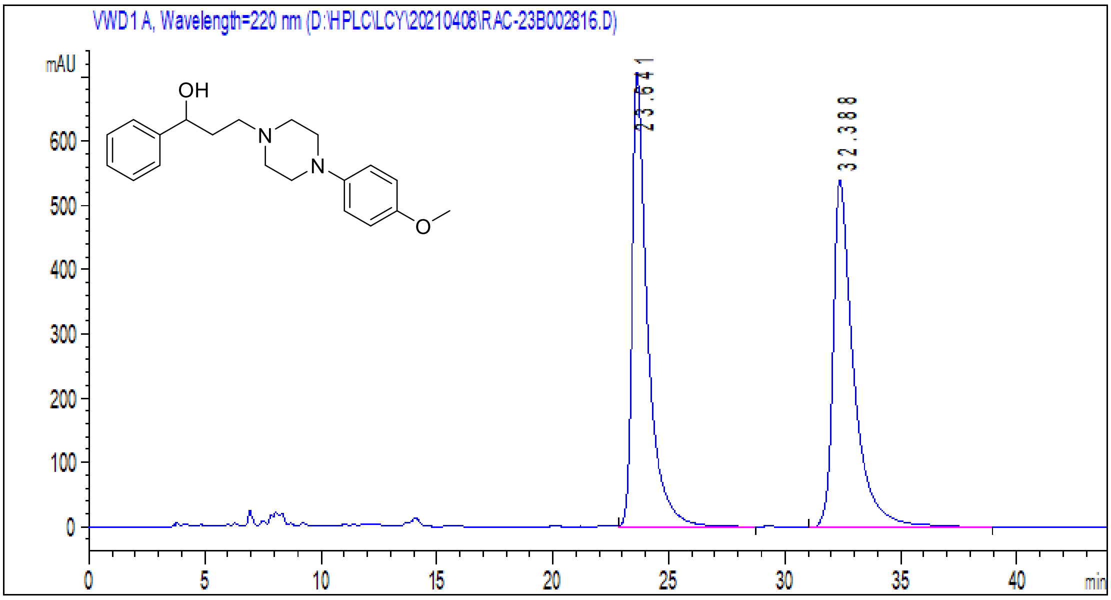

#	Time	Area	Height	Width	Symmetry	Area %
1	23.641	32813.5	706.8	0.6699	0.442	49.562
2	32.388	33393.9	540.9	0.8951	0.473	50.438

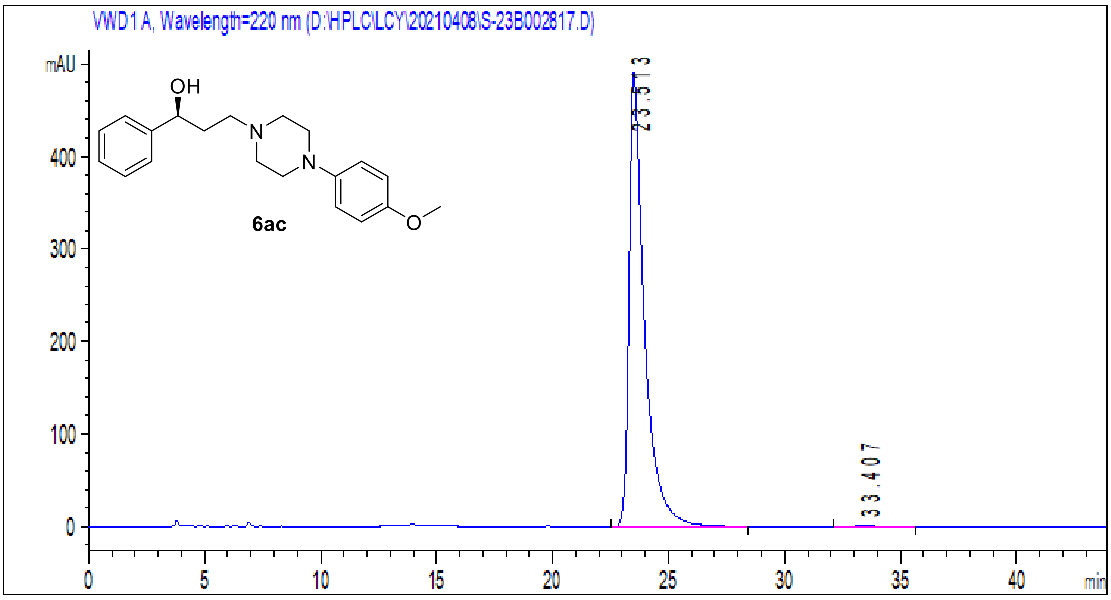

#	Time	Area	Height	Width	Symmetry	Area %
1	23.513	22645.5	490.7	0.6608	0.427	99.470
2	33.407	120.7	1.8	0.8429	0.61	0.530

Supplementary Figure 274. HPLC spectra of **6ac**

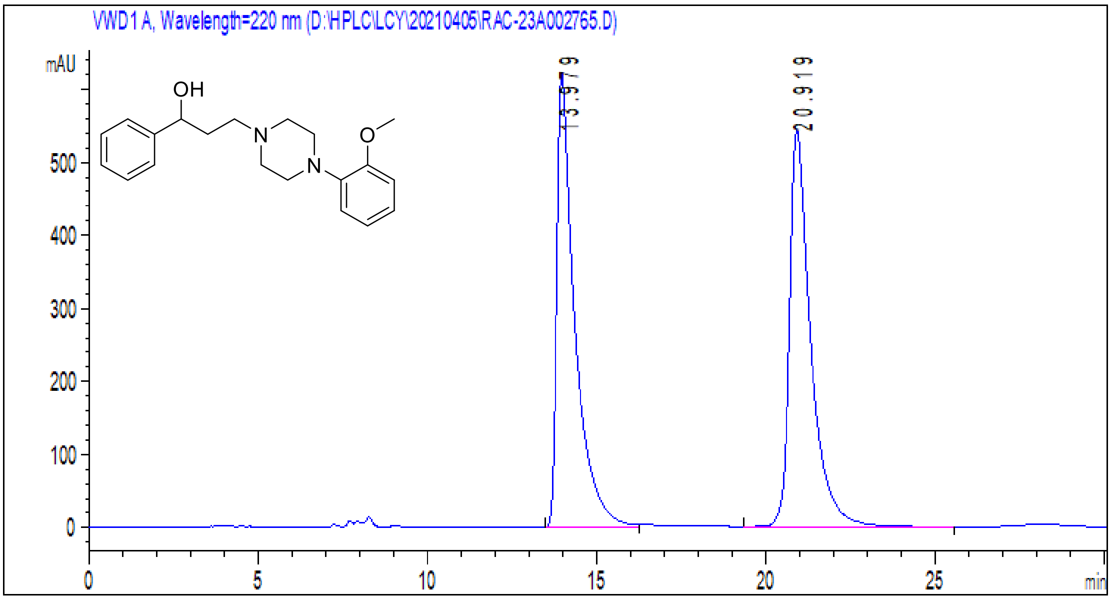

#	Time	Area	Height	Width	Symmetry	Area %
1	13.979	22801.2	621.7	0.5212	0.361	49.210
2	20.919	23533.2	543.3	0.6278	0.5	50.790

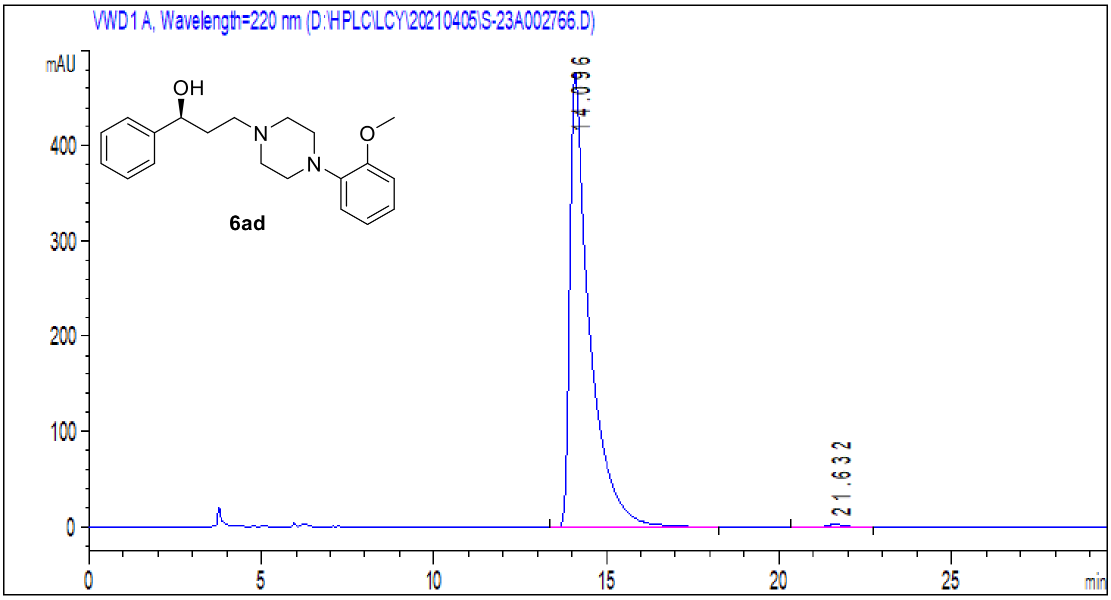

#	Time	Area	Height	Width	Symmetry	Area %
1	14.096	18871.7	476.5	0.5553	0.335	99.368
2	21.632	120	3	0.5958	0.811	0.632

Supplementary Figure 275. HPLC spectra of **6ad**

2.6. Crystal structure of 2a

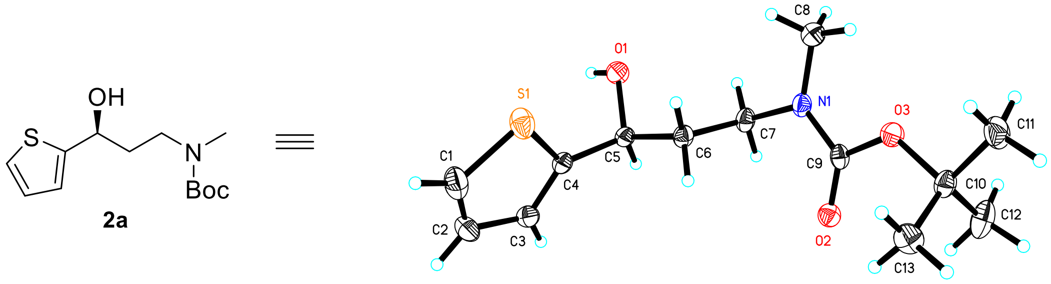

CCDC 2116544

Supplementary Figure 276. ORTEP Drawing of **2a**

Crystal data and structure refinement for **2a**

Identification code	2116544	
Empirical formula	C13 H21 N O3 S	
Formula weight	271.37	
Temperature	213(2) K	
Wavelength	0.71073 Å	
Crystal system	Orthorhombic	
Space group	P 21 21 21	
Unit cell dimensions	a = 6.2613(3) Å	a= 90°.
	b = 12.7030(5) Å	b= 90°.
	c = 18.3236(8) Å	g = 90°.
Volume	1457.41(11) $Å^3$	
Z	4	
Density (calculated)	1.237 Mg/m^3	
Absorption coefficient	0.223 mm^{-1}	
F(000)	584	
Crystal size	0.180 x 0.160 x 0.110 mm^3	
Theta range for data collection	2.741 to 25.991°.	
Index ranges	-7<=h<=7, -13<=k<=15, -15<=l<=22	
Reflections collected	7146	
Independent reflections	2853 [R(int) = 0.0591]	
Completeness to theta = 25.242°	99.1 %	
Absorption correction	Semi-empirical from equivalents	
Max. and min. transmission	0.7456 and 0.5131	

Refinement method	Full-matrix least-squares on F^2
Data / restraints / parameters	2853 / 0 / 169
Goodness-of-fit on F^2	1.023
Final R indices [I>2sigma(I)]	R1 = 0.0391, wR2 = 0.0980
R indices (all data)	R1 = 0.0454, wR2 = 0.1041
Absolute structure parameter	0.00(5)
Extinction coefficient	0.024(8)
Largest diff. peak and hole	0.211 and -0.186 e.Å^{-3}

3. Supplementary References

1. Kawamura, K., Fukuzawa, H. & Hayashi, M. Novel *N,N,P*-tridentate ligands for the highly enantioselective Copper-catalyzed 1,4-addition of dialkylzincs to enones, *Org. Lett.* **10**, 3509–3512 (2008).
2. Lockhart, Z. & Knipe, P. C. Conformationally programmable chiral foldamers with compact and extended domains controlled by monomer structure. *Angew. Chem. Int. Ed.* **57**, 8478–8482 (2018).
3. Nie, H., Zhou, G., Wang, Q., Chen, W. & Zhang, S. Asymmetric hydrogenation of aromatic ketones using an iridium(I) catalyst containing ferrocene-based P–N–N tridentate ligands. *Tetrahedron: Asymmetry* **24**, 1567–1571 (2013).
4. Nie, H., Yao, L., Li, B., Zhang, S. & Chen, W. Very simple and highly modular synthesis of ferrocene-based chiral phosphines with a wide variety of substituents at the phosphorus atom(s). *Organometallics* **33**, 2109–2114 (2014).
5. Liang, Z., Yang, T., Gu, G., Dang, L. & Zhang, X. Scope and mechanism on Iridium-f-amphamide catalyzed asymmetric hydrogenation of ketones. *Chin. J. Chem.* **36**, 851–856 (2018).
6. Ling, F. et al. Development of ferrocene-based diamine-phosphine-sulfonamide ligands for Iridium-catalyzed asymmetric hydrogenation of ketones. *J. Org. Chem.* **83**, 10749–10761 (2018).
7. Liu, D., Gao, W., Wang, C. & Zhang, X. Practical synthesis of enantiopure γ-amino alcohols by Rhodium-catalyzed asymmetric hydrogenation of β-secondary-amino ketones**.** *Angew. Chem. Int. Ed.* **44**, 1687–1689 (2005).
8. Wang, J. Liu, D., Liu, Y. & Zhang, W. Asymmetric hydrogenation of β-amino ketones with the bimetallic complex RuPHOX-Ru as the chiral catalyst. *Org. Biomol. Chem.* **11**, 3855–3861 (2013).

9. Chung, F., Tisné, C., Lecourt, T., Dardel, F. & Micouin, L. NMR-guided fragment-based approach for the design of $tRNA^{Lys3}$ ligands. *Angew. Chem. Int. Ed.* **46**, 4489–4491 (2007).
10. He, S. Z., Li, X. M., Dai, J. & Yan, M. Synthesis of antidepressant duloxetine via asymmetric transfer hydrogenation. *Chin. Chem. Lett.* **19**, 23–25 (2008).
11. Teo, W. J. & Ge, S. Cobalt-catalyzed diborylation of 1,1-disubstituted vinylarenes: a practical route to branched *gem*-bis(boryl)alkanes. *Angew. Chem. Int. Ed.* **57**, 1654-1658. (2018).
